# Supplementary figures and images for: An updated census of the maize TIFY family
Source: PLoS One. 2021 Feb 23;16(2):e0247271. doi: 10.1371/journal.pone.0247271 (PMC7901733; doi:10.1371/journal.pone.0247271)

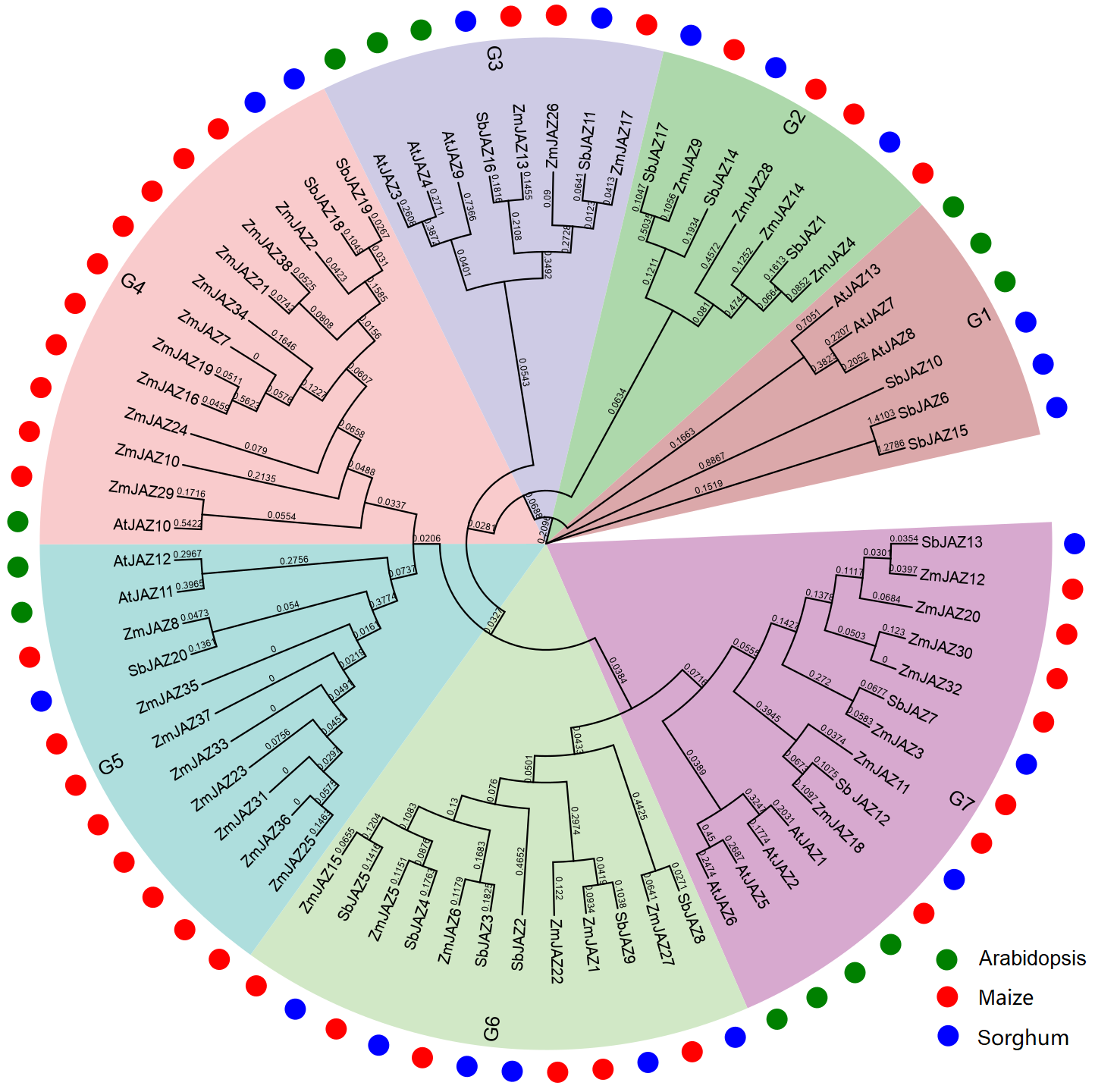

Supplement: S1 Fig — The tree was constructed by software Mega7, using the maximum likelihood method with a bootstrap test of 1000 replicates and all the amino acid sequences of JAZ proteins of the three species were aligned with online software Muscle. (PNG) [file pone.0247271.s001.png]

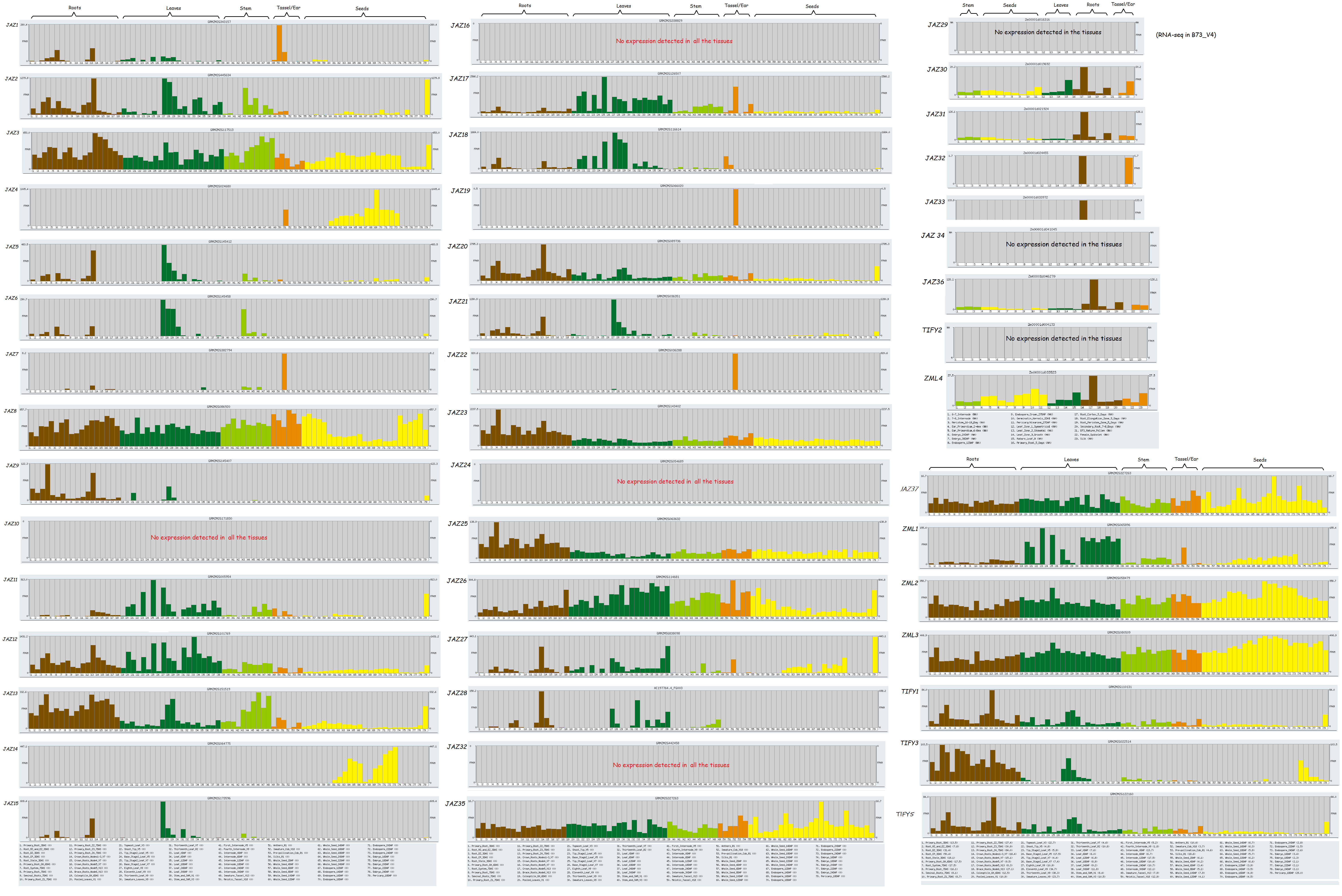

Supplement: S2 Fig — The expression data was downloaded from www.maizegdb.org. (TIF) [file pone.0247271.s002.tif]

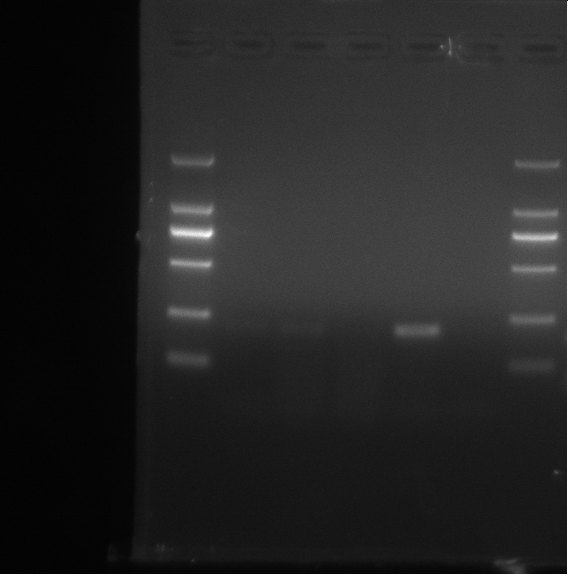

Supplement: S1 File — (ZIP) [file pone.0247271.s007.zip › JA treat_ZmJAZ9.png]

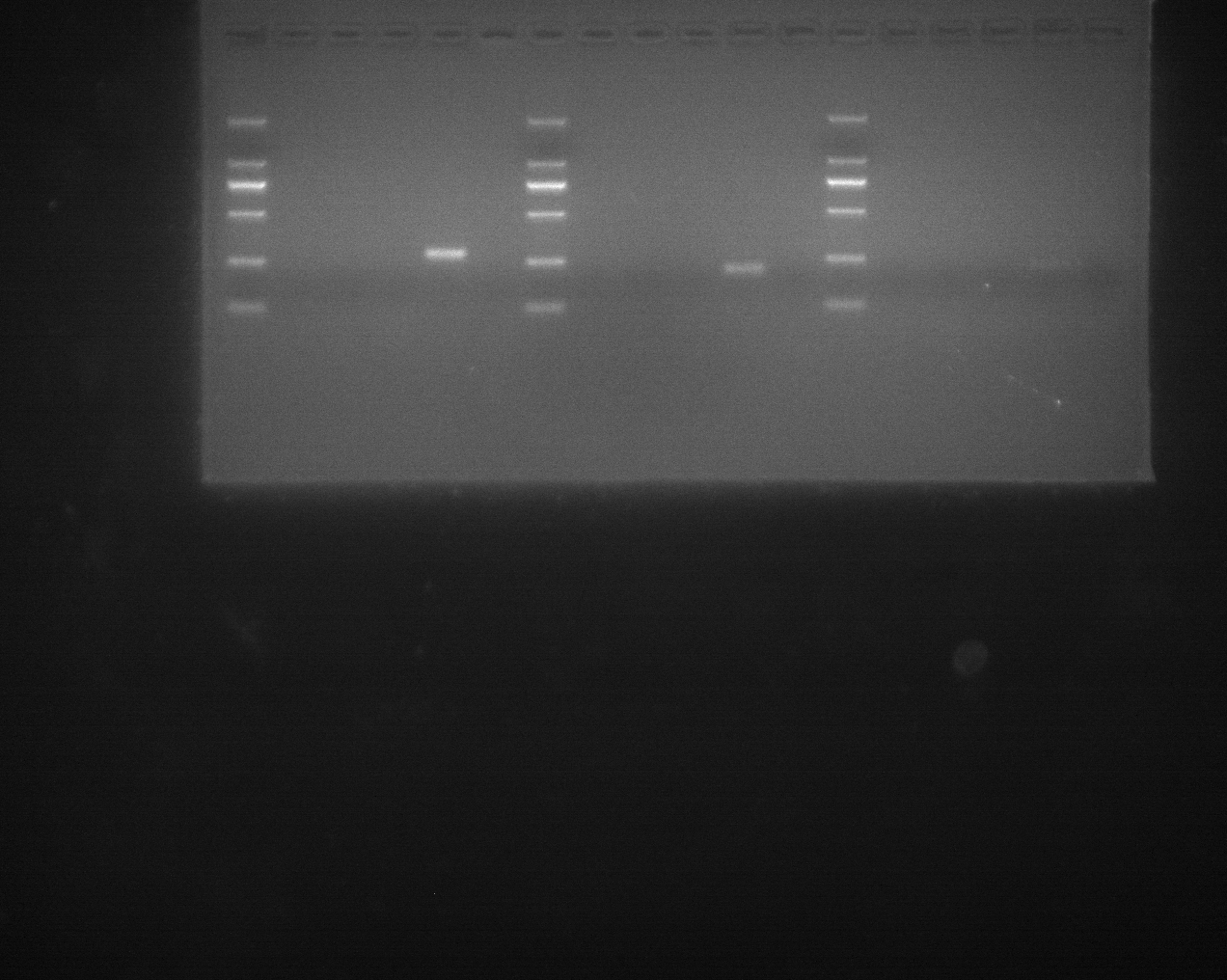

Supplement: S1 File — (ZIP) [file pone.0247271.s007.zip › JA treat_JAZ5 JAZ6.tif]

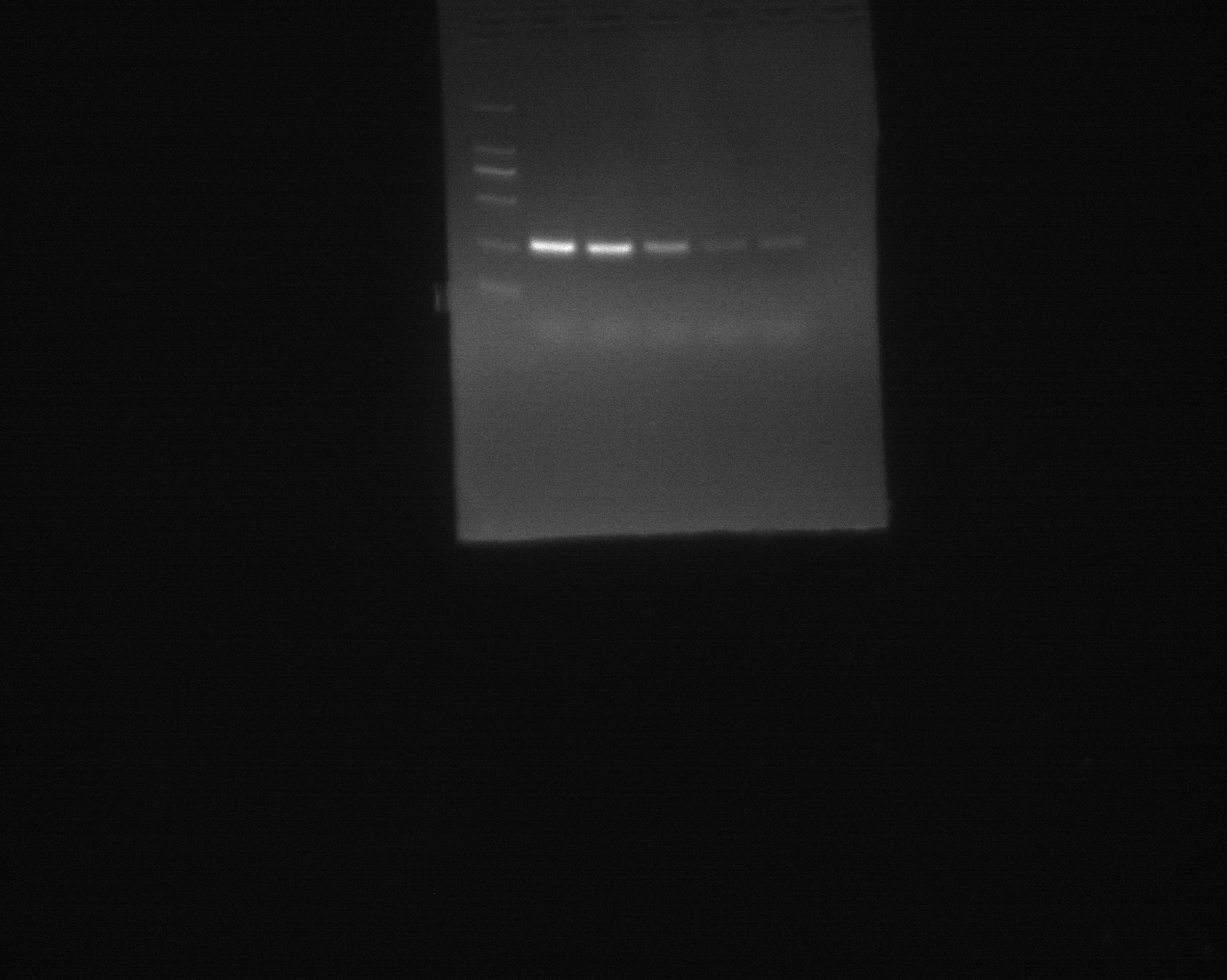

Supplement: S1 File — (ZIP) [file pone.0247271.s007.zip › JA treat_ZMJAZ17.tif]

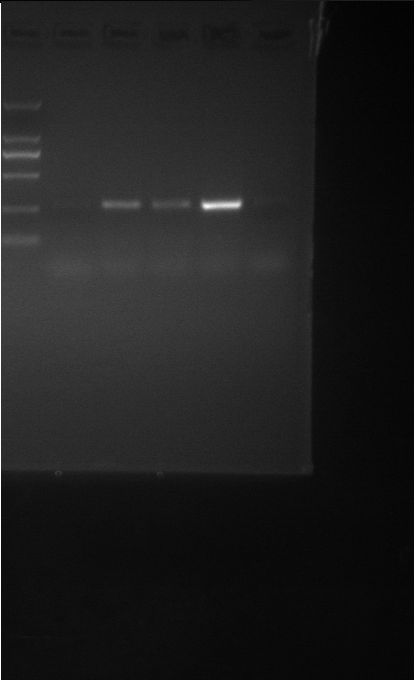

Supplement: S1 File — (ZIP) [file pone.0247271.s007.zip › JA treat_ZmJAZ11.png]

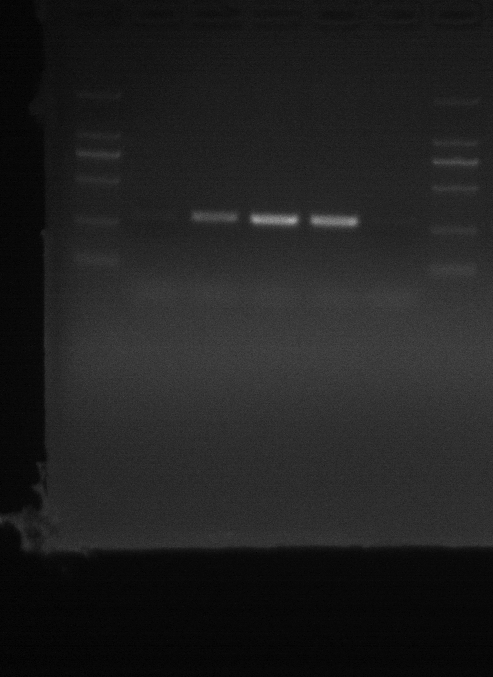

Supplement: S1 File — (ZIP) [file pone.0247271.s007.zip › JA treat_ZmJAZ12.png]

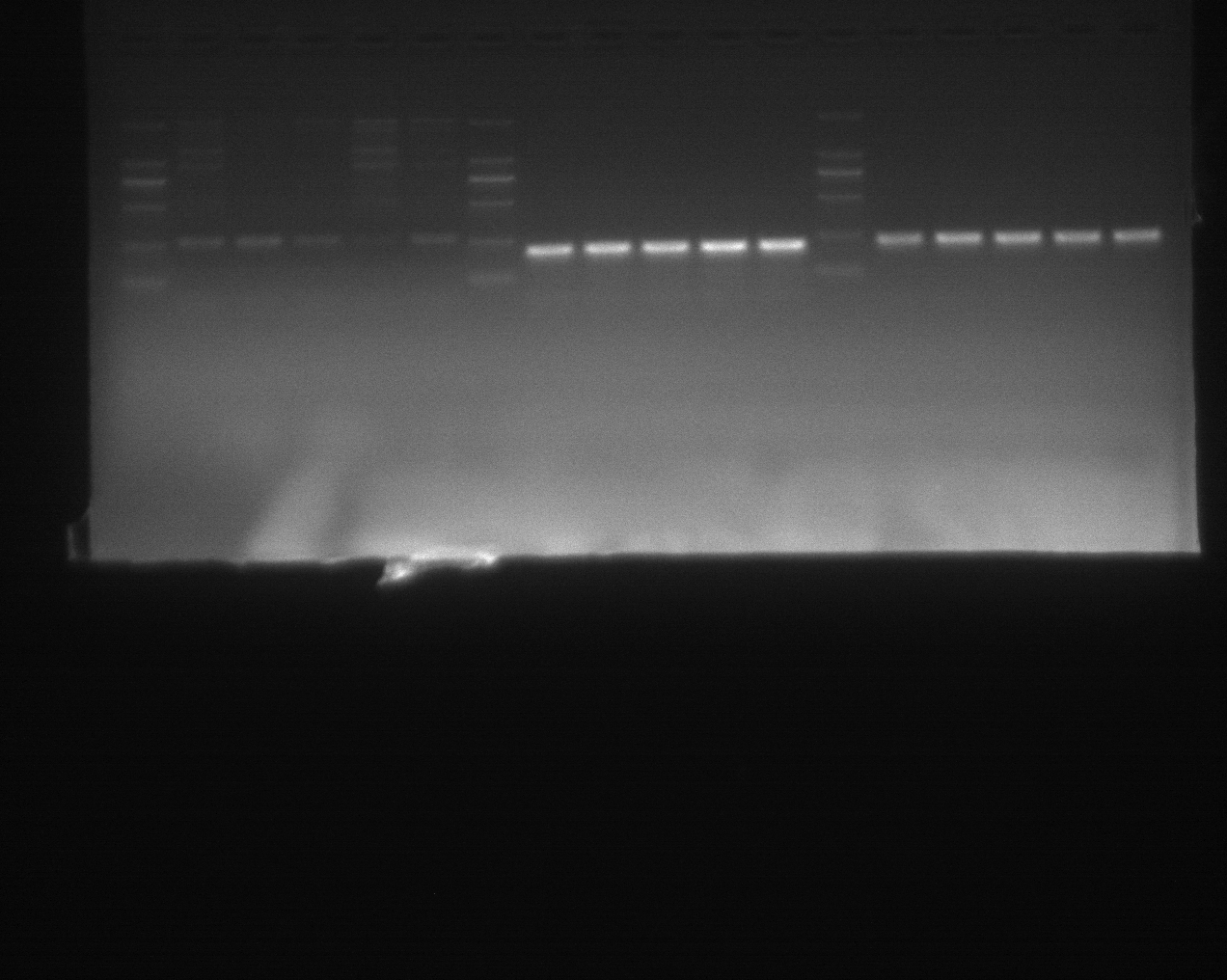

Supplement: S1 File — (ZIP) [file pone.0247271.s007.zip › JA treat_ZmJAZ13 15 20.tif]

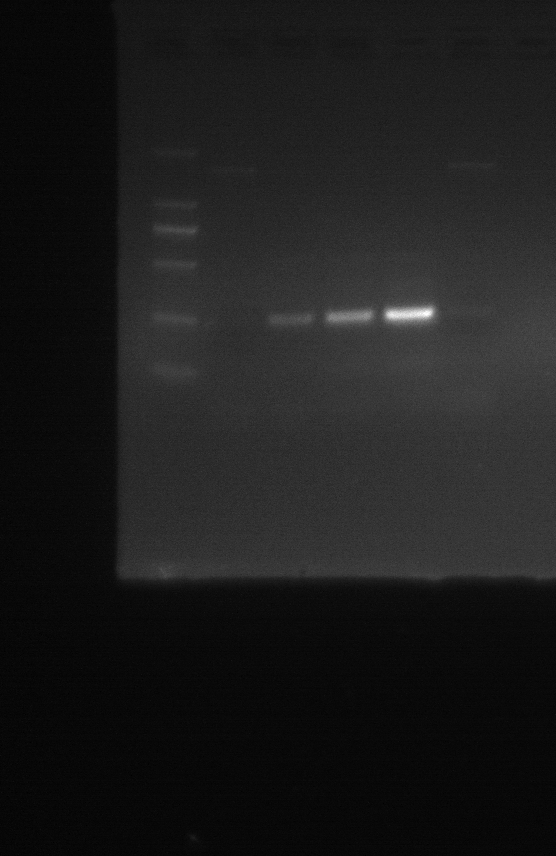

Supplement: S1 File — (ZIP) [file pone.0247271.s007.zip › JA treat_ZmJAZ18.png]

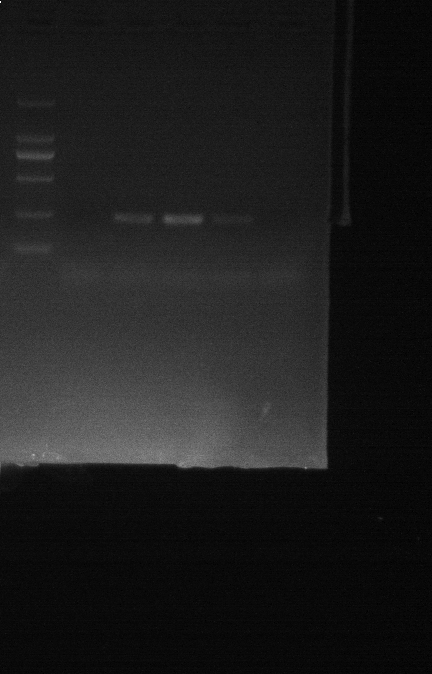

Supplement: S1 File — (ZIP) [file pone.0247271.s007.zip › JA treat_ZmJAZ23.png]

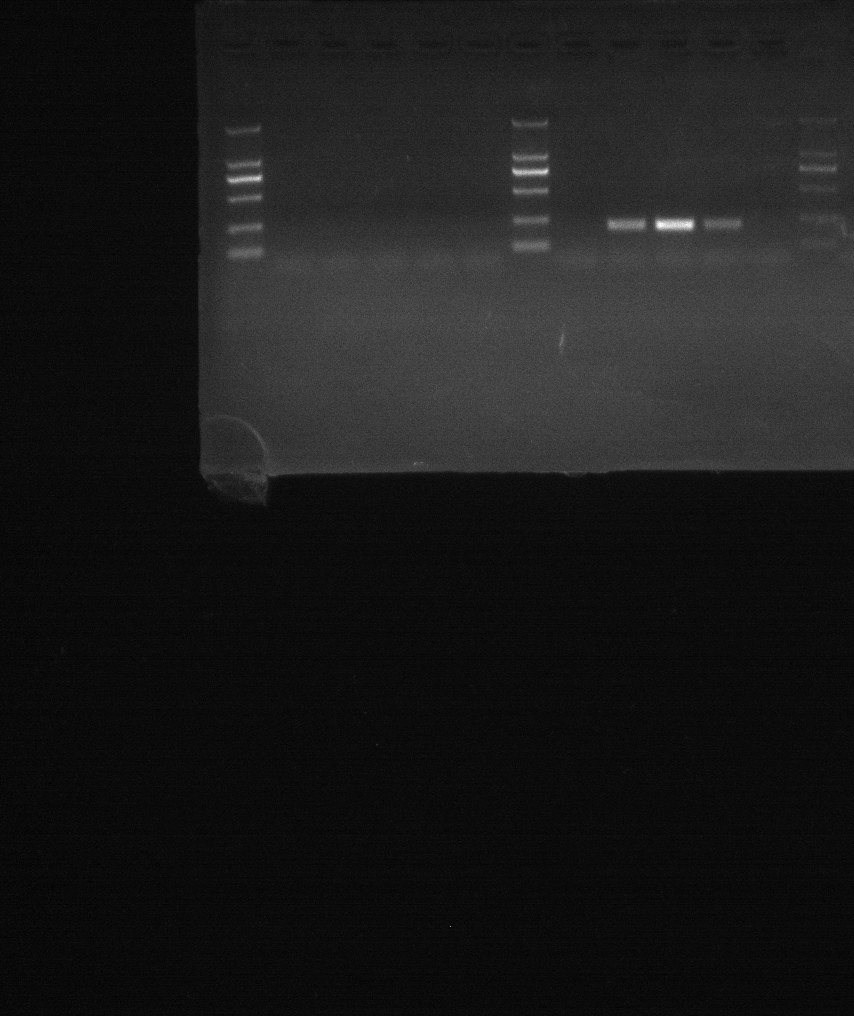

Supplement: S1 File — (ZIP) [file pone.0247271.s007.zip › JA treat_ZmJAZ25_the right half.png]

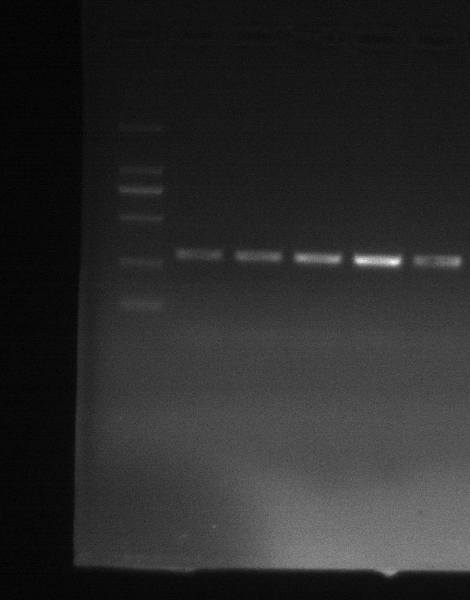

Supplement: S1 File — (ZIP) [file pone.0247271.s007.zip › JA treat_ZmJAZ3.png]

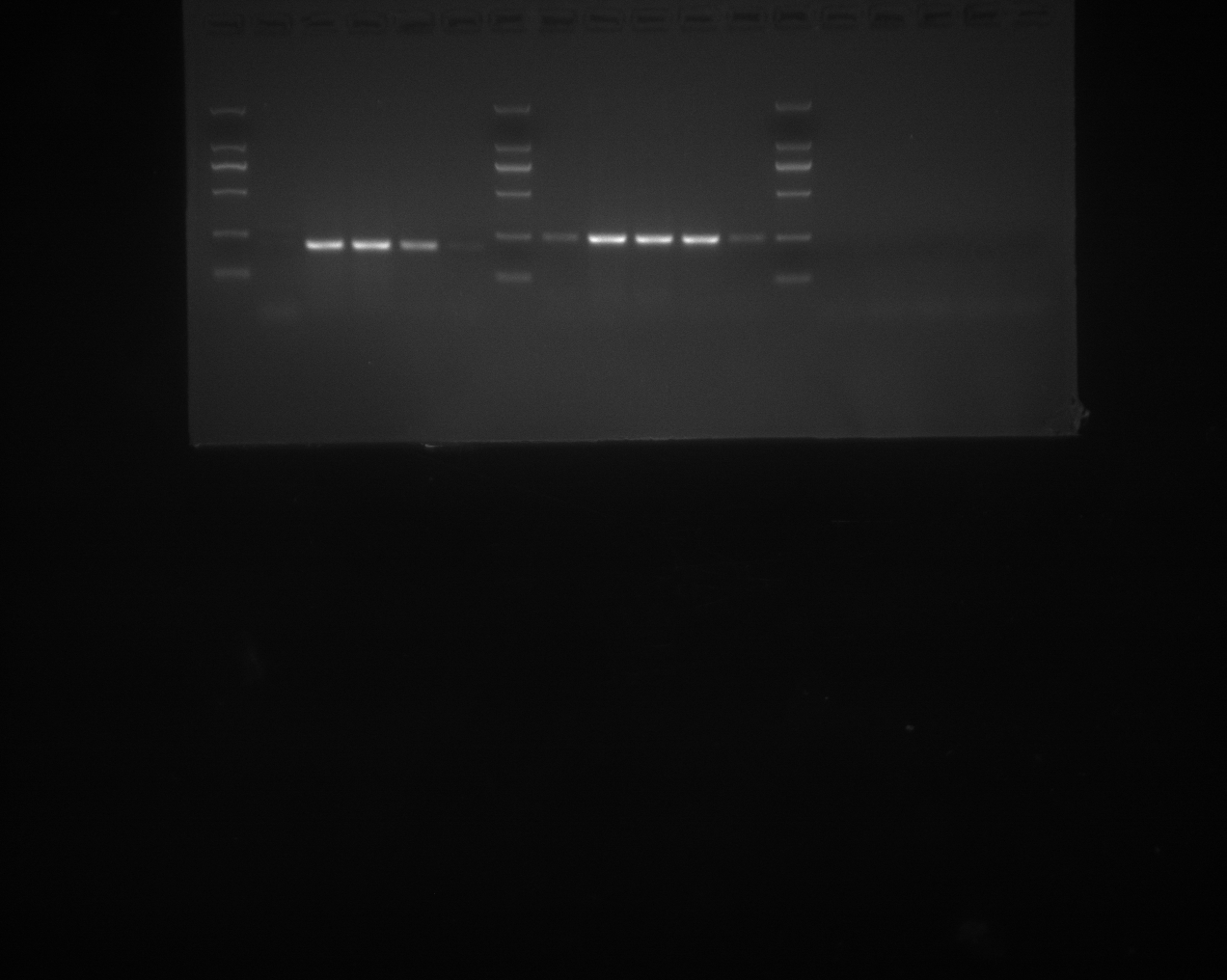

Supplement: S1 File — (ZIP) [file pone.0247271.s007.zip › JA treat_ZmJAZ31 32.tif]

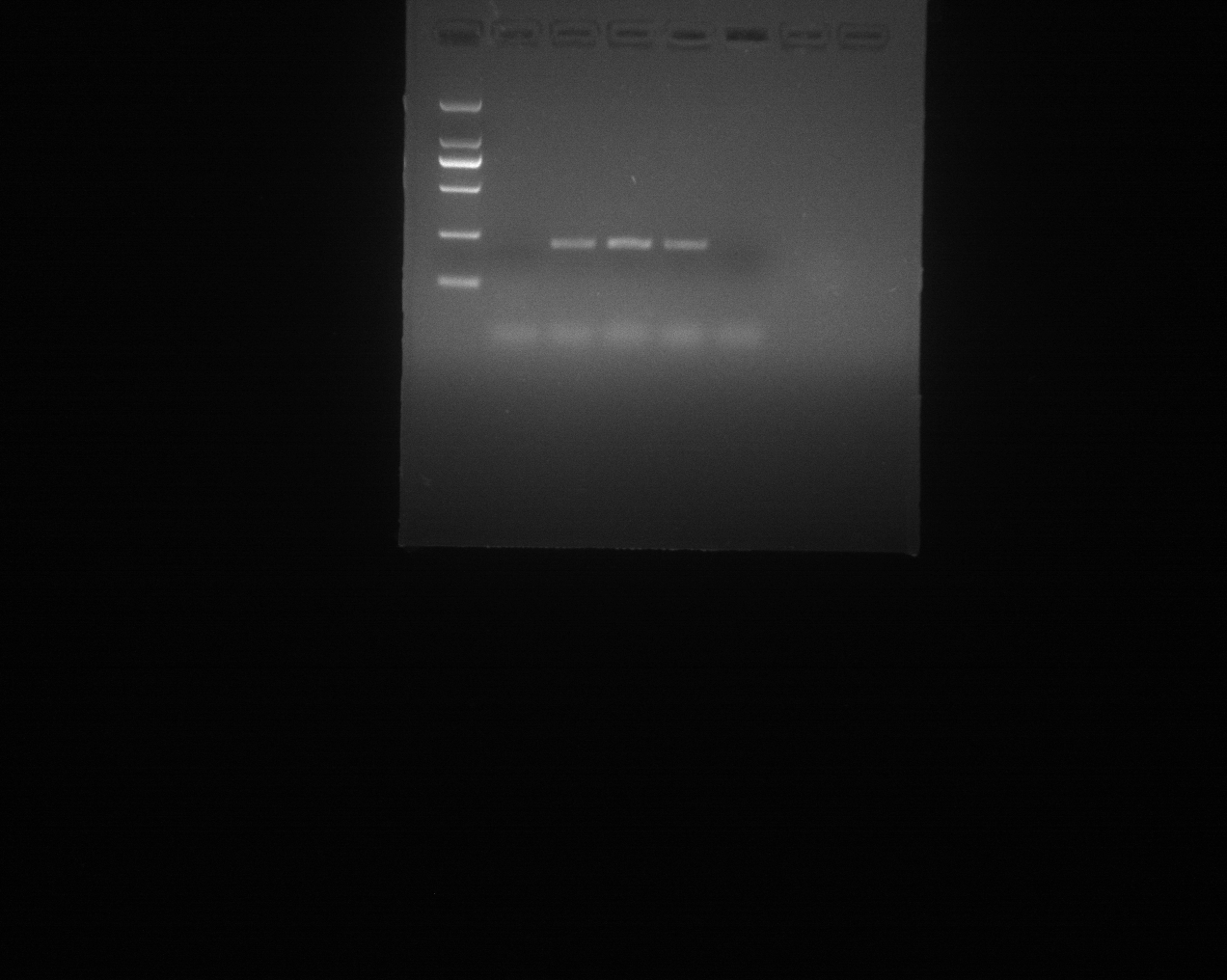

Supplement: S1 File — (ZIP) [file pone.0247271.s007.zip › JA treat_ZmJAZ33.tif]

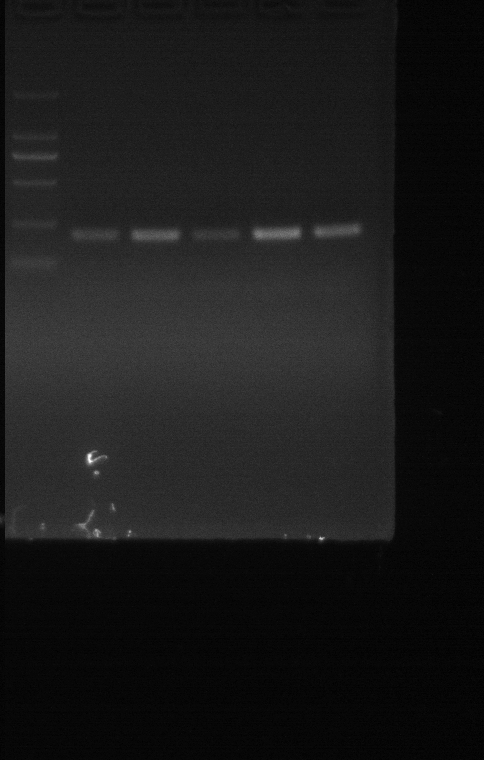

Supplement: S1 File — (ZIP) [file pone.0247271.s007.zip › JA treat_ZmJAZ36.png]

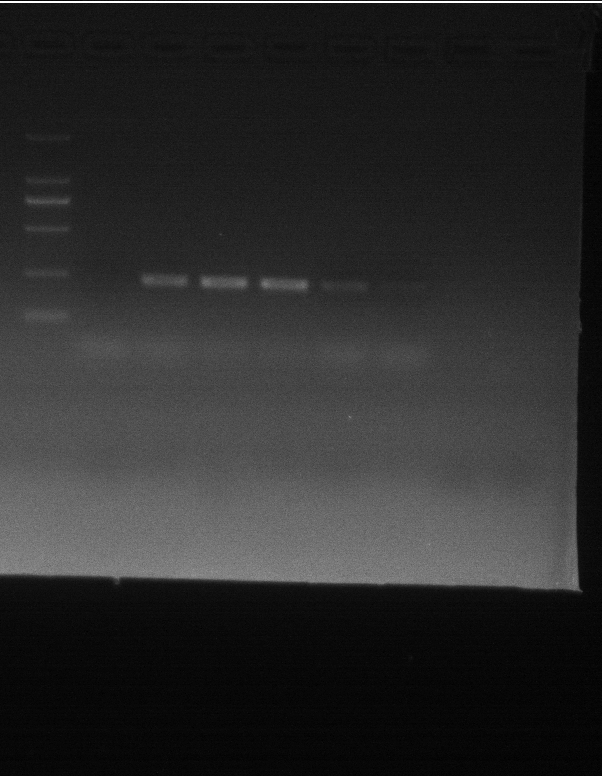

Supplement: S1 File — (ZIP) [file pone.0247271.s007.zip › JA treat_ZmJAZ8.png]

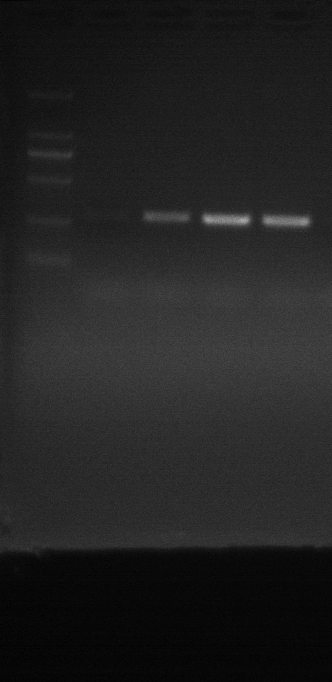

Supplement: S1 File — (ZIP) [file pone.0247271.s007.zip › Wounding_ZmJAZ9.png]

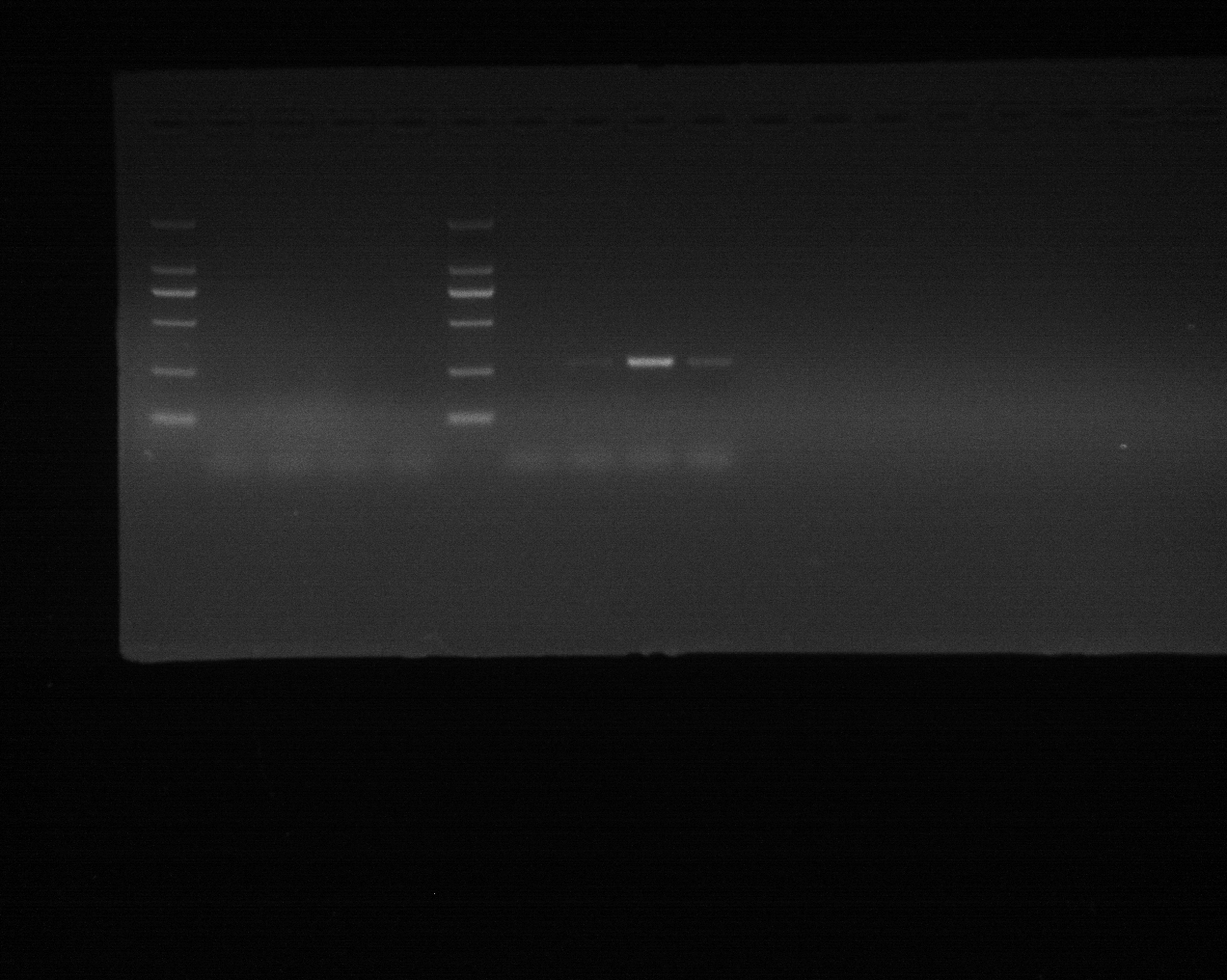

Supplement: S1 File — (ZIP) [file pone.0247271.s007.zip › Wounding_ZmJAZ11_the right half.tif]

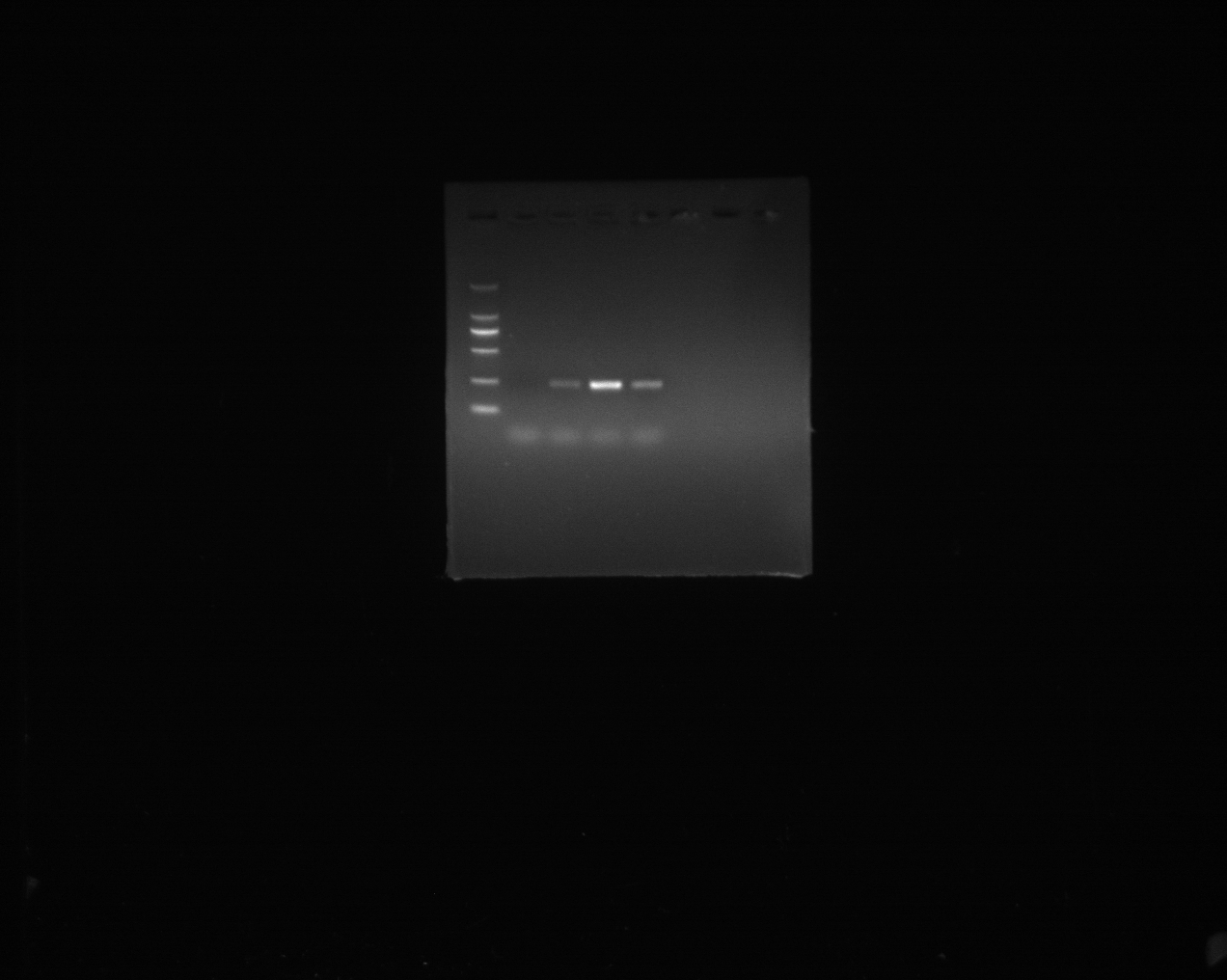

Supplement: S1 File — (ZIP) [file pone.0247271.s007.zip › Wounding_ZmJAZ12.tif]

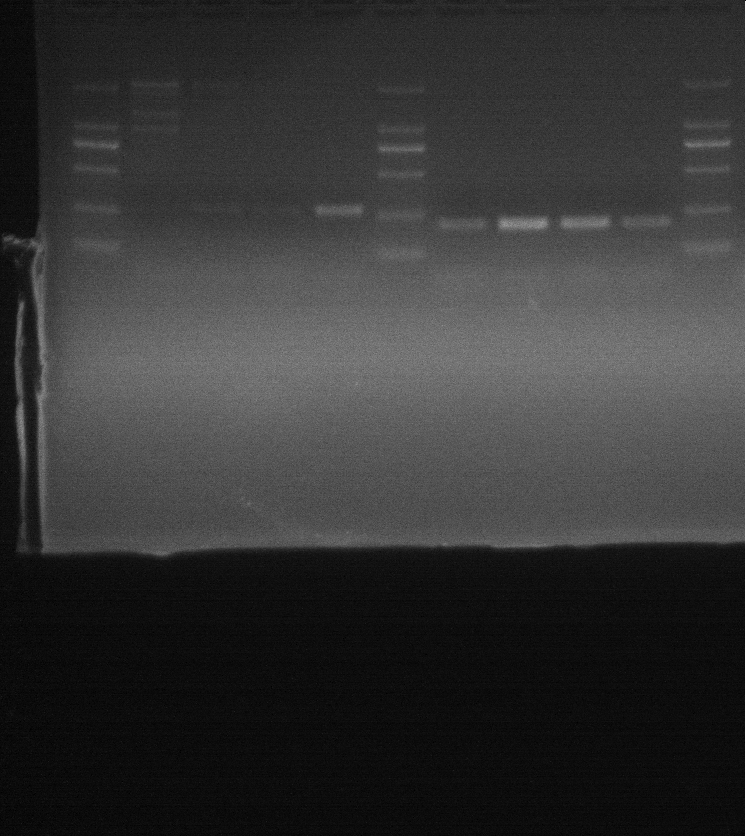

Supplement: S1 File — (ZIP) [file pone.0247271.s007.zip › Wounding_ZmJAZ13 15.png]

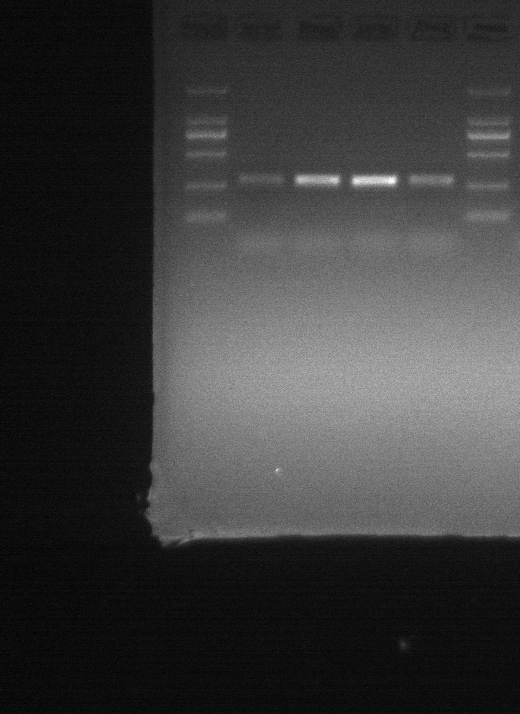

Supplement: S1 File — (ZIP) [file pone.0247271.s007.zip › Wounding_ZmJAZ17.png]

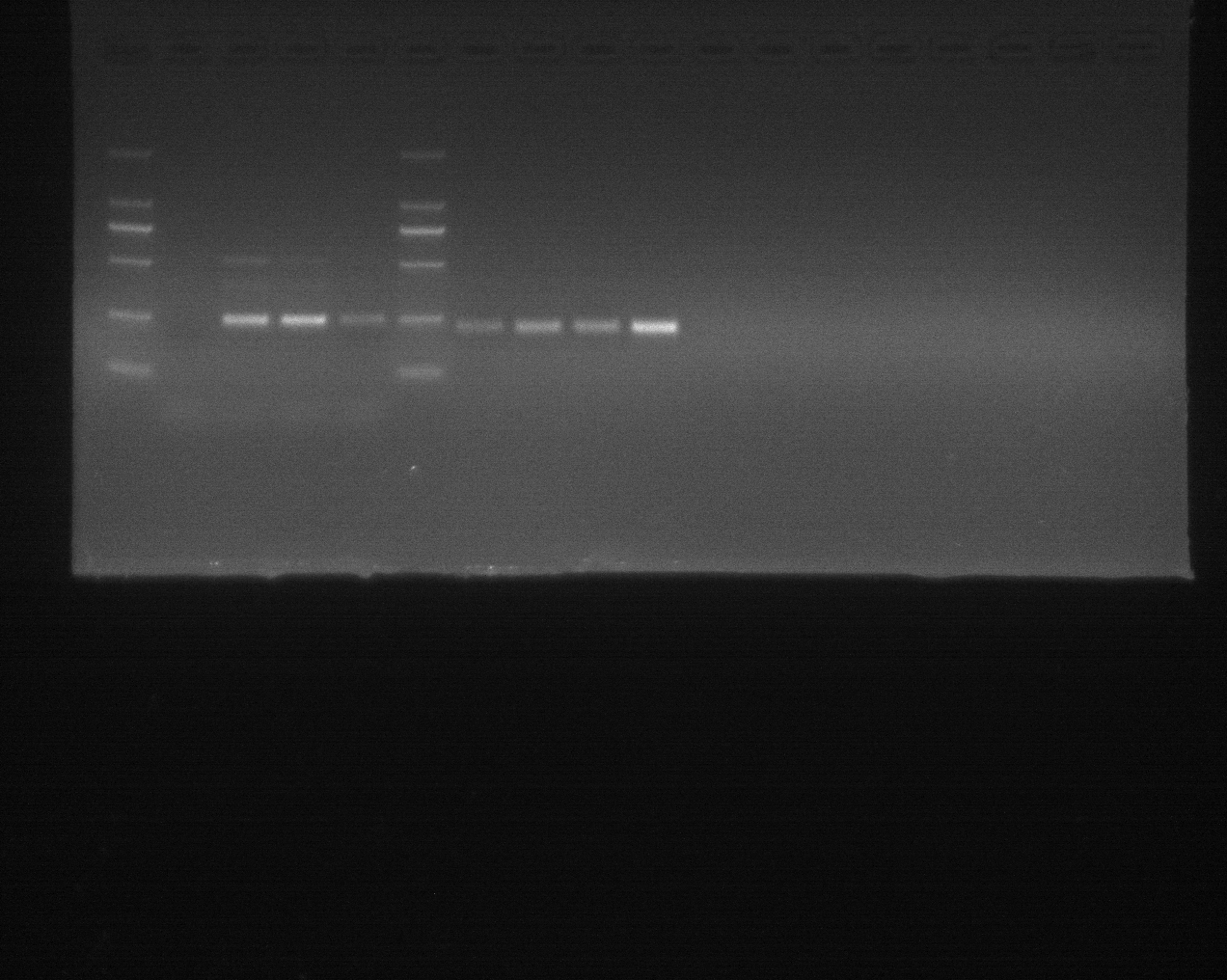

Supplement: S1 File — (ZIP) [file pone.0247271.s007.zip › Wounding_ZmJAZ18 20.tif]

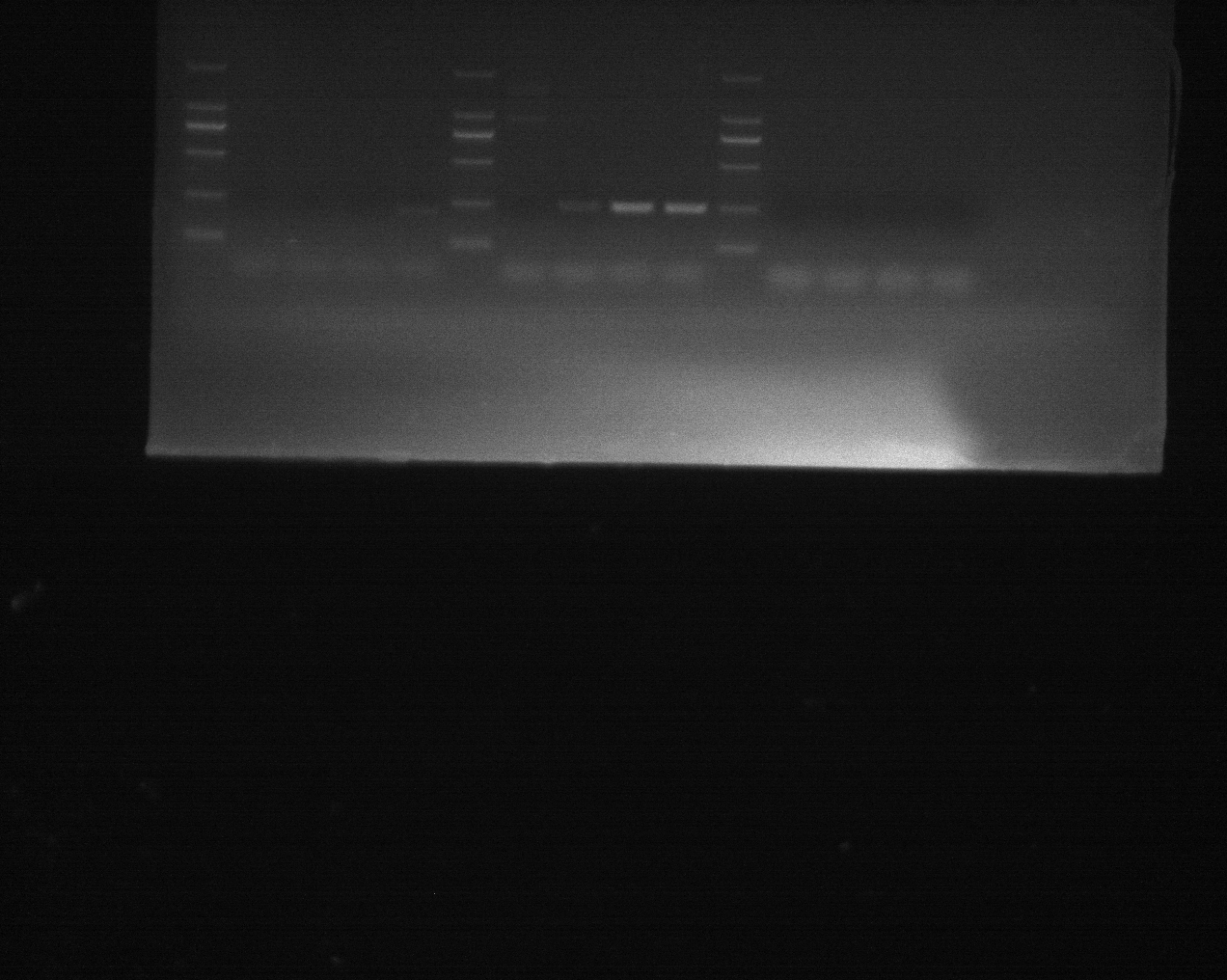

Supplement: S1 File — (ZIP) [file pone.0247271.s007.zip › Wounding_ZmJAZ23_the middle part.tif]

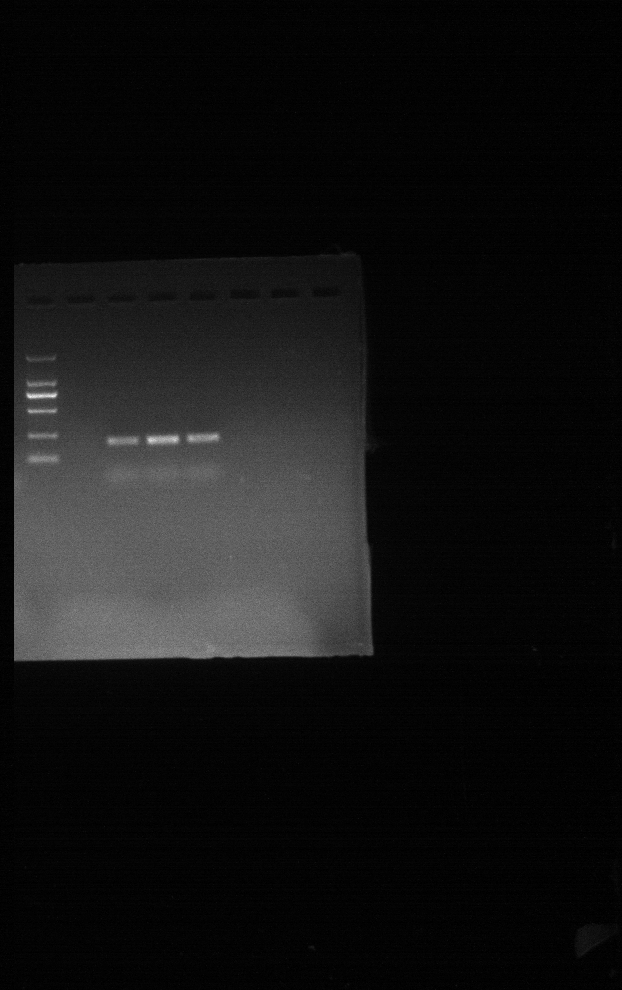

Supplement: S1 File — (ZIP) [file pone.0247271.s007.zip › Wounding_ZmJAZ25.png]

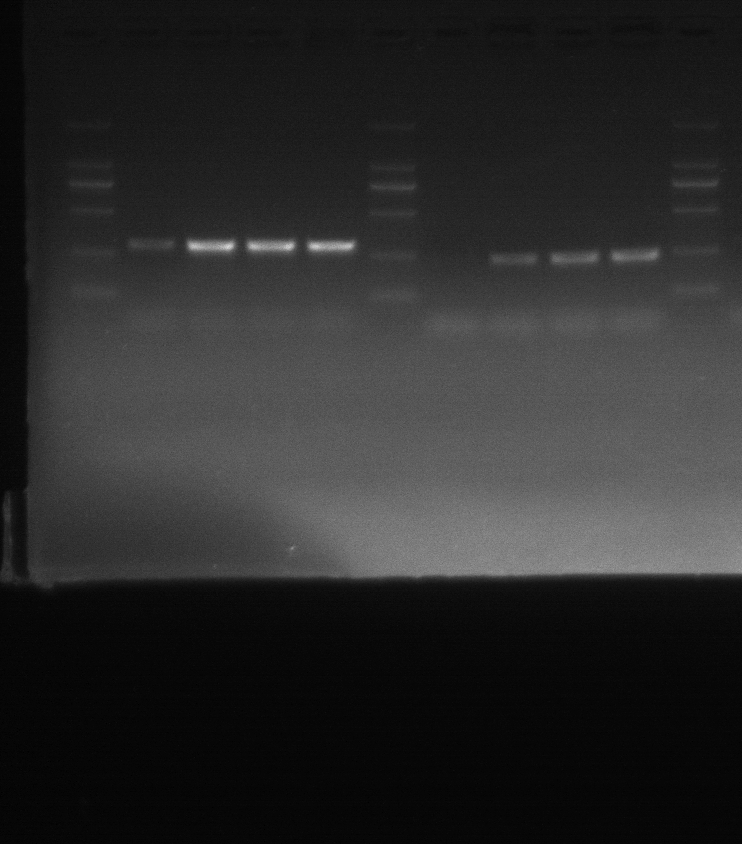

Supplement: S1 File — (ZIP) [file pone.0247271.s007.zip › Wounding_ZmJAZ3 8.png]

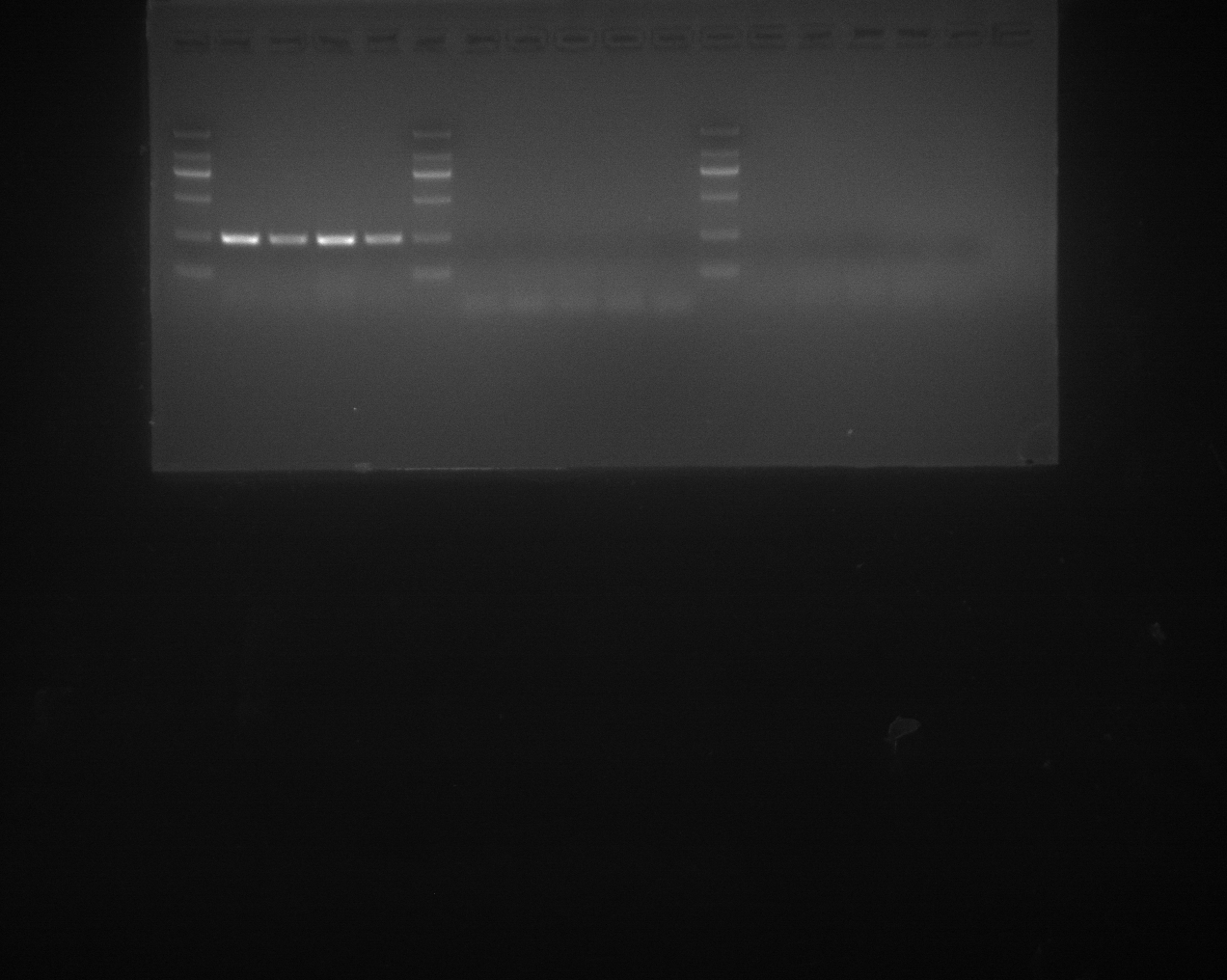

Supplement: S1 File — (ZIP) [file pone.0247271.s007.zip › Wounding_ZmJAZ32_the left part.tif]

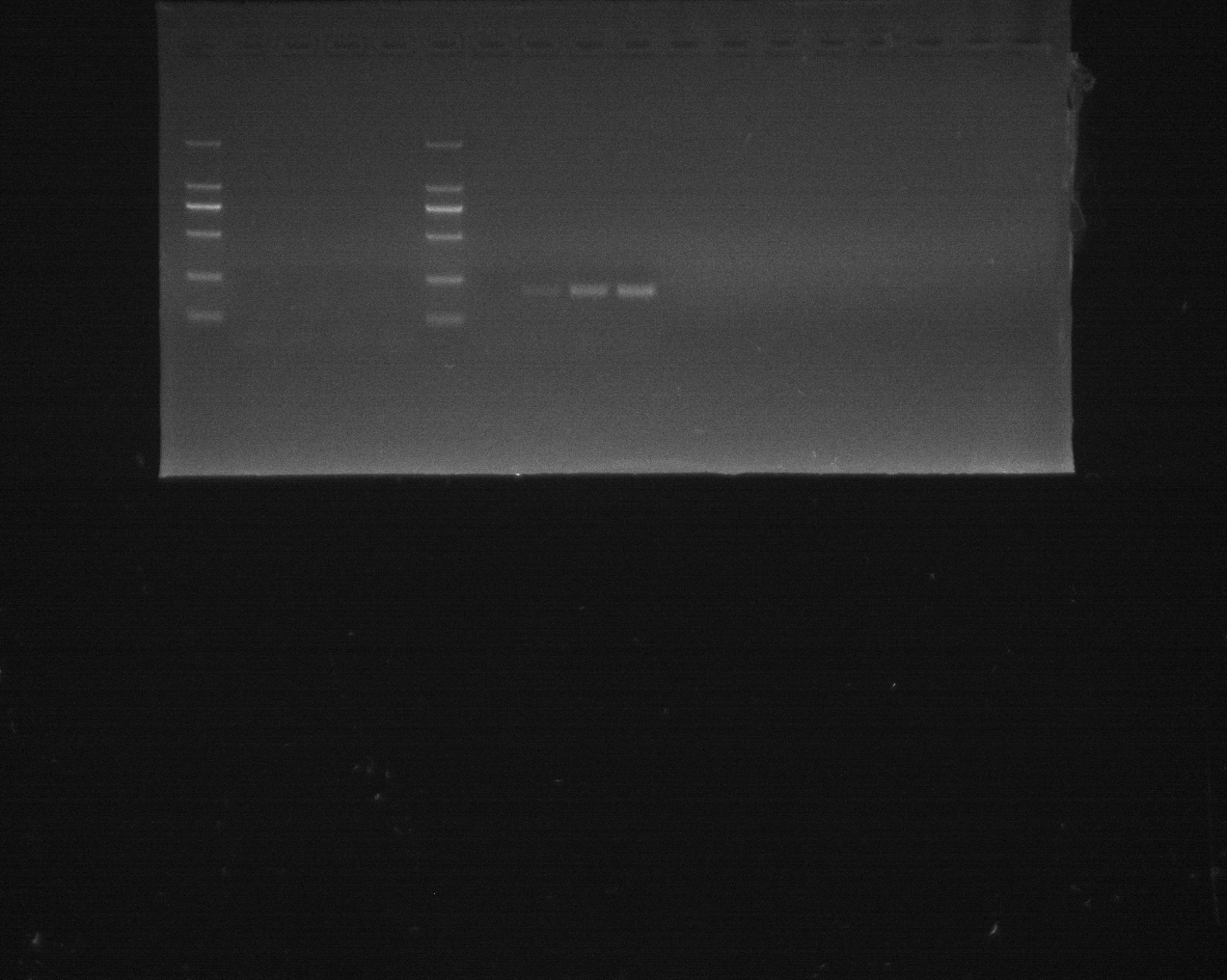

Supplement: S1 File — (ZIP) [file pone.0247271.s007.zip › Wounding_ZmJAZ33_the right part.tif]

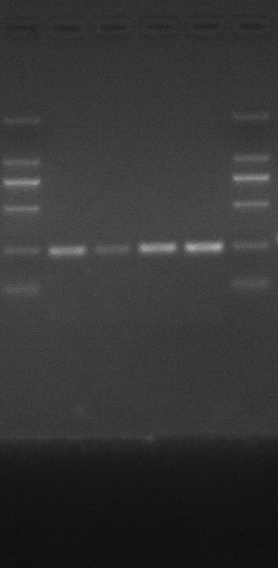

Supplement: S1 File — (ZIP) [file pone.0247271.s007.zip › Wounding_ZmJAZ36.png]

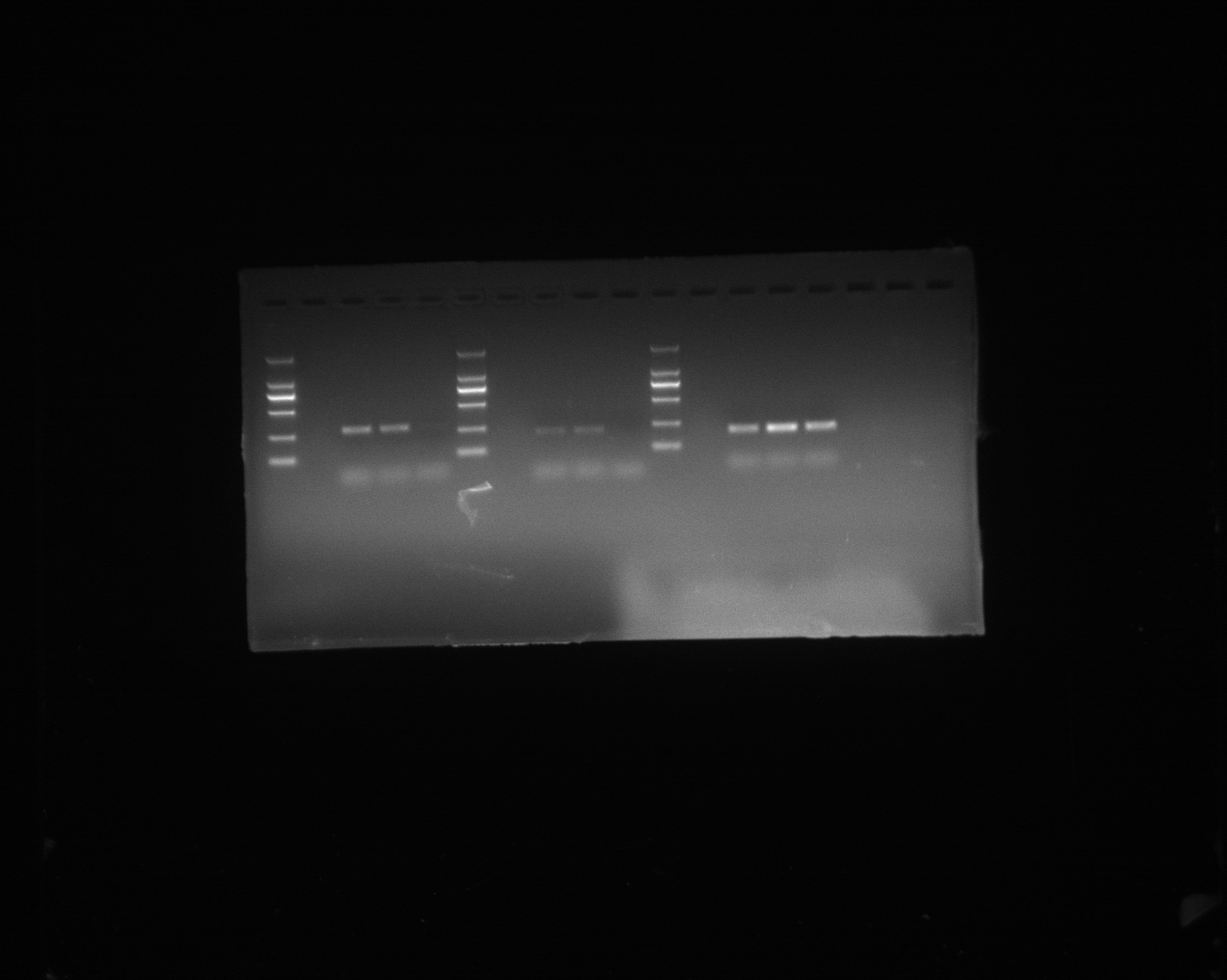

Supplement: S1 File — (ZIP) [file pone.0247271.s007.zip › Wounding_ZmJAZ5 6 31.tif]
