# Supplementary material for: An updated census of the maize TIFY family
Source: PLoS One. 2021 Feb 23;16(2):e0247271. doi: 10.1371/journal.pone.0247271 (PMC7901733; doi:10.1371/journal.pone.0247271)
Supplement: S1 Document — (PDF) [file pone.0247271.s006.pdf]

Supplemental Document for TIFY genes in maize

ZmJAZ1 (TIFY1)

B73 RefGen_v3:
GRMZM2G343157 (zim26 - ZIM-transcription factor 26)
Chr1: 16776170..16777317

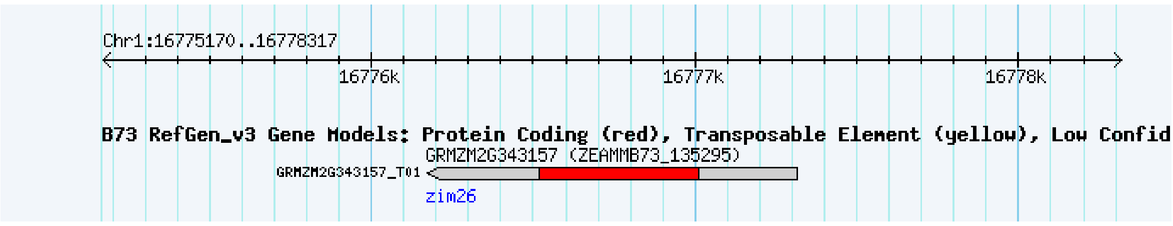

>GRMZM2G343157 Genomic DNA
ACCTTACCTTCGGTGGACACGTTTTCCCTCTCGCTCAGCAGTCAGGACCAGAGGCCCGGGGCCTCTCTCCCTCTGCTCGC
GTCGCGTCTCGCACTCTCGCCTAGTCGCCTAGCAGGCTAGCACTGCTCCTCCACCTCGTGTCGCACTACTCACTGGAGTA
GTAAGTATATAGAACCGTCTCCACATCTCCACAACCTCCAGGGGGTCCATCCATCATTCCATCGTCGAGTCACGTCTCGT
GCGTGCGCAGCCCAGGAACATCGAGCCCCCCCACGCCGACCGGCGGCCTGCCGTGAGCACGTTGCCGATGGCTGCCATGC
CCACGGACAGCATCACCCGCCGTTTCGCCGTCGCGTGCGGCGTGCTCAGCCAGCACGTCAGGAACGGCGGCGCGCCGTTC
CTGACGAAGCATGCGCAGGCCGGAGCCGGAGCCGGAGCCGGACCCGCCGCAGCCCAGGAGATGATCGCCGGCTCGCCGCA
GCAGCTGACCATCCTCTACGGCGGGAGGGTGGTGGTGCTGCTGGACGCCTGCCCGCCCGAGAAGGCGGCCGAGCTGATCC
GCCTCGCCGCCGCGGCCGCGCAGCAGGGGCCGCAGCCGCCGCCGGAGCAGGCGCTTGTGGACATGCCCATCGCCAGGAAG
GCCTCGCTGCGGCGCTTCCTCGCCAAGCGCAAGGACAGGTCCTCCTTCTACGAGGACGACGAGACGCCCGAGCCCAAGAA
GGGCAAGATGGCGGCGGCGAGCGAGGAGCATTCCTCTTCTTGGCTCGCGCTCGGCAGCCTGTGCTCCATGCACGGCCGCT
GAGATCGCCGGACGTGGTTCGTGTCTGTCGACCCGGCCATTGGAGGCGTTGTAGGCTTGTACTGTAGCAGCAATTTACTT
ATGCTTGTGCTACCTACGTAATTACCCCTACCTACTATCATATGCGTGCTCCGTCGGAGAGCGACAGCACGTACTATTCG
TTCGTATCCTTCGTTGGTGGTGGTGGTGGTGGTGTATCATCTATCTATTCTAATCTAACCAGACATGTGAACCTCAGCGA
CAGGAGATGTTCAGCGGTGTGTGTTGTTCATATGCTTTATTTATCATTTATTTTTGGCGTCAGGTTTGTTGTCCTTGTTG
CTTGGGGAATGAAAAGCCATTAAACTCT
>GRMZM2G343157_T01 cDNA
ACCTTACCTTCGGTGGACACGTTTTCCCTCTCGCTCAGCAGTCAGGACCAGAGGCCCGGGGCCTCTCTCCCTCTGCTCGC
GTCGCGTCTCGCACTCTCGCCTAGTCGCCTAGCAGGCTAGCACTGCTCCTCCACCTCGTGTCGCACTACTCACTGGAGTA
GTAAGTATATAGAACCGTCTCCACATCTCCACAACCTCCAGGGGGTCCATCCATCATTCCATCGTCGAGTCACGTCTCGT
GCGTGCGCAGCCCAGGAACATCGAGCCCCCCCACGCCGACCGGCGGCCTGCCGTGAGCACGTTGCCGATGGCTGCCATGC
CCACGGACAGCATCACCCGCCGTTTCGCCGTCGCGTGCGGCGTGCTCAGCCAGCACGTCAGGAACGGCGGCGCGCCGTTC
CTGACGAAGCATGCGCAGGCCGGAGCCGGAGCCGGAGCCGGACCCGCCGCAGCCCAGGAGATGATCGCCGGCTCGCCGCA
GCAGCTGACCATCCTCTACGGCGGGAGGGTGGTGGTGCTGCTGGACGCCTGCCCGCCCGAGAAGGCGGCCGAGCTGATCC
GCCTCGCCGCCGCGGCCGCGCAGCAGGGGCCGCAGCCGCCGCCGGAGCAGGCGCTTGTGGACATGCCCATCGCCAGGAAG
GCCTCGCTGCGGCGCTTCCTCGCCAAGCGCAAGGACAGGTCCTCCTTCTACGAGGACGACGAGACGCCCGAGCCCAAGAA
GGGCAAGATGGCGGCGGCGAGCGAGGAGCATTCCTCTTCTTGGCTCGCGCTCGGCAGCCTGTGCTCCATGCACGGCCGCT
GAGATCGCCGGACGTGGTTCGTGTCTGTCGACCCGGCCATTGGAGGCGTTGTAGGCTTGTACTGTAGCAGCAATTTACTT
ATGCTTGTGCTACCTACGTAATTACCCCTACCTACTATCATATGCGTGCTCCGTCGGAGAGCGACAGCACGTACTATTCG
TTCGTATCCTTCGTTGGTGGTGGTGGTGGTGGTGTATCATCTATCTATTCTAATCTAACCAGACATGTGAACCTCAGCGA
CAGGAGATGTTCAGCGGTGTGTGTTGTTCATATGCTTTATTTATCATTTATTTTTGGCGTCAGGTTTGTTGTCCTTGTTG

CTTGGGGAATGAAAAGCCATTAAACTCT
>GRMZM2G343157_P01 peptide
MAAMPTDSITRRFAVACGVLSQHVRNGGAPFLTKHAQAGAGAGAGPAAAQEMIAGSPQQLTILYGGRVVVLLDACPPEKA
AELIRLAAAAAQQGPQPPPEQALVDMPIARKASLRRFLAKRKDRSSFYEDDETPEPKKGKMAAASEEHSSSWLALGSLCS
MHGR

AGPv4:
Zm00001d027899 (zim26 - ZIM-transcription factor 26)
Chr1: 17141137..17141631

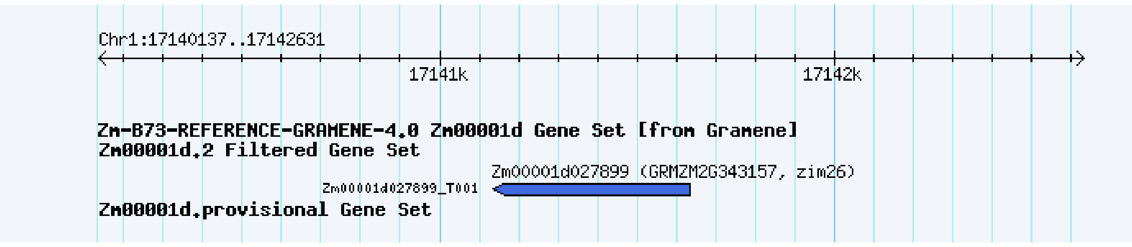

>Zm00001d027899 Genomic DNA
ATGGCTGCCATGCCCACGGACAGCATCACCCGCCGTTTCGCCGTCGCGTGCGGCGTGCTCAGCCAGCACGTCAGGAACGG
CGGCGCGCCGTTCCTGACGAAGCATGCGCAGGCCGGAGCCGGAGCCGGAGCCGGACCCGCCGCAGCCCAGGAGATGATCG
CCGGCTCGCCGCAGCAGCTGACCATCCTCTACGGCGGGAGGGTGGTGGTGCTGCTGGACGCCTGCCCGCCCGAGAAGGCG
GCCGAGCTGATCCGCCTCGCCGCCGCGGCCGCGCAGCAGGGGCCGCAGCCGCCGCCGGAGCAGGCGCTTGTGGACATGCC
CATCGCCAGGAAGGCCTCGCTGCGGCGCTTCCTCGCCAAGCGCAAGGACAGGTCCTCCTTCTACGAGGACGACGAGACGC
CCGAGCCCAAGAAGGGCAAGATGGCGGCGGCGAGCGAGGAGCATTCCTCTTCTTGGCTCGCGCTCGGCAGCCTGTGCTCC
ATGCACGGCCGCTGA
>Zm00001d027899_T001 cDNA
ATGGCTGCCATGCCCACGGACAGCATCACCCGCCGTTTCGCCGTCGCGTGCGGCGTGCTCAGCCAGCACGTCAGGAACGG
CGGCGCGCCGTTCCTGACGAAGCATGCGCAGGCCGGAGCCGGAGCCGGAGCCGGACCCGCCGCAGCCCAGGAGATGATCG
CCGGCTCGCCGCAGCAGCTGACCATCCTCTACGGCGGGAGGGTGGTGGTGCTGCTGGACGCCTGCCCGCCCGAGAAGGCG
GCCGAGCTGATCCGCCTCGCCGCCGCGGCCGCGCAGCAGGGGCCGCAGCCGCCGCCGGAGCAGGCGCTTGTGGACATGCC
CATCGCCAGGAAGGCCTCGCTGCGGCGCTTCCTCGCCAAGCGCAAGGACAGGTCCTCCTTCTACGAGGACGACGAGACGC
CCGAGCCCAAGAAGGGCAAGATGGCGGCGGCGAGCGAGGAGCATTCCTCTTCTTGGCTCGCGCTCGGCAGCCTGTGCTCC
ATGCACGGCCGCTGA
>Zm00001d027899_P001 peptide
MAAMPTDSITRRFAVACGVLSQHVRNGGAPFLTKHAQAGAGAGAGPAAAQEMIAGSPQQLTILYGGRVVVLLDACPPEKA
AELIRLAAAAAQQGPQPPPEQALVDMPIARKASLRRFLAKRKDRSSFYEDDETPEPKKGKMAAASEEHSSSWLALGSLCS
MHGR

ZmJAZ2 (TIFY4)

B73 RefGen_v3:
GRMZM2G445634 (zim16 - ZIM-transcription factor 16)
Chr1: 16791540..16792462

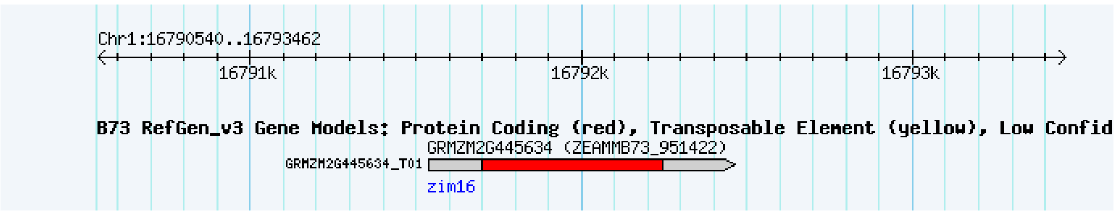

>GRMZM2G445634 Genomic DNA
CCCCGCCCCCATCCCGCTATAAAAGACAAGGCTCCCCGCGACGCCCCCATCCATTCCCACAGCACCAAGCAAGAGCAAAG
AGCAACACAAGCCAAGGCAGCAAACGACCCGCGGCGACGCAAGAAGAGAAGCCAGCAGTCGTCTGGATCCATCGATTGAC
ATGGCCATGGCGGCGGCAGCGGAGGGCAAGAGCCGGAGGTTCGCGCTGGCCTGCGGGGTGCTGAGCCAGTACGTGAAGGC
CGAGCAGCAGATGGCGGCCGCCGGGGCCCCTGCCCCGCGCGCCCCGGCCACGACGCTGAGCCTCATGCCCGGCGCGGACG
TCGGCGCCGAGCAGGAGCAGGCCGCCGCGAGGGGGCAGGAGACGGCCGGGTCCGCCTCCACGGCGGCGCCGCTCACCATC
TTCTACGGCGGCAGGGTGGTCGTGTTCGACGACTTCCCGGCGGAGAAGGCGGCCGAGGTCATGCGCCTCGCAGCTGGCGC
CGAGCGCCCGGCGGCCCCGGCCCCGGCGCCGGCGCCGCGCGACGACCTGCCCATCGCCCGGAAGGCGTCGCTGCAGCGGT
TCCTGGCCAAGCGCAAGGACCGCCTCGTGGAGCGCGCCCCCTACGCCCGCCCGTCCCCATCCGAGGAGGCGGAGAAGGCG
AAGCCGGCCTCCTCGTGGCTCGGGCTCGGCGGCACGGACGCCGAGCGCCTCAACATCGCGCTGTGATTTGGATCTCGCTT
GCTACCCACCTGGATCGACCGGGGGCGATTTGATTGGCTGGGTTCCTGGCTTCCTTTGGGTCTGCTGGATTGGGGTTGGT
GTGGAGATCGGAGACGGAGGATTGTAGAAACGTATTCTTGTTTTTTTTCTCTCTCCCTTACGAGAGAGTTTGGCTGTTAA
TTGATTCGATAATAGAAAGATGGAGGATGTACAATTCGTGTCT
>GRMZM2G445634_T01 cDNA
CCCCGCCCCCATCCCGCTATAAAAGACAAGAGCAACACAAGCCAAGGCAGCAAACGACCCGCGGCGACGCAAGAAGAGAA
GCCAGCAGTCGTCTGGATCCATCGATTGACATGGCCATGGCGGCGGCAGCGGAGGGCAAGAGCCGGAGGTTCGCGCTGGC
CTGCGGGGTGCTGAGCCAGTACGTGAAGGCCGAGCAGCAGATGGCGGCCGCCGGGGCCCCTGCCCCGCGCGCCCCGGCCA
CGACGCTGAGCCTCATGCCCGGCGCGGACGTCGGCGCCGAGCAGGAGCAGGCCGCCGCGAGGGGGCAGGAGACGGCCGGG
TCCGCCTCCACGGCGGCGCCGCTCACCATCTTCTACGGCGGCAGGGTGGTCGTGTTCGACGACTTCCCGGCGGAGAAGGC
GGCCGAGGTCATGCGCCTCGCAGCTGGCGCCGAGCGCCCGGCGGCCCCGGCCCCGGCGCCGGCGCCGCGCGACGACCTGC
CCATCGCCCGGAAGGCGTCGCTGCAGCGGTTCCTGGCCAAGCGCAAGGACCGCCTCGTGGAGCGCGCCCCCTACGCCCGC
CCGTCCCCATCCGAGGAGGCGGAGAAGGCGAAGCCGGCCTCCTCGTGGCTCGGGCTCGGCGGCACGGACGCCGAGCGCCT
CAACATCGCGCTGTGATTTGGATCTCGCTTGCTACCCACCTGGATCGACCGGGGGCGATTTGATTGGCTGGGTTCCTGGC
TTCCTTTGGGTCTGCTGGATTGGGGTTGGTGTGGAGATCGGAGACGGAGGATTGTAGAAACGTATTCTTGTTTTTTTTCT
CTCTCCCTTACGAGAGAGTTTGGCTGTTAATTGATTCGATAATAGAAAGATGGAGGATGTACAATTCGTGTCT
>GRMZM2G445634_P01 peptide
MAMAAAAEGKSRRFALACGVLSQYVKAEQQMAAAGAPAPRAPATTLSLMPGADVGAEQEQAAARGQETAGSASTAAPLTI
FYGGRVVVFDDFPAEKAAEVMRLAAGAERPAAPAPAPAPRDDLPIARKASLQRFLAKRKDRLVERAPYARPSPSEEAEKA
KPASSWLGLGGTDAERLNIAL

AGPv4:
Zm00001d027901 (zim16 - ZIM-transcription factor 16)
Chr1: 17156322..17156867

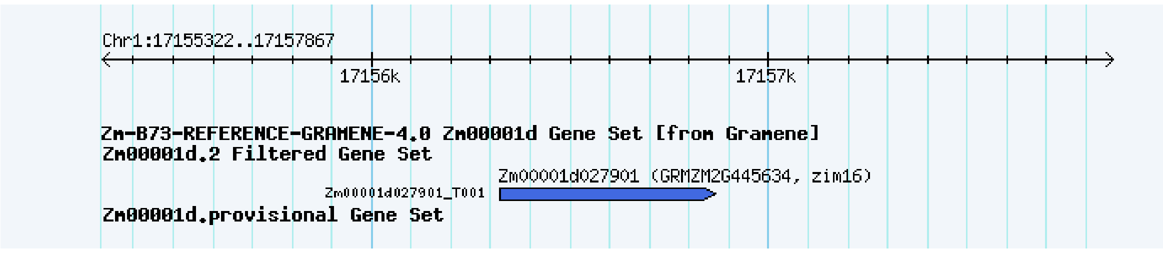

>Zm00001d027901 Genomic DNA
ATGGCCATGGCGGCGGCAGCGGAGGGCAAGAGCCGGAGGTTCGCGCTGGCCTGCGGGGTGCTGAGCCAGTACGTGAAGGC
CGAGCAGCAGATGGCGGCCGCCGGGGCCCCTGCCCCGCGCGCCCCGGCCACGACGCTGAGCCTCATGCCCGGCGCGGACG
TCGGCGCCGAGCAGGAGCAGGCCGCCGCGAGGGGGCAGGAGACGGCCGGGTCCGCCTCCACGGCGGCGCCGCTCACCATC
TTCTACGGCGGCAGGGTGGTCGTGTTCGACGACTTCCCGGCGGAGAAGGCGGCCGAGGTCATGCGCCTCGCAGCTGGCGC
CGAGCGCCCGGCGGCCCCGGCCCCGGCGCCGGCGCCGCGCGACGACCTGCCCATCGCCCGGAAGGCGTCGCTGCAGCGGT
TCCTGGCCAAGCGCAAGGACCGCCTCGTGGAGCGCGCCCCCTACGCCCGCCCGTCCCCATCCGAGGAGGCGGAGAAGGCG
AAGCCGGCCTCCTCGTGGCTCGGGCTCGGCGGCACGGACGCCGAGCGCCTCAACATCGCGCTGTGA
>Zm00001d027901_T001 cDNA
ATGGCCATGGCGGCGGCAGCGGAGGGCAAGAGCCGGAGGTTCGCGCTGGCCTGCGGGGTGCTGAGCCAGTACGTGAAGGC
CGAGCAGCAGATGGCGGCCGCCGGGGCCCCTGCCCCGCGCGCCCCGGCCACGACGCTGAGCCTCATGCCCGGCGCGGACG
TCGGCGCCGAGCAGGAGCAGGCCGCCGCGAGGGGGCAGGAGACGGCCGGGTCCGCCTCCACGGCGGCGCCGCTCACCATC
TTCTACGGCGGCAGGGTGGTCGTGTTCGACGACTTCCCGGCGGAGAAGGCGGCCGAGGTCATGCGCCTCGCAGCTGGCGC
CGAGCGCCCGGCGGCCCCGGCCCCGGCGCCGGCGCCGCGCGACGACCTGCCCATCGCCCGGAAGGCGTCGCTGCAGCGGT
TCCTGGCCAAGCGCAAGGACCGCCTCGTGGAGCGCGCCCCCTACGCCCGCCCGTCCCCATCCGAGGAGGCGGAGAAGGCG
AAGCCGGCCTCCTCGTGGCTCGGGCTCGGCGGCACGGACGCCGAGCGCCTCAACATCGCGCTGTGA
>Zm00001d027901_P001 peptide
MAMAAAAEGKSRRFALACGVLSQYVKAEQQMAAAGAPAPRAPATTLSLMPGADVGAEQEQAAARGQETAGSASTAAPLTI
FYGGRVVVFDDFPAEKAAEVMRLAAGAERPAAPAPAPAPRDDLPIARKASLQRFLAKRKDRLVERAPYARPSPSEEAEKA
KPASSWLGLGGTDAERLNIAL

ZmJAZ3 (TIFY6)

B73 RefGen_v3:
GRMZM2G117513 (zim24 - ZIM-transcription factor 24)
Chr1: 69894564..69897090

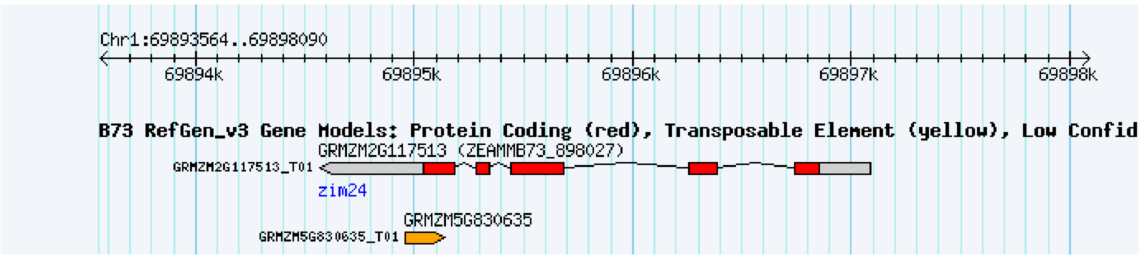

>GRMZM2G117513 Genomic DNA
GAGAAAGAATCCAACTCGCAATTTATGAATGGGAATAAAGATGAGCCCAGTGCCTCTTCATTTTTATGCACGCCCAGCAC
GCCCAGACGGCGGAGGAAGGGGGAAAGAGGAGGGCAGAGCAGCAACAGTACATCGAGCGAGGGAGTAGAGTAAATTGAAT
CAGGTTTCGTGTCGATTCGGAGGCTTTCGCTTCTGCTTGCGGAGTTGCGGTGGAGATTTGAGGCCTGCGGCGAAGATGGC
ACCTGCGGAATCCGGGGAGAAGGCGACCAGCTTCGCCATGGCCTGCAGCCTGCTCAGCCGCTACGTCAGGCAGAACGGTG
CCGCCGCTGCCGATCTCGGCCTCGCTATCAGAGGTGAAGCCTCTTCTCTTGTTGAGGAAAAATTTGGGGTCTCGAGGAGG
TCGGTAGGTGTTCTTTATCTGTTGGGAGTTTAGCGGTGTTTGTTGGGGAGAAGCGGTGAATTGAGGTGCGATTCATGCTA
AAAGTTGCTATTTTGAGGTGCTCCCGTAGTCGTCTCGTGTCAGATCTGGTCCCGAAGTCTTGACCTGGAAGCAAATATGT
CACTTGTATTTGGTTTCGATCAAGAATTTGGGCTTCTGGCTGATCTAACATCCGAATCTGATAGCTTCCTGTCCATCTCT
GTACAGTCTATCTGAAAATTATCGGCTGAACTTTTACTGAAGCAGAGTGCTCTATCCTGTTTCAGCTGAATCCGATGCAA
AGAGGACGTCGACGGACATGGAAAAGGGGGAGACGGGGAAGGAGACCATGGATCTCTTCCCCCAGATCGCTGGATTCGGC
TCGGAGGCCGCCACGAAGGAAGCCCCTGATGCGAGGTACTTGACGAAATAGATACCTCTCTGTTCTTTTGGGTTTCAGTT

CACAGCCATTCTCATGTCATGTTAGCATGGTACTATTGCTGTAGTCAGTTGATGACTGAAAACGTAAAAAAAAAAAAAAC
AATCTAGCTGCTGTGAGAAGCAGCTCTTGGTGGCCAGTTTTTATTTGTCAAACAGTTGTGCCTCAGATCGATGTAGATG
GTTCGATCTCCACACACAGCAGTACAACCCAGATCTTTGTCTTCAGAAAATAGTGAGAAGTTAATTTATCATCTGAAACT
GAATATTTGAAGAGTATTGTTCGTAGGGCTTCTCACACTGCAAACCAGACTGCCAAACAATCTCCGGCAAAGCTTTCTGT
GATGTGTGCCAAAGCTTTCTTGCTATGTCGTAGTGGTTGAATGGTAGAAGGTCCATCCATGAATCTGTAGAACTTGGCGC
TCCGAAACTAAATGAATGAACAATTGGTGCCACGCAAGTTCTGAAACTTCAAAATTTTAACTCGTTGAAGTCGTTTCATG
CGTATAAGTTAGCCACCAAATTTGACAACCGGAAACATCCTGATTCTCAGGGAGCCAGAGAAGCGCCAGCTGACTATCTT
CTATGGCGGGAAGGTCCTTGTGTTCGATGACTTCCCTGCTGAGAAGGCAAAAGACCTGATGCAAATGGCCAGCAAGGGTT
CACCCGTAGCCCAGAACCCTGGCCTGCTGCCTCCCTCCACAGCTGCAACCGTTACTGACAGCACCAAGATCGCAGCAGTG
CCAGCCGCACCAATTGCTGTGGCCAACGCCCAGAAGAGTGCAGCAGGTAGCTGACTCGTGCACCCCACCTTGTTCATATG
CACGCGGCATTTCTCTGAATGATTGATTTCTGTCTTGCTCACTGTTCCCAAATTGTATAATGTTTGCAGATATACCGCAG
GCTCCCAAGGCATCTCTTCGCAGGTTCCTTGAGAAGAGAAAGGATCGGTAGGTTACTCTTGCTGCTGTTGGGATGACAAG
GCTGTTGTTTTAACGATACAGCTCTTCGAGGAGGATCTATCTAATCTGTAATATTCCCTGTTTCAGCCTTACTGCGAAAG
CACCATACCCGGGTTCCCCTTCAGATGTTACACCTGTGAAGAAGGAGATGTCGGATGGTCAGCCATGGCTTGGGCTGGGA
CCTCAGATCGCCAACCCTGATCTGAGCTTGTCCAAGGAGGCGAGCCAGTGATCGATGATCCAGCATCACCATCTTCAAAA
CTGCGATACTCAACAGCCATGTGTGGAAGTGTTCTGTTTTGGGCCCCTATCATTCCGGGTTTTTTTTCGCATGGATAAAT
AGGTTGTACGAAAGTAAAGAACACAGCTAATGACGGACTTCAGTAAGGATTACCGGCGCTGGGGCAGGTTTGGCTGATCA
GCCTTTGCTGGCCTGACCCAAGTGGCCATTTTAGAAAGATGAAAATGTAATGTAGTAGTTGCTAGAACAAGTTGGCATCT
TGGGATGTTCTGTGTTGTTTGGCTTTGGCACTGGTGCGTGTCAGTGTCACAAAGCTATTTATTTTCACAGATACATGCTA
TATGATTTTTTTTATTATCTTCTGGTTGTCTCTGTAGGGTTATGAAATATCACAGCTTTTCTTTCTAAGTGATTGTGATC
AATGCCCCTGGACTATCAGAATGTTGTTCTCTGAAGTTACAATCTAC

>GRMZM2G117513_T01 cDNA

GAGAAAGAATCCAACTCGCAATTTATGAATGGGAATAAAGATGAGCCCAGTGCCTCTTCATTTTTATGCACGCCCAGCAC
GCCCAGACGGCGGAGGAAGGGGGAAAGAGGAGGGCAGAGCAGCAACAGTACATCGAGCGAGGGAGTAGAGTAAATTGAAT
CAGGTTTCGTGTCGATTCGGAGGCTTTCGCTTCTGCTTGCGGAGTTGCGGTGGAGATTTGAGGCCTGCGGCGAAGATGGC
ACCTGCGGAATCCGGGGAGAAGGCGACCAGCTTCGCCATGGCCTGCAGCCTGCTCAGCCGCTACGTCAGGCAGAACGGTG
CCGCCGCTGCCGATCTCGGCCTCGCTATCAGAGCTGAATCCGATGCAAAGAGGACGTCGACGGACATGGAAAAGGGGGAG
ACGGGGAAGGAGACCATGGATCTCTTCCCCCAGATCGCTGGATTCGGCTCGGAGGCCGCCACGAAGGAAGCCCCTGATGC
GAGGGAGCCAGAGAAGCGCCAGCTGACTATCTTCTATGGCGGGAAGGTCCTTGTGTTCGATGACTTCCCTGCTGAGAAGG
CAAAAGACCTGATGCAAATGGCCAGCAAGGGTTCACCCGTAGCCCAGAACCCTGGCCTGCTGCCTCCCTCCACAGCTGCA
ACCGTTACTGACAGCACCAAGATCGCAGCAGTGCCAGCCGCACCAATTGCTGTGGCCAACGCCCAGAAGAGTGCAGCAGA
TATACCGCAGGCTCCCAAGGCATCTCTTCGCAGGTTCCTTGAGAAGAGAAAGGATCGCCTTACTGCGAAAGCACCATACC
CGGGTTCCCCTTCAGATGTTACACCTGTGAAGAAGGAGATGTCGGATGGTCAGCCATGGCTTGGGCTGGGACCTCAGATC
GCCAACCCTGATCTGAGCTTGTCCAAGGAGGCGAGCCAGTGATCGATGATCCAGCATCACCATCTTCAAAACTGCGATAC
TCAACAGCCATGTGTGGAAGTGTTCTGTTTTGGGCCCCTATCATTCCGGGTTTTTTTTCGCATGGATAAATAGGTTGTAC
GAAAGTAAAGAACACAGCTAATGACGGACTTCAGTAAGGATTACCGGCGCTGGGGCAGGTTTGGCTGATCAGCCTTTGCT
GGCCTGACCCAAGTGGCCATTTTAGAAAGATGAAAATGTAATGTAGTAGTTGCTAGAACAAGTTGGCATCTTGGGATGTT
CTGTGTTGTTTGGCTTTGGCACTGGTGCGTGTCAGTGTCACAAAGCTATTTATTTTCACAGATACATGCTATATGATTTT
TTTTATTATCTTCTGGTTGTCTCTGTAGGGTTATGAAATATCACAGCTTTTCTTTCTAAGTGATTGTGATCAATGCCCCT
GGACTATCAGAATGTTGTTCTCTGAAGTTACAATCTAC

>GRMZM2G117513_P01 peptide

MAPAESGEKATSFAMACSLLSRYVRQNGAAAADLGLAIRAESDAKRTSTDMEKGETGKETMDLFPQIAGFGSEAATKEAP
DAREPEKRQLTIFYGGKVLVFDDFPAEKAKDLMQMASKGSPVAQNPGLLPPSTAATVTDSTKIAAVPAAPIAVANAQKSA
ADIPQAPKASLRRFLEKRKDRLTAKAPYPGSPSDVTPVKKEMSDGQPWLGLGPQIANPDLSLSKEASQ

AGPv4:

Zm00001d029448 (zim24 - ZIM-transcription factor 24)

Chr1: 71161670..71164215

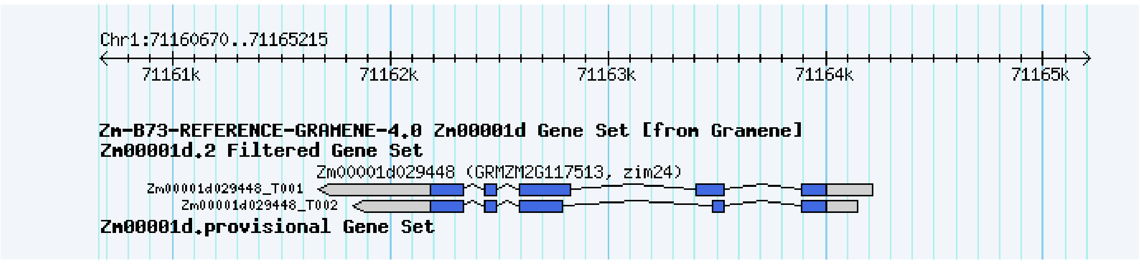

>Zm00001d029448 Genomic DNA

ATGAATGGGAATAAAGATGAGCCCAGTGCCTCTTCATTTTTATGCACGCCCAGCACGCCCAGACGGCGGAGGAAGGGGGA
AAGAGGAGGGCAGAGCAGCAACAGTACATCGAGCGAGGGAGTAGAGTAAATTGAATCAGGTTTCGTGTCGATTCGGAGGC
TTTCGCTTCTGCTTGCGGAGTTGCGGTGGAGATTTGAGGCCTGCGGCGAAGATGGCACCTGCGGAATCCGGGGAGAAGGC
GACCAGCTTCGCCATGGCCTGCAGCCTGCTCAGCCGCTACGTCAGGCAGAACGGTGCCGCCGCTGCCGATCTCGGCCTCG
CTATCAGAGGTGAAGCCTCTTCTCTTGTTGAGGAAAAATTTGGGGTCTCGAGGAGGTCGGTAGGTGTTCTTTATCTGTTG
GGAGTTTAGCGGTGTTTGTTGGGGAGAAGCGGTGAATTGAGGTGCGATTCATGCTAAAAGTTGCTATTTTGAGGTGCTCC
CGTAGTCGTCTCGTGTCAGATCTGGTCCCGAAGTCTTGACCTGGAAGCAAATATGTCACTTGTATTTGGTTTCGATCAAG
AATTTGGGCTTCTGGCTGATCTAACATCCGAATCTGATAGCTTCCTGTCCATCTCTGTACAGTCTATCTGAAAATTATCG
GCTGAACTTTTACTGAAGCAGAGTGCTCTATCCTGTTTCAGCTGAATCCGATGCAAAGAGGACGTCGACGGACATGGAAA
AGGGGGAGACGGGGAAGGAGACCATGGATCTCTTCCCCCAGATCGCTGGATTCGGCTCGGAGGCCGCCACGAAGGAAGCC
CCTGATGCGAGGTACTTGACGAAATAGATACCTCTCTGTTCTTTTGGGTTTCAGTTCACAGCCATTCTCATGTCATGTTA
GCATGGTACTATTGCTGTAGTCAGTTGATGACTGAAAACGTAAAAAAAAAAAAAACAATCTAGCTGCTGTGAGAAGCAGC
TCTTGGTGGCCAGTTTTTTATTTGTCAAACAGTTGTGCCTCAGATCGATGTAGATGGTTCGATCTCCACACACAGCAGTA
CAACCCAGATCTTTGTCTTCAGAAAATAGTGAGAAGTTAATTTATCATCTGAAACTGAATATTTGAAGAGTATTGTTCGT
AGGGCTTCTCACACTGCAAACCAGACTGCCAAACAATCTCCGGCAAAGCTTTCTGTGATGTGTGCCAAAGCTTTCTTGCT
ATGTCGTAGTGGTTGAATGGTAGAAGGTCCATCCATGAATCTGTAGAACTTGGCGCTCCGAAACTAAATGAATGAACAAT
TGGTGCCACGCAAGTTCTGAAACTTCAAAATTTTAACTCGTTGAAGTCGTTTCATGCGTATAAGTTAGCCACCAAATTTG
ACAACCGGAAACATCCTGATTCTCAGGGAGCCAGAGAAGCGCCAGCTGACTATCTTCTATGGCGGGAAGGTCCTTGTGTT
CGATGACTTCCCTGCTGAGAAGGCAAAAGACCTGATGCAAATGGCCAGCAAGGGTTCACCCGTAGCCCAGAACCCTGGCC
TGCTGCCTCCCTCCACAGCTGCAACCGTTACTGACAGCACCAAGATCGCAGCAGTGCCAGCCGCACCAATTGCTGTGGCC
AACGCCCAGAAGAGTGCAGCAGGTAGCTGACTCGTGCACCCCACCTTGTTCATATGCACGCGGCATTTCTCTGAATGATT
GATTTCTGTCTTGCTCACTGTTCCCAAATTGTATAATGTTTGCAGATATACCGCAGGCTCCCAAGGCATCTCTTCGCAGG
TTCCTTGAGAAGAGAAAGGATCGGTAGGTTACTCTTGCTGCTGTTGGGATGACAAGGCTGTTGTTTTAACGATACAGCTC
TTCGAGGAGGATCTATCTAATCTGTAATATTCCCTGTTTCAGCCTTACTGCGAAAGCACCATACCCGGGTTCCCCTTCAG
ATGTTACACCTGTGAAGAAGGAGATGTCGGATGGTCAGCCATGGCTTGGGCTGGGACCTCAGATCGCCAACCCTGATCTG
AGCTTGTCCAAGGAGGCGAGCCAGTGATCGATGATCCAGCATCACCATCTTCAAAACTGCGATACTCAACAGCCATGTGT
GGAAGTGTTCTGTTTTGGGCCCCTATCATTCCGGGTTTTTTTCGCATGGATAAATAGGTTGTACGAAAGTAAAGAACAC
AGCTAATGACGGACTTCAGTAAGGATTACCGGCGCTGGGGCAGGTTTGGCTGATCAGCCTTTGCTGGCCTGACCCAAGTG
GCCATTTTAGAAAGATGAAAATGTAATGTAGTAGTTGCTAGAACAAGTTGGCATCTTGGGATGTTCTGTGTTGTTTGGCT
TTGGCACTGGTGCGTGTCAGTGTCACAAAGCTATTTATTTTCACAGATACATGCTATATGATTTTTTTTATTATCTTCTG
GTTGTCTCTGTAGGGTTATGAAATATCACAGCTTTTCTTTCTAAGTGATTGTGATCAATGCCCCTGGACTATCAGAATGT
TGTTCTCTGAAGTTACAATCTACTACTCCCGTTGATCTAGTAAATTATAAGAAGTTTTGTGTTTTC

>Zm00001d029448_T001 cDNA
ATGAATGGGAATAAAGATGAGCCCAGTGCCTCTTCATTTTTATGCACGCCCAGCACGCCCAGACGGCGGAGGAAGGGGGA
AAGAGGAGGGCAGAGCAGCAACAGTACATCGAGCGAGGGAGTAGAGTAAATTGAATCAGGTTTCGTGTCGATTCGGAGGC
TTTCGCTTCTGCTTGCGGAGTTGCGGTGGAGATTTGAGGCCTGCGGCGAAGATGGCACCTGCGGAATCCGGGGAGAAGGC
GACCAGCTTCGCCATGGCCTGCAGCCTGCTCAGCCGCTACGTCAGGCAGAACGGTGCCGCCGCTGCCGATCTCGGCCTCG
CTATCAGAGCTGAATCCGATGCAAAGAGGACGTCGACGGACATGGAAAAGGGGGAGACGGGGAAGGAGACCATGGATCTC
TTCCCCCAGATCGCTGGATTCGGCTCGGAGGCCGCCACGAAGGAAGCCCCTGATGCGAGGGAGCCAGAGAAGCGCCAGCT
GACTATCTTCTATGGCGGGAAGGTCCTTGTGTTCGATGACTTCCCTGCTGAGAAGGCAAAAGACCTGATGCAAATGGCCA
GCAAGGGTTCACCCGTAGCCCAGAACCCTGGCCTGCTGCCTCCCTCCACAGCTGCAACCGTTACTGACAGCACCAAGATC
GCAGCAGTGCCAGCCGCACCAATTGCTGTGGCCAACGCCCAGAAGAGTGCAGCAGATATACCGCAGGCTCCCAAGGCATC
TCTTCGCAGGTTCCTTGAGAAGAGAAAGGATCGCCTTACTGCGAAAGCACCATACCCGGGTTCCCCTTCAGATGTTACAC
CTGTGAAGAAGGAGATGTCGGATGGTCAGCCATGGCTTGGGCTGGGACCTCAGATCGCCAACCCTGATCTGAGCTTGTCC
AAGGAGGCGAGCCAGTGATCGATGATCCAGCATCACCATCTTCAAAACTGCGATACTCAACAGCCATGTGTGGAAGTGTT
CTGTTTTGGGCCCCTATCATTCCGGGTTTTTTTTCGCATGGATAAATAGGTTGTACGAAAGTAAAGAACACAGCTAATGA
CGGACTTCAGTAAGGATTACCGGCGCTGGGGCAGGTTTGGCTGATCAGCCTTTGCTGGCCTGACCCAAGTGGCCATTTTA
GAAAGATGAAAATGTAATGTAGTAGTTGCTAGAACAAGTTGGCATCTTGGGATGTTCTGTGTTGTTTGGCTTTGGCACTG
GTGCGTGTCAGTGTCACAAAGCTATTTATTTTCACAGATACATGCTATATGATTTTTTTTATTATCTTCTGGTTGTCTCT
GTAGGGTTATGAAATATCACAGCTTTTCTTTCTAAGTGATTGTGATCAATGCCCCTGGACTATCAGAATGTTGTTCTCTG
AAGTTACAATCTACTACTCCCGTTGATCTAGTAAATTATAAGAAGTTTTGTGTTTTC
>Zm00001d029448_P001 peptide
MAPAESGEKATSFAMACSLLSRYVRQNGAAAADLGLAIRAESDAKRTSTDMEKGETGKETMDLFPQIAGFGSEAATKEAP
DAREPEKRQLTIFYGGKVLVFDDFPAEKAKDLMQMASKGSPVAQNPGLLPPSTAATVTDSTKIAAVPAAPIAVANAQKSA
ADIPQAPKASLRRFLEKRKDRLTAKAPYPGSPSDVTPVKKEMSDGQPWLGLGPQIANPDLSLSKEASQ

ZmJAZ 4 (TIFY8)

B73 RefGen_v3:

GRMZM2G024680 (zim21 - ZIM-transcription factor 21)

Chr1: 244829629..244830954

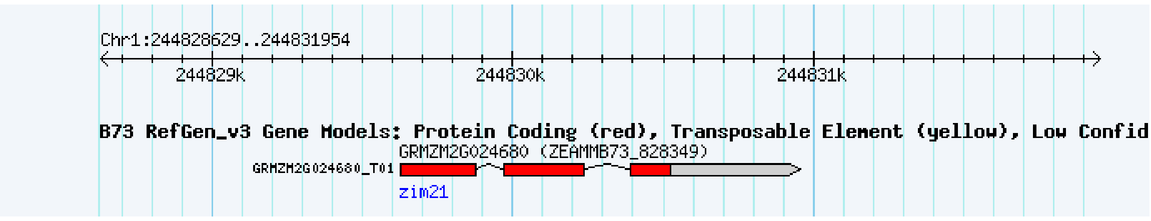

>GRMZM2G024680 Genomic DNA
ATGGCCGCCTCCGGGAACAACAGATTCTCCCTTACGTGCGCTCGCTTGCGCAAGTTCATGATGGAGCAGAACAGGAAGGT
GCGGATGGATGACCTCGTCGGCTCCTCCTCGTTCCAGCGGCCGCTCCAACTGACACCGGTCCCTGTAGCCACGGGGCCGG
CGGCGGCGGCGGCCTGGGAGACCGGCACGAGCAGGACGACCTTGCCGCTGTTCCCCGTCCGCACTGCTGGCACCGCCGAG
ATCACCAGGTGAACCGTAGTTATTATGTATTAATTGATCTGCTGGCTGACGTGTTCTTGGGCTAGCTGGTTTAATTTCTC
GATCGGTTTGTTCGTTGCAGGCCAGAGGAGGGCAAGGCCACCCTGACCATCTTCTACCAGGGGCAGGTGTCCACGTTCCA
CCACTTCCCAGCAGACAGAGCCAAGGTCCTGATGCAAATGGCAAGTTCTGTGACCGGGAACACGCCGGAGAAAGGGGTGG
CGGTGGCGACCGCCGTGCCAAAGAAGGCGCAGACCAGCGATGACCAGCCATCGCCTGCAGGGGCAGGCATGCCGCCCATC

GCAAGGAAGCTGACGCTGCAGAACTTTCTCAGGAAAAGGAAGAACAGGTGAGGAAGATCACATGCACTTTCACATCTCTG
AGATCCACTATCTATCTATCTATATATATGTGTCCTATATATATATATATATCAGTAATTTGGAATTTTGGGTAGAACAG
TGTGGATCGTAATGCACACGTGTGTGTTATTATTACTCGCAGGATCGCAGGCACCGACGACGCTGACCGCAACGAGGATG
CTTCGCCGTGGAAGAAGAGAGACTCCGCCGCCGGCGCCGGCGGCACCCCCGCGGGAGACGTGCCCGACGATGCCTCGTGG
CTCGCGCTCAGGCTCTGAAACAGTGAAACCCTTCACCGCTTATTACATGAGAATAATAATATGGAAGAGAAGGATACATC
CTCCGAGATGATACAATAATAATAATGCCGTGTCTGTCCCAGGATTCTTAGGACCTCCGAAGAATTCTTAGGCACTGTGT
CTGTCGTCGAGAGCCGTAGCTTATCAAAAAGGGGAAGCATATCTTTAGAAAGCTCTCTTTCGAAAAACTTGAGAAGCTGT
TCTAAGTGAGCTTACCTACTTGTAGTACAAAATGTAGTTTTTATTTGTCAATTTGAACTTTGAAGAAGCAGAGACCCTAC
CTGCTGGGGCGGAGCTTTTGGCCTTTCAATAGCTTTTTGGAGAAGCAGAAGCTTCCCCAAATAGAGCTTAATTACATTAC
TGTTGGCAGGACTAATAATAATGCGGCGTCAAGCAGTTATAAACTA
>GRMZM2G024680_T01 cDNA
ATGGCCGCCTCCGGGAACAACAGATTCTCCCTTACGTGCGCTCGCTTGCGCAAGTTCATGATGGAGCAGAACAGGAAGGT
GCGGATGGATGACCTCGTCGGCTCCTCCTCGTTCCAGCGGCCGCTCCAACTGACACCGGTCCCTGTAGCCACGGGGCCGG
CGGCGGCGGCGGCCTGGGAGACCGGCACGAGCAGGACGACCTTGCCGCTGTTCCCCGTCCGCACTGCTGGCACCGCCGAG
ATCACCAGGCCAGAGGAGGGCAAGGCCACCCTGACCATCTTCTACCAGGGGCAGGTGTCCACGTTCCACCACTTCCCAGC
AGACAGAGCCAAGGTCCTGATGCAAATGGCAAGTTCTGTGACCGGGAACACGCCGGAGAAAGGGGTGGCGGTGGCGACCG
CCGTGCCAAAGAAGGCGCAGACCAGCGATGACCAGCCATCGCCTGCAGGGGCAGGCATGCCGCCCATCGCAAGGAAGCTG
ACGCTGCAGAACTTTCTCAGGAAAAGGAAGAACAGGATCGCAGGCACCGACGACGCTGACCGCAACGAGGATGCTTCGCC
GTGGAAGAAGAGAGACTCCGCCGCCGGCGCCGGCGGCACCCCCGCGGGAGACGTGCCCGACGATGCCTCGTGGCTCGCGC
TCAGGCTCTGAAACAGTGAAACCCTTCACCGCTTATTACATGAGAATAATAATATGGAAGAGAAGGATACATCCTCCGAG
ATGATACAATAATAATAATGCCGTGTCTGTCCCAGGATTCTTAGGACCTCCGAAGAATTCTTAGGCACTGTGTCTGTCGT
CGAGAGCCGTAGCTTATCAAAAAGGGGAAGCATATCTTTAGAAAGCTCTCTTTCGAAAAACTTGAGAAGCTGTTCTAAGT
GAGCTTACCTACTTGTAGTACAAAATGTAGTTTTTATTTGTCAATTTGAACTTTGAAGAAGCAGAGACCCTACCTGCTGG
GGCGGAGCTTTTGGCCTTTCAATAGCTTTTTGGAGAAGCAGAAGCTTCCCCAAATAGAGCTTAATTACATTACTGTTGGC
AGGACTAATAATAATGCGGCGTCAAGCAGTTATAAACTA

>GRMZM2G024680_P01 peptide
MAASGNNRFSLTCARLRKFMMEQNRKVRMDDLVGSSSFQRPLQLTPVPVATGPAAAAAWETGTSRTTLPLFPVRTAGTAE
ITRPEEGKATLTIFYQGQVSTFHHFPADRAKVLMQMASSVTGNTPEKGVAVATAVPKKAQTSDDQPSPAGAGMPPIARKL
TLQNFLRKRKNRIAGTDDADRNEDASPWKKRDSAAGAGGTPAGDVPDDASWLALRL
AGPv4:
Zm00001d033048 (zim21 - ZIM-transcription factor 21)
Chr1: 248467942..248474649
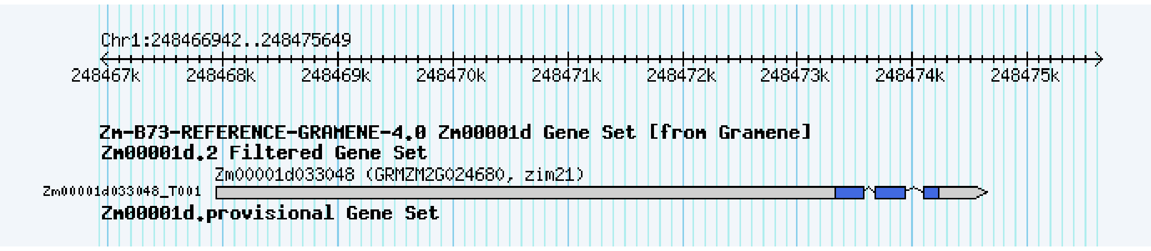

>Zm00001d033048 Genomic DNA
TGGACCTCCACCTGATCCACCACACATGATGTTGCATGTCCGTCCAATGCACTTCTGCTAGATTCCATCATGCAAGGCGG
TCATCATGCATAGGGGTCATCGTGTCAGGCATTCTATTGGGCCAATGTCATAGGGAGGAAGAGAGACGCCATGATAGGGA
GAGTTCGTCCTTCATATGAGAGAGAAATGTGGGGAACACAGAGGATAGAGAGAAGGTGGTATGACCAAGGATCTACATGC
TTCACACACATACATTTTCAATGTGACACGCAAGCGCTTGCACATGTTTCACCCCCTATTAGCCACCTAAAGAGAACACA

GACATTGAGGGGAGATACATACACATGCTTCTTTGAGCACATCTTTTCATTGTAAACTCTTTTCATGAACTAGATTTTGA
TGACTGATTGTATTCCAAACAAGTGATCATATATTATTAAAATGTTTTTATAAAACAACGGTGAAAATGCTATTAAAGAA
ATACTATTTATGTAGTAGTGTTTGTAGAAGTTAGAAGAAAATAAGCATTGGTGTACATATCTTTGCAAGAGGTATGAGGC
AGAAGTTAGAAAATGATGGGCTTGCAAGAGAACCACACATGTCGAACTTCTTTATCGAGTGCTTCTTATGGAAGTAAATG
TTATCCTTTGTTGTTATGTATATATGTCTTGCCTTATAAAACACGTGAGCATTTAACCATCTTAATTTATGCTTGGCCTT
GTCGTGGGGATGTGTCAACAAGATACACATCACGACATTCTCATGGGCCTAGTTTGTGAGGGTGCATGAAGCCCACTTTT
ATCATTGGACACTTACCACAACATGAGACCAACAAAATTATGATATACTTAACAAGGCTCCCATCATGGGATAAGAAACC
ATAAAATATTATGGCCACTTTGTATATATTTAGTGATCTCGATTAATTTACTATGTTGACCAAACATAGACTTATGCACA
AAACCTAACTATTAACCCAGCCGCTAGATAGTTAAGTAGAAAACATTTGCGAAATAGGGAGAAATAATATTAGTAAATGA
AATTAGATTTAAAATGACGCGGAACTGGATTTGGTGGAGTATGAATTGTTGTACTCAAAATTAGGACGGAAGGAGGGGTG
GAATAGGAATAATCTCATGAATTTTAGATTACTAATGCTTCCTATATATAAAAAAATGGAAAAGAGATCATGTCTATAAA
CTTATGGTCAAAGGTGATATCAATCAAGAATTTGAAATAGTTGCATTAATAGATCTAATTTACATTTTGTTGAATTTCAA
TCTATCACTTATTACTTTAGATGAGCTCCTGCCATCGATTCTGAAAGAAAATTTTATTTTCGTCCATATAAAGTGTGTGG
AATCAAATTTTGACCAATAAGCTAGAATCATTGAAGCCTACTTTGATGCATCGTTCGATGGCGATTCAGAAAACTTTGCT
CCATGTACATGTCTGCTTGTAGTATCAAGATGAACTATTTGTTTTTTCTCTTGCCATTTTAAATGTTAATACTTTTATTT
CACTTTACATCACATGTTCTTGGCATTAACAATTTAATGGAAGGTGTTAACATATCAACCTACTTCAGATGTAGCTTGCC
TATACCATTCTTTGAGAAGATACCCATAATATCCATTGATAATTTAGCAGTTTATTTACTTAATAGGGCTATGTTTTCTT
TATTCTAATGGGTTCTACCTTCTAGACAACAAAGAAGATCCTCCAAATACAATGAGGGAATGACAATACATAATTTTAAA
AATGGAACATTTTTGTGTTTTATTAATGACATATTTTTGATTACTTTGAAAGCACATTATTAATTGTGCAACATAATCAT
AACACACTCCTTGTTGCTCTTGCTTTTATCAAGTTCAAGGAAGCATTAGTAAATAATATGTTACACCAAGGCACCCTAAT
GAGGACAATATTCGCTCATAAGTTTGTGGAAAGAGGTGGGGATTATGTTGATATAGTTCCTCCAATCCACTATAAGTATA
CAAATTGGGGTATTTCATTCATCAAGAATAACCCAAAAATAAGTTATTAAATACTCTTGATAGCACATGAGCACGATGTC
CATTAATCGTCAACGCAATAAGAATTCGAGTGACAATGTTGCTTATGTGGACTCGAGTATAACCTATTTACACTAAGTTA
ACTATTTACTGATTTAGTACTTTTAATTCTGGTCTACGCCAAGCCTCGATACATAATATGATATACTTAGACAACTGTGT
AAGATGTTGTACAAGCATATGTACAATTCCAGTAGTAGTGTTATGTGTAAGGGAGGCTCTAGGACACACATATTGAGTAT
GTTTTACTCCTAGGTGTGTGTGTGTGTGTGGAAGTTGTAGAAAAAGAGATCTCTTATATTAGACGACACCAACAAACCAA
TATTGTATGTGGTATGTGATGACACAAGCATTATATCTACAAAATAGGGTGTTTTTCTTTCCTTGACTAGCAAAAGAATG
CTAATAATAAGCCACCAAACAAATATATTGCTTTGTTCATAATGAAAATAAAGGATACCATAAATTTGCCAACATTGTAT
TCCTTCTAGTAAGCAATATTGTTCATAATTAACCTTGAATGTAAATTTGGAAATTCCTTTCAACATTTAGAGTGTAGCTA
CTCCTTTGTCTATATCTCAAATTTTAGGTTGCATGTAACCAGATGAAATGTATACATAAGGACTATGTGACAATACTAGA
TCATTGATGGTGCATGTATATATACTACTATGACAACACTATAACAATAAACTAAAACAATATAGTCTCCATTATTTTTT
CACATAGTAAGATTAATTTGGAGTATCACAAAATGACTATATAAACATGCTCAATGTGATAAATGAGTACACAATATGCA
TCCATACACAATATGTCACTCTCCAAGGGCATGCACAAACACTAACCATAACATAATTAGTTTCCTATTTTGAAACATAT
AGATCACAAACCTGCATATTACAATTATTATAGATCTTCATCGGTATCAATTTCACCCCTCTTTAGTGAAAGTGGGCATG
TAATGAGGTGAGATATTATCACTAGTTCAAATTGTAGTGAAAAATAGTCATCACTATTGAACCCTTTTACGACGGTTGTG
ACCATCAGAAAAATAAGAAAAAGCATTGACCGAGCCCACTAACTTAACTTATCTGCCACTGTTATAGAGGGTGTATCCTT
GAGTCATTGCCAAAGGGGCCACACCGCTCTCGCTACTATTAGGATGAGGGTTAGGTGGGAGGAGGCAGAGGGGAAGCGAG
AGAGTAAATATAGAGAGAGATATAAAGAAGAAAGGTGCATGCAAAACTTATGATAATAAAGGAATGTGACCAAGATGAGG
TTGAAAGGGAAGGCTAGAGTTTCCTATTATATATTCTACTAGAGATGGTGGTCCTACCCGCTTGTATTTTTTTCTAACAA
GTGAGTGCATGGGTTCAAAATTTCACCACATGAATGCGGATTTGTCACACCACCCATTAGCGCAAAAATACATATATTTC
TATCTATTAGAGTGATATTGTAACTCATAGGCGCGAGTACCGACGAGTATTATAACGACCTGATGTGCATACGATCCCGA
CAAGGGCATCTATGAGCATAACATCTTACCCCTCGCAGGTAGTGAATGGAAGTGCATGTATAAATTTTTACAGAATAGAT
AATTTGTTGTGGGTGTGTATTTAGTCCACTCGCACCTTACTTAACATATCGCCATCACTATACTTAGGCTATCCTTGAGC
CAAGAATGAAAAATGGAATAGACGACCAGAGAGGGGGTGAATGGAAGCTAATAAAAAAATATTTACACTTAAGCAATCTT

AGCTTATTTCCGTAAAGTTCAGACCAAACCTCTAAGACACCAAAACTGCACAAAGGTCCGAGATAAAAAAACAATTCAAC
CAAGTGATGGTTGGATGCCTTGAATAAGTTTTCCAGCAAGAATATCAATCTACCGAAACAAGCACCGGAAAGACTGAGAA
GAGGCAAATCAAAGACTTTCTCTAAATAGCCCAGTAGTGTCGCTCTCCTCGCTGTCATTGCCATGGCCTGACAGTGTTGC
TCGTTATCTGACTAGTAGTGTCGCTCACGCTGTGTGGTAGTGTCACTCGACCAAAACTTCCTAAAATTAGAGGAACCTTT
GTAGACAACAACAACGAGAGGATTGAAGACAATTCCAAAATTTGAACTTCAAAAGATCAACCCTTAGATAGATCAGGAAT
TACGAACTTGAGTTTGGGTTTTCACAAAAAGCTCAAAATTATCGGGGACTTAATGCTTAAGTTGATTTGCCAATACCAGA
GGTGGTAATGGTTTTAGAAAAGATTTCTCAAGGCCTTAATCAGCCCAAATGCCCTAACCTTATATTGTAGCTCATTGGTG
CGAGTACGATCCCCCACGAGTACATCTTTAGTGCTAGTTTGGAAACTCAAATCCCCTTGGGGATTGAAGGGGATTGGAGA
GGAAATTAGTTTATTTCCACCTTAACCCCCTCCAATCCCGAAGGGGATATGAGGTTCCCAAAGTAGCCCTCAAGTATAAC
ATTCTCGTGGGTGTAAGTGCGAGTATTAAATTTTACATAAGAGGAAATTTGTTGCGGGTGTGTATTTAGTCTACCCATGC
ACACCTTGCCCAACTTATTGTCATCCATATACTTCCAGGCTTGCCTTTGAGACAGATAACAACAGTGTAAATATTATCAC
CAATATTTGTTATACCAAACTGTTGGTGCAAATTGTTCTCACCGTTAGGGTGGTAATGGATCACGATCCAAATGCTTCTT
CACAAATGTTAGGAATCTAAATAAATTTTAGTTAAAAAATGAATAGAAATAAAACCTGATTCTAATCTGATTTGGTCCTT
AAATAAATTTAAAGCCTATTGTCACCCCTAAGGACTTGTTTAGGATCGGAGCAAGAGTAATGTAGGGGATTGAGAGAGTT
AGTGATTGCCCCCACATGAAAGCCGATCATCTCAGTCGTCAACTCTTGGCCCTAAAAATGACACAACACATTAGATTAAC
CGGTACAAAATTAGAGGATAGAGAGGTGAGGGAAGTAAATGCATGCAAAATAACCCACGGAGCAAAAGAAGGGACTACAG
AAATAGAGCAGTGTGTACGTTGTTGCACACCAGTGGCTGTGGCTCTAGCCAGGGTCCTCCCACGCTCTCGTCGGCCCTCC
ACTAGCATGGCCTCGCATTTCACGTGCTCCCTCGCGCGCCCCGAGGCGACGCACAAACAAACGAAGGCTACCCGGGCCTC
GCGGCTATAATAGCAGGCGCCCGCCCACGCGAGAGCGAGAGAGAGAGAGACCGAAGCACCAGCACCACCAGTGCCATACA
TACCAAACGAGGACGGTGAGGAGAGGAGATGGCCGCCTCCGGGAACAACAGATTCTCCCTTACGTGCGCTCGCTTGCGCA
AGTTCATGATGGAGCAGAACAGGAAGGTGCGGATGGATGACCTCGTCGGCTCCTCCTCGTTCCAGCGGCCGCTCCAACTG
ACACCGGTCCCTGTAGCCACGGGGCCGGCGGCGGCGGCGGCCTGGGAGACCGGCACGAGCAGGACGACCTTGCCGCTGTT
CCCCGTCCGCACTGCTGGCACCGCCGAGATCACCAGGTGAACCGTAGTTATTATGTATTAATTGATCTGCTGGCTGACGT
GTTCTTGGGCTAGCTGGTTTAATTTCTCGATCGGTTTGTTCGTTGCAGGCCAGAGGAGGGCAAGGCCACCCTGACCATCT
TCTACCAGGGGCAGGTGTCCACGTTCCACCACTTCCCAGCAGACAGAGCCAAGGTCCTGATGCAAATGGCAAGTTCTGTG
ACCGGGAACACGCCGGAGAAAGGGGTGGCGGTGGCGACCGCCGTGCCAAAGAAGGCGCAGACCAGCGATGACCAGCCATC
GCCTGCAGGGGCAGGCATGCCGCCCATCGCAAGGAAGCTGACGCTGCAGAACTTTCTCAGGAAAAGGAAGAACAGGTGAG
GAAGATCACATGCACTTTCACATCTCTGAGATCCACTATCTATCTATCTATATATATGTGTCCTATATATATATATATAT
CAGTAATTTGGAATTTTGGGTAGAACAGTGTGGATCGTAATGCACACGTGTGTGTTATTATTACTCGCAGGATCGCAGGC
ACCGACGACGCTGACCGCAACGAGGATGCTTCGCCGTGGAAGAAGAGAGACTCCGCCGCCGGCGCCGGCGGCACCCCCGC
GGGAGACGTGCCCGACGATGCCTCGTGGCTCGCGCTCAGGCTCTGAAACAGTGAAACCCTTCACCGCTTATTACATGAGA
ATAATAATATGGAAGAGAAGGATACATCCTCCGAGATGATACAATAATAATAATGCCGTGTCTGTCCCAGGATTCTTAGG
ACCTCCGAAGAATTCTTAGGCACTGTGTCTGTCGTCGAGAGCCGTAGCTTATCAAAAAGGGGAAGCATATCTTTAGAAAG
CTCTCTTTCGAAAAACTTGAGAAGCTGTTCTAAGTGAGCTTACCTACTTGTAGTACAAAATGTAGTTTTTATTTGTCAAT
TTGAACTTTGAAGAAGCAGAGACCCTACCTGCTGGGGCGGAGCTTTTGGCCTTTCAATAGCTTTTTGGAGAAGCAGAAGC
TTCCCCAAATAGAGCTTAATTACATTACTGTTGGCAGGACTAATAATAATGCGGCGTCAAGCAGTTAT
>Zm00001d033048_T001 cDNA
TGGACCTCCACCTGATCCACCACACATGATGTTGCATGTCCGTCCAATGCACTTCTGCTAGATTCCATCATGCAAGGCGG
TCATCATGCATAGGGGTCATCGTGTCAGGCATTCTATTGGGCCAATGTCATAGGGAGGAAGAGAGACGCCATGATAGGGA
GAGTTCGTCCTTCATATGAGAGAGAAATGTGGGGAACACAGAGGATAGAGAGAAGGTGGTATGACCAAGGATCTACATGC
TTCACACACATACATTTTCAATGTGACACGCAAGCGCTTGCACATGTTTCACCCCCTATTAGCCACCTAAAGAGAACACA
GACATTGAGGGGAGATACATACACATGCTTCTTTGAGCACATCTTTTCATTGTAAACTCTTTTCATGAACTAGATTTTGA
TGACTGATTGTATTCCAAACAAGTGATCATATATTATTAAAATGTTTTTATAAAACAACGGTGAAAATGCTATTAAAGAA
ATACTATTTATGTAGTAGTGTTTGTAGAAGTTAGAAGAAAATAAGCATTGGTGTACATATCTTTGCAAGAGGTATGAGGC

AGAAGTTAGAAAATGATGGGCTTGCAAGAGAACCACACATGTCGAACTTCTTTATCGAGTGCTTCTTATGGAAGTAAATG
TTATCCTTTGTTGTTATGTATATATGTCTTGCCTTATAAAACACGTGAGCATTTAACCATCTTAATTTATGCTTGGCCTT
GTCGTGGGGATGTGTCAACAAGATACACATCACGACATTCTCATGGGCCTAGTTTGTGAGGGTGCATGAAGCCCACTTTT
ATCATTGGACACTTACCACAACATGAGACCAACAAAATTATGATATACTTAACAAGGCTCCCATCATGGGATAAGAAACC
ATAAAATATTATGGCCACTTTGTATATATTTAGTGATCTCGATTAATTTACTATGTTGACCAAACATAGACTTATGCACA
AAACCTAACTATTAACCCAGCCGCTAGATAGTTAAGTAGAAAACATTTGCGAAATAGGGAGAAATAATATTAGTAAATGA
AATTAGATTTAAAATGACGCGGAACTGGATTTGGTGGAGTATGAATTGTTGTACTCAAAATTAGGACGGAAGGAGGGGTG
GAATAGGAATAATCTCATGAATTTTAGATTACTAATGCTTCCTATATATAAAAAAATGGAAAAGAGATCATGTCTATAAA
CTTATGGTCAAAGGTGATATCAATCAAGAATTTGAAATAGTTGCATTAATAGATCTAATTTACATTTTGTTGAATTTCAA
TCTATCACTTATTACTTTAGATGAGCTCCTGCCATCGATTCTGAAAGAAAATTTTATTTTCGTCCATATAAAGTGTGTGG
AATCAAATTTTGACCAATAAGCTAGAATCATTGAAGCCTACTTTGATGCATCGTTCGATGGCGATTCAGAAAACTTTGCT
CCATGTACATGTCTGCTTGTAGTATCAAGATGAACTATTTGTTTTTTCTCTTGCCATTTTAAATGTTAATACTTTTATTT
CACTTTACATCACATGTTCTTGGCATTAACAATTTAATGGAAGGTGTTAACATATCAACCTACTTCAGATGTAGCTTGCC
TATACCATTCTTTGAGAAGATACCCATAATATCCATTGATAATTTAGCAGTTTATTTACTTAATAGGGCTATGTTTTCTT
TATTCTAATGGGTTCTACCTTCTAGACAACAAAGAAGATCCTCCAAATACAATGAGGGAATGACAATACATAATTTTAAA
AATGGAACATTTTTGTGTTTTATTAATGACATATTTTTGATTACTTTGAAAGCACATTATTAATTGTGCAACATAATCAT
AACACACTCCTTGTTGCTCTTGCTTTTATCAAGTTCAAGGAAGCATTAGTAAATAATATGTTACACCAAGGCACCCTAAT
GAGGACAATATTCGCTCATAAGTTTGTGGAAAGAGGTGGGGATTATGTTGATATAGTTCCTCCAATCCACTATAAGTATA
CAAATTGGGGTATTTCATTCATCAAGAATAACCCAAAAATAAGTTATTAAATACTCTTGATAGCACATGAGCACGATGTC
CATTAATCGTCAACGCAATAAGAATTCGAGTGACAATGTTGCTTATGTGGACTCGAGTATAACCTATTTACACTAAGTTA
ACTATTTACTGATTTAGTACTTTTAATTCTGGTCTACGCCAAGCCTCGATACATAATATGATATACTTAGACAACTGTGT
AAGATGTTGTACAAGCATATGTACAATTCCAGTAGTAGTGTTATGTGTAAGGGAGGCTCTAGGACACACATATTGAGTAT
GTTTTACTCCTAGGTGTGTGTGTGTGTGTGGAAGTTGTAGAAAAAGAGATCTCTTATATTAGACGACACCAACAAACCAA
TATTGTATGTGGTATGTGATGACACAAGCATTATATCTACAAAATAGGGTGTTTTTCTTTCCTTGACTAGCAAAAGAATG
CTAATAATAAGCCACCAAACAAATATATTGCTTTGTTCATAATGAAAATAAAGGATACCATAAATTTGCCAACATTGTAT
TCCTTCTAGTAAGCAATATTGTTCATAATTAACCTTGAATGTAAATTTGGAAATTCCTTTCAACATTTAGAGTGTAGCTA
CTCCTTTGTCTATATCTCAAATTTTAGGTTGCATGTAACCAGATGAAATGTATACATAAGGACTATGTGACAATACTAGA
TCATTGATGGTGCATGTATATATACTACTATGACAACACTATAACAATAAACTAAAACAATATAGTCTCCATTATTTTTT
CACATAGTAAGATTAATTTGGAGTATCACAAAATGACTATATAAACATGCTCAATGTGATAAATGAGTACACAATATGCA
TCCATACACAATATGTCACTCTCCAAGGGCATGCACAAACACTAACCATAACATAATTAGTTTCCTATTTTGAAACATAT
AGATCACAAACCTGCATATTACAATTATTATAGATCTTCATCGGTATCAATTTCACCCCTCTTTAGTGAAAGTGGGCATG
TAATGAGGTGAGATATTATCACTAGTTCAAATTGTAGTGAAAAATAGTCATCACTATTGAACCCTTTTACGACGGTTGTG
ACCATCAGAAAAATAAGAAAAAGCATTGACCGAGCCCACTAACTTAACTTATCTGCCACTGTTATAGAGGGTGTATCCTT
GAGTCATTGCCAAAGGGGCCACACCGCTCTCGCTACTATTAGGATGAGGGTTAGGTGGGAGGAGGCAGAGGGGAAGCGAG
AGAGTAAATATAGAGAGAGATATAAAGAAGAAAGGTGCATGCAAAACTTATGATAATAAAGGAATGTGACCAAGATGAGG
TTGAAAGGGAAGGCTAGAGTTTCCTATTATATATTCTACTAGAGATGGTGGTCCTACCCGCTTGTATTTTTTTCTAACAA
GTGAGTGCATGGGTTCAAAATTTCACCACATGAATGCGGATTTGTCACACCACCCATTAGCGCAAAAATACATATATTTC
TATCTATTAGAGTGATATTGTAACTCATAGGCGCGAGTACCGACGAGTATTATAACGACCTGATGTGCATACGATCCCGA
CAAGGGCATCTATGAGCATAACATCTTACCCCTCGCAGGTAGTGAATGGAAGTGCATGTATAAATTTTTACAGAATAGAT
AATTTGTTGTGGGTGTGTATTTAGTCCACTCGCACCTTACTTAACATATCGCCATCACTATACTTAGGCTATCCTTGAGC
CAAGAATGAAAAATGGAATAGACGACCAGAGAGGGGGTGAATGGAAGCTAATAAAAAAATATTTACACTTAAGCAATCTT
AGCTTATTTCCGTAAAGTTCAGACCAAACCTCTAAGACACCAAAACTGCACAAAGGTCCGAGATAAAAAAACAATTCAAC
CAAGTGATGGTTGGATGCCTTGAATAAGTTTTCCAGCAAGAATATCAATCTACCGAAACAAGCACCGGAAAGACTGAGAA
GAGGCAAATCAAAGACTTTCTCTAAATAGCCCAGTAGTGTCGCTCTCCTCGCTGTCATTGCCATGGCCTGACAGTGTTGC

TCGTTATCTGACTAGTAGTGTCGCTCACGCTGTGTGGTAGTGTCACTCGACCAAAACTTCCTAAAATTAGAGGAACCTTT
GTAGACAACAACAACGAGAGGATTGAAGACAATTCCAAAATTTGAACTTCAAAAGATCAACCCTTAGATAGATCAGGAAT
TACGAACTTGAGTTTGGGTTTTCACAAAAAGCTCAAAATTATCGGGGACTTAATGCTTAAGTTGATTTGCCAATACCAGA
GGTGGTAATGGTTTTAGAAAAGATTTCTCAAGGCCTTAATCAGCCCAAATGCCCTAACCTTATATTGTAGCTCATTGGTG
CGAGTACGATCCCCCACGAGTACATCTTTAGTGCTAGTTTGGAAACTCAAATCCCCTTGGGGATTGAAGGGGATTGGAGA
GGAAATTAGTTTATTTCCACCTTAACCCCCTCCAATCCCGAAGGGGATATGAGGTTCCCAAAGTAGCCCTCAAGTATAAC
ATTCTCGTGGGTGTAAGTGCGAGTATTAAATTTTACATAAGAGGAAATTTGTTGCGGGTGTGTATTTAGTCTACCCATGC
ACACCTTGCCCAACTTATTGTCATCCATATACTTCCAGGCTTGCCTTTGAGACAGATAACAACAGTGTAAATATTATCAC
CAATATTTGTTATACCAAACTGTTGGTGCAAATTGTTCTCACCGTTAGGGTGGTAATGGATCACGATCCAAATGCTTCTT
CACAAATGTTAGGAATCTAAATAAATTTTAGTTAAAAAATGAATAGAAATAAAACCTGATTCTAATCTGATTTGGTCCTT
AAATAAATTTAAAGCCTATTGTCACCCCTAAGGACTTGTTTAGGATCGGAGCAAGAGTAATGTAGGGGATTGAGAGAGTT
AGTGATTGCCCCCACATGAAAGCCGATCATCTCAGTCGTCAACTCTTGGCCCTAAAAATGACACAACACATTAGATTAAC
CGGTACAAAATTAGAGGATAGAGAGGTGAGGGAAGTAAATGCATGCAAAATAACCCACGGAGCAAAAGAAGGGACTACAG
AAATAGAGCAGTGTGTACGTTGTTGCACACCAGTGGCTGTGGCTCTAGCCAGGGTCCTCCCACGCTCTCGTCGGCCCTCC
ACTAGCATGGCCTCGCATTTCACGTGCTCCCTCGCGCGCCCCGAGGCGACGCACAAACAAACGAAGGCTACCCGGGCCTC
GCGGCTATAATAGCAGGCGCCCGCCCACGCGAGAGCGAGAGAGAGAGAGACCGAAGCACCAGCACCACCAGTGCCATACA
TACCAAACGAGGACGGTGAGGAGAGGAGATGGCCGCCTCCGGGAACAACAGATTCTCCCTTACGTGCGCTCGCTTGCGCA
AGTTCATGATGGAGCAGAACAGGAAGGTGCGGATGGATGACCTCGTCGGCTCCTCCTCGTTCCAGCGGCCGCTCCAACTG
ACACCGGTCCCTGTAGCCACGGGGCCGGCGGCGGCGGCCTGGGAGACCGGCACGAGCAGGACGACCTTGCCGCTGTT
CCCCGTCCGCACTGCTGGCACCGCCGAGATCACCAGGCCAGAGGAGGGCAAGGCCACCCTGACCATCTTCTACCAGGGGC
AGGTGTCCACGTTCCACCACTTCCCAGCAGACAGAGCCAAGGTCCTGATGCAAATGGCAAGTTCTGTGACCGGGAACACG
CCGGAGAAAGGGGTGGCGGTGGCGACCGCCGTGCCAAAGAAGGCGCAGACCAGCGATGACCAGCCATCGCCTGCAGGGGC
AGGCATGCCGCCCATCGCAAGGAAGCTGACGCTGCAGAACTTTCTCAGGAAAAGGAAGAACAGGATCGCAGGCACCGACG
ACGCTGACCGCAACGAGGATGCTTCGCCGTGGAAGAAGAGAGACTCCGCCGCCGGCGCCGGCGGCACCCCCGCGGGAGAC
GTGCCCGACGATGCCTCGTGGCTCGCGCTCAGGCTCTGAAACAGTGAAACCCTTCACCGCTTATTACATGAGAATAATAA
TATGGAAGAGAAGGATACATCCTCCGAGATGATACAATAATAATAATGCCGTGTCTGTCCCAGGATTCTTAGGACCTCCG
AAGAATTCTTAGGCACTGTGTCTGTCGTCGAGAGCCGTAGCTTATCAAAAAGGGGAAGCATATCTTTAGAAAGCTCTCTT
TCGAAAAACTTGAGAAGCTGTTCTAAGTGAGCTTACCTACTTGTAGTACAAAATGTAGTTTTTATTTGTCAATTTGAACT
TTGAAGAAGCAGAGACCCTACCTGCTGGGGCGGAGCTTTTGGCCTTTCAATAGCTTTTTGGAGAAGCAGAAGCTTCCCCA
AATAGAGCTTAATTACATTACTGTTGGCAGGACTAATAATAATGCGGCGTCAAGCAGTTAT
>Zm00001d033048_P001 peptide
MAASGNNRFSLTCARLRKFMMEQNRKVRMDDLVGSSSFQRPLQLTPVPVATGPAAAAAWETGTSRTTLPLFPVRTAGTAE
ITRPEEGKATLTIFYQGQVSTFHHFPADRAKVLMQMASSVTGNTPEKGVAVATAVPKKAQTSDDQPSPAGAGMPPIARKL
TLQNFLRKRKNRIAGTDDADRNEDASPWKKRDSAAGAGGTPAGDVPDDASWLALRL

ZmJAZ5 (TIFY9)

B73 RefGen_v3:
GRMZM2G145412 (zim18 - ZIM-transcription factor 18)
Chr1: 244885427..244886628

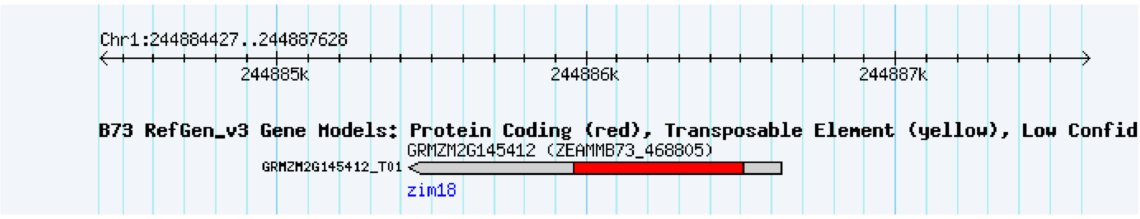

>GRMZM2G145412 Genomic DNA
AAAGCCCAAGCCAAACCAAACAATTTCACCTCACGCACGAAACCGGGAAGCAAGCAAGGTCCTCCCAACTCCCAGCAACC
CAAGGTAGCGCGCGGCAGCAGCAAGCAGCTCGACGGCGACGATGGCGGCAACAGCGGTTACTACTCCAGTAGGCACGGGC
GGCGCTGCGGCAGCGAGAAGCCGGTTCGCGGCGGCGTGCGGGGCGCTCAGCCAGTACGTCAAGGCGGCCGAATCCGAGAG
GATGCACGCGAGGCCGCCCGCGCCTCTGCGGCCGCTCCCGCTCATGCCGGGCGCCGACGTCGACGCCAGCCCTGCCGACC
TAGACCTGCCTCAGGCGGCGCCGGCGCCGGCGCCGGCGCAGTTGACCATCGTGTACGGAGGGCGTGTGCTGGTGCTCGCT
GACGTCCCCGCCGACAAGGCGGCGGGCCTGCTTCGCCTCGCCGCCGGCGCAGCAGCGGAACGTGCAGAAACAGAGGCGAT
CGGTGCCAAGCGCCGCGACCATGTCTCCGGCTCTGCGTCTGCGGGCGCTGACCTGCCAGTTGCGAGGAAGGCGTCGCTGC
AGCGGTTCATGGAGAAGCGCAAGGCTCGGGTCGCCGCCGTCCGCACGGAGCCGTACCGACGGCCGGACGTCAGCGACTCC
TGTCCTGACAACCTCAAGCTTGCGCTCTGATTCCTGTTCCGTGTCCCTACGCACTTTCTATAATTTGTAGGCGAGGTAT
AGGATTATAGGAAGAGCAGCGAAACATGGGTGTACCAAGTGAAATCCAGATCTGAACAAAGTTTTCTGAAAAACACAGTT
GCACGTGCTTTCTACGGCATTATTTGTAAATCTCCAGCTTTAACCACAACACAACTGTTTCTCAATAAAACAGCTTTTT
GTAGCTTCAACTATAGGTGTACATTCGGGCCGTCCGGCCTGGCCCGGCCCAAGCCCGAAAAGGCCCGTATTGTTTGAATT
TCGGGCCGGCCCGGCCCGTTTGAATTTCGGGCCATGCCGGGCCGACCCATGGGCCTAGCCCTCGGCCCACGGCCCGGCCC
GTAATTGATTAAACGTGCCGGGCTCATTTCGGGCGGCCCGAAATTATAAAAGCCCGAAATTCATTTTTTGGCCCGAAATT
CACATTAGGGCCCGAAATTCAATTTTTGGCCCGAAATTCAATTTTTGTCCCAAAATTCACATTAGAGCCCTAAATTCAAA
AA
>GRMZM2G145412_T01 cDNA
AAAGCCCAAGCCAAACCAAACAATTTCACCTCACGCACGAAACCGGGAAGCAAGCAAGGTCCTCCCAACTCCCAGCAACC
CAAGGTAGCGCGCGGCAGCAGCAAGCAGCTCGACGGCGACGATGGCGGCAACAGCGGTTACTACTCCAGTAGGCACGGGC
GGCGCTGCGGCAGCGAGAAGCCGGTTCGCGGCGGCGTGCGGGGCGCTCAGCCAGTACGTCAAGGCGGCCGAATCCGAGAG
GATGCACGCGAGGCCGCCCGCGCCTCTGCGGCCGCTCCCGCTCATGCCGGGCGCCGACGTCGACGCCAGCCCTGCCGACC
TAGACCTGCCTCAGGCGGCGCCGGCGCCGGCGCCGGCGCAGTTGACCATCGTGTACGGAGGGCGTGTGCTGGTGCTCGCT
GACGTCCCCGCCGACAAGGCGGCGGGCCTGCTTCGCCTCGCCGCCGGCGCAGCAGCGGAACGTGCAGAAACAGAGGCGAT
CGGTGCCAAGCGCCGCGACCATGTCTCCGGCTCTGCGTCTGCGGGCGCTGACCTGCCAGTTGCGAGGAAGGCGTCGCTGC
AGCGGTTCATGGAGAAGCGCAAGGCTCGGGTCGCCGCCGTCCGCACGGAGCCGTACCGACGGCCGGACGTCAGCGACTCC
TGTCCTGACAACCTCAAGCTTGCGCTCTGATTCCTGTTCCGTGTCCCTACGCACTTTCTATAATTTGTAGGCGAGGTAT
AGGATTATAGGAAGAGCAGCGAAACATGGGTGTACCAAGTGAAATCCAGATCTGAACAAAGTTTTCTGAAAAACACAGTT
GCACGTGCTTTCTACGGCATTATTTGTAAATCTCCAGCTTTAACCACAACACAACTGTTTCTCAATAAAACAGCTTTTT
GTAGCTTCAACTATAGGTGTACATTCGGGCCGTCCGGCCTGGCCCGGCCCAAGCCCGAAAAGGCCCGTATTGTTTGAATT
TCGGGCCGGCCCGGCCCGTTTGAATTTCGGGCCATGCCGGGCCGACCCATGGGCCTAGCCCTCGGCCCACGGCCCGGCCC
GTAATTGATTAAACGTGCCGGGCTCATTTCGGGCGGCCCGAAATTATAAAAGCCCGAAATTCATTTTTTGGCCCGAAATT
CACATTAGGGCCCGAAATTCAATTTTTGGCCCGAAATTCAATTTTTGTCCCAAAATTCACATTAGAGCCCTAAATTCAAA
AA
>GRMZM2G145412_P01 peptide
MAATAVTTPVGTGGAAAARSRFAAACGALSQYVKAAESERMHARPPAPLRPLPLMPGADVDASPADLDLPQAAPAPAPAQ
LTIVYGGRVLVLADVPADKAAGLLRLAAGAAAERAETEAIGAKRRDHVSGSASAGADLPVARKASLQRFMEKRKARVAAV
RTEPYRRPDVSDSCPDNLKLAL
AGPv4:
Zm00001d033050 (zim18 - ZIM-transcription factor 18)
Chr1: 248529926..248530474

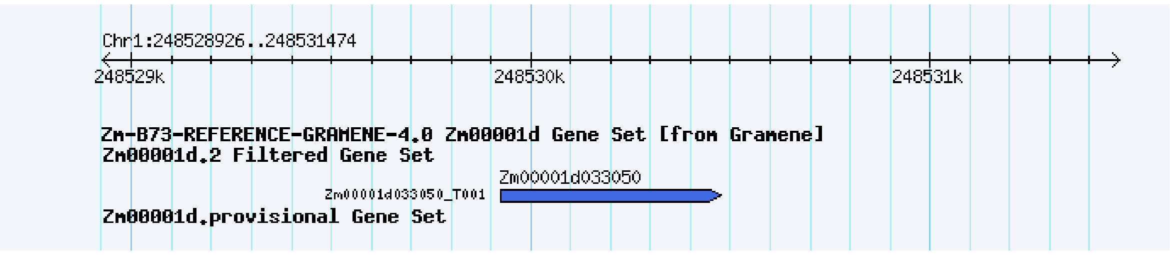

>Zm00001d033050 Genomic DNA
ATGGCGGCAACAGCGGTTACTACTCCAGTAGGCACGGGCGGCGCTGCGGCAGCGAGAAGCCGGTTCGCGGCGGCGTGCGG
GGCGCTCAGCCAGTACGTCAAGGCGGCCGAATCCGAGAGGATGCACGCGAGGCCGCCCGCGCCTCTGCGGCCGCTCCCGC
TCATGCCGGGCGCCGACGTCGACGCCAGCCCTGCCGACCTAGACCTGCCTCAGGCGGCGCCGGCGCCGGCGCCGGCGCAG
TTGACCATCGTGTACGGAGGGCGTGTGCTGGTGCTCGCTGACGTCCCCGCCGACAAGGCGGCGGGCCTGCTTCGCCTCGC
CGCCGGCGCAGCAGCGGAACGTGCAGAAACAGAGGCGATCGGTGCCAAGCGCCGCGACCATGTCTCCGGCTCTGCGTCTG
CGGGCGCTGACCTGCCAGTTGCGAGGAAGGCGTCGCTGCAGCGGTTCATGGAGAAGCGCAAGGCTCGGGTCGCCGCCGTC
CGCACGGAGCCGTACCGACGGCCGGACGTCAGCGACTCCTGTCCTGACAACCTCAAGCTTGCGCTCTGA
>Zm00001d033050_T001 cDNA
ATGGCGGCAACAGCGGTTACTACTCCAGTAGGCACGGGCGGCGCTGCGGCAGCGAGAAGCCGGTTCGCGGCGGCGTGCGG
GGCGCTCAGCCAGTACGTCAAGGCGGCCGAATCCGAGAGGATGCACGCGAGGCCGCCCGCGCCTCTGCGGCCGCTCCCGC
TCATGCCGGGCGCCGACGTCGACGCCAGCCCTGCCGACCTAGACCTGCCTCAGGCGGCGCCGGCGCCGGCGCCGGCGCAG
TTGACCATCGTGTACGGAGGGCGTGTGCTGGTGCTCGCTGACGTCCCCGCCGACAAGGCGGCGGGCCTGCTTCGCCTCGC
CGCCGGCGCAGCAGCGGAACGTGCAGAAACAGAGGCGATCGGTGCCAAGCGCCGCGACCATGTCTCCGGCTCTGCGTCTG
CGGGCGCTGACCTGCCAGTTGCGAGGAAGGCGTCGCTGCAGCGGTTCATGGAGAAGCGCAAGGCTCGGGTCGCCGCCGTC
CGCACGGAGCCGTACCGACGGCCGGACGTCAGCGACTCCTGTCCTGACAACCTCAAGCTTGCGCTCTGA
>Zm00001d033050_P001 peptide
MAATAVTTPVGTGGAAAARSRFAAACGALSQYVKAAESERMHARPPAPLRPLPLMPGADVDASPADLDLPQAAPAPAPAQ
LTIVYGGRVLVLADVPADKAAGLLRLAAGAAAERAETEAIGAKRRDHVSGSASAGADLPVARKASLQRFMEKRKARVAAV
RTEPYRRPDVSDSCPDNLKLAL

ZmJAZ6 (TIFY10)

B73 RefGen_v3:
Chr1: 244892947..244893692

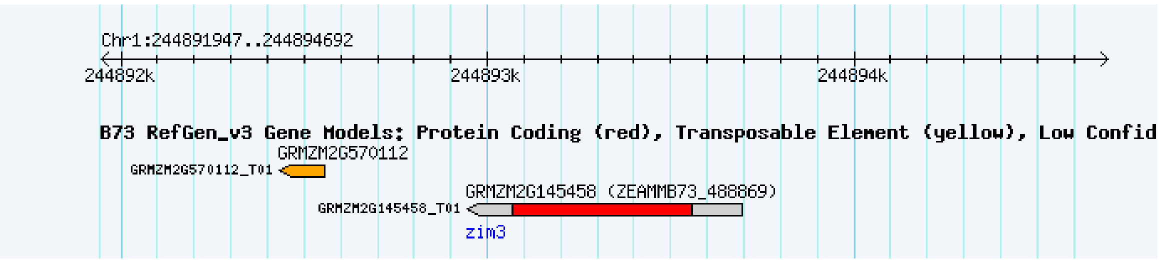

>GRMZM2G145458 Genomic DNA
GCGTCGCCGCCCCCGCGCCCCCGCCTATATAAAAAATCTCGTCCCGAGTCCATTCATCACGGGGAACTCCTGCAAAACTA
GACACTTTTACACAGCGTGGCAGGGCAGCGGCAAGCAGATCGCGAGCTAAAGACAATGGCAGCAGCGGGGAGCGTCCAGG
GCCACGGCGCGCGGTTCGCGGCGGCGTGCGGCGTGCTCAGCCGGTACGTAAAAGCAGCAGCGGTGGCGACTACGACGACG
GTGGAGCTGCGGCCGGCGGGCACGGTAGGGGTACTCCCTCTGATGCCTGGTGCGGACCTGTCCACGCAAGAGGAGCGCGA
GGCGGGGGCGGGGCCGGGGCCGTCGCCGTCGCCGAGCGCGCAGCTGACCATCTCGTACGGCGGGCGGGTGGTGGTGCTGG
ACGACGTCCCGGCGGACAAGGCCGCCGAGGTGGTCCGGCTCGCGGCCGCGCAAGGCGCACCGCGGGCGCTGCGAGCGCCG

CCGACCAAGGCGGATGATCTGCCCATGGCGAGGAAGGTGTCGCTGCAGCAGTTCATGGAGAGGCGCAAGGGCCGGGTCGC
CACGCGCGGTTCGCCCTACCGCCGTCCGGCGTCGTTGCCGGACCATCTCACGCTCACGCTCTGATACAGCTTGTTACTAC
TATTACTAGCTAGGTGTGTGTAGCTGTAGCAGCATAAGAACAGACGATTGTACATGGAAAAAAAAGAAAACAGAAATAAC
TCTCCGATCGAGTTCACGAAGCAAAA
>GRMZM2G145458_T01 cDNA
GCGTCGCCGCCCCCGCGCCCCCGCCTATATAAAAAATCTCGTCCCGAGTCCATTCATCACGGGGAACTCCTGCAAAACTA
GACACTTTTACACAGCGTGGCAGGGCAGCGGCAAGCAGATCGCGAGCTAAAGACAATGGCAGCAGCGGGGAGCGTCCAGG
GCCACGGCGCGCGGTTCGCGGCGGCGTGCGGCGTGCTCAGCCGGTACGTAAAAGCAGCAGCGGTGGCGACTACGACGACG
GTGGAGCTGCGGCCGGCGGGCACGGTAGGGGTACTCCCTCTGATGCCTGGTGCGGACCTGTCCACGCAAGAGGAGCGCGA
GGCGGGGGCGGGGCCGGGGCCGTCGCCGTCGCCGAGCGCGCAGCTGACCATCTCGTACGGCGGGCGGGTGGTGGTGCTGG
ACGACGTCCCGGCGGACAAGGCCGCCGAGGTGGTCCGGCTCGCGGCCGCGCAAGGCGCACCGCGGGCGCTGCGAGCGCCG
CCGACCAAGGCGGATGATCTGCCCATGGCGAGGAAGGTGTCGCTGCAGCAGTTCATGGAGAGGCGCAAGGGCCGGGTCGC
CACGCGCGGTTCGCCCTACCGCCGTCCGGCGTCGTTGCCGGACCATCTCACGCTCACGCTCTGATACAGCTTGTTACTAC
TATTACTAGCTAGGTGTGTGTAGCTGTAGCAGCATAAGAACAGACGATTGTACATGGAAAAAAAAGAAAACAGAAATAAC
TCTCCGATCGAGTTCACGAAGCAAAA
>GRMZM2G145458_P01 peptide
MAAAGSVQGHGARFAAACGVLSRYVKAAAVATTTTVELRPAGTVGVLPLMPGADLSTQEEREAGAGPGPSPSPSAQLTIS
YGGRVVVLDDVPADKAAEVVRLAAAQGAPRALRAPPTKADDLPMARKVSLQQFMERRKGRVATRGSPYRRPASLPDHLTL
TL
AGPv4:
Zm00001d033049 (zim3 - ZIM-transcription factor 3)
Chr1: 248522876..248523364

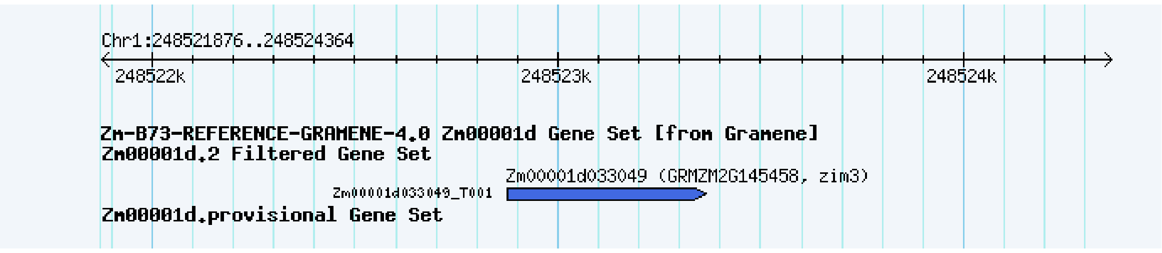

>Zm00001d033049 Genomic DNA
ATGGCAGCAGCGGGGAGCGTCCAGGGCCACGGCGCGCGGTTCGCGGCGGCGTGCGGCGTGCTCAGCCGGTACGTAAAAGC
AGCAGCGGTGGCGACTACGACGACGGTGGAGCTGCGGCCGGCGGGCACGGTAGGGGTACTCCCTCTGATGCCTGGTGCGG
ACCTGTCCACGCAAGAGGAGCGCGAGGCGGGGGCGGGGCCGGGGCCGTCGCCGTCGCCGAGCGCGCAGCTGACCATCTCG
TACGGCGGGCGGGTGGTGGTGCTGGACGACGTCCCGGCGGACAAGGCCGCCGAGGTGGTCCGGCTCGCGGCCGCGCAAGG
CGCACCGCGGGCGCTGCGAGCGCCGCCGACCAAGGCGGATGATCTGCCCATGGCGAGGAAGGTGTCGCTGCAGCAGTTCA
TGGAGAGGCGCAAGGGCCGGGTCGCCACGCGCGGTTCGCCCTACCGCCGTCCGGCGTCGTTGCCGGACCATCTCACGCTC
ACGCTCTGA
>Zm00001d033049_T001 cDNA
ATGGCAGCAGCGGGGAGCGTCCAGGGCCACGGCGCGCGGTTCGCGGCGGCGTGCGGCGTGCTCAGCCGGTACGTAAAAGC
AGCAGCGGTGGCGACTACGACGACGGTGGAGCTGCGGCCGGCGGGCACGGTAGGGGTACTCCCTCTGATGCCTGGTGCGG
ACCTGTCCACGCAAGAGGAGCGCGAGGCGGGGGCGGGGCCGGGGCCGTCGCCGTCGCCGAGCGCGCAGCTGACCATCTCG
TACGGCGGGCGGGTGGTGGTGCTGGACGACGTCCCGGCGGACAAGGCCGCCGAGGTGGTCCGGCTCGCGGCCGCGCAAGG
CGCACCGCGGGCGCTGCGAGCGCCGCCGACCAAGGCGGATGATCTGCCCATGGCGAGGAAGGTGTCGCTGCAGCAGTTCA
TGGAGAGGCGCAAGGGCCGGGTCGCCACGCGCGGTTCGCCCTACCGCCGTCCGGCGTCGTTGCCGGACCATCTCACGCTC
ACGCTCTGA

>Zm00001d033049_P001 peptide
MAAAGSVQGHGARFAAACGVLSRYVKAAAVATTTTVELRPAGTVGVLPLMPGADLSTQEEREAGAGPGPSPSPSAQLTIS
YGGRVVVLDDVPADKAAEVVRLAAAQGAPRALRAPPTKADDLPMARKVSLQQFMERRKGRVATRGSPYRRPASLPDHLTL
TL

ZmJAZ7

B73 RefGen_v3:
GRMZM2G382794 (zim19 - ZIM-transcription factor 19)
Chr1: 290452059..290455157

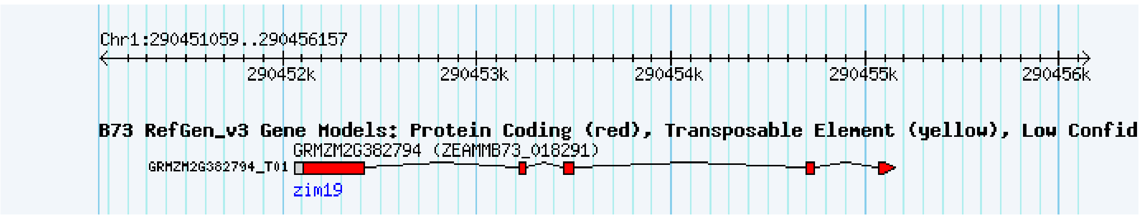

>GRMZM2G382794 Genomic DNA
CCTCCACCTCCGGGCGCCCCGGCCGCCACGACCCTGAGCCTGATGCCTGGCGCGGACGTCGGCGCCGACGAGCAGCAGGA
GCCGGCCGCCGCGAGGGCCGAGGAGATGCCCGAGCCTGCCCCCGCCCCCGCCGCGCCGCCGCCGCTCACCATCTTCTACG
GCGGCACGGTGGTGGTGTTCGAGGACTTCCCGGCGGAGAAGACGGCCGAGGTCATGCGCCTCGCGGCCGGCGACGACCTG
CCCATCGCCCGGAAGGCGTCGCTGCCGTGGTTCCTGGCCAAGCGCAAGGACCGCCTCGTCGAGCGCGCGCCCTACGCCCG
CCCGTCGTCCCCCGCGAAGGAGCCGGAGAAGAAGACGGTCAAGCCAGCCTCGGCCTCAGCCTCATGCTCGACAAAATGAC
TGTGTGACTGCTGTTGCCTGAACCGAACTGGCCACCTCTACGCTCATTCCTCTCACGTTTCTGAAGGGTGCAGAGTAGCC
GGTCGACTAATCGAGCTCCCTACGCCTCCTGCACATGGCCTAAAACTCCCTGCTCTTGCCTGCACGCCTCGCTCTGCTTA
GCATCGGAGCGCACGGACGCTGCAGCCATGTTTATTCCTTGCAGCGAAACGCATGGGCTTGAAGGAGTTCAAAATGGGGT
ATGAATCATCTTAGTTTGACTTTTGTTTTAAATCCATTGATTATTGACCCTTATGTCTAAACTGCCCTAAGATACTTATT
TCTGGCTTCATAAATCACAAAACAGAGCCAAAGCTATAGTCAATGCAGAAAGTAAACCGAGCTAACAAATAAAAAACAAT
GCTACGACACAAAAGATCAACAAGCTAGAGCCTAAATTAACATCAGTAATTCAAATCCACTTAAGTAATATAGTGTGTGA
TGTAAGCCAAAAAATCAGATGCAACCATTCTAGCCAAAGACTCTTTAGCCCATAAGTAATTCATGTTTTTGTAGATGCTC
GACATGGTTCTTGATGATCTATAGAATGAGCACCCTAATCTCTGTTCTCCTCGGTTCCCGCGTGTGTGGAAAAGCAGTGG
TGAGGTTCAATAACTGGTTACACATTGTTTTTGATTGCTCAGTGCAACCATGATGTTTGATGAAATGTTCAACTCTGCT
ATTGCACATTTGCACGCTGCATTGCAGTGTTGAAATGTAGTTTACTTTTTGTTTGAGAATTCACATGACAGGTCAGTTTG
TACATTTTTAAGAGTCCAAATTAGGTTCGTTCTGCATAATACATGTGGTGTTTAATACCATTGTATTCCGAAAATGATAT
GAGCTTACCTTGCTGTATCTCCATGTATATCATGAAAATTTATTTAAAATTGGACGCTTGATTGATTGAAAGACCTAATG
AATTGCTATTTGTCTTTGTTTTCAAAGGGATGGCCTATGACCCTTTAAGAACTCATCTAGAGCGAAGAGCAGCTGAGCAG
GTAATTACTCTGTCTCGATTTGTTTGAACGCATAATATAATCTATACATCAGAACCTAGCAAGCTTAAGGAAAACAGTC
AAATTCGCGGTAATTACATAGATAAGTCCATACTTTTGTGAAACCTGCAGCAAAAATAGAATAAAAAATATTAGATCTGT
CATGTGATGTTCTAACAATTACACATCTTTCCCTTATTAAGTGCCCATTTTTCCACAAGATGCTCACCTGCATGGCTACA
GGAAAATCATCCTAAAAATCTAACAATAATGTTGATGCCATTGACGAGAAATTTTCACTAAGGTGTGGTCACAGTCATGG
CTATCATGTAGGTGTTTAGCTGCTGTTGCATTAAGTCTAGATTTTAAGAATCCTAATGTCTAATCATTTCGATAGAAAAG
GAACCACAAGTCTTAAAACACCTTACTATGTTGCTTAGTTTTTATACCGTAATATATGTGTCGATAGGAGTTAGGATATA
ATTTTGTGAGATTTCTGATAATCTTTTGAGTTGCCAATATGCAATTTGATTGCTTTGTTTACAATAAGTGGTATATAAGT
TATGCTATGTTTCTTCCATGAATCCTGTGCCATCGATTATACTAGCAAAGAACTTTCTAACAACTATAGATATTACTTCA
ACTATTATTGGTTTCATTTCATACAATTTTGGCAATACCAGCAATTAATTACTGAAGTTATTTATGGTTCCATGGCACTA
AATAAGTGTCATAGAATTTTAGTTTGCATAACTTCAGTTGTTTAAAATTGGGAACCCATTGACCATGCAGATTTTTTGTA

AACCAACTTGACTCCCCTGCATTGCTATCCCACTAAATAATTATGGTACTCGAAAAGTTAATGTCAAATCTAGGTACTAA
AATCACACTATCTCTTAATCTCCTAACAACTTTAAGAATAAAGGACTCCAAAACTCCATCTTTTACCTGACAGAGGAAGT
AGGGAAGGAGAAAAGGCTGGACAAAATGGTGATCCGGGTTGTTTTTACTTTACCATCAACGTTTATCAATTACTCCATT
CCAAATTATAAGACGCCTTGAAATAGAATGCTTCAAGTTAAAGTTAGCTATTGTTTGCATGCTTCTTTATTTATATTTTA
TTGTCCAATTTCCCTGACTATTGGGTTAACTTCTGTCTCAATCTGTTAATTTTTTTCTTCTGTTTTGTGACACAGTTGGA
TATATTCAAGGAAATGGTATCTTTGCGTGTATTTGAGAGGTACATGATAAGTGTAATGGTTTCACGTTGTCTGCAGATTT
TGTTTGGATATTCACTAGATGAGTAATTTCACCATGGTTTAATTCTAACAGTTGCTCTTATTACATCCAAGACCGCCATG
TTCTACTGTAATATTGTCTTGTGTCCTATCAAAGCAAAGAAATGTGTTGACATGCTTTGGTTGTTCTGTTGAAATAATAT
TCCCAGTATCATATATCCCCCTTCTATTTCTTCTGAGCAAAATGCTTACCAGTTTGAACTTAGTTTGGTGCTGATGTTCT
TGTGATGACTGGACTACTCATCCGTTTTTTAGTTAATTCTGCTTTTTGCAGGATTTTTAGTGAACTCCTATACTGTGAGC
ACTGGACTACACATTATGTTTTTTTGCAGGATTACAAGTTCATTCTGTTTTTCAGGTAA
>GRMZM2G382794_T01 cDNA
CCTCCACCTCCGGGCGCCCCGGCCGCCACGACCCTGAGCCTGATGCCTGGCGCGGACGTCGGCGCCGACGAGCAGCAGGA
GCCGGCCGCCGCGAGGGCCGAGGAGATGCCCGAGCCTGCCCCCGCCCCCGCCGCGCCGCCGCCGCTCACCATCTTCTACG
GCGGCACGGTGGTGGTGTTCGAGGACTTCCCGGCGGAGAAGACGGCCGAGGTCATGCGCCTCGCGGCCGGCGACGACCTG
CCCATCGCCCGGAAGGCGTCGCTGCCGTGGTTCCTGGCCAAGCGCAAGGACCGCCTCGTCGAGCGCGCGCCCTACGCCCG
CCCGTCGTCCCCCGCGAAGGAGCCGGAGAAGAAGACGTTTACTTTTTGTTTGAGAATTCACATGACAGGGATGGCCTATG
ACCCTTTAAGAACTCATCTAGAGCGAAGAGCAGCTGAGCAGTTGGATATATTCAAGGAAATGGTATCTTTGCGTGTATTT
GAGAGGATTTTTAGTGAACTCCTATACTGTGAGCACTGGACTACACATTATGTTTTTTTGCAGGATTACAAGTTCATTCT
GTTTTTCAGGTAA
>GRMZM2G382794_P01 peptide
MPGADVGADEQQEPAAARAEEMPEPAPAPAAPPPLTIFYGGTVVVFEDFPAEKTAEVMRLAAGDDLPIARKASLPWFLAK
RKDRLVERAPYARPSSPAKEPEKKTFTFCLRIHMTGMAYDPLRTHLERRAAEQLDIFKEMVSLRVFERIFSELLYCEHWT
THYVFLQDYKFILFFR
AGPv4:
Zm00001d034536 (zim19 - ZIM-transcription factor 19)
Chr1: 295853517..295853873

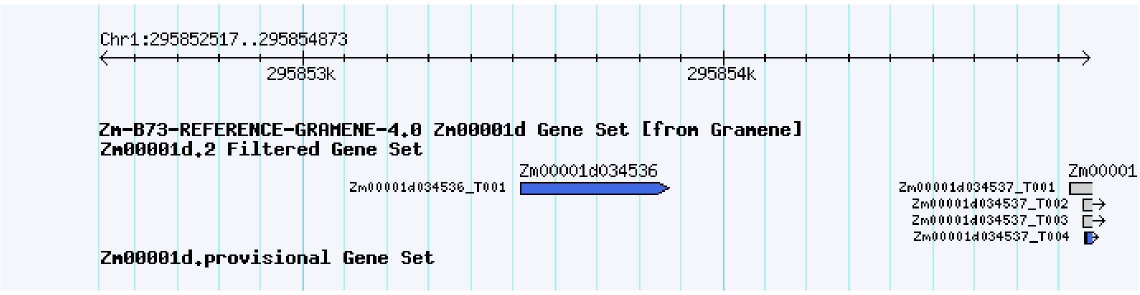

>Zm00001d034536 Genomic DNA
ATGCCTGGCGCGGACGTCGGCGCCGACGAGCAGCAGGAGCCGGCCGCCGCGAGGGCCGAGGAGATGCCCGAGCCTGCCCC
CGCCCCCGCCGCGCCGCCGCCGCTCACCATCTTCTACGGCGGCACGGTGGTGGTGTTCGAGGACTTCCCGGCGGAGAAGA
CGGCCGAGGTCATGCGCCTCGCGGCCGGCGACGACCTGCCCATCGCCCGGAAGGCGTCGCTGCCGTGGTTCCTGGCCAAG
CGCAAGGACCGCCTCGTCGAGCGCGCGCCCTACGCCCGCCCGTCGTCCCCCGCGAAGGAGCCGGAGAAGAAGACGGTCAA
GCCAGCCTCGGCCTCAGCCTCATGCTCGACAAAATGA
>Zm00001d034536_T001 cDNA
ATGCCTGGCGCGGACGTCGGCGCCGACGAGCAGCAGGAGCCGGCCGCCGCGAGGGCCGAGGAGATGCCCGAGCCTGCCCC
CGCCCCCGCCGCGCCGCCGCCGCTCACCATCTTCTACGGCGGCACGGTGGTGGTGTTCGAGGACTTCCCGGCGGAGAAGA
CGGCCGAGGTCATGCGCCTCGCGGCCGGCGACGACCTGCCCATCGCCCGGAAGGCGTCGCTGCCGTGGTTCCTGGCCAAG

CGCAAGGACCGCCTCGTCGAGCGCGCGCCCTACGCCCGCCCGTCGTCCCCCGCGAAGGAGCCGGAGAAGAAGACGGTCAA
GCCAGCCTCGGCCTCAGCCTCATGCTCGACAAAATGA
>Zm00001d034536_P001 peptide
MPGADVGADEQQEPAAARAEEMPEPAPAPAAPPPLTIFYGGTVVVFEDFPAEKTAEVMRLAAGDDLPIARKASLPWFLAK
RKDRLVERAPYARPSSPAKEPEKKTVKPASASASCSTK

ZmJAZ8 (TIFY12)

B73 RefGen_v3:
GRMZM2G086920 (zim32 - ZIM-transcription factor 32)
Chr2: 4702643..4706609

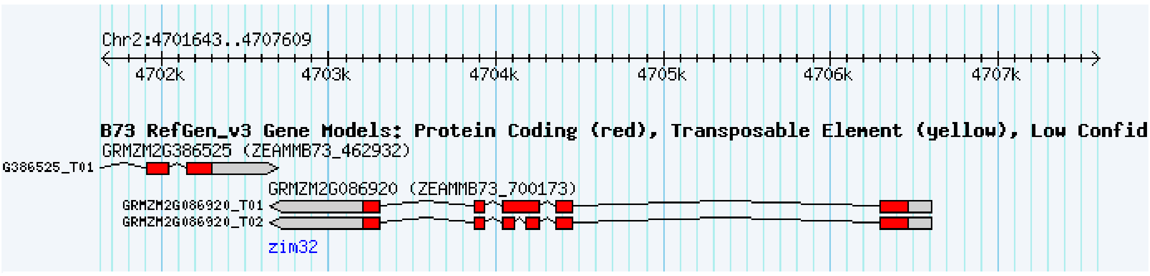

>GRMZM2G086920 Genomic DNA
GCGGTACTACACAGTTTCATCACATAGTTGCTGGCGGCTGGCCGGCCTCTTTCTCTTTCTCGTTTCTGCCCCCTCTGCTT
GCCCCCCTTTCATCTCAGGCGTCGAAGAGAGAGAGAGAGAGAGAGAGGAGTTGCAAGTTGGTGAGATGGATCTGTTGGAG
CGGAATATTAAGACGGAGACGGAGGAGACGCGCAAGGAACAGGAGCGTAAGGAGGAGGAGGCGGAGGCGGAGGCGGAGGC
GGGGGAGAGGAAGACCCAGGAGCCGCCGCAGCAAGGCCAGGGCCTCAGCCTCTCACTCGCCAACGGCAGTGGCAGGTACC
TAGCCTCACCTCTCCGAGACTCCCCGCACCGCACCGCCGAGTCCAGCGAGGGCTCTGCTTCCCTGGAGTGGTTCGCGGCT
GTCTAGTCCCTCGATTGGTGGCCTGGTGGGGGAAATTTGAGTTCCTTCTCTCGTTCGTCTCCCCCGCGCTTTCCCCTCTT
TCCTGTTCTCGGCGGTTGACGCGCTTTAGCACTTGCCACCCCAGCTTTTCGCGTTAACTGTGGAAGTGCCACGTGGGATG
GGCCTTGGGCTGTGGCCGTACCTTTCGCTGGGTCAAATCTGATTGCGGGCTTGCGGCCGCTCCTCCGGGCGAGGATACGG
AGCCGGCGTGGCGGATCCGGGAAGGCGTGTTCGCCCTCTCCTCGGCTCCTCGCTCGCAGGCGAAATCCCCTTCGGTTCTG
TCTATCGCTCCTGCTTTCCACTCTCTCCTGCTGTCGCGAAGCTGTAACCTGATTTCGGTGTAGTCTTATGTTAGCGTCAA
ATGTTCTTATCCATATTCCTTCTGCTGGGAAGGGGTTCATCAGTTGCCTGAAACCTTCGTTTCCGGTCCTCACTCATCTT
GCTGTCATTAGCTGTCTTGCGTTTCTATCTACCCTATTATATGGATATTGTCATACTGATATATCATTATGGTGAACGCG
AAACTGAACTCGGTGACTGGCTTAGCGCCAAATGTTTGCTTCCTTTTGTTTTATACTAGGGCACTCAGTTCAGTTTGTCT
ACTTTGGGTCCTTAATCAAATCTAAATTAAATGGGAGAGACGATTTGTTCATTTGTTTCGGCCAAAATGGTTGCCCTTTT
TTCATGGTTGTTGGGCTTTTGTAAAGAACTTCAGCAGATGGCGTGCAGATCCAAGCAACCCTTTCCCATCTTTTGTTGTA
TACAATTGTTTAAAGCTTTATTTACGAAAGGTTGTAAACGGAATTTCAGAATCAGATTAACGACGGATAACTGCTACTGT
TTGCAACGCAAACCTTTAGTTGGAGAAATGTGAAACACGCGTAGATCTGATGAAAGTTGAGTTTAACAGGCAATAATTCC
ATTCTGCTGCCAAGGACCTTATGCTATAGTGCTAGACTGTGTGATAGGGGACCAAGGACGCAGAGTCTGCCAAACCCTGC
AAACCCATTGGGGACATTGAACTGGTCCTTAAAATTTCCTCTGATGTTAAGCTACTTTTTCCTTGAACCGGACCATGTTT
TGCAGATTTGTCTGTTGTGCTATAATTAGTTGTTCAGTATGCTTCTTCAGAAGCATTTATTTGTTTTGCTTCGACAGTTC
TAGAACATTTAGTGTATGGTTCTGCTATTTTCAGTTCCATTTTATTTCCTGGTTAGTTGTTATCAGCCAGGCTTTGAACA
ATATGGACATCTGCACTAATCAGATTGGACAGACCCAGTTGATAGCGGCTCCCACTCATAACCGAGGCAGCCTTACCCTT
GTTTTTTAGAGAGGCTGCATTGAACACAAGACCTTTAAGAACTTTGTGACTGACAAAATGCTTTGCGATATCTGTACTGC
TAAGTCCCTATAAATGGTTTGAAAGCCACTGTCCAGAAGGCAATCCATTTGTGAATGGCCAAGACGCCAAGTAGATGTAT

CCTTCTATTAGAAATCTATCTCCATTTTCCGTCCATTAGGAAGTAGGATGACTGTTTTCCTGTACCCCGCACATGCATTT
TATCTCATGCTAGTGCTGAACCTATCACATGGCATTATTTCATGATTTAATCTGAACTACTAGTAAGGATGACATGGATC
TGCCTACTTCAGAGATGCCATTTTATCTGCCTTTGCATGTTCTTAATTTTTCTGCATTCTTTACTACAAGGTCTGGAATG
TTGCCGATGTCGAACGTGTCCGCTAATCCTTCACAGCTTACAATTTTCTATGGCGGATCAGTATGTGTGTATGACTCAGT
GCCACCGGAAAAGGTGATATTGTCTTCTTCATTTACAGATATCTTGTCCTTCTGTACTGTCTCTTAAAGACACATATGCA
TGCTAACTCACTAATCTGGAATTGACAGGCTCAAGCAATCATGCTTATAGCTGCAGCTGCAGCGGCAGCGGCAGCTACCA
AAAGCAGTGCTGCCCCTACTGTTAAGCCTCCAATGGTGCCTGCAGCCACTGTTGCCCCAGCAGCTGTCGTTTCTCCTGTG
CTTACACGGTCTCCATCACTGCAGAGCACTTCTGTAGCAACTGGGCAACCTCAGGTTGTTGCTGAACCTAGCTCGATATG
CAAACTTCAGGCTGGTAAGACCACAGCATCTTTTGGGTAAAAAACCAGAAATGGAATACCTTTTTCAGTGTTGACATTTT
TCTAACATATCTTTAAACTACCTTAATCCGCTGGCCAGATCTCCCCATTGCCAGGAGGCACTCTCTTCAGCGTTTCCTTG
AGAAACGCCGTGACAGGTTAGCATGGTAGATGATAGCAACAGCGCTATGATGTTCTGAATCTCTTCTTAGGCTCCTTCTA
CATGTTGAAAACTTTAGTTATAAACTGGTATATGTGGTACAACAGTATTCAACAGCATGCTTTGTTCCGTGTTCTATTTT
TATCCTCTAGGTAGGGTAAAAAATTGAAATAATTTGCTGTTATAATTGTATTTAAATGATTGCACTTTGTGCTCGATGAC
TGCATAAAGTAAACGAAATGTGACTAATTCATTTAAAACATTCCTCAAAAATCCTAAAAATTTGTTTACAGCAGGAATGA
GTAGAGAGCCTGCTTACATAATAGAACACATAGCAGCTAACAGCGTTGGATCATGCAACTTTCCTGACTTTTTAAATATC
TGCAAAAACCGAAATTAAGGGTGGTCACCTAAAGGCCCATGTGTCATCTGTAGGTTGCAAACTAGCTGAACACCATTTTG
TTCTTCAGATGTCAGGTTTAGATGGCTATTTTGCCTTATCTTAAAACTGAGTTTAGAATTGCCTTTTGTCATGGGTTTGC
TGAATTCGACTGCTGTTGGCATGAAACAGGGTCGTGAGCAAAGCTCCATACAGCACCGCCAAGTCGTCCGATGGCATGGA
GTCACCGAGGATTGAGGTGACAGCGGAGGGCAAGGCCCAGTAACTTGAAAGGTCCCCGCGGAGTCCCATCGGTGAAGGCT
GACGTTGTGGGAAGGGGCAGGGACGCGCTGTTGTTGCCTTATGTTATGTGGCGGCGGCAGCGTGGAACCGCACTAATAAT
GTGGACCGGAACCTCTGTTACCGTCGTTTGTGCTGTCGCGTGCTAGGTTTATGCTGAAGTGGATTCCTTTACTCCGCACA
CCGAACTGTGTCTGTTGTACATCACGCCGTGACGCCGGTGGTCTCGTCTGTCTGACTTCGTTACATGGATCGTCTAGTAG
TAACGGAATGCAGTACCACGTGTGGACATGTGTTTAGTGTGAAAGATGATGGGGCTTTGCTTGCTTGTTTCCACTACCAT
TTCCCATTTTGGCACTTAACGTTTGTTCTTCTCTTCGCTACCTTGTTCTCAGCTCAAATGGTCCCACGCAGATGCATGTG
TAGGGAGGTTGAAAATGATAGAAATACGCTCAGATCACCTTCGTTCCTTGCTTCTCTTGCCGTTTCTGTTGGACCAAAAA
TAAAATAAAATTTCGATGTCGTCCCACGCAGATGCATGCGGAACCCC

>GRMZM2G086920_T01 cDNA

GCGGTACTACACAGTTTCATCACATAGTTGCTGGCGGCTGGCCGGCCTCTTTCTCTTTCTCGTTTCTGCCCCCTCTGCTT
GCCCCCCTTTCATCTCAGGCGTCGAAGAGAGAGAGAGAGAGAGAGAGGAGTTGCAAGTTGGTGAGATGGATCTGTTGGAG
CGGAATATTAAGACGGAGACGGAGGAGACGCGCAAGGAACAGGAGCGTAAGGAGGAGGAGGCGGAGGCGGAGGCGGAGGC
GGGGGAGAGGAAGACCCAGGAGCCGCCGCAGCAAGGCCAGGGCCTCAGCCTCTCACTCGCCAACGGCAGTGGCAGGTCTG
GAATGTTGCCGATGTCGAACGTGTCCGCTAATCCTTCACAGCTTACAATTTTCTATGGCGGATCAGTATGTGTGTATGAC
TCAGTGCCACCGGAAAAGGCTCAAGCAATCATGCTTATAGCTGCAGCTGCAGCGGCAGCGGCAGCTACCAAAAGCAGTGC
TGCCCCTACTGTTAAGCCTCCAATGGTGCCTGCAGCCACTGTTGCCCCAGCAGCTGTCGTTTCTCCTGTGCTTACACGGT
CTCCATCACTGCAGAGCACTTCTGTAGCAACTGGGCAACCTCAGGTTGTTGCTGAACCTAGCTCGATATGCAAACTTCAG
GCTGATCTCCCCATTGCCAGGAGGCACTCTCTTCAGCGTTTCCTTGAGAAACGCCGTGACAGGGTCGTGAGCAAAGCTCC
ATACAGCACCGCCAAGTCGTCCGATGGCATGGAGTCACCGAGGATTGAGGTGACAGCGGAGGGCAAGGCCCAGTAACTTG
AAAGGTCCCCGCGGAGTCCCATCGGTGAAGGCTGACGTTGTGGGAAGGGGCAGGGACGCGCTGTTGTTGCCTTATGTTAT
GTGGCGGCGGCAGCGTGGAACCGCACTAATAATGTGGACCGGAACCTCTGTTACCGTCGTTTGTGCTGTCGCGTGCTAGG
TTTATGCTGAAGTGGATTCCTTTACTCCGCACACCGAACTGTGTCTGTTGTACATCACGCCGTGACGCCGGTGGTCTCGT
CTGTCTGACTTCGTTACATGGATCGTCTAGTAGTAACGGAATGCAGTACCACGTGTGGACATGTGTTTAGTGTGAAAGAT
GATGGGGCTTTGCTTGCTTGTTTCCACTACCATTTCCCATTTTGGCACTTAACGTTTGTTCTTCTCTTCGCTACCTTGTT
CTCAGCTCAAATGGTCCCACGCAGATGCATGTGTAGGGAGGTTGAAAATGATAGAAATACGCTCAGATCACCTTCGTTCC
TTGCTTCTCTTGCCGTTTCTGTTGGACCAAAAATAAAATAAAATTTCGATGTCGTCCCACGCAGATGCATGCGGAACCCC

>GRMZM2G086920_P01 peptide
MDLLERNIKTETEETRKEQERKEEEAEAEAEAGERKTQEPPQQGQGLSLSLANGSGRSGMLPMSNVSANPSQLTIFYGGS
VCVYDSVPPEKAQAIMLIAAAAAAAAATKSSAAPTVKPPMVPAATVAPAAVVSPVLTRSPSLQSTSVATGQPQVVAEPSS
ICKLQADLPIARRHSLQRFLEKRRDRVVSKAPYSTAKSSDGMESPRIEVTAEGKAQ
>GRMZM2G086920_T02 cDNA
GCGGTACTACACAGTTTCATCACATAGTTGCTGGCGGCTGGCCGGCCTCTTTCTCTTTCTCGTTTCTGCCCCCTCTGCTT
GCCCCCCTTTCATCTCAGGCGTCGAAGAGAGAGAGAGAGAGAGAGAGGAGTTGCAAGTTGGTGAGATGGATCTGTTGGAG
CGGAATATTAAGACGGAGACGGAGGAGACGCGCAAGGAACAGGAGCGTAAGGAGGAGGAGGCGGAGGCGGAGGCGGAGGC
GGGGGAGAGGAAGACCCAGGAGCCGCCGCAGCAAGGCCAGGGCCTCAGCCTCTCACTCGCCAACGGCAGTGGCAGGTCTG
GAATGTTGCCGATGTCGAACGTGTCCGCTAATCCTTCACAGCTTACAATTTTCTATGGCGGATCAGTATGTGTGTATGAC
TCAGTGCCACCGGAAAAGGCTCAAGCAATCATGCTTATAGCTGCAGCTGCAGCGGCAGCGGCAGCTACCAAAAGCAGTGC
TGCCCCTACTGTTAAGCCTCCAATGAGCACTTCTGTAGCAACTGGGCAACCTCAGGTTGTTGCTGAACCTAGCTCGATAT
GCAAACTTCAGGCTGATCTCCCCATTGCCAGGAGGCACTCTCTTCAGCGTTTCCTTGAGAAACGCCGTGACAGGGTCGTG
AGCAAAGCTCCATACAGCACCGCCAAGTCGTCCGATGGCATGGAGTCACCGAGGATTGAGGTGACAGCGGAGGGCAAGGC
CCAGTAACTTGAAAGGTCCCCGCGGAGTCCCATCGGTGAAGGCTGACGTTGTGGGAAGGGGCAGGGACGCGCTGTTGTTG
CCTTATGTTATGTGGCGGCGGCAGCGTGGAACCGCACTAATAATGTGGACCGGAACCTCTGTTACCGTCGTTTGTGCTGT
CGCGTGCTAGGTTTATGCTGAAGTGGATTCCTTTACTCCGCACACCGAACTGTGTCTGTTGTACATCACGCCGTGACGCC
GGTGGTCTCGTCTGTCTGACTTCGTTACATGGATCGTCTAGTAGTAACGGAATGCAGTACCACGTGTGGACATGTGTTTA
GTGTGAAAGATGATGGGGCTTTGCTTGCTTGTTTCCACTACCATTTCCCATTTTGGCACTTAACGTTTGTTCTTCTCTTC
GCTACCTTGTTCTCAGCTCAAATGGTCCCACGCAGATGCATGTGTAGGGAGGTTGAAAATGATAGAAATACGCTCAGATC
ACCTTCGTTCCTTGCTTCTCTTGCCGTTTCTGTTGGACCAAAAATAAAATAAAATTTCGATGTCGTCCCACGCAGATGCA
TGCGGAACCCC
>GRMZM2G086920_P02 peptide
MDLLERNIKTETEETRKEQERKEEEAEAEAEAGERKTQEPPQQGQGLSLSLANGSGRSGMLPMSNVSANPSQLTIFYGGS
VCVYDSVPPEKAQAIMLIAAAAAAAAATKSSAAPTVKPPMSTSVATGQPQVVAEPSSICKLQADLPIARRHSLQRFLEKR
RDRVVSKAPYSTAKSSDGMESPRIEVTAEGKAQ
AGPv4:
Zm00001d002029 (zim32 - ZIM-transcription factor 32)
Chr2: 4666311..4669471

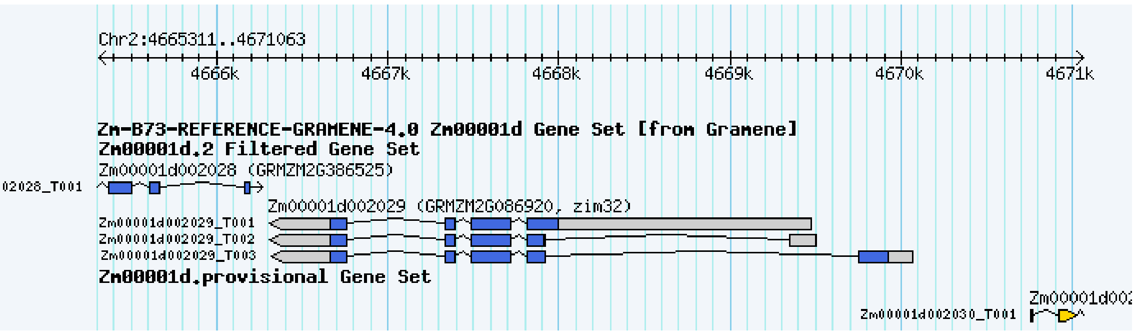

>Zm00001d002029 Genomic DNA
GGTACTACACAGTTTCATCACATAGTTGCTGGCGGCTGGCCGGCCTCTTTCTCTTTCTCGTTTCTGCCCCCTCTGCTTGC
CCCCCTTTCATCTCAGGCGTCGAAGAGAGAGAGAGAGAGAGAGAGGAGTTGCAAGTTGGTGAGATGGATCTGTTGGAGCG
GAATATTAAGACGGAGACGGAGGAGACGCGCAAGGAACAGGAGCGTAAGGAGGAGGAGGCGGAGGCGGAGGCGGAGGCGG
GGGAGAGGAAGACCCAGGAGCCGCCGCAGCAAGGCCAGGGCCTCAGCCTCTCACTCGCCAACGGCAGTGGCAGGTACCTA
GCCTCACCTCTCCGAGACTCCCCGCACCGCACCGCCGAGTCCAGCGAGGGCTCTGCTTCCCTGGAGTGGTTCGCGGCTGT

CTAGTCCCTCGATTGGTGGCCTGGTGGGGGAAATTTGAGTTCCTTCTCTCGTTCGTCTCCCCCGCGCTTTCCCCTCTTTC
CTGTTCTCGGCGGTTGACGCGCTTTAGCACTTGCCACCCCAGCTTTTCGCGTTAACTGTGGAAGTGCCACGTGGGATGGG
CCTTGGGCTGTGGCCGTACCTTTCGCTGGGTCAAATCTGATTGCGGGCTTGCGGCCGCTCCTCCGGGCGAGGATACGGAG
CCGGCGTGGCGGATCCGGGAAGGCGTGTTCGCCCTCTCCTCGGCTCCTCGCTCGCAGGCGAAATCCCCTTCGGTTCTGTC
TATCGCTCCTGCTTTCCACTCTCTCCTGCTGTCGCGAAGCTGTAACCTGATTTCGGTGTAGTCTTATGTTAGCGTCAAAT
GTTCTTATCCATATTCCTTCTGCTGGGAAGGGGTTCATCAGTTGCCTGAAACCTTCGTTTCCGGTCCTCACTCATCTTGC
TGTCATTAGCTGTCTTGCGTTTCTATCTACCCTATTATATGGATATTGTCATACTGATATATCATTATGGTGAACGCGAA
ACTGAACTCGGTGACTGGCTTAGCGCCAAATGTTTGCTTCCTTTTGTTTTATACTAGGGCACTCAGTTCAGTTTGTCTAC
TTTGGGTCCTTAATCAAATCTAAATTAAATGGGAGAGACGATTTGTTCATTTGTTTCGGCCAAAATGGTTGCCCTTTTTT
CATGGTTGTTGGGCTTTTGTAAAGAACTTCAGCAGATGGCGTGCAGATCCAAGCAACCCTTTCCCATCTTTTGTTGTATA
CAATTGTTTAAAGCTTTATTTACGAAAGGTTGTAAACGGAATTTCAGAATCAGATTAACGACGGATAACTGCTACTGTTT
GCAACGCAAACCTTTAGTTGGAGAAATGTGAAACACGCGTAGATCTGATGAAAGTTGAGTTTAACAGGCAATAATTCCAT
TCTGCTGCCAAGGACCTTATGCTATAGTGCTAGACTGTGTGATAGGGGACCAAGGACGCAGAGTCTGCCAAACCCTGCAA
ACCCATTGGGGACATTGAACTGGTCCTTAAAATTTCCTCTGATGTTAAGCTACTTTTTCCTTGAACCGGACCATGTTTTG
CAGATTTGTCTGTTGTGCTATAATTAGTTGTTCAGTATGCTTCTTCAGAAGCATTTATTTGTTTTGCTTCGACAGTTCTA
GAACATTTAGTGTATGGTTCTGCTATTTTCAGTTCCATTTTATTTCCTGGTTAGTTGTTATCAGCCAGGCTTTGAACAAT
ATGGACATCTGCACTAATCAGATTGGACAGACCCAGTTGATAGCGGCTCCCACTCATAACCGAGGCAGCCTTACCCTTGT
TTTTTAGAGAGGCTGCATTGAACACAAGACCTTTAAGAACTTTGTGACTGACAAAATGCTTTGCGATATCTGTACTGCTA
AGTCCCTATAAATGGTTTGAAAGCCACTGTCCAGAAGGCAATCCATTTGTGAATGGCCAAGACGCCAAGTAGATGTATCC
TTCTATTAGAAATCTATCTCCATTTTCCGTCCATTAGGAAGTAGGATGACTGTTTTCCTGTACCCCGCACATGCATTTTA
TCTCATGCTAGTGCTGAACCTATCACATGGCATTATTTCATGATTTAATCTGAACTACTAGTAAGGATGACATGGATCTG
CCTACTTCAGAGATGCCATTTTATCTGCCTTTGCATGTTCTTAATTTTTCTGCATTCTTTACTACAAGGTCTGGAATGTT
GCCGATGTCGAACGTGTCCGCTAATCCTTCACAGCTTACAATTTTCTATGGCGGATCAGTATGTGTGTATGACTCAGTGC
CACCGGAAAAGGTGATATTGTCTTCTTCATTTACAGATATCTTGTCCTTCTGTACTGTCTCTTAAAGACACATATGCATG
CTAACTCACTAATCTGGAATTGACAGGCTCAAGCAATCATGCTTATAGCTGCAGCTGCAGCGGCAGCGGCAGCTACCAAA
AGCAGTGCTGCCCCTACTGTTAAGCCTCCAATGGTGCCTGCAGCCACTGTTGCCCCAGCAGCTGTCGTTTCTCCTGTGCT
TACACGGTCTCCATCACTGCAGAGCACTTCTGTAGCAACTGGGCAACCTCAGGTTGTTGCTGAACCTAGCTCGATATGCA
AACTTCAGGCTGGTAAGACCACAGCATCTTTTGGGTAAAAAACCAGAAATGGAATACCTTTTTCAGTGTTGACATTTTTC
TAACATATCTTTAAACTACCTTAATCCGCTGGCCAGATCTCCCCATTGCCAGGAGGCACTCTCTTCAGCGTTTCCTTGAG
AAACGCCGTGACAGGTTAGCATGGTAGATGATAGCAACAGCGCTATGATGTTCTGAATCTCTTCTTAGGCTCCTTCTACA
TGTTGAAAACTTTAGTTATAAACTGGTATATGTGGTACAACAGTATTCAACAGCATGCTTTGTTCCGTGTTCTATTTTTA
TCCTCTAGGTAGGGTAAAAAATTGAAATAATTTGCTGTTATAATTGTATTTAAATGATTGCACTTTGTGCTCGATGACTG
CATAAAGTAAACGAAATGTGACTAATTCATTTAAAACATTCCTCAAAAATCCTAAAAATTTGTTTACAGCAGGAATGAGT
AGAGAGCCTGCTTACATAATAGAACACATAGCAGCTAACAGCGTTGGATCATGCAACTTTCCTGACTTTTTAAATATCTG
CAAAAACCGAAATTAAGGGTGGTCACCTAAAGGCCCATGTGTCATCTGTAGGTTGCAAACTAGCTGAACACCATTTTGTT
CTTCAGATGTCAGGTTTAGATGGCTATTTTGCCTTATCTTAAAACTGAGTTTAGAATTGCCTTTTGTCATGGGTTTGCTG
AATTCGACTGCTGTTGGCATGAAACAGGGTCGTGAGCAAAGCTCCATACAGCACCGCCAAGTCGTCCGATGGCATGGAGT
CACCGAGGATTGAGGTGACAGCGGAGGGCAAGGCCCAGTAACTTGAAAGGTCCCCGCGGAGTCCCATCGGTGAAGGCTGA
CGTTGTGGGAAGGGGCAGGGACGCGCTGTTGTTGCCTTATGTTATGTGGCGGCGGCAGCGTGGAACCGCACTAATAATGT
GGACCGGAACCTCTGTTACCGTCGTTTGTGCTGTCGCGTGCTAGGTTTATGCTGAAGTGGATTCCTTTACTCCGCACACC
GAACTGTGTCTGTTGTACATCACGCCGTGACGCCGGTGGTCTCGTCTGTCTGACTTCGTTACATGGATCGTCTAGTAGTA
ACGGAATGCAGTACCACGTGTGGACATGTGTTTAGTGTGAAAGATGATGGGGCTTTGCTTGCTTGTTTCCACT
>Zm00001d002029_T001 cDNA
AAATCTGATTGCGGGCTTGCGGCCGCTCCTCCGGGCGAGGATACGGAGCCGGCGTGGCGGATCCGGGAAGGCGTGTTCGC

CCTCTCCTCGGCTCCTCGCTCGCAGGCGAAATCCCCTTCGGTTCTGTCTATCGCTCCTGCTTTCCACTCTCTCCTGCTGT
CGCGAAGCTGTAACCTGATTTCGGTGTAGTCTTATGTTAGCGTCAAATGTTCTTATCCATATTCCTTCTGCTGGGAAGGG
GTTCATCAGTTGCCTGAAACCTTCGTTTCCGGTCCTCACTCATCTTGCTGTCATTAGCTGTCTTGCGTTTCTATCTACCC
TATTATATGGATATTGTCATACTGATATATCATTATGGTGAACGCGAAACTGAACTCGGTGACTGGCTTAGCGCCAAATG
TTTGCTTCCTTTTGTTTTATACTAGGGCACTCAGTTCAGTTTGTCTACTTTGGGTCCTTAATCAAATCTAAATTAAATGG
GAGAGACGATTTGTTCATTTGTTTCGGCCAAAATGGTTGCCCTTTTTTCATGGTTGTTGGGCTTTTGTAAAGAACTTCAG
CAGATGGCGTGCAGATCCAAGCAACCCTTTCCCATCTTTTGTTGTATACAATTGTTTAAAGCTTTATTTACGAAAGGTTG
TAAACGGAATTTCAGAATCAGATTAACGACGGATAACTGCTACTGTTTGCAACGCAAACCTTTAGTTGGAGAAATGTGAA
ACACGCGTAGATCTGATGAAAGTTGAGTTTAACAGGCAATAATTCCATTCTGCTGCCAAGGACCTTATGCTATAGTGCTA
GACTGTGTGATAGGGGACCAAGGACGCAGAGTCTGCCAAACCCTGCAAACCCATTGGGGACATTGAACTGGTCCTTAAAA
TTTCCTCTGATGTTAAGCTACTTTTCCTTGAACCGGACCATGTTTTGCAGATTTGTCTGTTGTGCTATAATTAGTTGTT
CAGTATGCTTCTTCAGAAGCATTTATTTGTTTTGCTTCGACAGTTCTAGAACATTTAGTGTATGGTTCTGCTATTTTCAG
TTCCATTTTATTTCCTGGTTAGTTGTTATCAGCCAGGCTTTGAACAATATGGACATCTGCACTAATCAGATTGGACAGAC
CCAGTTGATAGCGGCTCCCACTCATAACCGAGGCAGCCTTACCCTTGTTTTTAGAGAGGCTGCATTGAACACAAGACCT
TTAAGAACTTTGTGACTGACAAAATGCTTTGCGATATCTGTACTGCTAAGTCCCTATAAATGGTTTGAAAGCCACTGTCC
AGAAGGCAATCCATTTGTGAATGGCCAAGACGCCAAGTAGATGTATCCTTCTATTAGAAATCTATCTCCATTTTCCGTCC
ATTAGGAAGTAGGATGACTGTTTTCCTGTACCCCGCACATGCATTTTATCTCATGCTAGTGCTGAACCTATCACATGGCA
TTATTTCATGATTTAATCTGAACTACTAGTAAGGATGACATGGATCTGCCTACTTCAGAGATGCCATTTTATCTGCCTTT
GCATGTTCTTAATTTTTCTGCATTCTTTACTACAAGGTCTGGAATGTTGCCGATGTCGAACGTGTCCGCTAATCCTTCAC
AGCTTACAATTTTCTATGGCGGATCAGTATGTGTGTATGACTCAGTGCCACCGGAAAAGGCTCAAGCAATCATGCTTATA
GCTGCAGCTGCAGCGGCAGCGGCAGCTACCAAAAGCAGTGCTGCCCCTACTGTTAAGCCTCCAATGGTGCCTGCAGCCAC
TGTTGCCCCAGCAGCTGTCGTTTCTCCTGTGCTTACACGGTCTCCATCACTGCAGAGCACTTCTGTAGCAACTGGGCAAC
CTCAGGTTGTTGCTGAACCTAGCTCGATATGCAAACTTCAGGCTGATCTCCCCATTGCCAGGAGGCACTCTCTTCAGCGT
TTCCTTGAGAAACGCCGTGACAGGGTCGTGAGCAAAGCTCCATACAGCACCGCCAAGTCGTCCGATGGCATGGAGTCACC
GAGGATTGAGGTGACAGCGGAGGGCAAGGCCCAGTAACTTGAAAGGTCCCCGCGGAGTCCCATCGGTGAAGGCTGACGTT
GTGGGAAGGGGCAGGGACGCGCTGTTGTTGCCTTATGTTATGTGGCGGCGGCAGCGTGGAACCGCACTAATAATGTGGAC
CGGAACCTCTGTTACCGTCGTTTGTGCTGTCGCGTGCTAGGTTTATGCTGAAGTGGATTCCTTTACTCCGCACACCGAAC
TGTGTCTGTTGTACATCACGCCGTGACGCCGGTGGTCTCGTCTGTCTGACTTCGTTACATGGATCGTCTAGTAGTAACGG
AATGCAGTACCACGTGTGGACATGTGTTTAGTGTGAAAGATGATGGGGCTTTGCTTGCTTGTTTCCACT
>Zm00001d002029_P001 peptide
MDLPTSEMPFYLPLHVLNFSAFFTTRSGMLPMSNVSANPSQLTIFYGGSVCVYDSVPPEKAQAIMLIAAAAAAAAATKSS
AAPTVKPPMVPAATVAPAAVVSPVLTRSPSLQSTSVATGQPQVVAEPSSICKLQADLPIARRHSLQRFLEKRRDRVVSKA
PYSTAKSSDGMESPRIEVTAEGKAQ
>Zm00001d002029_T002 cDNA
Chr2: 4666311..4669499
GGGCTGTGGCCGTACCTTTCGCTGGGTCAAATCTGATTGCGGGCTTGCGGCCGCTCCTCCGGGCGAGGATACGGAGCCGG
CGTGGCGGATCCGGGAAGGCGTGTTCGCCCTCTCCTCGGCTCCTCGCTCGCAGGCGAAATCCCCTTCGGTCTGGAATGTT
GCCGATGTCGAACGTGTCCGCTAATCCTTCACAGCTTACAATTTTCTATGGCGGATCAGTATGTGTGTATGACTCAGTGC
CACCGGAAAAGGCTCAAGCAATCATGCTTATAGCTGCAGCTGCAGCGGCAGCGGCAGCTACCAAAAGCAGTGCTGCCCCT
ACTGTTAAGCCTCCAATGGTGCCTGCAGCCACTGTTGCCCCAGCAGCTGTCGTTTCTCCTGTGCTTACACGGTCTCCATC
ACTGCAGAGCACTTCTGTAGCAACTGGGCAACCTCAGGTTGTTGCTGAACCTAGCTCGATATGCAAACTTCAGGCTGATC
TCCCCATTGCCAGGAGGCACTCTCTTCAGCGTTTCCTTGAGAAACGCCGTGACAGGGTCGTGAGCAAAGCTCCATACAGC
ACCGCCAAGTCGTCCGATGGCATGGAGTCACCGAGGATTGAGGTGACAGCGGAGGGCAAGGCCCAGTAACTTGAAAGGTC
CCCGCGGAGTCCCATCGGTGAAGGCTGACGTTGTGGGAAGGGGCAGGGACGCGCTGTTGTTGCCTTATGTTATGTGGCGG

CGGCAGCGTGGAACCGCACTAATAATGTGGACCGGAACCTCTGTTACCGTCGTTTGTGCTGTCGCGTGCTAGGTTTATGC
TGAAGTGGATTCCTTTACTCCGCACACCGAACTGTGTCTGTTGTACATCACGCCGTGACGCCGGTGGTCTCGTCTGTCTG
ACTTCGTTACATGGATCGTCTAGTAGTAACGGAATGCAGTACCACGTGTGGACATGTGTTTAGTGTGAAAGATGATGGGG
CTTTGCTTGCTTGTTTCCACT
>Zm00001d002029_P002 peptide
MLPMSNVSANPSQLTIFYGGSVCVYDSVPPEKAQAIMLIAAAAAAAAATKSSAAPTVKPPMVPAATVAPAAVVSPVLTRS
PSLQSTSVATGQPQVVAEPSSICKLQADLPIARRHSLQRFLEKRRDRVVSKAPYSTAKSSDGMESPRIEVTAEGKAQ
>Zm00001d002029_T003 cDNA
Chr2: 4666314..4670063
GGTACTACACAGTTTCATCACATAGTTGCTGGCGGCTGGCCGGCCTCTTTCTCTTTCTCGTTTCTGCCCCCTCTGCTTGC
CCCCCTTTCATCTCAGGCGTCGAAGAGAGAGAGAGAGAGAGAGAGGAGTTGCAAGTTGGTGAGATGGATCTGTTGGAGCG
GAATATTAAGACGGAGACGGAGGAGACGCGCAAGGAACAGGAGCGTAAGGAGGAGGAGGCGGAGGCGGAGGCGGAGGCGG
GGGAGAGGAAGACCCAGGAGCCGCCGCAGCAAGGCCAGGGCCTCAGCCTCTCACTCGCCAACGGCAGTGGCAGGTCTGGA
ATGTTGCCGATGTCGAACGTGTCCGCTAATCCTTCACAGCTTACAATTTTCTATGGCGGATCAGTATGTGTGTATGACTC
AGTGCCACCGGAAAAGGCTCAAGCAATCATGCTTATAGCTGCAGCTGCAGCGGCAGCGGCAGCTACCAAAAGCAGTGCTG
CCCCTACTGTTAAGCCTCCAATGGTGCCTGCAGCCACTGTTGCCCCAGCAGCTGTCGTTTCTCCTGTGCTTACACGGTCT
CCATCACTGCAGAGCACTTCTGTAGCAACTGGGCAACCTCAGGTTGTTGCTGAACCTAGCTCGATATGCAAACTTCAGGC
TGATCTCCCCATTGCCAGGAGGCACTCTCTTCAGCGTTTCCTTGAGAAACGCCGTGACAGGGTCGTGAGCAAAGCTCCAT
ACAGCACCGCCAAGTCGTCCGATGGCATGGAGTCACCGAGGATTGAGGTGACAGCGGAGGGCAAGGCCCAGTAACTTGAA
AGGTCCCCGCGGAGTCCCATCGGTGAAGGCTGACGTTGTGGGAAGGGGCAGGGACGCGCTGTTGTTGCCTTATGTTATGT
GGCGGCGGCAGCGTGGAACCGCACTAATAATGTGGACCGGAACCTCTGTTACCGTCGTTTGTGCTGTCGCGTGCTAGGTT
TATGCTGAAGTGGATTCCTTTACTCCGCACACCGAACTGTGTCTGTTGTACATCACGCCGTGACGCCGGTGGTCTCGTCT
GTCTGACTTCGTTACATGGATCGTCTAGTAGTAACGGAATGCAGTACCACGTGTGGACATGTGTTTAGTGTGAAAGATGA
TGGGGCTTTGCTTGCTTGTTTCC
>Zm00001d002029_P003 peptide
MDLLERNIKTETEETRKEQERKEEEAEAEAEAGERKTQEPPQQGQGLSLSLANGSGRSGMLPMSNVSANPSQLTIFYGGS
VCVYDSVPPEKAQAIMLIAAAAAAAAATKSSAAPTVKPPMVPAATVAPAAVVSPVLTRSPSLQSTSVATGQPQVVAEPSS
ICKLQADLPIARRHSLQRFLEKRRDRVVSKAPYSTAKSSDGMESPRIEVTAEGKAQ

ZmJAZ9 (TIFY13)

B73 RefGen_v3:
GRMZM2G145407 (zim33 - ZIM-transcription factor 33)
Chr2: 65326353..65328811

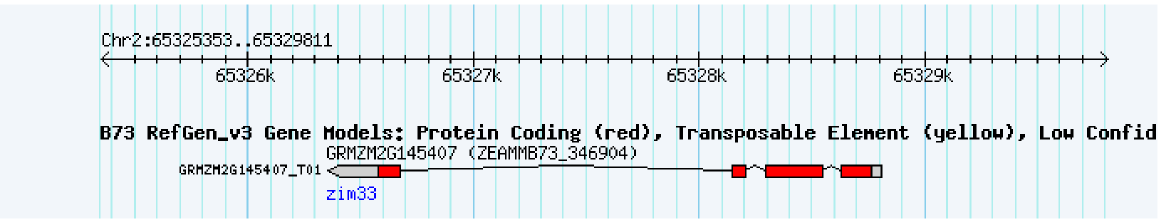

>GRMZM2G145407 Genomic DNA
CTAGCACGCAACGCAACCTCCCTCAGGTGTCGGGTGCCGGCGGCCATGCCGTCGACGGCGCCTGTAGAGCTCGACTTCCT
CGGCCTCCGTCCTGCTGCTGCGGCCGCCGCCGAAGACGACCACCACCACGGCAGCAGCACCACCTCCGCCGCCGCAGCTG
CCTCCTCCTCCGTCCGAGGTCTCTGTCTCTCGATCGCGTCTTTCTTTCTCCCATCGATCCCAACACCTACGTAAACCGGA
TCATCGGTGCATCCGTGCATGCAGGCATGAAGACGAGCGCGATAGCGAGCATCGGGGCGCACAAGCTGCGCCGCGTCATC

GCCGGCGACGAGGCGACCAAGCAGCATCAGCAGCAGCAGGCCCCCATGACGGTCTTCTACGGCGGCGCCGTGGCCACCTT
CGACGGCGTCTCCCAGGACACGGCGGAGGCTATAATGAAGATGGCGATGGAGGTCACTGCTTCCAGCGGAGGCCGCATCG
TCCGCCGCGACGCATTTCGTGGAAATTTAACCAAAGGTATATAGCTGGTCGTCGTTTTGGGTTCGCGCGACGACCTTATT
AATCACCATTTCGTCCTCGCTCTGATTTGTTCTTGCTTCGCAGACATGCCGCTCACAAGAACCAAATCGCTGCAGCAGTT
TCTACAGAAGCGAAAGGAGAGGTATAAACGCATACACGGCCACATCATCCAACCCCATGTACATGTGTAATTCTGTGTGA
TTAATTAGTTAATTAAGTAAAGTACTACGTACATTAGTTTCATATTAATATAAGAGATTAATTATATGTGTTCAATAAGA
AACTTACGCGTTACATTATATTTTTAGCACATATTATATAGTACATTTCTACTCACAAAGACGATTTGAGTAGCGCAAAT
AATAATCGCATGCGTATCAGGTTTGGTCACAAAGACGATGATTTCCCCAACTTTATGAATATTCCACCAATGACTCGCGT
ACGCATAGAAGTGGGAAGCAGTAGGTAAAAATTTAGAAAAGAAAAGAAACTGAGTGTTTAGATTCTATGAACCTTCTTCG
GTGTTCAGTATCTGTGTATCATGGTTAGCGTGTTTTGTGTGTAGAAATAATACCATACACACACGTACGTCCAGGACGCT
GAACTAACATATTTTGTTAGCGTGTTCAGTATCTGTTGGATACAAAGTTTTGTTAGAAGTTTCTCCTTTTTAAGGAAAAA
AAGCACATATAATAATACAAATGTGACTAGCGCTTTCAGCTGTTATATATTTTTAACACGTAGCAAAATATACTACTCCT
ATTATGAAACAGATTTACTTGTACCATCTTATTTTTTTTAAAAAAAAGATCAAATTTTGGAACAGAACAATAACTTGCT
GTAGTCTGAGTTCAAAATGCATGTTCGAGTACTATATAATAATAATAATAATAATAATAATAATAATAATAATAATAATA
ATAATAATGTCAAAACATCACTTATTTGTCTCCACAAAAACATACTACTTCCACTTCAACAGTATATATATAGATAAATA
TATAAACAAAATTAAACAGGGTACGTACGTTACGTACGTGTGTGTGATATCAGTCGTTGTTGCTGGCACCAGCGAAAGTA
TACATATAGACCGTGTGGGCCCATTATTTGGACCACACAACAATCTCCATCGGTCAGTTGAAGAAGGTAAGCGCACACAA
TCAAATCCCCACGTTTTGGACGTGACGGGACAGCGATAGCTAGCTAGCTTTTGCACGCGGTCGATTGCCCAGCACCTACC
GAATCGATCGACGACCGCAATTATACTGCCGCACACCAGGCCTCCAGCTGTTCTTTTTTCACAAAGGAATATTTTGTTCT
CATCACATCTGATTTGGTTGGTTAATTAGTTTGGACCGAAAAAACGCAGACACAGTTGGCGCGCGCGCGTGCGTCACGCT
TTCTACAATGTTTCTTACTACTCCGTAACATGCATGATCACATGTTTTCCAATCTAGATTTTGGGATACATGAATTTTGA
CCAAACTCTCTCTCTCTCTCTCTCATTAATAAACCACTACCTGCCATTTTTTAATTATAATCATGTAAGCTACAGATCGA
TGACGCTATTACAGTCAACTGATTCGTTTCGTGTGGATGAATTATTAAACGTGCAGGCTGAGTGGGCCGGGTCCTTATCT
GCCAGCATCGGGAGGACGAAGCAGCCGCGTTGGTGCAGCGACCATGACGTTTCATGTGAAGGAGGAAGCAGCTTAATAAG
CCTATAGCTACCTTACTTGGCTTGGAGTCCACTGCAGCAAGCTACTAATTCAAAAAGGATTCTTTGACAGTATTTTCTTT
ATTCGATAGTATAATTGTATCTACAATTTTTTTAAAAAAAATCCGCAGGGATAAGTTCGTTTCACAATTTCATTCATGAT
TGTAGATAAGGTTAACTCTCGAGTTGATCATAGCCATATCTATGCTTGTTATACATATA
>GRMZM2G145407_T01 cDNA
CTAGCACGCAACGCAACCTCCCTCAGGTGTCGGGTGCCGGCGGCCATGCCGTCGACGGCGCCTGTAGAGCTCGACTTCCT
CGGCCTCCGTCCTGCTGCTGCGGCCGCCGCCGAAGACGACCACCACCACGGCAGCAGCACCACCTCCGCCGCCGCAGCTG
CCTCCTCCTCCGTCCGAGGCATGAAGACGAGCGCGATAGCGAGCATCGGGGCGCACAAGCTGCGCCGCGTCATCGCCGGC
GACGAGGCGACCAAGCAGCATCAGCAGCAGCAGGCCCCCATGACGGTCTTCTACGGCGGCGCCGTGGCCACCTTCGACGG
CGTCTCCCAGGACACGGCGGAGGCTATAATGAAGATGGCGATGGAGGTCACTGCTTCCAGCGGAGGCCGCATCGTCCGCC
GCGACGCATTTCGTGGAAATTTAACCAAAGACATGCCGCTCACAAGAACCAAATCGCTGCAGCAGTTTCTACAGAAGCGA
AAGGAGAGGCTGAGTGGGCCGGGTCCTTATCTGCCAGCATCGGGAGGACGAAGCAGCCGCGTTGGTGCAGCGACCATGAC
GTTTCATGTGAAGGAGGAAGCAGCTTAATAAGCCTATAGCTACCTTACTTGGCTTGGAGTCCACTGCAGCAAGCTACTAA
TTCAAAAAGGATTCTTTGACAGTATTTTCTTTATTCGATAGTATAATTGTATCTACAATTTTTTTAAAAAAAATCCGCAG
GGATAAGTTCGTTTCACAATTTCATTCATGATTGTAGATAAGGTTAACTCTCGAGTTGATCATAGCCATATCTATGCTTG
TTATACATATA
>GRMZM2G145407_P01 peptide
MPSTAPVELDFLGLRPAAAAAAEDDHHHGSSTTSAAAAASSSVRGMKTSAIASIGAHKLRRVIAGDEATKQHQQQQAPMT
VFYGGAVATFDGVSQDTAEAIMKMAMEVTASSGGRIVRRDAFRGNLTKDMPLTRTKSLQQFLQKRKERLSGPGPYLPASG
GRSSRVGAATMTFHVKEEAA
AGPv4:

Zm00001d003903 (zim33 - ZIM-transcription factor 33)
Chr2: 66485018..66487506

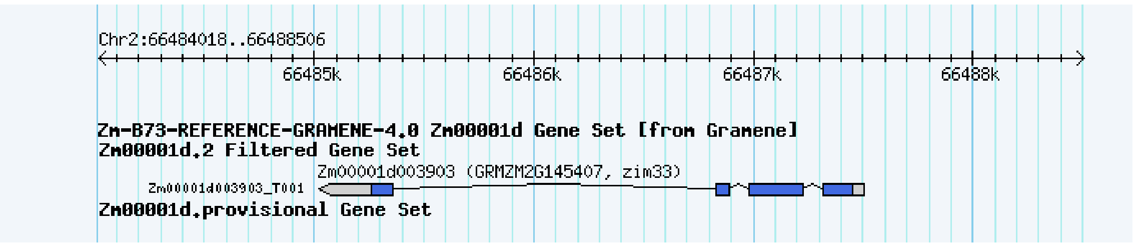

>Zm00001d003903 Genomic DNA
CTCGCTCCTTCAGCTAGCACGCAACGCAACCTCCCTCAGGTGTCGGGTGCCGGCGGCCATGCCGTCGACGGCGCCTGTAG
AGCTCGACTTCCTCGGCCTCCGTCCTGCTGCTGCGGCCGCCGCCGAAGACGACCACCACCACGGCAGCAGCACCACCTCC
GCCGCCGCAGCTGCCTCCTCCTCCGTCCGAGGTCTCTGTCTCTCGATCGCGTCTTTCTTTCTCCCATCGATCCCAACACC
TACGTAAACCGGATCATCGGTGCATCCGTGCATGCAGGCATGAAGACGAGCGCGATAGCGAGCATCGGGGCGCACAAGCT
GCGCCGCGTCATCGCCGGCGACGAGGCGACCAAGCAGCATCAGCAGCAGCAGGCCCCCATGACGGTCTTCTACGGCGGCG
CCGTGGCCACCTTCGACGGCGTCTCCCAGGACACGGCGGAGGCTATAATGAAGATGGCGATGGAGGTCACTGCTTCCAGC
GGAGGCCGCATCGTCCGCCGCGACGCATTTCGTGGAAATTTAACCAAAGGTATATAGCTGGTCGTCGTTTTGGGTTCGCG
CGACGACCTTATTAATCACCATTTCGTCCTCGCTCTGATTTGTTCTTGCTTCGCAGACATGCCGCTCACAAGAACCAAAT
CGCTGCAGCAGTTTCTACAGAAGCGAAAGGAGAGGTATAAACGCATACACGGCCACATCATCCAACCCCATGTACATGTG
TAATTCTGTGTGATTAATTAGTTAATTAAGTAAAGTACTACGTACATTAGTTTCATATTAATATAAGAGATTAATTATAT
GTGTTCAATAAGAAACTTACGCGTTACATTATATTTTTAGCACATATTATATAGTACATTTCTACTCACAAAGACGATTT
GAGTAGCGCAAATAATAATCGCATGCGTATCAGGTTTGGTCACAAAGACGATGATTTCCCCAACTTTATGAATATTCCAC
CAATGACTCGCGTACGCATAGAAGTGGGAAGCAGTAGGTAAAAATTTAGAAAAGAAAAGAAACTGAGTGTTTAGATTCTA
TGAACCTTCTTCGGTGTTCAGTATCTGTGTATCATGGTTAGCGTGTTTTGTGTGTAGAAATAATACCATACACACACGTA
CGTCCAGGACGCTGAACTAACATATTTGTTAGCGTGTTCAGTATCTGTTGGATACAAAGTTTTGTTAGAAGTTTCTCCT
TTTTAAGGAAAAAAAGCACATATAATAATACAAATGTGACTAGCGCTTTCAGCTGTTATATATTTTTAACACGTAGCAAA
ATATACTACTCCTATTATGAAACAGATTTACTTGTACCATCTTATTTTTTTTAAAAAAAAGATCAAATTTTGGAACAGA
ACAATAACTTGCTGTAGTCTGAGTTCAAAATGCATGTTCGAGTACTATATAATAATAATAATAATAATAATAATAATAAT
AATAATAATAATAATAATAATGTCAAAACATCACTTATTTGTCTCCACAAAAACATACTACTTCCACTTCAACAGTATAT
ATATAGATAAATATATAAACAAAATTAAACAGGGTACGTACGTTACGTACGTGTGTGTGATATCAGTCGTTGTTGCTGGC
ACCAGCGAAAGTATACATATAGACCGTGTGGGCCCATTATTTGGACCACACAACAATCTCCATCGGTCAGTTGAAGAAGG
TAAGCGCACACAATCAAATCCCCACGTTTTGGACGTGACGGGACAGCGATAGCTAGCTAGCTTTTGCACGCGGTCGATTG
CCCAGCACCTACCGAATCGATCGACGACCGCAATTATACTGCCGCACACCAGGCCTCCAGCTGTTCTTTTTTCACAAAGG
AATATTTTGTTCTCATCACATCTGATTTGGTTGGTTAATTAGTTTGGACCGAAAAAACGCAGACACAGTTGGCGCGCGCG
CGTGCGTCACGCTTTCTACAATGTTTCTTACTACTCCGTAACATGCATGATCACATGTTTTCCAATCTAGATTTTGGGAT
ACATGAATTTTGACCAAACTCTCTCTCTCTCTCTCTCATTAATAAACCACTACCTGCCATTTTTTAATTATAATCATGTA
AGCTACAGATCGATGACGCTATTACAGTCAACTGATTCGTTTCGTGTGGATGAATTATTAAACGTGCAGGCTGAGTGGGC
CGGGTCCTTATCTGCCAGCATCGGGAGGACGAAGCAGCCGCGTTGGTGCAGCGACCATGACGTTTCATGTGAAGGAGGAA
GCAGCTTAATAAGCCTATAGCTACCTTACTTGGCTTGGAGTCCACTGCAGCAAGCTACTAATTCAAAAAGGATTCTTTGA
CAGTATTTTCTTTATTCGATAGTATAATTGTATCTACAATTTTTTTAAAAAAAATCCGCAGGGATAAGTTCGTTTCACAA
TTTCATTCATGATTGTAGATAAGGTTAACTCTCGAGTTGATCATAGCCATATCTATGCTTGTTATACATATATTTATTTC
TTTTTACCC
>Zm00001d003903_T001 cDNA
CTCGCTCCTTCAGCTAGCACGCAACGCAACCTCCCTCAGGTGTCGGGTGCCGGCGGCCATGCCGTCGACGGCGCCTGTAG
AGCTCGACTTCCTCGGCCTCCGTCCTGCTGCTGCGGCCGCCGCCGAAGACGACCACCACCACGGCAGCAGCACCACCTCC

GCCGCCGCAGCTGCCTCCTCCTCCGTCCGAGGCATGAAGACGAGCGCGATAGCGAGCATCGGGGCGCACAAGCTGCGCCG
CGTCATCGCCGGCGACGAGGCGACCAAGCAGCATCAGCAGCAGCAGGCCCCCATGACGGTCTTCTACGGCGGCGCCGTGG
CCACCTTCGACGGCGTCTCCCAGGACACGGCGGAGGCTATAATGAAGATGGCGATGGAGGTCACTGCTTCCAGCGGAGGC
CGCATCGTCCGCCGCGACGCATTTCGTGGAAATTTAACCAAAGACATGCCGCTCACAAGAACCAAATCGCTGCAGCAGTT
TCTACAGAAGCGAAAGGAGAGGCTGAGTGGGCCGGGTCCTTATCTGCCAGCATCGGGAGGACGAAGCAGCCGCGTTGGTG
CAGCGACCATGACGTTTCATGTGAAGGAGGAAGCAGCTTAATAAGCCTATAGCTACCTTACTTGGCTTGGAGTCCACTGC
AGCAAGCTACTAATTCAAAAAGGATTCTTTGACAGTATTTTCTTTATTCGATAGTATAATTGTATCTACAATTTTTTTAA
AAAAAATCCGCAGGGATAAGTTCGTTTCACAATTTCATTCATGATTGTAGATAAGGTTAACTCTCGAGTTGATCATAGCC
ATATCTATGCTTGTTATACATATATTTATTTCTTTTTACCC
>Zm00001d003903_P001 peptide
MPSTAPVELDFLGLRPAAAAAAEDDHHHGSSTTSAAAAASSSVRGMKTSAIASIGAHKLRRVIAGDEATKQHQQQQAPMT
VFYGGAVATFDGVSQDTAEAIMKMAMEVTASSGGRIVRRDAFRGNLTKDMPLTRTKSLQQFLQKRKERLSGPGPYLPASG
GRSSRVGAATMTFHVKEEAA

ZmJAZ10

B73 RefGen_v3
GRMZM2G171830 (zim8 - ZIM-transcription factor 8)
Chr2: 97375739..97377764

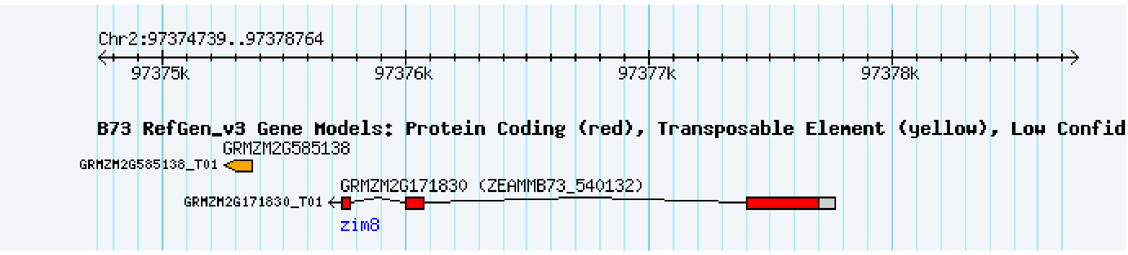

>GRMZM2G171830 Genomic DNA
GCCTGATGCCCGGCACGGACGTCGGCGCCGATGAGAAGTAGGAGCCGGCCGCCGCAAGGGCTGAGGAGATGTCTGGGCCC
GCCCCCGTCACGGCGCCACCGCTCACCATCTTCTACGACGGCAGGGTGGTGGTGTTCGAGGACTTTCTAGCGGAGAAGGC
GGTCGAGGTCATGCGCCTCGCGGCCGGTGACGACTTGCCCATCGCCCGGAAGGCGACGCTGCAGCGATTCCTGGCCAAGC
GCAAGGACCGCCTCGTCAAGCGCGTGGCCCTACACCCGCCCATCGTCCCCCACGGAGGAGCTGGAGAAGAAGACGGGCAA
GCCGGCCTCGGCCTCGGCCACATGCTCGACAAAATGACTGTGTGATCGCCGTTGCCTGAACCGGGCTGGCCACCTCTACG
CTCATGCCTCTCACGTTTCTGGAGGGTGCAGAGTAGCCGGTCGACCGGTCGAGCTCCCTACGCCTCCTGTGCATGGCCTA
AAACTCTCTGCTCTCGCTTGCATGCCTTGCTCTGCATGGCATCAGAGCGCACGGACGCCGCAACAATGGTTGCCAGTAGA
ACCTTGACGGCTGCCGCTGGTTTCCCTGCCCCTAGCTCGAGCGCGCATAGCCGATGGGACCTCCACTATTTCCCCTCCTC
AGCGCGCTCCATGCTGAGCTCCCGCAGGCCACAACGGTCGGGTGCCGATCACCATGGCCACTGACCACCGGACAGAGGCG
CAGTCCCGTGGAACTCCAATCCTGTTGAGCCCTCTCAGCCTCTCAGGTCCGCACTGAACCGTGCTACAACCGGAGGAATC
CTCTCTCGCTCCTCCACCACCGCATGGCGCCACCGAGCGATTGCCGCCGGCCTACATGCGATGAGTCCTGTGCAGACTAA
CTTCTCTTTCAAATAGTAGAACCACCCATAATCTCTTGTGATTTGGATGAGACCTTAATTACCATGAACTACATGGCACA
TGCTAATCGTTTTGTGTAATTACTAATTTACTATAGAAATACTATATAAAACCTCATGCCATAACTTAGGAATATGTTGT
GTTTCATTTTTCTTTATTTGTTAGGTTGTATTTGATGTGATGTCGGGCTGCATTTGTTGGGAAACTTTAATGTTCTATAC
TTCCATTGTTAGTGCTTGTATGCATATGCTATTGTATAAATAGTAGGATTTAATGTCCCCTTCTGAGAGTTAACCAACTT
CATTATTGCTCTTGAGGCAACTAAAATGACTAAACATTTATTATATACCATTGACTCATCATTTGTTGTACTATTTTTTA
ACAACACCCTACTCTATCCATTTAGGGTGTGATTTTCGCAATCGATAATGGAGCGCATCATGATTTCAAACTGGATGAAT
TATCCATTGCCTCGCTTGTTTGTAGACTGAAGCAGACTACGGTACTTAATTTATTTTTAGTTATTCCCTGTTTGTAGACT

GAAGCAGGCTAAAACAAATACAAGTATACAGTTAAGTGGGGATGACATATAGTTGTCTGAAAAAACCATCTTGTATTTAT
TTGAAATTATACAAGAATGATGAATGCATATGCATTTTCGGATCATCATGTTCCCCTGTATTGAGTTAACTCTGGATTTG
ACTATAAAGGGGATAGCTTATGCTTGGATTTCATGTTGGCTTTACTTTTAGTGTGTGCTACAAACATAACTAAATTGTAT
TTTGCGCAGGTTGATGGAATTGAAATTCTTGTTGTGAAGCAGGCATGCAAATTTGCAAAAGATCACGATGTCTCAAATGG
CCATATTGTGAGTAACTTCTATTACTTGGATGCACCCCATATAGAACTACTATTAGGAGTTCAGAGTTAGCTTGTTGTTT
AGTATATTTAGTTAAATTCACATGAACTGCACTACACTTAGATACTGGGAAACATAATATATAATTCTAATGTATGGATC
TTCCATAGTCAAAACTCCACATAATATTTTTTTAAGATTCCTGTCACTAGATTATTATTGAATTTTTTAGGTGAGCCGCC
GCAGCCAAGAGGAAGCGCGGGCATAG

>GRMZM2G171830_T01 cDNA

GCCTGATGCCCGGCACGGACGTCGGCGCCGATGAGAAGTAGGAGCCGGCCGCCGCAAGGGCTGAGGAGATGTCTGGGCCC
GCCCCCGTCACGGCGCCACCGCTCACCATCTTCTACGACGGCAGGGTGGTGGTGTTCGAGGACTTTCTAGCGGAGAAGGC
GGTCGAGGTCATGCGCCTCGCGGCCGGTGACGACTTGCCCATCGCCCGGAAGGCGACGCTGCAGCGATTCCTGGCCAAGC
GCAAGGACCGCCTCGTCAAGCGCGTGGCCCTACACCCGCCCATCGTCCCCCACGGAGGAGCTGGAGAAGAAGACGGGCAA
GCCGGCCTCGGCCTCGGCCACATGCTCGACAAAATGACTGTTGATGGAATTGAAATTCTTGTTGTGAAGCAGGCATGCAA
ATTTGCAAAAGATCACGATGTCTCAAATGGCCATATTGTGAGCCGCCGCAGCCAAGAGGAAGCGCGGGCATAG

>GRMZM2G171830_P01 peptide

MSGPAPVTAPPLTIFYDGRVVVFEDFLAEKAVEVMRLAAGDDLPIARKATLQRFLAKRKDRLVKRVALHPPIVPHGGAGE
EDGQAGLGLGHMLDKMTVDGIEILVVKQACKFAKDHDVSNGHIVSRRSQEEARA

AGPv4:

Zm00001d004277 (zim8 - ZIM-transcription factor 8)

Chr2: 99601657..99601953

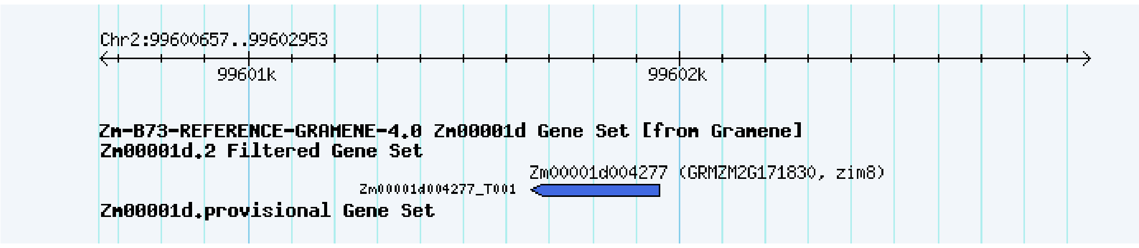

>Zm00001d004277 Genomic DNA

ATGTCTGGGCCCGCCCCCGTCACGGCGCCACCGCTCACCATCTTCTACGACGGCAGGGTGGTGGTGTTCGAGGACTTTCT
AGCGGAGAAGGCGGTCGAGGTCATGCGCCTCGCGGCCGGTGACGACTTGCCCATCGCCCGGAAGGCGACGCTGCAGCGAT
TCCTGGCCAAGCGCAAGGACCGCCTCGTCAAGCGCGTGGCCCTACACCCGCCCATCGTCCCCCACGGAGGAGCTGGAGAA
GAAGACGGGCAAGCCGGCCTCGGCCTCGGCCACATGCTCGACAAAATGACTGTGTGA

>Zm00001d004277_T001 cDNA

ATGTCTGGGCCCGCCCCCGTCACGGCGCCACCGCTCACCATCTTCTACGACGGCAGGGTGGTGGTGTTCGAGGACTTTCT
AGCGGAGAAGGCGGTCGAGGTCATGCGCCTCGCGGCCGGTGACGACTTGCCCATCGCCCGGAAGGCGACGCTGCAGCGAT
TCCTGGCCAAGCGCAAGGACCGCCTCGTCAAGCGCGTGGCCCTACACCCGCCCATCGTCCCCCACGGAGGAGCTGGAGAA
GAAGACGGGCAAGCCGGCCTCGGCCTCGGCCACATGCTCGACAAAATGACTGTGTGA

>Zm00001d004277_P001 peptide

MSGPAPVTAPPLTIFYDGRVVVFEDFLAEKAVEVMRLAAGDDLPIARKATLQRFLAKRKDRLVKRVALHPPIVPHGGAGE
EDGQAGLGLGHMLDKMTV

ZmJAZ11

B73 RefGen_v3:

GRMZM2G005954 (zim13 - ZIM-transcription factor 13)

Chr2: 184649167..184651569

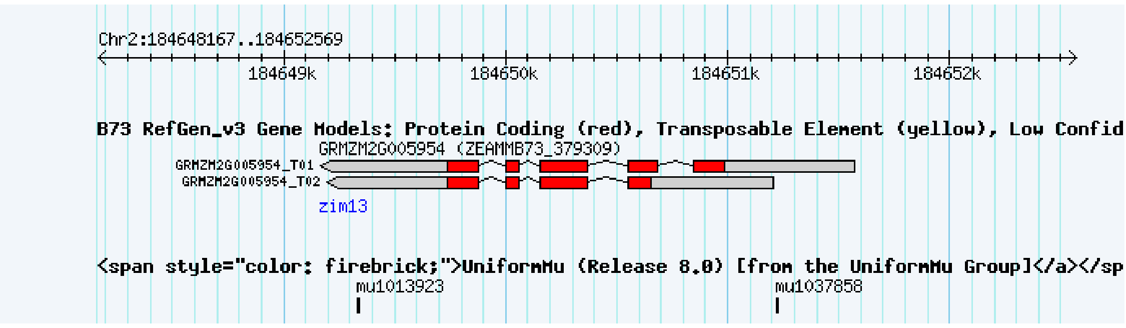

>GRMZM2G005954 Genomic DNA

CCCGTCGCTGCCATCGTCGCGTCCTCTCATCGTCCCCGGCGCGCGCGCCCCTCCTCCCAGATCACGTGTCGCTACCACGC
CTTCCCGTCGGGGCGTCCCTCTCGCTCGCCTGCCCCTCCTCCTCCTCCTCCTCCTCCTCCGTCCGATCACGTCGCCA
CCGCGGCCCCTCGGACCCGTCGCTCTCCCACATTCCCGTTCCCCGAGGACCACCATCCTCCGTTCCGGCGGCAGCCACGT
TTTTCATGCGAGGCGAAAGGCGGCCTAGTCGCAAAGGCCATTTTCTCCGTGGCCCCCCCCACACTCGCCGTTTCCTTGGC
ACACGCGCTATTATTAATAAAGGCGCAAAGGCCATTCGCGAGGCGAGGGGAGGAGAAGTGTACTACGCGAGAGCTTGCCG
GAGGCTCTGGATCCCTGTGATAATAATAAACTGATATAAGCAGAGGAAGCGGGGAGGAAGCTGCGAGTTGGAGGGGGCCG
GGAGAGGCGTCTCTGGTTATTGCTTGTCCCTGCTTCCCGGTGAGCTGCTGGGAGTGGACGGGCCGGGAAGGAGACGAGAA
GATAAGGGCGCCGCGCGCGGCCATGGCTGGAAGTGCGCCGGCGACGGCGATGGACAAGACCAGCTTTGCCACCACGTGCA
GCCTGCTGAGCCAGTACGTCAAGGAGAAGAAGGGCGGCCTGCTGCAGGGCCTTGGTGCCCTCGCCATGGCGCCGGCAGCT
GGTAAGGGCAACAGTTCTTTCGTCGTGCGATCCGCTCCTGTCCGTTAAGTGCCACACTGCCATGCACGAATAAGCTAGCT
TCCCCGTAGACACTAACACTACCGTCAATGAACAATACCGTTCTGCTTATCGCACATCGATCGCGCTCTTCCCATCGTCG
CAGGAGAAGGAGCTTTCCGGCCGCCGACCACCATGAACTTGCTGTCAGCTCTCGACGACGCGCCGGCCGAGGAGCGCAGC
GAGAAGGCCACCGCCGGGGAGCCAAAACACCAGGACAAATGCACCGGCGGAAATCCAAGGTATATATATTGATGACGAAG
GAAGGAAGGAAGCAAGGTCGTGTGCCTTGAATTAATTAACCGGAAGAACAACACTGCACGCTCGGGTTCAAAAGAAAAAG
GAAGGAATAACAGCAAGGTGTGAGTGTGACATGTCGTTCTTGTCGAATACAGTTTAAAAAGAGAGGCGTTTTGGTTCCTG
TAGGGAGGAGGCAGCCGGAGAGGAGGAGGAGGAGGCGCAGCAGCTGACCATCTTCTACGGCGGCAGAGTGGTCGTCTTCG
ACATGTTCCCCTCCGCCAAGGTCGAGGACCTGCTGCAGATCATGAGCCCTGGCGGCGACGGCGTGGACAGGGCAGGCGGT
GCCACTGTCCCTACGCGGAGCCTGCATAGGCCTTCACACGACAGCCTCTCTGGTAAAGCCTGTCTGCTCTTGCAGTTCTA
CCATGGCGTCGGCAACGGCAACACGAGTACTTGTCGGTTACACAAGTGCCTTTTTTTTTGTGCTGTGCGACAGATCTGCC
GATTGCGAGGAGGAATTCGCTCCACAGGTTTCTTGAGAAGAGAAAGGACAGGTAACGGAGTGGTCGTTCATATGCTTTTC
TTTTCTCTCGGGTTTTCAGTGATGGAGAACAGTTGCATTCAGAATCAAGTCCTGACATACTTTGTGTGTGTGTGTGTTTT
CATTCGAGTTCCAGGATAACTGCAAAGGCGCCATACCAACAAGTCAACAGCTCTGTTGTTGGCGTCGAGGCGTCCAAGCA
GGCGGCTGGAGCTGGGGTGGAAAAACCTTGGCTGGGGCTGGGCCAAGAAGCGACAACAGCGAAGCTGGAGATGTGATGAA
TGTTGCCCGACAAGAAGGTGCAGTGCACGAGATTCACGGATGGTGCAGGTCGTAGCTTCAGGTTAAGGTGAAGGATAACT
TACCGTCGGATACTCTACTCACTTTACTGCTCTACTACTACTACTGCTGGTGACGAAGTTAGCAAGAAACGTTCTTCTGC
TACCGGTGGGTCGGTCGGTCAGCCGTTCGTTGGCGAGATTGTTTGCTGCTGGAGACGGACGTGCTCGCAGCAGTTTCGCT
GGTCCGTGACCGCGCCATATGTTGCCACGAATGGTTGGTTGTTCGGTACGTTGAGGAACGATGTGTATGTAGTGTGCACG
TTTCCGGTGCATCTTGTTCTCCTCAAGCCTGTCCTGTATGTTTACCCCCCTGCACCCCCTCCTCCCTCTTTGTAGAGAGA
GGGTCTGTATGTTCAGTGGAAGATGGTTGTTCTATTTGATTTTTATTTTATGGACATGTATGCTCGGTGATGTGTTGTGG
TATGCTGTATATCGGGTATATCATAATTACGAGTATCCAAGTTATATCCCTAAACATGCTTAACGTCTAGCTACCAAACA

ATC
>GRMZM2G005954_T01 cDNA
CCCGTCGCTGCCATCGTCGCGTCCTCTCATCGTCCCCGGCGCGCGCGCCCCTCCTCCCAGATCACGTGTCGCTACCACGC
CTTCCCGTCGGGGCGTCCCTCTCGCTCGCCTGCCCCTCCTCCTCCTCCTCCTCCTCCTCCGTCCGATCACGTCGCCA
CCGCGGCCCCTCGGACCCGTCGCTCTCCCACATTCCCGTTCCCCGAGGACCACCATCCTCCGTTCCGGCGGCAGCCACGT
TTTTCATGCGAGGCGAAAGGCGGCCTAGTCGCAAAGGCCATTTTCTCCGTGGCCCCCCCCACACTCGCCGTTTCCTTGGC
ACACGCGCTATTATTAATAAAGGCGCAAAGGCCATTCGCGAGGCGAGGGGAGGAGAAGTGTACTACGCGAGAGCTTGCCG
GAGGCTCTGGATCCCTGTGATAATAATAAACTGATATAAGCAGAGGAAGCGGGGAGGAAGCTGCGAGTTGGAGGGGGCCG
GGAGAGGCGTCTCTGGTTATTGCTTGTCCCTGCTTCCCGGTGAGCTGCTGGGAGTGGACGGGCCGGGAAGGAGACGAGAA
GATAAGGGCGCCGCGCGCGGCCATGGCTGGAAGTGCGCCGGCGACGGCGATGGACAAGACCAGCTTTGCCACCACGTGCA
GCCTGCTGAGCCAGTACGTCAAGGAGAAGAAGGGCGGCCTGCTGCAGGGCCTTGGTGCCCTCGCCATGGCGCCGGCAGCT
GGAGAAGGAGCTTTCCGGCCGCCGACCACCATGAACTTGCTGTCAGCTCTCGACGACGCGCCGGCCGAGGAGCGCAGCGA
GAAGGCCACCGCCGGGGAGCCAAAACACCAGGACAAATGCACCGGCGGAAATCCAAGGGAGGAGGCAGCCGGAGAGGAGG
AGGAGGAGGCGCAGCAGCTGACCATCTTCTACGGCGGCAGAGTGGTCGTCTTCGACATGTTCCCCTCCGCCAAGGTCGAG
GACCTGCTGCAGATCATGAGCCCTGGCGGCGACGGCGTGGACAGGGCAGGCGGTGCCACTGTCCCTACGCGGAGCCTGCA
TAGGCCTTCACACGACAGCCTCTCTGATCTGCCGATTGCGAGGAGGAATTCGCTCCACAGGTTTCTTGAGAAGAGAAAGG
ACAGGATAACTGCAAAGGCGCCATACCAACAAGTCAACAGCTCTGTTGTTGGCGTCGAGGCGTCCAAGCAGGCGGCTGGA
GCTGGGGTGGAAAAACCTTGGCTGGGGCTGGGCCAAGAAGCGACAACAGCGAAGCTGGAGATGTGATGAATGTTGCCCGA
CAAGAAGGTGCAGTGCACGAGATTCACGGATGGTGCAGGTCGTAGCTTCAGGTTAAGGTGAAGGATAACTTACCGTCGGA
TACTCTACTCACTTTACTGCTCTACTACTACTACTGCTGGTGACGAAGTTAGCAAGAAACGTTCTTCTGCTACCGGTGGG
TCGGTCGGTCAGCCGTTCGTTGGCGAGATTGTTTGCTGCTGGAGACGGACGTGCTCGCAGCAGTTTCGCTGGTCCGTGAC
CGCGCCATATGTTGCCACGAATGGTTGGTTGTTCGGTACGTTGAGGAACGATGTGTATGTAGTGTGCACGTTTCCGGTGC
ATCTTGTTCTCCTCAAGCCTGTCCTGTATGTTTACCCCCCTGCACCCCCTCCTCCCTCTTTGTAGAGAGAGGGTCTGTAT
GTTCAGTGGAAGATGGTTGTTCTATTTGATTTTTATTTTATGGACATGTATGCTCGGTGATGTGTTGTGGTATGCTGTAT
ATCGGGTATATCATAATTACGAGTATCCAAGTTATATCCCTAAACATGCTTAACGTCTAGCTACCAAACAATC

>GRMZM2G005954_P01 peptide
MAGSAPATAMDKTSFATTCSLLSQYVKEKKGGLLQGLGALAMAPAAGEGAFRPPTTMNLLSALDDAPAEERSEKATAGEP
KHQDKCTGGNPREEAAGEEEEEAQQLTIFYGGRVVVFDMFPSAKVEDLLQIMSPGGDGVDRAGGATVPTRSLHRPSHDSL
SDLPIARRNSLHRFLEKRKDRITAKAPYQQVNSSVVGVEASKQAAGAGVEKPWLGLGQEATTAKLEM
>GRMZM2G005954_T02 cDNA
Chr2: 184649194..184651204
AGGGGAGGAGAAGTGTACTACGCGAGAGCTTGCCGGAGGCTCTGGATCCCTGTGATAATAATAAACTGATATAAGCAGAG
GAAGCGGGGAGGAAGCTGCGAGTTGGAGGGGGCCGGGAGAGGCGTCTCTGGTTATTGCTTGTCCCTGCTTCCCGGTGAGC
TGCTGGGAGTGGACGGGCCGGGAAGGAGACGAGAAGATAAGGGCGCCGCGCGCGGCCATGGCTGGAAGTGCGCCGGCGAC
GGCGATGGACAAGACCAGCTTTGCCACCACGTGCAGCCTGCTGAGCCAGTACGTCAAGGAGAAGAAGGGCGGCCTGCTGC
AGGGCCTTGGTGCCCTCGCCATGGCGCCGGCAGCTGGTAAGGGCAACAGTTCTTTCGTCGTGCGATCCGCTCCTGTCCGT
TAAGTGCCACACTGCCATGCACGAATAAGCTAGCTTCCCCGTAGACACTAACACTACCGTCAATGAACAATACCGTTCTG
CTTATCGCACATCGATCGCGCTCTTCCCATCGTCGCAGGAGAAGGAGCTTTCCGGCCGCCGACCACCATGAACTTGCTGT
CAGCTCTCGACGACGCGCCGGCCGAGGAGCGCAGCGAGAAGGCCACCGCCGGGGAGCCAAAACACCAGGACAAATGCACC
GGCGGAAATCCAAGGGAGGAGGCAGCCGGAGAGGAGGAGGAGGAGGCGCAGCAGCTGACCATCTTCTACGGCGGCAGAGT
GGTCGTCTTCGACATGTTCCCCTCCGCCAAGGTCGAGGACCTGCTGCAGATCATGAGCCCTGGCGGCGACGGCGTGGACA
GGGCAGGCGGTGCCACTGTCCCTACGCGGAGCCTGCATAGGCCTTCACACGACAGCCTCTCTGATCTGCCGATTGCGAGG
AGGAATTCGCTCCACAGGTTTCTTGAGAAGAGAAAGGACAGGATAACTGCAAAGGCGCCATACCAACAAGTCAACAGCTC

TGTTGTTGGCGTCGAGGCGTCCAAGCAGGCGGCTGGAGCTGGGGTGGAAAAACCTTGGCTGGGGCTGGGCCAAGAAGCGA
CAACAGCGAAGCTGGAGATGTGATGAATGTTGCCCGACAAGAAGGTGCAGTGCACGAGATTCACGGATGGTGCAGGTCGT
AGCTTCAGGTTAAGGTGAAGGATAACTTACCGTCGGATACTCTACTCACTTTACTGCTCTACTACTACTACTGCTGGTGA
CGAAGTTAGCAAGAAACGTTCTTCTGCTACCGGTGGGTCGGTCGGTCAGCCGTTCGTTGGCGAGATTGTTTGCTGCTGGA
GACGGACGTGCTCGCAGCAGTTTCGCTGGTCCGTGACCGCGCCATATGTTGCCACGAATGGTTGGTTGTTCGGTACGTTG
AGGAACGATGTGTATGTAGTGTGCACGTTTCCGGTGCATCTTGTTCTCCTCAAGCCTGTCCTGTATGTTTACCCCCCTGC
ACCCCCTCCTCCCTCTTTGTAGAGAGAGGGTCTGTATGTTCAGTGGAAGATGGTTGTTCTATTTGATTTTTATTTTATGG
ACATGTATGCTCGGTGATGTGTTGTGGTATGCTGTATATCGGGTATATCATAATTACGAGTATCCAAGTTATATCCCTAA
ACA
>GRMZM2G005954_P02 peptide
MNLLSALDDAPAEERSEKATAGEPKHQDKCTGGNPREEAAGEEEEEAQQLTIFYGGRVVVFDMFPSAKVEDLLQIMSPGG
DGVDRAGGATVPTRSLHRPSHDSLSDLPIARRNSLHRFLEKRKDRITAKAPYQQVNSSVVGVEASKQAAGAGVEKPWLGL
GQEATTAKLEM

AGPv4:
Zm00001d005813 (zim13 - ZIM-transcription factor 13)
Chr2: 189505960..189508316

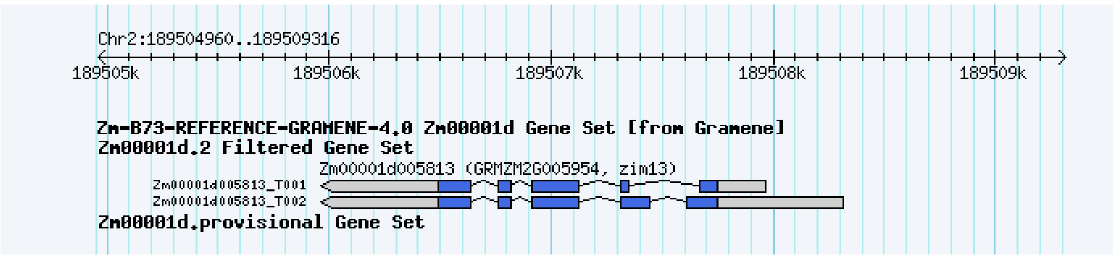

>Zm00001d005813 Genomic DNA
ATCGTCGCGTCCTCTCATCGTCCCCGGCGCGCGCGCCCCTCCTCCCAGATCACGTGTCGCTACCACGCCTTCCCGTCGGG
GCGTCCCTCTCGCTCGCCTGCCCCTCCTCCTCCTCCTCCTCCTCCTCCTCCGTCCGATCACGTCGCCACCGCGGCCCCTC
GGACCCGTCGCTCTCCCACATTCCCGTTCCCCGAGGACCACCATCCTCCGTTCCGGCGGCAGCCACGTTTTTCATGCGAG
GCGAAAGGCGGCCTAGTCGCAAAGGCCATTTTCTCCGTGGCCCCCCCCACACTCGCCGTTTCCTTGGCACACGCGCTATT
ATTAATAAAGGCGCAAAGGCCATTCGCGAGGCGAGGGGAGGAGAAGTGTACTACGCGAGAGCTTGCCGGAGGCTCTGGAT
CCCTGTGATAATAATAAACTGATATAAGCAGAGGAAGCGGGGAGGAAGCTGCGAGTTGGAGGGGGCCGGGAGAGGCGTCT
CTGGTTATTGCTTGTCCCTGCTTCCCGGTGAGCTGCTGGGAGTGGACGGGCCGGGAAGGAGACGAGAAGATAAGGGCGCC
GCGCGCGGCCATGGCTGGAAGTGCGCCGGCGACGGCGATGGACAAGACCAGCTTTGCCACCACGTGCAGCCTGCTGAGCC
AGTACGTCAAGGAGAAGAAGGGCGGCCTGCTGCAGGGCCTTGGTGCCCTCGCCATGGCGCCGGCAGCTGGTAAGGGCAAC
AGTTCTTTCGTCGTGCGATCCGCTCCTGTCCGTTAAGTGCCACACTGCCATGCACGAATAAGCTAGCTTCCCCGTAGACA
CTAACACTACCGTCAATGAACAATACCGTTCTGCTTATCGCACATCGATCGCGCTCTTCCCATCGTCGCAGGAGAAGGAG
CTTTCCGGCCGCCGACCACCATGAACTTGCTGTCAGCTCTCGACGACGCGCCGGCCGAGGAGCGCAGCGAGAAGGCCACC
GCCGGGGAGCCAAAACACCAGGACAAATGCACCGGCGGAAATCCAAGGTATATATATTGATGACGAAGGAAGGAAGGAAG
CAAGGTCGTGTGCCTTGAATTAATTAACCGGAAGAACAACACTGCACGCTCGGGTTCAAAAGAAAAAGGAAGGAATAACA
GCAAGGTGTGAGTGTGACATGTCGTTCTTGTCGAATACAGTTTAAAAAGAGAGGCGTTTTGGTTCCTGTAGGGAGGAGGC
AGCCGGAGAGGAGGAGGAGGAGGCGCAGCAGCTGACCATCTTCTACGGCGGCAGAGTGGTCGTCTTCGACATGTTCCCCT
CCGCCAAGGTCGAGGACCTGCTGCAGATCATGAGCCCTGGCGGCGACGGCGTGGACAGGGCAGGCGGTGCCACTGTCCCT
ACGCGGAGCCTGCATAGGCCTTCACACGACAGCCTCTCTGGTAAAGCCTGTCTGCTCTTGCAGTTCTACCATGGCGTCGG
CAACGGCAACACGAGTACTTGTCGGTTACACAAGTGCCTTTTTTTTTGTGCTGTGCGACAGATCTGCCGATTGCGAGGAG
GAATTCGCTCCACAGGTTTCTTGAGAAGAGAAAGGACAGGTAACGGAGTGGTCGTTCATATGCTTTTCTTTTCTCTCGGG

TTTTCAGTGATGGAGAACAGTTGCATTCAGAATCAAGTCCTGACATACTTTGTGTGTGTGTGTGTTTTCATTCGAGTTCC
AGGATAACTGCAAAGGCGCCATACCAACAAGTCAACAGCTCTGTTGTTGGCGTCGAGGCGTCCAAGCAGGCGGCTGGAGC
TGGGGTGGAAAAACCTTGGCTGGGGCTGGGCCAAGAAGCGACAACAGCGAAGCTGGAGATGTGATGAATGTTGCCCGACA
AGAAGGTGCAGTGCACGAGATTCACGGATGGTGCAGGTCGTAGCTTCAGGTTAAGGTGAAGGATAACTTACCGTCGGGTA
CTCTACTCACTTTACTGCTCTACTACTACTACTGCTGGTGACGAAGTTAGCAAGAAACGTTCTTCTGCTACCGGTGGGTC
GGTCGGTCAGCCGTTCGTTGGCGAGATTGTTTGCTGCTGGAGACGGACGTGCTCGCAGCAGTTTCGCTGGTCCGTGACCG
CGCCATATGTTGCCACGAATGGTTGGTTGTTCGGTACGTTGAGGAACGATGTGTATGTAGTGTGCACGTTTCCGGTGCAT
CTTGTTCTCCTCAAGCCTGTCCTGTATGTTTACCCCCCTGCACCCCCTCCTCCCTCTTTGTAGAGAGAGGGTCTGTATGT
TCAGTGGAAGATGGTTGTTCTATTTGATTTTTATTTTATGGACATGTATGCTCGGTGATGTGTTGTGGTATGCTGTATAT
CGGGTATATCATAATTACGAGTATCCAAGTTATATCC
>Zm00001d005813_T001 cDNA
GGGGAGGAGAAGTGTACTACGCGAGAGCTTGCCGGAGGCTCTGGATCCCTGTGATAATAATAAACTGATATAAGCAGAGG
AAGCGGGGAGGAAGCTGCGAGTTGGAGGGGGCCGGGAGAGGCGTCTCTGGTTATTGCTTGTCCCTGCTTCCCGGTGAGCT
GCTGGGAGTGGACGGGCCGGGAAGGAGACGAGAAGATAAGGGCGCCGCGCGCGGCCATGGCTGGAAGTGCGCCGGCGACG
GCGATGGACAAGACCAGCTTTGCCACCACGTGCAGCCTGCTGAGCCAGTACGTCAAGGAGCCAAAACACCAGGACAAATG
CACCGGCGGAAATCCAAGGGAGGAGGCAGCCGGAGAGGAGGAGGAGGAGGCGCAGCAGCTGACCATCTTCTACGGCGGCA
GAGTGGTCGTCTTCGACATGTTCCCCTCCGCCAAGGTCGAGGACCTGCTGCAGATCATGAGCCCTGGCGGCGACGGCGTG
GACAGGGCAGGCGGTGCCACTGTCCCTACGCGGAGCCTGCATAGGCCTTCACACGACAGCCTCTCTGATCTGCCGATTGC
GAGGAGGAATTCGCTCCACAGGTTTCTTGAGAAGAGAAAGGACAGGATAACTGCAAAGGCGCCATACCAACAAGTCAACA
GCTCTGTTGTTGGCGTCGAGGCGTCCAAGCAGGCGGCTGGAGCTGGGGTGGAAAAACCTTGGCTGGGGCTGGGCCAAGAA
GCGACAACAGCGAAGCTGGAGATGTGATGAATGTTGCCCGACAAGAAGGTGCAGTGCACGAGATTCACGGATGGTGCAGG
TCGTAGCTTCAGGTTAAGGTGAAGGATAACTTACCGTCGGGTACTCTACTCACTTTACTGCTCTACTACTACTACTGCTG
GTGACGAAGTTAGCAAGAAACGTTCTTCTGCTACCGGTGGGTCGGTCGGTCAGCCGTTCGTTGGCGAGATTGTTTGCTGC
TGGAGACGGACGTGCTCGCAGCAGTTTCGCTGGTCCGTGACCGCGCCATATGTTGCCACGAATGGTTGGTTGTTCGGTAC
GTTGAGGAACGATGTGTATGTAGTGTGCACGTTTCCGGTGCATCTTGTTCTCCTCAAGCCTGTCCTGTATGTTTACCCCC
CTGCACCCCCTCCTCCCTCTTTGTAGAGAGAGGGTCTGTATGTTCAGTGGAAGATGGTTGTTCTATTTGATTTTTATTTT
ATGGACATGTATGCTCGGTGATGTGTTGTGGTATGCTGTATATCGGGTATATCATAATTACGAGTATCCAAGTTATATCC

>Zm00001d005813_P001 peptide
MAGSAPATAMDKTSFATTCSLLSQYVKEPKHQDKCTGGNPREEAAGEEEEEAQQLTIFYGGRVVVFDMFPSAKVEDLLQI
MSPGGDGVDRAGGATVPTRSLHRPSHDSLSDLPIARRNSLHRFLEKRKDRITAKAPYQQVNSSVVGVEASKQAAGAGVEK
PWLGLGQEATTAKLEM

>Zm00001d005813_T002 cDNA
ATCGTCGCGTCCTCTCATCGTCCCCGGCGCGCGCGCCCCTCCTCCCAGATCACGTGTCGCTACCACGCCTTCCCGTCGGG
GCGTCCCTCTCGCTCGCCTGCCCCTCCTCCTCCTCCTCCTCCTCCTCCTCCGTCCGATCACGTCGCCACCGCGGCCCCTC
GGACCCGTCGCTCTCCCACATTCCCGTTCCCCGAGGACCACCATCCTCCGTTCCGGCGGCAGCCACGTTTTTCATGCGAG
GCGAAAGGCGGCCTAGTCGCAAAGGCCATTTTCTCCGTGGCCCCCCCCACACTCGCCGTTTCCTTGGCACACGCGCTATT
ATTAATAAAGGCGCAAAGGCCATTCGCGAGGCGAGGGGAGGAGAAGTGTACTACGCGAGAGCTTGCCGGAGGCTCTGGAT
CCCTGTGATAATAATAAACTGATATAAGCAGAGGAAGCGGGGAGGAAGCTGCGAGTTGGAGGGGGCCGGGAGAGGCGTCT
CTGGTTATTGCTTGTCCCTGCTTCCCGGTGAGCTGCTGGGAGTGGACGGGCCGGGAAGGAGACGAGAAGATAAGGGCGCC
GCGCGCGGCCATGGCTGGAAGTGCGCCGGCGACGGCGATGGACAAGACCAGCTTTGCCACCACGTGCAGCCTGCTGAGCC
AGTACGTCAAGGAGAAGAAGGGCGGCCTGCTGCAGGGCCTTGGTGCCCTCGCCATGGCGCCGGCAGCTGGAGAAGGAGCT
TTCCGGCCGCCGACCACCATGAACTTGCTGTCAGCTCTCGACGACGCGCCGGCCGAGGAGCGCAGCGAGAAGGCCACCGC

CGGGGAGCCAAAACACCAGGACAAATGCACCGGCGGAAATCCAAGGGAGGAGGCAGCCGGAGAGGAGGAGGAGGAGGCGC
AGCAGCTGACCATCTTCTACGGCGGCAGAGTGGTCGTCTTCGACATGTTCCCCTCCGCCAAGGTCGAGGACCTGCTGCAG
ATCATGAGCCCTGGCGGCGACGGCGTGGACAGGGCAGGCGGTGCCACTGTCCCTACGCGGAGCCTGCATAGGCCTTCACA
CGACAGCCTCTCTGATCTGCCGATTGCGAGGAGGAATTCGCTCCACAGGTTTCTTGAGAAGAGAAAGGACAGGATAACTG
CAAAGGCGCCATACCAACAAGTCAACAGCTCTGTTGTTGGCGTCGAGGCGTCCAAGCAGGCGGCTGGAGCTGGGGTGGAA
AAACCTTGGCTGGGGCTGGGCCAAGAAGCGACAACAGCGAAGCTGGAGATGTGATGAATGTTGCCCGACAAGAAGGTGCA
GTGCACGAGATTCACGGATGGTGCAGGTCGTAGCTTCAGGTTAAGGTGAAGGATAACTTACCGTCGGGTACTCTACTCAC
TTTACTGCTCTACTACTACTACTGCTGGTGACGAAGTTAGCAAGAAACGTTCTTCTGCTACCGGTGGGTCGGTCGGTCAG
CCGTTCGTTGGCGAGATTGTTTGCTGCTGGAGACGGACGTGCTCGCAGCAGTTTCGCTGGTCCGTGACCGCGCCATATGT
TGCCACGAATGGTTGGTTGTTCGGTACGTTGAGGAACGATGTGTATGTAGTGTGCACGTTTCCGGTGCATCTTGTTCTCC
TCAAGCCTGTCCTGTATGTTTACCCCCCTGCACCCCCTCCTCCCTCTTTGTAGAGAGAGGGTCTGTATGTTCAGTGGAAG
ATGGTTGTTCTATTTGATTTTTATTTTATGGACATGTATGCTCGGTGATGTGTTGTGGTATGCTGTATATCGGTATATC
ATAATTACGAGTATCCAAGTTATATCC

>Zm00001d005813_P002 peptide
MAGSAPATAMDKTSFATTCSLLSQYVKEKKGGLLQGLGALAMAPAAGEGAFRPPTTMNLLSALDDAPAEERSEKATAGEP
KHQDKCTGGNPREEAAGEEEEEAQQLTIFYGGRVVVFDMFPSAKVEDLLQIMSPGGDGVDRAGGATVPTRSLHRPSHDSL
SDLPIARRNSLHRFLEKRKDRITAKAPYQQVNSSVVGVEASKQAAGAGVEKPWLGLGQEATTAKLEM

ZmJAZ12

B73 RefGen_v3:
GRMZM2G101769 (zim12 - ZIM-transcription factor 12)
Chr2: 211875346..211877961

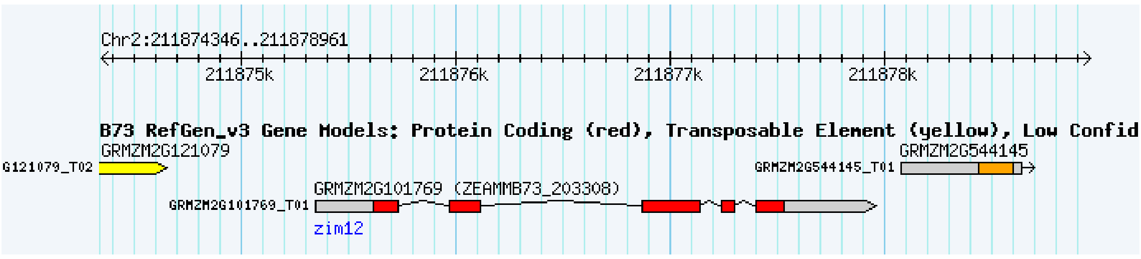

>GRMZM2G101769 Genomic DNA
TTTAGTCGCGGGATTTGGGTGGAAGTGGAAACCGCGCCGCGGGGGTCTTTTAAGCGCGTGGGGGCGCGGCGTGCTCACAC
GAGGCCCGCGCTCACGAGCCAGAGAAGAAGGGAGAGGAGAGGCTAACCACCGCGGCAGCTGCGACCTGCGACCGCGAGGA
GTGGCGCACGACGACCAGCGCGGAGGAGGTGGAGCCGGCGGCGGGGAGATTTGCTCGCCCGGCTGGGGTTGCGGTGGGAG
GAGGGTAGGAGGCAGAGACAACCAGCGATGGCGGCGTCCGCGAGGCCCGGGGAGAGGGCGACCAGCTTCGCCGTCGCGTG
CAGCCTCCTCAGCCGCTTCGTCCGCCAGAACGGCGCCGCGCCCGCCCAGCTAGGCCTCGGGATCAAAGGTGAGCAGCGGA
ATCAGGCGGCGGCGCCGGCCAGGCCTGCCCTGTTGTCTGTTGAGATCGGGATCCTTCTGGGTGCGCGGGGGTGGGGGTCG
GTCCAGTTTCAATTCCTGTGGGGGACGTGTTCGCCTGGTGCGAGATCTGGGCGGGCCCTTTGTTATATCCGGTTTGGAAA
CGACGTTTTTGGCCTCCGAATTCGCACTCCCTTATCCGTCTCTCTCCCTCTTTTTTTCCTTCTCAGGCGAGGTCGAGCA
GCAAAGGACGCCGGCGACAATTAACTTGCTCCCCGGAGCGGACGGCGAGGAGACCGAGAGGAGGAAGGAGACCATGGAGC
TTTTCCCGCAGAGCGCCGGGTTCGGTGTCAAGGATGCCGCTGCTGCCCCTAGGTGCGTACTGCTGATTCACATCATGTAA
ATCTCATTTCGGTTTTGGTTCGTGCAACACTTGTTCAGTAGTACTGTAGTAGCGCAGCTCGAACCTAGCTGTCTTAGGTC
TCATGGACAGAAGGCCAAGCGATTTGAGTTTGGAAACATAGTGATGTGAGTTTAATGAGTGTTCATAGGTGGACCGTGTT
ATAGGGTGGTCTAAAATCTACTTGAGCCGGATGGTAAACAACAGAGTATGTGTAAAGCATACTTGTAGTTCAGTCTGTTG

ATATTCGGTCAAAGCTGTATCTTTGTAGTTCAGTCTGTGCTGATATTCCCTGGTCAAAGCTATATCTTTGTAGTCTTGAG
AAGTTATTGCCTTCATCGAAACACTGTCTTATGCAAGGAGCAACAAAACGTTTCCTTTTATCAGGCAAAGCAAGTAACGT
GCTCGAAGTTTTTAGATTGCACCTAGGGCTCGTTTGAGAGTCATAAAACCGGATGGGCTAGATTCTCCTTATTTAATAAG
AAGTAAGAAGGGGAATCTAGCCCCTCAAACCCCTCCGGTTTTCTTGCTCCCAAACTAGTTCCTAATGTTTACCATTAACT
ACCTTGCTGGTAACAACATGAAAATGGGTTAACCTAAGATATATGTCAGAGATGAGGTCTTCTAACCAGAAAAAGAAAAC
AGAGTAGACAGTTGATTTGTTGAGCTTAAGTAGTGTTCTACATGCACTGTTCTGTTCTTAGTGTTCTTCTCTGTAATATT
TGTCAGAGAGCAAGAAAATAAAGAGAAGCCTAAACAGCTCACAATCTTCTATGGCGGGAAGGTGCTGGTGTTTGACGATT
TCCCTGCCGACAAGGCAAAGGACCTGATGCAGCTGGCCAGCAAGGGCGGCCCTGTGGTACAGAACGTTGTTTTGCCTCAA
CCCTCTGCACCTGCTGCTGCTGTCACCGACAAGGCCGTGCCGGTCCCCGTCATCAGCTTGCCTGCTGCTCAGGCTGATGC
TAAGAAGCCTACTCGCACAAATGCCTCTGGTAAATACTCCTCTCTCTGTGTTTCTATCCTTGGTTGAAGTTTTAAAATGT
GATTGGTGAACTTTGCCACACCGATTATTAATCAAGACATGTCTTCTCTTTTGTAGATATGCCAATTATGAGGAAGGCTT
CTCTTCACCGCTTCCTTGAGAAGAGAAAGGACCGGTAAGTTGTGGTTGTTATTTTCAGTGTGGCGATTTGTTTTTTTTCT
CTCTCTCTCTCTCAGTGTTCTGTTTGGAACTAATTTGTGCCCTTTTGATGTTCTTCAGTCTCAATGCAAATGCACCATAC
CAAACTTCTCCTTCAGACGCTGCACCAGTCAAGAAGGAGCCCGAGAGCCAGGCATGGCTCGGATTAGGACCGAATGCCGT
CAAGTCCAACCTGAACCTGAGCTAGCACACAACGACACAGCCAAATAAGACGGCACAGCAAACCTCACCAAAGCCTTTGC
AGGACGATCTGAAAATATGCTTTGCCACTAGAGTACCGAAACCATAAATATCCTCTCGTTATCGTGTTTTAGTGTTCTGT
TTAGGCCGTGGTCCTACGCCCTCTTGTCACATAGTTCTGGATGTAAAGATAAGTAGCCAGCCATGACCGAGAGAAGGTTT
TTTTTGGGGGGTGTTAACACACGGCCACTCTGAATTGGCCATGCTAAGGAAAATGATAGTAGTGCAAAGAGAAGTATATG
TCTTTTTCTTTTTCTGAAAGCCCAGATCGTTTGGCTCTAGCCTTGATGTATGCAGAACAAGTGATTGTAATGGATTCTGA
GTATCCTCATCGTCTTATTGTTGCTTCACCAACCCAAGTTTGTTTGGGTTCTTGCC
>GRMZM2G101769_T01 cDNA
TTTAGTCGCGGGATTTGGGTGGAAGTGGAAACCGCGCCGCGGGGGTCTTTTAAGCGCGTGGGGGCGCGGCGTGCTCACAC
GAGGCCCGCGCTCACGAGCCAGAGAAGAAGGGAGAGGAGAGGCTAACCACCGCGGCAGCTGCGACCTGCGACCGCGAGGA
GTGGCGCACGACGACCAGCGCGGAGGAGGTGGAGCCGGCGGCGGGGAGATTTGCTCGCCCGGCTGGGGTTGCGGTGGGAG
GAGGGTAGGAGGCAGAGACAACCAGCGATGGCGGCGTCCGCGAGGCCCGGGGAGAGGGCGACCAGCTTCGCCGTCGCGTG
CAGCCTCCTCAGCCGCTTCGTCCGCCAGAACGGCGCCGCGCCCGCCCAGCTAGGCCTCGGGATCAAAGGCGAGGTCGAGC
AGCAAAGGACGCCGGCGACAATTAACTTGCTCCCCGGAGCGGACGGCGAGGAGACCGAGAGGAGGAAGGAGACCATGGAG
CTTTTCCCGCAGAGCGCCGGGTTCGGTGTCAAGGATGCCGCTGCTGCCCCTAGAGAGCAAGAAAATAAAGAGAAGCCTAA
ACAGCTCACAATCTTCTATGGCGGGAAGGTGCTGGTGTTTGACGATTTCCCTGCCGACAAGGCAAAGGACCTGATGCAGC
TGGCCAGCAAGGGCGGCCCTGTGGTACAGAACGTTGTTTTGCCTCAACCCTCTGCACCTGCTGCTGCTGTCACCGACAAG
GCCGTGCCGGTCCCCGTCATCAGCTTGCCTGCTGCTCAGGCTGATGCTAAGAAGCCTACTCGCACAAATGCCTCTGATAT
GCCAATTATGAGGAAGGCTTCTCTTCACCGCTTCCTTGAGAAGAGAAAGGACCGTCTCAATGCAAATGCACCATACCAAA
CTTCTCCTTCAGACGCTGCACCAGTCAAGAAGGAGCCCGAGAGCCAGGCATGGCTCGGATTAGGACCGAATGCCGTCAAG
TCCAACCTGAACCTGAGCTAGCACACAACGACACAGCCAAATAAGACGGCACAGCAAACCTCACCAAAGCCTTTGCAGGA
CGATCTGAAAATATGCTTTGCCACTAGAGTACCGAAACCATAAATATCCTCTCGTTATCGTGTTTTAGTGTTCTGTTTAG
GCCGTGGTCCTACGCCCTCTTGTCACATAGTTCTGGATGTAAAGATAAGTAGCCAGCCATGACCGAGAGAAGGTTTTTTT
TGGGGGGTGTTAACACACGGCCACTCTGAATTGGCCATGCTAAGGAAAATGATAGTAGTGCAAAGAGAAGTATATGTCTT
TTTCTTTTTCTGAAAGCCCAGATCGTTTGGCTCTAGCCTTGATGTATGCAGAACAAGTGATTGTAATGGATTCTGAGTAT
CCTCATCGTCTTATTGTTGCTTCACCAACCCAAGTTTGTTTGGGTTCTTGCC

>GRMZM2G101769_P01 peptide
MAASARPGERATSFAVACSLLSRFVRQNGAAPAQLGLGIKGEVEQQRTPATINLLPGADGEETERRKETMELFPQSAGFG
VKDAAAAPREQENKEKPKQLTIFYGGKVLVFDDFPADKAKDLMQLASKGGPVVQNVVLPQPSAPAAAVTDKAVPVPVISL
PAAQADAKKPTRTNASDMPIMRKASLHRFLEKRKDRLNANAPYQTSPSDAAPVKKEPESQAWLGLGPNAVKSNLNLS

AGPv4:

Zm00001d006860 ([zim12 - ZIM-transcription factor 12])

Chr2: 218018545..218021082

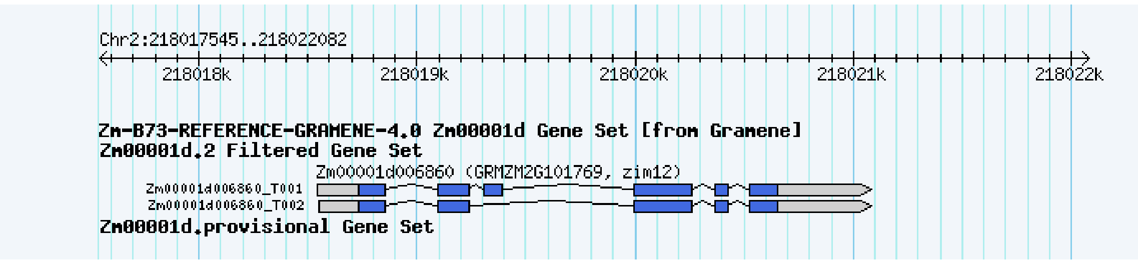

>Zm00001d006860 Genomic DNA

ACACGAGGCCCGCGCTCACGAGCCAGAGAAGAAGGGAGAGGAGAGGCTAACCACCGCGGCAGCTGCGACCTGCGACCGCG
AGGAGTGGCGCACGACGACCAGCGCGGAGGAGGTGGAGCCGGCGGCGGGGAGATTTGCTCGCCCGGCTGGGGTTGCGGTG
GGAGGAGGGTAGGAGGCAGAGACAACCAGCGATGGCGGCGTCCGCGAGGCCCGGGGAGAGGGCGACCAGCTTCGCCGTCG
CGTGCAGCCTCCTCAGCCGCTTCGTCCGCCAGAACGGCGCCGCGCCCGCCCAGCTAGGCCTCGGGATCAAAGGTGAGCAG
CGGAATCAGGCGGCGGCGCCGGCCAGGCCTGCCCTGTTGTCTGTTGAGATCGGGATCCTTCTGGGTGCGCGGGGGTGGGG
GTCGGTCCAGTTTCAATTCCTGTGGGGGACGTGTTCGCCTGGTGCGAGATCTGGGCGGGCCCTTTGTTATATCCGGTTTG
GAAACGACGTTTTTGGCCTCCGAATTCGCACTCCCTTATCCGTCTCTCTCCCTCTTTTTTTTCCTTCTCAGGCGAGGTCG
AGCAGCAAAGGACGCCGGCGACAATTAACTTGCTCCCCGGAGCGGACGGCGAGGAGACCGAGAGGAGGAAGGAGACCATG
GAGCTTTTCCCGCAGAGCGCCGGGTTCGGTGTCAAGGATGCCGCTGCTGCCCCTAGGTGCGTACTGCTGATTCACATCAT
GTAAATCTCATTTCGGTTTTGGTTCGTGCAACACTTGTTCAGTAGTACTGTAGTAGCGCAGCTCGAACCTAGCTGTCTTA
GGTCTCATGGACAGAAGGCCAAGCGATTTGAGTTTGGAAACATAGTGATGTGAGTTTAATGAGTGTTCATAGGTGGACCG
TGTTATAGGGTGGTCTAAAATCTACTTGAGCCGGATGGTAAACAACAGAGTATGTGTAAAGCATACTTGTAGTTCAGTCT
GTTGATATTCGGTCAAAGCTGTATCTTTGTAGTTCAGTCTGTGCTGATATTCCCTGGTCAAAGCTATATCTTTGTAGTCT
TGAGAAGTTATTGCCTTCATCGAAACACTGTCTTATGCAAGGAGCAACAAAACGTTTCCTTTTATCAGGCAAAGCAAGTA
ACGTGCTCGAAGTTTTTAGATTGCACCTAGGGCTCGTTTGAGAGTCATAAAACCGGATGGGCTAGATTCTCCTTATTTAA
TAAGAAGTAAGAAGGGGAATCTAGCCCCTCAAACCCCTCCGGTTTTCTTGCTCCCAAACTAGTTCCTAATGTTTACCATT
AACTACCTTGCTGGTAACAACATGAAAATGGGTTAACCTAAGATATATGTCAGAGATGAGGTCTTCTAACCAGAAAAAGA
AAACAGAGTAGACAGTTGATTTGTTGAGCTTAAGTAGTGTTCTACATGCACTGTTCTGTTCTTAGTGTTCTTCTCTGTAA
TATTTGTCAGAGAGCAAGAAAATAAAGAGAAGCCTAAACAGCTCACAATCTTCTATGGCGGGAAGGTGCTGGTGTTTGAC
GATTTCCCTGCCGACAAGGCAAAGGACCTGATGCAGCTGGCCAGCAAGGGCGGCCCTGTGGTACAGAACGTTGTTTTGCC
TCAACCCTCTGCACCTGCTGCTGCTGTCACCGACAAGGCCGTGCCGGTCCCCGTCATCAGCTTGCCTGCTGCTCAGGCTG
ATGCTAAGAAGCCTACTCGCACAAATGCCTCTGGTAAATACTCCTCTCTCTGTGTTTCTATCCTTGGTTGAAGTTTTAAA
ATGTGATTGGTGAACTTTGCCACACCGATTATTAATCAAGACATGTCTTCTCTTTTGTAGATATGCCAATTATGAGGAAG
GCTTCTCTTCACCGCTTCCTTGAGAAGAGAAAGGACCGGTAAGTTGTGGTTGTTATTTCAGTGTGGCGATTTGTTTTTT
TTCTCTCTCTCTCTCAGTGTTCTGTTTGGAACTAATTTGTGCCCTTTTGATGTTCTTCAGTCTCAATGCAAATGCACC
ATACCAAACTTCTCCTTCAGACGCTGCACCAGTCAAGAAGGAGCCCGAGAGCCAGGCATGGCTCGGATTAGGACCGAATG
CCGTCAAGTCCAACCTGAACCTGAGCTAGCACACAACGACACAGCCAAATAAGACGGCACAGCAAACCTCACCAAAGCCT
TTGCAGGACGATCTGAAAATATGCTTTGCCACTAGAGTACCGAAACCATAAATATCCTCTCGTTATCGTGTTTTAGTGTT
CTGTTTAGGCCGTGGTCCTACGCCCTCTTGTCACATAGTTCTGGATGTAAAGATAAGTAGCCAGCCATGACCGAGAGAAG
GTTTTTTTGGGGGGTGTTAACACACGGCCACTCTGAATTGGCCATGCTAAGGAAAATGATAGTAGTGCAAAGAGAAGTA
TATGTCTTTTTCTTTTTCTGAAAGCCCAGATCGTTTGGCTCTAGCCTTGATGTATGCAGAACAAGTGATTGTAATGGATT
CTGAGTATCCTCATCGTCTTATTGTTGCTTCACCAACCCAAGTTTGTTTGGGTTCTTG

>Zm00001d006860_T001 cDNA
ACACGAGGCCCGCGCTCACGAGCCAGAGAAGAAGGGAGAGGAGAGGCTAACCACCGCGGCAGCTGCGACCTGCGACCGCG
AGGAGTGGCGCACGACGACCAGCGCGGAGGAGGTGGAGCCGGCGGCGGGGAGATTTGCTCGCCCGGCTGGGGTTGCGGTG
GGAGGAGGGTAGGAGGCAGAGACAACCAGCGATGGCGGCGTCCGCGAGGCCCGGGGAGAGGGCGACCAGCTTCGCCGTCG
CGTGCAGCCTCCTCAGCCGCTTCGTCCGCCAGAACGGCGCCGCGCCCGCCCAGCTAGGCCTCGGGATCAAAGGCGAGGTC
GAGCAGCAAAGGACGCCGGCGACAATTAACTTGCTCCCCGGAGCGGACGGCGAGGAGACCGAGAGGAGGAAGGAGACCAT
GGAGCTTTTCCCGCAGAGCGCCGGGTTCGGTGTCAAGGATGCCGCTGCTGCCCCTAGTAGTACTGTAGTAGCGCAGCTCG
AACCTAGCTGTCTTAGGTCTCATGGACAGAAGGCCAAGCGATTTGAGTTTGGAAACATAGTGATAGAGCAAGAAAATAAA
GAGAAGCCTAAACAGCTCACAATCTTCTATGGCGGGAAGGTGCTGGTGTTTGACGATTTCCCTGCCGACAAGGCAAAGGA
CCTGATGCAGCTGGCCAGCAAGGGCGGCCCTGTGGTACAGAACGTTGTTTTGCCTCAACCCTCTGCACCTGCTGCTGCTG
TCACCGACAAGGCCGTGCCGGTCCCCGTCATCAGCTTGCCTGCTGCTCAGGCTGATGCTAAGAAGCCTACTCGCACAAAT
GCCTCTGATATGCCAATTATGAGGAAGGCTTCTCTTCACCGCTTCCTTGAGAAGAGAAAGGACCGTCTCAATGCAAATGC
ACCATACCAAACTTCTCCTTCAGACGCTGCACCAGTCAAGAAGGAGCCCGAGAGCCAGGCATGGCTCGGATTAGGACCGA
ATGCCGTCAAGTCCAACCTGAACCTGAGCTAGCACACAACGACACAGCCAAATAAGACGGCACAGCAAACCTCACCAAAG
CCTTTGCAGGACGATCTGAAAATATGCTTTGCCACTAGAGTACCGAAACCATAAATATCCTCTCGTTATCGTGTTTTAGT
GTTCTGTTTAGGCCGTGGTCCTACGCCCTCTTGTCACATAGTTCTGGATGTAAAGATAAGTAGCCAGCCATGACCGAGAG
AAGGTTTTTTTTGGGGGGTGTTAACACACGGCCACTCTGAATTGGCCATGCTAAGGAAAATGATAGTAGTGCAAAGAGAA
GTATATGTCTTTTTCTTTTTCTGAAAGCCCAGATCGTTTGGCTCTAGCCTTGATGTATGCAGAACAAGTGATTGTAATGG
ATTCTGAGTATCCTCATCGTCTTATTGTTGCTTCACCAACCCAAGTTTGTTTGGGTTCTTG

>Zm00001d006860_P001 peptide
MAASARPGERATSFAVACSLLSRFVRQNGAAPAQLGLGIKGEVEQQRTPATINLLPGADGEETERRKETMELFPQSAGFG
VKDAAAAPSSTVVAQLEPSCLRSHGQKAKRFEFGNIVIEQENKEKPKQLTIFYGGKVLVFDDFPADKAKDLMQLASKGGP
VVQNVVLPQPSAPAAAVTDKAVPVPVISLPAAQADAKKPTRTNASDMPIMRKASLHRFLEKRKDRLNANAPYQTSPSDAA
PVKKEPESQAWLGLGPNAVKSNLNLS

>Zm00001d006860_T002 cDNA
Chr2: 218018549..218021082
GAGGCCCGCGCTCACGAGCCAGAGAAGAAGGGAGAGGAGAGGCTAACCACCGCGGCAGCTGCGACCTGCGACCGCGAGGA
GTGGCGCACGACGACCAGCGCGGAGGAGGTGGAGCCGGCGGCGGGGAGATTTGCTCGCCCGGCTGGGGTTGCGGTGGGAG
GAGGGTAGGAGGCAGAGACAACCAGCGATGGCGGCGTCCGCGAGGCCCGGGGAGAGGGCGACCAGCTTCGCCGTCGCGTG
CAGCCTCCTCAGCCGCTTCGTCCGCCAGAACGGCGCCGCGCCCGCCCAGCTAGGCCTCGGGATCAAAGGCGAGGTCGAGC
AGCAAAGGACGCCGGCGACAATTAACTTGCTCCCCGGAGCGGACGGCGAGGAGACCGAGAGGAGGAAGGAGACCATGGAG
CTTTTCCCGCAGAGCGCCGGGTTCGGTGTCAAGGATGCCGCTGCTGCCCCTAGAGAGCAAGAAAATAAAGAGAAGCCTAA
ACAGCTCACAATCTTCTATGGCGGGAAGGTGCTGGTGTTTGACGATTTCCCTGCCGACAAGGCAAAGGACCTGATGCAGC
TGGCCAGCAAGGGCGGCCCTGTGGTACAGAACGTTGTTTTGCCTCAACCCTCTGCACCTGCTGCTGCTGTCACCGACAAG
GCCGTGCCGGTCCCCGTCATCAGCTTGCCTGCTGCTCAGGCTGATGCTAAGAAGCCTACTCGCACAAATGCCTCTGATAT
GCCAATTATGAGGAAGGCTTCTCTTCACCGCTTCCTTGAGAAGAGAAAGGACCGTCTCAATGCAAATGCACCATACCAAA
CTTCTCCTTCAGACGCTGCACCAGTCAAGAAGGAGCCCGAGAGCCAGGCATGGCTCGGATTAGGACCGAATGCCGTCAAG
TCCAACCTGAACCTGAGCTAGCACACAACGACACAGCCAAATAAGACGGCACAGCAAACCTCACCAAAGCCTTTGCAGGA
CGATCTGAAAATATGCTTTGCCACTAGAGTACCGAAACCATAAATATCCTCTCGTTATCGTGTTTTAGTGTTCTGTTTAG
GCCGTGGTCCTACGCCCTCTTGTCACATAGTTCTGGATGTAAAGATAAGTAGCCAGCCATGACCGAGAGAAGGTTTTTTT
TGGGGGGTGTTAACACACGGCCACTCTGAATTGGCCATGCTAAGGAAAATGATAGTAGTGCAAAGAGAAGTATATGTCTT
TTTCTTTTTCTGAAAGCCCAGATCGTTTGGCTCTAGCCTTGATGTATGCAGAACAAGTGATTGTAATGGATTCTGAGTAT

CCTCATCGTCTTATTGTTGCTTCACCAACCCAAGTTTGTTTGGGTTCTTG

>Zm00001d006860_P002 peptide
MAASARPGERATSFAVACSLLSRFVRQNGAAPAQLGLGIKGEVEQQRTPATINLLPGADGEETERRKETMELFPQSAGFG
VKDAAAAPREQENKEKPKQLTIFYGGKVLVFDDFPADKAKDLMQLASKGGPVVQNVVLPQPSAPAAAVTDKAVPVPVISL
PAAQADAKKPTRTNASDMPIMRKASLHRFLEKRKDRLNANAPYQTSPSDAAPVKKEPESQAWLGLGPNAVKSNLNLS

ZmJAZ13

B73 RefGen_v3:
GRMZM2G151519 (zim35 - ZIM-transcription factor 35)
Chr4: 80927473..80930017

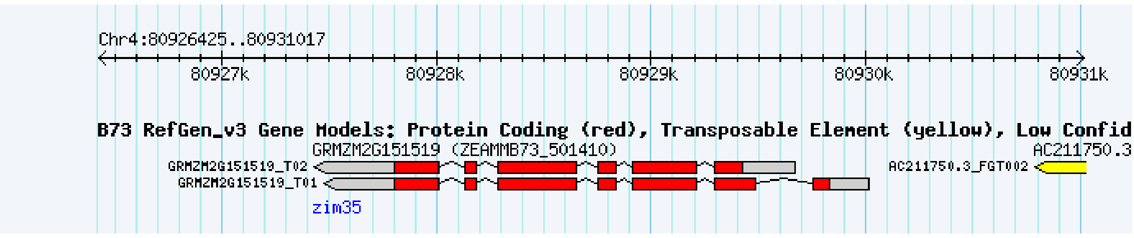

>GRMZM2G151519 Genomic DNA
AGAGAGAGTGAGAGAGAGAGGGGGAGAGAAAGGTTAAGGAATCCGAGCTAAGGCGCCGTTTCTTCTCCTCCTGCTTTTTT
CTTCTCGGCACCGTCACTGGAGGGAGAGGATTCACGGGACGAGGCGACGCGGAAGGGATTTCGTCGCTTGTGTGGGCGGC
GCGTCCGCTGGTTGCTTTGCTTCCATGGAGAGGGATTTCCTGGGTGCGATCGACAGGGCGTGGAAGTGCGGCGAGGCGGC
GAAGGAGGAGAGCCGATCGGAGCCAGGTCAGTGCTAGCTCCTCTCTCGATGCCCTGAAAGCGTGATTTACCTAACCGTAG
TACTAGTATGGCAGTAGCGACTCGGCGCGTCTACCGCCTGATCCACCGCGGTTTGCTTGCCGCGAGCATGGAAATTATTA
GTCATTCGACAAGTCAGAGAGATTTTATTTCCTCTCGTCGTCTCGTGGCCGCTGGTGCCCGCGCGTCAGATTTGGCCTGC
TCCGCTCCGCCTCCGCAGTGTGAATTTCTGAGAGATAGGTTTCTGTGCTGCAGACAGCCCGGCGGCGACGCGGTGGCAGT
TTGCGGCGAGGTCCGGCGTCGCGCCGGCGTTCATGTCGTTCAGGACGGAGGCGACGGGCGAGGGCTCTGAGGCGTTCTCG
GTCTCCGAGCTCCGGCCGGCCGGAGACGCCTTCGACGGCATCAAGAAGCAGAGCTCCATGCTTTCGATCTACCAGCAGCA
GGTAATAAGCTACTGCTACTCACCCGAGGTGCTGGATAGGGGCTCAACGCTCGGTAGTCTCAGCTGTTCACTTGTCTCTG
CAGAGGCAGTTTGGCCACAACAGCAGCCAGGCCACGGCACAGCAGCAGTACCCTGCAGCGGCGCACAGGCAGCGCTCGCA
GGTGGCGGATTACGGCGCGGCGGCACCGCATCGTCTCCCTGGTGGTGGTGGTGGTGGATGCAGGCTAGTGCAGCCGGTGT
CGGTGCGCCACCCTGTGCCGTTCCATCAGGCCAACCTGATGGTCAGGTCATCAGTGTCGCAGAGCTTCCACAACCAGCAG
CAGCCTTTCACCATGAGCAACAACGGGTTTGGCGGCTCTACGGCCGGCGTGTACGGGGCGAGGTATCGATCGGCAGTTCC
TTCCCTCCCTCCCTGTCGACATGTTGATCGACGGTTGATCGACCTTCCTCCTTGTTAGGAACCCACGGAACCAGGCCTCG
ACGCAGCTCACGATATTCTACAACGGCTCGGTGAATGTTTTCGACAACGTCCCGGTCGACAAGGTATGCAATGCTCTACC
CTTTTGCGACGAACTGAACACTTGGATCGCTCGTCACTGCAGTACGTTCCATCGGCTGCATCTGCTGATCGCTGTCCCGG
TGACAGGCTAAAGAGCTCATGATGCTGGCCAGCAGGGCGTCTGTTCCTGGTGCTACCCCGTCAAGTGAGCCGGACTCGCC
GCCTGTTCCTGCCCCTGCCCCTGCCAAGGTCAACGTGGCCGAGGTCTTCCCTGGTGCTAGACAGATCGCAGTTCAGAAAC
CGGAGCCCTGCGTGCCGCATCTATCAAGCGCGGCAACTGCAAGCCCAGTCCGCATCGTCGTGCCGCAAGCCGTGGCTCCC
TCCAGGAGCACGTCTCACTGCGCTACAGAGGCCTGCGGTTCCAAGCCTGCTGCTGCTCCTACCAGCCAGACAGTCTCGTC
GTCCCGGCAGTTGGCAGCTGCAAGTGCTGCTGCAGCTGTTACGCCAAGAGGTACTACCTATATACATACCTCTCGCTCGC
TTGCAAGTGCTGAAACCGTTCGTGCGACACGTTTGATTTACCCGTGTTTTGTTTGTTTGTTTGTTCTAGCTGTCCCTCAA
GCTCGGAAAGCGTCGCTCGCCCGGTTCTTGGAGAAGCGAAAGGAAAGGTATGGACTGTGCCTTGGTATACTTTCTATGGT
GTATATGCTCTGCTCAAGCTTGTTGTCGTCGACATCTGACCGATCCCTGCGTCAGGACACTAGTCTTCATCCTCCTTGTT

TCTCTCCGCAGAGTGGCTAGCGTCGAGCCATACCTGACGTCCAACTCCAAGAGCCCGCTAGAGAGCAGTGACGCCGTCGG
CAGCGCTAGCGCTCCAACGAAATCGTCATCCACAGACGTCGCCCCGGCGAGCAGCCACAACGGCGGCGGCGCAGAGCTTG
TGCGCCATGGCGGATATCCCAGGAGCATCAGCTTCAGCACAAATTTGCAGATCTGACTCTGGTGTTGCTGTCCATAGACT
ACCACTACCACTACGACTCCGTGTCCGTGAGCGCGCCCCTCCCTACCCATCGACACGAAAGCTGGATAGCTGTCTTGCTT
TGCATCCGGATGAGGCGGGAGGTATAGGAAGATACGATGAGATGAGAGTATAAAGATGCTGAGTGCTAGCCTAGCCGCCC
GCTGTATAGCTTTTGCAGCGCGCTGCTGACAAGATCGAGGGCCGTGACCTACTCGTTTCAGGCTCTTCTTGAATGGTTTT
TTTGTCGTGGTCCATTGTTGTATATTATTCAACTTGAGAAACAGAGAGTGCTGCTCTGTCTCTGATAAAAGTTTATATGA
AATTGATAAAAAGTTTCTGCTTTTTCCAAACAA

>GRMZM2G151519_T01 cDNA
AGAGAGAGTGAGAGAGAGAGGGGGAGAGAAAGGTTAAGGAATCCGAGCTAAGGCGCCGTTTCTTCTCCTCCTGCTTTTTT
CTTCTCGGCACCGTCACTGGAGGGAGAGGATTCACGGGACGAGGCGACGCGGAAGGGATTTCGTCGCTTGTGTGGGCGGC
GCGTCCGCTGGTTGCTTTGCTTCCATGGAGAGGGATTTCCTGGGTGCGATCGACAGGGCGTGGAAGTGCGGCGAGGCGGC
GAAGGAGGAGAGCCGATCGGAGCCAGACAGCCCGGCGGCGACGCGGTGGCAGTTTGCGGCGAGGTCCGGCGTCGCGCCGG
CGTTCATGTCGTTCAGGACGGAGGCGACGGGCGAGGGCTCTGAGGCGTTCTCGGTCTCCGAGCTCCGGCCGGCCGGAGAC
GCCTTCGACGGCATCAAGAAGCAGAGCTCCATGCTTTCGATCTACCAGCAGCAGAGGCAGTTTGGCCACAACAGCAGCCA
GGCCACGGCACAGCAGCAGTACCCTGCAGCGGCGCACAGGCAGCGCTCGCAGGTGGCGGATTACGGCGCGGCGGCACCGC
ATCGTCTCCCTGGTGGTGGTGGTGGTGGATGCAGGCTAGTGCAGCCGGTGTCGGTGCGCCACCCTGTGCCGTTCCATCAG
GCCAACCTGATGGTCAGGTCATCAGTGTCGCAGAGCTTCCACAACCAGCAGCAGCCTTTCACCATGAGCAACAACGGGTT
TGGCGGCTCTACGGCCGGCGTGTACGGGGCGAGGAACCCACGGAACCAGGCCTCGACGCAGCTCACGATATTCTACAACG
GCTCGGTGAATGTTTTCGACAACGTCCCGGTCGACAAGGCTAAAGAGCTCATGATGCTGGCCAGCAGGGCGTCTGTTCCT
GGTGCTACCCCGTCAAGTGAGCCGGACTCGCCGCCTGTTCCTGCCCCTGCCCCTGCCAAGGTCAACGTGGCCGAGGTCTT
CCCTGGTGCTAGACAGATCGCAGTTCAGAAACCGGAGCCCTGCGTGCCGCATCTATCAAGCGCGGCAACTGCAAGCCCAG
TCCGCATCGTCGTGCCGCAAGCCGTGGCTCCCTCCAGGAGCACGTCTCACTGCGCTACAGAGGCCTGCGGTTCCAAGCCT
GCTGCTGCTCCTACCAGCCAGACAGTCTCGTCGTCCCGGCAGTTGGCAGCTGCAAGTGCTGCTGCAGCTGTTACGCCAAG
AGCTGTCCCTCAAGCTCGGAAAGCGTCGCTCGCCCGGTTCTTGGAGAAGCGAAAGGAAAGAGTGGCTAGCGTCGAGCCAT
ACCTGACGTCCAACTCCAAGAGCCCGCTAGAGAGCAGTGACGCCGTCGGCAGCGCTAGCGCTCCAACGAAATCGTCATCC
ACAGACGTCGCCCCGGCGAGCAGCCACAACGGCGGCGGCGCAGAGCTTGTGCGCCATGGCGGATATCCCAGGAGCATCAG
CTTCAGCACAAATTTGCAGATCTGACTCTGGTGTTGCTGTCCATAGACTACCACTACCACTACGACTCCGTGTCCGTGAG
CGCGCCCCTCCCTACCCATCGACACGAAAGCTGGATAGCTGTCTTGCTTTGCATCCGGATGAGGCGGGAGGTATAGGAAG
ATACGATGAGATGAGAGTATAAAGATGCTGAGTGCTAGCCTAGCCGCCCGCTGTATAGCTTTTGCAGCGCGCTGCTGACA
AGATCGAGGGCCGTGACCTACTCGTTTCAGGCTCTTCTTGAATGGTTTTTTTGTCGTGGTCCATTGTTGTATATTATTCA
ACTTGAGAAACAGAGAGTGCTGCTCTGTCTCTGA
>GRMZM2G151519_P01 peptide
MERDFLGAIDRAWKCGEAAKEESRSEPDSPAATRWQFAARSGVAPAFMSFRTEATGEGSEAFSVSELRPAGDAFDGIKKQ
SSMLSIYQQQRQFGHNSSQATAQQQYPAAAHRQRSQVADYGAAAPHRLPGGGGGGCRLVQPVSVRHPVPFHQANLMVRSS
VSQSFHNQQQPFTMSNNGFGGSTAGVYGARNPRNQASTQLTIFYNGSVNVFDNVPVDKAKELMMLASRASVPGATPSSEP
DSPPVPAPAPAKVNVAEVFPGARQIAVQKPEPCVPHLSSAATASPVRIVVPQAVAPSRSTSHCATEACGSKPAAAPTSQT
VSSSRQLAAASAAAAVTPRAVPQARKASLARFLEKRKERVASVEPYLTSNSKSPLESSDAVGSASAPTKSSSTDVAPASS
HNGGGAELVRHGGYPRSISFSTNLQI
>GRMZM2G151519_T02 cDNA
TACCGCCTGATCCACCGCGGTTTGCTTGCCGCGAGCATGGAAATTATTAGTCATTCGACAAGTCAGAGAGATTTTATTTC
CTCTCGTCGTCTCGTGGCCGCTGGTGCCCGCGCGTCAGATTTGGCCTGCTCCGCTCCGCCTCCGCAGTGTGAATTTCTGA
GAGATAGGTTTCTGTGCTGCAGACAGCCCGGCGGCGACGCGGTGGCAGTTTGCGGCGAGGTCCGGCGTCGCGCCGGCGTT

CATGTCGTTCAGGACGGAGGCGACGGGCGAGGGCTCTGAGGCGTTCTCGGTCTCCGAGCTCCGGCCGGCCGGAGACGCCT
TCGACGGCATCAAGAAGCAGAGCTCCATGCTTTCGATCTACCAGCAGCAGAGGCAGTTTGGCCACAACAGCAGCCAGGCC
ACGGCACAGCAGCAGTACCCTGCAGCGGCGCACAGGCAGCGCTCGCAGGTGGCGGATTACGGCGCGGCGGCACCGCATCG
TCTCCCTGGTGGTGGTGGTGGTGGATGCAGGCTAGTGCAGCCGGTGTCGGTGCGCCACCCTGTGCCGTTCCATCAGGCCA
ACCTGATGGTCAGGTCATCAGTGTCGCAGAGCTTCCACAACCAGCAGCAGCCTTTCACCATGAGCAACAACGGGTTTGGC
GGCTCTACGGCCGGCGTGTACGGGGCGAGGAACCCACGGAACCAGGCCTCGACGCAGCTCACGATATTCTACAACGGCTC
GGTGAATGTTTTCGACAACGTCCCGGTCGACAAGGCTAAAGAGCTCATGATGCTGGCCAGCAGGGCGTCTGTTCCTGGTG
CTACCCCGTCAAGTGAGCCGGACTCGCCGCCTGTTCCTGCCCCTGCCCCTGCCAAGGTCAACGTGGCCGAGGTCTTCCCT
GGTGCTAGACAGATCGCAGTTCAGAAACCGGAGCCCTGCGTGCCGCATCTATCAAGCGCGGCAACTGCAAGCCCAGTCCG
CATCGTCGTGCCGCAAGCCGTGGCTCCCTCCAGGAGCACGTCTCACTGCGCTACAGAGGCCTGCGGTTCCAAGCCTGCTG
CTGCTCCTACCAGCCAGACAGTCTCGTCGTCCCGGCAGTTGGCAGCTGCAAGTGCTGCTGCAGCTGTTACGCCAAGAGCT
GTCCCTCAAGCTCGGAAAGCGTCGCTCGCCCGGTTCTTGGAGAAGCGAAAGGAAAGAGTGGCTAGCGTCGAGCCATACCT
GACGTCCAACTCCAAGAGCCCGCTAGAGAGCAGTGACGCCGTCGGCAGCGCTAGCGCTCCAACGAAATCGTCATCCACAG
ACGTCGCCCCGGCGAGCAGCCACAACGGCGGCGGCGCAGAGCTTGTGCGCCATGGCGGATATCCCAGGAGCATCAGCTTC
AGCACAAATTTGCAGATCTGACTCTGGTGTTGCTGTCCATAGACTACCACTACCACTACGACTCCGTGTCCGTGAGCGCG
CCCCTCCCTACCCATCGACACGAAAGCTGGATAGCTGTCTTGCTTTGCATCCGGATGAGGCGGGAGGTATAGGAAGATAC
GATGAGATGAGAGTATAAAGATGCTGAGTGCTAGCCTAGCCGCCCGCTGTATAGCTTTTGCAGCGCGCTGCTGACAAGAT
CGAGGGCCGTGACCTACTCGTTTCAGGCTCTTCTTGAATGGTTTTTTTGTCGTGGTCCATTGTTGTATATTATTCAACTT
GAGAAACAGAGAGTGCTGCTCTGTCTCTGATAAAAGTTTATATGAAATTGATAAAAAGTTTCTGCTTTTTCCAAACAA
>GRMZM2G151519_P02 peptide
MSFRTEATGEGSEAFSVSELRPAGDAFDGIKKQSSMLSIYQQQRQFGHNSSQATAQQQYPAAAHRQRSQVADYGAAAPHR
LPGGGGGGCRLVQPVSVRHPVPFHQANLMVRSSVSQSFHNQQQPFTMSNNGFGGSTAGVYGARNPRNQASTQLTIFYNGS
VNVFDNVPVDKAKELMMLASRASVPGATPSSEPDSPPVPAPAPAKVNVAEVFPGARQIAVQKPEPCVPHLSSAATASPVR
IVVPQAVAPSRSTSHCATEACGSKPAAAPTSQTVSSSRQLAAASAAAAVTPRAVPQARKASLARFLEKRKERVASVEPYL
TSNSKSPLESSDAVGSASAPTKSSSTDVAPASSHNGGGAELVRHGGYPRSISFSTNLQI

AGPv4:
Zm00001d050365 (zim35 - ZIM-transcription factor 35)
Chr4: 83772143..83775016

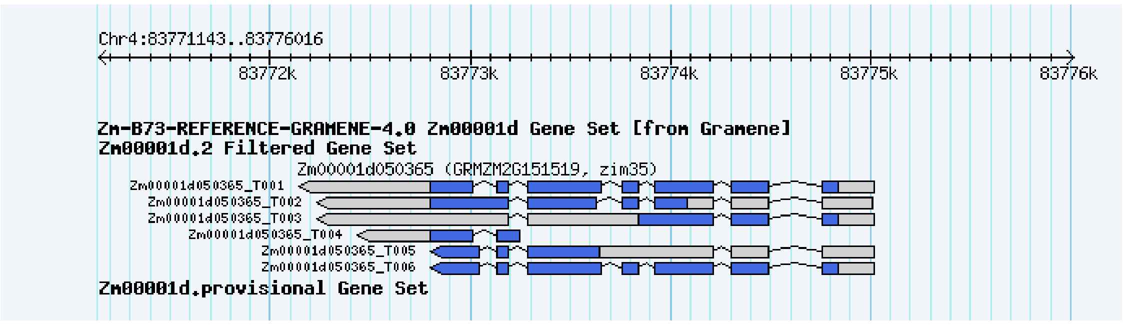

>Zm00001d050365 Genomic DNA
GAGAGTGAGAGAGAGAGGGGGAGAGAAAGGTTAAGGAATCCGAGCTAAGGCGCCGTTTCTTCTCCTCCTGCTTTTTTCTT
CTCGGCACCGTCACTGGAGGGAGAGGATTCACGGGACGAGGCGACGCGGAAGGGATTTCGTCGCTTGTGTGGGCGGCGCG
TCCGCTGGTTGCTTTGCTTCCATGGAGAGGGATTTCCTGGGTGCGATCGACAGGGCGTGGAAGTGCGGCGAGGCGGCGAA
GGAGGAGAGCCGATCGGAGCCAGGTCAGTGCTAGCTCCTCTCTCGATGCCCTGAAAGCGTGATTTACCTAACCGTAGTAC
TAGTATGGCAGTAGCGACTCGGCGCGTCTACCGCCTGATCCACCGCGGTTTGCTTGCCGCGAGCATGGAAATTATTAGTC
ATTCGACAAGTCAGAGAGATTTTATTTCCTCTCGTCGTCTCGTGGCCGCTGGTGCCCGCGCGTCAGATTTGGCCTGCTCC

GCTCCGCCTCCGCAGTGTGAATTTCTGAGAGATAGGTTTCTGTGCTGCAGACAGCCCGGCGGCGACGCGGTGGCAGTTTG
CGGCGAGGTCCGGCGTCGCGCCGGCGTTCATGTCGTTCAGGACGGAGGCGACGGGCGAGGGCTCTGAGGCGTTCTCGGTC
TCCGAGCTCCGGCCGGCCGGAGACGCCTTCGACGGCATCAAGAAGCAGAGCTCCATGCTTTCGATCTACCAGCAGCAGGT
AATAAGCTACTGCTACTCACCCGAGGTGCTGGATAGGGGCTCAACGCTCGGTAGTCTCAGCTGTTCACTTGTCTCTGCAG
AGGCAGTTTGGCCACAACAGCAGCCAGGCCACGGCACAGCAGCAGTACCCTGCAGCGGCGCACAGGCAGCGCTCGCAGGT
GGCGGATTACGGCGCGGCGGCACCGCATCGTCTCCCTGGTGGTGGTGGTGGTGGATGCAGGCTAGTGCAGCCGGTGTCGG
TGCGCCACCCTGTGCCGTTCCATCAGGCCAACCTGATGGTCAGGTCATCAGTGTCGCAGAGCTTCCACAACCAGCAGCAG
CCTTTCACCATGAGCAACAACGGGTTTGGCGGCTCTACGGCCGGCGTGTACGGGGCGAGGTATCGATCGGCAGTTCCTTC
CCTCCCTCCCTGTCGACATGTTGATCGACGGTTGATCGACCTTCCTCCTTGTTAGGAACCCACGGAACCAGGCCTCGACG
CAGCTCACGATATTCTACAACGGCTCGGTGAATGTTTTCGACAACGTCCCGGTCGACAAGGTATGCAATGCTCTACCCTT
TTGCGACGAACTGAACACTTGGATCGCTCGTCACTGCAGTACGTTCCATCGGCTGCATCTGCTGATCGCTGTCCCGGTGA
CAGGCTAAAGAGCTCATGATGCTGGCCAGCAGGGCGTCTGTTCCTGGTGCTACCCCGTCAAGTGAGCCGGACTCGCCGCC
TGTTCCTGCCCCTGCCCCTGCCAAGGTCAACGTGGCCGAGGTCTTCCCTGGTGCTAGACAGATCGCAGTTCAGAAACCGG
AGCCCTGCGTGCCGCATCTATCAAGCGCGGCAACTGCAAGCCCAGTCCGCATCGTCGTGCCGCAAGCCGTGGCTCCCTCC
AGGAGCACGTCTCACTGCGCTACAGAGGCCTGCGGTTCCAAGCCTGCTGCTGCTCCTACCAGCCAGACAGTCTCGTCGTC
CCGGCAGTTGGCAGCTGCAAGTGCTGCTGCAGCTGTTACGCCAAGAGGTACTACCTATATACATACCTCTCGCTCGCTTG
CAAGTGCTGAAACCGTTCGTGCGACACGTTTGATTTACCCGTGTTTTGTTTGTTTGTTTGTTCTAGCTGTCCCTCAAGCT
CGGAAAGCGTCGCTCGCCCGGTTCTTGGAGAAGCGAAAGGAAAGGTATGGACTGTGCCTTGGTATACTTTCTATGGTGTA
TATGCTCTGCTCAAGCTTGTTGTCGTCGACATCTGACCGATCCCTGCGTCAGGACACTAGTCTTCATCCTCCTTGTTTCT
CTCCGCAGAGTGGCTAGCGTCGAGCCATACCTGACGTCCAACTCCAAGAGCCCGCTAGAGAGCAGTGACGCCGTCGGCAG
CGCTAGCGCTCCAACGAAATCGTCATCCACAGACGTCGCCCCGGCGAGCAGCCACAACGGCGGCGGCGCAGAGCTTGTGC
GCCATGGCGGATATCCCAGGAGCATCAGCTTCAGCACAAATTTGCAGATCTGACTCTGGTGTTGCTGTCCATAGACTACC
ACTACCACTACGACTCCGTGTCCGTGAGCGCGCCCCTCCCTACCCATCGACACGAAAGCTGGATAGCTGTCTTGCTTTGC
ATCCGGATGAGGCGGGAGGTATAGGAAGATACGATGAGATGAGAGTATAAAGATGCTGAGTGCTAGCCTAGCCGCCCGCT
GTATAGCTTTTGCAGCGCGCTGCTGACAAGATCGAGGGCCGTGACCTACTCGTTTCAGGCTCTTCTTGAATGGTTTTTTT
GTCGTGGTCCATTGTTGTATATTATTCAACTTGAGAAACAGAGAGTGCTGCTCTGTCTCTGATAAAAGTTTATATGAAAT
TGATAAAAAGTTTCTGCTTTTTCCAAACAAGGTCCTTACTGACTCGGAAGTGGTACATGGAGCTGGGGAGGGGGGCTACG
TTTCGGACAGAACAGATAACAAGTTGAAGAGAGCCAATTCGATAACGGTGTTGCTGCCCAAGGCTGCAATACGCCAGCAA
AGTAGCTATGAACGTAATTTTGCTTGAATTATGTGCCAAACTTTGGGCAAAAATCATAAACTTTCATCGATATTGGTAGC
CTGGAAATTGCATCTGGTCGGTCAAAGATTCAAGATCCACAGTTTAGTAAAACCATTGGCAATCGTGACCACTT
>Zm00001d050365_T001 cDNA
Chr4: 83772143..83775014
GAGTGAGAGAGAGAGGGGGAGAGAAAGGTTAAGGAATCCGAGCTAAGGCGCCGTTTCTTCTCCTCCTGCTTTTTTCTTCT
CGGCACCGTCACTGGAGGGAGAGGATTCACGGGACGAGGCGACGCGGAAGGGATTTCGTCGCTTGTGTGGGCGGCGCGTC
CGCTGGTTGCTTTGCTTCCATGGAGAGGGATTTCCTGGGTGCGATCGACAGGGCGTGGAAGTGCGGCGAGGCGGCGAAGG
AGGAGAGCCGATCGGAGCCAGACAGCCCGGCGGCGACGCGGTGGCAGTTTGCGGCGAGGTCCGGCGTCGCGCCGGCGTTC
ATGTCGTTCAGGACGGAGGCGACGGGCGAGGGCTCTGAGGCGTTCTCGGTCTCCGAGCTCCGGCCGGCCGGAGACGCCTT
CGACGGCATCAAGAAGCAGAGCTCCATGCTTTCGATCTACCAGCAGCAGAGGCAGTTTGGCCACAACAGCAGCCAGGCCA
CGGCACAGCAGCAGTACCCTGCAGCGGCGCACAGGCAGCGCTCGCAGGTGGCGGATTACGGCGCGGCGGCACCGCATCGT
CTCCCTGGTGGTGGTGGTGGTGGATGCAGGCTAGTGCAGCCGGTGTCGGTGCGCCACCCTGTGCCGTTCCATCAGGCCAA
CCTGATGGTCAGGTCATCAGTGTCGCAGAGCTTCCACAACCAGCAGCAGCCTTTCACCATGAGCAACAACGGGTTTGGCG
GCTCTACGGCCGGCGTGTACGGGGCGAGGAACCCACGGAACCAGGCCTCGACGCAGCTCACGATATTCTACAACGGCTCG
GTGAATGTTTTCGACAACGTCCCGGTCGACAAGGCTAAAGAGCTCATGATGCTGGCCAGCAGGGCGTCTGTTCCTGGTGC
TACCCCGTCAAGTGAGCCGGACTCGCCGCCTGTTCCTGCCCCTGCCCCTGCCAAGGTCAACGTGGCCGAGGTCTTCCCTG

GTGCTAGACAGATCGCAGTTCAGAAACCGGAGCCCTGCGTGCCGCATCTATCAAGCGCGGCAACTGCAAGCCCAGTCCGC
ATCGTCGTGCCGCAAGCCGTGGCTCCCTCCAGGAGCACGTCTCACTGCGCTACAGAGGCCTGCGGTTCCAAGCCTGCTGC
TGCTCCTACCAGCCAGACAGTCTCGTCGTCCCGGCAGTTGGCAGCTGCAAGTGCTGCTGCAGCTGTTACGCCAAGAGCTG
TCCCTCAAGCTCGGAAAGCGTCGCTCGCCCGGTTCTTGGAGAAGCGAAAGGAAAGAGTGGCTAGCGTCGAGCCATACCTG
ACGTCCAACTCCAAGAGCCCGCTAGAGAGCAGTGACGCCGTCGGCAGCGCTAGCGCTCCAACGAAATCGTCATCCACAGA
CGTCGCCCCGGCGAGCAGCCACAACGGCGGCGGCGCAGAGCTTGTGCGCCATGGCGGATATCCCAGGAGCATCAGCTTCA
GCACAAATTTGCAGATCTGACTCTGGTGTTGCTGTCCATAGACTACCACTACCACTACGACTCCGTGTCCGTGAGCGCGC
CCCTCCCTACCCATCGACACGAAAGCTGGATAGCTGTCTTGCTTTGCATCCGGATGAGGCGGGAGGTATAGGAAGATACG
ATGAGATGAGAGTATAAAGATGCTGAGTGCTAGCCTAGCCGCCCGCTGTATAGCTTTTGCAGCGCGCTGCTGACAAGATC
GAGGGCCGTGACCTACTCGTTTCAGGCTCTTCTTGAATGGTTTTTTTGTCGTGGTCCATTGTTGTATATTATTCAACTTG
AGAAACAGAGAGTGCTGCTCTGTCTCTGATAAAAGTTTATATGAAATTGATAAAAAGTTTCTGCTTTTTCCAAACAAGGT
CCTTACTGACTCGGAAGTGGTACATGGAGCTGGGGAGGGGGGCTACGTTTCGGACAGAACAGATAACAAGTTGAAGAGAG
CCAATTCGATAACGGTGTTGCTGCCCAAGGCTGCAATACGCCAGCAAAGTAGCTATGAACGTAATTTTGCTTGAATTATG
TGCCAAACTTTGGGCAAAAATCATAAACTTTCATCGATATTGGTAGCCTGGAAATTGCATCTGGTCGGTCAAAGATTCAA
GATCCACAGTTTAGTAAAACCATTGGCAATCGTGACCACTT
>Zm00001d050365_P001 peptide
MERDFLGAIDRAWKCGEAAKEESRSEPDSPAATRWQFAARSGVAPAFMSFRTEATGEGSEAFSVSELRPAGDAFDGIKKQ
SSMLSIYQQQRQFGHNSSQATAQQQYPAAAHRQRSQVADYGAAAPHRLPGGGGGGCRLVQPVSVRHPVPFHQANLMVRSS
VSQSFHNQQQPFTMSNNGFGGSTAGVYGARNPRNQASTQLTIFYNGSVNVFDNVPVDKAKELMMLASRASVPGATPSSEP
DSPPVPAPAPAKVNVAEVFPGARQIAVQKPEPCVPHLSSAATASPVRIVVPQAVAPSRSTSHCATEACGSKPAAAPTSQT
VSSSRQLAAASAAAAVTPRAVPQARKASLARFLEKRKERVASVEPYLTSNSKSPLESSDAVGSASAPTKSSSTDVAPASS
HNGGGAELVRHGGYPRSISFSTNLQI
>Zm00001d050365_T002 cDNA
Chr4: 83772229..83775008
GAGAGAGAGGGGGAGAGAAAGGTTAAGGAATCCGAGCTAAGGCGCCGTTTCTTCTCCTCCTGCTTTTTTCTTCTCGGCAC
CGTCACTGGAGGGAGAGGATTCACGGGACGAGGCGACGCGGAAGGGATTTCGTCGCTTGTGTGGGCGGCGCGTCCGCTGG
TTGCTTTGCTTCCATGGAGAGGGATTTCCTGGGTGCGATCGACAGGGCGTGGAAGTGCGGCGAGGCGGCGAAGGAGGAGA
GCCGATCGGAGCCAGACAGCCCGGCGGCGACGCGGTGGCAGTTTGCGGCGAGGTCCGGCGTCGCGCCGGCGTTCATGTCG
TTCAGGACGGAGGCGACGGGCGAGGGCTCTGAGGCGTTCTCGGTCTCCGAGCTCCGGCCGGCCGGAGACGCCTTCGACGG
CATCAAGAAGCAGAGCTCCATGCTTTCGATCTACCAGCAGCAGAGGCAGTTTGGCCACAACAGCAGCCAGGCCACGGCAC
AGCAGCAGTACCCTGCAGCGGCGCACAGGCAGCGCTCGCAGGTGGCGGATTACGGCGCGGCGGCACCGCATCGTCTCCCT
GGTGGTGGTGGTGGTGGATGCAGGCTAGTGCAGCCGGTGTCGGTGCGCCACCCTGTGCCGTTCCATCAGGCCAACCTGAT
GGTCAGGTCATCAGTGTCGCAGAGCTTCCACAACCAGCAGCAGCCTTTCACCATGAGCAACAACGGGTTTGGCGGCTCTA
CGGCCGGCGTGTACGGGGCGAGGAACCCACGGAACCAGGCCTCGACGCAGCTCACGATATTCTACAACGGCTCGGTGAAT
GTTTTCGACAACGTCCCGGTCGACAAGGGCGTCTGTTCCTGGTGCTACCCCGTCAAGTGAGCCGGACTCGCCGCCTGTTC
CTGCCCCTGCCCCTGCCAAGGTCAACGTGGCCGAGGTCTTCCCTGGTGCTAGACAGATCGCAGTTCAGAAACCGGAGCCC
TGCGTGCCGCATCTATCAAGCGCGGCAACTGCAAGCCCAGTCCGCATCGTCGTGCCGCAAGCCGTGGCTCCCTCCAGGAG
CACGTCTCACTGCGCTACAGAGGCCTGCGGTTCCAAGCCTGCTGCTGCTCCTACCAGCCAGACAGTCTCGTCGTCCCGGC
AGTTGGCAGCTGCAAGTGCTGCTGCAGCTGTTACGCCAAGAGCTGTCCCTCAAGCTCGGAAAGCGTCGCTCGCCCGGTTC
TTGGAGAAGCGAAAGGAAAGGTATGGACTGTGCCTTGGTATACTTTCTATGGTGTATATGCTCTGCTCAAGCTTGTTGTC
GTCGACATCTGACCGATCCCTGCGTCAGGACACTAGTCTTCATCCTCCTTGTTTCTCTCCGCAGAGTGGCTAGCGTCGAG
CCATACCTGACGTCCAACTCCAAGAGCCCGCTAGAGAGCAGTGACGCCGTCGGCAGCGCTAGCGCTCCAACGAAATCGTC
ATCCACAGACGTCGCCCCGGCGAGCAGCCACAACGGCGGCGGCGCAGAGCTTGTGCGCCATGGCGGATATCCCAGGAGCA
TCAGCTTCAGCACAAATTTGCAGATCTGACTCTGGTGTTGCTGTCCATAGACTACCACTACCACTACGACTCCGTGTCCG

TGAGCGCGCCCCTCCCTACCCATCGACACGAAAGCTGGATAGCTGTCTTGCTTTGCATCCGGATGAGGCGGGAGGTATAG
GAAGATACGATGAGATGAGAGTATAAAGATGCTGAGTGCTAGCCTAGCCGCCCGCTGTATAGCTTTTGCAGCGCGCTGCT
GACAAGATCGAGGGCCGTGACCTACTCGTTTCAGGCTCTTCTTGAATGGTTTTTTTGTCGTGGTCCATTGTTGTATATTA
TTCAACTTGAGAAACAGAGAGTGCTGCTCTGTCTCTGATAAAAGTTTATATGAAATTGATAAAAAGTTTCTGCTTTTTCC
AAACAAGGTCCTTACTGACTCGGAAGTGGTACATGGAGCTGGGGAGGGGGGCTACGTTTCGGACAGAACAGATAACAAGT
TGAAGAGAGCCAATTCGATAACGGTGTTGCTGCCCAAGGCTGCAATACGCCAGCAAAGTAGCTATGAACGTAATTTTGCT
TGAATTATGTGCCAAACTTTGGGCAAAAATCATAAACTTTCATC
>Zm00001d050365_P002 peptide
MQASAAGVGAPPCAVPSGQPDGQVISVAELPQPAAAFHHEQQRVWRLYGRRVRGEEPTEPGLDAAHDILQRLGECFRQRP
GRQGRLFLVLPRQVSRTRRLFLPLPLPRSTWPRSSLVLDRSQFRNRSPACRIYQARQLQAQSASSCRKPWLPPGARLTAL
QRPAVPSLLLLLPARQSRRPGSWQLQVLLQLLRQELSLKLGKRRSPGSWRSERKGMDCALVYFLWCICSAQACCRRHLTD
PCVRTLVFILLVSLRRVASVEPYLTSNSKSPLESSDAVGSASAPTKSSSTDVAPASSHNGGGAELVRHGGYPRSISFSTN
LQI
>Zm00001d050365_T003 cDNA
Chr4: 83772229..83775016
GAGAGTGAGAGAGAGAGGGGGAGAGAAAGGTTAAGGAATCCGAGCTAAGGCGCCGTTTCTTCTCCTCCTGCTTTTTTCTT
CTCGGCACCGTCACTGGAGGGAGAGGATTCACGGGACGAGGCGACGCGGAAGGGATTTCGTCGCTTGTGTGGGCGGCGCG
TCCGCTGGTTGCTTTGCTTCCATGGAGAGGGATTTCCTGGGTGCGATCGACAGGGCGTGGAAGTGCGGCGAGGCGGCGAA
GGAGGAGAGCCGATCGGAGCCAGACAGCCCGGCGGCGACGCGGTGGCAGTTTGCGGCGAGGTCCGGCGTCGCGCCGGCGT
TCATGTCGTTCAGGACGGAGGCGACGGGCGAGGGCTCTGAGGCGTTCTCGGTCTCCGAGCTCCGGCCGGCCGGAGACGCC
TTCGACGGCATCAAGAAGCAGAGCTCCATGCTTTCGATCTACCAGCAGCAGAGGCAGTTTGGCCACAACAGCAGCCAGGC
CACGGCACAGCAGCAGTACCCTGCAGCGGCGCACAGGCAGCGCTCGCAGGTGGCGGATTACGGCGCGGCGGCACCGCATC
GTCTCCCTGGTGGTGGTGGTGGTGGATGCAGGCTAGTGCAGCCGGTGTCGGTGCGCCACCCTGTGCCGTTCCATCAGGCC
AACCTGATGGTCAGGTCATCAGTGTCGCAGAGCTTCCACAACCAGCAGCAGCCTTTCACCATGAGCAACAACGGGTTTGG
CGGCTCTACGGCCGGCGTGTACGGGGCGAGGTATCGATCGGCAGTTCCTTCCCTCCCTCCCTGTCGACATGTTGATCGAC
GGTTGATCGACCTTCCTCCTTGTTAGGAACCCACGGAACCAGGCCTCGACGCAGCTCACGATATTCTACAACGGCTCGGT
GAATGTTTTCGACAACGTCCCGGTCGACAAGGTATGCAATGCTCTACCCTTTTGCGACGAACTGAACACTTGGATCGCTC
GTCACTGCAGTACGTTCCATCGGCTGCATCTGCTGATCGCTGTCCCGGTGACAGGCTAAAGAGCTCATGATGCTGGCCAG
CAGGGCGTCTGTTCCTGGTGCTACCCCGTCAAGTGAGCCGGACTCGCCGCCTGTTCCTGCCCCTGCCCCTGCCAAGGTCA
ACGTGGCCGAGGTCTTCCCTGGTGCTAGACAGATCGCAGTTCAGAAACCGGAGCCCTGCGTGCCGCATCTATCAAGCGCG
GCAACTGCAAGCCCAGTCCGCATCGTCGTGCCGCAAGCCGTGGCTCCCTCCAGGAGCACGTCTCACTGCGCTACAGAGGC
CTGCGGTTCCAAGCCTGCTGCTGCTCCTACCAGCCAGACAGTCTCGTCGTCCCGGCAGTTGGCAGCTGCAAGTGCTGCTG
CAGCTGTTACGCCAAGAGCTGTCCCTCAAGCTCGGAAAGCGTCGCTCGCCCGGTTCTTGGAGAAGCGAAAGGAAAGGTAT
GGACTGTGCCTTGGTATACTTTCTATGGTGTATATGCTCTGCTCAAGCTTGTTGTCGTCGACATCTGACCGATCCCTGCG
TCAGGACACTAGTCTTCATCCTCCTTGTTTCTCTCCGCAGAGTGGCTAGCGTCGAGCCATACCTGACGTCCAACTCCAAG
AGCCCGCTAGAGAGCAGTGACGCCGTCGGCAGCGCTAGCGCTCCAACGAAATCGTCATCCACAGACGTCGCCCCGGCGAG
CAGCCACAACGGCGGCGGCGCAGAGCTTGTGCGCCATGGCGGATATCCCAGGAGCATCAGCTTCAGCACAAATTTGCAGA
TCTGACTCTGGTGTTGCTGTCCATAGACTACCACTACCACTACGACTCCGTGTCCGTGAGCGCGCCCCTCCCTACCCATC
GACACGAAAGCTGGATAGCTGTCTTGCTTTGCATCCGGATGAGGCGGGAGGTATAGGAAGATACGATGAGATGAGAGTAT
AAAGATGCTGAGTGCTAGCCTAGCCGCCCGCTGTATAGCTTTTGCAGCGCGCTGCTGACAAGATCGAGGGCCGTGACCTA
CTCGTTTCAGGCTCTTCTTGAATGGTTTTTTTGTCGTGGTCCATTGTTGTATATTATTCAACTTGAGAAACAGAGAGTGC
TGCTCTGTCTCTGATAAAAGTTTATATGAAATTGATAAAAAGTTTCTGCTTTTTCCAAACAAGGTCCTTACTGACTCGGA
AGTGGTACATGGAGCTGGGGAGGGGGGCTACGTTTCGGACAGAACAGATAACAAGTTGAAGAGAGCCAATTCGATAACGG
TGTTGCTGCCCAAGGCTGCAATACGCCAGCAAAGTAGCTATGAACGTAATTTTGCTTGAATTATGTGCCAAACTTTGGGC

AAAAATCATAAACTTTCATC
>Zm00001d050365_P003 peptide
MERDFLGAIDRAWKCGEAAKEESRSEPDSPAATRWQFAARSGVAPAFMSFRTEATGEGSEAFSVSELRPAGDAFDGIKKQ
SSMLSIYQQQRQFGHNSSQATAQQQYPAAAHRQRSQVADYGAAAPHRLPGGGGGGCRLVQPVSVRHPVPFHQANLMVRSS
VSQSFHNQQQPFTMSNNGFGGSTAGVYGARYRSAVPSLPPCRHVDRRLIDLPPC
>Zm00001d050365_T004 cDNA
Chr4: 83772435..83773248
GAAACCGTTCGTGCGACACGTTTGATTTACCCGTGTTTTGTTTGTTTGTTTGTTCTAGCTGTCCCTCAAGCTCGGAAAGC
GTCGCTCGCCCGGTTCTTGGAGAAGCGAAAGGAAAGAGTGGCTAGCGTCGAGCCATACCTGACGTCCAACTCCAAGAGCC
CGCTAGAGAGCAGTGACGCCGTCGGCAGCGCTAGCGCTCCAACGAAATCGTCATCCACAGACGTCGCCCCGGCGAGCAGC
CACAACGGCGGCGGCGCAGAGCTTGTGCGCCATGGCGGATATCCCAGGAGCATCAGCTTCAGCACAAATTTGCAGATCTG
ACTCTGGTGTTGCTGTCCATAGACTACCACTACCACTACGACTCCGTGTCCGTGAGCGCGCCCCTCCCTACCCATCGACA
CGAAAGCTGGATAGCTGTCTTGCTTTGCATCCGGATGAGGCGGGAGGTATAGGAAGATACGATGAGATGAGAGTATAAAG
ATGCTGAGTGCTAGCCTAGCCGCCCGCTGTATAGCTTTTGCAGCGCGCTGCTGACAAGATCGAGGGCCGTGACCTACTCG
TTTCAGGCTCTTCTTGAATGGTTTTTTTGTCGTGGTCCATTGTTGTATATTATTCAACTTGAGAAACAGAGAGTGCTGCT
CTGTCTCTGATAAAAGTTTATATGAAATTGATAAAAAGTTTCTGCTTTTT
>Zm00001d050365_P004 peptide
ETVRATRLIYPCFVCLFVLAVPQARKASLARFLEKRKERVASVEPYLTSNSKSPLESSDAVGSASAPTKSSSTDVAPASS
HNGGGAELVRHGGYPRSISFSTNLQI
>Zm00001d050365_T005 cDNA
Chr4: 83772804..83775016
GAGAGTGAGAGAGAGAGGGGGAGAGAAAGGTTAAGGAATCCGAGCTAAGGCGCCGTTTCTTCTCCTCCTGCTTTTTTCTT
CTCGGCACCGTCACTGGAGGGAGAGGATTCACGGGACGAGGCGACGCGGAAGGGATTTCGTCGCTTGTGTGGGCGGCGCG
TCCGCTGGTTGCTTTGCTTCCATGGAGAGGGATTTCCTGGGTGCGATCGACAGGGCGTGGAAGTGCGGCGAGGCGGCGAA
GGAGGAGAGCCGATCGGAGCCAGACAGCCCGGCGGCGACGCGGTGGCAGTTTGCGGCGAGGTCCGGCGTCGCGCCGGCGT
TCATGTCGTTCAGGACGGAGGCGACGGGCGAGGGCTCTGAGGCGTTCTCGGTCTCCGAGCTCCGGCCGGCCGGAGACGCC
TTCGACGGCATCAAGAAGCAGAGCTCCATGCTTTCGATCTACCAGCAGCAGAGGCAGTTTGGCCACAACAGCAGCCAGGC
CACGGCACAGCAGCAGTACCCTGCAGCGGCGCACAGGCAGCGCTCGCAGGTGGCGGATTACGGCGCGGCGGCACCGCATC
GTCTCCCTGGTGGTGGTGGTGGTGGATGCAGGCTAGTGCAGCCGGTGTCGGTGCGCCACCCTGTGCCGTTCCATCAGGCC
AACCTGATGGTCAGGTCATCAGTGTCGCAGAGCTTCCACAACCAGCAGCAGCCTTTCACCATGAGCAACAACGGGTTTGG
CGGCTCTACGGCCGGCGTGTACGGGGCGAGGTATCGATCGGCAGTTCCTTCCCTCCCTCCCTGTCGACATGTTGATCGAC
GGTTGATCGACCTTCCTCCTTGTTAGGAACCCACGGAACCAGGCCTCGACGCAGCTCACGATATTCTACAACGGCTCGGT
GAATGTTTTCGACAACGTCCCGGTCGACAAGGTATGCAATGCTCTACCCTTTTGCGACGAACTGAACACTTGGATCGCTC
GTCACTGCAGTACGTTCCATCGGCTGCATCTGCTGATCGCTGTCCCGGTGACAGGCTAAAGAGCTCATGATGCTGGCCAG
CAGGGCGTCTGTTCCTGGTGCTACCCCGTCAAGTGAGCCGGACTCGCCGCCTGTTCCTGCCCCTGCCCCTGCCAAGGTCA
ACGTGGCCGAGGTCTTCCCTGGTGCTAGACAGATCGCAGTTCAGAAACCGGAGCCCTGCGTGCCGCATCTATCAAGCGCG
GCAACTGCAAGCCCAGTCCGCATCGTCGTGCCGCAAGCCGTGGCTCCCTCCAGGAGCACGTCTCACTGCGCTACAGAGGC
CTGCGGTTCCAAGCCTGCTGCTGCTCCTACCAGCCAGACAGTCTCGTCGTCCCGGCAGTTGGCAGCTGCAAGTGCTGCTG
CAGCTGTTACGCCAAGAGCTGTCCCTCAAGCTCGGAAAGCGTCGCTCGCCCGGTTCTTGGAGAAGCGAAAGGAAAGGACA
CTAGTCTTCATCCTCCTTGTTTCTCTCCGCAGAGTGGCTAGCGTCGAGCCATACCTGACGTCCAACTCCAAGAGCCCGCT
AGAGAGCAGTGACGCCGTCGGCAGCGCTAGCGCTCCAACGAAATCGTCATCCACAGACGTCGCCCCGGCGAGCAGCCACA
ACGGCGGCGGCGCAGAGCTTGTGCGCCATGGCGGATATCCCAGGAGCATCAGCTTCAGCACAAATTTGCAGATCTGA
>Zm00001d050365_P005 peptide
MMLASRASVPGATPSSEPDSPPVPAPAPAKVNVAEVFPGARQIAVQKPEPCVPHLSSAATASPVRIVVPQAVAPSRSTSH

CATEACGSKPAAAPTSQTVSSSRQLAAASAAAAVTPRAVPQARKASLARFLEKRKERTLVFILLVSLRRVASVEPYLTSN
SKSPLESSDAVGSASAPTKSSSTDVAPASSHNGGGAELVRHGGYPRSISFSTNLQI
>Zm00001d050365_T006 cDNA
Chr4: 83772804..83775016
GAGAGTGAGAGAGAGAGGGGGAGAGAAAGGTTAAGGAATCCGAGCTAAGGCGCCGTTTCTTCTCCTCCTGCTTTTTTCTT
CTCGGCACCGTCACTGGAGGGAGAGGATTCACGGGACGAGGCGACGCGGAAGGGATTTCGTCGCTTGTGTGGGCGGCGCG
TCCGCTGGTTGCTTTGCTTCCATGGAGAGGGATTTCCTGGGTGCGATCGACAGGGCGTGGAAGTGCGGCGAGGCGGCGAA
GGAGGAGAGCCGATCGGAGCCAGACAGCCCGGCGGCGACGCGGTGGCAGTTTGCGGCGAGGTCCGGCGTCGCGCCGGCGT
TCATGTCGTTCAGGACGGAGGCGACGGGCGAGGGCTCTGAGGCGTTCTCGGTCTCCGAGCTCCGGCCGGCCGGAGACGCC
TTCGACGGCATCAAGAAGCAGAGCTCCATGCTTTCGATCTACCAGCAGCAGAGGCAGTTTGGCCACAACAGCAGCCAGGC
CACGGCACAGCAGCAGTACCCTGCAGCGGCGCACAGGCAGCGCTCGCAGGTGGCGGATTACGGCGCGGCGGCACCGCATC
GTCTCCCTGGTGGTGGTGGTGGTGGATGCAGGCTAGTGCAGCCGGTGTCGGTGCGCCACCCTGTGCCGTTCCATCAGGCC
AACCTGATGGTCAGGTCATCAGTGTCGCAGAGCTTCCACAACCAGCAGCAGCCTTTCACCATGAGCAACAACGGGTTTGG
CGGCTCTACGGCCGGCGTGTACGGGGCGAGGAACCCACGGAACCAGGCCTCGACGCAGCTCACGATATTCTACAACGGCT
CGGTGAATGTTTTCGACAACGTCCCGGTCGACAAGGCTAAAGAGCTCATGATGCTGGCCAGCAGGGCGTCTGTTCCTGGT
GCTACCCCGTCAAGTGAGCCGGACTCGCCGCCTGTTCCTGCCCCTGCCCCTGCCAAGGTCAACGTGGCCGAGGTCTTCCC
TGGTGCTAGACAGATCGCAGTTCAGAAACCGGAGCCCTGCGTGCCGCATCTATCAAGCGCGGCAACTGCAAGCCCAGTCC
GCATCGTCGTGCCGCAAGCCGTGGCTCCCTCCAGGAGCACGTCTCACTGCGCTACAGAGGCCTGCGGTTCCAAGCCTGCT
GCTGCTCCTACCAGCCAGACAGTCTCGTCGTCCCGGCAGTTGGCAGCTGCAAGTGCTGCTGCAGCTGTTACGCCAAGAGC
TGTCCCTCAAGCTCGGAAAGCGTCGCTCGCCCGGTTCTTGGAGAAGCGAAAGGAAAGGACACTAGTCTTCATCCTCCTTG
TTTCTCTCCGCAGAGTGGCTAGCGTCGAGCCATACCTGACGTCCAACTCCAAGAGCCCGCTAGAGAGCAGTGACGCCGTC
GGCAGCGCTAGCGCTCCAACGAAATCGTCATCCACAGACGTCGCCCCGGCGAGCAGCCACAACGGCGGCGGCGCAGAGCT
TGTGCGCCATGGCGGATATCCCAGGAGCATCAGCTTCAGCACAAATTTGCAGATCTGA
>Zm00001d050365_P006 peptide
MERDFLGAIDRAWKCGEAAKEESRSEPDSPAATRWQFAARSGVAPAFMSFRTEATGEGSEAFSVSELRPAGDAFDGIKKQ
SSMLSIYQQQRQFGHNSSQATAQQQYPAAAHRQRSQVADYGAAAPHRLPGGGGGGCRLVQPVSVRHPVPFHQANLMVRSS
VSQSFHNQQQPFTMSNNGFGGSTAGVYGARNPRNQASTQLTIFYNGSVNVFDNVPVDKAKELMMLASRASVPGATPSSEP
DSPPVPAPAPAKVNVAEVFPGARQIAVQKPEPCVPHLSSAATASPVRIVVPQAVAPSRSTSHCATEACGSKPAAAPTSQT
VSSSRQLAAASAAAAVTPRAVPQARKASLARFLEKRKERTLVFILLVSLRRVASVEPYLTSNSKSPLESSDAVGSASAPT
KSSSTDVAPASSHNGGGAELVRHGGYPRSISFSTNLQI

ZmJAZ14 (TIFY19)

B73 RefGen_v3:
GRMZM2G064775 (zim29 - ZIM-transcription factor 29)
Chr5: 36785899..36787242

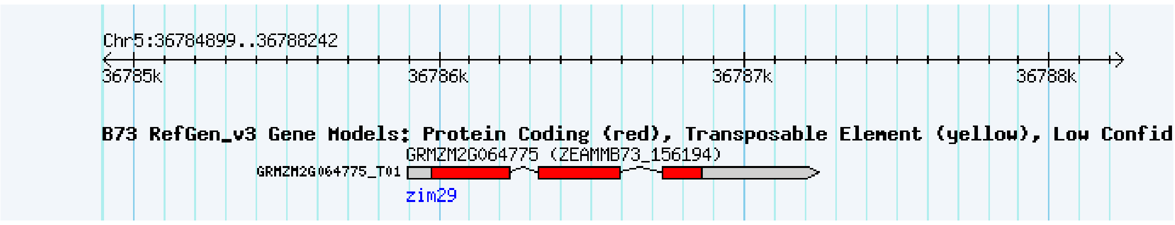

>GRMZM2G064775 Genomic DNA
GGCCTAAGCACCAGCACCAGCACCACCACCAGTGATCCGGCCAAGGTCCGTACCGACCAAAGACGGCCGGGGAGGAGATG

GCCGCCTCCGGGACCGGGAACAACAGGTTCGCCCATACATGCGCTCGCGTGCGGCAGTTCATGATGGAGCAGAACAGGCA
GGTGCGAATGGATGACCTCATCGGCTCCTCCTCGTCCCCGAGGCCACTGCAACTGACGCCAGTCCCTGTAGCGACGGGGC
CGCCGCCGGCGGCTGCCTGGGAGACCGACGCCGGCGCTAGGACTTTGCCGCTGTTCCCCGTCCGCAATAGCAGTAGCACG
GAGATCATCAGGTGAGCGAGCTCGAGTTATGCATTAATTGATCTGCTGGCTAGCTGACGTGTTCTTGGCCCGGCTTAATT
AATTTCTCGATCGGTTTGTTGCAGGCCAGAGCAGGAGGCCAAGGCCACCCTAACCATCTTCTACCAGGGCCAGGTGGCAA
CGTTCCACAACTTCCCGGCAGACAGAGCCAAGGACCTTATACAAATGGCAGGTTCTGTGACCGGGGAGGCGCCGGGGAAA
GGGGTGACGACGACGACCGCCGTGCCAGAAAAGGCCGGGACCAGTGGTGGTGAGCCGTCGGCTGCAGGGGTAGCTGGCAC
GCCGCCCATCGCAAGGAAGCTCACGCTACAGAGGTTCCTTAGGAAGAGGAAGGACAGGTGAGGACGAGAGATCACATGCG
TGCACTCTCTCACATCGGATTTGTCTTATCAGTAACTTCTTTCTCGATCGGTGCTGTTTGGAATTTTGCATAGAGCATTG
GATGATGACAATGCGCGTGTGTTTTTACTCGCAGGATCGCCGGCGCTGACGGCCGCGGCCACAATGAGGATGCTCCGCCG
CGGAAGAAGACAGACTCCGCTGGCGCCGGCGCCAACGACCCCGCCGAATACGTTCCTGATGAAGCCTCGTGGCTCAGGCT
CTGAAACCCGCTCTTCACTGCAAATTTGCAGGAGAATAATATGAATGAGAAGGGCAAGAGTGCTGGCATGCATGCCACAC
ATCCTCTAAAATGATACAATAATAGTGCCGTGTGTGCCTGTCAGAATTCCTAAGAATTCTTAGGCATTGTTTGAAAGGGC
TGTAACTTTTTTTTCTGAAAGCACCTTGCCGTGAAGCTTTTAGAACATCTCTAACAGGAGAGATGTATAGAACTCTAGCG
CAAAAAATTGCTTCAACGAGAGTTCTATTTTACAAAAATTCATCAAAATTATAGGACTCTGTCAGATATTGTTAACATTT
TATTTCCTAAATAATATGATATAGGACTCAGTTTTGTTGGAGCCGTAAATATTTTTATGGCCTT

>GRMZM2G064775_T01 cDNA

GGCCTAAGCACCAGCACCAGCACCACCACCAGTGATCCGGCCAAGGTCCGTACCGACCAAAGACGGCCGGGGAGGAGATG
GCCGCCTCCGGGACCGGGAACAACAGGTTCGCCCATACATGCGCTCGCGTGCGGCAGTTCATGATGGAGCAGAACAGGCA
GGTGCGAATGGATGACCTCATCGGCTCCTCCTCGTCCCCGAGGCCACTGCAACTGACGCCAGTCCCTGTAGCGACGGGGC
CGCCGCCGGCGGCTGCCTGGGAGACCGACGCCGGCGCTAGGACTTTGCCGCTGTTCCCCGTCCGCAATAGCAGTAGCACG
GAGATCATCAGGCCAGAGCAGGAGGCCAAGGCCACCCTAACCATCTTCTACCAGGGCCAGGTGGCAACGTTCCACAACTT
CCCGGCAGACAGAGCCAAGGACCTTATACAAATGGCAGGTTCTGTGACCGGGGAGGCGCCGGGGAAAGGGGTGACGACGA
CGACCGCCGTGCCAGAAAAGGCCGGGACCAGTGGTGGTGAGCCGTCGGCTGCAGGGGTAGCTGGCACGCCGCCCATCGCA
AGGAAGCTCACGCTACAGAGGTTCCTTAGGAAGAGGAAGGACAGGATCGCCGGCGCTGACGGCCGCGGCCACAATGAGGA
TGCTCCGCCGCGGAAGAAGACAGACTCCGCTGGCGCCGGCGCCAACGACCCCGCCGAATACGTTCCTGATGAAGCCTCGT
GGCTCAGGCTCTGAAACCCGCTCTTCACTGCAAATTTGCAGGAGAATAATATGAATGAGAAGGGCAAGAGTGCTGGCATG
CATGCCACACATCCTCTAAAATGATACAATAATAGTGCCGTGTGTGCCTGTCAGAATTCCTAAGAATTCTTAGGCATTGT
TTGAAAGGGCTGTAACTTTTTTTTCTGAAAGCACCTTGCCGTGAAGCTTTTAGAACATCTCTAACAGGAGAGATGTATAG
AACTCTAGCGCAAAAAATTGCTTCAACGAGAGTTCTATTTTACAAAAATTCATCAAAATTATAGGACTCTGTCAGATATT
GTTAACATTTTATTTCCTAAATAATATGATATAGGACTCAGTTTTGTTGGAGCCGTAAATATTTTTATGGCCTT

>GRMZM2G064775_P01 peptide

MAASGTGNNRFAHTCARVRQFMMEQNRQVRMDDLIGSSSSPRPLQLTPVPVATGPPPAAAWETDAGARTLPLFPVRNSSS
TEIIRPEQEAKATLTIFYQGQVATFHNFPADRAKDLIQMAGSVTGEAPGKGVTTTTAVPEKAGTSGGEPSAAGVAGTPPI
ARKLTLQRFLRKRKDRIAGADGRGHNEDAPPRKKTDSAGAGANDPAEYVPDEASWLRL

AGPv4:

Zm00001d014249 (zim29 - ZIM-transcription factor 29)

Chr5: 38005178..38006513

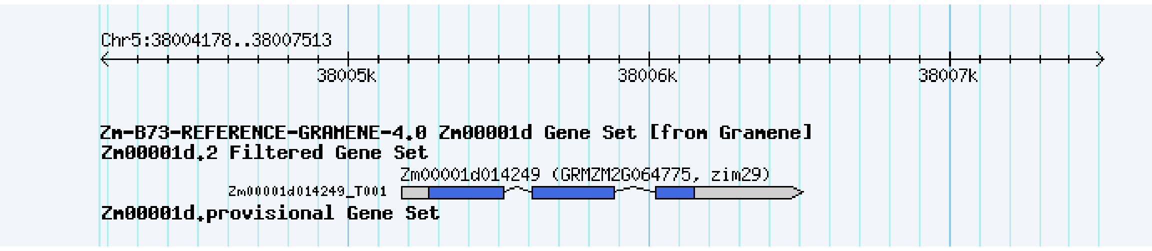

>Zm00001d014249 Genomic DNA
GCACAGAGAGAGGCCTAAGCACCAGCACCAGCACCACCACCAGTGATCCGGCCAAGGTCCGTACCGACCAAAGACGGCCG
GGGAGGAGATGGCCGCCTCCGGGACCGGGAACAACAGGTTCGCCCATACATGCGCTCGCGTGCGGCAGTTCATGATGGAG
CAGAACAGGCAGGTGCGAATGGATGACCTCATCGGCTCCTCCTCGTCCCCGAGGCCACTGCAACTGACGCCAGTCCCTGT
AGCGACGGGGCCGCCGCCGGCGGCTGCCTGGGAGACCGACGCCGGCGCTAGGACTTTGCCGCTGTTCCCCGTCCGCAATA
GCAGTAGCACGGAGATCATCAGGTGAGCGAGCTCGAGTTATGCATTAATTGATCTGCTGGCTAGCTGACGTGTTCTTGGC
CCGGCTTAATTAATTTCTCGATCGGTTTGTTGCAGGCCAGAGCAGGAGGCCAAGGCCACCCTAACCATCTTCTACCAGGG
CCAGGTGGCAACGTTCCACAACTTCCCGGCAGACAGAGCCAAGGACCTTATACAAATGGCAGGTTCTGTGACCGGGGAGG
CGCCGGGGAAAGGGGTGACGACGACGACCGCCGTGCCAGAAAAGGCCGGGACCAGTGGTGGTGAGCCGTCGGCTGCAGGG
GTAGCTGGCACGCCGCCCATCGCAAGGAAGCTCACGCTACAGAGGTTCCTTAGGAAGAGGAAGGACAGGTGAGGACGAGA
GATCACATGCGTGCACTCTCTCACATCGGATTTGTCTTATCAGTAACTTCTTTCTCGATCGGTGCTGTTTGGAATTTTGC
ATAGAGCATTGGATGATGACAATGCGCGTGTGTTTTTACTCGCAGGATCGCCGGCGCTGACGGCCGCGGCCACAATGAGG
ATGCTCCGCCGCGGAAGAAGACAGACTCCGCTGGCGCCGGCGCCAACGACCCCGCCGAATACGTTCCTGATGAAGCCTCG
TGGCTCAGGCTCTGAAACCCGCTCTTCACTGCAAATTTGCAGGAGAATAATATGAATGAGAAGGGCAAGAGTGCTGGCAT
GCATGCCACACATCCTCTAAAATGATACAATAATAGTGCCGTGTGTGCCTGTCAGAATTCCTAAGAATTCTTAGGCATTG
TTTGAAAGGGCTGTAACTTTTTTTTCTGAAAGCACCTTGCCGTGAAGCTTTTAGAACATCTCTAACAGGAGAGATGTATA
GAACTCTAGCGCAAAAAATTGCTTCAACGAGAGTTCTATTTTACAAAAATTCATCAAAATTATAGGACTCTGTCAGATAT
TGTTAACATTTTATTTCCTAAATAATATGATATAGGACTCAGTTTTGTTGGAGCCG
>Zm00001d014249_T001 cDNA
GCACAGAGAGAGGCCTAAGCACCAGCACCAGCACCACCACCAGTGATCCGGCCAAGGTCCGTACCGACCAAAGACGGCCG
GGGAGGAGATGGCCGCCTCCGGGACCGGGAACAACAGGTTCGCCCATACATGCGCTCGCGTGCGGCAGTTCATGATGGAG
CAGAACAGGCAGGTGCGAATGGATGACCTCATCGGCTCCTCCTCGTCCCCGAGGCCACTGCAACTGACGCCAGTCCCTGT
AGCGACGGGGCCGCCGCCGGCGGCTGCCTGGGAGACCGACGCCGGCGCTAGGACTTTGCCGCTGTTCCCCGTCCGCAATA
GCAGTAGCACGGAGATCATCAGGCCAGAGCAGGAGGCCAAGGCCACCCTAACCATCTTCTACCAGGGCCAGGTGGCAACG
TTCCACAACTTCCCGGCAGACAGAGCCAAGGACCTTATACAAATGGCAGGTTCTGTGACCGGGGAGGCGCCGGGGAAAGG
GGTGACGACGACGACCGCCGTGCCAGAAAAGGCCGGGACCAGTGGTGGTGAGCCGTCGGCTGCAGGGGTAGCTGGCACGC
CGCCCATCGCAAGGAAGCTCACGCTACAGAGGTTCCTTAGGAAGAGGAAGGACAGGATCGCCGGCGCTGACGGCCGCGGC
CACAATGAGGATGCTCCGCCGCGGAAGAAGACAGACTCCGCTGGCGCCGGCGCCAACGACCCCGCCGAATACGTTCCTGA
TGAAGCCTCGTGGCTCAGGCTCTGAAACCCGCTCTTCACTGCAAATTTGCAGGAGAATAATATGAATGAGAAGGGCAAGA
GTGCTGGCATGCATGCCACACATCCTCTAAAATGATACAATAATAGTGCCGTGTGTGCCTGTCAGAATTCCTAAGAATTC
TTAGGCATTGTTTGAAAGGGCTGTAACTTTTTTTTCTGAAAGCACCTTGCCGTGAAGCTTTTAGAACATCTCTAACAGGA
GAGATGTATAGAACTCTAGCGCAAAAAATTGCTTCAACGAGAGTTCTATTTTACAAAAATTCATCAAAATTATAGGACTC
TGTCAGATATTGTTAACATTTTATTTCCTAAATAATATGATATAGGACTCAGTTTTGTTGGAGCCG
>Zm00001d014249_P001 peptide
MAASGTGNNRFAHTCARVRQFMMEQNRQVRMDDLIGSSSSPRPLQLTPVPVATGPPPAAAWETDAGARTLPLFPVRNSSS
TEIIRPEQEAKATLTIFYQGQVATFHNFPADRAKDLIQMAGSVTGEAPGKGVTTTTAVPEKAGTSGGEPSAAGVAGTPPI
ARKLTLQRFLRKRKDRIAGADGRGHNEDAPPRKKTDSAGAGANDPAEYVPDEASWLRL

ZmJAZ15 (TIFY20)

B73 RefGen_v3:
GRMZM2G173596 (zim10 - ZIM-transcription factor 10)
Chr5: 36976140..36977007

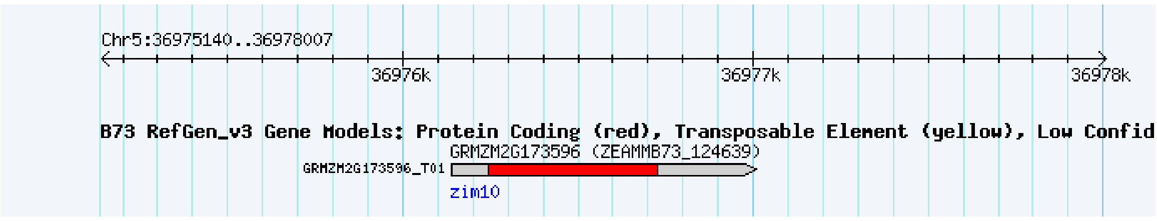

>GRMZM2G173596 Genomic DNA
ACCGAAACCCAAGCAACCAACAACGTCCGAGGCAAATCAAATTTCCATCAACCCTCCTCCTCCTCCATTCTCCAGAACAG
CAGCAGCCAGCAGGTCATCGCCAGTGATGGCGGCGGCGGCACCTTCTGGAGGCACAGGAAAGAACACAGCTGCGACTGCG
ACGACACCGAGCCGGTTCGCGGCGGCGTGCGGCGCGCTGAGCCAGTACGTCAAGGCGGCGGAGGCCGAGAGGACGCGCGC
GCGGCCGCCGGTGCGGCGGCCCCTGCCGCTCATGCCGGGCGCCGACGTCGACCAGGACGAGCCGGAAACGGCGGCGCAGC
TGATCATCGTGTACGGAGGGCGGGCGCTGGTGCTCGACGACGTCGCGGCGGACAAGGCGGCGGACCTGCTGCGCCTCGCC
GCGGCGGCGGCGGCGCGAGGAGGCACAGAGCAGCCGCTCTGCTCGCTCGCCGACCTACCCGTGGCCCGGAAGGCGTCGCT
GCAGCGGTTCATGGAGAAGCGCAAGGACAGGGTCGCCGCCCGCGCGGAGCCGTACCGCCGGCGCCGACCCGTCGGTGACC
GACGCAACGATCTCGCGCTCGAGCTGTGATTTGAGGATGTCTCGGCGGTAACTACTAGAGGACGCTGTGCTCAAGAAGCA
GCCGAACGATCTCTGATGCTTCGTACAGTCAGATCATATTAATTTGTTATAACTAGATAGGTATAGGTGGATTGTTCTTA
TGTGTTGTGCAGCAAAACAGAAGAAAAAACTTGGAGATGAAAAGACATGGTTTCGCATTACCTTTCTTTCGCCACTAAAA
ATGGTTCTTTTTATTTGTCTTGAGATTCTGTGACAAAGAGATATCTATAATAACGTAGTCTTGAGATT
>GRMZM2G173596_T01 cDNA
ACCGAAACCCAAGCAACCAACAACGTCCGAGGCAAATCAAATTTCCATCAACCCTCCTCCTCCTCCATTCTCCAGAACAG
CAGCAGCCAGCAGGTCATCGCCAGTGATGGCGGCGGCGGCACCTTCTGGAGGCACAGGAAAGAACACAGCTGCGACTGCG
ACGACACCGAGCCGGTTCGCGGCGGCGTGCGGCGCGCTGAGCCAGTACGTCAAGGCGGCGGAGGCCGAGAGGACGCGCGC
GCGGCCGCCGGTGCGGCGGCCCCTGCCGCTCATGCCGGGCGCCGACGTCGACCAGGACGAGCCGGAAACGGCGGCGCAGC
TGATCATCGTGTACGGAGGGCGGGCGCTGGTGCTCGACGACGTCGCGGCGGACAAGGCGGCGGACCTGCTGCGCCTCGCC
GCGGCGGCGGCGGCGCGAGGAGGCACAGAGCAGCCGCTCTGCTCGCTCGCCGACCTACCCGTGGCCCGGAAGGCGTCGCT
GCAGCGGTTCATGGAGAAGCGCAAGGACAGGGTCGCCGCCCGCGCGGAGCCGTACCGCCGGCGCCGACCCGTCGGTGACC
GACGCAACGATCTCGCGCTCGAGCTGTGATTTGAGGATGTCTCGGCGGTAACTACTAGAGGACGCTGTGCTCAAGAAGCA
GCCGAACGATCTCTGATGCTTCGTACAGTCAGATCATATTAATTTGTTATAACTAGATAGGTATAGGTGGATTGTTCTTA
TGTGTTGTGCAGCAAAACAGAAGAAAAAACTTGGAGATGAAAAGACATGGTTTCGCATTACCTTTCTTTCGCCACTAAAA
ATGGTTCTTTTTATTTGTCTTGAGATTCTGTGACAAAGAGATATCTATAATAACGTAGTCTTGAGATT
>GRMZM2G173596_P01 peptide
MAAAAPSGGTGKNTAATATTPSRFAAACGALSQYVKAAEAERTRARPPVRRPLPLMPGADVDQDEPETAAQLIIVYGGRA
LVLDDVAADKAADLLRLAAAAAARGGTEQPLCSLADLPVARKASLQRFMEKRKDRVAARAEPYRRRRPVGDRRNDLALEL

AGPv4:
Zm00001d014253 (zim10 - ZIM-transcription factor 10)
Chr5: 38196209..38196691

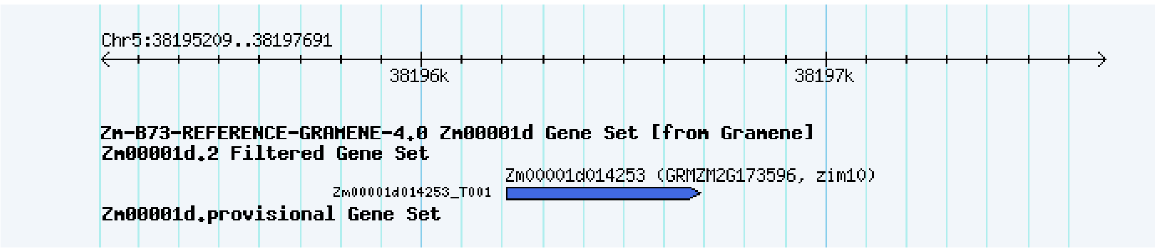

>Zm00001d014253 Genomic DNA
ATGGCGGCGGCGGCACCTTCTGGAGGCACAGGAAAGAACACAGCTGCGACTGCGACGACACCGAGCCGGTTCGCGGCGGC
GTGCGGCGCGCTGAGCCAGTACGTCAAGGCGGCGGAGGCCGAGAGGACGCGCGCGCGGCCGCCGGTGCGGCGGCCCCTGC
CGCTCATGCCGGGCGCCGACGTCGACCAGGACGAGCCGGAAACGGCGGCGCAGCTGATCATCGTGTACGGAGGGCGGGCG
CTGGTGCTCGACGACGTCGCGGCGGACAAGGCGGCGGACCTGCTGCGCCTCGCCGCGGCGGCGGCGGCGCGAGGAGGCAC
AGAGCAGCCGCTCTGCTCGCTCGCCGACCTACCCGTGGCCCGGAAGGCGTCGCTGCAGCGGTTCATGGAGAAGCGCAAGG
ACAGGGTCGCCGCCCGCGCGGAGCCGTACCGCCGGCGCCGACCCGTCGGTGACCGACGCAACGATCTCGCGCTCGAGCTG
TGA

>Zm00001d014253_T001 cDNA
ATGGCGGCGGCGGCACCTTCTGGAGGCACAGGAAAGAACACAGCTGCGACTGCGACGACACCGAGCCGGTTCGCGGCGGC
GTGCGGCGCGCTGAGCCAGTACGTCAAGGCGGCGGAGGCCGAGAGGACGCGCGCGCGGCCGCCGGTGCGGCGGCCCCTGC
CGCTCATGCCGGGCGCCGACGTCGACCAGGACGAGCCGGAAACGGCGGCGCAGCTGATCATCGTGTACGGAGGGCGGGCG
CTGGTGCTCGACGACGTCGCGGCGGACAAGGCGGCGGACCTGCTGCGCCTCGCCGCGGCGGCGGCGGCGCGAGGAGGCAC
AGAGCAGCCGCTCTGCTCGCTCGCCGACCTACCCGTGGCCCGGAAGGCGTCGCTGCAGCGGTTCATGGAGAAGCGCAAGG
ACAGGGTCGCCGCCCGCGCGGAGCCGTACCGCCGGCGCCGACCCGTCGGTGACCGACGCAACGATCTCGCGCTCGAGCTG
TGA

>Zm00001d014253_P001 peptide
MAAAAPSGGTGKNTAATATTPSRFAAACGALSQYVKAAEAERTRARPPVRRPLPLMPGADVDQDEPETAAQLIIVYGGRA
LVLDDVAADKAADLLRLAAAAAARGGTEQPLCSLADLPVARKASLQRFMEKRKDRVAARAEPYRRRRPVGDRRNDLALEL

ZmJAZ16

B73 RefGen_v3:
GRMZM2G338829 (zim9 - ZIM-transcription factor 9)
Chr6: 14014557..14015717

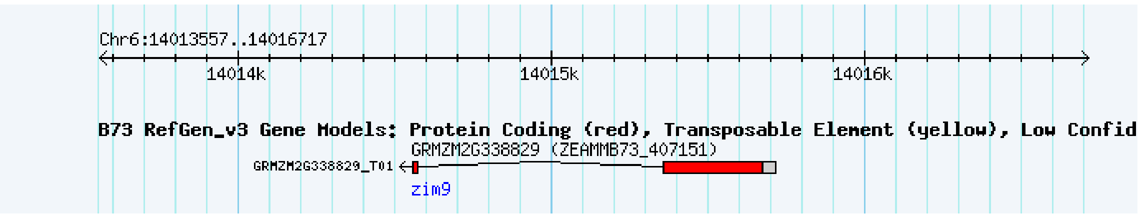

>GRMZM2G338829 Genomic DNA
CCTCCACCTCCGGGCGCCCCGACCACCACGACCCTGAGCCTGATGCCTGGCGCGGACGTCGGCGCCGACGAGCAGCAGGA
GCCGGCCGCCGCGAGGGCCGAGGAGATGCCCGAGCCTGCCCCCGCCCCCGCCGCGGCGCCGCCGCTCACCATCTTCTACG
GTGGCACGGTGGTGGTGTTCGAGGACTTCCCGGCGGAGAAGACGGCCGAGGTCATGCGCCCCGCGGCCGGCGACGACCTG
CCCATCGCCCGGAAGGCGTCGCTGCCGTGGTTCCTGACCAAGCGCAAGGACCGCCTCGTCGAGCGCGCGCCCTACGCCCG
CCCGTCGTCCCCCGCGAAGGAGCCGGAGAAGAAGACGGTAAAGCCAGCCTCGGCCTCAGCCTCATGCTCGACAAAATGAC
TGTGTGACTGCTGTTGCCTGAACCGAACTGGCCACCTCTACGCTCATTCCTCTCACGTTTCTGAAGGGTGCAGAGTAGCC

GGTCGACTAATCGAGCTCCCTACACCTCCTGCGCACGGCCTAAAACTCCCTGCTCTTGCCTGCACGCCTCGCTCTGCTTA
GCATCGGAGCGCACGGACGCTGCAGCCATGTTTATTCCTTGCAGCGAAACGCATGGGCTTGAAGGAGTTCAAAATGGGGT
ATGAATCATCTCAGTTTGACTTTTGTTTTAAATCCATTGATTATTGACCCTTATGTCTAAACTGCCCTAAGATACTTATT
TCTGGCTTCATAAATCACAAAACAGAGCCAAAGCTATAGTCAATGCAGAAAGTAAACCGAGCTAACAAATAAAAAACAAT
GCTACGACACACAAGATCAACAAGCTAGAGCCTAAATTAACATCAGTAATTCAAATCCACTTAAGTAATATAGTGTGTGA
TGTAAGCCAAAAAAACCAGATGCAACCATTCTAGCCAAAGACTCTTTAGCCCATAAGTAATTCATGTTTTTGTAGATGCT
CGACATGGTTCTTGATGATCTATAGAATGAGCACCCTAATCTCTGTTCTCCTCGGTTCCCGCGTGTGTGGAAAAGCAGCG
GTGAGGTTCAATAACTGGTTACACATTGTTTTTGATTGCTCAGTGCAACCATGATGTTTGATGAAATGTTCAACTCTGC
TATTGCACATTTGCACGCTGCAGTGCAGTGTTGAAATGTAG

>GRMZM2G338829_T01 cDNA
CCTCCACCTCCGGGCGCCCCGACCACCACGACCCTGAGCCTGATGCCTGGCGCGGACGTCGGCGCCGACGAGCAGCAGGA
GCCGGCCGCCGCGAGGGCCGAGGAGATGCCCGAGCCTGCCCCCGCCCCCGCCGCGGCGCCGCCGCTCACCATCTTCTACG
GTGGCACGGTGGTGGTGTTCGAGGACTTCCCGGCGGAGAAGACGGCCGAGGTCATGCGCCCCGCGGCCGGCGACGACCTG
CCCATCGCCCGGAAGGCGTCGCTGCCGTGGTTCCTGACCAAGCGCAAGGACCGCCTCGTCGAGCGCGCGCCCTACGCCCG
CCCGTCGTCCCCCGCGAAGGAGCCGGAGAAGAAGACGTGCAGTGTTGAAATGTAG

>GRMZM2G338829_P01 peptide
MPGADVGADEQQEPAAARAEEMPEPAPAPAAAPPLTIFYGGTVVVFEDFPAEKTAEVMRPAAGDDLPIARKASLPWFLTK
RKDRLVERAPYARPSSPAKEPEKKTCSVEM

AGPv4:

Zm00001d035382 (zim9 - ZIM-transcription factor 9)

Chr6: 23840275..23852509

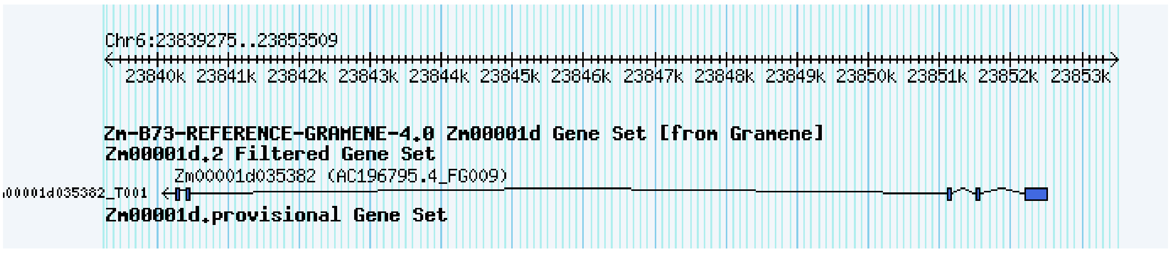

>Zm00001d035382 Genomic DNA
ATGCCTGGCGCGGACGTCGGCGCCGACGAGCAGCAGGAGCCGGCCGCCGCGAGGGCCGAGGAGATGCCCGAGCCTGCCCC
CGCCCCCGCCGCGGCGCCGCCGCTCACCATCTTCTACGGTGGCACGGTGGTGGTGTTCGAGGACTTCCCGGCGGAGAAGA
CGGCCGAGGTCATGCGCCCCGCGGCCGGCGACGACCTGCCCATCGCCCGGAAGGCGTCGCTGCCGTGGTTCCTGACCAAG
CGCAAGGACCGCCTCGTCGAGCGCGCGCCCTACGCCCGCCCGTCGTCCCCCGCGAAGGAGCCGGAGAAGAAGACGGTAAA
GCCAGCCTCGGCCTCAGCCTCATGCTCGACAAAATGACTGTGTGACTGCTGTTGCCTGAACCGAACTGGCCACCTCTACG
CTCATTCCTCTCACGTTTCTGAAGGGTGCAGAGTAGCCGGTCGACTAATCGAGCTCCCTACACCTCCTGCGCACGGCCTA
AAACTCCCTGCTCTTGCCTGCACGCCTCGCTCTGCTTAGCATCGGAGCGCACGGACGCTGCAGCCATGTTTATTCCTTGC
AGCGAAACGCATGGGCTTGAAGGAGTTCAAAATGGGGTATGAATCATCTCAGTTTGACTTTTGTTTTAAATCCATTGATT
ATTGACCCTTATGTCTAAACTGCCCTAAGATACTTATTTCTGGCTTCATAAATCACAAAACAGAGCCAAAGCTATAGTCA
ATGCAGAAAGTAAACCGAGCTAACAAATAAAAAACAATGCTACGACACACAAGATCAACAAGCTAGAGCCTAAATTAACA
TCAGTAATTCAAATCCACTTAAGTAATATAGTGTGTGATGTAAGCCAAAAAAACCAGATGCAACCATTCTAGCCAAAGAC
TCTTTAGCCCATAAGTAATTCATGTTTTTGTAGATGCTCGACATGGTTCTTGATGATCTATAGAATGAGCACCCTAATCT
CTGTTCTCCTCGGTTCCCGCGTGTGTGGAAAAGCAGCGGTGAGGTTCAATAACTGGTTACACATTGTTTTTGATTGCTC
AGTGCAACCATGATGTTTGATGAAATGTTCAACTCTGCTATTGCACATTTGCACGCTGCAGTGCAGTGTTGAAATGTAGT
TTACTTTTTGTTTGAGAATTCACATGACAGGTCAGTTTGTACATTTTTAAGAGTCCAAATTAGGTTCGTTCTGTATAATA

CATGTGGTGTTTAATACCATTGTATTCCGAAAATGATATGAGCTTACCTTGCTGTATCTCCATGTATATCATGAAAATTT
ATTTAAAATTGGACGCTTGATTGATTGAAAGACCTAATGAATTGCTATTTGTCTTTGTTTTCAAAGGGATGGCCTATGAC
CCTTTAAGAACTCATCTAGAGCGAAGAGCAGCTGAGCAGGTAATTACTCTGTCTCGATTTGTTTTGAACGCATAATATAA
TCTATACATCAGAACCTAGCAAGCTTAAGGAAAACAGTCAAATTCGCGGTAATTACATAGATAAGTCCATACTTTTGTGA
AACCTGTAGCAAAAATAGAATAAAAAATATTAGATCTGTCATGTGATGTTCTAACAATTATGATAGTCGCCTAGAGGGGG
GGTGAATAGGGCGAAACTGAAATTTACAAATATAAACACAACTACAAGCCGGGGTTAGCGTTAGTAATAAAGAAATGAGT
CCGCGAGAGAGGGCGCAAAACAAATCCCAAGCGAATAAGCAAGTAAGACACGGAGATTTGTTTTACCGAGGTTCGGTTCT
TGCAAACCTACTCCCCGTTGAGGAGGCCACAAAGGCCGGGTCTCTTTCAACCCTTCCCTCTCTCAAACGATCCACGGATC
GAGTGAGCTTTCTTTTCTCAATCACTTGGAACACAAAGTTCCCGCAAGGGCCACCACACAATTGGTGCCTCTTGCCTCGA
TTACAAGTGAGTGTTTGATGACAATGAAAGAGTCAAGAAGAAAGAAAGCGATCCAAGCGCAAGAGCTCGAAAGAACACAA
GCAAATCTCTCTCGCTAATCACTAAAGCGTTGTGTGGAGTTGGAGAGGATTTGATCTATTTGGTGTGTCTAGAATTGAAT
GCTAGAGCTCTTGTAGTAGTTGAGAGGTGGAAAACTTGGATGCAATGAATGGTGGGGTGGTTGGGGTATTTATAGCCCCA
ACCACCAAAAGTGGCCGTTGGGGAGCCAGTCTGCTCGATGGCGCACCAGACAGTCCGGTGCACACCGGACATGTCCGGTG
CCCCTACCACGTCATCAGTGCCGTTGGAATCTGACCGTTGGAGTTCTGACTTGTGGGCCACCCTTGATGTCCGGTGGCGC
ACCGGACAGCTCCTGTTCATTGTTCGGTGCGCCAGTATGGGCAAACCTGACGTCTGCGCGCGCTGCGGCACATTTATTGC
TTCTGCAGGTAGCCGTTGGCGCGGGATAGCCGTTGCGCTGGAGTCACACCGGACAGTCCGGTGCACACCGGACATGTCCG
GTGAATTATAGCGGACGAGCCGTTGGCTTTTCCCGAAGCTGAAGAGTTCCTGAGGCCGCTCCTCCATGGCGCACCGGACA
CTGTCCGGTGTACACCGGACAGTCCGGTGAATTATAGCGCGAGTGCCTCTAGAAATTCCCGAAGGTGACGAGTTTGAGCT
GGAGTCCCCTGGTGCACCGGACACTGTCCGGTGGCACACCGGACAGTCCGGTGCGCCCAGACCAGAGGGCCTTCGGTTGG
CTCTTCGCCCTTTTTGTTGAACCCAACACTTGGTTATTTTATTGGCTATGTTGTGAACCTTTGACACCTGTATAACTTAT
ACACTAGAACAAACTAGTTAGTCCAAAGACTTGTGTTGGGCAATTCAACCACCAAAATCAATTAGGGACTAGGTGTAAGC
CTAATTCCCTTTCAATCTCCCCCTTTTTGGTGATTGATGCCAACACAAGCCAAAACAAATATAGAGGTGCATAATTGAAC
TAGTTTGCATAATATAAGTGCAAAGGTTGCTTGGAATTGAGCCAATATAACTACTTCACAAGATATGCAAGGATTGTTTC
TTACTTATAACATTTTGGACCACGCTTGCACCACATGTTTTGTTTTTGCAAAAATCTTTTGTAAATCTTTTCTAAGTTAT
TTTGCAAATAGTCAAAGGTAAATGAATAAGATTTGGTAAAGCATTTTCAAGATTTGAAATTTTCTCCCCCTATTTCAAAT
GCTTTTCCTTTGACTAAACAAAACTCCCCCTAAATAAAATCCTCCTCTTAATGTTCAAGAGGGTTTTGAGATATGATTTT
GAAATACTACTTACTCCCCTTTTGAACACAAGAAGATACCAAATTGACAATTTGAAAACTTTAAAAACCATTTGGTGGTG
CGGTCCTTTTGCTTTGGGCTCTGTCTCTCTCCCCCTTTGGCATGAATCGCCAAAAACGGAATCATTAGAGCCCTTTAGCT
AATTTCTCCTCCTTTGGTCGTAAATAAAAGAGAGAAGATTATACCATAGTAGGAGTCCTTTTGCTTTGCTCTTTCTCCCC
CAAGGATAGAGAGCGGCTTGGAGTGCCGGCGAAGGATGAGTAGTGGAGTGGAAGCCTTTGTCTTCGCCGAAAACTCCAAT
TCCCTTTCAATACACCTATAACTTGGTTTGAAATAGACTTGAAAACACATTAGTCATAGCATATTAAAGAGACATGATCA
AAGGTATATTCATGAGCTATGTGTGCAAGTTAGCAAAAGAAATTTCTAGAATCAAGAATATTGAGCTCATGCCTAAGTTT
GGTAAAGGTTTGTTCATCAAGTGGCTTGGTAAAGATATCGGCTAATTGATCTTTAGTGTTAATGTAAGAAATCTCGATAT
CTCCCTTTTGTTGGTGATCCCTAAGAAAATGATACCGAATGGCTATGTGCTTAGTGCGGCTGTGCTCGACGGGATTGTCG
GCCATTTTGATTGCACTCTCATTATCACATAGCAAAGGGACTTTGATTAATTTGTAACCGTAGTCCCGCAGGGTTTGCCT
CATCCAAAGCAATTGCGCGCAACAATGACCTGCGACAATGTACTCGGCTTCGGCGGTGGAAAGAGCGACTGAATTTTGCT
TCTTTGAAGCCCATGACACCAAGGATCTTCCCAAGAACTGGCAGGTCCCTGATGTGCTCTTCCTATTAATTTTACACCCC
GCCCAATCGGCATCCGAATATCCAATCAAATCAAATGTGGATCCCCGAGGGTACCAAAGCCCAAACTTAGGAGTATAAGC
CAAATATCTCAAGATTCGTTTTACAGCCGTAAGGTGGGATTCCTTAGGGTCGGATTGGAATCTTGCACACATGCAAACGG
AAAGCATAATGTCCGGTCGAGATGCACAAAGATAAAGTAATGAACCTATCATCGACCGGTATACCTTTTGATCCACGGAC
TTACCTCCCGTGTCGAGGTCGAGATGCCCATTAGTTCCCATGGGTGTCTTGATGGGTTTGGCATCCTTCATCCCAAACTT
GGTTAGAATGTCTTGAGTGTACTTCGTTTGGCTGATGAAGGTGCCCTCTTGGAGTTGTTTGACTTGGAATCCTAGAAAAT
ACTTCAACTCCCCCATCATAGACATCTCGAATTTTTGTGTCATGATCCTACTAAATTCTTCACAAGTAGATTCGTTAGTA
GACCCAAATATGATATCATCAATATAAATTTGGCATACAAACAAATCATTGTCAAGAGTTTTAGTGAATAGAGTAGGATC

GGCCTTTCCGACTTTGAAGCCGTTAGCAATAAGAAAATCTCTAAGGCATTCATACCATGCTCTTGGGGCTTGCTTGAGCC
CATAAAGCGCCTTAGAGAGCTTATAGACATGGTTAGGGTACTCACTGTCTTCAAAGCCGGGAGGTTGCTCAACATAGACC
TCCTCCTTGATTGGTCCATTGAGGAAAGCACTTTTCACGTCCATTTGATAAAGCTTAAAGCCATGGTAAGTAGCATAGGC
TAATAATATGCGAATTGACTCAAGCCTAGCTACGGGTGCATAGGTTTCACCGAAATCCAAACCTTCGACTTGGGAGTATC
CCTTGGCCACAAGTCGTGCTTTGTTCCTTGTCACCACACCATGCTCATCTTGCTTGTTGCGGAAGACCCATTTGGTTCCT
ACAACATTTTGATTAGGACGTGGAACTAAATGCCATACCTCGTTCCTTGTGAAATTGTTGAGCTCCTCTTGCATCGCCAC
CACCCAATCCGAATCTTGAAGTGCCTCCTCTACCTTGTGTGGCTCAATAGAGGAAACAAACGAGTAATGTTCACAAAAAT
GAGCAACACGAGATCTAGTTGTTACCCCCTTATGAATGTCGTCGAGGATAGTGTCGACGGGGTGATCTCGTTGGATTGCT
TGGTGGACTCTTGGGTGTGGCGGCCTTCGCTCTTCATCCTCCTTGTCTTCTTCATTTGCATCTCCTCCTTGATCATTGCC
ATCATCTTGAGGTGGCTCAATTGCTTGATCTTCAACTTCATCAACTTGAGCTTCATCCTCATTTTGAGTTGGTGGAGATG
CTTGCATGGAGGAGGATGGTTGATCTTGTGCATTTGGAGGCTCTTCGGATTCCTTAGGACACACATCCCCAATGGACATG
TTCCTTAGCGCTATGCACGGAGCCTCTTCATTACCTATCTCATCAAGATCAACTTGCTCTACTTGAGAGCCGTTAGTTTC
ATCAAACACAACGTCACATGAGACTTCAACTAGTCCAGTGGACTTGTTAAAGACCCTATATGCCCTTGTGTTTGAGTCAT
AACCAAGTAAAAAGCATTCTACAGTTTTAGGAGCAAATTTAGATTTTCTACCTCTTTTAACAAGAATAAAGCATTTGCTA
CCAAAAACTCTAAAATATGAAATGTTGGGCTTTTTACCGGTTAGGAGTTCATATGATGTCTTCTTGAGGATTCGGTGTTA
GATACAACCGGTTGATGGCGTAGCAAGCGGTGTTGACCGCCTCAGCCCAAAACCAATCTGATGTCTTGTACTCATTAAGC
ATGGTTCTTGCCATGTCCAATAGAGTTCGATTCTTCCTCTCTACTACACCATTTTGTTGAGGTGTGTAGGGAGAAGAGAA
CTCATGCTTGATGCCCTCCTCCTCAAGAAAGCCTTCAATTTGTGAGTTCTTGAACTCCGTCCCGTTGTCGCTTCTTATTT
TCTTGATCCTTAATCCAAACTCATTTTGAGCCCGTCTCAAGAATCCCTTTAAGATCTCTTGGGTTTGAGATTTTTCCTGT
AAAAAGAATACCCAAGTGAAGCGAGAATACTCATCCACAATAACTAGACAGTACTTACTCCCGCCGATGCTTATGTAAGC
AATCGGGCCGAATAGATCCATGTGTAGGAGCTCCAGTGGCCTGTCCGTTGTCATGATGTTCTTGTGTGGATGATGAGCAC
CAACTTGCTTCCCTGCCTGGCATGCGCTACAAATCCTGTCTTTCTCAAAATGAACATTTGTTAATCCTAAAATGTGTTCT
CCCTTTAGAAGCTTATGAAGATTCTTCATCCCAACATGTGCTAGTCGACGGTGCCAGAGCCAGCCCATGTTAGTCTTAGC
AATTAAGCATGTGTCGAGTTCAGCTCTATCAAAATCTACTAAGTATAGCTGACCCTCTAACACACCCTTAAATGCTATTG
AATCATCACTTCTTCTAAAGACAGTGACACCTATATCAGTGAATAGACAGTTGTAGCCCATGTGACATAATTGAGAAACA
GAAAGCAAGTTGTAATCTAAAGAATCTACAAGAAAAACATTGGAAATAGAATGGTCAGGAGATATAGCAATTTTACCAAG
ACCTTTGACCAAACCTTGATTTCCATCCCCGAATGTGATAGCTCGTTGGGGATCTTGGTTTTTCTCATATGAGGAGAACA
TCCTTTTCTCCCCTGTCATGTGGTTTGTGCACCCGCTGTCGAGTATCCAACTTGAGCCCCCGGATGCATAAACCTACAAA
ACAATTTTAGTTCTTGACTTTAGGTACCCAAATGGTTTTGGGTCCTTTGGCATTAGAAACAAGAACTTTGGGTACCCAAA
CACAAGTCTTTGACCCCTTGTGCTTGCCCCCAACATATTTGGCAACTATTTTGCCGGATTTGTTAGTTAAAACATAAGAT
GCATCAAAAGTCTTAAATGAAATGCTATGTTCATTTGATGCATTAGGAGTTTTCTTCTTAGGCAACTTAGCACGGGTTGG
TTGCCTAGAACTAGATGTCTCACCCTTATACATAAAAGCATGATTAGGTCCAGAGTGAGACTTCCTAGAATGAATTCTCC
TAATTTTGCTCTCAGGATAGCCGGCAGGGTACAAAATGTAACCCTTGTTATCCTGAGGCATGGGAGCCTTGCCCTTTACA
AAATTTGACAATCTTTTAGGAGGGGCACTAAGTTTGACATTGTCTCCCCTTTGGAAGCCAATGCCATCCTTGATGCCAGG
GCGTCTCCCATTATAGAGCATACTTCTAGCAAATTTAAATTTTTCATTTTCTAAGTTATGCTCGGCAATTTTAGCATCTA
ATTTAGCTATATGATCATTTTGTTGTTTAATTAAAGCCATGTGATCATATATAGCATCAATGTTAAAATCTCTACATCTA
GTACAAATAGAAACATGCTCAACGGTAGATGTAGAGGGTTTGCAAGAATTAAGTTCAACAATCTTAGCATGAAGTAAATT
ATTCTTATCTCTAAGATTGGAAATTGTAATATTGCAAACATCTAGTTCCTTAGCCTTAGCAATTAAATTTTCATTTTCTA
CTCTAAGGCTAGCAAGAGAAATGTTTAATTCTTCAATCCTAGCAAGCAAATCATCATTATTATCCCTAGGATTGGGAATT
GAAACATTACAAACATGTGAATCAACCTTAGCATTTAAACTAGCATTTTCATTCCTAAGGTTGTCAATCAATTCATGGCA
AGTGCTTAGCTCACTAGATAATCTTTCACTTTTTTCTACTTCTAGAGCATAAGCATTTTTAACCTTAACATGCTTCTTGT
TTTCTTTAATAAGGAAGTCCTCTTGAGAATCCAAGAGATCATCCTTCTTATGAATAGCACTAATTAATTCATTCAATTTT
TCCTTTTGTTGCATGTTGAGGTTGGCAAAAAGGGTACGTAAATTGTCTTCCTCATCACTAACATTATCATCACTAGATGT
TTCATATTTAGTGGAGGATCTTGATTTTACCTTCTTTTTGCCGTCCTTTGCCATGAGGCACTTGTGGCCGATGTTGGGGA

AGAGGAGTCCCTTGGTGACGGCGATGTTGGCGGCGTCCTCGTCGTCGGAGGAGTCGCTTGAGCTTTCGTCGGAATCCCAC
TCCCGACAAACATGGGCATCGCCGCCCTTCTTCTTGTAGTATCTCTTCTTTTCTCTCCTCTTGCCCTTCTTGTCGTTGTC
CCTGTCACTATCACTTGATAATGGACATTTTGCAATAAAGTGACCGGGCTTACCACACTTGTAGCACACTTTCTTGGAGC
GGGACTTGTAGTCCTTCCCTTTCCTTTGCTTGAGGATTTGGCGGAAGCTCTTGATGACGAGCGCCATTTCCTCATTGTCG
AGCTTGGAGGCGTCAATTGGTTGTCTACTTGGTGTAGGCACCTCCTTCTTTTCTTCCGTCGCCTTGAATGCGACGGGTTG
AGCTTCGGATGGGTCACCAAGCTCGTTGATCTTCCTCGAGCCTTCTATCATGCACTCAAAACTTACAAAATGCCCGATAA
CTTCCTCGGGAGTCATTTTAGTATATCTAGGATTACCACGAATTAATTGAACTTGAGTGGGATTAAGAAAAATGAGAGAT
CTTAGAATAACATTCACCACTTCGTGGTCGTCCCACTTCTTGCTCCCGAGGTTGCGCACTTGGTTCACCAAGGTCTTGAG
CCGGTTGTACATGTGTTGTGGCTCCTCCCCTTTGTGAAGTCGGAACCGACCGAGCTCCCCCTCGATCGTTTCCCGCTTGG
TGATCTTGGTGAGCTCGTCTCCCTCATGCGCGGTTTTGAGTACATCCCAAACTTCCTTGGCGTTCTTCAACCCTTGTACT
TTGTTATACTCCTCTTTACTTAGTGAGGCGAGGAGTATCGTTGTGGCTTGAGAGTTGAAGTGCTCGATTTGGGCCACCTC
ATCCTCATCGTAGTCTTCATCCCCTACGGACGGTACCTGCGCACCAAACTCAACAACATCCCATATACCTTTGTGGAGCG
AGGTTAGATGAAATCGCATTAAATCGCTCCACCTAGCGTAATCTTCACCATCAAAAGTTGGTGGTTTGCCTAATGGGACG
GAAAATAAAGGTGTATGTTTGGAAATGCGAGGGTAGCGTAGGGGGATCTTACTATACTTCTTGCGCTCTTGGCGCTTAGA
AGTGACGGATGTCGTGTCGGAGCCGGAGGTGGATGGTGATGAAGTGTCGGTCTCGTAGTAGACCACCTTCCTCATCTTCT
TGTGCTTGTCCCCACTCCGATGCGGCTTGTGGGAAGAAGATTTTTCCTTCTTCTCTTTGTGGTGAGAAGAAGATTTCTTC
TCCTTCCCTTTGTTGGAGGAGATCTTCTTCTTCTCCTTTCTCTTGGTGCGGGACTCTTCCGATGAAGTGCTCCCGTAGCT
TGTAGTGGGCTTTTCGCCGGTCTCCATCTCCTTCTTGGCGTGATCTCCCGACATCACTTCGAGCGGTTAGGCTCTAATGA
AGCAACGGGCTCCGATACCAATTGATAGTCGCCTAGAGGGGGGGGGGTGAATAGGGCGAAACTGAAATTTACAAATATAA
ACACAACTACAAGCCGGGGTTAGCGTTAGTAATAAAGAAATGAGTCCGCGAGAGAGGGCGCAAAACAAATCCCAAGCGAA
TAAGCAAGTAAGACACGGAGATTTGTTTTACCGAGGTTCGGTTCTTGCAAACCTACTCCCCGTTGAGGAGGCCACAAAGG
CCGGGTCTCTTTCAACCCTTCCCTCTCTCAAACGATCCACGGATCGAGTGAGCTTTCTTTTCTCAATCACTTGGAACACA
AAGTTCCCGCAAGGGCCACCACACAATTGGTGCCTCTTGCCTCGATTACAAGTGAGTGTTTGATGACAATGAAAGAGTCA
AGAAGAAAGAAAGCGATCCAAGCGCAAGAGCTCGAAAGAACACAAGCAAATCTCTCTCGCTAATCACTAAAGCGTTGTGT
GGAGTTGGAGAGGATTTGATCTATTTGGTGTGTCTAGAATTGAATGCTAGAGCTCTTGTAGTAGTTGAGAGGTGGAAAAC
TTGGATGCAATGAATGGTGGGGTGGTTGGGGTATTTATAGCCCCAACCACCAAAAGTGGCCGTTGGGGAGCCAGTCTGCT
CGATGGCGCACCGGACAGTCCGGTGCACACCGGACATGTCCGGTGCCCCTACCACGTCATCAGTGCCGTTGGAATCTGAC
CGTTGGAGTTCTGACTTGTGGGCCACCCTTGATGTCCGGTGGCGCACCGGACAGCTCCTGTTCATTGTCCGGTGCGCCAG
TATGGGCAAACCTGACGTCTGCGCGCGCTGCGGCGCATTTATTGCTTCTGCAGGTAGCCGTTGGCGCGGGATAGCCGTTG
CGCTGGAGTCACACCGGACAGTCCGGTGCACACCGGACATGTCCGGTGAATTATAGCGGACGAGCCGTTGGCTTTTCCCG
AAGCTGAAGAGTTCCTGAGGCCGCTCCTCCATGGCGCACCGGACACTGTCCGGTGTACACCGGACAGTCCGGTGAATTAT
AGCGCGAGTGCCTCTAGAAATTCCCGAAGGTGACGAGTTTGAGCTGGAGTCCCCTGGTGCACCGGACACTGTCCGGTGGT
GCACCGGACACTGTCCGGTGGCACACCAGACAGTCCGGTGCGCCCAGACCAGAGGGCCTTCGGTTGGCTCTTCGCCCTTT
TTGTTGAACCCAACACTTGGTCATTTTATTGGCTATGTTGTGAACCTTTGACACCTGTATAACTTATACACTAGAACAAA
CTAGTTAGTCCAAAGACTTGTGTTGGGCAATTCAACCACCAAAATCAATTAGGGACTAGGTGTAAGCCTAATTCCCTTTC
AAATTACACATCTTTCCCTTATTAAGTGCCCATTTTTCCACAAGATGCTCACCTGCATGGCTACAGGAAAATCATCCTAA
AAATCTAACAATAATGTTGATGCCATTGACGAGAAATTTTCACTAAGGTGTGGTCACAGTCATGGCTATCATGTAGGTGT
TTAGCTGCTGTTGCATTAAGTCTAGATTTTAAGAATCCTAATGTCTAATCATTTCGATAGAAAAGGAACCACAAGTCTTA
AAACACCTTACTATGTTGCTTAGTTAGTATACCGTAATATATGTGTCGATAGGAGTTAGGATATAATTTTGTGAGATTTC
TGATAATCTTTTGAGTTGCCAATATGCAATTTGATTGCTTTGTTTACAATAAGTGGTATATAAGTTATGCTATGTTTCTT
CCATGAATCCCGTGCCATCGATTATACTAGCAAAGAACTTTCTAACAACTATAGATATTACTTCAACTATTATTGGTTTC
ATTTCATACAATTTTGGCAATACCAACAATTAATTACTGAAGTTATTTATGGTTCCATGGCACTAAATAAGTGTCATAGA
ATTTTAGTTTGCATAACTTCAGTTGTTTAAAATTGGGAACCCATTGACCATGCAGATTTTTGTAAACCAACTTGACTCCC
CTGCATTGCTATCCCACTAAATAATTATGGTACTCGAAAAGTTAATGTCAAATCTAGGTACTAAAATCACACTATCTCTT

AATCTCCTAACAACTTTAAGAATAAAGGACTCCAAAACTCCATCTTTTACCTGACAAAGGAAGTAGGGAAGGAGAAAAGG
CTGGACAAAATGGTGATCCGGGTTGTTTTTTACTTTACCATCAACGTTTATCAATTACTCCATTCCAAATTATAAGACGC
CTTGAAATAGAATGCTTCAAGTTAAAGTTAGCTATTGTTTGCATGCTTCTTTATTTATATTTTATTGTCCAATTTCCCTG
ACTATTGGGTTAATTTCTGTCTCAATCTGTTAATTTTTTTCTTCTGTTTTGTGACACAGTTGGATATATTCAAGGAAATG
GTATCTTTGCGTGTATTTGAGAGGTACATGATAAGTGTAATGGTTTCACGTTGTCTGCAGATTTTGTTTGGATATTCACT
AGATGAGTAATTTCACCATGGTTTAATTCTAACAGTTGCTCTTATTACATCCAAGACTGCCAAGTTCTACTGTAA
>Zm00001d035382_T001 cDNA
ATGCCTGGCGCGGACGTCGGCGCCGACGAGCAGCAGGAGCCGGCCGCCGCGAGGGCCGAGGAGATGCCCGAGCCTGCCCC
CGCCCCCGCCGCGGCGCCGCCGCTCACCATCTTCTACGGTGGCACGGTGGTGGTGTTCGAGGACTTCCCGGCGGAGAAGA
CGGCCGAGGTCATGCGCCCCGCGGCCGGCGACGACCTGCCCATCGCCCGGAAGGCGTCGCTGCCGTGGTTCCTGACCAAG
CGCAAGGACCGCCTCGTCGAGCGCGCGCCCTACGCCCGCCCGTCGTCCCCCGCGAAGGAGCCGGAGAAGAAGACGAATGA
GCACCCTAATCTCTGTTCTCCTCGGTTCCCGCGTGTGTGGAAAAGCAGCGGGATGGCCTATGACCCTTTAAGAACTCATC
TAGAGCGAAGAGCAGCTGAGCAGTTGGATATATTCAAGGAAATGGTATCTTTGCGTGTATTTGAGAGTTGCTCTTATTAC
ATCCAAGACTGCCAAGTTCTACTGTAA
>Zm00001d035382_P001 peptide
MPGADVGADEQQEPAAARAEEMPEPAPAPAAAPPLTIFYGGTVVVFEDFPAEKTAEVMRPAAGDDLPIARKASLPWFLTK
RKDRLVERAPYARPSSPAKEPEKKTNEHPNLCSPRFPRVWKSSGMAYDPLRTHLERRAAEQLDIFKEMVSLRVFESCSYY
IQDCQVLL

ZmJAZ17

B73 RefGen_v3:
GRMZM2G126507 (zim1 - ZIM-transcription factor 1)
Chr7: 108871320..108874327

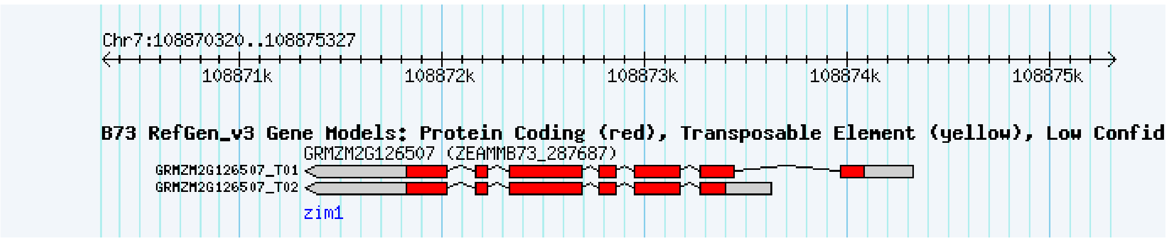

>GRMZM2G126507 Genomic DNA
AAGCCATTAAACAAACACAAATAGCACACACGGGCACACGGCAGGCCTACAACTACAAGAGGAGAGGAGAAGAGAGAGGA
GAATTTTCAATGCTGCCGCGCACCGTCATCACTAGCTAGTGGAGGGGCGGACGAGAAGGCGAGGAGCTGAGGGGAGAGAG
GCTTCACAGAGCTTGCTTGAGCCTGGAGGGGGCTGCCGCTGTTTGTCTCTCCACTCCACCCACCAGCAGTTACCTCGGGA
GGGCATGGCAAAATCTGGTGCCTCCTTTCCGGAGTCCAGTTGGATGGAGAGGGATTTCCTGGCCGCGATCGGCAAGGAGC
AGCAGCACCCGCACAAGGAGGAGGCAGGCGCGGAGGAATCTGGTATGCTCCCGGCCTCCTTTCGCCGCCTGCCGCGCTGC
GTTTCTTGATCTGTGCCCGCACCGAGCCGTGCCCTAGTTTCTTGCCGTGCTGGGGTTGGGTTCGCTCCATTCGGTGCGGC
GAGATGCGGCATGGGTGCGCTGCGAAATTCAAGCGTCCCTTTCCTCTCTCTCTCTCTTTTCTCTGTGTGTGTTTTGTTGC
TGATTACTTTATCTTCGCAACAAAGAGTGTGGGCTTGCTTGGCTGGCATGCCTGCCGAATTCCTTTTGACGGCCGGCCTG
CCGTGTACGCTCGATTGTGGGGAGAGTTAATACGTGAAGAAAGCGACGATGTTTCCGGGAGAGGCTGGCCGAGCCTTTCG
GACAGCCGCGCGTGTCGTTAGTTGATTTCGAGCTGCTAGTAGTCTGATCGCTCGTGTGGGATGGGGATGGTTAACGGATC
CATCTCTGGGTTCTTGCTTTGCTGTGTGTATTTATGTGCCGGCGTCTGATTGTCAGAGGCATGTGTGCAATCTCTCTCCC
CAGCTTACTTCGGCGGAGCAGGAGCAGCAGCAGCAGCCCCGGCAATGGACTGGTCCTTCGCTAGCAAGCCCGGCGCCGCC
CCGGCGTTGATGTCGTTCCGGTCAGCTTCGTTCCCCCAGTTCTCCTCCTTCGACGGCGCCAAGAACCCGGCCCCTCGTAT

CCTCACGCACCAGGTTTGCGCGGCTGCCCTTCTTGGTTCTGGTCCATGTCAGCTTCCGAGTTTTTACTGATACATCTAGG
TCCCTTTTCGGGCCTCTGATGATGCTTTTGCAGAGGTCGTTCGGCCCCGACAGCACGCACTACGCCGCCGCGCACCGCAC
GCAGCATGCGCTGAACGGGGCTAGAGTCACTCCGGTCTCGTCGCCGTTCAACCAGAACAGTCCGATGTTCAGGGTCCAGA
GCTCGCCTAGCCTTCCGAACGGTACTGCGTTCAAGCAGCCGCCTTTTGCCATCAACAACAACGCTGCGGCCAGTTCTACT
GTTGGTTTCTATGGAACAAGGTACTCCTGGTTTATCTGCTGTTACCACAGTGACTTATAAAAGTGGCTGTGGAATTAGTA
TCGACTGAAACATTGCTTGTTTTGCCTGTGAAGGGATGTGGTGAGGCCAAAGACAGCACAATTGACGATCTTCTATGCTG
GTTCTGTCAACGTATTCGACAACGTCTCAGCTGAGAAGGTATAATTTCTCATGGCCTTTCCATTTTGATGCTAATTTGCT
TTGCATTTAAGCTCCTTCATTGACATTTTCCGTTCCACAGGCTCAGGAGCTCATGCTCTTAGCCAGCAGAGGGTCTCTTC
CAAGCTCAGCCCCCGTGGCCCGTAAGCCAGAAGCTCCTATTCTCGCTCCAGCAAAAGTCACAGCGCCTGAGGTTTTGCAT
GCAACACAGATGCTGTTTCAAAAACCACAACACGTTTCTCCACCTTCGTCTGCCATCTCCAAACCAATCCCTGGCATCTT
GCAAGCTGCAAGTCTCCCCAGGAGCGCCTCGAGCTCTAACCTTGACTCCCCATTTCCAAAATCTTCAGTGCCATTTCCTG
TTTCTCCTGTAAGTCAGGCTCCGAGAGCTCAGCCTGCAACCATTGCCGCTACAACTGCAGCAGCAATAATGCCTAGAGGT
ACCGTTGGCTGTTTATGGTTTGCATAGCACCAACTGGCTATCATTGGAGTATTCAGTAATTCTGGTTGCTCATTTGTTGT
ATTGAAATTGTCATTGTCTGTTGCAGCTGTCCCTCAAGCTAGAAAGGCGTCCCTTGCTCGATTCTTGGAGAAAAGAAAGG
AAAGGTAGAATCCATATTCTTGCTCCCAATGTCCTAAAAACAGGGCATTGGTTTCCGAGTGCATCGCAGCACCTTAATTC
CTTACGACATATTTGCATTTGCGTTCCAAAGCTGGTTCTGATCTGTGTATACTTCTCTTGCAGGGTGACAACTGCTGCAC
CTTACCCATCAGCCAAGAGCCCGATGGAGAGCAGTGACACGTTTGGCAGTGGGAGCGCCAACGACAAGTCATCATGCACA
GACATTGCCCTCTCAAGCAACCATGAGGAGTCCCTATGTTTAGGGCAGCCCAGGAACATCAGCTTCATCCAAGAGTCCCC
CAGTACAAAACTACAGATCTGATCTGAATATCTGATCATGCCCAATGTTCATAGATTTGTCCAGGCTGACTGATGCAAGG
ACCATACGATACTTTTGCATTCAGGCAAGGACTCGAGAGGAAGAGATTACGTATGTTATCGGTGCCGCTAGCTGTTGTTT
ATGATGATGTTTTGTTGTGCTAGTTTACTGTGATGCTGGTGGTGCCCCCACCTAACTAACAAGAGAGCTGTCACTTGCAG
ACTCTTGATTTTTTTTGTCCTAGCGCCATTTCTTTAGCGGTATCAAGTCAATTTATATAGGCTTACAAATTCTGCGCCT
TTTGTATGGCCAAAGTGTAATATTTTTGTTCTTGTATTATGAACCTCCATGCTGCAGAATGCAAGAGCAAATCTTATTGT
CGATGGTGTTCTCATTGCCAGCATGTGTACGCAGTAGCGGTTCATGTTGTTTTGAGCTGTTCTGTTTTAATAATATCTCA
AAAGGTTGCCGATTGGTGCCCGTAAGGATGACATTTCAAGAACAGATG
>GRMZM2G126507_T01 cDNA
Chr7: 108871320..108874327
AAGCCATTAAACAAACACAAATAGCACACACGGGCACACGGCAGGCCTACAACTACAAGAGGAGAGGAGAAGAGAGAGGA
GAATTTTCAATGCTGCCGCGCACCGTCATCACTAGCTAGTGGAGGGGCGGACGAGAAGGCGAGGAGCTGAGGGGAGAGAG
GCTTCACAGAGCTTGCTTGAGCCTGGAGGGGGCTGCCGCTGTTTGTCTCTCCACTCCACCCACCAGCAGTTACCTCGGGA
GGGCATGGCAAAATCTGGTGCCTCCTTTCCGGAGTCCAGTTGGATGGAGAGGGATTTCCTGGCCGCGATCGGCAAGGAGC
AGCAGCACCCGCACAAGGAGGAGGCAGGCGCGGAGGAATCTGCTTACTTCGGCGGAGCAGGAGCAGCAGCAGCAGCCCCG
GCAATGGACTGGTCCTTCGCTAGCAAGCCCGGCGCCGCCCCGGCGTTGATGTCGTTCCGGTCAGCTTCGTTCCCCCAGTT
CTCCTCCTTCGACGGCGCCAAGAACCCGGCCCCTCGTATCCTCACGCACCAGAGGTCGTTCGGCCCCGACAGCACGCACT
ACGCCGCCGCGCACCGCACGCAGCATGCGCTGAACGGGGCTAGAGTCACTCCGGTCTCGTCGCCGTTCAACCAGAACAGT
CCGATGTTCAGGGTCCAGAGCTCGCCTAGCCTTCCGAACGGTACTGCGTTCAAGCAGCCGCCTTTTGCCATCAACAACAA
CGCTGCGGCCAGTTCTACTGTTGGTTTCTATGGAACAAGGGATGTGGTGAGGCCAAAGACAGCACAATTGACGATCTTCT
ATGCTGGTTCTGTCAACGTATTCGACAACGTCTCAGCTGAGAAGGCTCAGGAGCTCATGCTCTTAGCCAGCAGAGGGTCT
CTTCCAAGCTCAGCCCCCGTGGCCCGTAAGCCAGAAGCTCCTATTCTCGCTCCAGCAAAAGTCACAGCGCCTGAGGTTTT
GCATGCAACACAGATGCTGTTTCAAAAACCACAACACGTTTCTCCACCTTCGTCTGCCATCTCCAAACCAATCCCTGGCA
TCTTGCAAGCTGCAAGTCTCCCCAGGAGCGCCTCGAGCTCTAACCTTGACTCCCCATTTCCAAAATCTTCAGTGCCATTT
CCTGTTTCTCCTGTAAGTCAGGCTCCGAGAGCTCAGCCTGCAACCATTGCCGCTACAACTGCAGCAGCAATAATGCCTAG
AGCTGTCCCTCAAGCTAGAAAGGCGTCCCTTGCTCGATTCTTGGAGAAAAGAAAGGAAAGGGTGACAACTGCTGCACCTT
ACCCATCAGCCAAGAGCCCGATGGAGAGCAGTGACACGTTTGGCAGTGGGAGCGCCAACGACAAGTCATCATGCACAGAC

ATTGCCCTCTCAAGCAACCATGAGGAGTCCCTATGTTTAGGGCAGCCCAGGAACATCAGCTTCATCCAAGAGTCCCCCAG
TACAAAACTACAGATCTGATCTGAATATCTGATCATGCCCAATGTTCATAGATTTGTCCAGGCTGACTGATGCAAGGACC
ATACGATACTTTTGCATTCAGGCAAGGACTCGAGAGGAAGAGATTACGTATGTTATCGGTGCCGCTAGCTGTTGTTTATG
ATGATGTTTTGTTGTGCTAGTTTACTGTGATGCTGGTGGTGCCCCCACCTAACTAACAAGAGAGCTGTCACTTGCAGACT
CTTGATTTTTTTTTGTCCTAGCGCCATTTCTTTAGCGGTATCAAGTCAATTTATATAGGCTTACAAATTCTGCGCCTTTT
GTATGGCCAAAGTGTAATATTTTTGTTCTTGTATTATGAACCTCCATGCTGCAGAATGCAAGAGCAAATCTTATTGTCGA
TGGTGTTCTCATTGCCAGCATGTGTACGCAGTAGCGGTTCATGTTGTTTTGAGCTGTTCTGTTTTAATAATATCTCAAAA
GGTTGCCGATTGGTGCCCGTAAGGATGACATTTCAAGAACAGATG

>GRMZM2G126507_P01 peptide

MAKSGASFPESSWMERDFLAAIGKEQQHPHKEEAGAEESAYFGGAGAAAAAPAMDWSFASKPGAAPALMSFRSASFPQFS
SFDGAKNPAPRILTHQRSFGPDSTHYAAAHRTQHALNGARVTPVSSPFNQNSPMFRVQSSPSLPNGTAFKQPPFAINNNA
AASSTVGFYGTRDVVRPKTAQLTIFYAGSVNVFDNVSAEKAQELMLLASRGSLPSSAPVARKPEAPILAPAKVTAPEVLH
ATQMLFQKPQHVSPPSSAISKPIPGILQAASLPRSASSSNLDSPFPKSSVPFPVSPVSQAPRAQPATIAATTAAAIMPRA
VPQARKASLARFLEKRKERVTTAAPYPSAKSPMESSDTFGSGSANDKSSCTDIALSSNHEESLCLGQPRNISFIQESPST
KLQI

>GRMZM2G126507_T02 cDNA

Chr7: 108871320..108873623

CTGGCCGAGCCTTTCGGACAGCCGCGCGTGTCGTTAGTTGATTTCGAGCTGCTAGTAGTCTGATCGCTCGTGTGGGATGG
GGATGGTTAACGGATCCATCTCTGGGTTCTTGCTTTGCTGTGTGTATTTATGTGCCGGCGTCTGATTGTCAGAGGCATGT
GTGCAATCTCTCTCCCCAGCTTACTTCGGCGGAGCAGGAGCAGCAGCAGCAGCCCCGGCAATGGACTGGTCCTTCGCTAG
CAAGCCCGGCGCCGCCCCGGCGTTGATGTCGTTCCGGTCAGCTTCGTTCCCCCAGTTCTCCTCCTTCGACGGCGCCAAGA
ACCCGGCCCCTCGTATCCTCACGCACCAGAGGTCGTTCGGCCCCGACAGCACGCACTACGCCGCCGCGCACCGCACGCAG
CATGCGCTGAACGGGGCTAGAGTCACTCCGGTCTCGTCGCCGTTCAACCAGAACAGTCCGATGTTCAGGGTCCAGAGCTC
GCCTAGCCTTCCGAACGGTACTGCGTTCAAGCAGCCGCCTTTTGCCATCAACAACAACGCTGCGGCCAGTTCTACTGTTG
GTTTCTATGGAACAAGGGATGTGGTGAGGCCAAAGACAGCACAATTGACGATCTTCTATGCTGGTTCTGTCAACGTATTC
GACAACGTCTCAGCTGAGAAGGCTCAGGAGCTCATGCTCTTAGCCAGCAGAGGGTCTCTTCCAAGCTCAGCCCCCGTGGC
CCGTAAGCCAGAAGCTCCTATTCTCGCTCCAGCAAAAGTCACAGCGCCTGAGGTTTTGCATGCAACACAGATGCTGTTTC
AAAAACCACAACACGTTTCTCCACCTTCGTCTGCCATCTCCAAACCAATCCCTGGCATCTTGCAAGCTGCAAGTCTCCCC
AGGAGCGCCTCGAGCTCTAACCTTGACTCCCCATTTCCAAAATCTTCAGTGCCATTTCCTGTTTCTCCTGTAAGTCAGGC
TCCGAGAGCTCAGCCTGCAACCATTGCCGCTACAACTGCAGCAGCAATAATGCCTAGAGCTGTCCCTCAAGCTAGAAAGG
CGTCCCTTGCTCGATTCTTGGAGAAAAGAAAGGAAAGGGTGACAACTGCTGCACCTTACCCATCAGCCAAGAGCCCGATG
GAGAGCAGTGACACGTTTGGCAGTGGGAGCGCCAACGACAAGTCATCATGCACAGACATTGCCCTCTCAAGCAACCATGA
GGAGTCCCTATGTTTAGGGCAGCCCAGGAACATCAGCTTCATCCAAGAGTCCCCCAGTACAAAACTACAGATCTGATCTG
AATATCTGATCATGCCCAATGTTCATAGATTTGTCCAGGCTGACTGATGCAAGGACCATACGATACTTTTGCATTCAGGC
AAGGACTCGAGAGGAAGAGATTACGTATGTTATCGGTGCCGCTAGCTGTTGTTTATGATGATGTTTTGTTGTGCTAGTTT
ACTGTGATGCTGGTGGTGCCCCCACCTAACTAACAAGAGAGCTGTCACTTGCAGACTCTTGATTTTTTTTTGTCCTAGCG
CCATTTCTTTAGCGGTATCAAGTCAATTTATATAGGCTTACAAATTCTGCGCCTTTTGTATGGCCAAAGTGTAATATTTT
TGTTCTTGTATTATGAACCTCCATGCTGCAGAATGCAAGAGCAAATCTTATTGTCGATGGTGTTCTCATTGCCAGCATGT
GTACGCAGTAGCGGTTCATGTTGTTTTGAGCTGTTCTGTTTTAATAATATCTCAAAAGGTTGCCGATTGGTGCCCGTAAG
GATGACATTTCAAGAACAGATG

>GRMZM2G126507_P02 peptide

MDWSFASKPGAAPALMSFRSASFPQFSSFDGAKNPAPRILTHQRSFGPDSTHYAAAHRTQHALNGARVTPVSSPFNQNSP
MFRVQSSPSLPNGTAFKQPPFAINNNAAASSTVGFYGTRDVVRPKTAQLTIFYAGSVNVFDNVSAEKAQELMLLASRGSL
PSSAPVARKPEAPILAPAKVTAPEVLHATQMLFQKPQHVSPPSSAISKPIPGILQAASLPRSASSSNLDSPFPKSSVPFP

VSPVSQAPRAQPATIAATTAAAIMPRAVPQARKASLARFLEKRKERVTTAAPYPSAKSPMESSDTFGSGSANDKSSCTDI
ALSSNHEESLCLGQPRNISFIQESPSTKLQI

AGPv4:
Zm00001d020409 (zim1 - ZIM-transcription factor 1)
Chr7: 112014245..112017209

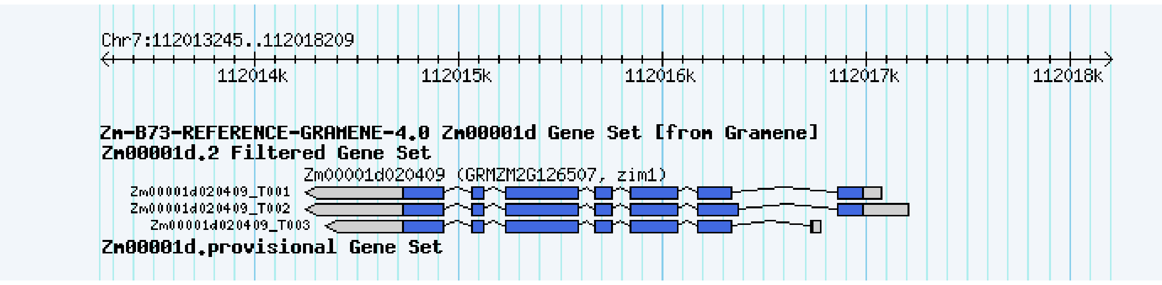

>Zm00001d020409 Genomic DNA
ACAAATAGCACACACGGGCACACGGCAGGCCTACAACTACAAGAGGAGAGGAGAAGAGAGAGGAGAATTTTCAATGCTGC
CGCGCACCGTCATCACTAGCTAGTGGAGGGGCGGACGAGAAGGCGAGGAGCTGAGGGGAGAGAGGCTTCACAGAGCTTGC
TTGAGCCTGGAGGGGGCTGCCGCTGTTTGTCTCTCCACTCCACCCACCAGCAGTTACCTCGGGAGGGCATGGCAAAATCT
GGTGCCTCCTTTCCGGAGTCCAGTTGGATGGAGAGGGATTTCCTGGCCGCGATCGGCAAGGAGCAGCAGCACCCGCACAA
GGAGGAGGCAGGCGCGGAGGAATCTGGTATGCTCCCGGCCTCCTTTCGCCGCCTGCCGCGCTGCGTTTCTTGATCTGTGC
CCGCACCGAGCCGTGCCCTAGTTTCTTGCCGTGCTGGGGTTGGGTTCGCTCCATTCGGTGCGGCGAGATGCGGCATGGGT
GCGCTGCGAAATTCAAGCGTCCCTTTCCTCTCTCTCTCTTTTCTCTGTGTGTGTTTTGTTGCTGATTACTTTATCTTC
GCAACAAAGAGTGTGGGCTTGCTTGGCTGGCATGCCTGCCGAATTCCTTTTGACGGCCGGCCTGCCGTGTACGCTCGATT
GTGGGGAGAGTTAATACGTGAAGAAAGCGACGATGTTTCCGGGAGAGGCTGGCCGAGCCTTTCGGACAGCCGCGCGTGTC
GTTAGTTGATTTCGAGCTGCTAGTAGTCTGATCGCTCGTGTGGGATGGGGATGGTTAACGGATCCATCTCTGGGTTCTTG
CTTTGCTGTGTGTATTTATGTGCCGGCGTCTGATTGTCAGAGGCATGTGTGCAATCTCTCTCCCCAGCTTACTTCGGCGG
AGCAGGAGCAGCAGCAGCAGCCCCGGCAATGGACTGGTCCTTCGCTAGCAAGCCCGGCGCCGCCCCGGCGTTGATGTCGT
TCCGGTCAGCTTCGTTCCCCCAGTTCTCCTCCTTCGACGGCGCCAAGAACCCGGCCCCTCGTATCCTCACGCACCAGGTT
TGCGCGGCTGCCCTTCTTGGTTCTGGTCCATGTCAGCTTCCGAGTTTTTACTGATACATCTAGGTCCCTTTTCGGGCCTC
TGATGATGCTTTTGCAGAGGTCGTTCGGCCCCGACAGCACGCACTACGCCGCCGCGCACCGCACGCAGCATGCGCTGAAC
GGGGCTAGAGTCACTCCGGTCTCGTCGCCGTTCAACCAGAACAGTCCGATGTTCAGGGTCCAGAGCTCGCCTAGCCTTCC
GAACGGTACTGCGTTCAAGCAGCCGCCTTTTGCCATCAACAACAACGCTGCGGCCAGTTCTACTGTTGGTTTCTATGGAA
CAAGGTACTCCTGGTTTATCTGCTGTTACCACAGTGACTTATAAAAGTGGCTGTGGAATTAGTATCGACTGAAACATTGC
TTGTTTTGCCTGTGAAGGGATGTGGTGAGGCCAAAGACAGCACAATTGACGATCTTCTATGCTGGTTCTGTCAACGTATT
CGACAACGTCTCAGCTGAGAAGGTATAATTTCTCATGGCCTTTCCATTTTGATGCTAATTTGCTTTGCATTTAAGCTCCT
TCATTGACATTTTCCGTTCCACAGGCTCAGGAGCTCATGCTCTTAGCCAGCAGAGGGTCTCTTCCAAGCTCAGCCCCCGT
GGCCCGTAAGCCAGAAGCTCCTATTCTCGCTCCAGCAAAAGTCACAGCGCCTGAGGTTTTGCATGCAACACAGATGCTGT
TTCAAAAACCACAACACGTTTCTCCACCTTCGTCTGCCATCTCCAAACCAATCCCTGGCATCTTGCAAGCTGCAAGTCTC
CCCAGGAGCGCCTCGAGCTCTAACCTTGACTCCCCATTTCCAAAATCTTCAGTGCCATTTCCTGTTTCTCCTGTAAGTCA
GGCTCCGAGAGCTCAGCCTGCAACCATTGCCGCTACAACTGCAGCAGCAATAATGCCTAGAGGTACCGTTGGCTGTTTAT
GGTTTGCATAGCACCAACTGGCTATCATTGGAGTATTCAGTAATTCTGGTTGCTCATTTGTTGTATTGAAATTGTCATTG
TCTGTTGCAGCTGTCCCTCAAGCTAGAAAGGCGTCCCTTGCTCGATTCTTGGAGAAAAGAAAGGAAAGGTAGAATCCATA
TTCTTGCTCCCAATGTCCTAAAAACAGGGCATTGGTTTCCGAGTGCATCGCAGCACCTTAATTCCTTACGACATATTTGC
ATTTGCGTTCCAAAGCTGGTTCTGATCTGTGTATACTTCTCTTGCAGGGTGACAACTGCTGCACCTTACCCATCAGCCAA
GAGCCCGATGGAGAGCAGTGACACGTTTGGCAGTGGGAGCGCCAACGACAAGTCATCATGCACAGACATTGCCCTCTCAA

GCAACCATGAGGAGTCCCTATGTTTAGGGCAGCCCAGGAACATCAGCTTCATCCAAGAGTCCCCCAGTACAAAACTACAG
ATCTGATCTGAATATCTGATCATGCCCAATGTTCATAGATTTGTCCAGGCTGACTGATGCAAGGACCATACGATACTTTT
GCATTCAGGCAAGGACTCGAGAGGAAGAGATTACGTATGTTATCGGTGCCGCTAGCTGTTGTTTATGATGATGTTTTGTT
GTGCTAGTTTACTGTGATGCTGGTGGTGCCCCCACCTAACTAACAAGAGAGCTGTCACTTGCAGACTCTTGATTTTTTTT
TGTCCTAGCGCCATTTCTTTAGCGGTATCAAGTCAATTTATATAGGCTTACAAATTCTGCGCCTTTTGTATGGCCAAAGT
GTAATATTTTTGTTCTTGTATTATGAACCTCCATGCTGCAGAATGCAAGAGCAAATCTTATTGTCGATGGTGTTCTCATT
GCCAGCATGTGTACGCAGTAGCGGTTCATGTTGTTTTGAGCTGTTCTGTTTTAATAATATCTCAAAAGGTTGCCGATTGG
TGCCC
>Zm00001d020409_T001 cDNA
Chr7: 112014245..112017077
GAGGGGAGAGAGGCTTCACAGAGCTTGCTTGAGCCTGGAGGGGGCTGCCGCTGTTTGTCTCTCCACTCCACCCACCAGCA
GTTACCTCGGGAGGGCATGGCAAAATCTGGTGCCTCCTTTCCGGAGTCCAGTTGGATGGAGAGGGATTTCCTGGCCGCGA
TCGGCAAGGAGCAGCAGCACCCGCACAAGGAGGAGGCAGGCGCGGAGGAATCTGCTTACTTCGGCGGAGCAGGAGCAGCA
GCAGCAGCCCCGGCAATGGACTGGTCCTTCGCTAGCAAGCCCGGCGCCGCCCCGGCGTTGATGTCGTTCCGGTCAGCTTC
GTTCCCCCAGTTCTCCTCCTTCGACGGCGCCAAGAACCCGGCCCCTCGTATCCTCACGCACCAGAGGTCGTTCGGCCCCG
ACAGCACGCACTACGCCGCCGCGCACCGCACGCAGCATGCGCTGAACGGGGCTAGAGTCACTCCGGTCTCGTCGCCGTTC
AACCAGAACAGTCCGATGTTCAGGGTCCAGAGCTCGCCTAGCCTTCCGAACGGTACTGCGTTCAAGCAGCCGCCTTTTGC
CATCAACAACAACGCTGCGGCCAGTTCTACTGTTGGTTTCTATGGAACAAGGGATGTGGTGAGGCCAAAGACAGCACAAT
TGACGATCTTCTATGCTGGTTCTGTCAACGTATTCGACAACGTCTCAGCTGAGAAGGCTCAGGAGCTCATGCTCTTAGCC
AGCAGAGGGTCTCTTCCAAGCTCAGCCCCCGTGGCCCGTAAGCCAGAAGCTCCTATTCTCGCTCCAGCAAAAGTCACAGC
GCCTGAGGTTTTGCATGCAACACAGATGCTGTTTCAAAAACCACAACACGTTTCTCCACCTTCGTCTGCCATCTCCAAAC
CAATCCCTGGCATCTTGCAAGCTGCAAGTCTCCCCAGGAGCGCCTCGAGCTCTAACCTTGACTCCCCATTTCCAAAATCT
TCAGTGCCATTTCCTGTTTCTCCTGTAAGTCAGGCTCCGAGAGCTCAGCCTGCAACCATTGCCGCTACAACTGCAGCAGC
AATAATGCCTAGAGCTGTCCCTCAAGCTAGAAAGGCGTCCCTTGCTCGATTCTTGGAGAAAAGAAAGGAAAGGGTGACAA
CTGCTGCACCTTACCCATCAGCCAAGAGCCCGATGGAGAGCAGTGACACGTTTGGCAGTGGGAGCGCCAACGACAAGTCA
TCATGCACAGACATTGCCCTCTCAAGCAACCATGAGGAGTCCCTATGTTTAGGGCAGCCCAGGAACATCAGCTTCATCCA
AGAGTCCCCCAGTACAAAACTACAGATCTGATCTGAATATCTGATCATGCCCAATGTTCATAGATTTGTCCAGGCTGACT
GATGCAAGGACCATACGATACTTTTGCATTCAGGCAAGGACTCGAGAGGAAGAGATTACGTATGTTATCGGTGCCGCTAG
CTGTTGTTTATGATGATGTTTTGTTGTGCTAGTTTACTGTGATGCTGGTGGTGCCCCCACCTAACTAACAAGAGAGCTGT
CACTTGCAGACTCTTGATTTTTTTTTGTCCTAGCGCCATTTCTTTAGCGGTATCAAGTCAATTTATATAGGCTTACAAAT
TCTGCGCCTTTTGTATGGCCAAAGTGTAATATTTTTGTTCTTGTATTATGAACCTCCATGCTGCAGAATGCAAGAGCAAA
TCTTATTGTCGATGGTGTTCTCATTGCCAGCATGTGTACGCAGTAGCGGTTCATGTTGTTTTGAGCTGTTCTGTTTTAAT
AATATCTCAAAAGGTTGCCGATTGGTGCCC
>Zm00001d020409_P001 peptide
MAKSGASFPESSWMERDFLAAIGKEQQHPHKEEAGAEESAYFGGAGAAAAAPAMDWSFASKPGAAPALMSFRSASFPQFS
SFDGAKNPAPRILTHQRSFGPDSTHYAAAHRTQHALNGARVTPVSSPFNQNSPMFRVQSSPSLPNGTAFKQPPFAINNNA
AASSTVGFYGTRDVVRPKTAQLTIFYAGSVNVFDNVSAEKAQELMLLASRGSLPSSAPVARKPEAPILAPAKVTAPEVLH
ATQMLFQKPQHVSPPSSAISKPIPGILQAASLPRSASSSNLDSPFPKSSVPFPVSPVSQAPRAQPATIAATTAAAIMPRA
VPQARKASLARFLEKRKERVTTAAPYPSAKSPMESSDTFGSGSANDKSSCTDIALSSNHEESLCLGQPRNISFIQESPST
KLQI
>Zm00001d020409_T002 cDNA
Chr7: 112014245..112017209
ACAAATAGCACACACGGGCACACGGCAGGCCTACAACTACAAGAGGAGAGGAGAAGAGAGAGGAGAATTTTCAATGCTGC
CGCGCACCGTCATCACTAGCTAGTGGAGGGGCGGACGAGAAGGCGAGGAGCTGAGGGGAGAGAGGCTTCACAGAGCTTGC

TTGAGCCTGGAGGGGGCTGCCGCTGTTTGTCTCTCCACTCCACCCACCAGCAGTTACCTCGGGAGGGCATGGCAAAATCT
GGTGCCTCCTTTCCGGAGTCCAGTTGGATGGAGAGGGATTTCCTGGCCGCGATCGGCAAGGAGCAGCAGCACCCGCACAA
GGAGGAGGCAGGCGCGGAGGAATCTGAGGCATGTGTGCAATCTCTCTCCCCAGCTTACTTCGGCGGAGCAGGAGCAGCAG
CAGCAGCCCCGGCAATGGACTGGTCCTTCGCTAGCAAGCCCGGCGCCGCCCCGGCGTTGATGTCGTTCCGGTCAGCTTCG
TTCCCCCAGTTCTCCTCCTTCGACGGCGCCAAGAACCCGGCCCCTCGTATCCTCACGCACCAGAGGTCGTTCGGCCCCGA
CAGCACGCACTACGCCGCCGCGCACCGCACGCAGCATGCGCTGAACGGGGCTAGAGTCACTCCGGTCTCGTCGCCGTTCA
ACCAGAACAGTCCGATGTTCAGGGTCCAGAGCTCGCCTAGCCTTCCGAACGGTACTGCGTTCAAGCAGCCGCCTTTTGCC
ATCAACAACAACGCTGCGGCCAGTTCTACTGTTGGTTTCTATGGAACAAGGGATGTGGTGAGGCCAAAGACAGCACAATT
GACGATCTTCTATGCTGGTTCTGTCAACGTATTCGACAACGTCTCAGCTGAGAAGGCTCAGGAGCTCATGCTCTTAGCCA
GCAGAGGGTCTCTTCCAAGCTCAGCCCCCGTGGCCCGTAAGCCAGAAGCTCCTATTCTCGCTCCAGCAAAAGTCACAGCG
CCTGAGGTTTTGCATGCAACACAGATGCTGTTTCAAAAACCACAACACGTTTCTCCACCTTCGTCTGCCATCTCCAAACC
AATCCCTGGCATCTTGCAAGCTGCAAGTCTCCCCAGGAGCGCCTCGAGCTCTAACCTTGACTCCCCATTTCCAAAATCTT
CAGTGCCATTTCCTGTTTCTCCTGTAAGTCAGGCTCCGAGAGCTCAGCCTGCAACCATTGCCGCTACAACTGCAGCAGCA
ATAATGCCTAGAGCTGTCCCTCAAGCTAGAAAGGCGTCCCTTGCTCGATTCTTGGAGAAAAGAAAGGAAAGGGTGACAAC
TGCTGCACCTTACCCATCAGCCAAGAGCCCGATGGAGAGCAGTGACACGTTTGGCAGTGGGAGCGCCAACGACAAGTCAT
CATGCACAGACATTGCCCTCTCAAGCAACCATGAGGAGTCCCTATGTTTAGGGCAGCCCAGGAACATCAGCTTCATCCAA
GAGTCCCCCAGTACAAAACTACAGATCTGATCTGAATATCTGATCATGCCCAATGTTCATAGATTTGTCCAGGCTGACTG
ATGCAAGGACCATACGATACTTTTGCATTCAGGCAAGGACTCGAGAGGAAGAGATTACGTATGTTATCGGTGCCGCTAGC
TGTTGTTTATGATGATGTTTTGTTGTGCTAGTTTACTGTGATGCTGGTGGTGCCCCCACCTAACTAACAAGAGAGCTGTC
ACTTGCAGACTCTTGATTTTTTTTTGTCCTAGCGCCATTTCTTTAGCGGTATCAAGTCAATTTATATAGGCTTACAAATT
CTGCGCCTTTTGTATGGCCAAAGTGTAATATTTTTGTTCTTGTATTATGAACCTCCATGCTGCAGAATGCAAGAGCAAAT
CTTATTGTCGATGGTGTTCTCATTGCCAGCATGTGTACGCAGTAGCGGTTCATGTTGTTTTGAGCTGTTCTGTTTTAATA
ATATCTCAAAAGGTTGCCGATTGGTGCCC
>Zm00001d020409_P002 peptide
MAKSGASFPESSWMERDFLAAIGKEQQHPHKEEAGAEESEACVQSLSPAYFGGAGAAAAAPAMDWSFASKPGAAPALMSF
RSASFPQFSSFDGAKNPAPRILTHQRSFGPDSTHYAAAHRTQHALNGARVTPVSSPFNQNSPMFRVQSSPSLPNGTAFKQ
PPFAINNNAAASSTVGFYGTRDVVRPKTAQLTIFYAGSVNVFDNVSAEKAQELMLLASRGSLPSSAPVARKPEAPILAPA
KVTAPEVLHATQMLFQKPQHVSPPSSAISKPIPGILQAASLPRSASSSNLDSPFPKSSVPFPVSPVSQAPRAQPATIAAT
TAAAIMPRAVPQARKASLARFLEKRKERVTTAAPYPSAKSPMESSDTFGSGSANDKSSCTDIALSSNHEESLCLGQPRNI
SFIQESPSTKLQI
>Zm00001d020409_T003 cDNA
Chr7: 112014347..112016779
GTGCTGGGGTTGGGTTCGCTCCATTCGGTGCGGCGAGATGCGGCATGGCTTACTTCGGCGGAGCAGGAGCAGCAGCAGCA
GCCCCGGCAATGGACTGGTCCTTCGCTAGCAAGCCCGGCGCCGCCCCGGCGTTGATGTCGTTCCGGTCAGCTTCGTTCCC
CCAGTTCTCCTCCTTCGACGGCGCCAAGAACCCGGCCCCTCGTATCCTCACGCACCAGAGGTCGTTCGGCCCCGACAGCA
CGCACTACGCCGCCGCGCACCGCACGCAGCATGCGCTGAACGGGGCTAGAGTCACTCCGGTCTCGTCGCCGTTCAACCAG
AACAGTCCGATGTTCAGGGTCCAGAGCTCGCCTAGCCTTCCGAACGGTACTGCGTTCAAGCAGCCGCCTTTTGCCATCAA
CAACAACGCTGCGGCCAGTTCTACTGTTGGTTTCTATGGAACAAGGGATGTGGTGAGGCCAAAGACAGCACAATTGACGA
TCTTCTATGCTGGTTCTGTCAACGTATTCGACAACGTCTCAGCTGAGAAGGCTCAGGAGCTCATGCTCTTAGCCAGCAGA
GGGTCTCTTCCAAGCTCAGCCCCCGTGGCCCGTAAGCCAGAAGCTCCTATTCTCGCTCCAGCAAAAGTCACAGCGCCTGA
GGTTTTGCATGCAACACAGATGCTGTTTCAAAAACCACAACACGTTTCTCCACCTTCGTCTGCCATCTCCAAACCAATCC
CTGGCATCTTGCAAGCTGCAAGTCTCCCCAGGAGCGCCTCGAGCTCTAACCTTGACTCCCCATTTCCAAAATCTTCAGTG
CCATTTCCTGTTTCTCCTGTAAGTCAGGCTCCGAGAGCTCAGCCTGCAACCATTGCCGCTACAACTGCAGCAGCAATAAT
GCCTAGAGCTGTCCCTCAAGCTAGAAAGGCGTCCCTTGCTCGATTCTTGGAGAAAAGAAAGGAAAGGGTGACAACTGCTG

CACCTTACCCATCAGCCAAGAGCCCGATGGAGAGCAGTGACACGTTTGGCAGTGGGAGCGCCAACGACAAGTCATCATGC
ACAGACATTGCCCTCTCAAGCAACCATGAGGAGTCCCTATGTTTAGGGCAGCCCAGGAACATCAGCTTCATCCAAGAGTC
CCCCAGTACAAAACTACAGATCTGATCTGAATATCTGATCATGCCCAATGTTCATAGATTTGTCCAGGCTGACTGATGCA
AGGACCATACGATACTTTTGCATTCAGGCAAGGACTCGAGAGGAAGAGATTACGTATGTTATCGGTGCCGCTAGCTGTTG
TTTATGATGATGTTTTGTTGTGCTAGTTTACTGTGATGCTGGTGGTGCCCCCACCTAACTAACAAGAGAGCTGTCACTTG
CAGACTCTTGATTTTTTTTTGTCCTAGCGCCATTTCTTTAGCGGTATCAAGTCAATTTATATAGGCTTACAAATTCTGCG
CCTTTTGTATGGCCAAAGTGTAATATTTTTGTTCTTGTATTATGAACCTCCATGCTGCAGAATGCAAGAGCAAATCTTAT
TG
>Zm00001d020409_P003 peptide
MAYFGGAGAAAAAPAMDWSFASKPGAAPALMSFRSASFPQFSSFDGAKNPAPRILTHQRSFGPDSTHYAAAHRTQHALNG
ARVTPVSSPFNQNSPMFRVQSSPSLPNGTAFKQPPFAINNNAAASSTVGFYGTRDVVRPKTAQLTIFYAGSVNVFDNVSA
EKAQELMLLASRGSLPSSAPVARKPEAPILAPAKVTAPEVLHATQMLFQKPQHVSPPSSAISKPIPGILQAASLPRSASS
SNLDSPFPKSSVPFPVSPVSQAPRAQPATIAATTAAAIMPRAVPQARKASLARFLEKRKERVTTAAPYPSAKSPMESSDT
FGSGSANDKSSCTDIALSSNHEESLCLGQPRNISFIQESPSTKLQI

ZmJAZ18 (TIFY25)

B73 RefGen_v3:
GRMZM2G116614 (zim28 - ZIM-transcription factor 28)
Chr7: 121257089..121259224

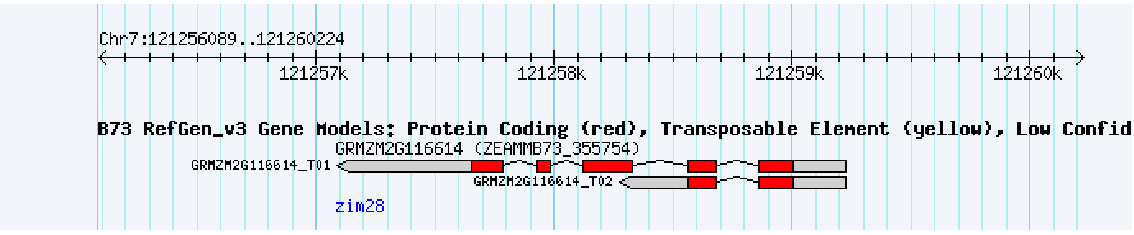

>GRMZM2G116614 Genomic DNA
GCTATTAAAGGGGGGGCAAAGGGCCATTTGCGAGAGGAGGAAAAGCACGCGAGAGCTTGTCGGAGGGCTCTCTGTCTCTG
TGATAAACCGATACGCAGAGGAAGCTGCGGAGAGGTGTCCCTGCTCTGCTGGTGAGCTGCGGCCAGCGGGTGAAGTGAAG
TGGACCGGACGTGGACGAAGGAGAGAGAGAGAGAGAGAGAGAAGAAAGGGCGCCGCGGGGGCCATGGCTGGACACGCGCCGG
CGAGGGACAAGACCACCACCGGCTTCGCCGCTACGTGCAGCCTGCTGAGCCAGTTTCTCAAGGAGAAGAAGGGCGGCCTG
CAGGGCCTCGGCGGCCTCGCCATGGCGCCGGCGCCGGCAGCTGGTAAGGCGACACTTGCTCTTCCGTGCAACCTGCTCGG
CTGCTCCTCTCAGTTCAGCCATCCATGCACGGTTAACTAAGCTCCCTGTGAACACTAACCGCCGTGTGTGCACCAAAGCT
TAATTAGCCAATGAAATGGACACTAGTACCGTTTTTGCTTACCGGACTCGCCCATCGTCGCAGGAGCAGGGGCTTTCCGG
CCGCCGACCACCATGAACTTGCTGTCCGCGCTCGACGCGGCCAAGGCCACCGTCGGGGAGCCAGAAGGCCATGGCCAACG
CACCGGCGGGAATCCAAGGTAAATCACAAGCGAAATTAAACCCACGACCGTCCGTGTGCAATACTCCTGGTAAATTGATG
ACCAGGAAATCAAGAAAAATAAAGCGTGTGTTTGCGCATGTGTTCAATTCTCCCCAACAAGAAAGAACAACACACACTCA
GCTCAAAAGAAAGAACGAACGAACGAACGTGTGATATATATATATACACACATATATATCATTATGGGCAAATAACAGAG
ACAGTGTTCCTGCAGGGAGGCAGCTGGGGAGGAGGCGCAGCAGCTGACCATCTTCTACGGCGGGAAAGTGGTCGTCTTCG
ACAGGTTCCCCTCCGCCAAGGTCAAGGACCTGCTGCAGATCGTGAGCCCGCCGGGGGCCGACGCCGTGGTGGACGGTGCC
GGTGCCGGTGCCGCTGTCCCTACGCAGAACCTGCCTAGGCCTTCACACGACAGTCTCTCTGCCGGTAAAGCCTCAGCTCC
TCCCTCTTTAGTACTTTTCTTACTACGTTGGTGAGAAAGAAAAAAAGAAATGCTGCTGTTGGTTTCGCACGACTTTGTCG
GTGACGCCAAGTGTTATTGATCATTTTTGCTCTGTGACAGATCTGCCGATCGCGAGGCGGAACTCGCTGCACAGGTTCCT
CGAGAAGAGAAAGGACAGGTAACTGAGTCGTTTTTTTTTGCCGAATTCTCAGTGATGCATGGAGGACAGTTGCGTTCAGG

ATCACCGGCTGTGGGCGGAATCAAGTTCAGATAAGCAGAGTTCTGACGAAGTTTTTTTTTTTTGTTCCAAATTCCAGGAT
AACCGCAAAGGCGCCGTACCAAGTCAACAGTTCCGTCGGCGCTGAGGCGTCCAAGGCGGAGAAACCCTGGCTGGGTCTGG
GCCAGGAACAGGAAGGAAGCGACGGTCGTCAAGCAGGAGAGGAGATGTGATCGATCAGTAGAGACTAGATTCGTTCGTGC
GCGCGTCGTCGTAGCTTCAGGTTAAGGGAAAGAATAATCCAACCGTCGGTTAATTGCTCGGCTTAATTACACTGCTCTAC
TACGTAGTACTGGCACTGCTACCACCACCACCACCACCAGAGTGACCAAGTTAAGCTAGGGATTGTTACATAGGCCGGCC
GGTCGGCCGTTCGTTCGCGAGATTGTTTCGCTGCTGGAGATGCGCATGCACGCTCGCAGCAGCTTCGCTGGTCCGCGTGG
CCGCGTCTTGTGTTCGCGCAACGAACGGTGCACGTTCCGGTACGTTTTCATTTGTCTCCGACCTGTCCTGTGTGTGTGCG
TGTGTTTTTGCACTGGATATTCCCCCTCCCCTCTCGTGGTCGTAGGGAGGGTTTGTATGTTCAGTGGAAAGATGTTTGGT
ATGGTCTACTCCATTCTATTTCATGATTCATGAATATGTATGTATGTATGTATGCGTGTGATGTACTACTGTGGTATGCT
GTATAAATTTATAAACACGCGCTTTATTATATTCTGAGCTCCAGAGGTGTTTTGGC
>GRMZM2G116614_T01 cDNA
Chr7: 121257089..121259224
GCTATTAAAGGGGGGGCAAAGGGCCATTTGCGAGAGGAGGAAAAGCACGCGAGAGCTTGTCGGAGGGCTCTCTGTCTCTG
TGATAAACCGATACGCAGAGGAAGCTGCGGAGAGGTGTCCCTGCTCTGCTGGTGAGCTGCGGCCAGCGGGTGAAGTGAAG
TGGACCGGACGTGGACGAAGGAGAGAGAGAGAGAGAGAGAGAAGAAAGGGCGCCGCGGGGGCCATGGCTGGACACGCGCCGG
CGAGGGACAAGACCACCACCGGCTTCGCCGCTACGTGCAGCCTGCTGAGCCAGTTTCTCAAGGAGAAGAAGGGCGGCCTG
CAGGGCCTCGGCGGCCTCGCCATGGCGCCGGCGCCGGCAGCTGGAGCAGGGGCTTTCCGGCCGCCGACCACCATGAACTT
GCTGTCCGCGCTCGACGCGGCCAAGGCCACCGTCGGGGAGCCAGAAGGCCATGGCCAACGCACCGGCGGGAATCCAAGGG
AGGCAGCTGGGGAGGAGGCGCAGCAGCTGACCATCTTCTACGGCGGGAAAGTGGTCGTCTTCGACAGGTTCCCCTCCGCC
AAGGTCAAGGACCTGCTGCAGATCGTGAGCCCGCCGGGGGCCGACGCCGTGGTGGACGGTGCCGGTGCCGGTGCCGCTGT
CCCTACGCAGAACCTGCCTAGGCCTTCACACGACAGTCTCTCTGCCGATCTGCCGATCGCGAGGCGGAACTCGCTGCACA
GGTTCCTCGAGAAGAGAAAGGACAGGATAACCGCAAAGGCGCCGTACCAAGTCAACAGTTCCGTCGGCGCTGAGGCGTCC
AAGGCGGAGAAACCCTGGCTGGGTCTGGGCCAGGAACAGGAAGGAAGCGACGGTCGTCAAGCAGGAGAGGAGATGTGATC
GATCAGTAGAGACTAGATTCGTTCGTGCGCGCGTCGTCGTAGCTTCAGGTTAAGGGAAAGAATAATCCAACCGTCGGTTA
ATTGCTCGGCTTAATTACACTGCTCTACTACGTAGTACTGGCACTGCTACCACCACCACCACCACCAGAGTGACCAAGTT
AAGCTAGGGATTGTTACATAGGCCGGCCGGTCGGCCGTTCGTTCGCGAGATTGTTTCGCTGCTGGAGATGCGCATGCACG
CTCGCAGCAGCTTCGCTGGTCCGCGTGGCCGCGTCTTGTGTTCGCGCAACGAACGGTGCACGTTCCGGTACGTTTTCATT
TGTCTCCGACCTGTCCTGTGTGTGTGCGTGTGTTTTTGCACTGGATATTCCCCCTCCCCTCTCGTGGTCGTAGGGAGGGT
TTGTATGTTCAGTGGAAAGATGTTTGGTATGGTCTACTCCATTCTATTTCATGATTCATGAATATGTATGTATGTATGTA
TGCGTGTGATGTACTACTGTGGTATGCTGTATAAATTTATAAACACGCGCTTTATTATATTCTGAGCTCCAGAGGTGTTT
TGGC
>GRMZM2G116614_P01 peptide
MAGHAPARDKTTTGFAATCSLLSQFLKEKKGGLQGLGGLAMAPAPAAGAGAFRPPTTMNLLSALDAAKATVGEPEGHGQR
TGGNPREAAGEEAQQLTIFYGGKVVVFDRFPSAKVKDLLQIVSPPGADAVVDGAGAGAAVPTQNLPRPSHDSLSADLPIA
RRNSLHRFLEKRKDRITAKAPYQVNSSVGAEASKAEKPWLGLGQEQEGSDGRQAGEEM
>GRMZM2G116614_T02 cDNA
Chr7: 121258276..121259224
GCTATTAAAGGGGGGGCAAAGGGCCATTTGCGAGAGGAGGAAAAGCACGCGAGAGCTTGTCGGAGGGCTCTCTGTCTCTG
TGATAAACCGATACGCAGAGGAAGCTGCGGAGAGGTGTCCCTGCTCTGCTGGTGAGCTGCGGCCAGCGGGTGAAGTGAAG
TGGACCGGACGTGGACGAAGGAGAGAGAGAGAGAGAGAGAGAAGAAAGGGCGCCGCGGGGGCCATGGCTGGACACGCGCCGG
CGAGGGACAAGACCACCACCGGCTTCGCCGCTACGTGCAGCCTGCTGAGCCAGTTTCTCAAGGAGAAGAAGGGCGGCCTG
CAGGGCCTCGGCGGCCTCGCCATGGCGCCGGCGCCGGCAGCTGGAGCAGGGGCTTTCCGGCCGCCGACCACCATGAACTT
GCTGTCCGCGCTCGACGCGGCCAAGGCCACCGTCGGGGAGCCAGAAGGCCATGGCCAACGCACCGGCGGGAATCCAAGGT
AAATCACAAGCGAAATTAAACCCACGACCGTCCGTGTGCAATACTCCTGGTAAATTGATGACCAGGAAATCAAGAAAAAT

AAAGCGTGTGTTTGCGCATGTGTTCAATTCTCCCCAACAAGAAAGAACAACACACACTCAGCTCAAAAGAAAGAACGAAC
GAACGAACGTGTGATATATATATATACACACATATATATCATTATGGGCAAATAACAGAGACAGTGTTCCTGCAGGGAGG
CAGCTGGGGAGGAGGCGCAGCAGCTGACCATCTTCTACGGCGGGAAAGT
>GRMZM2G116614_P02 peptide
MAGHAPARDKTTTGFAATCSLLSQFLKEKKGGLQGLGGLAMAPAPAAGAGAFRPPTTMNLLSALDAAKATVGEPEGHGQR
TGGNPR

AGPv4:
Zm00001d020614 (zim28 - ZIM-transcription factor 28)
Chr7: 125133740..125135827

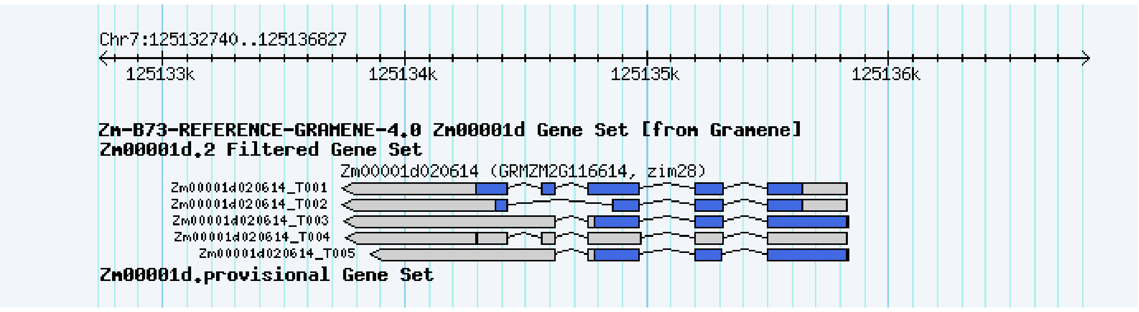

>Zm00001d020614 Genomic DNA
GAGGAGGAAAAGCACGCGAGAGCTTGTCGGAGGGCTCTCTGTCTCTGTGATAAACCGATACGCAGAGGAAGCTGCGGAGA
GGTGTCCCTGCTCTGCTGGTGAGCTGCGGCCAGCGGGTGAAGTGAAGTGGACCGGACGTGGACGAAGGAGAGAGAGAGAG
AGAGAGAAGAAAGGGCGCCGCGGGGGCCATGGCTGGACACGCGCCGGCGAGGGACAAGACCACCACCGGCTTCGCCGCTA
CGTGCAGCCTGCTGAGCCAGTTTCTCAAGGAGAAGAAGGGCGGCCTGCAGGGCCTCGGCGGCCTCGCCATGGCGCCGGCG
CCGGCAGCTGGTAAGGCGACACTTGCTCTTCCGTGCAACCTGCTCGGCTGCTCCTCTCAGTTCAGCCATCCATGCACGGT
TAACTAAGCTCCCTGTGAACACTAACCGCCGTGTGTGCACCAAAGCTTAATTAGCCAATGAAATGGACACTAGTACCGTT
TTTGCTTACCGGACTCGCCCATCGTCGCAGGAGCAGGGGCTTTCCGGCCGCCGACCACCATGAACTTGCTGTCCGCGCTC
GACGCGGCCAAGGCCACCGTCGGGGAGCCAGAAGGCCATGGCCAACGCACCGGCGGGAATCCAAGGTAAATCACAAGCGA
AATTAAACCCACGACCGTCCGTGTGCAATACTCCTGGTAAATTGATGACCAGGAAATCAAGAAAAATAAAGCGTGTGTTT
GCGCATGTGTTCAATTCTCCCCAACAAGAAAGAACAACACACACTCAGCTCAAAAGAAAGAACGAACGAACGAACGTGTG
ATATATATATATACACACATATATATCATTATGGGCAAATAACAGAGACAGTGTTCCTGCAGGGAGGCAGCTGGGGAGGA
GGCGCAGCAGCTGACCATCTTCTACGGCGGGAAAGTGGTCGTCTTCGACAGGTTCCCCTCCGCCAAGGTCAAGGACCTGC
TGCAGATCGTGAGCCCGCCGGGGGCCGACGCCGTGGTGGACGGTGCCGGTGCCGGTGCCGCTGTCCCTACGCAGAACCTG
CCTAGGCCTTCACACGACAGTCTCTCTGCCGGTAAAGCCTCAGCTCCTCCCTCTTTAGTACTTTTCTTACTACGTTGGTG
AGAAAGAAAAAAAGAAATGCTGCTGTTGGTTTCGCACGACTTTGTCGGTGACGCCAAGTGTTATTGATCATTTTTGCTCT
GTGACAGATCTGCCGATCGCGAGGCGGAACTCGCTGCACAGGTTCCTCGAGAAGAGAAAGGACAGGTAACTGAGTCGTTT
TTTTTTGCCGAATTCTCAGTGATGCATGGAGGACAGTTGCGTTCAGGATCACCGGCTGTGGGCGGAATCAAGTTCAGATA
AGCAGAGTTCTGACGAAGTTTTTTTTTTTGTTCCAAATTCCAGGATAACCGCAAAGGCGCCGTACCAAGTCAACAGTTC
CGTCGGCGCTGAGGCGTCCAAGGCGGAGAAACCCTGGCTGGGTCTGGGCCAGGAACAGGAAGGAAGCGACGGTCGTCAAG
CAGGAGAGGAGATGTGATCGATCAGTAGAGACTAGATTCGTTCGTGCGCGCGTCGTCGTAGCTTCAGGTTAAGGGAAAGA
ATAATCCAACCGTCGGTTAATTGCTCGGCTTAATTACACTGCTCTACTACGTAGTACTGGCACTGCTACCACCACCACCA
CCACCAGAGTGACCAAGTTAAGCTAGGGATTGTTACATAGGCCGGCCGGTCGGCCGTTCGTTCGCGAGATTGTTTCGCTG
CTGGAGATGCGCATGCACGCTCGCAGCAGCTTCGCTGGTCCGCGTGGCCGCGTCTTGTGTTCGCGCAACGAACGGTGCAC
GTTCCGGTACGTTTTCATTTGTCTCCGACCTGTCCTGTGTGTGTGCGTGTGTTTTTGCACTGGATATTCCCCCTCCCCTC
TCGTGGTCGTAGGGAGGGTTTGTATGTTCAGTGGAAAGATGTTTGGTATGGTCTACTCCATTCTATTTCATGATTCATGA

ATATGTATGTATGTATGTATGCGTGTGATGTACTACTGTGGTATGCTGTATAAATTTATAAACACGCGCTTTATTATATT
CTGAGCTC
>Zm00001d020614_T001 cDNA
Chr7: 125133740..125135825
GGAGGAAAAGCACGCGAGAGCTTGTCGGAGGGCTCTCTGTCTCTGTGATAAACCGATACGCAGAGGAAGCTGCGGAGAGG
TGTCCCTGCTCTGCTGGTGAGCTGCGGCCAGCGGGTGAAGTGAAGTGGACCGGACGTGGACGAAGGAGAGAGAGAGAGAG
AGAGAAGAAAGGGCGCCGCGGGGGCCATGGCTGGACACGCGCCGGCGAGGGACAAGACCACCACCGGCTTCGCCGCTACG
TGCAGCCTGCTGAGCCAGTTTCTCAAGGAGAAGAAGGGCGGCCTGCAGGGCCTCGGCGGCCTCGCCATGGCGCCGGCGCC
GGCAGCTGGAGCAGGGGCTTTCCGGCCGCCGACCACCATGAACTTGCTGTCCGCGCTCGACGCGGCCAAGGCCACCGTCG
GGGAGCCAGAAGGCCATGGCCAACGCACCGGCGGGAATCCAAGGGAGGCAGCTGGGGAGGAGGCGCAGCAGCTGACCATC
TTCTACGGCGGGAAAGTGGTCGTCTTCGACAGGTTCCCCTCCGCCAAGGTCAAGGACCTGCTGCAGATCGTGAGCCCGCC
GGGGGCCGACGCCGTGGTGGACGGTGCCGGTGCCGGTGCCGCTGTCCCTACGCAGAACCTGCCTAGGCCTTCACACGACA
GTCTCTCTGCCGATCTGCCGATCGCGAGGCGGAACTCGCTGCACAGGTTCCTCGAGAAGAGAAAGGACAGGATAACCGCA
AAGGCGCCGTACCAAGTCAACAGTTCCGTCGGCGCTGAGGCGTCCAAGGCGGAGAAACCCTGGCTGGGTCTGGGCCAGGA
ACAGGAAGGAAGCGACGGTCGTCAAGCAGGAGAGGAGATGTGATCGATCAGTAGAGACTAGATTCGTTCGTGCGCGCGTC
GTCGTAGCTTCAGGTTAAGGGAAAGAATAATCCAACCGTCGGTTAATTGCTCGGCTTAATTACACTGCTCTACTACGTAG
TACTGGCACTGCTACCACCACCACCACCACCAGAGTGACCAAGTTAAGCTAGGGATTGTTACATAGGCCGGCCGGTCGGC
CGTTCGTTCGCGAGATTGTTTCGCTGCTGGAGATGCGCATGCACGCTCGCAGCAGCTTCGCTGGTCCGCGTGGCCGCGTC
TTGTGTTCGCGCAACGAACGGTGCACGTTCCGGTACGTTTTCATTTGTCTCCGACCTGTCCTGTGTGTGTGCGTGTGTTT
TTGCACTGGATATTCCCCCTCCCCTCTCGTGGTCGTAGGGAGGGTTTGTATGTTCAGTGGAAAGATGTTTGGTATGGTCT
ACTCCATTCTATTTCATGATTCATGAATATGTATGTATGTATGTATGCGTGTGATGTACTACTGTGGTATGCTGTATAAA
TTTATAAACACGCGCTTTATTATATTCTGAGCTC
>Zm00001d020614_P001 peptide
MAGHAPARDKTTTGFAATCSLLSQFLKEKKGGLQGLGGLAMAPAPAAGAGAFRPPTTMNLLSALDAAKATVGEPEGHGQR
TGGNPREAAGEEAQQLTIFYGGKVVVFDRFPSAKVKDLLQIVSPPGADAVVDGAGAGAAVPTQNLPRPSHDSLSADLPIA
RRNSLHRFLEKRKDRITAKAPYQVNSSVGAEASKAEKPWLGLGQEQEGSDGRQAGEEM

>Zm00001d020614_T002 cDNA
Chr7: 125133740..125135827
GAGGAGGAAAAGCACGCGAGAGCTTGTCGGAGGGCTCTCTGTCTCTGTGATAAACCGATACGCAGAGGAAGCTGCGGAGA
GGTGTCCCTGCTCTGCTGGTGAGCTGCGGCCAGCGGGTGAAGTGAAGTGGACCGGACGTGGACGAAGGAGAGAGAGAGAG
AGAGAGAAGAAAGGGCGCCGCGGGGGCCATGGCTGGACACGCGCCGGCGAGGGACAAGACCACCACCGGCTTCGCCGCTA
CGTGCAGCCTGCTGAGCCAGTTTCTCAAGGAGAAGAAGGGCGGCCTGCAGGGCCTCGGCGGCCTCGCCATGGCGCCGGCG
CCGGCAGCTGGAGCAGGGGCTTTCCGGCCGCCGACCACCATGAACTTGCTGTCCGCGCTCGACGCGGCCAAGGCCACCGT
CGGGGAGCCAGAAGGCCATGGCCAACGCACCGGCGGGAATCCAAGGGAGGCAGCTGGGGAGGAGGCGCAGCAGCTGACCA
TCTTCTACGGCGGGAAAGTGGTCGTCTTCGACAGGTTCCCCTCCGCCAAGGTCAAGGACCTGCTGCAGATCGATAACCGC
AAAGGCGCCGTACCAAGTCAACAGTTCCGTCGGCGCTGAGGCGTCCAAGGCGGAGAAACCCTGGCTGGGTCTGGGCCAGG
AACAGGAAGGAAGCGACGGTCGTCAAGCAGGAGAGGAGATGTGATCGATCAGTAGAGACTAGATTCGTTCGTGCGCGCGT
CGTCGTAGCTTCAGGTTAAGGGAAAGAATAATCCAACCGTCGGTTAATTGCTCGGCTTAATTACACTGCTCTACTACGTA
GTACTGGCACTGCTACCACCACCACCACCACCAGAGTGACCAAGTTAAGCTAGGGATTGTTACATAGGCCGGCCGGTCGG
CCGTTCGTTCGCGAGATTGTTTCGCTGCTGGAGATGCGCATGCACGCTCGCAGCAGCTTCGCTGGTCCGCGTGGCCGCGT
CTTGTGTTCGCGCAACGAACGGTGCACGTTCCGGTACGTTTTCATTTGTCTCCGACCTGTCCTGTGTGTGTGCGTGTGTT
TTTGCACTGGATATTCCCCCTCCCCTCTCGTGGTCGTAGGGAGGGTTTGTATGTTCAGTGGAAAGATGTTTGGTATGGTC
TACTCCATTCTATTTCATGATTCATGAATATGTATGTATGTATGTATGCGTGTGATGTACTACTGTGGTATGCTGTATAA

ATTTATAAACACGCGCTTTATTATATTCTGAGCTC
>Zm00001d020614_P002 peptide
MAGHAPARDKTTTGFAATCSLLSQFLKEKKGGLQGLGGLAMAPAPAAGAGAFRPPTTMNLLSALDAAKATVGEPEGHGQR
TGGNPREAAGEEAQQLTIFYGGKVVVFDRFPSAKVKDLLQIDNRKGAVPSQQFRRR
>Zm00001d020614_T003 cDNA
Chr7: 125133749..125135827
GAGGAGGAAAAGCACGCGAGAGCTTGTCGGAGGGCTCTCTGTCTCTGTGATAAACCGATACGCAGAGGAAGCTGCGGAGA
GGTGTCCCTGCTCTGCTGGTGAGCTGCGGCCAGCGGGTGAAGTGAAGTGGACCGGACGTGGACGAAGGAGAGAGAGAGAG
AGAGAGAAGAAAGGGCGCCGCGGGGGCCATGGCTGGACACGCGCCGGCGAGGGACAAGACCACCACCGGCTTCGCCGCTA
CGTGCAGCCTGCTGAGCCAGTTTCTCAAGGAGAAGAAGGGCGGCCTGCAGGGCCTCGGCGGCCTCGCCATGGCGCCGGCG
CCGGCAGCTGGAGCAGGGGCTTTCCGGCCGCCGACCACCATGAACTTGCTGTCCGCGCTCGACGCGGCCAAGGCCACCGT
CGGGGAGCCAGAAGGCCATGGCCAACGCACCGGCGGGAATCCAAGGGAGGCAGCTGGGGAGGAGGCGCAGCAGCTGACCA
TCTTCTACGGCGGGAAAGTGGTCGTCTTCGACAGGTTCCCCTCCGCCAAGGTCAAGGACCTGCTGCAGATCGTGAGCCCG
CCGGGGGCCGACGCCGTGGTGGACGGTGCCGGTGCCGGTGCCGCTGTCCCTACGCAGAACCTGCCTAGGCCTTCACACGA
CAGTCTCTCTGCCGATCTGCCGATCGCGAGGCGGAACTCGCTGCACAGGTTCCTCGAGAAGAGAAAGGACAGGTAACTGA
GTCGTTTTTTTTTGCCGAATTCTCAGTGATGCATGGAGGACAGTTGCGTTCAGGATCACCGGCTGTGGGCGGAATCAAGT
TCAGATAAGCAGAGTTCTGACGAAGTTTTTTTTTTTTGTTCCAAATTCCAGGATAACCGCAAAGGCGCCGTACCAAGTCA
ACAGTTCCGTCGGCGCTGAGGCGTCCAAGGCGGAGAAACCCTGGCTGGGTCTGGGCCAGGAACAGGAAGGAAGCGACGGT
CGTCAAGCAGGAGAGGAGATGTGATCGATCAGTAGAGACTAGATTCGTTCGTGCGCGCGTCGTCGTAGCTTCAGGTTAAG
GGAAAGAATAATCCAACCGTCGGTTAATTGCTCGGCTTAATTACACTGCTCTACTACGTAGTACTGGCACTGCTACCACC
ACCACCACCACCAGAGTGACCAAGTTAAGCTAGGGATTGTTACATAGGCCGGCCGGTCGGCCGTTCGTTCGCGAGATTGT
TTCGCTGCTGGAGATGCGCATGCACGCTCGCAGCAGCTTCGCTGGTCCGCGTGGCCGCGTCTTGTGTTCGCGCAACGAAC
GGTGCACGTTCCGGTACGTTTTCATTTGTCTCCGACCTGTCCTGTGTGTGTGCGTGTGTTTTTGCACTGGATATTCCCCC
TCCCCTCTCGTGGTCGTAGGGAGGGTTTGTATGTTCAGTGGAAAGATGTTTGGTATGGTCTACTCCATTCTATTTCATGA
TTCATGAATATGTATGTATGTATGTATGCGTGTGATGTACTACTGTGGTATGCTGTATAAATTTATAAACACGCGCTTTA
TTATAT
>Zm00001d020614_P003 peptide
RRKSTRELVGGLSVSVINRYAEEAAERCPCSAGELRPAGEVKWTGRGRRRERERERKKGRRGGHGWTRAGEGQDHHRLRRY
VQPAEPVSQGEEGRPAGPRRPRHGAGAGSWSRGFPAADHHELAVRARRGQGHRRGARRPWPTHRRESKGGSWGGGAAADH
LLRRESGRLRQVPLRQGQGPAADREPAGGRRRGGRCRCRCRCPYAEPA
>Zm00001d020614_T004 cDNA
Chr7: 125133756..125135825
GGAGGAAAAGCACGCGAGAGCTTGTCGGAGGGCTCTCTGTCTCTGTGATAAACCGATACGCAGAGGAAGCTGCGGAGAGG
TGTCCCTGCTCTGCTGGTGAGCTGCGGCCAGCGGGTGAAGTGAAGTGGACCGGACGTGGACGAAGGAGAGAGAGAGAGAG
AGAGAAGAAAGGGCGCCGCGGGGGCCATGGCTGGACACGCGCCGGCGAGGGACAAGACCACCACCGGCTTCGCCGCTACG
TGCAGCCTGCTGAGCCAGTTTCTCAAGGAGAAGAAGGGCGGCCTGCAGGGCCTCGGCGGCCTCGCCATGGCGCCGGCGCC
GGCAGCTGGAGCAGGGGCTTTCCGGCCGCCGACCACCATGAACTTGCTGTCCGCGCTCGACGCGGCCAAGGCCACCGTCG
GGGAGCCAGAAGGCCATGGCCAACGCACCGGCGGGAATCCAAGTGTTCCTGCAGGGAGGCAGCTGGGGAGGAGGCGCAGC
AGCTGACCATCTTCTACGGCGGGAAAGTGGTCGTCTTCGACAGGTTCCCCTCCGCCAAGGTCAAGGACCTGCTGCAGATC
GTGAGCCCGCCGGGGGCCGACGCCGTGGTGGACGGTGCCGGTGCCGGTGCCGCTGTCCCTACGCAGAACCTGCCTAGGCC
TTCACACGACAGTCTCTCTGCCGATCTGCCGATCGCGAGGCGGAACTCGCTGCACAGGTTCCTCGAGAAGAGAAAGGACA
GGATAACCGCAAAGGCGCCGTACCAAGTCAACAGTTCCGTCGGCGCTGAGGCGTCCAAGGCGGAGAAACCCTGGCTGGGT
CTGGGCCAGGAACAGGAAGGAAGCGACGGTCGTCAAGCAGGAGAGGAGATGTGATCGATCAGTAGAGACTAGATTCGTTC
GTGCGCGCGTCGTCGTAGCTTCAGGTTAAGGGAAAGAATAATCCAACCGTCGGTTAATTGCTCGGCTTAATTACACTGCT

CTACTACGTAGTACTGGCACTGCTACCACCACCACCACCACCAGAGTGACCAAGTTAAGCTAGGGATTGTTACATAGGCC
GGCCGGTCGGCCGTTCGTTCGCGAGATTGTTTCGCTGCTGGAGATGCGCATGCACGCTCGCAGCAGCTTCGCTGGTCCGC
GTGGCCGCGTCTTGTGTTCGCGCAACGAACGGTGCACGTTCCGGTACGTTTTCATTTGTCTCCGACCTGTCCTGTGTGTG
TGCGTGTGTTTTTGCACTGGATATTCCCCCTCCCCTCTCGTGGTCGTAGGGAGGGTTTGTATGTTCAGTGGAAAGATGTT
TGGTATGGTCTACTCCATTCTATTTCATGATTCATGAATATGTATGTATGTATGTATGCGTGTGATGTACTACTGTGGTA
TGCTGTATAAATTTATAAACACGCGCTTT
>Zm00001d020614_P004
M
>Zm00001d020614_T005 cDNA
Chr7: 125133855..125135825
GGAGGAAAAGCACGCGAGAGCTTGTCGGAGGGCTCTCTGTCTCTGTGATAAACCGATACGCAGAGGAAGCTGCGGAGAGG
TGTCCCTGCTCTGCTGGTGAGCTGCGGCCAGCGGGTGAAGTGAAGTGGACCGGACGTGGACGAAGGAGAGAGAGAGAGAG
AGAGAAGAAAGGGCGCCGCGGGGGCCATGGCTGGACACGCGCCGGCGAGGGACAAGACCACCACCGGCTTCGCCGCTACG
TGCAGCCTGCTGAGCCAGTTTCTCAAGGAGAAGAAGGGCGGCCTGCAGGGCCTCGGCGGCCTCGCCATGGCGCCGGCGCC
GGCAGCTGGGGCTTTCCGGCCGCCGACCACCATGAACTTGCTGTCCGCGCTCGACGCGGCCAAGGCCACCGTCGGGGAGC
CAGAAGGCCATGGCCAACGCACCGGCGGGAATCCAAGGGAGGCAGCTGGGGAGGAGGCGCAGCAGCTGACCATCTTCTAC
GGCGGGAAAGTGGTCGTCTTCGACAGGTTCCCCTCCGCCAAGGTCAAGGACCTGCTGCAGATCGTGAGCCCGCCGGGGGC
CGACGCCGTGGTGGACGGTGCCGGTGCCGGTGCCGCTGTCCCTACGCAGAACCTGCCTAGGCCTTCACACGACAGTCTCT
CTGCCGATCTGCCGATCGCGAGGCGGAACTCGCTGCACAGGTTCCTCGAGAAGAGAAAGGACAGGTAACTGAGTCGTTTT
TTTTTGCCGAATTCTCAGTGATGCATGGAGGACAGTTGCGTTCAGGATCACCGGCTGTGGGCGGAATCAAGTTCAGATAA
GCAGAGTTCTGACGAAGTTTTTTTTTTTTGTTCCAAATTCCAGGATAACCGCAAAGGCGCCGTACCAAGTCAACAGTTCC
GTCGGCGCTGAGGCGTCCAAGGCGGAGAAACCCTGGCTGGGTCTGGGCCAGGAACAGGAAGGAAGCGACGGTCGTCAAGC
AGGAGAGGAGATGTGATCGATCAGTAGAGACTAGATTCGTTCGTGCGCGCGTCGTCGTAGCTTCAGGTTAAGGGAAAGAA
TAATCCAACCGTCGGTTAATTGCTCGGCTTAATTACACTGCTCTACTACGTAGTACTGGCACTGCTACCACCACCACCAC
CACCAGAGTGACCAAGTTAAGCTAGGGATTGTTACATAGGCCGGCCGGTCGGCCGTTCGTTCGCGAGATTGTTTCGCTGC
TGGAGATGCGCATGCACGCTCGCAGCAGCTTCGCTGGTCCGCGTGGCCGCGTCTTGTGTTCGCGCAACGAACGGTGCACG
TTCCGGTACGTTTTCATTTGTCTCCGACCTGTCCTGTGTGTGTGCGTGTGTTTTTGCACTGGATATTCCCCCTCCCCTCT
CGTGGTCGTAGGGAGGGTTTGTATGTTCAGTGGAAAGATGTTTGGTATGGTC
>Zm00001d020614_P005 peptide
RKSTRELVGGLSVSVINRYAEEAAERCPCSAGELRPAGEVKWTGRGRRREREREKKGRRGGHGWTRAGEGQDHHRLRRYV
QPAEPVSQGEEGRPAGPRRPRHGAGAGSWGFPAADHHELAVRARRGQGHRRGARRPWPTHRRESKGGSWGGGAAADHLLR
RESGRLRQVPLRQGQGPAADREPAGGRRRGGRCRCRCRCPYAEPA

ZmJAZ19

B73 RefGen_v3:
GRMZM2G066020 (zim31 - ZIM-transcription factor 31)
Chr7: 142622294..142626934

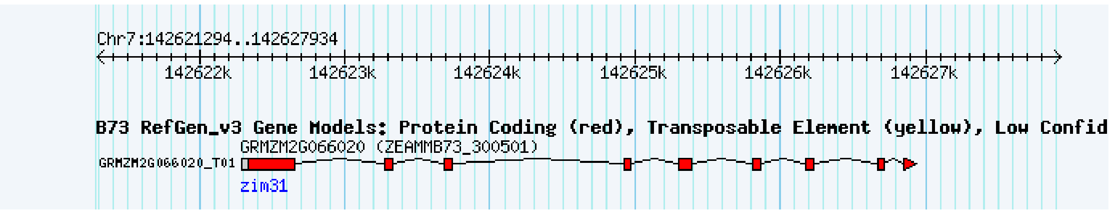

>GRMZM2G066020 Genomic DNA

CCTCCACCTCCGGGCGCCCCGGCCACCACGACCCTGAGCCTGATGCCTGGCGCGGACGTCGGCGCCGACGAGCAGCAGGA
GCCGGCCGCCGCGAGGGCCGAGGAGATGCCCGAGCCTACCCCAGCCCCCGCCGCGGCGCCGCCGCTCACCATCTTCTACG
GTGGCACGGTGGTGGTGTTCGAGGACTTCCCGGCGGAGAAGACGGCCGAGGTCATGCGCCCCGCGGCCGGCGACGACCTG
CCCATCGCCCGGAAGGCGTCGCTGCCGTGGTTCCTGGCCAAGCGCAAGGACCGCCTCGTCGAGCGCGCGCCCTACGCCCG
CCCGTCGTCCCCCGCGAAGGAGCCGGAGAAGAAGACGGTCAAGCCAGCCTCGGCCTCAGCCTCATGCTCAACAAAATGAC
TGTGTGACTGCTGTTGCCTGAACCGAACTGGCCACCTCTACGCTCATTCCTCTCACGTTTCTGAAGGGTGCAGAGTAGCC
GGTCGACTAATCGAGCTCCCTACGCCTCCTGCGCACGGCCTAAAACTCCCTGCTCTTGCCTGCACGCCTCGCTCTGCTTA
GCATCGGAGCGCACGGACGCTGCAGCCATGTTTATTCCTTGCAGCGAAACGCATGGGCTTGAAGGAGTTCAAAATGGGGT
ATGAATCATCTCAGTTTGACTTTTGTTTTAAATCCATTGATTATTGACCCTTATGTCTAAACTGCCCTAAGATACTTATT
TCTGGCTTCATAAATCACAAAACAGAGCCAAAGCTATAGTCAATGCAGAAAGTAAACCGAGCTAACAAATAAAAAACAAT
GCTACGACACACAAGATCAACAAGCTAGAGCCTAAATTAACATCAGTAATTCAAATCCACTTAAGTAATATAGTGTGTGA
TGTAAGCCAAAAAAACCAGATGCAACCATTCTAGCCAAAGACTCTTTAGCCCATAAGTAATTCATGTTTTTGTAGATGCT
CGACATGGTTCTTGATGATCTATAGAATGAGCACCCTAATCTCTGTTCTCCTCGGTTCCCGCGTGTGTGGAAAAGCAGCG
GTGAGGTTCAATAACTGGTTACACATTGTTTTTTGATTGCTCAGTGCAACCATGATGTTTGATGAAATGTTCAACTCTGC
TATTGCACATTTGCACGCTGCAGTGCAGTGTTGAAATGTAGTTTACTTTTTGTTTGAGAATTCACATGACAGGTCAGTTT
GTACATTTTTAAGAGTCCAAATTAGGTTCGTTCTGCATAATACATGTGGTGTTTAATACCATTGTATTCCGAAAATGATA
TGAGCTTACCTTGCTGTATCTCCATGTATATCATGAAAATTTACTTAAAATTGGACGCTTGATTGATTGAAAGACCTAAT
GAATTGCTATTTGTCTTTGTTTTCAAAGGGATAGCCTATGACCCTTTAAGAACTCATCTAGAGCGAAGAGCAGCTGAGCA
GGTAATTACTCTGTCTCGATTTGTTTTGAACGCATAATATAATCTATACATCAGAACCTAGCAAGCTTAAGGAAAACAGT
CAAATTCGCAGTAATTACATAGATAAGTCCATACTTTTGTGAAATCTGTAGCAAAAATAGAATAAAAAATATTAGATCTG
TCATGTGATGTTCTAACAATTACACATCTTTCCCTTATTAAGTGCCCATTTTTCCACAAGATGCTCACCTGCATGGCTAC
AGGAAAATCATCCTAAAAATCTAACAATAATGTTGATGCCATTGACGAGAAATTTTCACTAAGGTGTGGTCACAGTCATG
GCTATCATGTAGGTGTTTAGCTGCTGCTGCATTAAGTCTAGATTTTAAGAATCCTAATGTCTAATCATTTCGATAGAAAA
GGAACCACAAGTCTTAAAACACCTTACTATGTTGCTTAGTTAGTATACCGTAATATATGTGTCGATAGGAGTTAGGATAT
AATTTTGTGAGATTTCTGATAATCTTTTGAGTTGCCAATATGCAATTTGATTGCTTTGTTTACAATAAGTGGTATATAAG
TTATGCTATGTTTCTTCCATGAATCCTGTGCCATCGATTATACTAGCAAAGAACTTTCTAACAACTATAGATATTACTTC
AACTATTATTGGTTTCATTTCATACAATTTTGGCAATACCAGCAATTAATTACTGAAGTTATTTATGGTTCCATGGCACT
AAATAAGTGTCATAGAATTTTAGTTTGCATAACTTCAGTTGTTTAAAATTGGGAACCCATTGACCATGCAGATTTTTTGT
AAACCAACTTGACTCCCCTGCATTGCTATCCCACTAAATAATTATGGTACTCGAAAAGTTAATGTCAAATCTAGGAACTA
AAATCACACTATCTCTTAATCTCCTAACAACTTTAAGAATAAAGGACTCCAAAACTCCATCTTTTACCTGACAAAGGAAG
TAGGGAAGGAGAAAAGGCTGGACAAAATGGTGATCCGGGTTGTTTTTTACTTTACCATCAACGTTTATCAATTACTCCAT
TCCAAATTATAAGACGCCTTGAAATAGAATGCTTCAAGTTAAAGTTAGCTATTGTTTGCATGCTTCTTTATTTATATTTT
ATTGTCCAATTTCCCTGACTATTGGGTTAATTTCTGTCTCAATCTGTTAATTTTTTTCTTCTGTTTTGTGACAGTTGGAT
ATATTCAAGGAAATGGTATCTTTGCGTGTATTTGAGAGGTACATGATAAGTGTAATGGTTTCACGTTGTCTGCAGATTTT
GTTTGGATATTCACTAGATGAGTAATTTCACCATGGTTTAATTCTAACAGTTGCTCTTATTACATCCAAGACTGCCAAGT
TCTACTGTAATATTGTCTTGTGTCCTATCAAAGCAAAGAAATATGTTGACATGCTTTGGTTGTTCTGTTGAAATAATATT
CCCAGTATCATATATCCCCCTTCTATTTCTTCTGAGCAAAATGCTTACCAGTTTGAACATAGTTTGGTGCTGATGTTCTT
GTGATGACTGGACTACTCATCCGTTTTTTTAGTTAATTCTGCTTTTTGCAGGATTTTTAGTGAACTCCTATACTGTGAGC
ATTGGACTACACATTATGTTTTTTTGCAGGATTACAAGTTCATTCTGTTTTTCAGGTAAGCAGCCAATTAAGGACAAAAA
TTCCAAAAAAAGTATTCTCTCTTCATTCTTGGAGTAAAAACTGAGAACCAGGTGGTTTGCTCTCATGAACCAAAACTGGT
TAACTACTAATATTCATGTGCCGATACAAATATTCTCTGATTGCACCACAAATGTTGGGTCATTCACAATTCTGTTTCCA
CCTACGATATCTTGTACTAATGGTGCTTTTGTTGGTTTCTGTAAATTGGCTGGTGGACAGATAGTTGGCTATATTGCTGA
CAGATAGCTGGTTGCACAATTACCTGTTGATAAGGCGATTGAACTGTGGGGTTTTAGCAGGGAGTTTGAAGTCCTATTTG
GTGCCAAGCTCCTAAACTGAGAAGGCACAGTAGGCACACAAGTAACTATTGTTTCTATGCTCCAAACTTAGATGTTCTAA

GCTCTCCATGATTAGGAAATATCTATTGTTTCTGTGCACCAAATCCAAGTTGCAGGTACCTCCAAGTTTTTATTTAATGT
TGTTAATCTACTTCAATATGGTCTAGCTACAAGTTGATGTGAACCTATGATTGTGTTATATAGGGCTTCAATCCTAGCAT
TGGTCACGTGTTATGACAGTGTGGTCGAGCATGAGATTTGAACCACTAATTGTGTTATGTCGAGCTTGTATAAGCTATCC
TTTATTATCTATCCTTATTATCTTCAGATACATGTGATCAGCGATCATCTATAGAGTAATTTGAACTCTATGTGCTGTAG
ACTTTTTACTCATAACCTCCACATTCCTAAACTTATGTTTTTTAAGTTTGAGATGATGATGTGCTTATGTGAAGTTAATG
CTGACAACAATACTATTGGATGGTAAGCTAAGATATTTCATTTTCAACTAATATCATATAGATACTTGGTTAATTGAGAA
TTAGTCAGTTGTGTATTGCACAACTAAGAAGCATACAAGTGTTTATGCCTCTGATATGTTTGTCACCTTTTACTGGTCTA
GAATCGATGAAAACATTAGCTTTCTTAGCCATAAGAGTTTTTTGTGCTTCCTTCTATATCACTATTTGTTGGGAACTTTG
CGCTTTTAACTATGGTGGAGCAATATGGGCATGTCGGCGCTACAGAAAGTTGTAGCAGCGGTAGTCCATGATGTCAACGC
TGAGGGCTGGGATCGAGGATTGGAGGGGTGAGAGGCGGGCCCGACGACGGTGGGGGTGGGCGTGGGCGTGGAGGAGTGTT
CGGTGCTGCGGGAGGTGGTGGTGGCAGCGTAGTCCATGGCCATGATGATGTGTCTCCAGCTTGAGAAGGCCGAGGTCCAA
ATGGAGCTCCACCAGTTCCACTGAGGTCAGTTTCATTCATTTCATGCAGTCGAGTGCCACTATGTAATACCTATGTTCAT
TTTCTAACTGAGGTTCTAGCATATTTAATTAGAATGTTTTTGTTGCATGTTTCATTGTTTTTGTTTCTCTAAAGATTCTG
GGCGCCTCCACGACGCCGGTAAACATAGATAAGTGGATAAAACAACAAGCTATTTGGATAAAAAGCAGGGGACAGAGTTA
G
>GRMZM2G066020_T01 cDNA
CCTCCACCTCCGGGCGCCCCGGCCACCACGACCCTGAGCCTGATGCCTGGCGCGGACGTCGGCGCCGACGAGCAGCAGGA
GCCGGCCGCCGCGAGGGCCGAGGAGATGCCCGAGCCTACCCCAGCCCCCGCCGCGGCGCCGCCGCTCACCATCTTCTACG
GTGGCACGGTGGTGGTGTTCGAGGACTTCCCGGCGGAGAAGACGGCCGAGGTCATGCGCCCCGCGGCCGGCGACGACCTG
CCCATCGCCCGGAAGGCGTCGCTGCCGTGGTTCCTGGCCAAGCGCAAGGACCGCCTCGTCGAGCGCGCGCCCTACGCCCG
CCCGTCGTCCCCCGCGAAGGAGCCGGAGAAGAAGACGAATGAGCACCCTAATCTCTGTTCTCCTCGGTTCCCGCGTGTGT
GGAAAAGCAGCGGGATAGCCTATGACCCTTTAAGAACTCATCTAGAGCGAAGAGCAGCTGAGCAGTTGGATATATTCAAG
GAAATGGTATCTTTGCGTGTATTTGAGAGGATTTTTAGTGAACTCCTATACTGTGAGCATTGGACTACACATTATGTTTT
TTTGCAGGATTACAAGTTCATTCTGTTTTTCAGATGTTCTAAGCTCTCCATGATTAGGAAATATCTATTGTTTCTGTGCA
CCAAATCCAAGTTGCAGTTTGAGATGATGATGTGCTTATGTGAAGTTAATGCTGACAACAATACTATTGGATGCTTGAGA
AGGCCGAGGTCCAAATGGAGCTCCACCAGTTCCACTGAGATTCTGGGCGCCTCCACGACGCCGGTAAACATAGATAAGTG
GATAAAACAACAAGCTATTTGGATAAAAAGCAGGGGACAGAGTTAG
>GRMZM2G066020_P01 peptide
MPGADVGADEQQEPAAARAEEMPEPTPAPAAAPPLTIFYGGTVVVFEDFPAEKTAEVMRPAAGDDLPIARKASLPWFLAK
RKDRLVERAPYARPSSPAKEPEKKTNEHPNLCSPRFPRVWKSSGIAYDPLRTHLERRAAEQLDIFKEMVSLRVFERIFSE
LLYCEHWTTHYVFLQDYKFILFFRCSKLSMIRKYLLFLCTKSKLQFEMMMCLCEVNADNNTIGCLRRPRSKWSSTSSTEI
LGASTTPVNIDKWIKQQAIWIKSRGQS

AGPv4:

Zm00001d021274 (zim31 - ZIM-transcription factor 31)

Chr7: 147534788..147542179

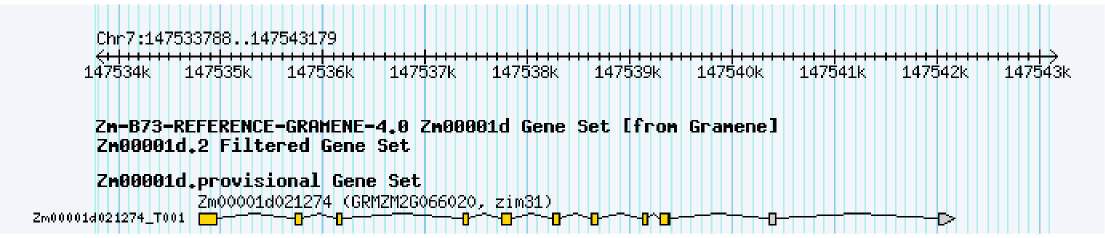

>Zm00001d021274 Genomic DNA

ATGCCTGGCGCGGACGTCGGCGCCGACGAGCAGCAGGAGCCGGCCGCCGCGAGGGCCGAGGAGATGCCCGAGCCTACCCC

AGCCCCCGCCGCGGCGCCGCCGCTCACCATCTTCTACGGTGGCACGGTGGTGGTGTTCGAGGACTTCCCGGCGGAGAAGA
CGGCCGAGGTCATGCGCCCGCGGCCGGCGACGACCTGCCCATCGCCCGGAAGGCGTCGCTGCCGTGGTTCCTGGCCAAGC
GCAAGGACCGCCTCGTCGAGCGCGCGCCCTACGCCCGCCCGTCGTCCCCCGCGAAGGAGCCGGAGAAGAAGACGGTCAAG
CCAGCCTCGGCCTCAGCCTCATGCTCAACAAAATGACTGTGTGACTGCTGTTGCCTGAACCGAACTGGCCACCTCTACGC
TCATTCCTCTCACGTTTCTGAAGGGTGCAGAGTAGCCGGTCGACTAATCGAGCTCCCTACGCCTCCTGCGCACGGCCTAA
AACTCCCTGCTCTTGCCTGCACGCCTCGCTCTGCTTAGCATCGGAGCGCACGGACGCTGCAGCCATGTTTATTCCTTGCA
GCGAAACGCATGGGCTTGAAGGAGTTCAAAATGGGGTATGAATCATCTCAGTTTGACTTTTGTTTTAAATCCATTGATTA
TTGACCCTTATGTCTAAACTGCCCTAAGATACTTATTTCTGGCTTCATAAATCACAAAACAGAGCCAAAGCTATAGTCAA
TGCAGAAAGTAAACCGAGCTAACAAATAAAAAACAATGCTACGACACACAAGATCAACAAGCTAGAGCCTAAATTAACAT
CAGTAATTCAAATCCACTTAAGTAATATAGTGTGTGATGTAAGCCAAAAAAACCAGATGCAACCATTCTAGCCAAAGACT
CTTTAGCCCATAAGTAATTCATGTTTTTGTAGATGCTCGACATGGTTCTTGATGATCTATAGAATGAGCACCCTAATCTC
TGTTCTCCTCGGTTCCCGCGTGTGTGGAAAAGCAGCGGTGAGGTTCAATAACTGGTTACACATTGTTTTTGATTGCTCA
GTGCAACCATGATGTTTGATGAAATGTTCAACTCTGCTATTGCACATTTGCACGCTGCAGTGCAGTGTTGAAATGTAGTT
TACTTTTTGTTTGAGAATTCACATGACAGGTCAGTTTGTACATTTTTAAGAGTCCAAATTAGGTTCGTTCTGCATAATAC
ATGTGGTGTTTAATACCATTGTATTCCGAAAATGATATGAGCTTACCTTGCTGTATCTCCATGTATATCATGAAAATTTA
CTTAAAATTGGACGCTTGATTGATTGAAAGACCTAATGAATTGCTATTTGTCTTTGTTTTCAAAGGGATAGCCTATGACC
CTTTAAGAACTCATCTAGAGCGAAGAGCAGCTGAGCAGGTAATTACTCTGTCTCGATTTGTTTTGAACGCATAATATAAT
CTATACATCAGAACCTAGCAAGCTTAAGGAAAACAGTCAAATTCGCAGTAATTACATAGATAAGTCCATACTTTTGTGAA
ATCTGTAGCAAAAATAGAATAAAAAATATTAGATCTGTCATGTGATGTTCTAACAATTACACATCTTTCCCTTATTAAGT
GCCCATTTTTCCACAAGATGCTCACCTGCATGGCTACAGGAAAATCATCCTAAAAATCTAACAATAATGTTGATGCCATT
GACGAGAAATTTTCACTAAGGTGTGGTCACAGTCATGGCTATCATGTAGGTGTTTAGCTGCTGCTGCATTAAGTCTAGAT
TTTAAGAATCCTAATGTCTAATCATTTCGATAGAAAAGGAACCACAAGTCTTAAAACACCTTACTATGTTGCTTAGTTAG
TATACCGTAATATATGTGTCGATAGGAGTTAGGATATAATTTTGTGAGATTTCTGATAATCTTTTGAGTTGCCAATATGC
AATTTGATTGCTTTGTTTACAATAAGTGGTATATAAGTTATGCTATGTTTCTTCCATGAATCCTGTGCCATCGATTATAC
TAGCAAAGAACTTTCTAACAACTATAGATATTACTTCAACTATTATTGGTTTCATTTCATACAATTTTGGCAATACCAGC
AATTAATTACTGAAGTTATTTATGGTTCCATGGCACTAAATAAGTGTCATAGAATTTTAGTTTGCATAACTTCAGTTGTT
TAAAATTGGGAACCCATTGACCATGCAGATTTTTTGTAAACCAACTTGACTCCCCTGCATTGCTATCCCACTAAATAATT
ATGGTACTCGAAAAGTTAATGTCAAATCTAGGAACTAAAATCACACTATCTCTTAATCTCCTAACAACTTTAAGAATAAA
GGACTCCAAAACTCCATCTTTTACCTGACAAAGGAAGTAGGGAAGGAGAAAAGGCTGGACAAAATGGTGATCCGGGTTGT
TTTTTACTTTACCATCAACGTTTATCAATTACTCCATTCCAAATTATAAGACGCCTTGAAATAGAATGCTTCAAGTTAAA
GTTAGCTATTGTTTGCATGCTTCTTTATTTATATTTTATTGTCCAATTTCCCTGACTATTGGGTTAATTTCTGTCTCAAT
CTGTTAATTTTTTTCTTCTGTTTTGTGACAGTTGGATATATTCAAGGAAATGGTATCTTTGCGTGTATTTGAGAGGTACA
TGATAAGTGTAATGGTTTCACGTTGTCTGCAGATTTTGTTTGGATATTCACTAGATGAGTAATTTCACCATGGTTTAATT
CTAACAGTTGCTCTTATTACATCCAAGACTGCCAAGTTCTACTGTAATATTGTCTTGTGTCCTATCAAAGCAAAGAAATT
GTTGACATGCTTTGGTTGTTCTGTTGAAATAATATTCCCAGTATCATATATCCCCCTTCTATTTCTTCTGAGCAAAATGC
TTACCAGTTTGAACATAGTTTGGTGCTGATGTTCTTGTGATGACTGGACTACTCATCCGTTTTTTAGTTAATTCTGCTTT
TTGCAGGATTTTTAGTGAACTCCTATACTGTGAGCATTGGACTACACATTATGTTTTTTTGCAGGATTACAAGTTCATTC
TGTTTTTCAGGTAAGCAGCCAATTAAGGACAAAAATTCCAAAAAAAGTATTCTCTCTTCATTCTTGGAGTAAAAACTGAG
AACCAGGTGGTTTGCTCTCATGAACCAAAACTGGTTAACTACTAATATTCATGTGCCGATACAAATATTCTCTGATTGCA
CCACAAATGTTGGGTCATTCACAATTCTGTTTCCACCTACGATATCTTGTACTAATGGTGCTTTTGTTGGTTTCTGTAAA
TTGGCTGGTGGACAGATAGTTGGCTATATTGCTGACAGATAGCTGGTTGCACAATTACCTGTTGATAAGGCGATTGAACT
GTGGGGTTTTAGCAGGGAGTTTGAAGTCCTATTTGGTGCCAAGCTCCTAAACTGAGAAGGCACAGTAGGCACACAAGTAA
CTATTGTTTCTATGCTCCAAACTTAGATGTTCTAAGCTCTCCATGATTAGGAAATATCTATTGTTTCTGTGCACCAAATC
CAAGTTGCAGGTACCTCCAAGTTTTTATTTAATGTTGTTAATCTACTTCAATATGGTCTAGCTACAAGTTGATGTGAACC

TATGATTGTGTTATATAGGGCTTCAATCCTAGCATTGGTCACGTGTTATGACAGTGTGGTCGAGCATGAGATTTGAACCA
CTAATTGTGTTATGTCGAGCTTGTATAAGCTATCCTTTATTATCTATCCTTATTATCTTCAGATACATGTGATCAGCGAT
CATCTATAGAGTAATTTGAACTCTATGTGCTGTAGACTTTTTACTCATAACCTCCACATTCCTAAACTTATGTTTTTTAA
GTTTGAGATGATGATGTGCTTATGTGAAGTTAATGCTGACAACAATACTATTGGATGGTAAGCTAAGATATTTCATTTTC
AACTAATATCATATAGATACTTGGTTAATTGAGAATTAGTCAGTTGTGTATTGCACAACTAAGAAGCATACAAGTGTTTA
TGCCTCTGATATGTTTGTCACCTTTTACTGGTCTAGAATCGATGAAAACATTAGCTTTCTTAGCCATAAGAGTTTTTTGT
GCTTCCTTCTATATCACTATTTGTTGGGAACTTTGCGCTTTTAACTATGGTTGAGCAATATGGGCATGTCGGCGCTACAG
AAAGCTGTAGCAGCGGTAGTCCATGATGTCAACGCTGAGGGCTGGGATCGAGGATTGGAGGGGTGAGAGGCGGGCCCGAC
GACGGTGGGGGTGGGCGTGGGCGTGGAGGAGTGTTCGGTGCTGCGGGAGGTGGTGGTGGCAGCGTAGTCCATGGCCATGA
TGATGTGTCTCCAGCTTGAGAAGGCCGAGGTCCAAATGGAGCTCCACCAGTTCCACTGAGGTCAGTTTCATTCATTTCAT
GCAGTCGAGTGCCACTATGTAATACCTATGTTCATTTTCTAACTGAGGTTCTAGCATATTTAATTAGAATGTTTTTGTTG
CATGTTTCATTGTTTTTGTTTCTCTAAAGATTCTGGGCGCCTCCACGACGCCGGTAAACATAGATAAGTGGATAAAACAA
CAAGCTATTTGGATAAAAAGCAGGGGACAGAGTTAGAGGTCGGTCATGCTAATTCCTTTTTTTCCTTGGAATTGTTAAAA
TAAGGGTAGGAGAAATATTTTTCTGGAACTCAAAGAAACAAAAACTTCTAAATAGCACCAAGAACTATGCTCTTCAGCCC
AAAGCCAGGTGTTATTCACGAAAATAGTGGCAACAATATCTTTTAGTCCTCTATAGAAGATGGAGGAGTTGGCTTTTGAG
TTGGTGTAGGCTTCTCAACTGATTTTGTAATTGTTTGATTTACTACTTTTGTAATTGTTTGATTTACTACTTTGAATGCC
AGCAACGATATCTTTTAGTCTCTCTACAGAAACAGAGGAGTTGGCTTTTGAACTGGTGTAGGCCTCTCAACTGATTTTGG
AGCTTCCACATTGCCAAAGAGATAGTCTAAGGAACTCTGACCAATATATTTAATATGACAAAATATCATCGTAACACTAA
TATTCACTATGAGATGCTAACAACAAAGTGAAGTTTTACGGACAAATAAAAGAGCACGAATGGAACTTCTTTGGTTGTTT
TAAGCGTGCATGCTGTCTTTGGTACTTTATTATTATGTTGCGTGCTGCTTGAGCTTTTTAGTTCCTCCAGGGATAACGGT
GGTCCAGCATTTGAAACTGATTTTGCAGACCAATTGAAAAACTTCAGTAAGATGGGTCATGGTGTTAGCAGTGGGGGTGG
TTAGAGTTCCTTAGGCTATCTCTTCTATGACATATAGAATACAAAAAATCTTTGTTGTACATGCATTCAAGCATTCAAGT
TTTTGTAGTGTGCATATTAACTCAATATCTTCAAATGAGTAGTATTCAATCCAAAGAAGCAGCATTGGTTGTTATGAAAA
GAAAGTGACCCTGGAATACACCGCCACAATATTACAGTTTTATGAACCCATATATTGCAGTTACAATGTTATTGCAGGCT
TGATTTGATCAGTTTATATTTTTTCTTAATTGCATTGTGTTGCTGGACAACAGTTTTGCTTAAGTCTTCTGGATGGCACA
CTCCTGTCGTACCACAAATCAGAACCAATGCGTCAATAGGTAAGCATTATTTATTTGCTTAAATCTTTTAAGGTGTTTGT
AGTACTGGGACTGAATTTCTGAAGCATTCTAACTTTGAAATAATCAAAGGTTAAGGGTGCAACATTGTAACATACTACTG
AAATATGTGGTTCAACATCTCTCAGTAACAATCTCCTCTGGACATTTGATAAAATTTGTGTAGTAAAGGTGCAAGGATGT
TTAAAATTGGAAAACAGCGTCCAATAGGCTCAGGTATGGGATTGTTGGTCCTGATATATTCGTTGGAAATATTTGGGTAT
CTTCCTTCTATGAAATTTGTTTAACCACTAACCAATATTTTGAAAGCCATCAAAACATCTTCCTTGAGGTATATGACATT
TTTATTTGTTATATCTCTTCTCATTTTCAATGCCTAGCAAGCTATGATATGGGAAACATGTATTAGTTCATTTGTCATTT
TCATTGACTGGTGCAAGTATATCTAACAAATTTGAGTTGAGTGAGTAGTACTAACTGAATAGTAGTTGGTCAATGGGAAT
TCCTCATTGTTGGTTCGATTGACTTATGAAGAAAAACACAGACATTTTCTAATATCCAATAAGTTCTCTGAATCAATAGA
TGTACCCCTTACAAACTTCAATAAGAGGGAAAATAACTTCCTTTCCCTACTCTACATTCCCTTTGGTTGCTGCTTCAAAT
TCAAGACCAAGCAACCTGAACATAACGAACTTGGTCCTTCCAGGATGGTGGTAAATTTAAGACCAAAACGACAGTAAAAG
GCTGGCAGACTAAATTCAAATTCATTCTTCTAGGACTGGAAGAAAACAATAGATTTAAACATAGGAGATGGTTTGATAGT
GGTAATAGAGATAAGAATCTCAGAGCATTATAGCTGTATGGGACTCTAGATCGATTGCAGTTATCGGTAATATAGGTATA
TATATAGTAGTTAGAGTACTTGTTACCAAGTAGCACGACATGGCGTAAGCACATAACTGTTGACTGTTGAGCCATAGTAA
GAAGTGGAAAGGGAACTGAGAAATGAATAAGTAGCTAGCATCGACCGATTACTTACCTATATGAGGAATAACAGTTTTGC
AGGAATTGACCGTTCAATATCAAGATCCTTTGTTGAATACCCTCCACTCAAAAGAAAGATTGATAAAAATAGAAACAATA
TTATGAGTTCATGCTAAATAGTACAGAATAAACACCAAAATCTGTACCACTCAGCATTTCGGGAAGCCTTCAAGTTGAAT
TTATAACAAGCAATAGCATTTAATACAGAAGCTAAAAATAAACAGGCAATAGAATTTGACCTGCTTTCTCGCTCTTGATC
GCACAACTGATTTCCTATTATTTTTCATCCTCTTCTGCCTTCTTTCAACCACCTTATCCACTACCTACCCTAAGGCACTA
CGCTTGCACCTAGGTGTCTGAGGGTCCAAAACTGCACTGAATGATGGCGAAGTAATCTTGTCATCAGAGTAAATTGAATC

CAAAACAACACCTGTACCAAGAGCCTGCTGTAGAACTCACATTTTTTGTTTAACTTATATTTTGTGTTAGAAGTCATGGT
AAATATATTTTTTAAACTATGTGGTCATTTATGCAGGCCTAATTCACGTGTTATAAAGTTAATTAAATGTTTGGGTACGG
AGGTGAAGCCAAACTAGAGGCATATATTGTGCATGAATAAGGTTGACCTGGTGGAAGACAAGGACCTCCTGATAGTTGCA
AAAGAAATCGAAGATCTTCTTGTGTATGAAAG
>Zm00001d021274_T001 cDNA
ATGCCTGGCGCGGACGTCGGCGCCGACGAGCAGCAGGAGCCGGCCGCCGCGAGGGCCGAGGAGATGCCCGAGCCTACCCC
AGCCCCCGCCGCGGCGCCGCCGCTCACCATCTTCTACGGTGGCACGGTGGTGGTGTTCGAGGACTTCCCGGCGGAGAAGA
CGGCCGAGAATGAGCACCCTAATCTCTGTTCTCCTCGGTTCCCGCGTGTGTGGAAAAGCAGCGGGATAGCCTATGACCCT
TTAAGAACTCATCTAGAGCGAAGAGCAGCTGAGCAGTTGGATATATTCAAGGAAATGGTATCTTTGCGTGTATTTGAGAG
GATTTTTAGTGAACTCCTATACTGTGAGCATTGGACTACACATTATGTTTTTTTGCAGGATTACAAGTTCATTCTGTTTT
TCAGATGTTCTAAGCTCTCCATGATTAGGAAATATCTATTGTTTCTGTGCACCAAATCCAAGTTGCAGTTTGAGATGATG
ATGTGCTTATGTGAAGTTAATGCTGACAACAATACTATTGGATGCTTGAGAAGGCCGAGGTCCAAATGGAGCTCCACCAG
TTCCACTGAGATTCTGGGCGCCTCCACGACGCCGGTAAACATAGATAAGTGGATAAAACAACAAGCTATTTGGATAAAAA
GCAGGGGACAGAGTTAGAGTTTTGCTTAAGTCTTCTGGATGGCACACTCCTGTCGTACCACAAATCAGAACCAATGCGTC
AATAGGCCTAATTCACGTGTTATAAAGTTAATTAAATGTTTGGGTACGGAGGTGAAGCCAAACTAGAGGCATATATTGTG
CATGAATAAGGTTGACCTGGTGGAAGACAAGGACCTCCTGATAGTTGCAAAAGAAATCGAAGATCTTCTTGTGTATGAAA
G
>Zm00001d021274_P001 peptide
MPGADVGADEQQEPAAARAEEMPEPTPAPAAAPPLTIFYGGTVVVFEDFPAEKTAENEHPNLCSPRFPRVWKSSGIAYDP
LRTHLERRAAEQLDIFKEMVSLRVFERIFSELLYCEHWTTHYVFLQDYKFILFFRCSKLSMIRKYLLFLCTKSKLQFEMM
MCLCEVNADNNTIGCLRRPRSKWSSTSSTEILGASTTPVNIDKWIKQQAIWIKSRGQS*SFA*VFWMAHSCRTTNQNQCV
NRPNSRVIKLIKCLGTEVKPN*RHILCMNKVDLVEDKDLLIVAKEIEDLLVYEX

ZmJAZ20 (TIFY27)

B73 RefGen_v3:
GRMZM2G089736 (zim23 - ZIM-transcription factor 23)
Chr7: 165496239..165498650

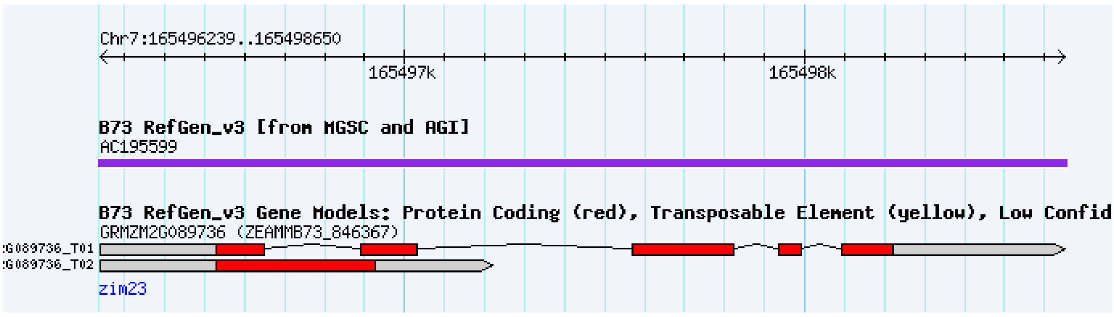

>GRMZM2G089736 Genomic DNA
AGCGAATACGAGGATAAACAATTTCGAGCAATCCGTTCCCGTGGGTTTTTAGTCGCGGCCTCACGGGATTTGGGTGGAAA
CCGCGGGGGGTCCTTTATAAGCGCGTGGGCGCGCGGCGTGCTCACACGAGCCCCCCCGCGTTCACGAGCCAGAAGAGAAG
AGAGGCTAACCACCGCAGCGGCTGCGACCTGCGAGTGCGACAACCTCCGCGGAGGAGTTGGAGCCGGCGGCGGGGATATT
TGCTTGCCCGGCTGGGGTTGCGGCGGAAGGAGGGAGAGACAATCGGCGATGGCGGCGTCCGCGAGGCCCGGGGAGAGGGC
GACCAGCTTCGCCGTCGCGTGCAGCCTCCTCAGCCGCTTCGTCCGCCAGAACGGCGTCGCGGCTGCCGACCTAGGCCTCA
GGATCAAAGGTGAGCGGCGGAATCCGGCGGCGGCCTTGTCGAGGCCGGGCTCCTTCTGCTCGGTGGTGGGAGGATTTCGA
AATCCGGGAGGGTGTGTGGGGGGTCCAGTTTCGTTTCCAGTGGGGAGCGTGTTGTGCTGGTGTGAGATCTGGGCGGGGCC

TTTGTTATCCGGTTTGAAACCACGTTTTCGGGCTTCGAATTTTACGGTCCCTTATCCAGATGTTTTGTTTTGTTTTGTTC
CCGTTCTCAGGCGAGGTCGAGCAGCAGAGGACGCCGGCGACAACTAACTCGCTCCCCGGAGCGGAGGGCGAGGAGGTCGA
AAGGAGGAAGGAGACCATGGAGCTGTTCCCGCAGAGCGTCGGGTTCAGCATCAAGGATGCTGCTGCCCCTAGGTGCGTAC
CACTGCTGCTTCACATCATCTAAATCCATTTCGATTTTGGTTTGCGCAACAGTTGTTACGTAGTAGCACAGCAGAACTCA
AGCCTAGCTGTCGTAGGTCTCCTGGACAAAAGGCCAAGCAATTTGACTTTGTAACATAGAGATCTGAGTTTAATGAGTGT
TTATAGGTGGAACATGTGGTATAAAATCTAGTTGAGCCGGATGGCAAATGACAGAGCATGTTATTACGTATGTGTAAAGC
TAAAGCATCCCTTGCTGTTCAGTCCGTGTTGGTATTCCTTGGTCAAAGCTGTATCTTTGTAATCTTAAGAAGTTACCGCC
TTTGCTGAAACACCGTGTTCTGCAAGGTAGCACAATGTTTCCTTTTTATCAGGCAAAGCAACCAACATGCTCAGAGATTG
TACCTAATGTTTATCATTAACTGCCAAGGATTATTAACAGGGAAAAAAACAGAGTGGATAATTGATTGGCTAAGCTGAAG
TAGCCTACTACATGCACTGTTCTTAGTGTTCTTCTCTGTAATATTTGTCAGGGAGGAGCAAGGAGATAAAGAGAAGCCTA
AGCAGCTCACAATCTTCTATGGCGGGAAGGTGCTGGTATTTGACGATTTCCCCGCCGACAAGGCAAAGGATCTGATGCAG
CTGGCCAGCAAGGGCAGCCCAGTGGTACAGAACGTTGCTTTGCCTCAACCCTCTGCAGCAGCTGCTGTCACTACTGACAA
GGCCGTGCTGGACCCGGTCATCAGCTTGGCCGCTGCTAAGAAGCCTGCTCGCACAAATGCTTCTGGTATAAACACTCTCC
CTCTGTTTCTATTCTTACCTAGCTGAAGTTCTAAAATATGATTGCTGGACTTTGTTATGCCGATTATTGATCGAAACGTG
CCTTTTCTTCTGTAGATATGCCTATTATGAGGAAGGCTTCTCTTCACCGCTTCCTTGAGAAGAGAAAAGATCGGTAAGCT
GTGGTTGCTGTGATTATTATTTTCAGTATGGCGATTTCCTTTCTTTTCCAGTGTTGTATTTGGAACTAATTTGTGCACTT
CTGGTTTTCTTCAGCCTCAATGCAAAGACACCATATCAAACTGCTCCTTCAGATGCCGCACCAGTCAAGAAGGAGCCTGA
GAGCCAGCCATGGCTCGGATTAGGACCGAATGCCGTGGATTCCAGCCTGAACCTCAGCTAGTACACAACAACACAGCCAT
AATAAGACGGCACAGCAACGGCACCCACCATGACCTGGAATTTGCTTTGCCACTAGATCGAGTTCAGAAACCATAAATAT
TCTTAGTTATCGTGTTTTAGTGTTCGCTTATGTCGTGGTTCTACGCCCTCTTGTCGCATAGTTCTGGATGTAAAGAAAAG
TAGCCAGTCATGACCATCACCGGGAGTCGGGGAGAAGTTTTTGGAGGGTGTTAACACACGGCGACTCTGAATTGGCCATG
CTAAGGAAAAGGATAGTGCAATGTGAGGTATATGTATTTTCTGAAAGCCAGATCGTTTGGGTCTAGCCCTGATGTATGCA
GAACAAGTGATTATTGTAATGGATCCTGATTATCCTCATCATCCTATTGTTGTTTTCTGATCACATGGCTTCACCAACCC
AAGTTTGTTTGG

>GRMZM2G089736_T01 cDNA

Chr7: 165496239..165498650

AGCGAATACGAGGATAAACAATTTCGAGCAATCCGTTCCCGTGGGTTTTTAGTCGCGGCCTCACGGGATTTGGGTGGAAA
CCGCGGGGGGTCCTTTATAAGCGCGTGGGCGCGCGGCGTGCTCACACGAGCCCCCCGCGTTCACGAGCCAGAAGAGAAG
AGAGGCTAACCACCGCAGCGGCTGCGACCTGCGAGTGCGACAACCTCCGCGGAGGAGTTGGAGCCGGCGGCGGGGATATT
TGCTTGCCCGGCTGGGGTTGCGGCGGAAGGAGGGAGAGACAATCGGCGATGGCGGCGTCCGCGAGGCCCGGGGAGAGGGC
GACCAGCTTCGCCGTCGCGTGCAGCCTCCTCAGCCGCTTCGTCCGCCAGAACGGCGTCGCGGCTGCCGACCTAGGCCTCA
GGATCAAAGGCGAGGTCGAGCAGCAGAGGACGCCGGCGACAACTAACTCGCTCCCCGGAGCGGAGGGCGAGGAGGTCGAA
AGGAGGAAGGAGACCATGGAGCTGTTCCCGCAGAGCGTCGGGTTCAGCATCAAGGATGCTGCTGCCCCTAGGGAGGAGCA
AGGAGATAAAGAGAAGCCTAAGCAGCTCACAATCTTCTATGGCGGGAAGGTGCTGGTATTTGACGATTTCCCCGCCGACA
AGGCAAAGGATCTGATGCAGCTGGCCAGCAAGGGCAGCCCAGTGGTACAGAACGTTGCTTTGCCTCAACCCTCTGCAGCA
GCTGCTGTCACTACTGACAAGGCCGTGCTGGACCCGGTCATCAGCTTGGCCGCTGCTAAGAAGCCTGCTCGCACAAATGC
TTCTGATATGCCTATTATGAGGAAGGCTTCTCTTCACCGCTTCCTTGAGAAGAGAAAAGATCGCCTCAATGCAAAGACAC
CATATCAAACTGCTCCTTCAGATGCCGCACCAGTCAAGAAGGAGCCTGAGAGCCAGCCATGGCTCGGATTAGGACCGAAT
GCCGTGGATTCCAGCCTGAACCTCAGCTAGTACACAACAACACAGCCATAATAAGACGGCACAGCAACGGCACCCACCAT
GACCTGGAATTTGCTTTGCCACTAGATCGAGTTCAGAAACCATAAATATTCTTAGTTATCGTGTTTTAGTGTTCGCTTAT
GTCGTGGTTCTACGCCCTCTTGTCGCATAGTTCTGGATGTAAAGAAAAGTAGCCAGTCATGACCATCACCGGGAGTCGGG
GAGAAGTTTTTGGAGGGTGTTAACACACGGCGACTCTGAATTGGCCATGCTAAGGAAAAGGATAGTGCAATGTGAGGTAT
ATGTATTTTCTGAAAGCCAGATCGTTTGGGTCTAGCCCTGATGTATGCAGAACAAGTGATTATTGTAATGGATCCTGATT
ATCCTCATCATCCTATTGTTGTTTTCTGATCACATGGCTTCACCAACCCAAGTTTGTTTGG

>GRMZM2G089736_P01 peptide
MAASARPGERATSFAVACSLLSRFVRQNGVAAADLGLRIKGEVEQQRTPATTNSLPGAEGEEVERRKETMELFPQSVGFS
IKDAAAPREEQGDKEKPKQLTIFYGGKVLVFDDFPADKAKDLMQLASKGSPVVQNVALPQPSAAAAVTTDKAVLDPVISL
AAAKKPARTNASDMPIMRKASLHRFLEKRKDRLNAKTPYQTAPSDAAPVKKEPESQPWLGLGPNAVDSSLNLS
>GRMZM2G089736_T02 cDNA
Chr7: 165496239..165497218
AGCGAATACGAGGATAAACAATTTCGAGCAATCCGTTCCCGTGGGTTTTTAGTCGCGGCCTCACGGGATTTGGGTGGAAA
CCGCGGGGGGTCCTTTATAAGCGCGTGGGCGCGCGGCGTGCTCACACGAGCCCCCCCGCGTTCACGAGCCAGAAGAGAAG
AGAGGCTAACCACCGCAGCGGCTGCGACCTGCGAGTGCGACAACCTCCGCGGAGGAGTTGGAGCCGGCGGCGGGGATATT
TGCTTGCCCGGCTGGGGTTGCGGCGGAAGGAGGGAGAGACAATCGGCGATGGCGGCGTCCGCGAGGCCCGGGGAGAGGGC
GACCAGCTTCGCCGTCGCGTGCAGCCTCCTCAGCCGCTTCGTCCGCCAGAACGGCGTCGCGGCTGCCGACCTAGGCCTCA
GGATCAAAGGTGAGCGGCGGAATCCGGCGGCGGCCTTGTCGAGGCCGGGCTCCTTCTGCTCGGTGGTGGGAGGATTTCGA
AATCCGGGAGGGTGTGTGGGGGGTCCAGTTTCGTTTCCAGTGGGGAGCGTGTTGTGCTGGTGTGAGATCTGGGCGGGGCC
TTTGTTATCCGGTTTGAAACCACGTTTTCGGGCTTCGAATTTTACGGTCCCTTATCCAGATGTTTTGTTTTGTTTTGTTC
CCGTTCTCAGGCGAGGTCGAGCAGCAGAGGACGCCGGCGACAACTAACTCGCTCCCCGGAGCGGAGGGCGAGGAGGTCGA
AAGGAGGAAGGAGACCATGGAGCTGTTCCCGCAGAGCGTCGGGTTCAGCATCAAGGATGCTGCTGCCCCTAGGTGCGTAC
CACTGCTGCTTCACATCATCTAAATCCATTTCGATTTTGGTTTGCGCAACAGTTGTTACGTAGTAGCACAGCAGAACTCA
AGCCTAGCTGTCGTAGGTCTCCTGGACAAAAGGCCAAGCAATTTGACTTTGTAACATAGAGATCTGAGTTTAATGAGTGT
TTATAGGTGGAACATGTGGT
>GRMZM2G089736_P02 peptide
MAASARPGERATSFAVACSLLSRFVRQNGVAAADLGLRIKGERRNPAAALSRPGSFCSVVGGFRNPGGCVGGPVSFPVGS
VLCWCEIWAGPLLSGLKPRFRASNFTVPYPDVLFCFVPVLRRGRAAEDAGDN

AGPv4:
Zm00001d022139 (zim23 - ZIM-transcription factor 23)
Chr7: 171049645..171052026

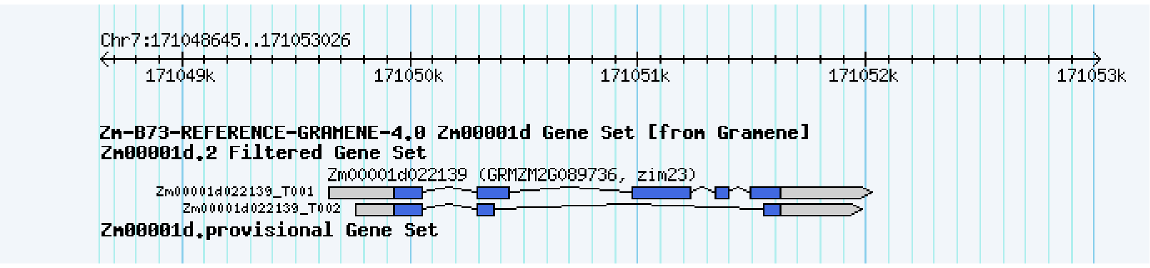

>Zm00001d022139 Genomic DNA
GAATACGAGGATAAACAATTTCGAGCAATCCGTTCCCGTGGGTTTTTAGTCGCGGCCTCACGGGATTTGGGTGGAAACCG
CGGGGGGTCCTTTATAAGCGCGTGGGCGCGCGGCGTGCTCACACGAGCCCCCCCGCGTTCACGAGCCAGAAGAGAAGAGA
GGCTAACCACCGCAGCGGCTGCGACCTGCGAGTGCGACAACCTCCGCGGAGGAGTTGGAGCCGGCGGCGGGGATATTTGC
TTGCCCGGCTGGGGTTGCGGCGGAAGGAGGGAGAGACAATCGGCGATGGCGGCGTCCGCGAGGCCCGGGGAGAGGGCGAC
CAGCTTCGCCGTCGCGTGCAGCCTCCTCAGCCGCTTCGTCCGCCAGAACGGCGTCGCGGCTGCCGACCTAGGCCTCAGGA
TCAAAGGTGAGCGGCGGAATCCGGCGGCGGCCTTGTCGAGGCCGGGCTCCTTCTGCTCGGTGGTGGGAGGATTTCGAAAT
CCGGGAGGGTGTGTGGGGGGTCCAGTTTCGTTTCCAGTGGGGAGCGTGTTGTGCTGGTGTGAGATCTGGGCGGGGCCTTT
GTTATCCGGTTTGAAACCACGTTTTCGGGCTTCGAATTTTACGGTCCCTTATCCAGATGTTTTGTTTTGTTTTGTTCCCG
TTCTCAGGCGAGGTCGAGCAGCAGAGGACGCCGGCGACAACTAACTCGCTCCCCGGAGCGGAGGGCGAGGAGGTCGAAAG
GAGGAAGGAGACCATGGAGCTGTTCCCGCAGAGCGTCGGGTTCAGCATCAAGGATGCTGCTGCCCCTAGGTGCGTACCAC

TGCTGCTTCACATCATCTAAATCCATTTCGATTTTGGTTTGCGCAACAGTTGTTACGTAGTAGCACAGCAGAACTCAAGC
CTAGCTGTCGTAGGTCTCCTGGACAAAAGGCCAAGCAATTTGACTTTGTAACATAGAGATCTGAGTTTAATGAGTGTTTA
TAGGTGGAACATGTGGTATAAAATCTAGTTGAGCCGGATGGCAAATGACAGAGCATGTTATTACGTATGTGTAAAGCTAA
AGCATCCCTTGCTGTTCAGTCCGTGTTGGTATTCCTTGGTCAAAGCTGTATCTTTGTAATCTTAAGAAGTTACCGCCTTT
GCTGAAACACCGTGTTCTGCAAGGTAGCACAATGTTTCCTTTTTATCAGGCAAAGCAACCAACATGCTCAGAGATTGTAC
CTAATGTTTATCATTAACTGCCAAGGATTATTAACAGGGAAAAAAACAGAGTGGATAATTGATTGGCTAAGCTGAAGTAG
CCTACTACATGCACTGTTCTTAGTGTTCTTCTCTGTAATATTTGTCAGGGAGGAGCAAGGAGATAAAGAGAAGCCTAAGC
AGCTCACAATCTTCTATGGCGGGAAGGTGCTGGTATTTGACGATTTCCCCGCCGACAAGGCAAAGGATCTGATGCAGCTG
GCCAGCAAGGGCAGCCCAGTGGTACAGAACGTTGCTTTGCCTCAACCCTCTGCAGCAGCTGCTGTCACTACTGACAAGGC
CGTGCTGGACCCGGTCATCAGCTTGGCCGCTGCTAAGAAGCCTGCTCGCACAAATGCTTCTGGTATAAACACTCTCCCTC
TGTTTCTATTCTTACCTAGCTGAAGTTCTAAAATATGATTGCTGGACTTTGTTATGCCGATTATTGATCGAAACGTGCCT
TTTCTTCTGTAGATATGCCTATTATGAGGAAGGCTTCTCTTCACCGCTTCCTTGAGAAGAGAAAAGATCGGTAAGCTGTG
GTTGCTGTGATTATTATTTTCAGTATGGCGATTTCCTTTCTTTTCCAGTGTTGTATTTGGAACTAATTTGTGCACTTCTG
GTTTTCTTCAGCCTCAATGCAAAGACACCATATCAAACTGCTCCTTCAGATGCCGCACCAGTCAAGAAGGAGCCTGAGAG
CCAGCCATGGCTCGGATTAGGACCGAATGCCGTGGATTCCAGCCTGAACCTCAGCTAGTACACAACAACACAGCCATAAT
AAGACGGCACAGCAACGGCACCCACCATGACCTGGAATTTGCTTTGCCACTAGATCGAGTTCAGAAACCATAAATATTCT
TAGTTATCGTGTTTTAGTGTTCGCTTATGTCGTGGTTCTACGCCCTCTTGTCGCATAGTTCTGGATGTAAAGAAAAGTAG
CCAGTCATGACCATCACCGGGAGTCGGGGAGAAGTTTTTGGAGGGTGTTAACACACGGCGACTCTGAATTGGCCATGCTA
AGGAAAAGGATAGTGCAATGTGAGGTATATGTATTTTCTGAAAGCCAGATCGTTTGGGTCTAGCCCTGATGTATGCAGAA
CAAGTGATTATTGTAATGGATCCTGATTATCCTCATCATCCTATTGTTGTTTTCTGATCACA
>Zm00001d022139_T001 cDNA
Chr7: 171049645..171052026
GAATACGAGGATAAACAATTTCGAGCAATCCGTTCCCGTGGGTTTTTAGTCGCGGCCTCACGGGATTTGGGTGGAAACCG
CGGGGGGTCCTTTATAAGCGCGTGGGCGCGCGGCGTGCTCACACGAGCCCCCCCGCGTTCACGAGCCAGAAGAGAAGAGA
GGCTAACCACCGCAGCGGCTGCGACCTGCGAGTGCGACAACCTCCGCGGAGGAGTTGGAGCCGGCGGCGGGGATATTTGC
TTGCCCGGCTGGGGTTGCGGCGGAAGGAGGGAGAGACAATCGGCGATGGCGGCGTCCGCGAGGCCCGGGGAGAGGGCGAC
CAGCTTCGCCGTCGCGTGCAGCCTCCTCAGCCGCTTCGTCCGCCAGAACGGCGTCGCGGCTGCCGACCTAGGCCTCAGGA
TCAAAGGCGAGGTCGAGCAGCAGAGGACGCCGGCGACAACTAACTCGCTCCCCGGAGCGGAGGGCGAGGAGGTCGAAAGG
AGGAAGGAGACCATGGAGCTGTTCCCGCAGAGCGTCGGGTTCAGCATCAAGGATGCTGCTGCCCCTAGGGAGGAGCAAGG
AGATAAAGAGAAGCCTAAGCAGCTCACAATCTTCTATGGCGGGAAGGTGCTGGTATTTGACGATTTCCCCGCCGACAAGG
CAAAGGATCTGATGCAGCTGGCCAGCAAGGGCAGCCCAGTGGTACAGAACGTTGCTTTGCCTCAACCCTCTGCAGCAGCT
GCTGTCACTACTGACAAGGCCGTGCTGGACCCGGTCATCAGCTTGGCCGCTGCTAAGAAGCCTGCTCGCACAAATGCTTC
TGATATGCCTATTATGAGGAAGGCTTCTCTTCACCGCTTCCTTGAGAAGAGAAAAGATCGCCTCAATGCAAAGACACCAT
ATCAAACTGCTCCTTCAGATGCCGCACCAGTCAAGAAGGAGCCTGAGAGCCAGCCATGGCTCGGATTAGGACCGAATGCC
GTGGATTCCAGCCTGAACCTCAGCTAGTACACAACAACACAGCCATAATAAGACGGCACAGCAACGGCACCCACCATGAC
CTGGAATTTGCTTTGCCACTAGATCGAGTTCAGAAACCATAAATATTCTTAGTTATCGTGTTTTAGTGTTCGCTTATGTC
GTGGTTCTACGCCCTCTTGTCGCATAGTTCTGGATGTAAAGAAAAGTAGCCAGTCATGACCATCACCGGGAGTCGGGGAG
AAGTTTTTGGAGGGTGTTAACACACGGCGACTCTGAATTGGCCATGCTAAGGAAAAGGATAGTGCAATGTGAGGTATATG
TATTTTCTGAAAGCCAGATCGTTTGGGTCTAGCCCTGATGTATGCAGAACAAGTGATTATTGTAATGGATCCTGATTATC
CTCATCATCCTATTGTTGTTTTCTGATCACA
>Zm00001d022139_P001 peptide
MAASARPGERATSFAVACSLLSRFVRQNGVAAADLGLRIKGEVEQQRTPATTNSLPGAEGEEVERRKETMELFPQSVGFS
IKDAAAPREEQGDKEKPKQLTIFYGGKVLVFDDFPADKAKDLMQLASKGSPVVQNVALPQPSAAAAVTTDKAVLDPVISL
AAAKKPARTNASDMPIMRKASLHRFLEKRKDRLNAKTPYQTAPSDAAPVKKEPESQPWLGLGPNAVDSSLNLS

>Zm00001d022139_T002 cDNA
Chr7: 171049765..171051982
ACACGAGCCCCCCGCGTTCACGAGCCAGAAGAGAAGAGAGGCTAACCACCGCAGCGGCTGCGACCTGCGAGTGCGACAA
CCTCCGCGGAGGAGTTGGAGCCGGCGGCGGGGATATTTGCTTGCCCGGCTGGGGTTGCGGCGGAAGGAGGGAGAGACAAT
CGGCGATGGCGGCGTCCGCGAGGCCCGGGGAGAGGGCGACCAGCTTCGCCGTCGCGTGCAGCCTCCTCAGCCGCTTCGTC
CGCCAGAACGGCGTCGCGGCTGCCGACCTAGGCCTCAGGATCAAAGGCGAGGTCGAGCAGCAGAGGACGCCGGCGACAAC
TAACTCGCTCCCCGGAGCGGAGGGCGAGGAGGTCGAAAGGAGGAAGGAGCCTGAGAGCCAGCCATGGCTCGGATTAGGAC
CGAATGCCGTGGATTCCAGCCTGAACCTCAGCTAGTACACAACAACACAGCCATAATAAGACGGCACAGCAACGGCACCC
ACCATGACCTGGAATTTGCTTTGCCACTAGATCGAGTTCAGAAACCATAAATATTCTTAGTTATCGTGTTTTAGTGTTCG
CTTATGTCGTGGTTCTACGCCCTCTTGTCGCATAGTTCTGGATGTAAAGAAAAGTAGCCAGTCATGACCATCACCGGGAG
TCGGGGAGAAGTTTTTGGAGGGTGTTAACACACGGCGACTCTGAATTGGCCATGCTAAGGAAAAGGATAGTGCAATGTGA
GGTATATGTATTTTCTGAAAGCCAGATCGTTTGGGTCTAGCCCTGATGTATGCAGAACAAGTGATTATTGTAATG
>Zm00001d022139_P002 peptide
MAASARPGERATSFAVACSLLSRFVRQNGVAAADLGLRIKGEVEQQRTPATTNSLPGAEGEEVERRKEPESQPWLGLGPN
AVDSSLNLS

ZmJAZ21

B73 RefGen_v3:
GRMZM2G036351 (zim4 - ZIM-transcription factor 4)
Chr9: 150514712..150515694

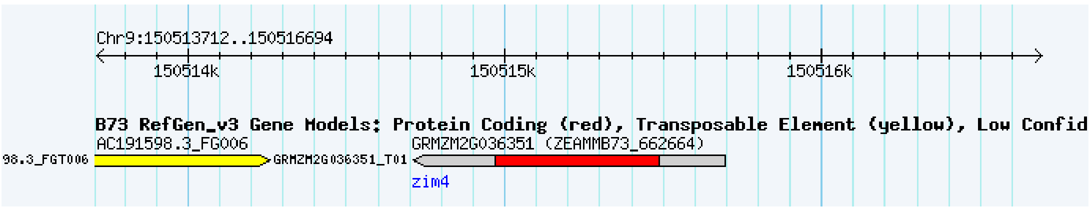

>GRMZM2G036351 Genomic DNA
GTGTCCTCTCCGTTGCCTTGCCTTCCCCTGCCCCGCCCCCATCTCCGATCTCCCCTATAAAACACAATGCTCCCCGCGTC
GCCCCCCGAATCAATTCCCACAGCACCACCAAACCAATAGGCAGGCAGCAAACACAAGCCAATAGGCAACACCCGCGGCG
ACGCAAGAGGGAGAAGCGAGCGTGCAGCGGTCCTCCTCTCGATCCACATGGCCATGGCGGCAGCGGACGGCAAGAGCCGG
AGGTTCGCGCTGGCCTGCGGGGTGCTGAGCCAGTACGTCAAGGCCGAGCAGCAGCAGCAGCAGCAGATGGCGGCCCCGCG
CGCCCCGGCCACCACGACCCTGAGCCTGATGCCCGGCGCGGACGTCGGCGCCGACGAGCAGCAGGAGGCGGCCGCCGCTG
GGGCCGAGGAGATGCCCGGGCCCGCCCCCGCCGCGGCGCCGCCGCTCACCATCTTCTACGGCGGCAGGGTGGTGGTGTTC
GAGGACTTCCCGGCGGAGAAGGCGGCCGAGGTCATGCGCCTCGCGGCCGGCGACGACCTGCCCATCGCCCGGAAGGCGTC
GCTGCAGCGGTTCCTGGCCAAGCGCAAAGATCGCCTCGTCGAGCGCGCGCCCTACGCCCGCCCGTCGTCCCCCGCGGAGG
CGGTCAAGCCGGCCTCGGGCTCGGCCTCGGCCTCCTGGCTCGGGCTCCGCAGCACGGAAGCCGACCGCCTCACCATCGCG
CTGTGATTTACTGGATCGACCTCGACCAGTGGCGATTTAATTGGCTAATGATGGGTTCATGATCATGCCTGCCTTTGGGG
CTGGAATGCTGGATGGAGATGGGAGACAGAGACAGGATTGTAGAGATGTTTTTTTTTCATTTCTTACGAGAGTTTCGCT
GCTGTCGTGAGATCTGATTCGATAATAGAGAGAAGATGAAGATCGAACAATTTGTGCCTTTCGTTGTGCTTGTGCACGTA
TATTCTCCTGCAAAAATCCCTGA
>GRMZM2G036351_T01 cDNA
GTGTCCTCTCCGTTGCCTTGCCTTCCCCTGCCCCGCCCCCATCTCCGATCTCCCCTATAAAACACAATGCTCCCCGCGTC
GCCCCCCGAATCAATTCCCACAGCACCACCAAACCAATAGGCAGGCAGCAAACACAAGCCAATAGGCAACACCCGCGGCG
ACGCAAGAGGGAGAAGCGAGCGTGCAGCGGTCCTCCTCTCGATCCACATGGCCATGGCGGCAGCGGACGGCAAGAGCCGG

AGGTTCGCGCTGGCCTGCGGGGTGCTGAGCCAGTACGTCAAGGCCGAGCAGCAGCAGCAGCAGCAGATGGCGGCCCCGCG
CGCCCCGGCCACCACGACCCTGAGCCTGATGCCCGGCGCGGACGTCGGCGCCGACGAGCAGCAGGAGGCGGCCGCCGCTG
GGGCCGAGGAGATGCCCGGGCCCGCCCCCGCCGCGGCGCCGCCGCTCACCATCTTCTACGGCGGCAGGGTGGTGGTGTTC
GAGGACTTCCCGGCGGAGAAGGCGGCCGAGGTCATGCGCCTCGCGGCCGGCGACGACCTGCCCATCGCCCGGAAGGCGTC
GCTGCAGCGGTTCCTGGCCAAGCGCAAAGATCGCCTCGTCGAGCGCGCGCCCTACGCCCGCCCGTCGTCCCCCGCGGAGG
CGGTCAAGCCGGCCTCGGGCTCGGCCTCGGCCTCCTGGCTCGGGCTCCGCAGCACGGAAGCCGACCGCCTCACCATCGCG
CTGTGATTTACTGGATCGACCTCGACCAGTGGCGATTTAATTGGCTAATGATGGGTTCATGATCATGCCTGCCTTTGGGG
CTGGAATGCTGGATGGAGATGGGAGACAGAGACAGGATTGTAGAGATGTTTTTTTTTCATTTCTTACGAGAGTTTCGCT
GCTGTCGTGAGATCTGATTCGATAATAGAGAGAAGATGAAGATCGAACAATTTGTGCCTTTCGTTGTGCTTGTGCACGTA
TATTCTCCTGCAAAAATCCCTGA
>GRMZM2G036351_P01 peptide
MAMAAADGKSRRFALACGVLSQYVKAEQQQQQQMAAPRAPATTTLSLMPGADVGADEQQEAAAAGAEEMPGPAPAAAPPL
TIFYGGRVVVFEDFPAEKAAEVMRLAAGDDLPIARKASLQRFLAKRKDRLVERAPYARPSSPAEAVKPASGSASASWLGL
RSTEADRLTIAL

AGPv4:
Zm00001d048263 (zim4 - ZIM-transcription factor 4)
Chr9: 153418013..153418531

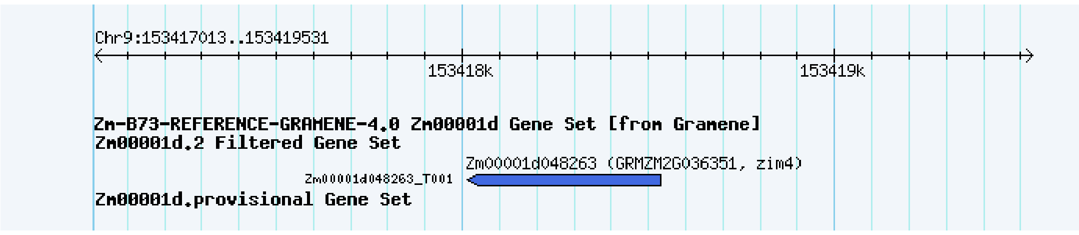

>Zm00001d048263 Genomic DNA
ATGGCCATGGCGGCAGCGGACGGCAAGAGCCGGAGGTTCGCGCTGGCCTGCGGGGTGCTGAGCCAGTACGTCAAGGCCGA
GCAGCAGCAGCAGCAGCAGATGGCGGCCCCGCGCGCCCCGGCCACCACGACCCTGAGCCTGATGCCCGGCGCGGACGTCG
GCGCCGACGAGCAGCAGGAGGCGGCCGCCGCTGGGGCCGAGGAGATGCCCGGGCCCGCCCCCGCCGCGGCGCCGCCGCTC
ACCATCTTCTACGGCGGCAGGGTGGTGGTGTTCGAGGACTTCCCGGCGGAGAAGGCGGCCGAGGTCATGCGCCTCGCGGC
CGGCGACGACCTGCCCATCGCCCGGAAGGCGTCGCTGCAGCGGTTCCTGGCCAAGCGCAAAGATCGCCTCGTCGAGCGCG
CGCCCTACGCCCGCCCGTCGTCCCCCGCGGAGGCGGTCAAGCCGGCCTCGGGCTCGGCCTCGGCCTCCTGGCTCGGGCTC
CGCAGCACGGAAGCCGACCGCCTCACCATCGCGCTGTGA
>Zm00001d048263_T001 cDNA
ATGGCCATGGCGGCAGCGGACGGCAAGAGCCGGAGGTTCGCGCTGGCCTGCGGGGTGCTGAGCCAGTACGTCAAGGCCGA
GCAGCAGCAGCAGCAGCAGATGGCGGCCCCGCGCGCCCCGGCCACCACGACCCTGAGCCTGATGCCCGGCGCGGACGTCG
GCGCCGACGAGCAGCAGGAGGCGGCCGCCGCTGGGGCCGAGGAGATGCCCGGGCCCGCCCCCGCCGCGGCGCCGCCGCTC
ACCATCTTCTACGGCGGCAGGGTGGTGGTGTTCGAGGACTTCCCGGCGGAGAAGGCGGCCGAGGTCATGCGCCTCGCGGC
CGGCGACGACCTGCCCATCGCCCGGAAGGCGTCGCTGCAGCGGTTCCTGGCCAAGCGCAAAGATCGCCTCGTCGAGCGCG
CGCCCTACGCCCGCCCGTCGTCCCCCGCGGAGGCGGTCAAGCCGGCCTCGGGCTCGGCCTCGGCCTCCTGGCTCGGGCTC
CGCAGCACGGAAGCCGACCGCCTCACCATCGCGCTGTGA
>Zm00001d048263_P001 peptide
MAMAAADGKSRRFALACGVLSQYVKAEQQQQQQMAAPRAPATTTLSLMPGADVGADEQQEAAAAGAEEMPGPAPAAAPPL
TIFYGGRVVVFEDFPAEKAAEVMRLAAGDDLPIARKASLQRFLAKRKDRLVERAPYARPSSPAEAVKPASGSASASWLGL
RSTEADRLTIAL

ZmJAZ22

B73 RefGen_v3:

GRMZM2G036288 (zim14 - ZIM-transcription factor 14)

Chr9: 150582432..150583458

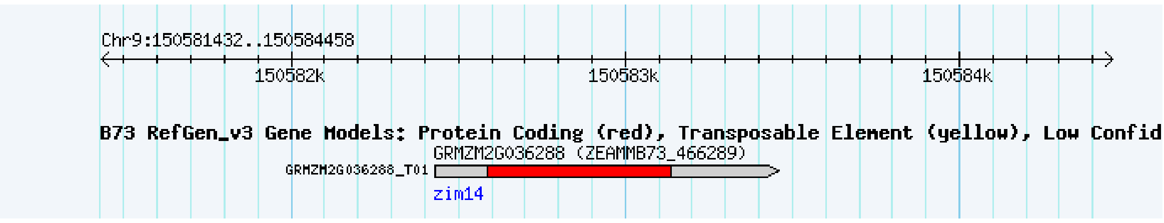

>GRMZM2G036288 Genomic DNA

CCACGGTCCATCCATCGACGACGAGTCACACGAGTCACGGCTCGTGCGTGCGTGCGTACGTACGCAGCCCAGGGACACCG
ACCGCCGCCGCCGCCCGCAGCTCCTCCTCCTCCTCCTCCGACCCCCTCTCAACCGCACGTACACGTCCTCGCTCCATGGC
GGGCATGCCCACGGAAAGCGTGACCCGGCGCTTCGCCGCCGCGTGCGGCGTGCTCAGCCAGTACGTCCGGACGACCGGCG
CGCCCGCGATGACGCCGCCGCCGCCGTTCCTGAAGCCAGCCGCCGCCCAGGAGACGACGGTCGCGCCGCGCACGCAGCAG
CTGACCATCTTCTACGGCGGGAGGGTGGTGGTGCTGGACGCCTGCCCGGCCGACAAGGCGGACGAGCTGATCCGCCTCGC
CGCCTCGGCGGCGGCCGCGCAGGGCCCGCTGCAGCAGCCGCCGGAGGAGCAGGCGCTGGTGGACATGCCCATCGCCCGGA
AGGCCTCGCTGCGGCGCTTCCTCGCCAAGCGCAAGGACCGGTGGTCCTCCGCCAGCTCTACGGCCTATGACGACCGCCGC
CAGGACGACGACGACGACGACGAGGCGGAGGAGGAGGAGGAGCCGCCGGCGCCCAAGAAAGGCAAGATGGCGGCGGCGGC
GCGCGAGGACCCTTCTTCCTCTTCCTGGCTCGCGCTCGGCAGCATGTGCTCCATGCACGGCCGCTGATGAGGGAGCCAGC
GGTGGTTATTAGTAGTGCAGACAAGAGCTCGGGCGCCCGCCCGGCCATGGGATGGGATGGGAGGCGTTGCAGCAGCAATT
TATGTGCTACCTACGTACTAGTATTTATTATAATTGTTATTACTAACAACAACGACTGGTAGTATCGATCAGAGACCGAC
GCTATTCGATCGTTGGTTCGTTTCATTGTCCTTCCTTGCTAGCTGGTGGTGTATCGACCATCTAAATCTAACAGGACACG
TGGACCTGGACGGCAAGAGATGTCCAGTCCAGCAGTGTGGAACGTTTGTTGCTTTGTCTATCATTAA

>GRMZM2G036288_T01 cDNA

CCACGGTCCATCCATCGACGACGAGTCACACGAGTCACGGCTCGTGCGTGCGTGCGTACGTACGCAGCCCAGGGACACCG
ACCGCCGCCGCCGCCCGCAGCTCCTCCTCCTCCTCCTCCGACCCCCTCTCAACCGCACGTACACGTCCTCGCTCCATGGC
GGGCATGCCCACGGAAAGCGTGACCCGGCGCTTCGCCGCCGCGTGCGGCGTGCTCAGCCAGTACGTCCGGACGACCGGCG
CGCCCGCGATGACGCCGCCGCCGCCGTTCCTGAAGCCAGCCGCCGCCCAGGAGACGACGGTCGCGCCGCGCACGCAGCAG
CTGACCATCTTCTACGGCGGGAGGGTGGTGGTGCTGGACGCCTGCCCGGCCGACAAGGCGGACGAGCTGATCCGCCTCGC
CGCCTCGGCGGCGGCCGCGCAGGGCCCGCTGCAGCAGCCGCCGGAGGAGCAGGCGCTGGTGGACATGCCCATCGCCCGGA
AGGCCTCGCTGCGGCGCTTCCTCGCCAAGCGCAAGGACCGGTGGTCCTCCGCCAGCTCTACGGCCTATGACGACCGCCGC
CAGGACGACGACGACGACGACGAGGCGGAGGAGGAGGAGGAGCCGCCGGCGCCCAAGAAAGGCAAGATGGCGGCGGCGGC
GCGCGAGGACCCTTCTTCCTCTTCCTGGCTCGCGCTCGGCAGCATGTGCTCCATGCACGGCCGCTGATGAGGGAGCCAGC
GGTGGTTATTAGTAGTGCAGACAAGAGCTCGGGCGCCCGCCCGGCCATGGGATGGGATGGGAGGCGTTGCAGCAGCAATT
TATGTGCTACCTACGTACTAGTATTTATTATAATTGTTATTACTAACAACAACGACTGGTAGTATCGATCAGAGACCGAC
GCTATTCGATCGTTGGTTCGTTTCATTGTCCTTCCTTGCTAGCTGGTGGTGTATCGACCATCTAAATCTAACAGGACACG
TGGACCTGGACGGCAAGAGATGTCCAGTCCAGCAGTGTGGAACGTTTGTTGCTTTGTCTATCATTAA

>GRMZM2G036288_P01 peptide

MAGMPTESVTRRFAAACGVLSQYVRTTGAPAMTPPPPFLKPAAAQETTVAPRTQQLTIFYGGRVVVLDACPADKADELIR
LAASAAAAQGPLQQPPEEQALVDMPIARKASLRRFLAKRKDRWSSASSTAYDDRRQDDDDDDEAEEEEEPPAPKKGKMAA
AAREDPSSSSWLALGSMCSMHGR

AGPv4:

Zm00001d048268 (zim14 - ZIM-transcription factor 14)

Chr9: 153485703..153486254

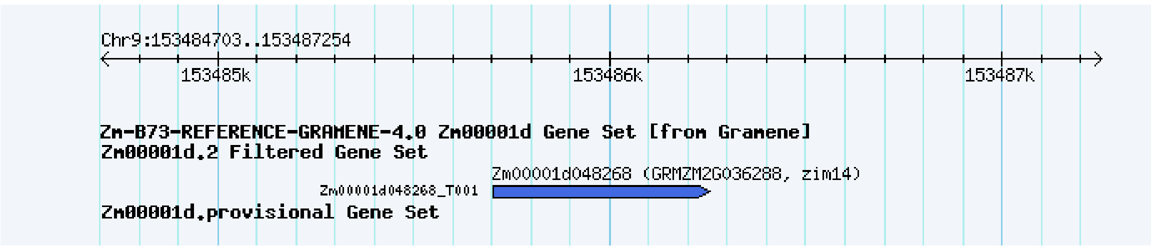

>Zm00001d048268 Genomic DNA

ATGGCGGGCATGCCCACGGAAAGCGTGACCCGGCGCTTCGCCGCCGCGTGCGGCGTGCTCAGCCAGTACGTCCGGACGAC
CGGCGCGCCCGCGATGACGCCGCCGCCGCCGTTCCTGAAGCCAGCCGCCGCCCAGGAGACGACGGTCGCGCCGCGCACGC
AGCAGCTGACCATCTTCTACGGCGGGAGGGTGGTGGTGCTGGACGCCTGCCCGGCCGACAAGGCGGACGAGCTGATCCGC
CTCGCCGCCTCGGCGGCGGCCGCGCAGGGCCCGCTGCAGCAGCCGCCGGAGGAGCAGGCGCTGGTGGACATGCCCATCGC
CCGGAAGGCCTCGCTGCGGCGCTTCCTCGCCAAGCGCAAGGACCGGTGGTCCTCCGCCAGCTCTACGGCCTATGACGACC
GCCGCCAGGACGACGACGACGACGACGAGGCGGAGGAGGAGGAGGAGCCGCCGGCGCCCAAGAAAGGCAAGATGGCGGCG
GCGGCGCGCGAGGACCCTTCTTCCTCTTCCTGGCTCGCGCTCGGCAGCATGTGCTCCATGCACGGCCGCTGA

>Zm00001d048268_T001 cDNA

ATGGCGGGCATGCCCACGGAAAGCGTGACCCGGCGCTTCGCCGCCGCGTGCGGCGTGCTCAGCCAGTACGTCCGGACGAC
CGGCGCGCCCGCGATGACGCCGCCGCCGCCGTTCCTGAAGCCAGCCGCCGCCCAGGAGACGACGGTCGCGCCGCGCACGC
AGCAGCTGACCATCTTCTACGGCGGGAGGGTGGTGGTGCTGGACGCCTGCCCGGCCGACAAGGCGGACGAGCTGATCCGC
CTCGCCGCCTCGGCGGCGGCCGCGCAGGGCCCGCTGCAGCAGCCGCCGGAGGAGCAGGCGCTGGTGGACATGCCCATCGC
CCGGAAGGCCTCGCTGCGGCGCTTCCTCGCCAAGCGCAAGGACCGGTGGTCCTCCGCCAGCTCTACGGCCTATGACGACC
GCCGCCAGGACGACGACGACGACGACGAGGCGGAGGAGGAGGAGGAGCCGCCGGCGCCCAAGAAAGGCAAGATGGCGGCG
GCGGCGCGCGAGGACCCTTCTTCCTCTTCCTGGCTCGCGCTCGGCAGCATGTGCTCCATGCACGGCCGCTGA

>Zm00001d048268_P001 peptide

MAGMPTESVTRRFAAACGVLSQYVRTTGAPAMTPPPPFLKPAAAQETTVAPRTQQLTIFYGGRVVVLDACPADKADELIR
LAASAAAAQGPLQQPPEEQALVDMPIARKASLRRFLAKRKDRWSSASSTAYDDRRQDDDDDDEAEEEEEPPAPKKGKMAA
AAREDPSSSSWLALGSMCSMHGR

ZmJAZ23

B73 RefGen_v3:

GRMZM2G143402 (zim34 - ZIM-transcription factor 34)

145369328..145373351

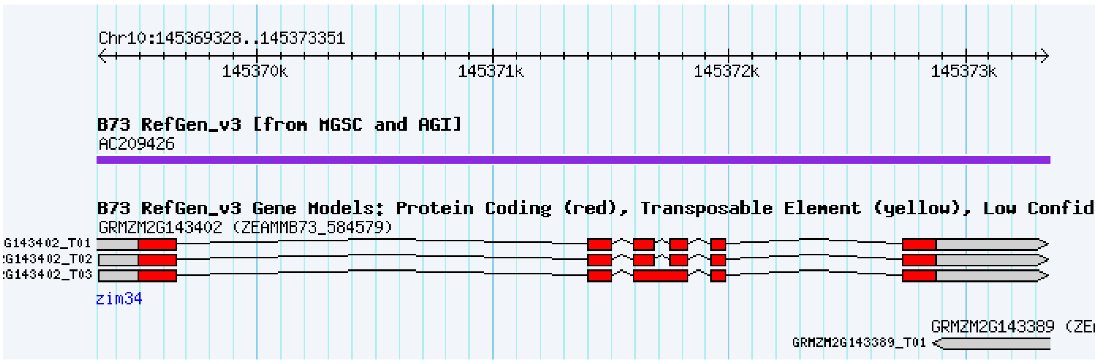

>GRMZM2G143402 Genomic DNA
CTACGACTTTTTCGTCCAGTCCTGCTCACGATATTCACATGCGCCGTTTCCTCTGTACACCCGCGCCGCGCCCCTTTCCC
TTTCTCGCTTCTGCCCCTCTGCGGCTCTGCTGCCTGCGGCCTGCCCCTTTTTTATTCCCAAAAAAACTCCCATCTCGCG
GCGGAAAGAGAGAGAGGAGAGGTGCGAGCTGGTAAGATGGATCGGTTGGAGGAGCGGAACATTAAGAAGGGGGAGAGGGA
GATGGAGGAGGCGCGCAAGGAAGAGGAGCGGAAGGAGGAGGCGGGGGACAGGAATACCCAGGAGCCGCAGCAAGGTCAAG
GCCTCAGCCTCTCACTCGCCAACGGCAGCGACAGCGGCAGGTACGTAGCCTCACCTGACTCTCCGCCCCGCCGTGTCCGG
CGAGGGCTCTGCTTCCCTGGATTGGTTCGCGGCTAATTGCTCCCTAGATTGCTTGGGTGGAATTTGGATTCCTTCGTCTC
CCCCGCGCTTTCCCCCCTTCCTGTTCTCGGTGGTTGACGCGCTTTAGCACTTGCCACCCCGGCTTTTCGCGGTAATTGTG
GAAGCGCCGCGTGGGACGGGCTCTGTGGCCGTACCGTTCGAGTTCGAGGCACCGAATCTCACGCCCACTGCCCAGGGCGA
GGATGCGGAGCCGGCGTGGCGGATCCGGGGACGCGTGCTCGTGCTCTCCTCGCTCGCAGACGGAAAACGGAATCACATCC
GTTGCTTCGGTTCTCTACCGCTCCCGCTTTCCACTCCCGGCTGTGGTGCTGTCACCGTGTAAAGCACCATTGTAAACTAA
CCCCTGTCCCGCGGGGTGCAGATTTAGGTGTACTTTTAGCATCAAATGTTCTTATCTAGAAAGAATTTCAGATGCTGGTC
TGCTTGGAGGGGGTCATCAGCTGCCGGAAATATTCGTTTCCGGTCCTCGGTCATCCTTGCTGTCATTAGCTGTCTTGCGT
TTCTATCTACTCTATTATGATATTGATGTATCATTATGTCGAGCGCGAAACTGAAGCGGAACTCCTGGCTGTCTTAGTGC
CAAATGTCCTTTTGTTGTAGGGCACTCAGCTCAGTTTGTCTAATTTTCTTTGGTGCTAATAAGTAATAAATCTTAAAGGT
CCTTGATCAAACTTAAGTTAAATGGGAGAGACGATTTAGTTTCGTTGTTTCCCATGGTCCCTAGGCTTTGGGAAAGAACT
CTGGCAGATGGCGTGCACATCCAAGCAACGACTCCCCCTCGCTTGCTGAGGTTCTTGTTGTGAAATTTGTTAAAACTTTA
TGAAAGGCTCTAAATTGAATTGCGCAACCAGGTTAACAACTAATAACTGCTACTGTTTGCGATGCAAACCTTGAGTTGGA
GAAATGGCAAACATGCATAGATCTGATGAAAGTTGAGTTTAGCAGGCATATAATTTCCTTGTAGCTGCTATTCAAGATGG
ATAATTTGAACCTCCAGTCCTACCTACAAGGACCACTAGACTGTGTGATAGGGGGCCAAGGTCGCAGAGTCTGAACTGTT
CCTTAAATTTTCCTCTGATGGTAACTGGGTCATTTTTCACAGATTTGGTTGATGTGCTATATTTAGTTGTTCAGTATGTT
TCTTCAGAAGCATTTATTTGTTTTGCCTCGGCAGTGCAGCCAGTGCTAGAACAATAGTGCTATGGTTCTGCTATTTCAGT
TCCATTTGTTTCCTGGTTAGTTGTTATCCTCCAGGCTCTGAACAATAGGGACATCTGAATTAATCAGATTCCAGGAAAAA
TCGTTGTGACTAGATCCGATATCTGTACCACTAAGGCCTACAAATGATTTGAATGCCACTGTCCAGAAAGCCAATCCGTT
CACTGGCCAAGTAGACATGCCTTTCTATCAGAATTTTACCTCCGTTTTTTTAATCATTTTAGGAAGTAGGGCGATTGCTT
TCTACCCCCGTGTCCGCGCACATATTCTATCCTATGCCGGTTTTGAACCCACCACATGGCTTTCTGATATGCTTTTATCT
GAACTAGTAGTGGGAGTGACATGGATCTGCCTACTTTCAATTCAGAGGTGTTGTTTTATCTGCCTTTGTGTGTTCTTATT
TTTTTGCATTCTTTACTACAAGGTCTGGAATGTTGCCGATGTCGAACCCTTCAGCTAATCCCACACAGCTTACAATTTTC
TATGGTGGATCAGTATGTGTGTATGACTCGGTGCCACCAGAAAAGGTAAATATTATCTTCACGTCATTTACAGATATCTT
GTCCTTTTGTACCATCTGTTAATACACATGCTAATACACTAATTTGGCATTGACAGGCTCAGGCAATCATGCTTATAGCT
GCGGCTGCAGCAGCTGCGGCAGCCACCAAAGGCAGTGCTGCCACTGCTTTTAATCCTCCAATGGTACATACAGACACTGT
CGCCCCAGCAGCAGTCTTCTCTCCTGTGCTTACACGGTCTCCATCACTGCAGAGCACTTCTGTAGCAGCTGGACAAGCTC
AGGTTGTTGCTGACCCTAGCTCAATAAGCAAGCTTCAGGCTGGTAAGACCACTGCATCTCTGTGTAAAAATCCAGAAATG
GAATACCTTTTCCAGTGTTGACATTGTCCAACACGTCTTTAAACTATCTTGATCTGTTGGCCAGATCTCCCCATTGCCAG
GAGGCACTCTCTCCAGCGCTTCCTTGAGAAACGTCGTGACAGGTTAGCATGATAGATGATAGCAACAGGGCTCTGAAATT
CTGAATCTTATTGTTAGGCCCCTTATTCTACAATCTACATGTTTAAAACTTTAGTTATAAACTGCTGTATGTGGTACCAC
ACTAATTTTTTCCCCGACTATTGGCACCACAGTAGTCAACAGTATATTTTGTACCGTATTCTGTTTTTTGTCCTCCAGGC
AGGGTAAAAATAAAATAAAATTTGCTGTTATAGCATTTAAATGATTGCACTTTCTGCTGACAAGTGCTCAATGACATCCC
TAAAAAAAATCTATAATTTCTTCACAGAAAGATTGGTAGAGAGCCTGCTTCCATAATACAACATATAGCTGCAAATAGCC
TTGGATCATGTAACTCCCTTGAGATTTTTTTTTAAAAAAAAAATCTGCAAATCGATATTAACACAAGACAGAGTTCTGG
GACCAGCAGTAGATACAAAAACCAACATTACCCCCATTTAGCTGCTCCAAATACCATGTTTTTCCCCTGTTAGGAAAATA
ACATATCTGAGAATCGGGATGTTCCTGTGTATTATCCTGTTCTATTTGGAAAAGCTGGCCACTTAAAGATCCAGTCTCAA
GGTTGCAAACTAGCACCATTTTTTTTTCTGATGTAAGCTTTAGATGCCTGTTTTTGCCTTATCTTAGAACTGAGTTTAGA
ATTGTTTCTTAGTATAACTTTTTTTTTATCGTGGATCTACTGATTTCAAGTGATTACATAAATGATGGCACGAAACAGGG

TCGTGAGCAAAGCTCCGTACAGCCCCGCCAAGTCGTTCGATGGCATGGAGTCAGCGGGAATGGAGATGACTGCGGACGGC
AAGCAGGGCGCAAGGCCCAGTATCTTGAAAGGTTGGGGAGCCGCGAAGTCTGAAGTCTGAACGGTGAAAGGCTGGCTGAC
GCTGTGGGGAGGAAGGGACAGGGACGTGCTGTTGGTTTTATTATATTGGTATTTATGTTATTATATACTGTATGTGGTAG
CAGCAGCGTGGAACCGCACTAATAATGGGGACCTTAACACCTCTATTTTACTGTCGTTGCTTTGTTGTCGCGTGCTAGGT
TGTATTCTGAAGTGGATTCCTCAATACCGCATACCGTACTGCTACGCCTGTTGACTTTGTTACATTGATTGATCGTTGTT
CACTTGTAATGGAATGCCGCACCATGTTTTCTGTCGTGGCATCATACGATTGCCCGCGGCTATCAATCAGCTCGAATGCT
ATCTGTTATTACCCAGAAGACGAACTAACCTGTGGGTACAGTCAACAAATCATCCACAGAGGAGTAAAAAAGAAGCAGTC
CCTTAAAGACACCAAAGGCGAGCAAAACTGCATGAAGAAACAGAAAAAAAAAA

>GRMZM2G143402_T01 cDNA

Chr10: 145369299..145373351

CTACGACTTTTTCGTCCAGTCCTGCTCACGATATTCACATGCGCCGTTTCCTCTGTACACCCGCGCCGCGCCCCTTTCCC
TTTCTCGCTTCTGCCCCTCTGCGGCTCTGCTGCCTGCGGCCTGCCCCTTTTTTTATTCCCAAAAAAACTCCCATCTCGCG
GCGGAAAGAGAGAGAGGAGAGGTGCGAGCTGGTAAGATGGATCGGTTGGAGGAGCGGAACATTAAGAAGGGGGAGAGGGA
GATGGAGGAGGCGCGCAAGGAAGAGGAGCGGAAGGAGGAGGCGGGGGACAGGAATACCCAGGAGCCGCAGCAAGGTCAAG
GCCTCAGCCTCTCACTCGCCAACGGCAGCGACAGCGGCAGGTCTGGAATGTTGCCGATGTCGAACCCTTCAGCTAATCCC
ACACAGCTTACAATTTTCTATGGTGGATCAGTATGTGTGTATGACTCGGTGCCACCAGAAAAGGCTCAGGCAATCATGCT
TATAGCTGCGGCTGCAGCAGCTGCGGCAGCCACCAAAGGCAGTGCTGCCACTGCTTTTAATCCTCCAATGAGCACTTCTG
TAGCAGCTGGACAAGCTCAGGTTGTTGCTGACCCTAGCTCAATAAGCAAGCTTCAGGCTGATCTCCCCATTGCCAGGAGG
CACTCTCTCCAGCGCTTCCTTGAGAAACGTCGTGACAGGGTCGTGAGCAAAGCTCCGTACAGCCCCGCCAAGTCGTTCGA
TGGCATGGAGTCAGCGGGAATGGAGATGACTGCGGACGGCAAGCAGGGCGCAAGGCCCAGTATCTTGAAAGGTTGGGGAG
CCGCGAAGTCTGAAGTCTGAACGGTGAAAGGCTGGCTGACGCTGTGGGGAGGAAGGGACAGGGACGTGCTGTTGGTTTTA
TTATATTGGTATTTATGTTATTATATACTGTATGTGGTAGCAGCAGCGTGGAACCGCACTAATAATGGGGACCTTAACAC
CTCTATTTTACTGTCGTTGCTTTGTTGTCGCGTGCTAGGTTGTATTCTGAAGTGGATTCCTCAATACCGCATACCGTACT
GCTACGCCTGTTGACTTTGTTACATTGATTGATCGTTGTTCACTTAAACAGAAAAAAAAAA

>GRMZM2G143402_P01 peptide

MDRLEERNIKKGEREMEEARKEEERKEEAGDRNTQEPQQGQGLSLSLANGSDSGRSGMLPMSNPSANPTQLTIFYGGSVC
VYDSVPPEKAQAIMLIAAAAAAAAATKGSAATAFNPPMSTSVAAGQAQVVADPSSISKLQADLPIARRHSLQRFLEKRRD
RVVSKAPYSPAKSFDGMESAGMEMTADGKQGARPSILKGWGAAKSEV

>GRMZM2G143402_T02 cDNA

Chr10: 145369328..145373351

GATATTCACATGCGCCGTTTCCTCTGTACACCCGCGCCGCGCCCCTTTCCCTTTCTCGCTTCTGCCCCTCTGCGGCTCTG
CTGCCTGCGGCCTGCCCCTTTTTTTATTCCCAAAAAAACTCCCATCTCGCGGCGGAAAGAGAGAGAGGAGAGGTGCGAGC
TGGTAAGATGGATCGGTTGGAGGAGCGGAACATTAAGAAGGGGGAGAGGGAGATGGAGGAGGCGCGCAAGGAAGAGGAGC
GGAAGGAGGAGGCGGGGGACAGGAATACCCAGGAGCCGCAGCAAGGTCAAGGCCTCAGCCTCTCACTCGCCAACGGCAGC
GACAGCGGCAGGTCTGGAATGTTGCCGATGTCGAACCCTTCAGCTAATCCCACACAGCTTACAATTTTCTATGGTGGATC
AGTATGTGTGTATGACTCGGTGCCACCAGAAAAGGCTCAGGCAATCATGCTTATAGCTGCGGCTGCAGCAGCTGCGGCAG
CCACCAAAGGCAGTGCTGCCACTGCTTTTAATCCTCCAATGAGCACTTCTGTAGCAGCTGGACAAGCTCAGGTTGTTGCT
GACCCTAGCTCAATAAGCAAGCTTCAGGCTGATCTCCCCATTGCCAGGAGGCACTCTCTCCAGCGCTTCCTTGAGAAACG
TCGTGACAGGGTCGTGAGCAAAGCTCCGTACAGCCCCGCCAAGTCGTTCGATGGCATGGAGTCAGCGGGAATGGAGATGA
CTGCGGACGGCAAGCAGGGCGCAAGGCCCAGTATCTTGAAAGGTTGGGGAGCCGCGAAGTCTGAAGTCTGAACGGTGAAA
GGCTGGCTGACGCTGTGGGGAGGAAGGGACAGGGACGTGCTGTTGGTTTTATTATATTGGTATTTATGTTATTATATACT
GTATGTGGTAGCAGCAGCGTGGAACCGCACTAATAATGGGGACCTTAACACCTCTATTTTACTGTCGTTGCTTTGTTGTC
GCGTGCTAGGTTGTATTCTGAAGTGGATTCCTCAATACCGCATACCGTACTGCTACGCCTGTTGACTTTGTTACATTGAT
TGATCGTTGTTCACTTGTAATGGAATGCCGCACCATGTTTTCTGTCGTGGCATCATACGATTGCCCGCGGCTATCAATCA

GCTCGAATGCTATCTGTTATTACCCAGAAGACGAACTAACCTGTGGGTACAGTCAACAAATCATCCACAGAGGAGTAAAA
AAGAAGCAGTCCCTTAAAGACACCAAAGGCGAGCAAAACTGCATGAAGAAACAGAAAAAAAAAA
>GRMZM2G143402_P02 peptide
MDRLEERNIKKGEREMEEARKEEERKEEAGDRNTQEPQQGQGLSLSLANGSDSGRSGMLPMSNPSANPTQLTIFYGGSVC
VYDSVPPEKAQAIMLIAAAAAAAAATKGSAATAFNPPMSTSVAAGQAQVVADPSSISKLQADLPIARRHSLQRFLEKRRD
RVVSKAPYSPAKSFDGMESAGMEMTADGKQGARPSILKGWGAAKSEV
>GRMZM2G143402_T03 cDNA
Chr10: 145369328..145373351
GATATTCACATGCGCCGTTTCCTCTGTACACCCGCGCCGCGCCCCTTTCCCTTTCTCGCTTCTGCCCCTCTGCGGCTCTG
CTGCCTGCGGCCTGCCCCTTTTTTTATTCCCAAAAAAACTCCCATCTCGCGGCGGAAAGAGAGAGAGGAGAGGTGCGAGC
TGGTAAGATGGATCGGTTGGAGGAGCGGAACATTAAGAAGGGGGAGAGGGAGATGGAGGAGGCGCGCAAGGAAGAGGAGC
GGAAGGAGGAGGCGGGGGACAGGAATACCCAGGAGCCGCAGCAAGGTCAAGGCCTCAGCCTCTCACTCGCCAACGGCAGC
GACAGCGGCAGGTCTGGAATGTTGCCGATGTCGAACCCTTCAGCTAATCCCACACAGCTTACAATTTTCTATGGTGGATC
AGTATGTGTGTATGACTCGGTGCCACCAGAAAAGGCTCAGGCAATCATGCTTATAGCTGCGGCTGCAGCAGCTGCGGCAG
CCACCAAAGGCAGTGCTGCCACTGCTTTTAATCCTCCAATGGTACATACAGACACTGTCGCCCCAGCAGCAGTCTTCTCT
CCTGTGCTTACACGGTCTCCATCACTGCAGAGCACTTCTGTAGCAGCTGGACAAGCTCAGGTTGTTGCTGACCCTAGCTC
AATAAGCAAGCTTCAGGCTGATCTCCCCATTGCCAGGAGGCACTCTCTCCAGCGCTTCCTTGAGAAACGTCGTGACAGGG
TCGTGAGCAAAGCTCCGTACAGCCCCGCCAAGTCGTTCGATGGCATGGAGTCAGCGGGAATGGAGATGACTGCGGACGGC
AAGCAGGGCGCAAGGCCCAGTATCTTGAAAGGTTGGGGAGCCGCGAAGTCTGAAGTCTGAACGGTGAAAGGCTGGCTGAC
GCTGTGGGGAGGAAGGGACAGGGACGTGCTGTTGGTTTTATTATATTGGTATTTATGTTATTATATACTGTATGTGGTAG
CAGCAGCGTGGAACCGCACTAATAATGGGGACCTTAACACCTCTATTTTACTGTCGTTGCTTTGTTGTCGCGTGCTAGGT
TGTATTCTGAAGTGGATTCCTCAATACCGCATACCGTACTGCTACGCCTGTTGACTTTGTTACATTGATTGATCGTTGTT
CACTTAAACAGAAAAAAAAAA
>GRMZM2G143402_P03 peptide
MDRLEERNIKKGEREMEEARKEEERKEEAGDRNTQEPQQGQGLSLSLANGSDSGRSGMLPMSNPSANPTQLTIFYGGSVC
VYDSVPPEKAQAIMLIAAAAAAAAATKGSAATAFNPPMVHTDTVAPAAVFSPVLTRSPSLQSTSVAAGQAQVVADPSSIS
KLQADLPIARRHSLQRFLEKRRDRVVSKAPYSPAKSFDGMESAGMEMTADGKQGARPSILKGWGAAKSEV

AGPv4:
Zm00001d026477 (zim34 - ZIM-transcription factor 34)
Chr10: 146705762..146709602

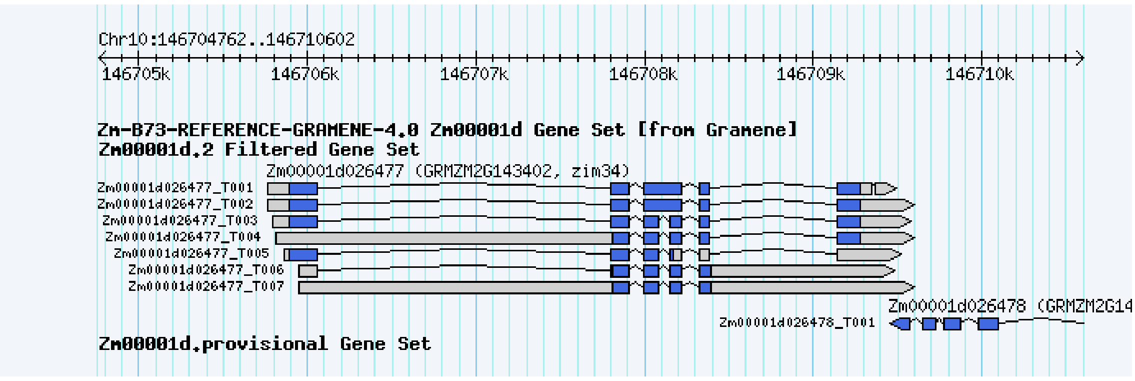

>Zm00001d026477 Genomic DNA
GCGCCGCGCCCCTTTCCCTTTCTCGCTTCTGCCCCTCTGCGGCTCTGCTGCCTGCGGCCTGCCCCTTTTTTTATTCCCAA
AAAAACTCCCATCTCGCGGCGGAAAGAGAGAGAGGAGAGGTGCGAGCTGGTAAGATGGATCGGTTGGAGGAGCGGAACAT
TAAGAAGGGGGAGAGGGAGATGGAGGAGGCGCGCAAGGAAGAGGAGCGGAAGGAGGAGGCGGGGGACAGGAATACCCAGG

AGCCGCAGCAAGGTCAAGGCCTCAGCCTCTCACTCGCCAACGGCAGCGACAGCGGCAGGTACGTAGCCTCACCTGACTCT
CCGCCCCGCCGTGTCCGGCGAGGGCTCTGCTTCCCTGGATTGGTTCGCGGCTAATTGCTCCCTAGATTGCTTGGGTGGAA
TTTGGATTCCTTCGTCTCCCCCGCGCTTTCCCCCCTTCCTGTTCTCGGTGGTTGACGCGCTTTAGCACTTGCCACCCCGG
CTTTTCGCGGTAATTGTGGAAGCGCCGCGTGGGACGGGCTCTGTGGCCGTACCGTTCGAGTTCGAGGCACCGAATCTCAC
GCCCACTGCCCAGGGCGAGGATGCGGAGCCGGCGTGGCGGATCCGGGGACGCGTGCTCGTGCTCTCCTCGCTCGCAGACG
GAAAACGGAATCACATCCGTTGCTTCGGTTCTCTACCGCTCCCGCTTTCCACTCCCGGCTGTGGTGCTGTCACCGTGTAA
AGCACCATTGTAAACTAACCCCTGTCCCGCGGGGTGCAGATTTAGGTGTACTTTTAGCATCAAATGTTCTTATCTAGAAA
GAATTTCAGATGCTGGTCTGCTTGGAGGGGGTCATCAGCTGCCGGAAATATTCGTTTCCGGTCCTCGGTCATCCTTGCTG
TCATTAGCTGTCTTGCGTTTCTATCTACTCTATTATGATATTGATGTATCATTATGTCGAGCGCGAAACTGAAGCGGAAC
TCCTGGCTGTCTTAGTGCCAAATGTCCTTTTGTTGTAGGGCACTCAGCTCAGTTTGTCTAATTTTCTTTGGTGCTAATAA
GTAATAAATCTTAAAGGTCCTTGATCAAACTTAAGTTAAATGGGAGAGACGATTTAGTTTCGTTGTTTCCCATGGTCCCT
AGGCTTTGGGAAAGAACTCTGGCAGATGGCGTGCACATCCAAGCAACGACTCCCCCTCGCTTGCTGAGGTTCTTGTTGTG
AAATTTGTTAAAACTTTATGAAAGGCTCTAAATTGAATTGCGCAACCAGGTTAACAACTAATAACTGCTACTGTTTGCGA
TGCAAACCTTGAGTTGGAGAAATGGCAAACATGCATAGATCTGATGAAAGTTGAGTTTAGCAGGCATATAATTTCCTTGT
AGCTGCTATTCAAGATGGATAATTTGAACCTCCAGTCCTACCTACAAGGACCACTAGACTGTGTGATAGGGGGCCAAGGT
CGCAGAGTCTGAACTGTTCCTTAAATTTTCCTCTGATGGTAACTGGGTCATTTTTCACAGATTTGGTTGATGTGCTATAT
TTAGTTGTTCAGTATGTTTCTTCAGAAGCATTTATTTGTTTTGCCTCGGCAGTGCAGCCAGTGCTAGAACAATAGTGCTA
TGGTTCTGCTATTTCAGTTCCATTTGTTTCCTGGTTAGTTGTTATCCTCCAGGCTCTGAACAATAGGGACATCTGAATTA
ATCAGATTCCAGGAAAAATCGTTGTGACTAGATCCGATATCTGTACCACTAAGGCCTACAAATGATTTGAATGCCACTGT
CCAGAAAGCCAATCCGTTCACTGGCCAAGTAGACATGCCTTTCTATCAGAATTTTACCTCCGTTTTTTTAATCATTTTAG
GAAGTAGGGCGATTGCTTTCTACCCCCGTGTCCGCGCACATATTCTATCCTATGCCGGTTTTGAACCCACCACATGGCTT
TCTGATATGCTTTTATCTGAACTAGTAGTGGGAGTGACATGGATCTGCCTACTTTCAATTCAGAGGTGTTGTTTTATCTG
CCTTTGTGTGTTCTTATTTTTTTGCATTCTTTACTACAAGGTCTGGAATGTTGCCGATGTCGAACCCTTCAGCTAATCCC
ACACAGCTTACAATTTTCTATGGTGGATCAGTATGTGTGTATGACTCGGTGCCACCAGAAAAGGTAAATATTATCTTCAC
GTCATTTACAGATATCTTGTCCTTTTGTACCATCTGTTAATACACATGCTAATACACTAATTTGGCATTGACAGGCTCAG
GCAATCATGCTTATAGCTGCGGCTGCAGCAGCTGCGGCAGCCACCAAAGGCAGTGCTGCCACTGCTTTTAATCCTCCAAT
GGTACATACAGACACTGTCGCCCCAGCAGCAGTCTTCTCTCCTGTGCTTACACGGTCTCCATCACTGCAGAGCACTTCTG
TAGCAGCTGGACAAGCTCAGGTTGTTGCTGACCCTAGCTCAATAAGCAAGCTTCAGGCTGGTAAGACCACTGCATCTCTG
TGTAAAAATCCAGAAATGGAATACCTTTTCCAGTGTTGACATTGTCCAACACGTCTTTAAACTATCTTGATCTGTTGGCC
AGATCTCCCCATTGCCAGGAGGCACTCTCTCCAGCGCTTCCTTGAGAAACGTCGTGACAGGTTAGCATGATAGATGATAG
CAACAGGGCTCTGAAATTCTGAATCTTATTGTTAGGCCCCTTATTCTACAATCTACATGTTTAAAACTTTAGTTATAAAC
TGCTGTATGTGGTACCACACTAATTTTTTCCCCGACTATTGGCACCACAGTAGTCAACAGTATATTTTGTACCGTATTCT
GTTTTTTGTCCTCCAGGCAGGGTAAAAATAAAATAAAATTTGCTGTTATAGCATTTAAATGATTGCACTTTCTGCTGACA
AGTGCTCAATGACATCCCTAAAAAAAATCTATAATTTCTTCACAGAAAGATTGGTAGAGAGCCTGCTTCCATAATACAAC
ATATAGCTGCAAATAGCCTTGGATCATGTAACTCCCTTGAGATTTTTTTTTAAAAAAAAAATCTGCAAATCGATATTAA
CACAAGACAGAGTTCTGGGACCAGCAGTAGATACAAAAACCAACATTACCCCCATTTAGCTGCTCCAAATACCATGTTTT
TCCCCTGTTAGGAAAATAACATATCTGAGAATCGGGATGTTCCTGTGTATTATCCTGTTCTATTTGGAAAAGCTGGCCAC
TTAAAGATCCAGTCTCAAGGTTGCAAACTAGCACCATTTTTTTTTCTGATGTAAGCTTTAGATGCCTGTTTTTGCCTTAT
CTTAGAACTGAGTTTAGAATTGTTTCTTAGTATAACTTTTTTTTTATCGTGGATCTACTGATTTCAAGTGATTACATAAA
TGATGGCACGAAACAGGGTCGTGAGCAAAGCTCCGTACAGCCCCGCCAAGTCGTTCGATGGCATGGAGTCAGCGGGAATG
GAGATGACTGCGGACGGCAAGCAGGGCGCAAGGCCCAGTATCTTGAAAGGTTGGGGAGCCGCGAAGTCTGAAGTCTGAAC
GGTGAAAGGCTGGCTGACGCTGTGGGGAGGAAGGGACAGGGACGTGCTGTTGGTTTTATTATATTGGTATTTATGTTATT
ATATACTGTATGTGGTAGCAGCAGCGTGGAACCGCACTAATAATGGGGACCTTAACACCTCTATTTTACTGTCGTTGCTT
TGTTGTCGCGTGCTAGGTTGTATTCTGAAGTGGATTCCTCAATACCGCATACCGTACTGCTACGCCTGTTGACTTTGTTA

CATTGATTGATCGTTGTTCACTTGTAATGGAATGCCGCACCATGTTTTCTGTCGTGGCATCATACGATTGCCCGCGGCTA
T
>Zm00001d026477_T001 cDNA
GCGCCGCGCCCCTTTCCCTTTCTCGCTTCTGCCCCTCTGCGGCTCTGCTGCCTGCGGCCTGCCCCTTTTTTTATTCCCAA
AAAAACTCCCATCTCGCGGCGGAAAGAGAGAGAGGAGAGGTGCGAGCTGGTAAGATGGATCGGTTGGAGGAGCGGAACAT
TAAGAAGGGGGAGAGGGAGATGGAGGAGGCGCGCAAGGAAGAGGAGCGGAAGGAGGAGGCGGGGGACAGGAATACCCAGG
AGCCGCAGCAAGGTCAAGGCCTCAGCCTCTCACTCGCCAACGGCAGCGACAGCGGCAGGTCTGGAATGTTGCCGATGTCG
AACCCTTCAGCTAATCCCACACAGCTTACAATTTTCTATGGTGGATCAGTATGTGTGTATGACTCGGTGCCACCAGAAAA
GGCTCAGGCAATCATGCTTATAGCTGCGGCTGCAGCAGCTGCGGCAGCCACCAAAGGCAGTGCTGCCACTGCTTTTAATC
CTCCAATGGTACATACAGACACTGTCGCCCCAGCAGCAGTCTTCTCTCCTGTGCTTACACGGTCTCCATCACTGCAGAGC
ACTTCTGTAGCAGCTGGACAAGCTCAGGTTGTTGCTGACCCTAGCTCAATAAGCAAGCTTCAGGCTGATCTCCCCATTGC
CAGGAGGCACTCTCTCCAGCGCTTCCTTGAGAAACGTCGTGACAGGGTCGTGAGCAAAGCTCCGTACAGCCCCGCCAAGT
CGTTCGATGGCATGGAGTCAGCGGGAATGGAGATGACTGCGGACGGCAAGCAGGGCGCAAGGCCCAGTATCTTGAAAGGT
TGGGGAGCCGCGAAGTCTGAAGTCTGAACGGTGAAAGGCTGGCTGACGCTGTGGGGAGGAAGGGACAGGGACGTGCTGTT
GGTTTTATTATATTGTATGTGGTAGCAGCAGCGTGGAACCGCACTAATAATGGGGACCTTAACACCTCTATTTTACTGTC
GTTGCTTTGTTGTCGCGTGCTAGGTTGTATTCTGAAGTGGATTCCTCAATACCGC
>Zm00001d026477_P001 peptide
Chr10: 146705762..146709489
MDRLEERNIKKGEREMEEARKEEERKEEAGDRNTQEPQQGQGLSLSLANGSDSGRSGMLPMSNPSANPTQLTIFYGGSVC
VYDSVPPEKAQAIMLIAAAAAAAAATKGSAATAFNPPMVHTDTVAPAAVFSPVLTRSPSLQSTSVAAGQAQVVADPSSIS
KLQADLPIARRHSLQRFLEKRRDRVVSKAPYSPAKSFDGMESAGMEMTADGKQGARPSILKGWGAAKSEV
>Zm00001d026477_T002 cDNA
Chr10: 146705762..146709600
GCGCCGCGCCCCTTTCCCTTTCTCGCTTCTGCCCCTCTGCGGCTCTGCTGCCTGCGGCCTGCCCCTTTTTTTATTCCCAA
AAAAACTCCCATCTCGCGGCGGAAAGAGAGAGAGGAGAGGTGCGAGCTGGTAAGATGGATCGGTTGGAGGAGCGGAACAT
TAAGAAGGGGGAGAGGGAGATGGAGGAGGCGCGCAAGGAAGAGGAGCGGAAGGAGGAGGCGGGGGACAGGAATACCCAGG
AGCCGCAGCAAGGTCAAGGCCTCAGCCTCTCACTCGCCAACGGCAGCGACAGCGGCAGGTCTGGAATGTTGCCGATGTCG
AACCCTTCAGCTAATCCCACACAGCTTACAATTTTCTATGGTGGATCAGTATGTGTGTATGACTCGGTGCCACCAGAAAA
GGCTCAGGCAATCATGCTTATAGCTGCGGCTGCAGCAGCTGCGGCAGCCACCAAAGGCAGTGCTGCCACTGCTTTTAATC
CTCCAATGGTACATACAGACACTGTCGCCCCAGCAGCAGTCTTCTCTCCTGTGCTTACACGGTCTCCATCACTGCAGAGC
ACTTCTGTAGCAGCTGGACAAGCTCAGGTTGTTGCTGACCCTAGCTCAATAAGCAAGCTTCAGGCTGATCTCCCCATTGC
CAGGAGGCACTCTCTCCAGCGCTTCCTTGAGAAACGTCGTGACAGGGTCGTGAGCAAAGCTCCGTACAGCCCCGCCAAGT
CGTTCGATGGCATGGAGTCAGCGGGAATGGAGATGACTGCGGACGGCAAGCAGGGCGCAAGGCCCAGTATCTTGAAAGGT
TGGGGAGCCGCGAAGTCTGAAGTCTGAACGGTGAAAGGCTGGCTGACGCTGTGGGGAGGAAGGGACAGGGACGTGCTGTT
GGTTTTATTATATTGGTATTTATGTTATTATATACTGTATGTGGTAGCAGCAGCGTGGAACCGCACTAATAATGGGGACC
TTAACACCTCTATTTTACTGTCGTTGCTTTGTTGTCGCGTGCTAGGTTGTATTCTGAAGTGGATTCCTCAATACCGCATA
CCGTACTGCTACGCCTGTTGACTTTGTTACATTGATTGATCGTTGTTCACTTGTAATGGAATGCCGCACCATGTTTTCTG
TCGTGGCATCATACGATTGCCCGCGGCT
>Zm00001d026477_P002 peptide
MDRLEERNIKKGEREMEEARKEEERKEEAGDRNTQEPQQGQGLSLSLANGSDSGRSGMLPMSNPSANPTQLTIFYGGSVC
VYDSVPPEKAQAIMLIAAAAAAAAATKGSAATAFNPPMVHTDTVAPAAVFSPVLTRSPSLQSTSVAAGQAQVVADPSSIS
KLQADLPIARRHSLQRFLEKRRDRVVSKAPYSPAKSFDGMESAGMEMTADGKQGARPSILKGWGAAKSEV
>Zm00001d026477_T003 cDNA
Chr10: 146705792..146709577

GCCCCTCTGCGGCTCTGCTGCCTGCGGCCTGCCCCTTTTTTTATTCCCAAAAAAACTCCCATCTCGCGGCGGAAAGAGAG
AGAGGAGAGGTGCGAGCTGGTAAGATGGATCGGTTGGAGGAGCGGAACATTAAGAAGGGGGAGAGGGAGATGGAGGAGGC
GCGCAAGGAAGAGGAGCGGAAGGAGGAGGCGGGGGACAGGAATACCCAGGAGCCGCAGCAAGGTCAAGGCCTCAGCCTCT
CACTCGCCAACGGCAGCGACAGCGGCAGGTCTGGAATGTTGCCGATGTCGAACCCTTCAGCTAATCCCACACAGCTTACA
ATTTTCTATGGTGGATCAGTATGTGTGTATGACTCGGTGCCACCAGAAAAGGCTCAGGCAATCATGCTTATAGCTGCGGC
TGCAGCAGCTGCGGCAGCCACCAAAGGCAGTGCTGCCACTGCTTTTAATCCTCCAATGAGCACTTCTGTAGCAGCTGGAC
AAGCTCAGGTTGTTGCTGACCCTAGCTCAATAAGCAAGCTTCAGGCTGATCTCCCCATTGCCAGGAGGCACTCTCTCCAG
CGCTTCCTTGAGAAACGTCGTGACAGGGTCGTGAGCAAAGCTCCGTACAGCCCCGCCAAGTCGTTCGATGGCATGGAGTC
AGCGGGAATGGAGATGACTGCGGACGGCAAGCAGGGCGCAAGGCCCAGTATCTTGAAAGGTTGGGGAGCCGCGAAGTCTG
AAGTCTGAACGGTGAAAGGCTGGCTGACGCTGTGGGGAGGAAGGGACAGGGACGTGCTGTTGGTTTTATTATATTGGTAT
TTATGTTATTATATACTGTATGTGGTAGCAGCAGCGTGGAACCGCACTAATAATGGGGACCTTAACACCTCTATTTTACT
GTCGTTGCTTTGTTGTCGCGTGCTAGGTTGTATTCTGAAGTGGATTCCTCAATACCGCATACCGTACTGCTACGCCTGTT
GACTTTGTTACATTGATTGATCGTTGTTCACTTGTAATGGAATGCCGCACCATGTTTTCTGTCGTG
>Zm00001d026477_P003 peptide
MDRLEERNIKKGEREMEEARKEEERKEEAGDRNTQEPQQGQGLSLSLANGSDSGRSGMLPMSNPSANPTQLTIFYGGSVC
VYDSVPPEKAQAIMLIAAAAAAAAATKGSAATAFNPPMSTSVAAGQAQVVADPSSISKLQADLPIARRHSLQRFLEKRRD
RVVSKAPYSPAKSFDGMESAGMEMTADGKQGARPSILKGWGAAKSEV
>Zm00001d026477_T004 cDNA
Chr10: 146705811..146709600
GCCTGCGGCCTGCCCCTTTTTTTATTCCCAAAAAAACTCCCATCTCGCGGCGGAAAGAGAGAGAGGAGAGGTGCGAGCTG
GTAAGATGGATCGGTTGGAGGAGCGGAACATTAAGAAGGGGGAGAGGGAGATGGAGGAGGCGCGCAAGGAAGAGGAGCGG
AAGGAGGAGGCGGGGGACAGGAATACCCAGGAGCCGCAGCAAGGTCAAGGCCTCAGCCTCTCACTCGCCAACGGCAGCGA
CAGCGGCAGGTACGTAGCCTCACCTGACTCTCCGCCCCGCCGTGTCCGGCGAGGGCTCTGCTTCCCTGGATTGGTTCGCG
GCTAATTGCTCCCTAGATTGCTTGGGTGGAATTTGGATTCCTTCGTCTCCCCCGCGCTTTCCCCCCTTCCTGTTCTCGGT
GGTTGACGCGCTTTAGCACTTGCCACCCCGGCTTTTCGCGGTAATTGTGGAAGCGCCGCGTGGGACGGGCTCTGTGGCCG
TACCGTTCGAGTTCGAGGCACCGAATCTCACGCCCACTGCCCAGGGCGAGGATGCGGAGCCGGCGTGGCGGATCCGGGGA
CGCGTGCTCGTGCTCTCCTCGCTCGCAGACGGAAAACGGAATCACATCCGTTGCTTCGGTTCTCTACCGCTCCCGCTTTC
CACTCCCGGCTGTGGTGCTGTCACCGTGTAAAGCACCATTGTAAACTAACCCCTGTCCCGCGGGGTGCAGATTTAGGTGT
ACTTTTAGCATCAAATGTTCTTATCTAGAAAGAATTTCAGATGCTGGTCTGCTTGGAGGGGGTCATCAGCTGCCGGAAAT
ATTCGTTTCCGGTCCTCGGTCATCCTTGCTGTCATTAGCTGTCTTGCGTTTCTATCTACTCTATTATGATATTGATGTAT
CATTATGTCGAGCGCGAAACTGAAGCGGAACTCCTGGCTGTCTTAGTGCCAAATGTCCTTTTGTTGTAGGGCACTCAGCT
CAGTTTGTCTAATTTTCTTTGGTGCTAATAAGTAATAAATCTTAAAGGTCCTTGATCAAACTTAAGTTAAATGGGAGAGA
CGATTTAGTTTCGTTGTTTCCCATGGTCCCTAGGCTTTGGGAAAGAACTCTGGCAGATGGCGTGCACATCCAAGCAACGA
CTCCCCCTCGCTTGCTGAGGTTCTTGTTGTGAAATTTGTTAAAACTTTATGAAAGGCTCTAAATTGAATTGCGCAACCAG
GTTAACAACTAATAACTGCTACTGTTTGCGATGCAAACCTTGAGTTGGAGAAATGGCAAACATGCATAGATCTGATGAAA
GTTGAGTTTAGCAGGCATATAATTTCCTTGTAGCTGCTATTCAAGATGGATAATTTGAACCTCCAGTCCTACCTACAAGG
ACCACTAGACTGTGTGATAGGGGGCCAAGGTCGCAGAGTCTGAACTGTTCCTTAAATTTTCCTCTGATGGTAACTGGGTC
ATTTTTCACAGATTTGGTTGATGTGCTATATTTAGTTGTTCAGTATGTTTCTTCAGAAGCATTTATTTGTTTTGCCTCGG
CAGTGCAGCCAGTGCTAGAACAATAGTGCTATGGTTCTGCTATTTCAGTTCCATTTGTTTCCTGGTTAGTTGTTATCCTC
CAGGCTCTGAACAATAGGGACATCTGAATTAATCAGATTCCAGGAAAAATCGTTGTGACTAGATCCGATATCTGTACCAC
TAAGGCCTACAAATGATTTGAATGCCACTGTCCAGAAAGCCAATCCGTTCACTGGCCAAGTAGACATGCCTTTCTATCAG
AATTTACCTCCGTTTTTTTAATCATTTTAGGAAGTAGGGCGATTGCTTTCTACCCCCGTGTCCGCGCACATATTCTATC
CTATGCCGGTTTTGAACCCACCACATGGCTTTCTGATATGCTTTTATCTGAACTAGTAGTGGGAGTGACATGGATCTGCC
TACTTTCAATTCAGAGGTGTTGTTTTATCTGCCTTTGTGTGTTCTTATTTTTTTGCATTCTTTACTACAAGGTCTGGAAT

GTTGCCGATGTCGAACCCTTCAGCTAATCCCACACAGCTTACAATTTTCTATGGTGGATCAGTATGTGTGTATGACTCGG
TGCCACCAGAAAAGGCTCAGGCAATCATGCTTATAGCTGCGGCTGCAGCAGCTGCGGCAGCCACCAAAGGCAGTGCTGCC
ACTGCTTTTAATCCTCCAATGAGCACTTCTGTAGCAGCTGGACAAGCTCAGGTTGTTGCTGACCCTAGCTCAATAAGCAA
GCTTCAGGCTGATCTCCCCATTGCCAGGAGGCACTCTCTCCAGCGCTTCCTTGAGAAACGTCGTGACAGGGTCGTGAGCA
AAGCTCCGTACAGCCCCGCCAAGTCGTTCGATGGCATGGAGTCAGCGGGAATGGAGATGACTGCGGACGGCAAGCAGGGC
GCAAGGCCCAGTATCTTGAAAGGTTGGGGAGCCGCGAAGTCTGAAGTCTGAACGGTGAAAGGCTGGCTGACGCTGTGGGG
AGGAAGGGACAGGGACGTGCTGTTGGTTTTATTATATTGGTATTTATGTTATTATATACTGTATGTGGTAGCAGCAGCGT
GGAACCGCACTAATAATGGGGACCTTAACACCTCTATTTTACTGTCGTTGCTTTGTTGTCGCGTGCTAGGTTGTATTCTG
AAGTGGATTCCTCAATACCGCATACCGTACTGCTACGCCTGTTGACTTTGTTACATTGATTGATCGTTGTTCACTTGTAA
TGGAATGCCGCACCATGTTTTCTGTCGTGGCATCATACGATTGCCCGCGGCT
>Zm00001d026477_P004 peptide
MLPMSNPSANPTQLTIFYGGSVCVYDSVPPEKAQAIMLIAAAAAAAAATKGSAATAFNPPMSTSVAAGQAQVVADPSSIS
KLQADLPIARRHSLQRFLEKRRDRVVSKAPYSPAKSFDGMESAGMEMTADGKQGARPSILKGWGAAKSEV
>Zm00001d026477_T005 cDNA
Chr10: 146705862..146709520
GGAAAGAGAGAGAGGAGAGGTGCGAGCTGGTAAGATGGATCGGTTGGAGGAGCGGAACATTAAGAAGGGGGAGAGGGAGA
TGGAGGAGGCGCGCAAGGAAGAGGAGCGGAAGGAGGAGGCGGGGGACAGGAATACCCAGGAGCCGCAGCAAGGTCAAGGC
CTCAGCCTCTCACTCGCCAACGGCAGCGACAGCGGCAGGTCTGGAATGTTGCCGATGTCGAACCCTTCAGCTAATCCCAC
ACAGCTTACAATTTTCTATGGTGGATCAGTATGTGTGTATGACTCGGTGCCACCAGAAAAGGCTCAGGCAATCATGCTTA
TAGCTGCGGCTGCAGCAGCTGCGGCAGCCACCAAAGGCAGTGCTGCCACTGCTTTTAATCCTCCAATGTGCAGAGCACTT
CTGTAGCAGCTGGACAAGCTCAGGTTGTTGCTGACCCTAGCTCAATAAGCAAGCTTCAGGCTGATCTCCCCATTGCCAGG
AGGCACTCTCTCCAGCGCTTCCTTGAGAAACGTCGTGACAGGGTCGTGAGCAAAGCTCCGTACAGCCCCGCCAAGTCGTT
CGATGGCATGGAGTCAGCGGGAATGGAGATGACTGCGGACGGCAAGCAGGGCGCAAGGCCCAGTATCTTGAAAGGTTGGG
GAGCCGCGAAGTCTGAAGTCTGAACGGTGAAAGGCTGGCTGACGCTGTGGGGAGGAAGGGACAGGGACGTGCTGTTGGTT
TTATTATATTGGTATTTATGTTATTATATACTGTATGTGGTAGCAGCAGCGTGGAACCGCACTAATAATGGGGACCTTAA
CACCTCTATTTTACTGTCGTTGCTTTGTTGTCGCGTGCTAGGTTGTATTCTGAAGTGGATTCCTCAATACCGCATACCGT
ACTGCTACGCCTGTTGACTTTGTT
>Zm00001d026477_P005 peptide
MDRLEERNIKKGEREMEEARKEEERKEEAGDRNTQEPQQGQGLSLSLANGSDSGRSGMLPMSNPSANPTQLTIFYGGSVC
VYDSVPPEKAQAIMLIAAAAAAAAATKGSAATAFNPPMCRALL
>Zm00001d026477_T006 cDNA
Chr10: 146705952..146709480
GCGCAAGGAAGAGGAGCGGAAGGAGGAGGCGGGGGACAGGAATACCCAGGAGCCGCAGCAAGGTCAAGGCCTCAGCCTCT
CACTCGCCAACGGCAGCGACAGCGGCAGGTCTGGAATGTTGCCGATGTCGAACCCTTCAGCTAATCCCACACAGCTTACA
ATTTTCTATGGTGGATCAGTATGTGTGTATGACTCGGTGCCACCAGAAAAGGCTCAGGCAATCATGCTTATAGCTGCGGC
TGCAGCAGCTGCGGCAGCCACCAAAGGCAGTGCTGCCACTGCTTTTAATCCTCCAATGAGCACTTCTGTAGCAGCTGGAC
AAGCTCAGGTTGTTGCTGACCCTAGCTCAATAAGCAAGCTTCAGGCTGATCTCCCCATTGCCAGGAGGCACTCTCTCCAG
CGCTTCCTTGAGAAACGTCGTGACAGGTTAGCATGATAGATGATAGCAACAGGGCTCTGAAATTCTGAATCTTATTGTTA
GGCCCCTTATTCTACAATCTACATGTTTAAAACTTTAGTTATAAACTGCTGTATGTGGTACCACACTAATTTTTTCCCCG
ACTATTGGCACCACAGTAGTCAACAGTATATTTTGTACCGTATTCTGTTTTTTGTCCTCCAGGCAGGGTAAAAATAAAAT
AAAATTTGCTGTTATAGCATTTAAATGATTGCACTTTCTGCTGACAAGTGCTCAATGACATCCCTAAAAAAAATCTATAA
TTTCTTCACAGAAAGATTGGTAGAGAGCCTGCTTCCATAATACAACATATAGCTGCAAATAGCCTTGGATCATGTAACTC
CCTTGAGATTTTTTTTTAAAAAAAAAATCTGCAAATCGATATTAACACAAGACAGAGTTCTGGGACCAGCAGTAGATAC
AAAAACCAACATTACCCCCATTTAGCTGCTCCAAATACCATGTTTTTCCCCTGTTAGGAAAATAACATATCTGAGAATCG

GGATGTTCCTGTGTATTATCCTGTTCTATTTGGAAAAGCTGGCCACTTAAAGATCCAGTCTCAAGGTTGCAAACTAGCAC
CATTTTTTTTTCTGATGTAAGCTTTAGATGCCTGTTTTTGCCTTATCTTAGAACTGAGTTTAGAATTGTTTCTTAGTATA
ACTTTTTTTTTATCGTGGATCTACTGATTTCAAGTGATTACATAAATGATGGCACGAAACAGGGTCGTGAGCAAAGCTCC
GTACAGCCCCGCCAAGTCGTTCGATGGCATGGAGTCAGCGGGAATGGAGATGACTGCGGACGGCAAGCAGGGCGCAAGGC
CCAGTATCTTGAAAGGTTGGGGAGCCGCGAAGTCTGAAGTCTGAACGGTGAAAGGCTGGCTGACGCTGTGGGGAGGAAGG
GACAGGGACGTGCTGTTGGTTTTATTATATTGGTATTTATGTTATTATATACTGTATGTGGTAGCAGCAGCGTGGAACCG
CACTAATAATGGGGACCTTAACACCTCTATTTTACTGTCGTTGCTTTGTTGTCGCGTGCTAGGTTGTATTCTGAAGTGGA
TTCCT
>Zm00001d026477_P006 peptide
MLPMSNPSANPTQLTIFYGGSVCVYDSVPPEKAQAIMLIAAAAAAAAATKGSAATAFNPPMSTSVAAGQAQVVADPSSIS
KLQADLPIARRHSLQRFLEKRRDRLA
>Zm00001d026477_T007 cDNA
Chr10: 146705954..146709602
GCAAGGAAGAGGAGCGGAAGGAGGAGGCGGGGGACAGGAATACCCAGGAGCCGCAGCAAGGTCAAGGCCTCAGCCTCTCA
CTCGCCAACGGCAGCGACAGCGGCAGGTACGTAGCCTCACCTGACTCTCCGCCCCGCCGTGTCCGGCGAGGGCTCTGCTT
CCCTGGATTGGTTCGCGGCTAATTGCTCCCTAGATTGCTTGGGTGGAATTTGGATTCCTTCGTCTCCCCCGCGCTTTCCC
CCCTTCCTGTTCTCGGTGGTTGACGCGCTTTAGCACTTGCCACCCCGGCTTTTCGCGGTAATTGTGGAAGCGCCGCGTGG
GACGGGCTCTGTGGCCGTACCGTTCGAGTTCGAGGCACCGAATCTCACGCCCACTGCCCAGGGCGAGGATGCGGAGCCGG
CGTGGCGGATCCGGGGACGCGTGCTCGTGCTCTCCTCGCTCGCAGACGGAAAACGGAATCACATCCGTTGCTTCGGTTCT
CTACCGCTCCCGCTTTCCACTCCCGGCTGTGGTGCTGTCACCGTGTAAAGCACCATTGTAAACTAACCCCTGTCCCGCGG
GGTGCAGATTTAGGTGTACTTTTAGCATCAAATGTTCTTATCTAGAAAGAATTTCAGATGCTGGTCTGCTTGGAGGGGGT
CATCAGCTGCCGGAAATATTCGTTTCCGGTCCTCGGTCATCCTTGCTGTCATTAGCTGTCTTGCGTTTCTATCTACTCTA
TTATGATATTGATGTATCATTATGTCGAGCGCGAAACTGAAGCGGAACTCCTGGCTGTCTTAGTGCCAAATGTCCTTTTG
TTGTAGGGCACTCAGCTCAGTTTGTCTAATTTTCTTTGGTGCTAATAAGTAATAAATCTTAAAGGTCCTTGATCAAACTT
AAGTTAAATGGGAGAGACGATTTAGTTTCGTTGTTTCCCATGGTCCCTAGGCTTTGGGAAAGAACTCTGGCAGATGGCGT
GCACATCCAAGCAACGACTCCCCCTCGCTTGCTGAGGTTCTTGTTGTGAAATTTGTTAAAACTTTATGAAAGGCTCTAAA
TTGAATTGCGCAACCAGGTTAACAACTAATAACTGCTACTGTTTGCGATGCAAACCTTGAGTTGGAGAAATGGCAAACAT
GCATAGATCTGATGAAAGTTGAGTTTAGCAGGCATATAATTTCCTTGTAGCTGCTATTCAAGATGGATAATTTGAACCTC
CAGTCCTACCTACAAGGACCACTAGACTGTGTGATAGGGGGCCAAGGTCGCAGAGTCTGAACTGTTCCTTAAATTTTCCT
CTGATGGTAACTGGGTCATTTTTCACAGATTTGGTTGATGTGCTATATTTAGTTGTTCAGTATGTTTCTTCAGAAGCATT
TATTTGTTTTGCCTCGGCAGTGCAGCCAGTGCTAGAACAATAGTGCTATGGTTCTGCTATTTCAGTTCCATTTGTTTCCT
GGTTAGTTGTTATCCTCCAGGCTCTGAACAATAGGGACATCTGAATTAATCAGATTCCAGGAAAAATCGTTGTGACTAGA
TCCGATATCTGTACCACTAAGGCCTACAAATGATTTGAATGCCACTGTCCAGAAAGCCAATCCGTTCACTGGCCAAGTAG
ACATGCCTTTCTATCAGAATTTTACCTCCGTTTTTTTAATCATTTTAGGAAGTAGGGCGATTGCTTTCTACCCCCGTGTC
CGCGCACATATTCTATCCTATGCCGGTTTTGAACCCACCACATGGCTTTCTGATATGCTTTTATCTGAACTAGTAGTGGG
AGTGACATGGATCTGCCTACTTTCAATTCAGAGGTGTTGTTTTATCTGCCTTTGTGTGTTCTTATTTTTTTGCATTCTTT
ACTACAAGGTCTGGAATGTTGCCGATGTCGAACCCTTCAGCTAATCCCACACAGCTTACAATTTTCTATGGTGGATCAGT
ATGTGTGTATGACTCGGTGCCACCAGAAAAGGCTCAGGCAATCATGCTTATAGCTGCGGCTGCAGCAGCTGCGGCAGCCA
CCAAAGGCAGTGCTGCCACTGCTTTTAATCCTCCAATGAGCACTTCTGTAGCAGCTGGACAAGCTCAGGTTGTTGCTGAC
CCTAGCTCAATAAGCAAGCTTCAGGCTGATCTCCCCATTGCCAGGAGGCACTCTCTCCAGCGCTTCCTTGAGAAACGTCG
TGACAGGTTAGCATGATAGATGATAGCAACAGGGCTCTGAAATTCTGAATCTTATTGTTAGGCCCCTTATTCTACAATCT
ACATGTTTAAAACTTTAGTTATAAACTGCTGTATGTGGTACCACACTAATTTTTTCCCCGACTATTGGCACCACAGTAGT
CAACAGTATATTTTGTACCGTATTCTGTTTTTTGTCCTCCAGGCAGGGTAAAAATAAAATAAAATTTGCTGTTATAGCAT
TTAAATGATTGCACTTTCTGCTGACAAGTGCTCAATGACATCCCTAAAAAAAATCTATAATTTCTTCACAGAAAGATTGG

TAGAGAGCCTGCTTCCATAATACAACATATAGCTGCAAATAGCCTTGGATCATGTAACTCCCTTGAGATTTTTTTTTAA
AAAAAAAATCTGCAAATCGATATTAACACAAGACAGAGTTCTGGGACCAGCAGTAGATACAAAAACCAACATTACCCCCA
TTTAGCTGCTCCAAATACCATGTTTTTCCCCTGTTAGGAAAATAACATATCTGAGAATCGGGATGTTCCTGTGTATTATC
CTGTTCTATTTGGAAAAGCTGGCCACTTAAAGATCCAGTCTCAAGGTTGCAAACTAGCACCATTTTTTTTTCTGATGTAA
GCTTTAGATGCCTGTTTTTGCCTTATCTTAGAACTGAGTTTAGAATTGTTTCTTAGTATAACTTTTTTTTTATCGTGGAT
CTACTGATTTCAAGTGATTACATAAATGATGGCACGAAACAGGGTCGTGAGCAAAGCTCCGTACAGCCCCGCCAAGTCGT
TCGATGGCATGGAGTCAGCGGGAATGGAGATGACTGCGGACGGCAAGCAGGGCGCAAGGCCCAGTATCTTGAAAGGTTGG
GGAGCCGCGAAGTCTGAAGTCTGAACGGTGAAAGGCTGGCTGACGCTGTGGGGAGGAAGGGACAGGGACGTGCTGTTGGT
TTTATTATATTGGTATTTATGTTATTATATACTGTATGTGGTAGCAGCAGCGTGGAACCGCACTAATAATGGGGACCTTA
ACACCTCTATTTTACTGTCGTTGCTTTGTTGTCGCGTGCTAGGTTGTATTCTGAAGTGGATTCCTCAATACCGCATACCG
TACTGCTACGCCTGTTGACTTTGTTACATTGATTGATCGTTGTTCACTTGTAATGGAATGCCGCACCATGTTTTCTGTCG
TGGCATCATACGATTGCCCGCGGCTAT
>Zm00001d026477_P007 peptide
MLPMSNPSANPTQLTIFYGGSVCVYDSVPPEKAQAIMLIAAAAAAAAATKGSAATAFNPPMSTSVAAGQAQVVADPSSIS
KLQADLPIARRHSLQRFLEKRRDRLA

ZmJAZ24

B73 RefGen_v3:
GRMZM2G054689 (zim5 - ZIM-transcription factor 5)
Chr8: 64176829..64179518

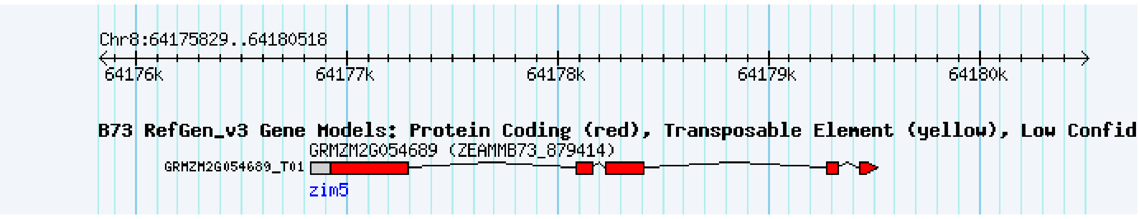

>GRMZM2G054689 Genomic DNA
GCACGCTAGCAACCGCTCCCTTCCTCCACCTCCACCTCCGGAGCGGACGTCGGCGCCGACGAGCAGCAGGAGCCGGCCGC
CGTGAGGGCTGAGGAGATGCCCGGGCCCGCCCCCGCCGTGGCGCCGCCGCTCACCATCTTCTACGGCGGCATGGTGGTGG
TGTTCGAGGACTTCCCGGCGGAGAAGGCGGCCGAGGTCATGTGCCTCGCGGCCGGCGACGACCTACCCATCGCCCGCAAG
GCGTCGCTGCAGCGGTTCCTGGCCAAGCGCAAGGATCGCCTCGTCGAGCGCGCGCCCTACGCCCGCCCATCATCCCCCGC
GGAGGAGCCGGAGAAGAAGACGGTAAAGCCGGCCTCGGCCTTGGCCTCCTGGCTCGGACTCGACAGCACGGAAGCCGACT
GCCTCACCATCACGCTGTTCTGCCATCGACTGGTTCTGCGGTTCTGCCCACCCCCGACAGGTGGGCCCCATCTTCGAGCG
TCTGTCTCCTGCTGTAGCTCGCCCGCAGAACCGCGCACTCCGCTCCACTCCCAAGATTTTCACCGTGCTCGCTACCTGGT
CTGCCCCGCCGTCTTCCCCCAGATCGTGCAAGACCTAGGTCTACCCAGTCGCTGCTCGCCCGCTCCCCCCCTCAACCTCC
AAGCCCAGGTAACCTCCGCCTCACGCTCTCTAGCTCCGCCTCATCCACTGCCCCGTCATTGCCGACGGTGCCGCCAGATG
CCGCAAGGAAGGGGGGGGATGCGGATGGGGACGATCTATGCCGTCGCAGCACTGGCGCTCGACGTGGCGGTGGACTGCGA
GGACAACCTCATCGCCGCAAAAGGGTCACCTCTGGCACTTCGGCATCGCCTCGGGTGAGGGGCCGACAGGCATGTTCTCC
TCCATCGACCCCGTCCATATCTTGTAGGCCATCGAAGTCGACGCCAAAGCGGGGAAGGAGAGCATGAGGGCACACTCTTC
ATTCTCTTAAATCTCACCGTGCCTCACCCTGCGCCAGCCATAGCGCCGAGGGCGGGATCAGTTCCGTAGACAGCTCCCAT
ATCTTCGAAGTCCTAGACCTGGTGGGTCACACGTTTGGGCCATGAGAGGCAGAGATTTATTATGGCTAGGTTGTCTTCCA
TTCCCATCCCACCCTTTCTCGTGCTTGCTGCTCCCTCTCCTATGGCTGATGTGCTCTCCACACTCCTCGAATGGTGCCCG
TCTGGGACTGATTGATGATGTGATGTTGGTTGACCTGACTCCTGTGTCGCATGGGCAGTACGCGGCTATGGGCCTGGTGG

TGTGCGCCGCCTTGGGGAGCGGCTGGACGACGGGGTCAGGAATGGAGGGGCCACCGACGGTGAGCAGAGTCGGTGGGACA
GGCAGACAAGAGGTGTTGACACTGCCTTGGTCCCTCTTTAGTCTTCACAAAATCACCACGGTGGCACATGCGGGTGGTGT
TGACACTACCTTGGTCCCTCTTGACGAAATCACCACGGTGGCACATGCGGGTGGCCTTTGCCTGCAGCGTGTGCGGGCAG
TGTACGACCAGGGCCATCAATCCTCATGCCTACACCAATGAGACGGTGTTTGTTCAGGTAAGATTGTAAGAATTTTGTGA
TTAGTGTATCCATTTTGCATTAATAAGGGTCAAGTTAAGCTGATCCTTCTACTGCCTTTAGGTATATGTATTGAGATGCC
ACCGTTATGCTTGTGTGTTCGTTTTTCTAAGTTGCAGCATCCTTAATTTGTTATTACTCCTTTATAGCATACTATTACAA
TCTTACCTGAACAAACACAGTTCCATCGATGTAGGCATGAGGATTTATGGCCCTGGTCAAGTTAAGCTGATCCTTGTATT
GCCTTTAAACCGCTATGCTTCTGTCTTCGTTTTTCTAAGTTGCAGCATCGTTAATTTGTTATTACTCCTTTATAGCATAC
AATTTAGGTAGGCTTCTGGACGTTGGGAAGAAATGACCAATTCAGTCAATGATTAGTATAGTATCACTATGGGCTACGTG
TTCTCCCTCAAAATCAGAGAGCAGTACAATTCAAATATGAAGGGCTTGGCTTGATAGTTGGTACTTAATATCATGACAAA
AGAGAAGTCACGTTGCTATATTCTGTTGCATTTCTTCTGAATATTGAAATACATTACTAACACCATCGATAATTAGTACA
ATTTCACCATGGGCTATATGTTCTTCCACAAAATTGGGTATATGCATGGTTCGAATTTTAAGAACATGGTTATTCTAGAG
ATGAGAATCTCTTGATACTTTATACTTGATATCATGAGAAGGGAGACATCATCCTGTAATAACTAACGCAATTCTTCTGA
ATATTTGAAAAAATGACTAGCACACATTGCTGGTCATCTCGTTTCTTCTTCATGCTTCATACTCTGTGCTGAGATATGTA
GAAAATACTGAGTGTTTTGCCTTGTATTCATGTATTGTCTCCTAGGGTGTTCAGAAGCACGAAGAGGCTAGTAAAGTCAA
GGACGACAGTGTTCTCAGGTGTGTTTCTTACGCTTACTGTACAATTGATTACTAGATGGACCTTATGCCACATAATATGC
TAATTTGAGTTGTTATTGTTTCTAAAGATTTTGCGCGTGTAGGTATCAATTGATACTTCTCATTTTCCTAAATATAAGTG
GAAAATTTTACACATGTAGCTGCCAGTTGATACTCGTCTACCTTACATAA
>GRMZM2G054689_T01 cDNA
GCACGCTAGCAACCGCTCCCTTCCTCCACCTCCACCTCCGGAGCGGACGTCGGCGCCGACGAGCAGCAGGAGCCGGCCGC
CGTGAGGGCTGAGGAGATGCCCGGGCCCGCCCCCGCCGTGGCGCCGCCGCTCACCATCTTCTACGGCGGCATGGTGGTGG
TGTTCGAGGACTTCCCGGCGGAGAAGGCGGCCGAGGTCATGTGCCTCGCGGCCGGCGACGACCTACCCATCGCCCGCAAG
GCGTCGCTGCAGCGGTTCCTGGCCAAGCGCAAGGATCGCCTCGTCGAGCGCGCGCCCTACGCCCGCCCATCATCCCCCGC
GGAGGAGCCGGAGAAGAAGACGGTAAAGCCGGCCTCGGCCTTGGCCTCCTGGCTCGGACTCGACAGCACGGAAGCCGACT
GCCTCACCATCACGCTGTTCTGCCATCGACTGGTTCTGCGGTTCTGCCCACCCCCGACAGTACGCGGCTATGGGCCTGGT
GGTGTGCGCCGCCTTGGGGAGCGGCTGGACGACGGGGTCAGGAATGGAGGGGCCACCGACGTCTTCACAAAATCACCACG
GTGGCACATGCGGGTGGTGTTGACACTACCTTGGTCCCTCTTGACGAAATCACCACGGTGGCACATGCGGGTGGCCTTTG
CCTGCAGCGTGTGCGGGCAGTGTACGACCAGGGCCATCAATCCTCATGCCTACACCAATGAGACGGTGTTTGTTCAGGGT
GTTCAGAAGCACGAAGAGGCTAGTAAAGTCAAGGACGACAGTGTTCTCAGGTATCAATTGATACTTCTCATTTTCCTAAA
TATAAGTGGAAAATTTTACACATGTAGCTGCCAGTTGATACTCGTCTACCTTACATAA
>GRMZM2G054689_P01 peptide
MPGPAPAVAPPLTIFYGGMVVVFEDFPAEKAAEVMCLAAGDDLPIARKASLQRFLAKRKDRLVERAPYARPSSPAEEPEK
KTVKPASALASWLGLDSTEADCLTITLFCHRLVLRFCPPPTVRGYGPGGVRRLGERLDDGVRNGGATDVFTKSPRWHMRV
VLTLPWSLLTKSPRWHMRVAFACSVCGQCTTRAINPHAYTNETVFVQGVQKHEEASKVKDDSVLRYQLILLIFLNISGKF
YTCSCQLILVYLT

AGPv4:
Zm00001d009438 (zim5 - ZIM-transcription factor 5)
Chr8: 64583138..64584442

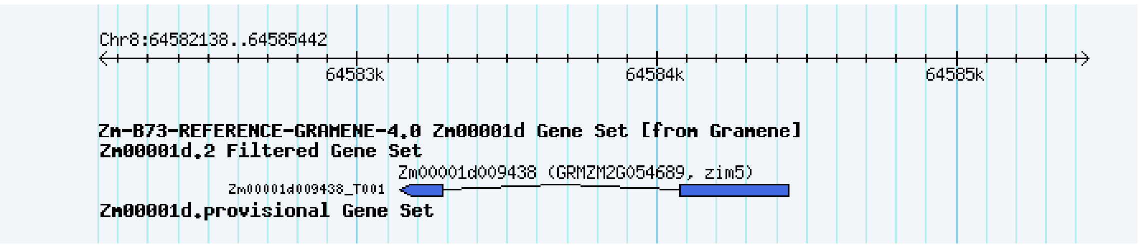

>Zm00001d009438 Genomic DNA
ATGCCCGGGCCCGCCCCCGCCGTGGCGCCGCCGCTCACCATCTTCTACGGCGGCATGGTGGTGGTGTTCGAGGACTTCCC
GGCGGAGAAGGCGGCCGAGGTCATGTGCCTCGCGGCCGGCGACGACCTACCCATCGCCCGCAAGGCGTCGCTGCAGCGGT
TCCTGGCCAAGCGCAAGGATCGCCTCGTCGAGCGCGCGCCCTACGCCCGCCCATCATCCCCCGCGGAGGAGCCGGAGAAG
AAGACGGTAAAGCCGGCCTCGGCCTTGGCCTCCTGGCTCGGACTCGACAGCACGGAAGCCGACTGCCTCACCATCACGCT
GTTCTGCCATCGACTGGTTCTGCGGTTCTGCCCACCCCCGACAGGTGGGCCCCATCTTCGAGCGTCTGTCTCCTGCTGTA
GCTCGCCCGCAGAACCGCGCACTCCGCTCCACTCCCAAGATTTTCACCGTGCTCGCTACCTGGTCTGCCCCGCCGTCTTC
CCCCAGATCGTGCAAGACCTAGGTCTACCCAGTCGCTGCTCGCCCGCTCCCCCCCTCAACCTCCAAGCCCAGGTAACCTC
CGCCTCACGCTCTCTAGCTCCGCCTCATCCACTGCCCCGTCATTGCCGACGGTGCCGCCAGATGCCGCAAGGAAGGGGGG
GGATGCGGATGGGGACGATCTATGCCGTCGCAGCACTGGCGCTCGACGTGGCGGTGGACTGCGAGGACAACCTCATCGCC
GCAAAAGGGTCACCTCTGGCACTTCGGCATCGCCTCGGGTGAGGGGCCGACAGGCATGTTCTCCTCCATCGACCCCGTCC
ATATCTTGTAGGCCATCGAAGTCGACGCCAAAGCGGGGAAGGAGAGCATGAGGGCACACTCTTCATTCTCTTAAATCTCA
CCGTGCCTCACCCTGCGCCAGCCATAGCGCCGAGGGCGGGATCAGTTCCGTAGACAGCTCCCATATCTTCGAAGTCCTAG
ACCTGGTGGGTCACACGTTTGGGCCATGAGAGGCAGAGATTTATTATGGCTAGGTTGTCTTCCATTCCCATCCCACCCTT
TCTCGTGCTTGCTGCTCCCTCTCCTATGGCTGATGTGCTCTCCACACTCCTCGAATGGTGCCCGTCTGGGACTGATTGAT
GATGTGATGTTGGTTGACCTGACTCCTGTGTCGCATGGGCAGTACGCGGCTATGGGCCTGGTGGTGTGCGCCGCCTTGGG
GAGCGGCTGGACGACGGGGTCAGGAATGGAGGGGCCACCGACGGTGAGCAGAGTCGGTGGGACAGGCAGACAAGAGGTGT
TGACACTGCCTTGGTCCCTCTTTAG
>Zm00001d009438_T001 cDNA
ATGCCCGGGCCCGCCCCCGCCGTGGCGCCGCCGCTCACCATCTTCTACGGCGGCATGGTGGTGGTGTTCGAGGACTTCCC
GGCGGAGAAGGCGGCCGAGGTCATGTGCCTCGCGGCCGGCGACGACCTACCCATCGCCCGCAAGGCGTCGCTGCAGCGGT
TCCTGGCCAAGCGCAAGGATCGCCTCGTCGAGCGCGCGCCCTACGCCCGCCCATCATCCCCCGCGGAGGAGCCGGAGAAG
AAGACGGTAAAGCCGGCCTCGGCCTTGGCCTCCTGGCTCGGACTCGACAGCACGGAAGCCGACTGCCTCACCATCACGCT
GTTCTGCCATCGACTGGTTCTGCGGTTCTGCCCACCCCCGACAGTACGCGGCTATGGGCCTGGTGGTGTGCGCCGCCTTG
GGAGCGGCTGGACGACGGGGTCAGGAATGGAGGGGCCACCGACGGTGAGCAGAGTCGGTGGGACAGGCAGACAAGAGGT
GTTGACACTGCCTTGGTCCCTCTTTAG
>Zm00001d009438_P001 peptide
MPGPAPAVAPPLTIFYGGMVVVFEDFPAEKAAEVMCLAAGDDLPIARKASLQRFLAKRKDRLVERAPYARPSSPAEEPEK
KTVKPASALASWLGLDSTEADCLTITLFCHRLVLRFCPPPTVRGYGPGGVRRLGERLDDGVRNGGATDGEQSRWDRQTRG
VDTALVPL

ZmJAZ25

B73 RefGen_v3:
GRMZM2G063632 (zim7 - ZIM-transcription factor 7)
Chr5: 22279974..22282078

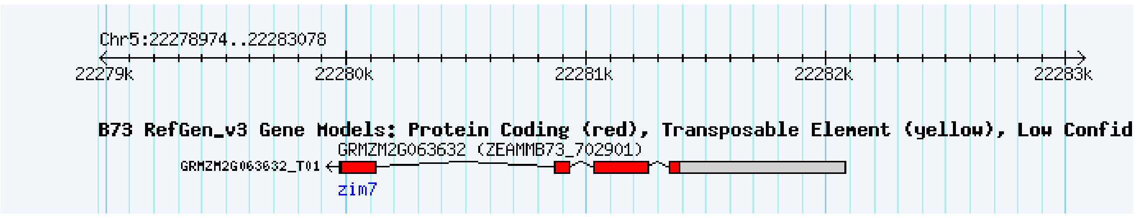

>GRMZM2G063632 Genomic DNA

GCCGGCGGCAAGGCCGACGCTGCTTACCCGCCCTCATCCGGCTACCCACCCGCGGCAGGCAAGCCAGGCAAGGGGGGCGA
ACCGGTGACGGCTTACCCTTCGGCCGGTCCCAGCACGGCGGCGCCCTACGCTAGGTTCTTCAGAAGCATTTATTTGTTTT
CCCACGGCAGTGCTAGAACAATAGTGTTATCGTTCTGCTATTTCAGTTCCATTTGTTTCCTGGTTAGTTGTTATCCTCCA
GGCTCTGAACAATAGGGACATCTGAATTAATCAGATTCCAGGAAAAATCGTTGTGACTAGATCCGATATCTGTATCACTA
AGGCCTACAAATGATTTGAACGCCACTGTCCAGAAAGCCAATCCGTTCACTGGCCAAGTAGACATACCTTTCTATCAGAA
TTTTACCTCCGTTTTTTTAATCATTTTAGGAAGTAGGACGATTGCTTTCTACCCCCGTGTCCGCGCACACATTCTATCCT
ATGCCGGTTTGAACCCACCACATGGCTTTCTGATATGCTTTTATCTGAACCAGTAGTGGGAGTGACATGGATCTGCCTAC
TTTCAATTCAGAGGTGCTGTTTTATCTGCCTTTGTGTGTTCTTATTTTTTTGCATTCTTTACTACAAGGTCTGCAATGTT
GCCGATGTCGAACCCTTCAGCTAATCCCACACAGCTTACAATTTTCTATGGTGGATCAGTATGTGTATGACTCGGTGCCA
CCAGAAAAGGTAAATATTATCTTCACGCCATTTACAGATATCTTGTCCTTCTGTACCATCTGTTAAAATACACATGCTAA
TACACTAATTTGGCATTGACAGGCTCAGGCAATCATGCTTATAGCTGCAGCTGCGGCAGCTGCGGCAGCCACCAAAGGCA
GTGCTGCCACTGCTTTTAATCCTCCAATGGTACATACAGCCACTGTCGCCCCAGCAGCAGTCTTCTCTCCTGTGCTTACA
CGGTCTCCATCACTGCAGAGCACTTCTGTAGCAGCTGGACAAGCTCAGGTTGTTGCTGACCCTAGCTCAATAAGCAAGCT
TCACGCTGGTAAGACCACTGCATCTTTGTGTAAAAATCCAGAAATGGAATACCTTTTCCAGTGTTGACATTGTCCAACAC
GTCTTTAAACTATCTTGATCTTTTTGCCAGATCTCCCCATTGCCAGGAGGCACTCTCTCCAGCGCTTCCTTGAGAAACGT
CGTGACAGGTTAGCATGATACATGAAAGCAACAGGGCTCTGATATTCTGAATCTTATTGTTAGGCCTCTTATTCTACAAT
CTACATGTTTAAAACTTTTGTTATAAACTGCTGTATGTGGTACCACACTAATTTTTTCCCCGACTATTGGCACCACAGTA
ATCAACAGTATATTTTGTACCGTATTCTGTTTTTTGTCCTCCAGGCAGGGTAAAAATAAAATAAAATTTGCTGTTATAGC
ATTTAAATGATTGCACTTTCTGCTGACAAGTGCTCAATGACATCCCTAAAAAAAATCTATAATTTCTTCACAGAAAGATT
GGTAGAGAGCCTGCTTCCATAATACAACATATAGCTGCAAATAGCCTTGGATCATGTAACTCCCTTGAAATTTTTTTTAA
AAAAAATCTGCAAATCGATATTAACACAAGACAGAGTTCTGGGACCAGCAGTAGATACAAAAACCAACATTACCCCCATT
TAGCTGCTCCAAATACCATGTTTTTCCCCTGTTAGGAAAATAACATATCTGAGAATCGGGATGTTCCTGTGTATTATCCT
GTTCTATTTGGAAAAGCTGGCCACTTAAAGATCCAGTCTCAAGGTTGCAAACTAGCACCATTTTTTTTTTCTGATGTAAG
CTTTAGATGCCTGTTTTTGCCTTATCTTAGAACTGAGTTTAGAATTGTTTCTTAGTATAACTTTTTTTATCGTGGATCTA
CTGATTTCAAGTGATTAAATAAATGATGGCACGAAACAGGGTCGTGCGCAAAGCTCCGTATAGCCCCGCCAAGTCGTTCG
ATGGCATGGAGTCAGCGGGAATGGAGATGACTGCGGACGGCAAGCAGGGCGCAAGGCCCAGTATCTTGAAAGGTTGGGGA
GCCGCGAAGTCTGAAGTCTGAACGG

>GRMZM2G063632_T01 cDNA

GCCGGCGGCAAGGCCGACGCTGCTTACCCGCCCTCATCCGGCTACCCACCCGCGGCAGGTCTGCAATGTTGCCGATGTCG
AACCCTTCAGCTAATCCCACACAGCTTACAATTTTCTATGGTGGATCAGTATGTGTATGACTCGGTGCCACCAGAAAAGG
CTCAGGCAATCATGCTTATAGCTGCAGCTGCGGCAGCTGCGGCAGCCACCAAAGGCAGTGCTGCCACTGCTTTTAATCCT
CCAATGGTACATACAGCCACTGTCGCCCCAGCAGCAGTCTTCTCTCCTGTGCTTACACGGTCTCCATCACTGCAGAGCAC
TTCTGTAGCAGCTGGACAAGCTCAGGTTGTTGCTGACCCTAGCTCAATAAGCAAGCTTCACGCTGATCTCCCCATTGCCA
GGAGGCACTCTCTCCAGCGCTTCCTTGAGAAACGTCGTGACAGGGTCGTGCGCAAAGCTCCGTATAGCCCCGCCAAGTCG
TTCGATGGCATGGAGTCAGCGGGAATGGAGATGACTGCGGACGGCAAGCAGGGCGCAAGGCCCAGTATCTTGAAAGGTTG
GGGAGCCGCGAAGTCTGAAGTCTGAACGG

>GRMZM2G063632_P01 peptide

MVDQYVYDSVPPEKAQAIMLIAAAAAAAAATKGSAATAFNPPMVHTATVAPAAVFSPVLTRSPSLQSTSVAAGQAQVVAD

PSSISKLHADLPIARRHSLQRFLEKRRDRVVRKAPYSPAKSFDGMESAGMEMTADGKQGARPSILKGWGAAKSEV

AGPv4:

Zm00001d013855 (zim7 - ZIM-transcription factor 7)

Chr5: 22766950..22770790

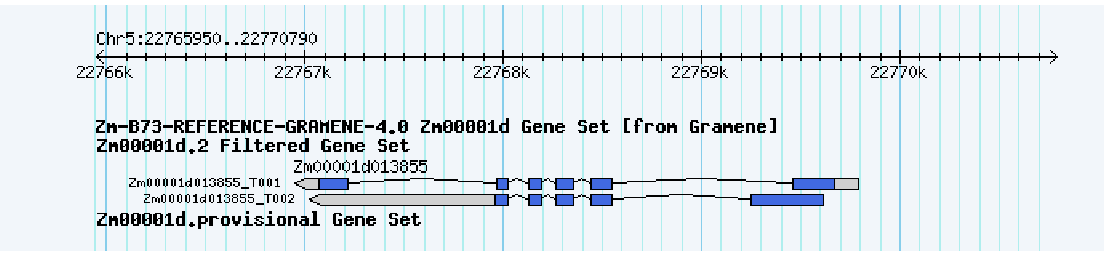

>Zm00001d013855 Genomic DNA

AGCCAGAGAGCGTCACCTTGGACCCTCAGGGTCGCGAGCCATACACGGGCGTCGCTGACGACACCACGCTGCCAACCCTA
GCCGCGCCTGCCTTCCTCCCCCGCTTGCCGTCGAGTATCGATGTTTGGAGGTGGGGGCGCCTTCGCCGGTGAGGTACCTG
GTGGGGGCAGCGATCATGATGAGCGGTGTGGTGCCTTCCTCCCTCCATGTCCTCCTTCGCGCCGAGCGCGCCCTCCGCGA
CCGCGACGTCGGCGAGGTCCACATCCCGCTGTCCGAGCTCCTCTCCGGCGCCCCCGACGGCCCCGTCCCCGCCAAGTTCG
TCGCCTACCAGGTGCGCAAGATCTCCTCCGGCAAGCCCCAGGGAGTCCTCAACCTCTCGTACAAGCTCGGCGAGGTCGCC
AACGGCTACGCCCCCGCCCCGCCGCCCAGTCCGCCTACGCCCAGCCTCCACCGACCGCCGCGTACCCGCCTCCTTCAGGC
AAGGCTGATGCCTACCCGCCTCCCGCCGCGTACCCGCCTCCTTCAGGCAAGGCCGACGCGTACGCCCCCCTGCCACCGCA
TACCCGCCGGCAGCCAAGGCCGACGCGTACCCACCTCCATACGGCGCCTACCCGCCCGCCGGCGGCAAGGCCGACGCTGC
TTACCCGCCCTCATCCGGCTACCCACCCGCGGCAGGCAAGCCAGGCAAGGGGGGCGAACCGGTGACGGCTTACCCTTCGG
CCGGTCCCAGCACGGCGGCGCCCTACGCTAGGTTCTTCAGAAGCATTTATTTGTTTTCCCACGGCAGTGCTAGAACAATA
GTGTTATCGTTCTGCTATTTCAGTTCCATTTGTTTCCTGGTTAGTTGTTATCCTCCAGGCTCTGAACAATAGGGACATCT
GAATTAATCAGATTCCAGGAAAAATCGTTGTGACTAGATCCGATATCTGTATCACTAAGGCCTACAAATGATTTGAACGC
CACTGTCCAGAAAGCCAATCCGTTCACTGGCCAAGTAGACATACCTTTCTATCAGAATTTTACCTCCGTTTTTTTAATCA
TTTTAGGAAGTAGGACGATTGCTTTCTACCCCCGTGTCCGCGCACACATTCTATCCTATGCCGGTTTGAACCCACCACAT
GGCTTTCTGATATGCTTTTATCTGAACCAGTAGTGGGAGTGACATGGATCTGCCTACTTTCAATTCAGAGGTGCTGTTTT
ATCTGCCTTTGTGTGTTCTTATTTTTTTGCATTCTTTACTACAAGGTCTGCAATGTTGCCGATGTCGAACCCTTCAGCTA
ATCCCACACAGCTTACAATTTTCTATGGTGGATCAGTATGTGTATGACTCGGTGCCACCAGAAAAGGTAAATATTATCTT
CACGCCATTTACAGATATCTTGTCCTTCTGTACCATCTGTTAAAATACACATGCTAATACACTAATTTGGCATTGACAGG
CTCAGGCAATCATGCTTATAGCTGCAGCTGCGGCAGCTGCGGCAGCCACCAAAGGCAGTGCTGCCACTGCTTTTAATCCT
CCAATGGTACATACAGCCACTGTCGCCCCAGCAGCAGTCTTCTCTCCTGTGCTTACACGGTCTCCATCACTGCAGAGCAC
TTCTGTAGCAGCTGGACAAGCTCAGGTTGTTGCTGACCCTAGCTCAATAAGCAAGCTTCACGCTGGTAAGACCACTGCAT
CTTTGTGTAAAAATCCAGAAATGGAATACCTTTTCCAGTGTTGACATTGTCCAACACGTCTTTAAACTATCTTGATCTTT
TTGCCAGATCTCCCCATTGCCAGGAGGCACTCTCTCCAGCGCTTCCTTGAGAAACGTCGTGACAGGTTAGCATGATACAT
GAAAGCAACAGGGCTCTGATATTCTGAATCTTATTGTTAGGCCTCTTATTCTACAATCTACATGTTTAAAACTTTTGTTA
TAAACTGCTGTATGTGGTACCACACTAATTTTTTCCCCGACTATTGGCACCACAGTAATCAACAGTATATTTTGTACCGT
ATTCTGTTTTTTGTCCTCCAGGCAGGGTAAAAATAAAATAAAATTTGCTGTTATAGCATTTAAATGATTGCACTTTCTGC
TGACAAGTGCTCAATGACATCCCTAAAAAAAATCTATAATTTCTTCACAGAAAGATTGGTAGAGAGCCTGCTTCCATAAT
ACAACATATAGCTGCAAATAGCCTTGGATCATGTAACTCCCTTGAAATTTTTTTTAAAAAAAATCTGCAAATCGATATTA
ACACAAGACAGAGTTCTGGGACCAGCAGTAGATACAAAAACCAACATTACCCCCATTTAGCTGCTCCAAATACCATGTTT
TTCCCCTGTTAGGAAAATAACATATCTGAGAATCGGGATGTTCCTGTGTATTATCCTGTTCTATTTGGAAAAGCTGGCCA
CTTAAAGATCCAGTCTCAAGGTTGCAAACTAGCACCATTTTTTTTTTCTGATGTAAGCTTTAGATGCCTGTTTTTGCCTT
ATCTTAGAACTGAGTTTAGAATTGTTTCTTAGTATAACTTTTTTTATCGTGGATCTACTGATTTCAAGTGATTAAATAAA

TGATGGCACGAAACAGGGTCGTGCGCAAAGCTCCGTATAGCCCCGCCAAGTCGTTCGATGGCATGGAGTCAGCGGGAATG
GAGATGACTGCGGACGGCAAGCAGGGCGCAAGGCCCAGTATCTTGAAAGGTTGGGGAGCCGCGAAGTCTGAAGTCTGAAC
GGTGAAAGCGTAGCCCCGCGGGAGCGTGGCGGTGTCTGGGAACCGTGAGAGCACCTAGAGGGGGGGGTGAATAGGTGATC
CTGTAAAAACTTAACTTATAGCCACAAAGACTTGTTAAGGG
>Zm00001d013855_T001 cDNA
Chr5: 22766950..22769790
AGCCAGAGAGCGTCACCTTGGACCCTCAGGGTCGCGAGCCATACACGGGCGTCGCTGACGACACCACGCTGCCAACCCTA
GCCGCGCCTGCCTTCCTCCCCCGCTTGCCGTCGAGTATCGATGTTTGGAGGTGGGGGCGCCTTCGCCGGTGAGGTACCTG
GTGGGGGCAGCGATCATGATGAGCGGTGTGGTGCCTTCCTCCCTCCATGTCCTCCTTCGCGCCGAGCGCGCCCTCCGCGA
CCGCGACGTCGGCGAGGTCCACATCCCGCTGTCCGAGCTCCTCTCCGGCGCCCCCGACGGCCCCGTCCCCGCCAAGTTCG
TCGCCTACCAGGTCTGCAATGTTGCCGATGTCGAACCCTTCAGCTAATCCCACACAGCTTACAATTTTCTATGGTGGATC
AGTATGTGTATGACTCGGTGCCACCAGAAAAGGCTCAGGCAATCATGCTTATAGCTGCAGCTGCGGCAGCTGCGGCAGCC
ACCAAAGGCAGTGCTGCCACTGCTTTTAATCCTCCAATGAGCACTTCTGTAGCAGCTGGACAAGCTCAGGTTGTTGCTGA
CCCTAGCTCAATAAGCAAGCTTCACGCTGATCTCCCCATTGCCAGGAGGCACTCTCTCCAGCGCTTCCTTGAGAAACGTC
GTGACAGGGTCGTGCGCAAAGCTCCGTATAGCCCCGCCAAGTCGTTCGATGGCATGGAGTCAGCGGGAATGGAGATGACT
GCGGACGGCAAGCAGGGCGCAAGGCCCAGTATCTTGAAAGGTTGGGGAGCCGCGAAGTCTGAAGTCTGAACGGTGAAAGC
GTAGCCCCGCGGGAGCGTGGCGGTGTCTGGGAACCGTGAGAGCACCTAGAGGGGGGGGTGAATAGGTGATCCTGTAAAAA
CTTAACTTATAGCCACAAAGACTTGTTAAGGG
>Zm00001d013855_P001 peptide
MFGGGGAFAGEVPGGGSDHDERCGAFLPPCPPSRRARPPRPRRRRGPHPAVRAPLRRPRRPRPRQVRRLPGLQCCRCRTL
QLIPHSLQFSMVDQYVYDSVPPEKAQAIMLIAAAAAAAAATKGSAATAFNPPMSTSVAAGQAQVVADPSSISKLHADLPI
ARRHSLQRFLEKRRDRVVRKAPYSPAKSFDGMESAGMEMTADGKQGARPSILKGWGAAKSEV
>Zm00001d013855_T002 cDNA
Chr5: 22767024..22769615
ATGATGAGCGGTGTGGTGCCTTCCTCCCTCCATGTCCTCCTTCGCGCCGAGCGCGCCCTCCGCGACCGCGACGTCGGCGA
GGTCCACATCCCGCTGTCCGAGCTCCTCTCCGGCGCCCCCGACGGCCCCGTCCCCGCCAAGTTCGTCGCCTACCAGGTGC
GCAAGATCTCCTCCGGCAAGCCCCAGGGAGTCCTCAACCTCTCGTACAAGCTCGGCGAGGTCGCCAACGGCTACGCCCCC
GCCCCGCCGCCCAGTCCGCCTACGCCCAGCCTCCACCGACCGCCGCGTACCCGCCTCCTTCAGGCAAGGCTGATGCCTAC
CCGCCTCCCGCCGCGTACCCGCCTCCTTCAGGCAAGGCCGACGCGTCTGCAATGTTGCCGATGTCGAACCCTTCAGCTAA
TCCCACACAGCTTACAATTTTCTATGGTGGATCAGTATGTGTATGACTCGGTGCCACCAGAAAAGGCTCAGGCAATCATG
CTTATAGCTGCAGCTGCGGCAGCTGCGGCAGCCACCAAAGGCAGTGCTGCCACTGCTTTTAATCCTCCAATGAGCACTTC
TGTAGCAGCTGGACAAGCTCAGGTTGTTGCTGACCCTAGCTCAATAAGCAAGCTTCACGCTGATCTCCCCATTGCCAGGA
GGCACTCTCTCCAGCGCTTCCTTGAGAAACGTCGTGACAGGTTAGCATGATACATGAAAGCAACAGGGCTCTGATATTCT
GAATCTTATTGTTAGGCCTCTTATTCTACAATCTACATGTTTAAAACTTTTGTTATAAACTGCTGTATGTGGTACCACAC
TAATTTTTTCCCCGACTATTGGCACCACAGTAATCAACAGTATATTTTGTACCGTATTCTGTTTTTTGTCCTCCAGGCAG
GGTAAAAATAAAATAAAATTTGCTGTTATAGCATTTAAATGATTGCACTTTCTGCTGACAAGTGCTCAATGACATCCCTA
AAAAAAATCTATAATTTCTTCACAGAAAGATTGGTAGAGAGCCTGCTTCCATAATACAACATATAGCTGCAAATAGCCTT
GGATCATGTAACTCCCTTGAAATTTTTTTTAAAAAAAATCTGCAAATCGATATTAACACAAGACAGAGTTCTGGGACCAG
CAGTAGATACAAAAACCAACATTACCCCCATTTAGCTGCTCCAAATACCATGTTTTTCCCCTGTTAGGAAAATAACATAT
CTGAGAATCGGGATGTTCCTGTGTATTATCCTGTTCTATTTGGAAAAGCTGGCCACTTAAAGATCCAGTCTCAAGGTTGC
AAACTAGCACCATTTTTTTTTTTCTGATGTAAGCTTTAGATGCCTGTTTTTGCCTTATCTTAGAACTGAGTTTAGAATTGT
TTCTTAGTATAACTTTTTTTATCGTGGATCTACTGATTTCAAGTGATTAAATAAATGATGGCACGAAACAGGGTCGTGCG
CAAAGCTCCGTATAGCCCCGCCAAGTCGTTCGATGGCATGGAGTCAGCGGGAATGGAGATGACTGCGGACGGCAAGCAGG
GCGCAAGGCCCAGTATCTTGAAAGGTTGGGGAGCCGCGAAGTCTGAAGTCTGAACGGTGAAAGCGTAGCCCCGCGGGAGC

GTGGCGGTGTCTGGGAACCGTG
>Zm00001d013855_P002 peptide
MMSGVVPSSLHVLLRAERALRDRDVGEVHIPLSELLSGAPDGPVPAKFVAYQVRKISSGKPQGVLNLSYKLGEVANGYAP
APPPSPPTPSLHRPPRTRLLQARLMPTRLPPRTRLLQARPTRLQCCRCRTLQLIPHSLQFSMVDQYVYDSVPPEKAQAIM
LIAAAAAAAAATKGSAATAFNPPMSTSVAAGQAQVVADPSSISKLHADLPIARRHSLQRFLEKRRDRLA

ZmJAZ26

B73 RefGen_v3:
GRMZM2G114681 (zim15 - ZIM-transcription factor 15)
Chr2: 180085933..180090653

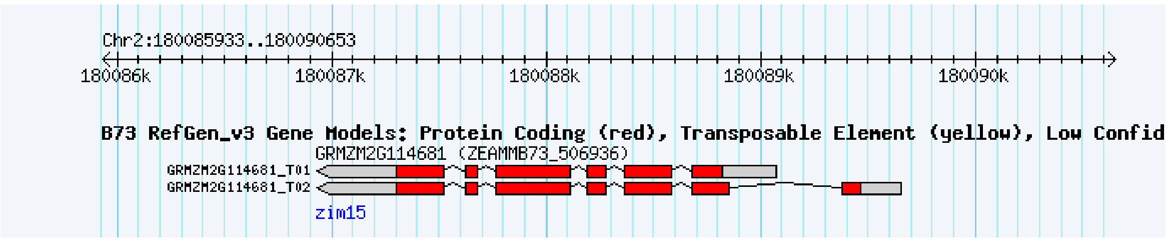

>GRMZM2G114681 Genomic DNA
CGCGCGCATCGTCATCAGTTCACTAGTCAGTCGAGGTCGAGGGGCGGACGAGGAGCTGAGGAGAGAGAAGCTTCAAGGAG
TTGGGGACTTGGGGGTTGCCGAGCTGTCCGTCTTTCCCCTCCCCTCCACCCACCAGCTACCTCGGGAGGGCAAGGCAAAC
ATCTGCGGCCTCCTTTCCCGAACCCGGGTTGGATGGAGCGGGACTTCCTGGCCGCGATCGGCAAGGAGCAGCAGCACCCG
CGCAAGGAGAAGGCAGGCGGAGGCGCGGAGGAATCAGGTAGGCTCCCCCGCCCTCCTTTCGCCGCCTGCCGCGCTGCGTT
TCTTGATCTGTACGCGCGCCGTGCCGTGCTGGGTTGGGTTGGGTTGGGCTCCGCTCACTCCATTCGTTTCGGCGAGATGC
GGCTTGGGTGCGCTGCGAAATTCAAGCGCCCCTTCCTCTCTCTCTCTCTCCTGTCTTATTTATTTATTTATTTATTTTGG
TTGTGCTCGCAACAAAGAGTGTGCGGGCTTGGCTGCCATGCCTGCCGAATTCCTTCTGACGGCCGGCCTGCCGTGTACGC
TCGATTCGGGCGAGAGTTAATGCGTGAAAAGCCACGATGGAGAGTGGCCGAACCTTTCGGACAGCCGCGCGTGTCGTTAG
TTGATTTCGAGCTGCCTCTGCCTCTGCTTCTGCTTGCTTGCTGGTGGGAAGGACGGGGATGCTTAACGGATCCATCTCTG
GGTTCGTGCTTTGTTGTTGTATGTATTTATGTGCTGCCGCCTACCGGAATCTGATTCCCAGAGCCGTGTGCAATCTCTGC
CAGCTTACTTCGGGGCAGCAGCCGTCCCGGCGATGGACTGGTCCTTCGCGAGCAAGCCCTGCGCCGCCCCGGCGCTGATG
TCGTTCCGGTCGGCGGCGAGGGAGGAGCCTTCGTTCCCGCAGTTCTCCGCCTTGGACGGCACCAAGAACACAGCCCCTCG
TATGCTCACGCACCAGGTACGCGCGGCAGCTCTTCTTGGTTCTGACTGATTCCTGTCAGCTCGCGTGTTTCGGCCCTCTG
ATGGTGTTTCTTCTTTTTTTTTGTTTCAGAGATCGTTCGGCCCCGACAGCACGCAGTACGCCGCGCTGCACCGCGCGCAG
AACGGGGCTAGAGTCGTTCCGGTCTCGTCGCCGTTCAGCCAGAGCAACCCGATGTTCAGGGTCCAGAGCTCGCCCAGCCT
TCCGAACAGTACCGCGTTCAAGCAGCCGCCTTTCGCCATCAGCAACGCCGTGGCCAGTTCGACTGTGGGTTCCTATGGCG
GAACAAGGTACTAGTTTGTTTGCTGTTAGTCTGTTACGACCATGACTTGTACAAGTGGCTGTGAAATTAGCTTCGACTGA
AACGCTTTGCCTGCGAAGGGATGCGGTGAGGCCAAGGACGGCACAACTGACGATCTTCTACGCCGGTTCCGTCAATGTGT
TCAACAACGTCTCAGCTGAGAAGGTATAGTTTCTCTCAGGATCATTTCATTCTAGTTCGCTTTGCCTTTGGGCTCCCTCG
TTGACATATTTCGCATCGCCACAGGCTCAGGAGCTCATGTTCCTAGCCAGCAGAGGAAGCTCAGCCCCCGTGGCCTGTAA
GCCAGAGGCTCCTCCTACTCTCGCTCCAGCGAAGGTCACAGCGCCTGAGGTTCTTCTGCCTGCGAAGCAGATGCTGTTTC
AAAAACCACAGCATCTCTCGCCGCCTCCATCCAGTGTCCCTGGCATCTTGCAGTCTGCAGCTCTCCCCAGGAGCGCCTCA
TCTAGCTCTAACCTTGACTCCCCAGCTCCAAAATCTTCGGTCCCGTTAGCTGTTCCTCCTGTAAGCCAGGCTCCGCCAGC
AACACTGATCGCCACTACAACTGCAGCAGCAATAATGCCTAGAGGTACGGTACTGCTGGCTGCATCGTAGTTCTCGTTGC
TCTCTTCCTCCACTGTATTGAAATTGTATCGTTCTTGTCCGTCCGATGCAGCTGTCCCTCAAGCTAGGAAGGCGTCCCTT
GCTCGATTCTTGGAGAAAAGGAAGGAAAGGTAGAATCCATACTCTTATTCTCGGTGATTCCATTCCACCACACATCTGTT

TTGGTTTTGAGGCAGATCTGCAACTGTTCTGACTCTGTGCGTGCTTCTCTCGCAGGGTGACAACTGCCGCGCCTTACCCA
TCAGCCAAGAGCCCACTGGAGAGCAGCGACACGTTCGGCAGCGGGAGCGCCAGCGCCAACGCCAACGACAAGTCCTCGTG
CACAGACATTGCCCTCTCCAGCAACCATGAAGAGTCCCTGTGTTTAGGGGGGCAGCCCAGGAGCATCATCAGCTTCAGCG
AAGAGTCCCCCAGCACCAAGCTACAGATCTGATCGTGCCGTGCCCAGTGTTCATAGATTTGTCCAGGCTGACCGATGCAG
AAAACCATAAGAATACTTTTTTCCACTCAAGAGGAAGAGATTACTATCGATGCCGTTGTTGTTTACCGTGATGCTGTTTT
GCCGTGTTAGGTTACTGTACTGCTGGTACAGATGGTAGTGCCCCCCACCCACCTAACTAACAAGAGAGCTGTCACTTGCA
GACTCTTGATTTTTTTTTGCCATTTCTCTTGGAAATGAACTCAGATGTGTTATTCTAGCAGTAATCAAATTATACAGGCA
TACAAATTCTGCGCCATTTGTATGGCCAAAGTGTAATACACTTGTTCTTGAATTATGAACCTCTCTCAAGCTGCAGAATG
C
>GRMZM2G114681_T01 cDNA
Chr2: 180086933..180089072
GCGTGAAAAGCCACGATGGAGAGTGGCCGAACCTTTCGGACAGCCGCGCGTGTCGTTAGTTGATTTCGAGCTGCCTCTGC
CTCTGCTTCTGCTTGCTTGCTGGTGGGAAGGACGGGGATGCTTAACGGATCCATCTCTGGCTTACTTCGGGGCAGCAGCC
GTCCCGGCGATGGACTGGTCCTTCGCGAGCAAGCCCTGCGCCGCCCCGGCGCTGATGTCGTTCCGGTCGGCGGCGAGGGA
GGAGCCTTCGTTCCCGCAGTTCTCCGCCTTGGACGGCACCAAGAACACAGCCCCTCGTATGCTCACGCACCAGAGATCGT
TCGGCCCCGACAGCACGCAGTACGCCGCGCTGCACCGCGCGCAGAACGGGGCTAGAGTCGTTCCGGTCTCGTCGCCGTTC
AGCCAGAGCAACCCGATGTTCAGGGTCCAGAGCTCGCCCAGCCTTCCGAACAGTACCGCGTTCAAGCAGCCGCCTTTCGC
CATCAGCAACGCCGTGGCCAGTTCGACTGTGGGTTCCTATGGCGGAACAAGGGATGCGGTGAGGCCAAGGACGGCACAAC
TGACGATCTTCTACGCCGGTTCCGTCAATGTGTTCAACAACGTCTCAGCTGAGAAGGCTCAGGAGCTCATGTTCCTAGCC
AGCAGAGGAAGCTCAGCCCCCGTGGCCTGTAAGCCAGAGGCTCCTCCTACTCTCGCTCCAGCGAAGGTCACAGCGCCTGA
GGTTCTTCTGCCTGCGAAGCAGATGCTGTTTCAAAAACCACAGCATCTCTCGCCGCCTCCATCCAGTGTCCCTGGCATCT
TGCAGTCTGCAGCTCTCCCCAGGAGCGCCTCATCTAGCTCTAACCTTGACTCCCCAGCTCCAAAATCTTCGGTCCCGTTA
GCTGTTCCTCCTGTAAGCCAGGCTCCGCCAGCAACACTGATCGCCACTACAACTGCAGCAGCAATAATGCCTAGAGCTGT
CCCTCAAGCTAGGAAGGCGTCCCTTGCTCGATTCTTGGAGAAAAGGAAGGAAAGGGTGACAACTGCCGCGCCTTACCCAT
CAGCCAAGAGCCCACTGGAGAGCAGCGACACGTTCGGCAGCGGGAGCGCCAGCGCCAACGCCAACGACAAGTCCTCGTGC
ACAGACATTGCCCTCTCCAGCAACCATGAAGAGTCCCTGTGTTTAGGGGGGCAGCCCAGGAGCATCATCAGCTTCAGCGA
AGAGTCCCCCAGCACCAAGCTACAGATCTGATCGTGCCGTGCCCAGTGTTCATAGATTTGTCCAGGCTGACCGATGCAGA
AAACCATAAGAATACTTTTTTCCACTCAAGAGGAAGAGATTACTATCGATGCCGTTGTTGTTTACCGTGATGCTGTTTTG
CCGTGTTAGGTTACTGTACTGCTGGTACAGATGGTAGTGCCCCCCACCCACCTAACTAACAAGAGAGCTGTCACTTGCAG
ACTCTTGATTTTTTTTTGCCATTTCTCTTGGAAATGAACTCAGATGTGTTATTCTAGCAGTAATCAAATTATACAGGCAT
ACAAATTCTGCGCCATTTGTATGGCCAAAGTGTAATACACTTGTTCTTGAATTATGAACCTCTCTCAAGCTGCAGAATGC
>GRMZM2G114681_P01 peptide
MDWSFASKPCAAPALMSFRSAAREEPSFPQFSALDGTKNTAPRMLTHQRSFGPDSTQYAALHRAQNGARVVPVSSPFSQS
NPMFRVQSSPSLPNSTAFKQPPFAISNAVASSTVGSYGGTRDAVRPRTAQLTIFYAGSVNVFNNVSAEKAQELMFLASRG
SSAPVACKPEAPPTLAPAKVTAPEVLLPAKQMLFQKPQHLSPPPSSVPGILQSAALPRSASSSNLDSPAPKSSVPLAVP
PVSQAPPATLIATTAAAIMPRAVPQARKASLARFLEKRKERVTTAAPYPSAKSPLESSDTFGSGSASANANDKSSCTDI
ALSSNHEESLCLGGQPRSIISFSEESPSTKLQI
>GRMZM2G114681_T02 cDNA
Chr2: 180086933..180089653
CGCGCGCATCGTCATCAGTTCACTAGTCAGTCGAGGTCGAGGGGCGGACGAGGAGCTGAGGAGAGAGAAGCTTCAAGGAG
TTGGGGACTTGGGGGTTGCCGAGCTGTCCGTCTTTCCCCTCCCCTCCACCCACCAGCTACCTCGGGAGGGCAAGGCAAAC
ATCTGCGGCCTCCTTTCCCGAACCCGGGTTGGATGGAGCGGGACTTCCTGGCCGCGATCGGCAAGGAGCAGCAGCACCCG
CGCAAGGAGAAGGCAGGCGGAGGCGCGGAGGAATCAGCTTACTTCGGGGCAGCAGCCGTCCCGGCGATGGACTGGTCCTT
CGCGAGCAAGCCCTGCGCCGCCCCGGCGCTGATGTCGTTCCGGTCGGCGGCGAGGGAGGAGCCTTCGTTCCCGCAGTTCT

CCGCCTTGGACGGCACCAAGAACACAGCCCCTCGTATGCTCACGCACCAGAGATCGTTCGGCCCCGACAGCACGCAGTAC
GCCGCGCTGCACCGCGCGCAGAACGGGGCTAGAGTCGTTCCGGTCTCGTCGCCGTTCAGCCAGAGCAACCCGATGTTCAG
GGTCCAGAGCTCGCCCAGCCTTCCGAACAGTACCGCGTTCAAGCAGCCGCCTTTCGCCATCAGCAACGCCGTGGCCAGTT
CGACTGTGGGTTCCTATGGCGGAACAAGGGATGCGGTGAGGCCAAGGACGGCACAACTGACGATCTTCTACGCCGGTTCC
GTCAATGTGTTCAACAACGTCTCAGCTGAGAAGGCTCAGGAGCTCATGTTCCTAGCCAGCAGAGGAAGCTCAGCCCCCGT
GGCCTGTAAGCCAGAGGCTCCTCCTACTCTCGCTCCAGCGAAGGTCACAGCGCCTGAGGTTCTTCTGCCTGCGAAGCAGA
TGCTGTTTCAAAAACCACAGCATCTCTCGCCGCCTCCATCCAGTGTCCCTGGCATCTTGCAGTCTGCAGCTCTCCCCAGG
AGCGCCTCATCTAGCTCTAACCTTGACTCCCCAGCTCCAAAATCTTCGGTCCCGTTAGCTGTTCCTCCTGTAAGCCAGGC
TCCGCCAGCAACACTGATCGCCACTACAACTGCAGCAGCAATAATGCCTAGAGCTGTCCCTCAAGCTAGGAAGGCGTCCC
TTGCTCGATTCTTGGAGAAAAGGAAGGAAAGGGTGACAACTGCCGCGCCTTACCCATCAGCCAAGAGCCCACTGGAGAGC
AGCGACACGTTCGGCAGCGGGAGCGCCAGCGCCAACGCCAACGACAAGTCCTCGTGCACAGACATTGCCCTCTCCAGCAA
CCATGAAGAGTCCCTGTGTTTAGGGGGGCAGCCCAGGAGCATCATCAGCTTCAGCGAAGAGTCCCCCAGCACCAAGCTAC
AGATCTGATCGTGCCGTGCCCAGTGTTCATAGATTTGTCCAGGCTGACCGATGCAGAAAACCATAAGAATACTTTTTTCC
ACTCAAGAGGAAGAGATTACTATCGATGCCGTTGTTGTTTACCGTGATGCTGTTTTGCCGTGTTAGGTTACTGTACTGCT
GGTACAGATGGTAGTGCCCCCCACCCACCTAACTAACAAGAGAGCTGTCACTTGCAGACTCTTGATTTTTTTTTGCCATT
TCTCTTGGAAATGAACTCAGATGTGTTATTCTAGCAGTAATCAAATTATACAGGCATACAAATTCTGCGCCATTTGTATG
GCCAAAGTGTAATACACTTGTTCTTGAATTATGAACCTCTCTCAAGCTGCAGAATGC

>GRMZM2G114681_P02 peptide

MERDFLAAIGKEQQHPRKEKAGGGAEESAYFGAAAVPAMDWSFASKPCAAPALMSFRSAAREEPSFPQFSALDGTKNTAP
RMLTHQRSFGPDSTQYAALHRAQNGARVVPVSSPFSQSNPMFRVQSSPSLPNSTAFKQPPFAISNAVASSTVGSYGGTRD
AVRPRTAQLTIFYAGSVNVFNNVSAEKAQELMFLASRGSSAPVACKPEAPPTLAPAKVTAPEVLLPAKQMLFQKPQHLSP
PPSSVPGILQSAALPRSASSSSNLDSPAPKSSVPLAVPPVSQAPPATLIATTTAAAIMPRAVPQARKASLARFLEKRKER
VTTAAPYPSAKSPLESSDTFGSGSASANANDKSSCTDIALSSNHEESLCLGGQPRSIISFSEESPSTKLQI

AGPv4:

Zm00001d005726 (zim15 - ZIM-transcription factor 15)

Chr2: 184841614..184846272

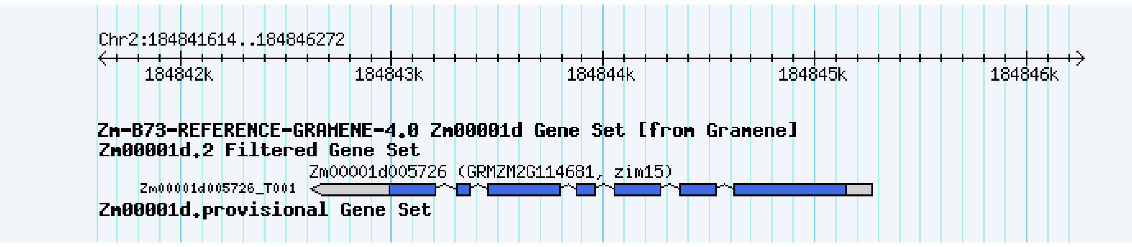

>Zm00001d005726 Genomic DNA

GAAGCTTCAAGGAGTTGGGGACTTGGGGGTTGCCGAGCTGTCCGTCTTTCCCCTCCCCTCCACCCACCAGCTACCTCGGG
AGGGCAAGGCAAACATCTGCGGCCTCCTTTCCCGAACCCGGGTTGGATGGAGCGGGACTTCCTGGCCGCGATCGGCAAGG
AGCAGCAGCACCCGCGCAAGGAGAAGGCAGGCGGAGGCGCGGAGGAATCAGGTAGGCTCCCCCGCCCTCCTTTCGCCGCC
TGCCGCGCTGCGTTTCTTGATCTGTACGCGCGCCGTGCCGTGCTGGGTTGGGTTGGGTTGGGCTCCGCTCACTCCATTCG
TTTCGGCGAGATGCGGCTTGGGTGCGCTGCGAAATTCAAGCGCCCCTTCCTCTCTCTCTCTCCTGTCTTATTTATTTA
TTTATTTATTTTGGTTGTGCTCGCAACAAAGAGTGTGCGGGCTTGGCTGCCATGCCTGCCGAATTCCTTCTGACGGCCGG
CCTGCCGTGTACGCTCGATTCGGGCGAGAGTTAATGCGTGAAAAGCCACGATGGAGAGTGGCCGAACCTTTCGGACAGCC
GCGCGTGTCGTTAGTTGATTTCGAGCTGCCTCTGCCTCTGCTTCTGCTTGCTTGCTGGTGGGAAGGACGGGGATGCTTAA
CGGATCCATCTCTGGGTTCGTGCTTTGTTGTTGTATGTATTTATGTGCTGCCGCCTACCGGAATCTGATTCCCAGAGCCG
TGTGCAATCTCTGCCAGCTTACTTCGGGGCAGCAGCCGTCCCGGCGATGGACTGGTCCTTCGCGAGCAAGCCCTGCGCCG
CCCCGGCGCTGATGTCGTTCCGGTCGGCGGCGAGGGAGGAGCCTTCGTTCCCGCAGTTCTCCGCCTTGGACGGCACCAAG

AACACAGCCCCTCGTATGCTCACGCACCAGGTACGCGCGGCAGCTCTTCTTGGTTCTGACTGATTCCTGTCAGCTCGCGT
GTTTCGGCCCTCTGATGGTGTTTCTTCTTTTTTTTTGTTTCAGAGATCGTTCGGCCCCGACAGCACGCAGTACGCCGCGC
TGCACCGCGCGCAGAACGGGGCTAGAGTCGTTCCGGTCTCGTCGCCGTTCAGCCAGAGCAACCCGATGTTCAGGGTCCAG
AGCTCGCCCAGCCTTCCGAACAGTACCGCGTTCAAGCAGCCGCCTTTCGCCATCAGCAACGCCGTGGCCAGTTCGACTGT
GGGTTCCTATGGCGGAACAAGGTACTAGTTTGTTTGCTGTTAGTCTGTTACGACCATGACTTGTACAAGTGGCTGTGAAA
TTAGCTTCGACTGAAACGCTTTGCCTGCGAAGGGATGCGGTGAGGCCAAGGACGGCACAACTGACGATCTTCTACGCCGG
TTCCGTCAATGTGTTCAACAACGTCTCAGCTGAGAAGGTATAGTTTCTCTCAGGATCATTTCATTCTAGTTCGCTTTGCC
TTTGGGCTCCCTCGTTGACATATTTCGCATCGCCACAGGCTCAGGAGCTCATGTTCCTAGCCAGCAGAGGAAGCTCAGCC
CCCGTGGCCTGTAAGCCAGAGGCTCCTCCTACTCTCGCTCCAGCGAAGGTCACAGCGCCTGAGGTTCTTCTGCCTGCGAA
GCAGATGCTGTTTCAAAAACCACAGCATCTCTCGCCGCCTCCATCCAGTGTCCCTGGCATCTTGCAGTCTGCAGCTCTCC
CCAGGAGCGCCTCATCTAGCTCTAACCTTGACTCCCCAGCTCCAAAATCTTCGGTCCCGTTAGCTGTTCCTCCTGTAAGC
CAGGCTCCGCCAGCAACACTGATCGCCACTACAACTGCAGCAGCAATAATGCCTAGAGGTACGGTACTGCTGGCTGCATC
GTAGTTCTCGTTGCTCTCTTCCTCCACTGTATTGAAATTGTATCGTTCTTGTCCGTCCGATGCAGCTGTCCCTCAAGCTA
GGAAGGCGTCCCTTGCTCGATTCTTGGAGAAAAGGAAGGAAAGGTAGAATCCATACTCTTATTCTCGGTGATTCCATTCC
ACCACACATCTGTTTTGGTTTTGAGGCAGATCTGCAACTGTTCTGACTCTGTGCGTGCTTCTCTCGCAGGGTGACAACTG
CCGCGCCTTACCCATCAGCCAAGAGCCCACTGGAGAGCAGCGACACGTTCGGCAGCGGGAGCGCCAGCGCCAACGCCAAC
GACAAGTCCTCGTGCACAGACATTGCCCTCTCCAGCAACCATGAAGAGTCCCTGTGTTTAGGGGGGCAGCCCAGGAGCAT
CATCAGCTTCAGCGAAGAGTCCCCCAGCACCAAGCTACAGATCTGATCGTGCCGTGCCCAGTGTTCATAGATTTGTCCAG
GCTGACCGATGCAGAAAACCATAAGAATACTTTTTTCCACTCAAGAGGAAGAGATTACTATCGATGCCGTTGTTGTTTAC
CGTGATGCTGTTTTGCCGTGTTAGGTTACTGTACTGCTGGTACAGATGGTAGTGCCCCCCACCCACCTAACTAACAAGAG
AGCTGTCACTTGCAGACTCTTGATTTTTTTTTGCCATTTCTCTTGGAAATGAACTCAGATGTGTTATTCTAGCAGTAATC
AAATTATACAGGCATACAAATTCTGCGCCATTTGTATGGCCAAAGTGTAATACACTTGTTCTTGAATTATGAACCTCTCT
CAAGCTGCAGAATGCAATT
>Zm00001d005726_T001 cDNA
GAAGCTTCAAGGAGTTGGGGACTTGGGGGTTGCCGAGCTGTCCGTCTTTCCCCTCCCCTCCACCCACCAGCTACCTCGGG
AGGGCAAGGCAAACATCTGCGGCCTCCTTTCCCGAACCCGGGTTGGATGGAGCGGGACTTCCTGGCCGCGATCGGCAAGG
AGCAGCAGCACCCGCGCAAGGAGAAGGCAGGCGGAGGCGCGGAGGAATCAGGTAGGCTCCCCCGCCCTCCTTTCGCCGCC
TGCCGCGCTGCGTTTCTTGATCTGTACGCGCGCCGTGCCGTGCTGGGTTGGGTTGGGTTGGGCTCCGCTCACTCCATTCG
TTTCGGCGAGATGCGGCTTGGGTGCGCTGCGAAATTCAAGCGCCCCTTCCTCTCTCTCTCTCTCCTGTCTTATTTATTTA
TTTATTTATTTTGGTTGTGCTCGCAACAAAGAGTGTGCGGGCTTGGCTGCCATGCCTGCCGAATTCCTTCTGACGGCCGG
CCTGCCGTGTACGCTCGATTCGGGCGAGAGTTAATGCGTGAAAAGCCACGATGGAGAGTGGCCGAACCTTTCGGACAGCC
GCGCGTGTCGTTAGTTGATTTCGAGCTGCCTCTGCCTCTGCTTCTGCTTGCTTGCTGGTGGGAAGGACGGGGATGCTTAA
CGGATCCATCTCTGGCTTACTTCGGGGCAGCAGCCGTCCCGGCGATGGACTGGTCCTTCGCGAGCAAGCCCTGCGCCGCC
CCGGCGCTGATGTCGTTCCGGTCGGCGGCGAGGGAGGAGCCTTCGTTCCCGCAGTTCTCCGCCTTGGACGGCACCAAGAA
CACAGCCCCTCGTATGCTCACGCACCAGAGATCGTTCGGCCCCGACAGCACGCAGTACGCCGCGCTGCACCGCGCGCAGA
ACGGGGCTAGAGTCGTTCCGGTCTCGTCGCCGTTCAGCCAGAGCAACCCGATGTTCAGGGTCCAGAGCTCGCCCAGCCTT
CCGAACAGTACCGCGTTCAAGCAGCCGCCTTTCGCCATCAGCAACGCCGTGGCCAGTTCGACTGTGGGTTCCTATGGCGG
AACAAGGGATGCGGTGAGGCCAAGGACGGCACAACTGACGATCTTCTACGCCGGTTCCGTCAATGTGTTCAACAACGTCT
CAGCTGAGAAGGCTCAGGAGCTCATGTTCCTAGCCAGCAGAGGAAGCTCAGCCCCCGTGGCCTGTAAGCCAGAGGCTCCT
CCTACTCTCGCTCCAGCGAAGGTCACAGCGCCTGAGGTTCTTCTGCCTGCGAAGCAGATGCTGTTTCAAAAACCACAGCA
TCTCTCGCCGCCTCCATCCAGTGTCCCTGGCATCTTGCAGTCTGCAGCTCTCCCCAGGAGCGCCTCATCTAGCTCTAACC
TTGACTCCCCAGCTCCAAAATCTTCGGTCCCGTTAGCTGTTCCTCCTGTAAGCCAGGCTCCGCCAGCAACACTGATCGCC
ACTACAACTGCAGCAGCAATAATGCCTAGAGCTGTCCCTCAAGCTAGGAAGGCGTCCCTTGCTCGATTCTTGGAGAAAAG
GAAGGAAAGGGTGACAACTGCCGCGCCTTACCCATCAGCCAAGAGCCCACTGGAGAGCAGCGACACGTTCGGCAGCGGGA

GCGCCAGCGCCAACGCCAACGACAAGTCCTCGTGCACAGACATTGCCCTCTCCAGCAACCATGAAGAGTCCCTGTGTTTA
GGGGGGCAGCCCAGGAGCATCATCAGCTTCAGCGAAGAGTCCCCCAGCACCAAGCTACAGATCTGATCGTGCCGTGCCCA
GTGTTCATAGATTTGTCCAGGCTGACCGATGCAGAAAACCATAAGAATACTTTTTTCCACTCAAGAGGAAGAGATTACTA
TCGATGCCGTTGTTGTTTACCGTGATGCTGTTTTGCCGTGTTAGGTTACTGTACTGCTGGTACAGATGGTAGTGCCCCCC
ACCCACCTAACTAACAAGAGAGCTGTCACTTGCAGACTCTTGATTTTTTTTTGCCATTTCTCTTGGAAATGAACTCAGAT
GTGTTATTCTAGCAGTAATCAAATTATACAGGCATACAAATTCTGCGCCATTTGTATGGCCAAAGTGTAATACACTTGTT
CTTGAATTATGAACCTCTCTCAAGCTGCAGAATGCAATT
>Zm00001d005726_P001 peptide
MERDFLAAIGKEQQHPRKEKAGGGAEESGRLPRPPFAACRAAFLDLYARRAVLGWVGLGSAHSIRFGEMRLGCAAKFKRP
FLSLSLLSYLFIYLFWLCSQQRVCGLGCHACRIPSDGRPAVYARFGRELMREKPRWRVAEPFGQPRVSLVDFELPLPLLL
LACWWEGRGCLTDPSLAYFGAAAVPAMDWSFASKPCAAPALMSFRSAAREEPSFPQFSALDGTKNTAPRMLTHQRSFGPD
STQYAALHRAQNGARVVPVSSPFSQSNPMFRVQSSPSLPNSTAFKQPPFAISNAVASSTVGSYGGTRDAVRPRTAQLTIF
YAGSVNVFNNVSAEKAQELMFLASRGSSAPVACKPEAPPTLAPAKVTAPEVLLPAKQMLFQKPQHLSPPPSSVPGILQSA
ALPRSASSSSNLDSPAPKSSVPLAVPPVSQAPPATLIATTTAAAIMPRAVPQARKASLARFLEKRKERVTTAAPYPSAKS
PLESSDTFGSGSASANANDKSSCTDIALSSNHEESLCLGGQPRSIISFSEESPSTKLQI

ZmJAZ27

B73 RefGen_v3:
GRMZM5G838098 (zim27 - ZIM-transcription factor 27)
Chr1: 16782463..16783895

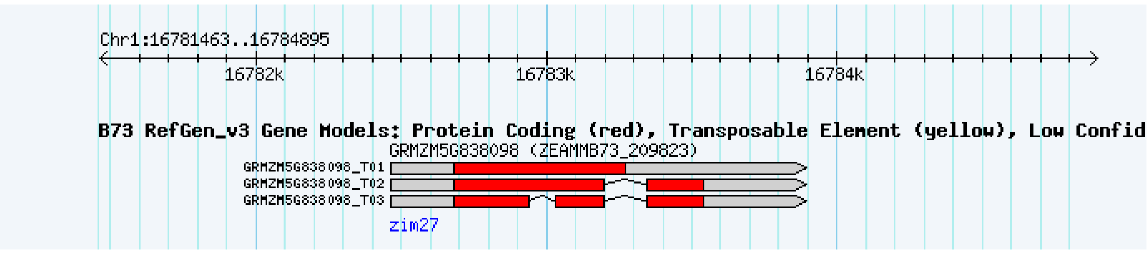

>GRMZM5G838098 Genomic DNA
GACCGCACCTCGTATCGTCGTCTAGCTCCGTTTCGTTTCCGGGAAGCAACCAACCTTCGTTCCCGGGCAGCCGACGACCC
TTGCGCCGTCTTCGTCTGTCAAGAGTACAGTTTGACGCGAGCAGCTGCCGGTTCCTTCTCTCGTGCGCCTTTGCTAGCTT
GGGATCGATCAAAGCCGGTGAGCGACCGAGCGAGCGAGTGAGGATCGGAACATCTGCCATCATGGCCTCCGAGCATGCGC
AGCTGTCTGCCAAGGCCGCCGCCGGCAGCAGGTTCGCGGTGACGTACGGCCTGCTGAGGCAGTACATGAAGGAGCAGGGC
GGCAGCGGCGCCACCAGGAGCCTCGCGCCGGCCGTGGGCGTGGGCCTCATGCCGGAAGCCGACGCTGCTGCGGCGGCGGA
GGCGACCGAGGAGAGGACCACCGTGCTGGAGCTCTTCCCGCAGCAGGCTGGCACGCTCAAAGACGAGCAGCAAAGGTGTG
TTAACTCGCCGTTGCCTTCAGTCAGTGCCTCACCGTGCGCGTGTGAACTTCTGATCGCGGATCTCTCCGTCGTCCGTCGT
CCCCTTAGGAAGAGGAAGGAGCGTGCTGACGGGAGAGCGCCGCTGACCATCTTCTACGGCGGCAAGATGGTGGTCTTCGA
CGACTTCCCCGCCGAGAAGGCCGAGGAGCTGATGCAGCTCGCCGGCTCCGGCAACGCCGCCCAGGACGCGCTGGGACAGC
CCAGCCTCACAGGTACGAACACGAACGACTCAACGCATATACGTACGCTGTCCAGAGCTCACACGGGCAGCCTGCAGAGG
CCCATGTAGTAGCGTCCAGCAGCCGTTCATACAGCGGAGATGATAACGACAGCATTCATTTGTCCCTTGCACGCGTTTCT
TCAGACATGCCCCTCGCGAGGAAGGTGTCGCTAAAGAGGTTCCTGGAGAAGAGGAAGAACCGGCTCACCGCGGCAGATCC
ATACCCAGCAGCGGCGGCGACGGCGGCGGCAAGCGAGTCGTCGACTAAGCCTCCGGCAGTGAAAGACGAGGGCGCGCCGT
GGGTCGGCGTGAACTCGGCGCTCAGCCTCAGCTGAACAATCTCATCGACGGCAGCCTGCCTACCTATCGCTATAGGGAAA
AACTAGCTATTTCCCGGCCGTAAAAATATGTACGTGTTTGGCCCCCCTTGACATTTACACCAGTAAAATGGCTGCTTCAG
TTCAGGGGAATTATTAGCTGGGTAGCTAATTACCATGATCGGTTTATTTAGTTGTCTGTTTGAGAATTCTTTGGCCAGAA

CGAAGAAGAATTCTTTTTTTCTTCCTTGCTGGTATTCATCATATCTGCGAGGATACGAACTGATTAATTAGACCTAGTCG
AACTCATATATATACATAGTGTGTAAATTCAAATAAGATGATGGAACAGGTTATATGCAATTCTTTCGCTTCA
>GRMZM5G838098_T01 cDNA
Chr1: 16782463..16783895
GACCGCACCTCGTATCGTCGTCTAGCTCCGTTTCGTTTCCGGGAAGCAACCAACCTTCGTTCCCGGGCAGCCGACGACCC
TTGCGCCGTCTTCGTCTGTCAAGAGTACAGTTTGACGCGAGCAGCTGCCGGTTCCTTCTCTCGTGCGCCTTTGCTAGCTT
GGGATCGATCAAAGCCGGTGAGCGACCGAGCGAGCGAGTGAGGATCGGAACATCTGCCATCATGGCCTCCGAGCATGCGC
AGCTGTCTGCCAAGGCCGCCGCCGGCAGCAGGTTCGCGGTGACGTACGGCCTGCTGAGGCAGTACATGAAGGAGCAGGGC
GGCAGCGGCGCCACCAGGAGCCTCGCGCCGGCCGTGGGCGTGGGCCTCATGCCGGAAGCCGACGCTGCTGCGGCGGCGGA
GGCGACCGAGGAGAGGACCACCGTGCTGGAGCTCTTCCCGCAGCAGGCTGGCACGCTCAAAGACGAGCAGCAAAGGTGTG
TTAACTCGCCGTTGCCTTCAGTCAGTGCCTCACCGTGCGCGTGTGAACTTCTGATCGCGGATCTCTCCGTCGTCCGTCGT
CCCCTTAGGAAGAGGAAGGAGCGTGCTGACGGGAGAGCGCCGCTGACCATCTTCTACGGCGGCAAGATGGTGGTCTTCGA
CGACTTCCCCGCCGAGAAGGCCGAGGAGCTGATGCAGCTCGCCGGCTCCGGCAACGCCGCCCAGGACGCGCTGGGACAGC
CCAGCCTCACAGGTACGAACACGAACGACTCAACGCATATACGTACGCTGTCCAGAGCTCACACGGGCAGCCTGCAGAGG
CCCATGTAGTAGCGTCCAGCAGCCGTTCATACAGCGGAGATGATAACGACAGCATTCATTTGTCCCTTGCACGCGTTTCT
TCAGACATGCCCCTCGCGAGGAAGGTGTCGCTAAAGAGGTTCCTGGAGAAGAGGAAGAACCGGCTCACCGCGGCAGATCC
ATACCCAGCAGCGGCGGCGACGGCGGCGGCAAGCGAGTCGTCGACTAAGCCTCCGGCAGTGAAAGACGAGGGCGCGCCGT
GGGTCGGCGTGAACTCGGCGCTCAGCCTCAGCTGAACAATCTCATCGACGGCAGCCTGCCTACCTATCGCTATAGGGAAA
AACTAGCTATTTCCCGGCCGTAAAAATATGTACGTGTTTGGCCCCCCTTGACATTTACACCAGTAAAATGGCTGCTTCAG
TTCAGGGGAATTATTAGCTGGGTAGCTAATTACCATGATCGGTTTATTTAGTTGTCTGTTTGAGAATTCTTTGGCCAGAA
CGAAGAAGAATTCTTTTTTTCTTCCTTGCTGGTATTCATCATATCTGCGAGGATACGAACTGATTAATTAGACCTAGTCG
AACTCATATATATACATAGTGTGTAAATTCAAATAAGATGATGGAACAGGTTATATGCAATTCTTTCGCTTCA
>GRMZM5G838098_P01 peptide
MASEHAQLSAKAAAGSRFAVTYGLLRQYMKEQGGSGATRSLAPAVGVGLMPEADAAAAAEATEERTTVLELFPQQAGTLK
DEQQRCVNSPLPSVSASPCACELLIADLSVVRRPLRKRKERADGRAPLTIFYGGKMVVFDDFPAEKAEELMQLAGSGNAA
QDALGQPSLTGTNTNDSTHIRTLSRAHTGSLQRPM
>GRMZM5G838098_T02
Chr1: 16782463..16783894
GACCGCACCTCGTATCGTCGTCTAGCTCCGTTTCGTTTCCGGGAAGCAACCAACCTTCGTTCCCGGGCAGCCGACGACCC
TTGCGCCGTCTTCGTCTGTCAAGAGTACAGTTTGACGCGAGCAGCTGCCGGTTCCTTCTCTCGTGCGCCTTTGCTAGCTT
GGGATCGATCAAAGCCGGTGAGCGACCGAGCGAGCGAGTGAGGATCGGAACATCTGCCATCATGGCCTCCGAGCATGCGC
AGCTGTCTGCCAAGGCCGCCGCCGGCAGCAGGTTCGCGGTGACGTACGGCCTGCTGAGGCAGTACATGAAGGAGCAGGGC
GGCAGCGGCGCCACCAGGAGCCTCGCGCCGGCCGTGGGCGTGGGCCTCATGCCGGAAGCCGACGCTGCTGCGGCGGCGGA
GGCGACCGAGGAGAGGACCACCGTGCTGGAGCTCTTCCCGCAGCAGGCTGGCACGCTCAAAGACGAGCAGCAAAGGTGTG
TTAACTCGCCGTTGCCTTCAGTCAGTGCCTCACCGTGCGCGTGTGAACTTCTGATCGCGGATCTCTCCGTCGTCCGTCGT
CCCCTTAGGAAGAGGAAGGAGCGTGCTGACGGGAGAGCGCCGCTGACCATCTTCTACGGCGGCAAGATGGTGGTCTTCGA
CGACTTCCCCGCCGAGAAGGCCGAGGAGCTGATGCAGCTCGCCGGCTCCGGCAACGCCGCCCAGGACGCGCTGGGACAGC
CCAGCCTCACAGACATGCCCCTCGCGAGGAAGGTGTCGCTAAAGAGGTTCCTGGAGAAGAGGAAGAACCGGCTCACCGCG
GCAGATCCATACCCAGCAGCGGCGGCGACGGCGGCGGCAAGCGAGTCGTCGACTAAGCCTCCGGCAGTGAAAGACGAGGG
CGCGCCGTGGGTCGGCGTGAACTCGGCGCTCAGCCTCAGCTGAACAATCTCATCGACGGCAGCCTGCCTACCTATCGCTA
TAGGGAAAAACTAGCTATTTCCCGGCCGTAAAAATATGTACGTGTTTGGCCCCCCTTGACATTTACACCAGTAAAATGGC
TGCTTCAGTTCAGGGGAATTATTAGCTGGGTAGCTAATTACCATGATCGGTTTATTTAGTTGTCTGTTTGAGAATTCTTT
GGCCAGAACGAAGAAGAATTCTTTTTTTCTTCCTTGCTGGTATTCATCATATCTGCGAGGATACGAACTGATTAATTAGA
CCTAGTCGAACTCATATATATACATAGTGTGTAAATTCAAATAAGATGATGGAACAGGTTATATGCAATTCTTTCGCTTC

>GRMZM5G838098_P02 peptide
MASEHAQLSAKAAAGSRFAVTYGLLRQYMKEQGGSGATRSLAPAVGVGLMPEADAAAAAEATEERTTVLELFPQQAGTLK
DEQQRCVNSPLPSVSASPCACELLIADLSVVRRPLRKRKERADGRAPLTIFYGGKMVVFDDFPAEKAEELMQLAGSGNAA
QDALGQPSLTDMPLARKVSLKRFLEKRKNRLTAADPYPAAAATAAASESSTKPPAVKDEGAPWVGVNSALSLS
>GRMZM5G838098_T03 cDNA
Chr1: 16782463..16783894
GACCGCACCTCGTATCGTCGTCTAGCTCCGTTTCGTTTCCGGGAAGCAACCAACCTTCGTTCCCGGGCAGCCGACGACCC
TTGCGCCGTCTTCGTCTGTCAAGAGTACAGTTTGACGCGAGCAGCTGCCGGTTCCTTCTCTCGTGCGCCTTTGCTAGCTT
GGGATCGATCAAAGCCGGTGAGCGACCGAGCGAGCGAGTGAGGATCGGAACATCTGCCATCATGGCCTCCGAGCATGCGC
AGCTGTCTGCCAAGGCCGCCGCCGGCAGCAGGTTCGCGGTGACGTACGGCCTGCTGAGGCAGTACATGAAGGAGCAGGGC
GGCAGCGGCGCCACCAGGAGCCTCGCGCCGGCCGTGGGCGTGGGCCTCATGCCGGAAGCCGACGCTGCTGCGGCGGCGGA
GGCGACCGAGGAGAGGACCACCGTGCTGGAGCTCTTCCCGCAGCAGGCTGGCACGCTCAAAGACGAGCAGCAAAGGAAGA
GGAAGGAGCGTGCTGACGGGAGAGCGCCGCTGACCATCTTCTACGGCGGCAAGATGGTGGTCTTCGACGACTTCCCCGCC
GAGAAGGCCGAGGAGCTGATGCAGCTCGCCGGCTCCGGCAACGCCGCCCAGGACGCGCTGGGACAGCCCAGCCTCACAGA
CATGCCCCTCGCGAGGAAGGTGTCGCTAAAGAGGTTCCTGGAGAAGAGGAAGAACCGGCTCACCGCGGCAGATCCATACC
CAGCAGCGGCGGCGACGGCGGCGGCAAGCGAGTCGTCGACTAAGCCTCCGGCAGTGAAAGACGAGGGCGCGCCGTGGGTC
GGCGTGAACTCGGCGCTCAGCCTCAGCTGAACAATCTCATCGACGGCAGCCTGCCTACCTATCGCTATAGGGAAAAACTA
GCTATTTCCCGGCCGTAAAAATATGTACGTGTTTGGCCCCCCTTGACATTTACACCAGTAAAATGGCTGCTTCAGTTCAG
GGGAATTATTAGCTGGGTAGCTAATTACCATGATCGGTTTATTTAGTTGTCTGTTTGAGAATTCTTTGGCCAGAACGAAG
AAGAATTCTTTTTTTCTTCCTTGCTGGTATTCATCATATCTGCGAGGATACGAACTGATTAATTAGACCTAGTCGAACTC
ATATATATACATAGTGTGTAAATTCAAATAAGATGATGGAACAGGTTATATGCAATTCTTTCGCTTC
>GRMZM5G838098_P03 peptide
MASEHAQLSAKAAAGSRFAVTYGLLRQYMKEQGGSGATRSLAPAVGVGLMPEADAAAAAEATEERTTVLELFPQQAGTLK
DEQQRKRKERADGRAPLTIFYGGKMVVFDDFPAEKAEELMQLAGSGNAAQDALGQPSLTDMPLARKVSLKRFLEKRKNRL
TAADPYPAAAATAAASESSTKPPAVKDEGAPWVGVNSALSLS

AGPv4:

Zm00001d027900 (zim27 - ZIM-transcription factor 27)
Chr1: 17147073..17148511

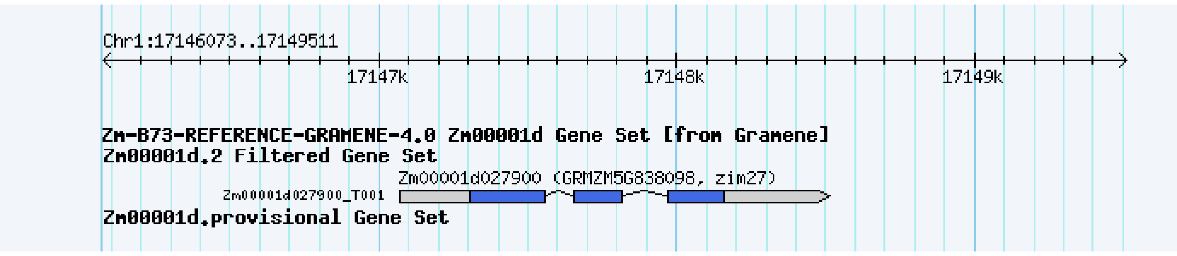

>Zm00001d027900 Genomic DNA
GCTATACCGCATGACCGCACCTCGTATCGTCGTCTAGCTCCGTTTCGTTTCCGGGAAGCAACCAACCTTCGTTCCCGGGC
AGCCGACGACCCTTGCGCCGTCTTCGTCTGTCAAGAGTACAGTTTGACGCGAGCAGCTGCCGGTTCCTTCTCTCGTGCGC
CTTTGCTAGCTTGGGATCGATCAAAGCCGGTGAGCGACCGAGCGAGCGAGTGAGGATCGGAACATCTGCCATCATGGCCT
CCGAGCATGCGCAGCTGTCTGCCAAGGCCGCCGCCGGCAGCAGGTTCGCGGTGACGTACGGCCTGCTGAGGCAGTACATG
AAGGAGCAGGGCGGCAGCGGCGCCACCAGGAGCCTCGCGCCGGCCGTGGGCGTGGGCCTCATGCCGGAAGCCGACGCTGC
TGCGGCGGCGGAGGCGACCGAGGAGAGGACCACCGTGCTGGAGCTCTTCCCGCAGCAGGCTGGCACGCTCAAAGACGAGC
AGCAAAGGTGTGTTAACTCGCCGTTGCCTTCAGTCAGTGCCTCACCGTGCGCGTGTGAACTTCTGATCGCGGATCTCTCC
GTCGTCCGTCGTCCCCTTAGGAAGAGGAAGGAGCGTGCTGACGGGAGAGCGCCGCTGACCATCTTCTACGGCGGCAAGAT
GGTGGTCTTCGACGACTTCCCCGCCGAGAAGGCCGAGGAGCTGATGCAGCTCGCCGGCTCCGGCAACGCCGCCCAGGACG

CGCTGGGACAGCCCAGCCTCACAGGTACGAACACGAACGACTCAACGCATATACGTACGCTGTCCAGAGCTCACACGGGC
AGCCTGCAGAGGCCCATGTAGTAGCGTCCAGCAGCCGTTCATACAGCGGAGATGATAACGACAGCATTCATTTGTCCCTT
GCACGCGTTTCTTCAGACATGCCCCTCGCGAGGAAGGTGTCGCTAAAGAGGTTCCTGGAGAAGAGGAAGAACCGGCTCAC
CGCGGCAGATCCATACCCAGCAGCGGCGGCGACGGCGGCGGCAAGCGAGTCGTCGACTAAGCCTCCGGCAGTGAAAGACG
AGGGCGCGCCGTGGGTCGGCGTGAACTCGGCGCTCAGCCTCAGCTGAACAATCTCATCGACGGCAGCCTGCCTACCTATC
GCTATAGGGAAAAACTAGCTATTTCCCGGCCGTAAAAATATGTACGTGTTTGGCCCCCCTTGACATTTACACCAGTAAAA
TGGCTGCTTCAGTTCAGGGGAATTATTAGCTGGGTAGCTAATTACCATGATCGGTTTATTTAGTTGTCTGTTTGAGAATT
CTTTGGCCAGAACGAAGAAGAATTCTTTTTTTCTTCCTTGCTGGTATTCATCATATCTGCGAGGATACGAACTGATTAAT
TAGACCTAGTCGAACTCATATATATACATAGTGTGTAAATTCAAATAAGATGATGGAACAGGTTATATGCAATTCTTTC
>Zm00001d027900_T001 cDNA
GCTATACCGCATGACCGCACCTCGTATCGTCGTCTAGCTCCGTTTCGTTTCCGGGAAGCAACCAACCTTCGTTCCCGGGC
AGCCGACGACCCTTGCGCCGTCTTCGTCTGTCAAGAGTACAGTTTGACGCGAGCAGCTGCCGGTTCCTTCTCTCGTGCGC
CTTTGCTAGCTTGGGATCGATCAAAGCCGGTGAGCGACCGAGCGAGCGAGTGAGGATCGGAACATCTGCCATCATGGCCT
CCGAGCATGCGCAGCTGTCTGCCAAGGCCGCCGCCGGCAGCAGGTTCGCGGTGACGTACGGCCTGCTGAGGCAGTACATG
AAGGAGCAGGGCGGCAGCGGCGCCACCAGGAGCCTCGCGCCGGCCGTGGGCGTGGGCCTCATGCCGGAAGCCGACGCTGC
TGCGGCGGCGGAGGCGACCGAGGAGAGGACCACCGTGCTGGAGCTCTTCCCGCAGCAGGCTGGCACGCTCAAAGACGAGC
AGCAAAGGAAGAGGAAGGAGCGTGCTGACGGGAGAGCGCCGCTGACCATCTTCTACGGCGGCAAGATGGTGGTCTTCGAC
GACTTCCCCGCCGAGAAGGCCGAGGAGCTGATGCAGCTCGCCGGCTCCGGCAACGCCGCCCAGGACGCGCTGGGACAGCC
CAGCCTCACAGACATGCCCCTCGCGAGGAAGGTGTCGCTAAAGAGGTTCCTGGAGAAGAGGAAGAACCGGCTCACCGCGG
CAGATCCATACCCAGCAGCGGCGGCGACGGCGGCGGCAAGCGAGTCGTCGACTAAGCCTCCGGCAGTGAAAGACGAGGGC
GCGCCGTGGGTCGGCGTGAACTCGGCGCTCAGCCTCAGCTGAACAATCTCATCGACGGCAGCCTGCCTACCTATCGCTAT
AGGGAAAAACTAGCTATTTCCCGGCCGTAAAAATATGTACGTGTTTGGCCCCCCTTGACATTTACACCAGTAAAATGGCT
GCTTCAGTTCAGGGGAATTATTAGCTGGGTAGCTAATTACCATGATCGGTTTATTTAGTTGTCTGTTTGAGAATTCTTTG
GCCAGAACGAAGAAGAATTCTTTTTTTCTTCCTTGCTGGTATTCATCATATCTGCGAGGATACGAACTGATTAATTAGAC
CTAGTCGAACTCATATATATACATAGTGTGTAAATTCAAATAAGATGATGGAACAGGTTATATGCAATTCTTTC
>Zm00001d027900_P001 peptide
MASEHAQLSAKAAAGSRFAVTYGLLRQYMKEQGGSGATRSLAPAVGVGLMPEADAAAAAEATEERTTVLELFPQQAGTLK
DEQQRKRKERADGRAPLTIFYGGKMVVFDDFPAEKAEELMQLAGSGNAAQDALGQPSLTDMPLARKVSLKRFLEKRKNRL
TAADPYPAAAATAAASESSTKPPAVKDEGAPWVGVNSALSLS

ZmJAZ28

B73 RefGen_v3:
AC197764.4_FG003 (zim30 - ZIM-transcription factor 30)
Chr5: 36853650..36854491

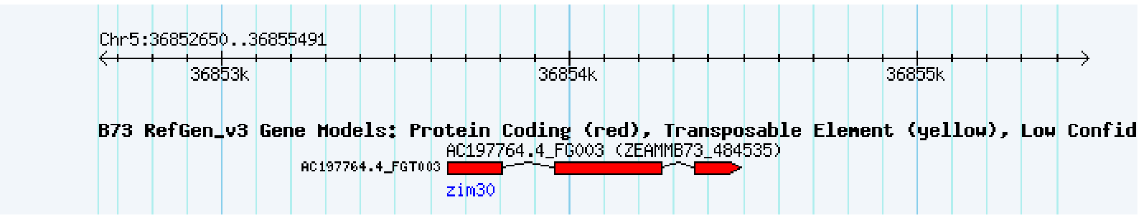

>AC197764.4_FG003 Genomic DNA
ATGTCGGCCGCCACTGGTTACAGCCGGTTCCTGCGGCAGTACATGAGAGAGCCGCAGCGGCATCAGCTGGTGGAGGCGGA
GGAGACGGACTCCGGCCGGACCATGCAGCTGTTCCCCACGCGCGCCGGCGGCGGCACGTCGTCGTCGTCGCATGAACAGT
GAGTACAGCAGTCTTCAATAATCTCCGTTAATTAGCTGGCTCTCTGTTTCTGCATGTATGTTCGAGGTACGGAGTGATGA

CGAGAGAGGTTCTTGCTGGCCATGCATGGCTGCTCTGGATTTGATCGCCGGCAGCGACTTCGTTTCAGGTCAGATGATGG
TGCGCAGGCGGCCAACAAGGCAACGCTGAGCATCTTCTACGAGGGCCGGATGCTCGTGTTCGAGGAGTTCCCGGCCGACA
AGGCCAAGGCGCTGATGCAGCTGGCCGCCGGGTCGTCGGGCTCCTCGTCGGCGGCGGCGCCCAATAACAAGGACGCCCCA
GTACCAGTGGTGCGGGTGCGGCGGGTGCCAGAGCAGCCGGCAGCCACCGCGCCATTGGCAGTCGTGCCCTCCGACCTGCT
GCCCATCGCGAGGAAAGTGTCGCTGCAGAGGTTCCTCCAGAAGAGGAAGGAGAGGTGATGAGGAGACATTATTCCATCTG
ATTCACGTCAACGTACAATTCATCTTATTCCATACTTCATCTGACTGACGTACGTGTGGATTGTCGCAGGATCGCCGCCA
CCGAGCCTTACCCGGAGCCGGAGAAAGATGATGTTCCTGCCACCGCCACCGCCACCGCCACCGGCTGGCAAGCGACGGCC
TTAAAGGATAAACCTGCACCTGCCTCATGGCTCGGGCTTTGA

>AC197764.4_FGT003_T001 cDNA

ATGTCGGCCGCCACTGGTTACAGCCGGTTCCTGCGGCAGTACATGAGAGAGCCGCAGCGGCATCAGCTGGTGGAGGCGGA
GGAGACGGACTCCGGCCGGACCATGCAGCTGTTCCCCACGCGCGCCGGCGGCGGCACGTCGTCGTCGTCGCATGAACAGT
CAGATGATGGTGCGCAGGCGGCCAACAAGGCAACGCTGAGCATCTTCTACGAGGGCCGGATGCTCGTGTTCGAGGAGTTC
CCGGCCGACAAGGCCAAGGCGCTGATGCAGCTGGCCGCCGGGTCGTCGGGCTCCTCGTCGGCGGCGGCGCCCAATAACAA
GGACGCCCCAGTACCAGTGGTGCGGGTGCGGCGGGTGCCAGAGCAGCCGGCAGCCACCGCGCCATTGGCAGTCGTGCCCT
CCGACCTGCTGCCCATCGCGAGGAAAGTGTCGCTGCAGAGGTTCCTCCAGAAGAGGAAGGAGAGGATCGCCGCCACCGAG
CCTTACCCGGAGCCGGAGAAAGATGATGTTCCTGCCACCGCCACCGCCACCGCCACCGGCTGGCAAGCGACGGCCTTAAA
GGATAAACCTGCACCTGCCTCATGGCTCGGGCTTTGA

AGPv4:

Zm00001d014250 (zim30 - ZIM-transcription factor 30)

Chr5: 38072928..38075939

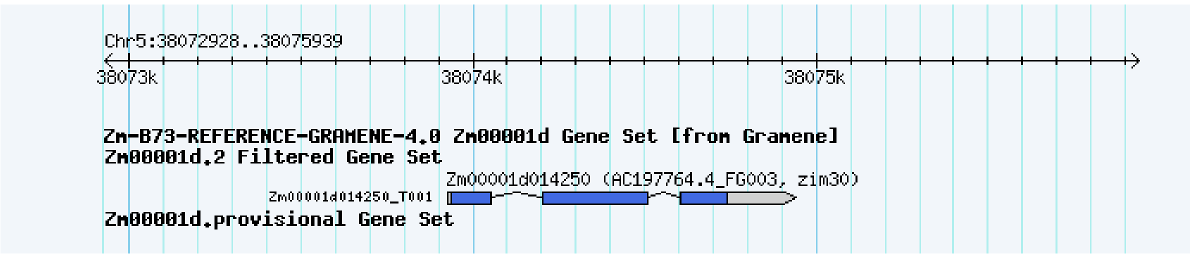

>Zm00001d014250 Genomic DNA

GCGGCAGTACATGAGAGAGCCGCAGCGGCATCAGCTGGTGGAGGCGGAGGAGACGGACTCCGGCCGGACCATGCAGCTGT
TCCCCACGCGCGCCGGCGGCGGCACGTCGTCGTCGTCGCATGAACAGTGAGTACAGCAGTCTTCAATAATCTCCGTTAAT
TAGCTGGCTCTCTGTTTCTGCATGTATGTTCGAGGTACGGAGTGATGACGAGAGAGGTTCTTGCTGGCCATGCATGGCTG
CTCTGGATTTGATCGCCGGCAGCGACTTCGTTTCAGGTCAGATGATGGTGCGCAGGCGGCCAACAAGGCAACGCTGAGCA
TCTTCTACGAGGGCCGGATGCTCGTGTTCGAGGAGTTCCCGGCCGACAAGGCCAAGGCGCTGATGCAGCTGGCCGCCGGG
TCGTCGGGCTCCTCGTCGGCGGCGGCGCCCAATAACAAGGACGCCCCAGTACCAGTGGTGCGGGTGCGGCGGGTGCCAGA
GCAGCCGGCAGCCACCGCGCCATTGGCAGTCGTGCCCTCCGACCTGCTGCCCATCGCGAGGAAAGTGTCGCTGCAGAGGT
TCCTCCAGAAGAGGAAGGAGAGGTGATGAGGAGACATTATTCCATCTGATTCACGTCAACGTACAATTCATCTTATTCCA
TACTTCATCTGACTGACGTACGTGTGGATTGTCGCAGGATCGCCGCCACCGAGCCTTACCCGGAGCCGGAGAAAGATGAT
GTTCCTGCCACCGCCACCGCCACCGCCACCGGCTGGCAAGCGACGGCCTTAAAGGATAAACCTGCACCTGCCTCATGGCT
CGGGCTTTGAAAATAATGATCCGATCATCTTGTTGTCTCCGTTTCAGAATTTTTCAGCGTTGCGAAGAATTCTTCTTCTT
CTTTTTTTTTTAAAAAAAAAGCAACAGTTAATTATCCGTTGGTCTATCGAGAGATTTGATGGATCATATATAATAACCCG
GCAGCTACATTTCTCCTGGACATCTATATGTATTGATTATTGTATGTTTTTT

>Zm00001d014250_T001 cDNA

GCGGCAGTACATGAGAGAGCCGCAGCGGCATCAGCTGGTGGAGGCGGAGGAGACGGACTCCGGCCGGACCATGCAGCTGT
TCCCCACGCGCGCCGGCGGCGGCACGTCGTCGTCGTCGCATGAACAGTCAGATGATGGTGCGCAGGCGGCCAACAAGGCA
ACGCTGAGCATCTTCTACGAGGGCCGGATGCTCGTGTTCGAGGAGTTCCCGGCCGACAAGGCCAAGGCGCTGATGCAGCT

GGCCGCCGGGTCGTCGGGCTCCTCGTCGGCGGCGGCGCCCAATAACAAGGACGCCCCAGTACCAGTGGTGCGGGTGCGGC
GGGTGCCAGAGCAGCCGGCAGCCACCGCGCCATTGGCAGTCGTGCCCTCCGACCTGCTGCCCATCGCGAGGAAAGTGTCG
CTGCAGAGGTTCCTCCAGAAGAGGAAGGAGAGGATCGCCGCCACCGAGCCTTACCCGGAGCCGGAGAAAGATGATGTTCC
TGCCACCGCCACCGCCACCGCCACCGGCTGGCAAGCGACGGCCTTAAAGGATAAACCTGCACCTGCCTCATGGCTCGGGC
TTTGAAAATAATGATCCGATCATCTTGTTGTCTCCGTTTCAGAATTTTTCAGCGTTGCGAAGAATTCTTCTTCTTCTTTT
TTTTTTAAAAAAAAAGCAACAGTTAATTATCCGTTGGTCTATCGAGAGATTTGATGGATCATATATAATAACCCGGCAGC
TACATTTCTCCTGGACATCTATATGTATTGATTATTGTATGTTTTTT
>Zm00001d014250_P001 peptide
MREPQRHQLVEAEETDSGRTMQLFPTRAGGGTSSSSHEQSDDGAQAANKATLSIFYEGRMLVFEEFPADKAKALMQLAAG
SSGSSSAAAPNNKDAPVPVVRVRRVPEQPAATAPLAVVPSDLLPIARKVSLQRFLQKRKERIAATEPYPEPEKDDVPATA
TATATGWQATALKDKPAPASWLGL

ZmJAZ29

AGPv4:
Zm00001d016316
Chr5: 156926728..156927201

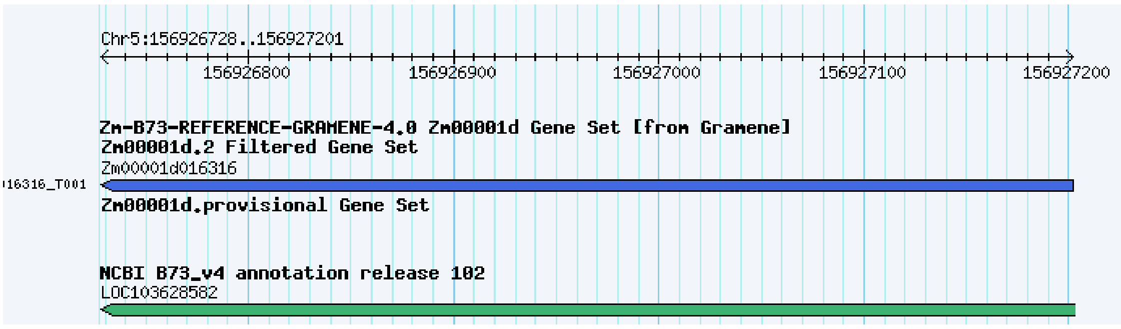

>Zm00001d016316 Genomic DNA
ATGGTGGTGGTGTTCGAGGACTTCCCGGCGGAGAAGGCGGCCAAGGTCATGCGCCTCGCGACCGACGACGACCTGCCCAT
CGCCCGGAAGGCGTCGCTGCAGCGGTTCCTGGCCAAGCGCAAGGACCGCCTCGTCGAGCGCGCGCCCTACGCCCGCCCGT
CGTCCCCTGCGGAGGAGCCAGAGAAGAAGACGGTCAAGCCGGCCTCGGCCTTGGCCTCCTGGCTCGGGCTCGGCAGCACG
GAAGCCGACCGCCTCACCATCGCGTTGTTCTGCCCACCCTCGACAGGTGGGCCCCATCTTCGAGCGTCTATTTCCTGCTG
CAGCTCACCCACAGAACCGCGCCCGCCGCCCCACTCTCAAGATTTTTGCCGTGCTCGCTACCTGGTCTGCCCCGCCGTCT
TCCCCAGATCGTGCGAGACCTGGGTCCACCCAGTCGCCACTCCCCCCCCCTCTCAACCTCCAAGCCCAGGTAA
>Zm00001d016316_T001 cDNA
ATGGTGGTGGTGTTCGAGGACTTCCCGGCGGAGAAGGCGGCCAAGGTCATGCGCCTCGCGACCGACGACGACCTGCCCAT
CGCCCGGAAGGCGTCGCTGCAGCGGTTCCTGGCCAAGCGCAAGGACCGCCTCGTCGAGCGCGCGCCCTACGCCCGCCCGT
CGTCCCCTGCGGAGGAGCCAGAGAAGAAGACGGTCAAGCCGGCCTCGGCCTTGGCCTCCTGGCTCGGGCTCGGCAGCACG
GAAGCCGACCGCCTCACCATCGCGTTGTTCTGCCCACCCTCGACAGGTGGGCCCCATCTTCGAGCGTCTATTTCCTGCTG
CAGCTCACCCACAGAACCGCGCCCGCCGCCCCACTCTCAAGATTTTTGCCGTGCTCGCTACCTGGTCTGCCCCGCCGTCT
TCCCCAGATCGTGCGAGACCTGGGTCCACCCAGTCGCCACTCCCCCCCCCTCTCAACCTCCAAGCCCAGGTAA
>Zm00001d016316_P001 peptide
MVVVFEDFPAEKAAKVMRLATDDDLPIARKASLQRFLAKRKDRLVERAPYARPSSPAEEPEKKTVKPASALASWLGLGST
EADRLTIALFCPPSTGGPHLRASISCCSSPTEPRPPPHSQDFCRARYLVCPAVFPRSCETWVHPVATPPPLSTSKPR

ZmJAZ30

AGPv4:

Zm00001d019692

Chr7: 51183119..51190112

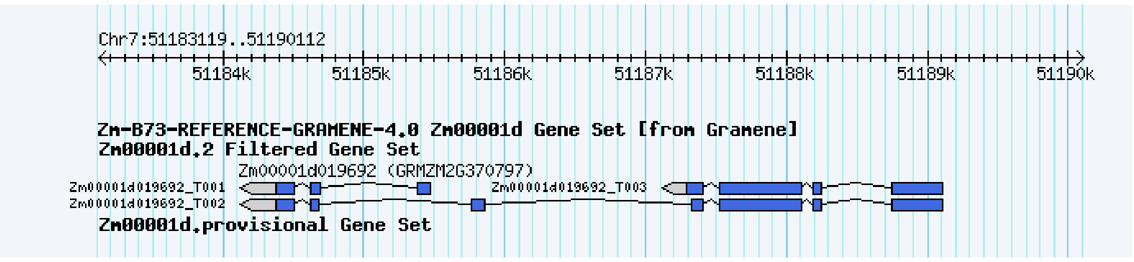

>Zm00001d019692 Genomic DNA

ATGTCGCGCGAGGGGGTGGTCCGGTGCAAGGACGCCACCGCAGCAGCAGATGAGCTCCCTGGGCTGCTGCGCTACAGGTC
GGCGCCGAGCATGCTGCTCGGCGAGGTGATGTGCGGGGATCAGGACTTCCCCGCGGCGGCTGGCGGCGAAGGGCACGGAC
CACCGGACCATGCCGCCACAGCCCACAGACAACATCCTCGCGCTCTTTCTCACCGGGCACCACTCAGAGGTCCAGGACTG
AAAGCCTCCCTTCCCCGCCGCGGCCGCTCACTTCATGGACGAAGCTGCCGCCTCGCAACAACAGTAGCAGTAGCTCATGT
ACCAGTTGCAACAGCAGCAGACAGAGATCGGCATGCGTGGTGGGGAAGCGCCCCCAAGATCCGACCAGCTTTCATCATTG
GATCGGGGCAGGCGCGACGCGGAGAGCAAGGACGTCGGGGGCGGCCATGGCGCAGTGGATCCATTGATGGAGTGAGGCAT
GCGCGGAGGACCCGTTTTGATAGCGCCATGCCAGGCGCGACCGCGCCACTCACCTTCCATCCACGATTTGCGGCAGGCAT
GGTGGGGACGCGTGACATATTTCTACTGGCACTTTAGAAGCAGTCCGTGTTTTCATCAAATTTGTTAGCTTGATTTAGCT
CTTCTGCAAACTGGACTATTTTGTGCTAATTTGTTAGCTTGATTTAGCTTTTCTGCATTTAGGTACTTTGCACATCTTCA
TGTTGAGCTACTGCTTCAACTGCATTTGATAATAGTACTCAGAGTTGATCTATTTCTTGCAGTGACTCAAGGATGTGGTG
TCTTGCTAGATGTTACTTGTTCTTATGAATGCAGCCTTTGATTTGACTTAGTTTGTACTTCTGTTTGCAGGTTATGCATG
ATGCTTTTGGAGTTCGGTTACATGCCGGAAAGCAGGCAGAGGTTTGCCACTGTCACCTAATACTCATATATGGCCTTGTG
TTTAAAGAGAACACAACATTTTCTGCAAAATTGCTATTTGGGTTTAGTTACATTGATAAGTTTTCTGCTATTGGCATAAA
AATATGTATATTTTCTTGGGTGATGTCTGCCCTTTCCTGGGTGGATGTGATGGCCGCGAGCTGTATGGATGATGAGAGAG
TGAGTGGGGAGGTCAGGGAGCAGGAAGACGACGACGACAAAGTGCCTCAACTCAGCGCTGCGGCGATGGAGGCGCTCCGG
GAGTTCCTGGCGGGACAGCATCGTCCGAAGGAGCAGAATGAAGCTGGAGGAGGGGAAGACGAGGTAGAGCTGGTCCCGGA
GGACTGGCGGTTGAGCCAGTTCTGGTACGATGAGCGTACCGCGCGGGAGCTCGTGGAGGAGGTCGTCCGCCTTGTCTCCC
CTTCTGGATCAGGCTCCGTCGCCGGTGTCATGGCCTGCATCGCATGCCTGACGCTCTATGCCTACCTGAAGAAGACGGAC
CCCGGAGTGCCCGCGCAGCTGTTGGAGTACGACGAGCGGTTCGGGCAGTACGGCTGCGACTTCACCTTCTACGACGACAA
CCGACCGGAGGAGCTGCCGGCGGCGATGAAGCACGCCTACCGAGTCATCGTTGCCGACCCTCCCTATCTGGTACTGTTTC
TACCCTACCAGAGATTGCCTATTAAAAAGTGTTTGATCAGTTGTTAACATTTGAGATGAGAGGAAATATTGAGGAGAAAT
GGCTATTATTCGTTGGTTGATGATGCAGAGTAAGGAGTGCTTGGAAAAGGTTGCCAAAACAGTATCTTTCCTCGCACGAC
CTGAAGGTTCATTCCTGCTACTACTCATAGGTAATTGTAGCTCATGGCAATGCTCTGTGGAGTGAAAGCGATTAATTTGA
TTTTAATTTGGTATTTTGATTTTGTTCTGCAATCAATAAGCTATAGTTTGGAATTCCTATATGATAGCTTCACGTTTATA
ATCTAGCCAAGAGTGTTGATAATGACTTCAACTTTTTCGAGCATGGTTAGTTTAATATTGTTTTGGCAATTTAGCAATAG
TGCAAATATCCTAGATGATAGTTTTGCCTTATAATCTAGCTAAGGGTGCTGGTGATGACTTCAAGTTTAATGCTGTGTCA
AGTGAACATGATTAGTTTGATATTGCTTTGATATTTTAATGTTGTTTATGCAATCAATAAACATTCTTGTTGAACTCCTA
TATTGTAGTTTCACCTTATAATCTAGCCAAGGGTGTTTATGATGCCTTAGACTTCATTGTGCCTTCTTACTTTTGACAGA
ATGATCCATTAATTAATGTATTAATAGCGCAGGAGTTGCACATTTACGATGGCATCATCTGCTTCTACATGTATGCAAAT
GTATATAATGTAGCTAAAACCATTTACGATGTGCTGACAGTTCCAAATTGTACTGTTGTTCCTTATCAAAGTCCGTTTGT
TTTCCTTTCAAGAAAAAAGGTCTCTCCATCTCATACTGAAAGCATAGTTAAACCCTGTATTTATCAAGCAAAAACTTAGT
TGGAGTCACTCTATGTTGCCAGGGGAAGTTCAGATGGACCGGGCATTGGAGCTGCTGAATGTTCGTCCCTGCGGTTTCAG
GCCTCGCCACTCGAACAAACTGGGCAACGAGTTCCGGCTGTTCACGAATTAAGATCCTGCTGACAGACTTGGTGGTTGGG

AGCAAAGTGATGGTGCCCCCATTTAACTATAAACTAATACAATTTTTGTTCTAGAGCTGTTCCATCTTTATGAACATGCT
GCATCTTTGATCCACAGCTCACTGATCCCTCCAATTTAAATTGTAGTGACAATTATTTGAGGAATCTGTGTGCTATGATG
TATGGTCAATCAAACATTTACACTGTTAGTGTACCTCATTTTAATTTCGTTTTAAAATACACTGTTAGTGTATGTTTCAT
TTTAATTTCATCAGTATTCTTTAGAATGTCTCGTTGGCTTATTCAGATTTATAGCCAAAATGACATTATCTTGGGATAGT
TCATCAGTATTTTTTAATTTCATTATCTCGTTCGGCGGCGGCATCCCGGACGGGCACAGCCTGCACACCGGCGGGCAGGT
CGTGAAGGCACCCACCATCGAGTAGAACCTGTCGTGCAACCTTGAGTAGCTCTACAAGGGCATCACCAAGAAGTTGAAGA
TTTCTAGGGAAATTGTTGACGCAAGTGGGTAAGTGCTTGCCTGCTCTTCTCATCTCGGTCCCAACATTGTTTATTTACTA
TGTTCATTGATAGTATTTGAAATTGAACTGATGACGCCTTGAAATTGCTTCAGCTAACCAGATGGACCAGTCCCTGTCAG
ATTTATGCTTGGTTAATTTCTGCTATTGGATTCAAGTTGAACTTGCATCAATGTATAGATCACTCAGTTGAATTGATTGA
TTTGTATTTGGCCATTTGGGTGTGCAACTGGGCCTCGGCTTCTCCAGATGTGATAGGTCAACGACAGAGGCACCCTCTTG
CTCCCATCGGCGGCGGCGGCGATGGCACCTCCTTCCATCTGCAACAACCCATGGAGGCCGACGACGGTGCATTTGGGGTT
TGGAACGACGGTCGCGTGGATGCCCCTACCCCTCACGTTTGTGCGGTCCACCCTGGCTCCCTCCCTGGATGCAGCTGGCG
CTCGCTGCGGGTGCTGGTGGACAAGCGATCGCTCGAGATGGCGGTGGAGGTGGATGCGAGGTGGGAGCCCATGGGGGCGC
TGGAGTACTACGACTACTGGCGGGCCATGTACAGCGCCCGCAGCAACAGGTGACCGCTCGGGATGGCAGATGATGCGGCT
GCAAGGTGACCGCTCGAGATCTATAAATATTGATGTTAGATGTGGTACTAACCTTAGCAAAAAGTAACTCTCAACACTCC
TCCCATTTCTCAACCACGTATAGGACAAAACAGTTAGTGGTATAAAATGGAAGTTATGGATGAATTGTTCTTTCTACACT
CATGCTTTTACTCCATGAATATTATAGGTAAATAGCTATTTTATTCTTTGAATTTATTTTATATTCTGCAGGAACTGTTA
GAAGTTTTCTGGAGAAGAAAAAGGACAAAATAGCTGGTGTTGTTTTTTTGTACTAGGCCATCATCTAACATATAGATATG
CAAACGATACTTAAACTGAACCCTCTACACAAACAGGAGGAAGAGATTGTGATAGTAAAGCTCCCTATTGTTGGGGATGA
GAATGGTGAGACGGCAATAGATGGAAGGAAAATAAGAATAAGACATTTGCCTGCTGGTGTGGTAAATAGATAGTGTCTGC
AACTATTGTTACTCTTCCTCTTTTCTGATTCTATAGAGGGAGCAGGTGCAGAGGGTTGAGGCAAAACAACGTTCTGTACC
AATGTCTCTGGTAAATACTCCTCTCTATGTGTTTTTATCCTTGGTTGAAGTTTTAAAATGTGATTGGTGAACTTTGCCAC
ACCGATTATTAATCAAGACATGTCTTCTCTTTTGTAGATATGCCAATTACGAGGAAGGCTTCTCTTCACCGCTTCCTTGA
GAAGAGAAAGGACCGGTAAGTTGTGGTTGTTGTGATTGTTATTTCAGTGTGGCAATTTGTTTGTTTTTTTCTCTCTCTC
AGTGTTCTGTTTGGAACTAATTTGTGCCCTTTTGATGTTCTTCAGTCTCAATGCAAATGGACCATACCAAACTTCTCCTT
CAGACGCTGCACCAGTCAAGAAGGAGCCCGAGAGCCAGGCATGGCTCGGATTAGGACCGAATGCCGTCAAGTCCAACCTG
AACCTAAGCTAGCACACAATGACACAGCCAAATAAGACGGCACAGCAACCTCACCAAAGCCTTTGCAGGACGATCTGAAA
ATATGCTTTGCCACTAGAGTACCGAAACCATAAATATCCTCTCGTTATCGTGTTTTAGTGTTCTGTTTAGGCCGTGGTCC
TACGCCCTCTTGTCACATAGTTCTGGATGTAAAGATAAGTAGCCAGCCATGATCGAGAGAAGTTTTTTTTGGGGGTGTTA
ACACACGGCCACTCTGAATTGGCCATGCTAAGGA
>Zm00001d019692_T001 cDNA
Chr7: 51184119..51185475
ATGGCGGTGGAGGTGGATGCGAGGTGGGAGCCCATGGGGGCGCTGGAGTACTACGACTACTGGCGGGCCATGTACAGCGC
CCGCAGCAACAGACATGTCTTCTCTTTTGTAGATATGCCAATTACGAGGAAGGCTTCTCTTCACCGCTTCCTTGAGAAGA
GAAAGGACCGTCTCAATGCAAATGGACCATACCAAACTTCTCCTTCAGACGCTGCACCAGTCAAGAAGGAGCCCGAGAGC
CAGGCATGGCTCGGATTAGGACCGAATGCCGTCAAGTCCAACCTGAACCTAAGCTAGCACACAATGACACAGCCAAATAA
GACGGCACAGCAACCTCACCAAAGCCTTTGCAGGACGATCTGAAAATATGCTTTGCCACTAGAGTACCGAAACCATAAAT
ATCCTCTCGTTATCGTGTTTTAGTGTTCTGTTTAGGCCGTGGTCCTACGCCCTCTTGTCACATAGTTCTGGATGTAAAGA
TAAGTAGCCAGCCATGATCGAGAGAAGTTTTTTTTGGGGGTGTTAACACACGGCCACTCTGAATTGGCCATGCTAAGGA
>Zm00001d019692_P001 peptide
MAVEVDARWEPMGALEYYDYWRAMYSARSNRHVFSFVDMPITRKASLHRFLEKRKDRLNANGPYQTSPSDAAPVKKEPES
QAWLGLGPNAVKSNLNLS
>Zm00001d019692_T002 cDNA
Chr7: 51184119..51189112

ATGTCGCGCGAGGGGGTGGTCCGGTGCAAGGACGCCACCGCAGCAGCAGATGAGCTCCCTGGGCTGCTGCGCTACAGGTC
GGCGCCGAGCATGCTGCTCGGCGAGGTGATGTGCGGGGATCAGGACTTCCCCGCGGCGGCTGGCGGCGAAGGGCACGGAC
CACCGGACCATGCCGCCACAGCCCACAGACAACATCCTCGCGCTCTTTCTCACCGGGCACCACTCAGAGGTCCAGGACTG
AAAGCCTCCCTTCCCCGCCGCGGCCGCTCACTTCATGGACGAAGCTGCCGCCTCGCAACAACAGTAGCAGTAGCTCATGT
ACCAGTTGCAACAGCAGCAGACAGAGATCGGCATGCGTGGTTATGCATGATGCTTTTGGAGTTCGGTTACATGCCGGAAA
GCAGGCAGAGTTACATTGATAAGTTTTCTGCTATTGGCATAAAAATATGTATATTTTCTTGGGTGATGTCTGCCCTTTCC
TGGGTGGATGTGATGGCCGCGAGCTGTATGGATGATGAGAGAGTGAGTGGGGAGGTCAGGGAGCAGGAAGACGACGACGA
CAAAGTGCCTCAACTCAGCGCTGCGGCGATGGAGGCGCTCCGGGAGTTCCTGGCGGGACAGCATCGTCCGAAGGAGCAGA
ATGAAGCTGGAGGAGGGGAAGACGAGGTAGAGCTGGTCCCGGAGGACTGGCGGTTGAGCCAGTTCTGGTACGATGAGCGT
ACCGCGCGGGAGCTCGTGGAGGAGGTCGTCCGCCTTGTCTCCCCTTCTGGATCAGGCTCCGTCGCCGGTGTCATGGCCTG
CATCGCATGCCTGACGCTCTATGCCTACCTGAAGAAGACGGACCCCGGAGTGCCCGCGCAGCTGTTGGAGTACGACGAGC
GGTTCGGGCAGTACGGCTGCGACTTCACCTTCTACGACGACAACCGACCGGAGGAGCTGCCGGCGGCGATGAAGCACGCC
TACCGAGTCATCGTTGCCGACCCTCCCTATCTGAGTAAGGAGTGCTTGGAAAAGGTTGCCAAAACAGTATCTTTCCTCGC
ACGACCTGAAGGTTCATTCCTGCTACTACTCATAGCTAACCAGATGGACCAGTCCCTGTCAGATTTATGCTTGGTTAATT
TCTGCTATTGGATTCAAGTTGAACTTGCATCAATGTATAGATCACTCAATATGCCAATTACGAGGAAGGCTTCTCTTCAC
CGCTTCCTTGAGAAGAGAAAGGACCGTCTCAATGCAAATGGACCATACCAAACTTCTCCTTCAGACGCTGCACCAGTCAA
GAAGGAGCCCGAGAGCCAGGCATGGCTCGGATTAGGACCGAATGCCGTCAAGTCCAACCTGAACCTAAGCTAGCACACAA
TGACACAGCCAAATAAGACGGCACAGCAACCTCACCAAAGCCTTTGCAGGACGATCTGAAAATATGCTTTGCCACTAGAG
TACCGAAACCATAAATATCCTCTCGTTATCGTGTTTTAGTGTTCTGTTTAGGCCGTGGTCCTACGCCCTCTTGTCACATA
GTTCTGGATGTAAAGATAAGTAGCCAGCCATGATCGAGAGAAGTTTTTTTGGGGGTGTTAACACACGGCCACTCTGAAT
TGGCCATGCTAAGGA

>Zm00001d019692_P002 peptide

MSREGVVRCKDATAAADELPGLLRYRSAPSMLLGEVMCGDQDFPAAAGGEGHGPPDHAATAHRQHPRALSHRAPLRGPGL
KASLPRRGRSLHGRSCRLATTVAVAHVPVATAADRDRHAWLCMMLLEFGYMPESRQSYIDKFSAIGIKICIFSWVMSALS
WVDVMAASCMDDERVSGEVREQEDDDDKVPQLSAAAMEALREFLAGQHRPKEQNEAGGGEDEVELVPEDWRLSQFWYDER
TARELVEEVVRLVSPSGSGSVAGVMACIACLTLYAYLKKTDPGVPAQLLEYDERFGQYGCDFTFYDDNRPEELPAAMKHA
YRVIVADPPYLSKECLEKVAKTVSFLARPEGSFLLLLIANQMDQSLSDLCLVNFCYWIQVELASMYRSLNMPITRKASLH
RFLEKRKDRLNANGPYQTSPSDAAPVKKEPESQAWLGLGPNAVKSNLNLS

>Zm00001d019692_T003 cDNA

Chr7: 51187117..51189112

ATGTCGCGCGAGGGGGTGGTCCGGTGCAAGGACGCCACCGCAGCAGCAGATGAGCTCCCTGGGCTGCTGCGCTACAGGTC
GGCGCCGAGCATGCTGCTCGGCGAGGTGATGTGCGGGGATCAGGACTTCCCCGCGGCGGCTGGCGGCGAAGGGCACGGAC
CACCGGACCATGCCGCCACAGCCCACAGACAACATCCTCGCGCTCTTTCTCACCGGGCACCACTCAGAGGTCCAGGACTG
AAAGCCTCCCTTCCCCGCCGCGGCCGCTCACTTCATGGACGAAGCTGCCGCCTCGCAACAACAGTAGCAGTAGCTCATGT
ACCAGTTGCAACAGCAGCAGACAGAGATCGGCATGCGTGGTTATGCATGATGCTTTTGGAGTTCGGTTACATGCCGGAAA
GCAGGCAGAGTTACATTGATAAGTTTTCTGCTATTGGCATAAAAATATGTATATTTTCTTGGGTGATGTCTGCCCTTTCC
TGGGTGGATGTGATGGCCGCGAGCTGTATGGATGATGAGAGAGTGAGTGGGGAGGTCAGGGAGCAGGAAGACGACGACGA
CAAAGTGCCTCAACTCAGCGCTGCGGCGATGGAGGCGCTCCGGGAGTTCCTGGCGGGACAGCATCGTCCGAAGGAGCAGA
ATGAAGCTGGAGGAGGGGAAGACGAGGTAGAGCTGGTCCCGGAGGACTGGCGGTTGAGCCAGTTCTGGTACGATGAGCGT
ACCGCGCGGGAGCTCGTGGAGGAGGTCGTCCGCCTTGTCTCCCCTTCTGGATCAGGCTCCGTCGCCGGTGTCATGGCCTG
CATCGCATGCCTGACGCTCTATGCCTACCTGAAGAAGACGGACCCCGGAGTGCCCGCGCAGCTGTTGGAGTACGACGAGC
GGTTCGGGCAGTACGGCTGCGACTTCACCTTCTACGACGACAACCGACCGGAGGAGCTGCCGGCGGCGATGAAGCACGCC
TACCGAGTCATCGTTGCCGACCCTCCCTATCTGAGTAAGGAGTGCTTGGAAAAGGTTGCCAAAACAGTATCTTTCCTCGC
ACGACCTGAAGGTTCATTCCTGCTACTACTCATAGGTAATTGTAGCTCATGGCAATGCTCTGTGGAGTGAAAGCGATTAA

TTTGATTTTAATTTGGTATTTTGATTTTGTTCTGCAATCAATAAGCTATAGTTTGGAATTCCTATATGATAGCTTCACGT
TTATAATCTAGCCAAGAGTGTTGATAATGACTTCAACTTTTTCGAGCATGGTTAGTTTAATATTGTTTTGGCAATTTAGC
A
>Zm00001d019692_P003 peptide
MSREGVVRCKDATAAADELPGLLRYRSAPSMLLGEVMCGDQDFPAAAGGEGHGPPDHAATAHRQHPRALSHRAPLRGPGL
KASLPRRGRSLHGRSCRLATTVAVAHVPVATAADRDRHAWLCMMLLEFGYMPESRQSYIDKFSAIGIKICIFSWVMSALS
WVDVMAASCMDDERVSGEVREQEDDDDKVPQLSAAAMEALREFLAGQHRPKEQNEAGGGEDEVELVPEDWRLSQFWYDER
TARELVEEVVRLVSPSGSGSVAGVMACIACLTLYAYLKKTDPGVPAQLLEYDERFGQYGCDFTFYDDNRPEELPAAMKHA
YRVIVADPPYLSKECLEKVAKTVSFLARPEGSFLLLLIGNCSSWQCSVE

ZmJAZ31

Zm00001d021924
AGPv4:
Chr7: 165961049..165962395

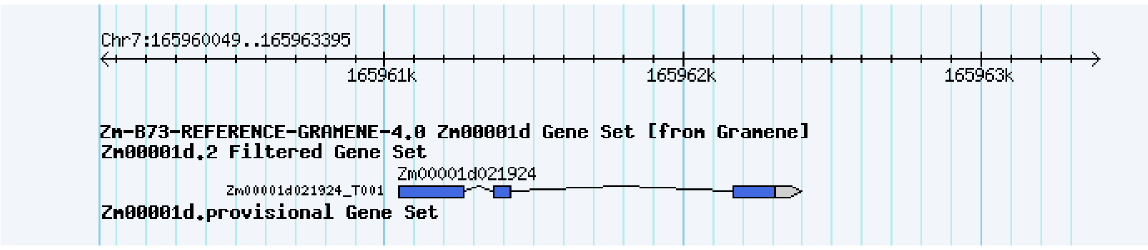

>Zm00001d021924 Genomic DNA
ATGCTTATAGCTGCAGCTGCGGCAGCTGCGGCAGCCACCAAAGGCAGTGCTGCCACTGCTTTTAATCCTCCAATGGTACA
TACAGCCACTGTCGCCCCAGCAGCAGTCTTCTCTCCTGTGCTTACACGGTCTCCATCACTGCAGAGCACTTCTGTAGCAG
CTGGACAAGCTCAGGTTGTTGCTGACCCTAGCTCAATAAGCAAGCTTCAGGCTGGTAAGACCACCGCATCTTTGTGTAAA
AATCCAGAAATGGAATACCTTTTCCAGTGTTGACATTGTCCAACACGTCTTTAAACTATCTTGATCTGTTGGCCAGATCT
CCCCATTGCCAGGAGGCACTCTCTCCAGCGCTTCCTTGAGAAACGTCGTGACAGGTTAGCATGATACATGAGAGCAACAG
GGCTCTGATATTCTGAATCTTATTGTTAGGCCTCTTATTCTACAATCTACATGTTTAAAACTTTTGTTATAAACTGCTGT
ATGTGGTACCACACTAATTTTTTCCCCGACTATTGGCACCACAGTAATCAACAGTATATTTTGTACCGTATTCTGTTTTT
TGTCCTCCAGGCAGGGTAAAAATAAAATAAAATTTGCTGTTATAGCATTTAAATGATTGCACTTTCTGCTGACAAGTGCT
CAATGACATCCCTAAAAAAATCTATAATTTCTTCACAGAAAGATTGGTAGAGAGCCTGCTTCCATAATACAACATATAGC
TGCAAATAGCCTTGGATCATGTAACTCCCTTGAGATTTTTTTAAAAAATCTGCAAATCGATATTAACACAAGACAGAGT
TCTGGGACCAGCAGTAGATACAAAAACCAACATTACCCCCATTTAGCTGCTCCAAATACCATGTTTTTCCCCTGTTAGGA
AAATAACATATCTGAGAATCGGGATGTTCCTGTGTATTATCCTGTTCTATTTGGAAAAGCTGGCCACTTAAAGATCCAGT
CTCAAGGTTGCAAACTAGCACCATTTTTTTCTGATGTAAGCTTTAGATGCCTGTTTTTGCCTTATCTTAGAACTGAGTTT
AGAATTGTTTCTTAGTATAACTTTTTTTATCGTGGATCTACTGATTTCAAGTGATTAAATAAATGATGGCACGAAACAGG
GTCGTGAGCAAAGCTCCGTACAGCCCCGCCAAGTCGTTCGATGGCATGGAGTCAGCGGGAATGGAGATGACTGTGGACGG
CAAGCAGGGCGCAAGGCCCAGTATCTTGAAAGATTGGGGAGCCGCGAAGTCTGAAGTCTGAACGGTGAAAGCGTAGCCCC
GCGGGAGCGTGGCGTTGTCTGGGAAGCGCCAGAAGCCGAGGTAGAGGGTGCAGAGGGAGGCAAGGTG
>Zm00001d021924_T001 cDNA
ATGCTTATAGCTGCAGCTGCGGCAGCTGCGGCAGCCACCAAAGGCAGTGCTGCCACTGCTTTTAATCCTCCAATGGTACA
TACAGCCACTGTCGCCCCAGCAGCAGTCTTCTCTCCTGTGCTTACACGGTCTCCATCACTGCAGAGCACTTCTGTAGCAG
CTGGACAAGCTCAGGTTGTTGCTGACCCTAGCTCAATAAGCAAGCTTCAGGCTGATCTCCCCATTGCCAGGAGGCACTCT
CTCCAGCGCTTCCTTGAGAAACGTCGTGACAGGGTCGTGAGCAAAGCTCCGTACAGCCCCGCCAAGTCGTTCGATGGCAT

GGAGTCAGCGGGAATGGAGATGACTGTGGACGGCAAGCAGGGCGCAAGGCCCAGTATCTTGAAAGATTGGGGAGCCGCGA
AGTCTGAAGTCTGAACGGTGAAAGCGTAGCCCCGCGGGAGCGTGGCGTTGTCTGGGAAGCGCCAGAAGCCGAGGTAGAGG
GTGCAGAGGGAGGCAAGGTG
>Zm00001d021924_P001 peptide
MLIAAAAAAAAATKGSAATAFNPPMVHTATVAPAAVFSPVLTRSPSLQSTSVAAGQAQVVADPSSISKLQADLPIARRHS
LQRFLEKRRDRVVSKAPYSPAKSFDGMESAGMEMTVDGKQGARPSILKDWGAAKSEV

ZmJAZ32

B73 RefGen_v3:

GRMZM2G442458

Chr10: 72558238..7256185

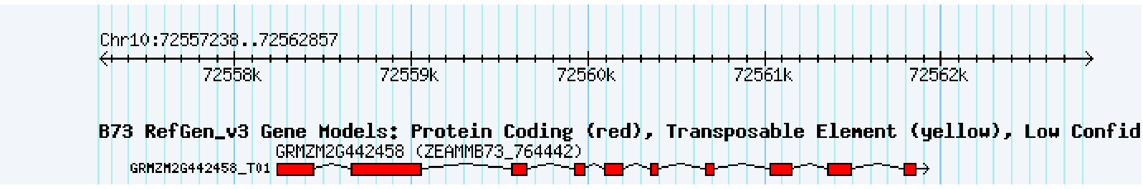

>GRMZM2G442458 Genomic DNA
ATGGCACCGATTGTGTTCCGGTTTAACCTGCGCAACACAGTTGATATCTTTGTTGTGTTCTTCGGCGGCTCCAGCCGCGC
GGCATGGGTGGGGTGGGATGCTTGGCATGCGGGCCAGTGGGACAATGTTCTTCTCGTCCATCTTCGGGACCGGTGCTCTA
CGTTCGGTGGCGGCATCCCGGATGGGCACAGCCTGCACACAGGCGGGCAGGTCGTGAAGGCACCCACCATCGAGCAGAAC
TTGTCATGCAACCTGGAGTAGCTGTACAAGGGCATCACCAAGAAGTTGAAGATTTCTAGGGAAATTGTTGACGCAAGTGG
GTAAGTGCTTGCCTGCTCTTCTCATCTCGGTCCCAACATTGTTTATTTACTCTGTTCATTGATAGTATTTGAAATTGAAC
TGATGACGCCTTGAAATTGCTTCAGCTAACCAGATGGACCAGTCCCTGTCAGATTTATGCTTGGTTAATTTCTTCTATTG
GATTCAAGTTGAACTTGCATCAATGTATAGATCACTCAGTTGAATTGATTGATTTGTATTTGGCCATTTGGGTGTGCAAC
TGGGCCTCGGCTTCTCCAGATGTGGTGGGTCAACGACAGAGACACCCTCTTGCTCCCATCGGCGGCAGCGGCGATGGCAC
CTCCTTCCATCTGCAACAACCCATGGAGGCCGACGATGGTGCATTTGGGGTTTGGAACGACGGTCGCGTGGATGCCCCTA
CCCCTCATGGATGTGCGGTCCACCCTGGCTCCCTCCCTGGATGCAGCTGGCGCTCGCTGCAGGTGCCGGTGGACAAGCGA
TCGCTCGAGATGGCGGTGTAGGTGGATGCGAGGTGGGAGCCCACGGGGGCGCTGGAGTACTACGACTACTGGCGGGCCAT
GTACAGCGCCCGCAACAACAGGTGACCGCTCGGGATGGCAGAGGATGCGGCTGCAAGGTGACCGCTCGAGATCTATAAAT
ATTGATGTTAGATGTGGTACTAACCTTAGCAAAAAGTAACTCTCAACACTCCTCCCATTTCTCAACCACGTATAGGACAA
AACAGTTAGTGGTATAAAATGGAAGTTATGGATGAATTGTTCTTTCTACACTCATGTTTTTACTCCATGAATATTATAGG
TAAATAGCTATTTTTATCTTTGAATTTATTTTATATTCTGCGGGAACTGTTAGAAGTTTTCTGGAGAAGAAAAAGGACAA
AATAGCTGGTGTTGTTTTTTTGTACTAGGCCATCATCTAACATATAGATATGCAAACGATACTTAAACTGAACCCTAGAA
TCTGTATTACCTTTTAGCTGTTCATCAGCTTCTTATAAGCAATTAAACAATGCAGATTGCTCCCACTGTATTTCCCTCTA
GACAAACAGGAGGAAGAGATTGTGATACTAAAGCTCCCTATTGTTGGGGATGAGAATGGTGAGACGGCAATAGATGGAAG
GAAAATAAGAATAAGACATTTGCCTGCTGGTGTGGTAGATAGAATAGTGTCTGCAACTATTGTTACTCTTCCTCTTTTCT
AATTCTATAGAGGGAGCAGGTGCAGAAGGTTGAGGCAAAACAACATTCTGTACCAATGTCTCTGGTAAATACTCCTCTCT
CTGTGTTTTTTTCCTTGGTTGAAGTTTTAAAATGTGATTGGTGAACTTTGCCACACCGATTATTAATCAAGACATGTCTT
CTCTTTTGTAGATATGCCAATTATGAGGAAGGCTTCTCTTCACCGCTTCCTTGAGAAGAGAAAGGACCGGTAAGTTGTGG
TTGTTGTTATTGTTATTTTCAGTGTGGTGATTTGTTTGTTTTTTCTCTCTCTCAGTGTTCTGTTTGGAACTAATTTGTGC
CCTTTTGATGTTCTTCAGTCTCAATGCAAATGGACCATACCAAACTTCTCCTTCAGACGCTGCACCAGTCAAGAAGGAGC
CCGAGAGCCAGGCATGGCTCGGATTAGGACCGAATGCCGTCAAGTCCAACCTGAACCTAAGCTAGCACACAACGACACAG
CCAAATAAGACGGCACAGCAACCTCACCAAAGCCTTTGCAGGACGATCTAAAAATATGCTTTGCCACTAGAGTACCGAAA

CCATAAATATTCTCTCGTTATCGTGTTTTGTGTTATGTTTAGGTCGTGGTCCTATGCCCTCTTGTCACAGTTCTGGATGT
AAAGATAAGTAGCCAGCCATGACCAAGAGAAGTTTTTTTTGGGGGGGTGTTAACACACGGCCACTCTGAATTGGCCATG
CTAAGGAAAATGATAGTAGTGCAAAGAGAAGTATATGTCTTTTTCTTTTTCTTTTTCTGAAAGCCAGATCATTTGGCTC
TAGCCTTGATGTATGCAGAACAAGTGATTGTAATGGATTCTGAGTATCCTCATCGTCTTGCTGTTGCTTCACCAACCCAA
GTTTGTTTGGATTCTTGCCATTCCTGCTGCAGCAAATCTCTGGTAGTTTATGGTAGCAACTCGAGCAATCTAAGGTAAAA
AAATAGAGGGAGAATTTATTATATCATTTTTGTTCATATTAATTAGCTTGAATTTTATTTTTATGTTCCTATTTGTGCAG
GTAAATATATTTAAGTTGTTATCATTCTTGTTTTCTGAAAGAAATATCCTTATTTAGTGAGATGATTGTTCTTTTTCTAG
CGATACATTTGCCGTCTAAGTCATTTGTTACAAGTTCTTTATCTCATTGTTGTTTGATTTACATGGTCTCTTGTATTGAC
TACATTTAGTTGTAGATCTTTATCTTCCAATATTTGTTCTTTGACCCACTTTCTCTGGTTATTCTACAGGGGTTATTTCA
AATCGGATTCATACTTGGATCCTTTGTTTATGTCAATAATTAAAGATCCTGATCTATGGTGCAAGGAGATGGGAAAATTT
GCTCAAGCTCAAAAGGGATTTAGTTATGCTAGGTAATTGGATATGGGGATCTAGGTTGCCATTCATTATCTGCAGCATAG
GAATACTCAATTCATTTACGATACCTCGCTAAATCAAATTCTCTTAATCTTTTAGAGATTGCTGAAATTATGTTATGATC
TTATACTTCTTGTTTGTGCTTAATTATGTTTATGCGAAGGTATATTTAATGACATGTGCTCCAATGACTTCATTGCCTAC
AGACTGATATTTTCTAGCAACTACAATTATCGTGGAGTTGATATTGAAGGACACCCGATTATGGTGGTTGTTGGTGCACA
CTTCCTTCTTCAATGCCTTGATCTTGAACGGTTTGTTCTACATGTAGTGAGGGTACGATAATGTACCTTGATATCCTAAA
GCATTGGATCAAGTGTCAGTATGCACTTTATAATCATTTGGGTGTGTATATCATGTACATTTGTATATCAAGTGTCCTGC
TGCCCCATGCTCTAGAAATTTTTTAGAGCAGGTCATGTACATTTTGTAGATCGACTTCAGTGCTGGCTTCTTAGGTGCCT
TGCCTTGATGCTTTGGCGAGATGTGTGCTTGGAAAGCTGTAGGTGCATTGTCCGGTGCAACATGTAGTAGCTATTTTGTT
GTCCTCATCGGTAGTTGCTTGTTTGTTTTCTTAGGTGAATATTCAAAAAGATAATGGGGTGCAGATGAAGATTATTGGTA
CTCATGTCGATGCTACATAA

>GRMZM2G442458_T01 cDNA

ATGGCACCGATTGTGTTCCGGTTTAACCTGCGCAACACAGTTGATATCTTTGTTGTGTTCTTCGGCGGCTCCAGCCGCGC
GGCATGGGTGGGGTGGGATGCTTGGCATGCGGGCCAGTGGGACAATGTTCTTCTCGTCCATCTTCGGGACCGGTGCTCTA
CGTTCGGTGGCGGCATCCCGGATGGGCACAGCCTGCACACAGGCGGGCAGCTAACCAGATGGACCAGTCCCTGTCAGATT
TATGCTTGGTTAATTTCTTCTATTGGATTCAAGTTGAACTTGCATCAATGTATAGATCACTCAGTTGAATTGATTGATTT
GTATTTGGCCATTTGGGTGTGCAACTGGGCCTCGGCTTCTCCAGATGTGGTGGGTCAACGACAGAGACACCCTCTTGCTC
CCATCGGCGGCAGCGGCGATGGCACCTCCTTCCATCTGCAACAACCCATGGAGGCCGACGATGGTGCATTTGGGGTTTGG
AACGACGGTCGCGTGGATGCCCCTACCCCTCATGGATGTGCGGTCCACCCTGGCTCCCTCCCTGGATGCAGCTGGCGCTC
GCTGCAGGTGCCGGTGGACAAGCGATCGCTCGAGATGGCGGTATTGCTCCCACTGTATTTCCCTCTAGACAAACAGGAGG
AAGAGATTGTGATACTAAAGCTCCCTATTGTTGGGGATGAGAATGATATGCCAATTATGAGGAAGGCTTCTCTTCACCGC
TTCCTTGAGAAGAGAAAGGACCGTCTCAATGCAAATGGACCATACCAAACTTCTCCTTCAGACGCTGCACCAGTCAAGAA
GGAGCCCGAGAGCCAGGCATGGCTCGGATTAGGACCGAATGCCGTCAAGTCGTGGTCCTATGCCCTCTTGTCACAGTTCT
GGATCAAATCTCTGGTAGTTTATGGTAGCAACTCGAGCAATCTAAGGGGTTATTTCAAATCGGATTCATACTTGGATCCT
TTGTTTATGTCAATAATTAAAGATCCTGATCTATGGTGCAAGGAGATGGGAAAATTTGCTCAAGCTCAAAAGGGATTTAG
TTATGCTAGACTGATATTTTCTAGCAACTACAATTATCGTGGAGTTGATATTGAAGGACACCCGATTATGGTGGTTGTTG
GTGCACACTTCCTTCTTCAATGCCTTGATCTTGAACGGTTTGTTCTACATGTAGTGAGGGTGAATATTCAAAAAGATAAT
GGGGTGCAGATGAAGATTATTGGTACTCATGTCGATGCTACATAA

>GRMZM2G442458_P01 peptide

MAPIVFRFNLRNTVDIFVVFFGGSSRAAWVGWDAWHAGQWDNVLLVHLRDRCSTFGGGIPDGHSLHTGGQLTRWTSPCQI
YAWLISSIGFKLNLHQCIDHSVELIDLYLAIWVCNWASASPDVVGQRQRHPLAPIGGSGDGTSFHLQQPMEADDGAFGVW
NDGRVDAPTPHGCAVHPGSLPGCSWRSLQVPVDKRSLEMAVLLPLYFPLDKQEEEIVILKLPIVGDENDMPIMRKASLHR
FLEKRKDRLNANGPYQTSPSDAAPVKKEPESQAWLGLGPNAVKSWSYALLSQFWIKSLVVYGSNSSNLRGYFKSDSYLDP
LFMSIIKDPDLWCKEMGKFAQAQKGFSYARLIFSSNYNYRGVDIEGHPIMVVVGAHFLLQCLDLERFVLHVVRVNIQKDN
GVQMKIIGTHVDAT

AGPv4:
Zm00001d024455
Chr10: 71686709..71688619

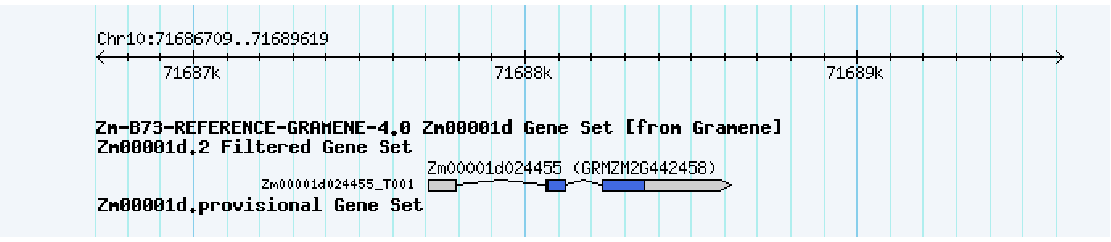

>Zm00001d024455 Genomic DNA
TTGCTCCCACTGTATTTCCCTCTAGACAAACAGGAGGAAGAGATTGTGATACTAAAGCTCCCTATTGTTGGGGATGAGAA
TGGTGAGACGGCAATAGATGGAAGGAAAATAAGAATAAGACATTTGCCTGCTGGTGTGGTAGATAGAATAGTGTCTGCAA
CTATTGTTACTCTTCCTCTTTTCTAATTCTATAGAGGGAGCAGGTGCAGAAGGTTGAGGCAAAACAACATTCTGTACCAA
TGTCTCTGGTAAATACTCCTCTCTCTGTGTTTTTTTCCTTGGTTGAAGTTTTAAAATGTGATTGGTGAACTTTGCCACAC
CGATTATTAATCAAGACATGTCTTCTCTTTTGTAGATATGCCAATTATGAGGAAGGCTTCTCTTCACCGCTTCCTTGAGA
AGAGAAAGGACCGGTAAGTTGTGGTTGTTGTTATTGTTATTTTCAGTGTGGTGATTTGTTTGTTTTTTCTCTCTCTCAGT
GTTCTGTTTGGAACTAATTTGTGCCCTTTTGATGTTCTTCAGTCTCAATGCAAATGGACCATACCAAACTTCTCCTTCAG
ACGCTGCACCAGTCAAGAAGGAGCCCGAGAGCCAGGCATGGCTCGGATTAGGACCGAATGCCGTCAAGTCCAACCTGAAC
CTAAGCTAGCACACAACGACACAGCCAAATAAGACGGCACAGCAACCTCACCAAAGCCTTTGCAGGACGATCTAAAAATA
TGCTTTGCCACTAGAGTACCGAAACCATAAATATTCTCTCGTTATCGTGTTTTGTGTTATGTTTAGGTCGTGGTCCTATG
CCCTCTTGTCACAGTTCTGGATGTAAAGATAAGTAGCCAGCCATGACCAAGAGAAGTTTTTTTTGGGGGGGTGTTAACA
CACGGCCACTCTGAATTGGCCATGCTAAGGA
>Zm00001d024455_T001 cDNA
TTGCTCCCACTGTATTTCCCTCTAGACAAACAGGAGGAAGAGATTGTGATACTAAAGCTCCCTATTGTTGGGGATGAGAA
TGATATGCCAATTATGAGGAAGGCTTCTCTTCACCGCTTCCTTGAGAAGAGAAAGGACCGTCTCAATGCAAATGGACCAT
ACCAAACTTCTCCTTCAGACGCTGCACCAGTCAAGAAGGAGCCCGAGAGCCAGGCATGGCTCGGATTAGGACCGAATGCC
GTCAAGTCCAACCTGAACCTAAGCTAGCACACAACGACACAGCCAAATAAGACGGCACAGCAACCTCACCAAAGCCTTTG
CAGGACGATCTAAAAATATGCTTTGCCACTAGAGTACCGAAACCATAAATATTCTCTCGTTATCGTGTTTTGTGTTATGT
TTAGGTCGTGGTCCTATGCCCTCTTGTCACAGTTCTGGATGTAAAGATAAGTAGCCAGCCATGACCAAGAGAAGTTTTTT
TTGGGGGGGTGTTAACACACGGCCACTCTGAATTGGCCATGCTAAGGA
>Zm00001d024455_P001 peptide
MPIMRKASLHRFLEKRKDRLNANGPYQTSPSDAAPVKKEPESQAWLGLGPNAVKSNLNLS

ZmJAZ33

AGPv4:
Zm00001d033972
Chr1: 279899021..279902559

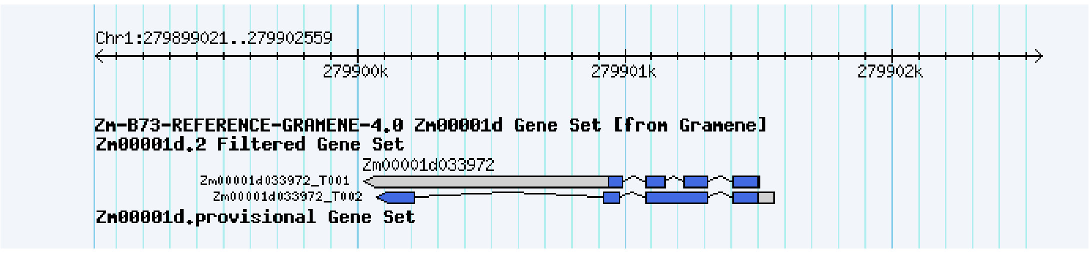

>Zm00001d033972 Genomic DNA
GGTGCTGTTTTATCTGCCTTTGTGTGTTCTTATTTTTTTGCATTCTTTACTACAAGGTCTGCAATGTTGCCGATGTCGAA
CCCTTCAGCTAATCCCACACAGCTTACAATTTTCTATGGTGGATCAGTATGTGTGTATGACTCGGTGCCACCAGAAAAGG
TAAATATTATCTTCACGCCATTTACAGATATCTTGTCCTTCTGTACCATCTGTTAAAATACACATGCTAATACACTAATT
TGGCATTGACAGGCTCAGGCAATCATGCTTATAGCTGCAGCTGCGGCAGCTGCGGCAGCCACCAAAGGCAGTGCTGCCAC
TGCTTTTAATCCTCCAATGGTACATACAGCCACTGTCGCCCCAGCAGCAGTCTTCTCTCCTGTGCTTACACGGTCTCCAT
CACTGCAGAGCACTTCTGTAGCAGCTGGACAAGCTCAGGTTGTTGCTGACCCTAGCTCAATAAGCAAGCTTCAGGCTGGT
AAGACCACTGCATCTTTGTGTAAAAATCCAGAAATGGAATACCTTTTCCAGTGTTGACATTGTCCAACACGTCTTTAAAC
TATCTTGATCTGTTGGCCAGATCTCCCCATTGCCAGGAGGCACTCTCTCCAGCGCTTCCTTGAGAAACGTCGTGACAGGT
TAGCATGATACATGAGAGCCCTGTTATTCTACAATCTACATGTTTAAAACTTTTGTTATAAACTGCTGTATGTGGTACCA
CACTAATTTTTTCCCCGACTATTGGCACCACAGTAATCAACAGTATATTTTGTACCGTATTCTGTTTTTTGTCCTCCAGG
CAGGGTAAAAATAAAATAAAATTTGCTGTTATAGCATTTAAATGATTGCACTTTCTGCTGACAAGTGCTCAATGACATCC
CTAAAAAAATCTATAATTTCTTCACAGAAAGATTGGTAGAGAGCCTGCTTCCATAATACAACATATAGCTGCAAATAGCC
TTGGATCATGTAACTCCCTTGAGATTTTTTTTAAAAAATCTGCAAATCGATATTAACACAAGACAGAGTTCTGGGACCA
GCAGTAGATACAAAAACCAACATTACCCCCATTTAGCTGCTCCAAATACCATGTTTTTCCCCTGTTAGAAAAATAACATA
TCTGAGAATCGGGATGTTCCTGTGTATTATCCTGTTCTATTTGGAAAAGCTGGCCACTTAAAGATCCAGTCTCAAGGTTG
CAAACTAGCACCATTTTTTTTCTGATGTAAGCTTTAGATGCCTGTTTTTGCCTTATCTTAGAACTGAGTTTAGAATTGTT
TCTTAGTATAACTTTTTTATCGTGGATCTACTGATTTCAAGTGATTAAATAAATGATGGCACGAAACAGGGTCGTGAGCA
AAGCTCCGTACAGCCCCGCCAAGTCGTTCGATGGCATGGAGTCAGCGGGAATGGAGATGACTGTGGACGGCAAGCAGGGC
GCAAGGCCCAGTATCTTGAAAGGTTGGGGAGCCGCGAAGTCTGAAGTCTGAACGGTGAAAGCGTAGCCCCGCGGGAGCGT
GGCGTTGTCTGGGAAGCGC
>Zm00001d033972_T001 cDNA
Chr1: 279900021..279901505
AGGTCTGCAATGTTGCCGATGTCGAACCCTTCAGCTAATCCCACACAGCTTACAATTTTCTATGGTGGATCAGTATGTGT
GTATGACTCGGTGCCACCAGAAAAGGCTCAGGCAATCATGCTTATAGCTGCAGCTGCGGCAGCTGCGGCAGCCACCAAAG
GCAGTGCTGCCACTGCTTTTAATCCTCCAATGAGCACTTCTGTAGCAGCTGGACAAGCTCAGGTTGTTGCTGACCCTAGC
TCAATAAGCAAGCTTCAGGCTGATCTGTTGGCCAGATCTCCCCATTGCCAGGAGGCACTCTCTCCAGCGCTTCCTTGAGA
AACGTCGTGACAGGTTAGCATGATACATGAGAGCCCTGTTATTCTACAATCTACATGTTTAAAACTTTTGTTATAAACTG
CTGTATGTGGTACCACACTAATTTTTTCCCCGACTATTGGCACCACAGTAATCAACAGTATATTTTGTACCGTATTCTGT
TTTTTGTCCTCCAGGCAGGGTAAAAATAAAATAAAATTTGCTGTTATAGCATTTAAATGATTGCACTTTCTGCTGACAAG
TGCTCAATGACATCCCTAAAAAAATCTATAATTTCTTCACAGAAAGATTGGTAGAGAGCCTGCTTCCATAATACAACATA
TAGCTGCAAATAGCCTTGGATCATGTAACTCCCTTGAGATTTTTTTTAAAAAATCTGCAAATCGATATTAACACAAGAC
AGAGTTCTGGGACCAGCAGTAGATACAAAAACCAACATTACCCCCATTTAGCTGCTCCAAATACCATGTTTTTCCCCTGT
TAGAAAAATAACATATCTGAGAATCGGGATGTTCCTGTGTATTATCCTGTTCTATTTGGAAAAGCTGGCCACTTAAAGAT
CCAGTCTCAAGGTTGCAAACTAGCACCATTTTTTTTCTGATGTAAGCTTTAGATGCCTGTTTTTGCCTTATCTTAGAACT
GAGTTTAGAATTGTTTCTTAGTATAACTTTTTTATCGTGGATCTACTGATTTCAAGTGATTAAATAAATGATGGCACGAA
ACAGGGTCGTGAGCAAAGCTCCGTACAGCCCCGCCAAGTCGTTCGATGGCATGGAGTCAGCGGGAATGGAGATGACTGTG
GACGGCAAGCAGGGCGCAAGGCCCAGTATCTTGAAAGGTTGGGGAGCCGCGAAGTCTGAAGTCTGAACGGTGAAAGCGTA
GCCCCGCGGGAGCGTGGCGTTGTCTGGGAAGCGC
>Zm00001d033972_P001 peptide
MLPMSNPSANPTQLTIFYGGSVCVYDSVPPEKAQAIMLIAAAAAAAAATKGSAATAFNPPMSTSVAAGQAQVVADPSSIS
KLQADLLARSPHCQEALSPALP
>Zm00001d033972_T002 cDNA
Chr1: 279900069..279901559

GGTGCTGTTTTATCTGCCTTTGTGTGTTCTTATTTTTTTGCATTCTTTACTACAAGGTCTGCAATGTTGCCGATGTCGAA
CCCTTCAGCTAATCCCACACAGCTTACAATTTTCTATGGTGGATCAGTATGTGTGTATGACTCGGTGCCACCAGAAAAGG
CTCAGGCAATCATGCTTATAGCTGCAGCTGCGGCAGCTGCGGCAGCCACCAAAGGCAGTGCTGCCACTGCTTTTAATCCT
CCAATGGTACATACAGCCACTGTCGCCCCAGCAGCAGTCTTCTCTCCTGTGCTTACACGGTCTCCATCACTGCAGAGCAC
TTCTGTAGCAGCTGGACAAGCTCAGGTTGTTGCTGACCCTAGCTCAATAAGCAAGCTTCAGGCTGATCTCCCCATTGCCA
GGAGGCACTCTCTCCAGCGCTTCCTTGAGAAACGTCGTGACAGGGTCGTGAGCAAAGCTCCGTACAGCCCCGCCAAGTCG
TTCGATGGCATGGAGTCAGCGGGAATGGAGATGACTGTGGACGGCAAGCAGGGCGCAAGGCCCAGTATCTTGAAAGGTTG
GGGAGCCGCGAAGTCTGAAGTCTGA

>Zm00001d033972_P002 peptide

MLPMSNPSANPTQLTIFYGGSVCVYDSVPPEKAQAIMLIAAAAAAAAATKGSAATAFNPPMVHTATVAPAAVFSPVLTRS
PSLQSTSVAAGQAQVVADPSSISKLQADLPIARRHSLQRFLEKRRDRVVSKAPYSPAKSFDGMESAGMEMTVDGKQGARP
SILKGWGAAKSEV

ZmJAZ34

AGPv4:

Zm00001d041045

Chr3: 92629179..92631799

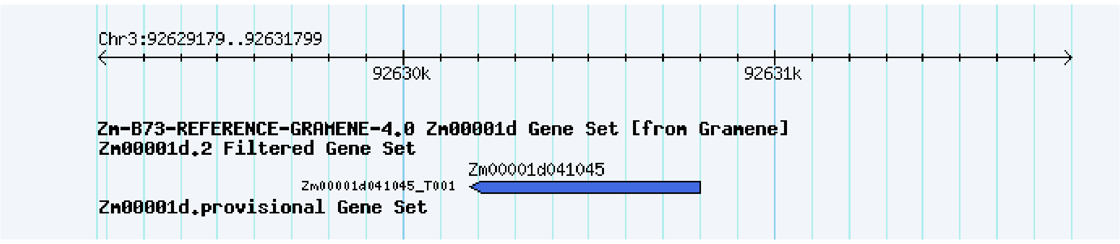

>Zm00001d041045 Genomic DNA

ATGGCTCCCTCTGCCCCTCCTGTTCGTAGTTCTTCTCCCAACACGCACGTTACCAACCGCTCCCTCCCTCCACCTCCGGG
CGCCCCGGCCACCACGACCCTGAGCCTGATGCCCGGAGCGGACGTCGGCGCCGACGAGCAGCAGGAGTCGGCCGCCGCGA
GGGCCGAGGAGATGCCCGGGCCCGCCACCGCCGCGGCGCCGCCGCTCACCATCTTCTATGGCGGCATGGTGGTGGTGTTC
GAGGACTTCCCGGCGGAGAAGGCAGCCGAGGTCATGCGCCTCGCGGGCGGCGACGACCTGCCCATCGCCCGAAAGGCGTC
GCTCCAGCGGTTCCTGGCCAAGCGCAAGGACCGGCTCATCGAGCCCTACGCCCGCCCGTCGTCCCCCGCGGAGGAGCCGG
AGAAGAAGACAGTCAAGACAGCCTTGGCCTTGGCCTCCTGGCTCGGGCTCGGCAGCACGGAGGCCAACCGCCTCACCATC
ACGCTGTTCTGCGGTTCTGCCCACCCCGACGGGTGGGCCCCATCTTCGAGACTCTGTCTCCTGCTGCAGCTCGCCCGCCG
CCCCACTCCCAAGATTTTCGCCGTGCTCGCTACCTGGTCTGCCCCGCCGTCTTCCCCCTGA

>Zm00001d041045_T001 cDNA

ATGGCTCCCTCTGCCCCTCCTGTTCGTAGTTCTTCTCCCAACACGCACGTTACCAACCGCTCCCTCCCTCCACCTCCGGG
CGCCCCGGCCACCACGACCCTGAGCCTGATGCCCGGAGCGGACGTCGGCGCCGACGAGCAGCAGGAGTCGGCCGCCGCGA
GGGCCGAGGAGATGCCCGGGCCCGCCACCGCCGCGGCGCCGCCGCTCACCATCTTCTATGGCGGCATGGTGGTGGTGTTC
GAGGACTTCCCGGCGGAGAAGGCAGCCGAGGTCATGCGCCTCGCGGGCGGCGACGACCTGCCCATCGCCCGAAAGGCGTC
GCTCCAGCGGTTCCTGGCCAAGCGCAAGGACCGGCTCATCGAGCCCTACGCCCGCCCGTCGTCCCCCGCGGAGGAGCCGG
AGAAGAAGACAGTCAAGACAGCCTTGGCCTTGGCCTCCTGGCTCGGGCTCGGCAGCACGGAGGCCAACCGCCTCACCATC
ACGCTGTTCTGCGGTTCTGCCCACCCCGACGGGTGGGCCCCATCTTCGAGACTCTGTCTCCTGCTGCAGCTCGCCCGCCG
CCCCACTCCCAAGATTTTCGCCGTGCTCGCTACCTGGTCTGCCCCGCCGTCTTCCCCCTGA

>Zm00001d041045_P001 peptide

MAPSAPPVRSSSPNTHVTNRSLPPPPGAPATTTLSLMPGADVGADEQQESAAARAEEMPGPATAAAPPLTIFYGGMVVVF

EDFPAEKAAEVMRLAGGDDLPIARKASLQRFLAKRKDRLIEPYARPSSPAEEPEKKTVKTALALASWLGLGSTEANRLTI
TLFCGSAHPDGWAPSSRLCLLLQLARRPTPKIFAVLATWSAPPSSP

ZmJAZ35

AGPv4:
Zm00001d044708
Chr3: 235520147..235523705

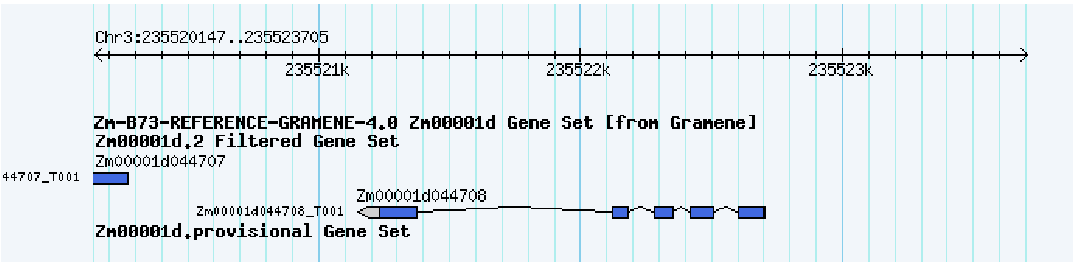

>Zm00001d044708 Genomic DNA
AGGTCTGCAATGTTGCCGATGTCGAACCCTTCAGCTAATCCCACACAGCTTACAATTTTCTATGGTGGATCAGTATGTGT
GTATGACTCGGTGCCACCAGAAAAGGTAAATATTATCTTCACGCCATTTACAGATATCTTGTCCTTCTGTACCATCTGTT
AAAATACACATGCTAATACACTAATTTGGCATTGACAGGCTCAGGCAATCATGCTTATAGCTGCAGCTGCGGCAGCTGCG
GCAGCCACCAAAGGCAGTGCTGCCACTGCTTTTAATCCTCCAATGGTACATACAGCCACTGTCTCCCCAGCAGCAGTCTT
CTCTCCTGTGCTTACACGGTCTCCATCACTGCAGAGCACTTCTGTAGCAGCTGGACAAGCTCAGGTTGTTGCTGACCCTA
GCTCAATAAGCAAGCTTCAGGCTGGTAAGACCACTGCATCTTTGTGTAAAAATCCATAAATGGAATACCTTTTCCAGTGT
TGACATTGTCCAACACGTCTTTAAACTATCTTGATCTGTTGGCCAGATCTCCCCATTGCCAGGAGGCACTCTCTCCATCG
CTTCCTTGAGAAACGTCGTGACAGGTTAGCATGATACATGAGAGCAACAGGGCTCTGATATTCTGAATCTTATTGTTAGG
CCTCTTATTCTACAATCTACATGTTTAAAACTTTTGTTATAAACTGCTGTATGTGGTACCACACTAATTTTTTCCCCGAC
TATTGGCACCACAGTAATCAACAGTATATTTTGTACCGTATTCTGTTTTTTGTCCTCCAGGCAGGGTAAAAATAAAATAA
AATTTGCTGTTATAGCATTTAAATGATTGCACTTTCTGCTGACAAGTGCTCAATGACATCCCTAAAAAAATCTATAATTT
CTTCACAGAAAGATTGGTAGAGAGCCTGCTTCCATAATACAACATATAGCTGCAAATAGCCTTGGATCATGTAACTCCCT
TGAGATTTTTTTAAAAAATCTGCAAATCGATATTAACACAAGACAGAGTTCTGGGACCAGCAGTAGATACAAAAACCAA
CATTACCCCCATTTAGCTGCTCCAAATACCATGTTTTTCCCCTGTTAGGAAAATAACATATTTGAGAATCGGGATGTTCC
TGTGTATTATCCTGTTCTATTTGGAAAAGCTGGCCACTTAAAGATCCAGTCTCAAGGTTGCAAACTAGCACCATTTTTTT
TCTGATGTAAGCTTTAGATGCCTGTTTTTGCCTTATCTTAGAACTGAGTTTAGAATTGTTTCTTAGTATAACTTTTTTTT
ATCGTGGATCTACTGATTTCAAGTGATTAAATAAATGATGGCACGAAACAGGGTCGTGAGCAAAGCTCCGTACAGCCCCG
CCAAGTCGTTCGATGGCATGGAGTCAGCGGGAATGGAGATGACTGTGGACGGCAAGCAGGGCGCAAGGCCCAGTATCTTG
AAAGGTTGGGGAGCCGCGAAGTCTGAAGTCTGAACGGTGAAAGCGTAGCCCTGCGGGAGCGTGGCGTTGTCTGGGAAGCG
CCAGAAGCCGAGGTAGAGGGTGCAGAGGGAGGCAAGGTG
>Zm00001d044708_T001 cDNA
AGGTCTGCAATGTTGCCGATGTCGAACCCTTCAGCTAATCCCACACAGCTTACAATTTTCTATGGTGGATCAGTATGTGT
GTATGACTCGGTGCCACCAGAAAAGGCTCAGGCAATCATGCTTATAGCTGCAGCTGCGGCAGCTGCGGCAGCCACCAAAG
GCAGTGCTGCCACTGCTTTTAATCCTCCAATGAGCACTTCTGTAGCAGCTGGACAAGCTCAGGTTGTTGCTGACCCTAGC
TCAATAAGCAAGCTTCAGGCTGATCTCCCCATTGCCAGGAGGCACTCTCTCCATCGCTTCCTTGAGAAACGTCGTGACAG
GGTCGTGAGCAAAGCTCCGTACAGCCCCGCCAAGTCGTTCGATGGCATGGAGTCAGCGGGAATGGAGATGACTGTGGACG
GCAAGCAGGGCGCAAGGCCCAGTATCTTGAAAGGTTGGGGAGCCGCGAAGTCTGAAGTCTGAACGGTGAAAGCGTAGCCC
TGCGGGAGCGTGGCGTTGTCTGGGAAGCGCCAGAAGCCGAGGTAGAGGGTGCAGAGGGAGGCAAGGTG

>Zm00001d044708_P001 peptide
MLPMSNPSANPTQLTIFYGGSVCVYDSVPPEKAQAIMLIAAAAAAAAATKGSAATAFNPPMSTSVAAGQAQVVADPSSIS
KLQADLPIARRHSLHRFLEKRRDRVVSKAPYSPAKSFDGMESAGMEMTVDGKQGARPSILKGWGAAKSEV

ZmJAZ36

AGPv4:
Zm00001d046270
Chr9: 77364055..77367403

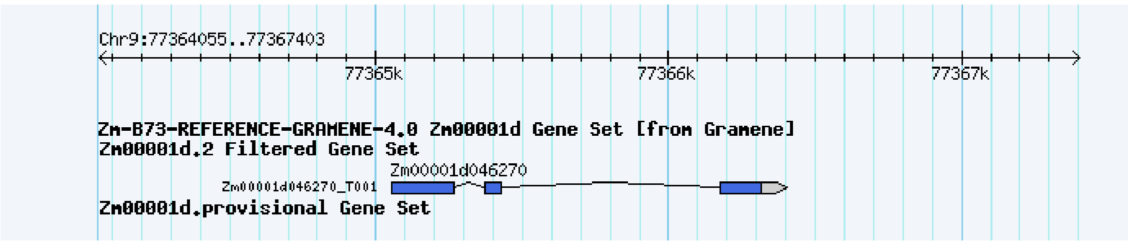

>Zm00001d046270 Genomic DNA
ATGCTTATAGCTGCAGCTGCGGCAGCTGCGGCAGCCACCAAAGGCAGTGCTGCCACTGCTTTTAATCCTCCAATGGTACA
TACAGCCACTGTCGCCCCAGCAGCAGTCTTCTCTCCTGTGCTTACACGGTCTCCATCACTGCAGAGCACTTCTGTAGCAG
CTGGACAAGCTCAGGTTGTTGCTGACCCTAGCTCAATAAGCAAGCTTCAGGCTGGTAAGACCACTACATCTTTGTGTAAA
AATCCAGAAATGGAATACCTTTTCCAGTGTTGACATTGTCCAACACGTCTTTAAACTATCTTGATCTGTTGGCCAGATCT
CCCCATTGCCAGGAGGCACTCTCTCCAGCGCTTCCTTGAGAAACGTCGTGACAGGTTAGCATGATACATGAGAGCAACAG
GGCTCTGATATTCTGAATCTTATTGTTAGGCCTCTTATTCTACAATCTACATGTTTAAAACTTTTGTTATAAACTGCTGT
ATGTGGTACCACACTAATTTTTTCCCCGACTATTGGCACCACAGTAATCAACAGTATATTTTGTACCGTATTCTGTTTTT
TGTCCTCCAGGCAGGGTAAAAATAAAATAAAATTTGCTGTTATAGCATTTAAATGATTGCACTTTCTGCTGACAAGTGCT
CAATGACATCCCTAAAAAAAATCTATAATTTCTTCACAGAAAGATTGGTAGAGAGCCTGCTTCCATAATACAACATATAG
CTGCAAATAGCCTTGGATCATGTAACTCCCTTGAGATTTTTTTAAAAAATCTGCAAATCGATATTAACACAAGACAGAG
TTCTGGGACCAGCAGTAGATACAAAAACCAACATTACCCCCATTTAGCTGCTCCAAATACCATGTTTTTCCCCTGTTAGG
AAAATAACATATCTGAGAATCGGGATGTTCCTGTGTATTATCCTGTTCTATTTGGAAAAGCTGGCCACTTAAAGATCCAG
TCTCAAGGTTGCAAACTAGCACCATTTTTTTTCTGATGTAAGCTTTAGATGCCTGTTTTTGCCTTATCTTAGAACTGAGT
TTAGAATTGTTTCTTAGTATAACTTTTTTTATCGTGGATCTACTGATTTCAAGTGATTAAATAAATGATGGCACGAAACA
GGGTCGTGAGCAAAGCTCCGTACAGCCCCGCCAAGTCGTTCGATGGCATGGAGTCAGCGGGAATGGAGATGACTGTGGAC
GGCAAGCAGGGCGCAAGGCCCAGTATCTTGAAAGATTGGGGAGCCGCGAAGTCTGAAGTCTGAACGGTGAAAGCGTAGCC
CCGCGGGAGCGTGGCGTTGTCTGGGAAGCGCCAGAAGCCGAGGTAGAGGGTGCAGAGGGAGGCAAGGTG
>Zm00001d046270_T001 cDNA
ATGCTTATAGCTGCAGCTGCGGCAGCTGCGGCAGCCACCAAAGGCAGTGCTGCCACTGCTTTTAATCCTCCAATGGTACA
TACAGCCACTGTCGCCCCAGCAGCAGTCTTCTCTCCTGTGCTTACACGGTCTCCATCACTGCAGAGCACTTCTGTAGCAG
CTGGACAAGCTCAGGTTGTTGCTGACCCTAGCTCAATAAGCAAGCTTCAGGCTGATCTCCCCATTGCCAGGAGGCACTCT
CTCCAGCGCTTCCTTGAGAAACGTCGTGACAGGGTCGTGAGCAAAGCTCCGTACAGCCCCGCCAAGTCGTTCGATGGCAT
GGAGTCAGCGGGAATGGAGATGACTGTGGACGGCAAGCAGGGCGCAAGGCCCAGTATCTTGAAAGATTGGGGAGCCGCGA
AGTCTGAAGTCTGAACGGTGAAAGCGTAGCCCCGCGGGAGCGTGGCGTTGTCTGGGAAGCGCCAGAAGCCGAGGTAGAGG
GTGCAGAGGGAGGCAAGGTG
>Zm00001d046270_P001 peptide
MLIAAAAAAAAATKGSAATAFNPPMVHTATVAPAAVFSPVLTRSPSLQSTSVAAGQAQVVADPSSISKLQADLPIARRHS
LQRFLEKRRDRVVSKAPYSPAKSFDGMESAGMEMTVDGKQGARPSILKDWGAAKSEV

ZmTIFY1

B73 RefGen_v3:

GRMZM2G110131 (zim22 - ZIM-transcription factor 22)

Chr1: 30037028..30039032

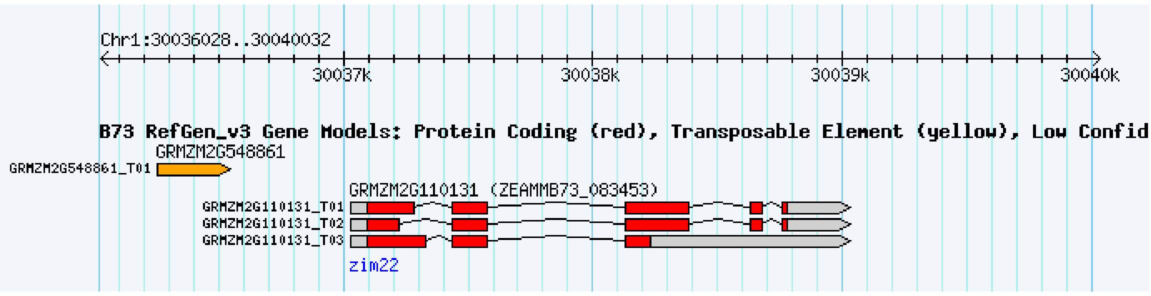

>GRMZM2G110131 Genomic DNA

ACTACCCTGCTCCCTCTCCCAAGCCACCGCCACTCCTTCTCCCTCTTGCGCAACCAGAGCGCACAAACCCAATGCGGCGC
CGGCCCCTCCTCCCTCTTGCGCAATTTGGCTGTGTGGCCACGGACTATCAAGAACAGGCAGTGGATCCTGACCCCTTTTT
CTCTCATTCCAGCAGCAGCGGTGGAGAAGATCCTGCAGGTAGTGCAGCAGTGAGGCGGTGGATCTCCAGACAGTGCAGCA
GTTGGGTGGCAGGTGGAGCAGCCCCAGCCAGTTTCGTTTCTAGTGGGGAGCGTGTTGTGCTGGTGTGAGATCTGGGCGGG
GCCTTTGTTATCCGGTTTGAAACCACGTTTTCGGGCTTCGAATTTTGCGGTCCCTTATCCGGATGTTTTTTTTGGGTTCC
CGTTCTCAGGCGAGGTCGAGCAGCAGAGGACGCCGGCGACAACTAACTTGCTCTCCGGAGCGGAGGGCGAGGAGGTCGAA
AGGAGGAAGGAGACCATGGAGCTGTTCCCGCAGAGCGTCGGGTTCAGCATCAAGGATGCTGCTGCCCCTAGGTGCGTACC
ACTGCTGCTTCACATCATCTAAATCCATTTCGATTTTGGTTTGCGCAACAGTTGTTACGTAGTAGCACAGCAGAACTCAA
GCCTAGCTAGCTGTCTTATGTCTCCTGGACAAAAGGCCAAGCAATTTGACTTTGTAACATAGAGATCTGAGTTTAATGAG
TGTTTATAGGTGGAAGTGGAACATGTGGTATAAAATCTAGTTGAGCCGGATGGCAAATGACAGAGCATGTTATTACGTAT
GTGTAAAGCTAAAGCATCCCTTGCTGTTCAGTCTGTGTTGGTATTCCTTGGTCAAAGCTGTATCTTTGTAATCTTAAGAA
GTTACCGCCTTTGCTAAAACACCGTGTTCTGCAAGGTAACACTGTTTCCTTTTATCAGGCAAAGCAACCAACATGCTCAG
AGATTGTACCTAATGTTTATCATTAACTGCCAAGGATTATTAACAGGAAAAAAAAAACAGAGTGGATAATTGATTGGCTA
AGCTGAAGTAGCCTACTACATGCACTGTTCCTTAGTGTTCTTCTCTGTAATATTTGTCAGGGAGGAGCAAGGAGATAAAG
AGAAGCCTAAGCAGCTCACAATCTTCTATGGCGGGAAGGTGCTGGTATTTGACGATTTCCCCGCCGACAAGGCAAAGGAT
CTGATGCAGCTGGCCAGCAAGGGCAGCCCAGTGGTACAGAACGTTGTTTGCCTCAACCCTCTGCAGCTGCTGCTGTCAG
TACTGACAAGGCCGTGCTGGACCCGGTCATCAGCTTGGCCGCTGCTAAGAAGCCTGCTCGCACAAATGCTTCTGGTATAA
ACACTCTCCCTCTGGCCAGCTGGGGTATTTATTGAGTTAGCATCTCTAAAATATTACATTTAAGTTAGCATCTCTAAAAT
ATTACATGATATTTATTGAGTTTTTTATATGTCTTATAAGTTTTCCCAATTATTTCTAAACCAACCTATAGCTTATGTTT
TGATCTACATGCTCACTAAATATATTTGATATCTTTTCAGCGACAAGAAAATTATAACCAAGTGCAATTGTTTTTTTAAA
GCAGATGCATTACATCAAGAAAAAGATTTAGTGAAACTGTTTAGAAAGCTATTGTAAGTACATATTTACCTTTTTCCCTT
CATTTTCGACTTATGTTACGCTAGACTAAGTTTTAATCTTGATGCTAGGATGATATTGATGCTCGGATGATATGGAAGCA
ACGATAGCAGTACCAGTTGAAGATGGACAGTTTGCGAAGTCTTCTACATAAATTTCACATGTCTTGCTTTTGTGACTGAT
CAGCTCTGGAGTAGCTAGAGTGATGTAGTTGTGGGCTTTTGAACAATAGCTGGAGTGATATGAAACATTAGTGATGTAGT
TGTGGGCTTTATAATTGTGAATGATATTAGTAAATGATCTAGTTGTGAATGATATTATAATTGTGATGCTTTTGTGAATA
TATAA

>GRMZM2G110131_T01 cDNA

Chr1: 30037028..30039032

ACTACCCTGCTCCCTCTCCCAAGCCACCGCCACTCCTTCTCCCTCTTGCGCAACCAGAGCGCACAAACCCAATGCGGCGC
CGGCCCCTCCTCCCTCTTGCGCAATTTGGCTGTGTGGCCACGGACTATCAAGAACAGGCAGTGGATCCTGACCCCTTTTT

CTCTCATTCCAGCAGCAGCGGTGGAGAAGATCCTGCAGGTAGTGCAGCAGTGAGGCGGTGGATCTCCAGACAGTGCAGCA
GTTGGGTGGCAGGCGAGGTCGAGCAGCAGAGGACGCCGGCGACAACTAACTTGCTCTCCGGAGCGGAGGGCGAGGAGGTC
GAAAGGAGGAAGGAGACCATGGAGCTGTTCCCGCAGAGCGTCGGGTTCAGCATCAAGGATGCTGCTGCCCCTAGGGAGGA
GCAAGGAGATAAAGAGAAGCCTAAGCAGCTCACAATCTTCTATGGCGGGAAGGTGCTGGTATTTGACGATTTCCCCGCCG
ACAAGGCAAAGGATCTGATGCAGCTGGCCAGCAAGGGCAGCCCAGTGGTACAGAACGTTGTTTTGCCTCAACCCTCTGCA
GCTGCTGCTGTCAGTACTGACAAGGCCGTGCTGGACCCGGTCATCAGCTTGGCCGCTGCTAAGAAGCCTGCTCGCACAAA
TGCTTCTGATGCATTACATCAAGAAAAAGATTTAGTGAAACTGTTTAGAAAGCTATTGATGATATTGATGCTCGGATGAT
ATGGAAGCAACGATAGCAGTACCAGTTGAAGATGGACAGTTTGCGAAGTCTTCTACATAAATTTCACATGTCTTGCTTTT
GTGACTGATCAGCTCTGGAGTAGCTAGAGTGATGTAGTTGTGGGCTTTTGAACAATAGCTGGAGTGATATGAAACATTAG
TGATGTAGTTGTGGGCTTTATAATTGTGAATGATATTAGTAAATGATCTAGTTGTGAATGATATTATAATTGTGATGCTT
TTGTGAATATATAA

>GRMZM2G110131_P01 peptide

MRRRPLLPLAQFGCVATDYQEQAVDPDPFFSHSSSSGGEDPAGSAAVRRWISRQCSSWVAGEVEQQRTPATTNLLSGAEG
EEVERRKETMELFPQSVGFSIKDAAAPREEQGDKEKPKQLTIFYGGKVLVFDDFPADKAKDLMQLASKGSPVVQNVVLPQ
PSAAAAVSTDKAVLDPVISLAAAKKPARTNASDALHQEKDLVKLFRKLLMILMLG

>GRMZM2G110131_T02 cDNA

Chr1: 30037028..30039032

ACTACCCTGCTCCCTCTCCCAAGCCACCGCCACTCCTTCTCCCTCTTGCGCAACCAGAGCGCACAAACCCAATGCGGCGC
CGGCCCCTCCTCCCTCTTGCGCAATTTGGCTGTGTGGCCACGGACTATCAAGAACAGGCAGTGGATCCTGACCCCTTTTT
CTCTCATTCCAGCAGCAGCGGTGGAGAAGATCCTGCAGGCGAGGTCGAGCAGCAGAGGACGCCGGCGACAACTAACTTGC
TCTCCGGAGCGGAGGGCGAGGAGGTCGAAAGGAGGAAGGAGACCATGGAGCTGTTCCCGCAGAGCGTCGGGTTCAGCATC
AAGGATGCTGCTGCCCCTAGGGAGGAGCAAGGAGATAAAGAGAAGCCTAAGCAGCTCACAATCTTCTATGGCGGGAAGGT
GCTGGTATTTGACGATTTCCCCGCCGACAAGGCAAAGGATCTGATGCAGCTGGCCAGCAAGGGCAGCCCAGTGGTACAGA
ACGTTGTTTTGCCTCAACCCTCTGCAGCTGCTGCTGTCAGTACTGACAAGGCCGTGCTGGACCCGGTCATCAGCTTGGCC
GCTGCTAAGAAGCCTGCTCGCACAAATGCTTCTGATGCATTACATCAAGAAAAAGATTTAGTGAAACTGTTTAGAAAGCT
ATTGATGATATTGATGCTCGGATGATATGGAAGCAACGATAGCAGTACCAGTTGAAGATGGACAGTTTGCGAAGTCTTCT
ACATAAATTTCACATGTCTTGCTTTTGTGACTGATCAGCTCTGGAGTAGCTAGAGTGATGTAGTTGTGGGCTTTTGAACA
ATAGCTGGAGTGATATGAAACATTAGTGATGTAGTTGTGGGCTTTATAATTGTGAATGATATTAGTAAATGATCTAGTTG
TGAATGATATTATAATTGTGATGCTTTTGTGAATATATAA

>GRMZM2G110131_P02 peptide

MRRRPLLPLAQFGCVATDYQEQAVDPDPFFSHSSSSGGEDPAGEVEQQRTPATTNLLSGAEGEEVERRKETMELFPQSVG
FSIKDAAAPREEQGDKEKPKQLTIFYGGKVLVFDDFPADKAKDLMQLASKGSPVVQNVVLPQPSAAAAVSTDKAVLDPVI
SLAAAKKPARTNASDALHQEKDLVKLFRKLLMILMLG

>GRMZM2G110131_T03 cDNA

Chr1: 30037028..30039032

ACTACCCTGCTCCCTCTCCCAAGCCACCGCCACTCCTTCTCCCTCTTGCGCAACCAGAGCGCACAAACCCAATGCGGCGC
CGGCCCCTCCTCCCTCTTGCGCAATTTGGCTGTGTGGCCACGGACTATCAAGAACAGGCAGTGGATCCTGACCCCTTTTT
CTCTCATTCCAGCAGCAGCGGTGGAGAAGATCCTGCAGGTAGTGCAGCAGTGAGGCGGTGGATCTCCAGACAGTGCAGCA
GTTGGGTGGCAGGTGGAGCAGCCCCAGCCAGTTTCGTTTCTAGTGGGGAGCGTGTTGTGCTGGCGAGGTCGAGCAGCAGA
GGACGCCGGCGACAACTAACTTGCTCTCCGGAGCGGAGGGCGAGGAGGTCGAAAGGAGGAAGGAGACCATGGAGCTGTTC
CCGCAGAGCGTCGGGTTCAGCATCAAGGATGCTGCTGCCCCTAGGGAGGAGCAAGGAGATAAAGAGAAGCCTAAGCAGCT
CACAATCTTCTATGGCGGGAAGGTGCTGGTATTTGACGATTTCCCCGCCGACAAGGCAAAGGATCTGATGCAGCTGGCCA
GCAAGGGCAGCCCAGTGGTACAGAACGTTGTTTTGCCTCAACCCTCTGCAGCTGCTGCTGTCAGTACTGACAAGGCCGTG
CTGGACCCGGTCATCAGCTTGGCCGCTGCTAAGAAGCCTGCTCGCACAAATGCTTCTGATGCATTACATCAAGAAAAAGA

TTTAGTGAAACTGTTTAGAAAGCTATTGATGATATTGATGCTCGGATGATATGGAAGCAACGATAGCAGTACCAGTTGAA
GATGGACAGTTTGCGAAGTCTTCTACATAAATTTCACATGTCTTGCTTTTGTGACTGATCAGCTCTGGAGTAGCTAGAGT
GATGTAGTTGTGGGCTTTTGAACAATAGCTGGAGTGATATGAAACATTAGTGATGTAGTTGTGGGCTTTATAATTGTGAA
TGATATTAGTAAATGATCTAGTTGTGAATGATATTATAATTGTGATGCTTTTGTGAATATATAA

>GRMZM2G110131_P03 peptide

MRRRPLLPLAQFGCVATDYQEQAVDPDPFFSHSSSSGGEDPAGSAAVRRWISRQCSSWVAGGAAPASFVSSGERVVLARS
SSRGRRRQLTCSPERRARRSKGGRRPWSCSRRASGSASRMLLPLGRSKEIKRSLSSSQSSMAGRCWYLTISPPTRQRI

AGPv4:

Zm00001d028313 (zim22 - ZIM-transcription factor 22)

Chr1: 30341336..30345337

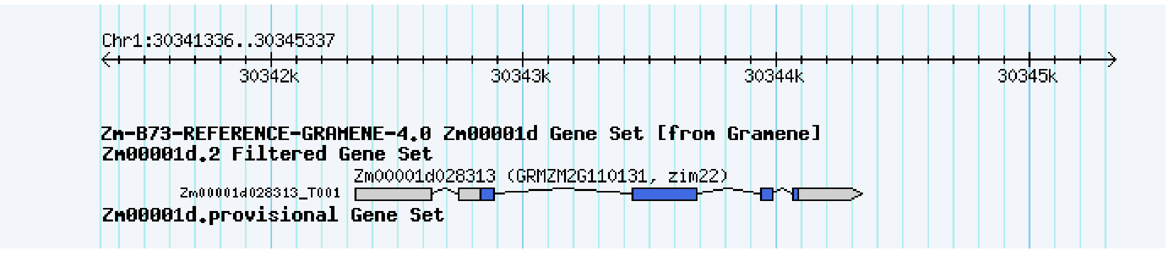

>Zm00001d028313 Genomic DNA

ACCCTGCTCCCTCTCCCAAGCCACCGCCACTCCTTCTCCCTCTTGCGCAACCAGAGCGCACAAACCCAATGCGGCGCCGG
CCCCTCCTCCCTCTTGCGCAATTTGGCTGTGTGGCCACGGACTATCAAGAACAGGCAGTGGATCCTGACCCCTTTTTCTC
TCATTCCAGCAGCAGCGGTGGAGAAGATCCTGCAGGTAGTGCAGCAGTGAGGCGGTGGATCTCCAGACAGTGCAGCAGTT
GGGTGGCAGGTGGAGCAGCCCCAGCCAGTTTCGTTTCTAGTGGGGAGCGTGTTGTGCTGGTGTGAGATCTGGGCGGGGCC
TTTGTTATCCGGTTTGAAACCACGTTTTCGGGCTTCGAATTTTGCGGTCCCTTATCCGGATGTTTTTTTTGGGTTCCCGT
TCTCAGGCGAGGTCGAGCAGCAGAGGACGCCGGCGACAACTAACTTGCTCTCCGGAGCGGAGGGCGAGGAGGTCGAAAGG
AGGAAGGAGACCATGGAGCTGTTCCCGCAGAGCGTCGGGTTCAGCATCAAGGATGCTGCTGCCCCTAGGTGCGTACCACT
GCTGCTTCACATCATCTAAATCCATTTCGATTTTGGTTTGCGCAACAGTTGTTACGTAGTAGCACAGCAGAACTCAAGCC
TAGCTAGCTGTCTTATGTCTCCTGGACAAAAGGCCAAGCAATTTGACTTTGTAACATAGAGATCTGAGTTTAATGAGTGT
TTATAGGTGGAAGTGGAACATGTGGTATAAAATCTAGTTGAGCCGGATGGCAAATGACAGAGCATGTTATTACGTATGTG
TAAAGCTAAAGCATCCCTTGCTGTTCAGTCTGTGTTGGTATTCCTTGGTCAAAGCTGTATCTTTGTAATCTTAAGAAGTT
ACCGCCTTTGCTAAAACACCGTGTTCTGCAAGGTAACACTGTTTCCTTTTATCAGGCAAAGCAACCAACATGCTCAGAGA
TTGTACCTAATGTTTATCATTAACTGCCAAGGATTATTAACAGGAAAAAAAAAACAGAGTGGATAATTGATTGGCTAAGC
TGAAGTAGCCTACTACATGCACTGTTCCTTAGTGTTCTTCTCTGTAATATTTGTCAGGGAGGAGCAAGGAGATAAAGAGA
AGCCTAAGCAGCTCACAATCTTCTATGGCGGGAAGGTGCTGGTATTTGACGATTTCCCCGCCGACAAGGCAAAGGATCTG
ATGCAGCTGGCCAGCAAGGGCAGCCCAGTGGTACAGAACGTTGTTTTGCCTCAACCCTCTGCAGCTGCTGCTGTCAGTAC
TGACAAGGCCGTGCTGGACCCGGTCATCAGCTTGGCCGCTGCTAAGAAGCCTGCTCGCACAAATGCTTCTGGTATAAACA
CTCTCCCTCTGGCCAGCTGGGGTATTTATTGAGTTAGCATCTCTAAAATATTACATTTAAGTTAGCATCTCTAAAATATT
ACATGATATTTATTGAGTTTTTTATATGTCTTATAAGTTTTCCCAATTATTTCTAAACCAACCTATAGCTTATGTTTTGA
TCTACATGCTCACTAAATATATTTGATATCTTTTCAGCGACAAGAAAATTATAACCAAGTGCAATTGTTTTTTTAAAGCA
GATGCATTACATCAAGAAAAAGATTTAGTGAAACTGTTTAGAAAGCTATTGTAAGTACATATTTACCTTTTTCCCTTCAT
TTTCGACTTATGTTACGCTAGACTAAGTTTTAATCTTGATGCTAGGATGATATTGATGCTCGGATGATATGGAAGCAACG
ATAGCAGTACCAGTTGAAGATGGACAGTTTGCGAAGTCTTCTACATAAATTTCACATGTCTTGCTTTTGTGACTGATCAG
CTCTGGAGTAGCTAGAGTGATGTAGTTGTGGGCTTTTGAACAATAGCTGGAGTGATATGAAACATTAGTGATGTAGTTGT
GGGCTTTATAATTGTGAATGATATTAGTAAATGATCTAGTTGTGAATGATATTATAATTGTGATGCTTTTGTGAATATAT
AA

>Zm00001d028313_T001 cDNA

ACCCTGCTCCCTCTCCCAAGCCACCGCCACTCCTTCTCCCTCTTGCGCAACCAGAGCGCACAAACCCAATGCGGCGCCGG
CCCCTCCTCCCTCTTGCGCAATTTGGCTGTGTGGCCACGGACTATCAAGAACAGGCAGTGGATCCTGACCCCTTTTTCTC
TCATTCCAGCAGCAGCGGTGGAGAAGATCCTGCAGGTAGTGCAGCAGTGAGGCGGTGGATCTCCAGACAGTGCAGCAGTT
GGGTGGCAGGTGGAGCAGCCCCAGCCAGTTTCGTTTCTAGTGGGGAGCGTGTTGTGCTGGCGAGGTCGAGCAGCAGAGGA
CGCCGGCGACAACTAACTTGCTCTCCGGAGCGGAGGGCGAGGAGGTCGAAAGGAGGAAGGAGACCATGGAGCTGTTCCCG
CAGAGCGTCGGGTTCAGCATCAAGGATGCTGCTGCCCCTAGGGAGGAGCAAGGAGATAAAGAGAAGCCTAAGCAGCTCAC
AATCTTCTATGGCGGGAAGGTGCTGGTATTTGACGATTTCCCCGCCGACAAGGCAAAGGATCTGATGCAGCTGGCCAGCA
AGGGCAGCCCAGTGGTACAGAACGTTGTTTTGCCTCAACCCTCTGCAGCTGCTGCTGTCAGTACTGACAAGGCCGTGCTG
GACCCGGTCATCAGCTTGGCCGCTGCTAAGAAGCCTGCTCGCACAAATGCTTCTGATGCATTACATCAAGAAAAAGATTT
AGTGAAACTGTTTAGAAAGCTATTGATGATATTGATGCTCGGATGATATGGAAGCAACGATAGCAGTACCAGTTGAAGAT
GGACAGTTTGCGAAGTCTTCTACATAAATTTCACATGTCTTGCTTTTGTGACTGATCAGCTCTGGAGTAGCTAGAGTGAT
GTAGTTGTGGGCTTTTGAACAATAGCTGGAGTGATATGAAACATTAGTGATGTAGTTGTGGGCTTTATAATTGTGAATGA
TATTAGTAAATGATCTAGTTGTGAATGATATTATAATTGTGATGCTTTTGTGAATATATAA

>Zm00001d028313_P001 peptide

MELFPQSVGFSIKDAAAPREEQGDKEKPKQLTIFYGGKVLVFDDFPADKAKDLMQLASKGSPVVQNVVLPQPSAAAAVST
DKAVLDPVISLAAAKKPARTNASDALHQEKDLVKLFRKLLMILMLG

ZmTIFY2

AGPv4:

Zm00001d004173

Chr2: 89164906..89166345

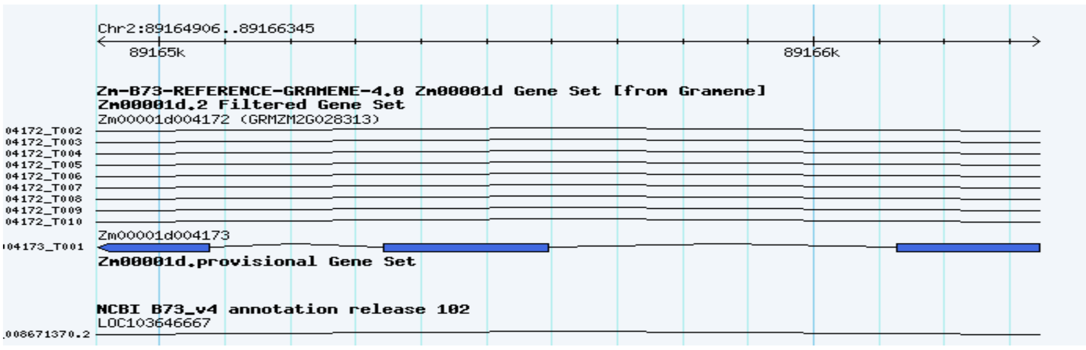

>Zm00001d004173 Genomic DNA

ATGGTTATCCCGTTTGAAACCATGTTTTCGGGCTTCGAATTTTCCATACCCTTCTCGGGCTTGTTTTCCTTCCCATTCTC
AGGCGAGGTCGAGCAACAGAGGACGCTGGCAACAATTAACTTGCCCTCCGGAGCGGAGGGCGAGGAGGCCAAGAAGAGGA
AGGAGAGCATGGAGCTGTTCCCACGGAGCGCCGGCGTCAAGGATGATGCTGCCCCTAGGTGCGTACTGCTGCTGCTTCAT
ATCATCTAAATCCATTTCGATTTTGGTTTTGCGCAACAGTTATTAGGTAGTAGCAGAACTCGAACCTAGTTGTCTTAGGT
CTCGTCTCGTGGACAAAAGGCCAAGCAATCAATTTGACTCTGGAACATAGTAATCTGAGTTTAATGAGTGTTCATAAGTG
GAACATGTTATAAGGGGGTCTAAAATATAGTTAAGCCGGATGGTGAATGACAGAGCATGTTATCTATGAGTAGCATCCTT
GTAGTCCAGTCTGTGTTGACATTCCCTGGTCAAAGTTGTATCTTTGTAGTCTTAAGAAGTTATCGTCTTTGTTGAAACAC
TGTCTTCTGCAAGGATCGACACAATGTTTCCTTTTATCAGGCAAAGCAACCAACATGCTCAGAGATTGTACCTAACGTTT
ATCATTAACTACCAAGGAATAACAGAAAAACAGAGTAGATAATTGATTTGCTAAGCTGAAGTAGCATTCTATATGCATTA
TTCTTAGTGTTCATCTATGTAATATTTGTCAGAGAGCAAGAAGATAAAGAGAAGCCTAAGCAGCTCACAATCTTCTATGG
CGGGAAGGCGCTGGTATTTGACGATTTCTCCACAGACAAGGCAAAGGATCTGATGCAGCTGGCCAGCAAGGGTAGCCCAG
TAGTACAGAACGCTGTTTTGCCTCAACCCTCTGCAACTGCTGCTGTCACTACTGACAAGGCCGTGCTGGACCCAGTCATC

AGCTTGGCTGCTGCTAAGAAGCCTACTCACACAGATACTTCTGGTATGAACACTCTCCCTCTGTTTCCATTCTTACTTAG
TTGAAGTACTAAAATATGATTGCTGGTCTTTGTTATGCCGATTACTGATCGAAACATGTCTTTTCTTTTGTAGATATGCC
TATTATGAGGAAGGCTTCTCTTCACCGCTTCCTTGAGAAGAGAAGGATCGGCAAGTTGCGGTTGCTGTCATTGTTATTTT
CAGTAATGGCTACTTCCTTTTTATCATTGTTGTGTTTGGAACTAATTTGTGCACTTCTGTTTTTCTTCAGCCTCGGTGCA
AAGACCATATCAAACTGCTCCTTCAGATGTAGCACCAGTCAAGGAGACCGAGAGCCAGCCATGGCTCGGATTAGGACCGA
ATGCTGTGAAGTCCAACCTGAATCTGAGCTGTGATTCTGAAATATTTGCAAACAGTTTTCTCCTTCATCATCTTCAGATA
>Zm00001d004173_T001 cDNA
ATGGTTATCCCGTTTGAAACCATGTTTTCGGGCTTCGAATTTTCCATACCCTTCTCGGGCTTGTTTTCCTTCCCATTCTC
AGGCGAGGTCGAGCAACAGAGGACGCTGGCAACAATTAACTTGCCCTCCGGAGCGGAGGGCGAGGAGGCCAAGAAGAGGA
AGGAGAGCATGGAGCTGTTCCCACGGAGCGCCGGCGTCAAGGATGATGCTGCCCCTAGAGAGCAAGAAGATAAAGAGAAG
CCTAAGCAGCTCACAATCTTCTATGGCGGGAAGGCGCTGGTATTTGACGATTTCTCCACAGACAAGGCAAAGGATCTGAT
GCAGCTGGCCAGCAAGGGTAGCCCAGTAGTACAGAACGCTGTTTTGCCTCAACCCTCTGCAACTGCTGCTGTCACTACTG
ACAAGGCCGTGCTGGACCCAGTCATCAGCTTGGCTGCTGCTAAGAAGCCTACTCACACAGATACTTCTGCCTCGGTGCAA
AGACCATATCAAACTGCTCCTTCAGATGTAGCACCAGTCAAGGAGACCGAGAGCCAGCCATGGCTCGGATTAGGACCGAA
TGCTGTGAAGTCCAACCTGAATCTGAGCTGTGATTCTGAAATATTTGCAAACAGTTTTCTCCTTCATCATCTTCAGATA
>Zm00001d004173_P001 peptide
MVIPFETMFSGFEFSIPFSGLFSFPFSGEVEQQRTLATINLPSGAEGEEAKKRKESMELFPRSAGVKDDAAPREQEDKEK
PKQLTIFYGGKALVFDDFSTDKAKDLMQLASKGSPVVQNAVLPQPSATAAVTTDKAVLDPVISLAAAKKPTHTDTSASVQ
RPYQTAPSDVAPVKETESQPWLGLGPNAVKSNLNLSCDSEIFANSFLLHHLQI

ZmTIFY3

B73 RefGen_v3:

GRMZM2G022514

Chr4: 161580030..161583386

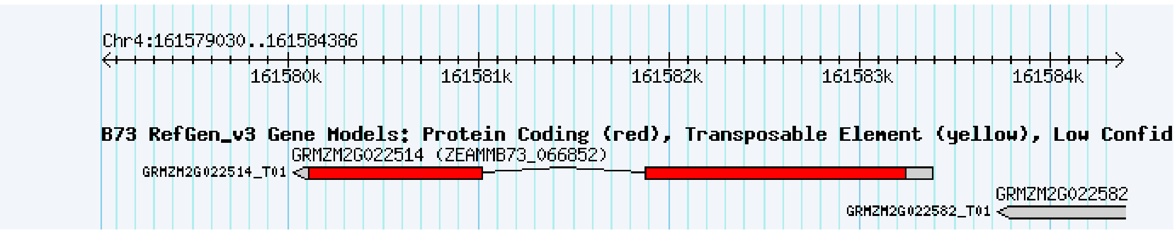

>GRMZM2G022514 Genomic DNA
TCCAAACCGCCCCTTAACCCCTTTGTCCCCGGCGCACACCCCGTCTCCGCAGCACCACACCACCTCCTTCCCCTGCGGGC
TGCGGCTCTGCTGCCTGCCACTCTTCTCTTCTCTTCTCCTCCGCTCCTGCAGAGTGCAGGCCAGCACATGACGACGGGGC
CTTGGCGCTGGTGCCACGGCGTCCGCGCCACGGCCAGCCTGGCGCTGCTCCTGCTGCTCGCGTGCGTGTCCGGCCGCTCC
GTCACGTGCGCGCGAGCGCAGTCCACGGCGTTCACCGGCAGGGAGACCACCAGCTTCTCCTTCCCCAAGTTCGACCAGAA
CCGGCTCCAGCTCTCCACCAACCTGACGTTCACCGGAAACTCCACCGTCAGCCAGGGGGCGCTGCAGGTCACCCCGGACA
GCGGCAACAACTTCAGCACGTACCTCGCCAACCAGGCCGGCCGCGTCTTCTACTCCACGCCCTTCGTGCTCTGGGCCTCC
AACGCGTCCAGCGCCGCCGCCGCCGGCCGCCGCGTCGCGTCCTTCTCCACGGTGTTCCAGTTCAACCTGTACCGCACCAA
CGCGTCCGTCAAGGGCGAGGGGCTGGCGTTCGTCGTCGCGTCCGCGATAGCCGACCCGCCGCCCGGCAGCCACGGCGGGT
TCCTCGGCCTCACCAACGCGTCCACGGACGGGCTCGGCGCCAACGGGTTCGCCGCCGTGGAGCTGGACACGGTGAAGCAG
CCCTACGACCCCGACGGCAACCACATCGGCCTCGACGTCAACGGCGTCCGCTCCTCCAGCGCCACCTGCCCGCTCGCGGC
CCTGGGCATCGAGCTCGCGCCGGCCGACACCGGCGCCAGCGACGGCAGCAACTTCGTCTGGGTCGACTACGACGGCGCCG
CGCGGCGCCTGCGGGCGTACATCTCGCCCAACGCCACCAAGCCGTCCGCCGCCGCGCTCGACGCGTCGCTGGACCTCTCC

GCGGTCGTCGCCGCCAGGGACGCCTACTTCGGCTTCTCCGCGTCCACCGGCGCGGACGACTACCAGCTCAACTGCGTCAA
GATGTGGAACATGACCGTAGAGGTGCTGCACGACGACGACGACAGGCTGCCGAAGAAGCTGTCCGGCTGGAAGCTCGGGC
TGGCCGTCGGCGCGCCGTGCGCCGCCGCGCTGGCGGCGCTCGCGCTGCTCGCGGGCCTGTACCTGATGAAGAAGCGGAGG
AAGGTCGGGGACGACCCGAGCTCCGTGTCCCACAGCGCCATCGACCTGAGGAGCATCCCGGGCGTGCCCAAGGAGTTCGA
CTACGAGGCGCTCAGGAAAGGCACCAACGGCTTCGACGACAAGATGAAGCTGGGGCAGGGCGGGTACGGCGTGGTGTACC
GCGCCACCGTGCCCGGGGACAACGGCCGCAGCATGGAGGTGGCCGTCAAGCAGTTCTCCGGCGCCAACACCAAGGGGCAG
GAGGACTTCCTCGCCGAGCTCGGCATCATCAACCGCCTCCGCCACCGGAATCTCGTCAAGCTCATCGGTAAGGAACTCCT
TGTCATGCTCATTACATTACATTACATCTTACGTATTATTATTTATTATTGCTCTTATAAACTTTTTTATCGTTATCGTC
TGTTATCGTCTTCGTCTTGGCTGCCGTGGCCGTTCTTATCGACAGTAATGTCAAACTTAGTTGGATTGATTAGATATATA
GTAGTAGATCACAGAATTCCAATAAGCACTCCACTGCAAACGCCAAACCGAATCAGAAATACCGGCACGGGGCTTTGGTA
CTGAGACAGCTACGCTTTCATTTCTTGCTGTGGACGGTTCAACGAGACGCTTTCCGCGGTCGTACCTGGAATAGAGAAAT
TAGAGACAACAATTAGCCGTAAATTGCAGCGCTTTAGAGATGCCGATCCCTGTCGTCGACTCGTCGTTACTTGCTTAACA
ACCATTTTCTATTTTCATTGGATGATGGATGCTAGTTGCGTGAATGACAATGAAATGCTTCCAGGCTGCCAAACGCAACC
TGTAAAAAAATGTCAGTTTTTTTTTCTCGTAGGACACCAATGTCAATGGGGAGATAACGAGCGACTATTCAACTCTCAT
TATTCTACAAGATCTCAAGCCAATCGAAACATTATTACTACATGCTCTCGTTGAGATTGAGAGTAAACAATGCTTAGAAT
CGTACGGTACGGACCAGTTGGCGCCTAATCACAAAGATCAAACTAGGACTACTTTGTGGTGAAGGTTTTGGCTATATATT
ATTTAGTTTGGGATCTGCGATCACGGTCGGATCGTGAGCAATGTACCCGGCCGGACGTTTAAAAAACGAGTAAAACAAG
CTTCTGAAACCCAGCAAAGTAATCTGAATGCCACGTCTCTGCAGGCTGGTGCCACCAGGACGGCGTGCTGCTGCTGGTGT
ACGACTACATGCCACACGGCAGCCTGGACAGGCACCTGTTCGGCGGCAGGGAGGCGTCGGCGGCGGCGACGACGACGACG
ACGCTGGACTGGAAGCAGCGCTACAACGTGGTCGCCGGCGTGGCGTCGGCGCTGAACTACCTGCACCACGAGTTCGAGCA
GACGGTGATCCACCGCGACATCAAGCCGTCCAACATCATGCTGGACTCGTCGTTCCACGCGCGGCTGGGCGACTTCGGCC
TGGCGCGCGCGCTCGAGTCCGACAAGACCTCGTACACGGACAAGCTGGGCGTGCCGGGCACGCTGGGGTACATCGCGCCC
GAGTGCTTCCACACGGGGCGGGCCACGCGCGAGTCGGACGTGTTCGGCTTCGGCGCCGTGGTCCTGGAGACCGTCTGCGG
CCGCCGCGTCTCCTGCGACAACCCGGCGGGGTGCAGCCAGCTGCTGGAGCTGGTGTGGAGGCTCCACGGCGCCGGGCGCC
TCCTGGAGGCCGTGGACCCGCGCCTCGCCGCCGGCGGGTACGGGTACGACGGCGAGGAGGCCGAGCGGCTGCTGCTGCTG
GGCCTGGCGTGCAGCCACCCCAACCCGCGGCAGCGGCCCAAGGCGCAGGCCATCCTGCAGAACCTGCAGACGCGGTCCGT
GCCGCCGCTCCCCGTGCCCATGTCCAAGCCGGTGTTCATGTGGCCCGCGCCGCTCGCCGACGGGGAGGAGGACGCGACGC
AGGCGTCGTCCATGTCGCGCAGCGGGGTCACCAGCTCGGGCGTCACCTCCTCGTCCAACTACCCCTACACGTGGTCGTCG
TCGGGGTACAATACCCAGACCTTCCAGGTGAGCAGGGAGGTGCACGACGCGGCGGAGAGGGACGTGGCGACCGTTTGACT
GAGAAGAAGACGGTGTCGAGCTCAGCATGCTGCCGCCGGCAGGACGGCTCACTGACGGTGGTGCACGTGGAGCCCGG
>GRMZM2G022514_T01 cDNA
TCCAAACCGCCCCTTAACCCCTTTGTCCCCGGCGCACACCCCGTCTCCGCAGCACCACACCACCTCCTTCCCCTGCGGGC
TGCGGCTCTGCTGCCTGCCACTCTTCTCTTCTCTTCTCCTCCGCTCCTGCAGAGTGCAGGCCAGCACATGACGACGGGGC
CTTGGCGCTGGTGCCACGGCGTCCGCGCCACGGCCAGCCTGGCGCTGCTCCTGCTGCTCGCGTGCGTGTCCGGCCGCTCC
GTCACGTGCGCGCGAGCGCAGTCCACGGCGTTCACCGGCAGGGAGACCACCAGCTTCTCCTTCCCCAAGTTCGACCAGAA
CCGGCTCCAGCTCTCCACCAACCTGACGTTCACCGGAAACTCCACCGTCAGCCAGGGGGCGCTGCAGGTCACCCCGGACA
GCGGCAACAACTTCAGCACGTACCTCGCCAACCAGGCCGGCCGCGTCTTCTACTCCACGCCCTTCGTGCTCTGGGCCTCC
AACGCGTCCAGCGCCGCCGCCGCCGGCCGCCGCGTCGCGTCCTTCTCCACGGTGTTCCAGTTCAACCTGTACCGCACCAA
CGCGTCCGTCAAGGGCGAGGGGCTGGCGTTCGTCGTCGCGTCCGCGATAGCCGACCCGCCGCCCGGCAGCCACGGCGGGT
TCCTCGGCCTCACCAACGCGTCCACGGACGGGCTCGGCGCCAACGGGTTCGCCGCCGTGGAGCTGGACACGGTGAAGCAG
CCCTACGACCCCGACGGCAACCACATCGGCCTCGACGTCAACGGCGTCCGCTCCTCCAGCGCCACCTGCCCGCTCGCGGC
CCTGGGCATCGAGCTCGCGCCGGCCGACACCGGCGCCAGCGACGGCAGCAACTTCGTCTGGGTCGACTACGACGGCGCCG
CGCGGCGCCTGCGGGCGTACATCTCGCCCAACGCCACCAAGCCGTCCGCCGCCGCGCTCGACGCGTCGCTGGACCTCTCC
GCGGTCGTCGCCGCCAGGGACGCCTACTTCGGCTTCTCCGCGTCCACCGGCGCGGACGACTACCAGCTCAACTGCGTCAA

GATGTGGAACATGACCGTAGAGGTGCTGCACGACGACGACGACAGGCTGCCGAAGAAGCTGTCCGGCTGGAAGCTCGGGC
TGGCCGTCGGCGCGCCGTGCGCCGCCGCGCTGGCGGCGCTCGCGCTGCTCGCGGGCCTGTACCTGATGAAGAAGCGGAGG
AAGGTCGGGGACGACCCGAGCTCCGTGTCCCACAGCGCCATCGACCTGAGGAGCATCCCGGGCGTGCCCAAGGAGTTCGA
CTACGAGGCGCTCAGGAAAGGCACCAACGGCTTCGACGACAAGATGAAGCTGGGGCAGGGCGGGTACGGCGTGGTGTACC
GCGCCACCGTGCCCGGGGACAACGGCCGCAGCATGGAGGTGGCCGTCAAGCAGTTCTCCGGCGCCAACACCAAGGGGCAG
GAGGACTTCCTCGCCGAGCTCGGCATCATCAACCGCCTCCGCCACCGGAATCTCGTCAAGCTCATCGGCTGGTGCCACCA
GGACGGCGTGCTGCTGCTGGTGTACGACTACATGCCACACGGCAGCCTGGACAGGCACCTGTTCGGCGGCAGGGAGGCGT
CGGCGGCGGCGACGACGACGACGACGCTGGACTGGAAGCAGCGCTACAACGTGGTCGCCGGCGTGGCGTCGGCGCTGAAC
TACCTGCACCACGAGTTCGAGCAGACGGTGATCCACCGCGACATCAAGCCGTCCAACATCATGCTGGACTCGTCGTTCCA
CGCGCGGCTGGGCGACTTCGGCCTGGCGCGCGCGCTCGAGTCCGACAAGACCTCGTACACGGACAAGCTGGGCGTGCCGG
GCACGCTGGGGTACATCGCGCCCGAGTGCTTCCACACGGGGCGGGCCACGCGCGAGTCGGACGTGTTCGGCTTCGGCGCC
GTGGTCCTGGAGACCGTCTGCGGCCGCCGCGTCTCCTGCGACAACCCGGCGGGGTGCAGCCAGCTGCTGGAGCTGGTGTG
GAGGCTCCACGGCGCCGGGCGCCTCCTGGAGGCCGTGGACCCGCGCCTCGCCGCCGGCGGGTACGGGTACGACGGCGAGG
AGGCCGAGCGGCTGCTGCTGCTGGGCCTGGCGTGCAGCCACCCCAACCCGCGGCAGCGGCCCAAGGCGCAGGCCATCCTG
CAGAACCTGCAGACGCGGTCCGTGCCGCCGCTCCCCGTGCCCATGTCCAAGCCGGTGTTCATGTGGCCCGCGCCGCTCGC
CGACGGGGAGGAGGACGCGACGCAGGCGTCGTCCATGTCGCGCAGCGGGGTCACCAGCTCGGGCGTCACCTCCTCGTCCA
ACTACCCCTACACGTGGTCGTCGTCGGGGTACAATACCCAGACCTTCCAGGTGAGCAGGGAGGTGCACGACGCGGCGGAG
AGGGACGTGGCGACCGTTTGACTGAGAAGAAGACGGTGTCGAGCTCAGCATGCTGCCGCCGGCAGGACGGCTCACTGACG
GTGGTGCACGTGGAGCCCGG

>GRMZM2G022514_P01 peptide

MTTGPWRWCHGVRATASLALLLLLACVSGRSVTCARAQSTAFTGRETTSFSFPKFDQNRLQLSTNLTFTGNSTVSQGALQ
VTPDSGNNFSTYLANQAGRVFYSTPFVLWASNASSAAAAGRRVASFSTVFQFNLYRTNASVKGEGLAFVVASAIADPPPG
SHGGFLGLTNASTDGLGANGFAAVELDTVKQPYDPDGNHIGLDVNGVRSSSATCPLAALGIELAPADTGASDGSNFVWVD
YDGAARRLRAYISPNATKPSAAALDASLDLSAVVAARDAYFGFSASTGADDYQLNCVKMWNMTVEVLHDDDDRLPKKLSG
WKLGLAVGAPCAAALAALALLAGLYLMKKRRKVGDDPSSVSHSAIDLRSIPGVPKEFDYEALRKGTNGFDDKMKLGQGGY
GVVYRATVPGDNGRSMEVAVKQFSGANTKGQEDFLAELGIINRLRHRNLVKLIGWCHQDGVLLLVYDYMPHGSLDRHLFG
GREASAAATTTTTLDWKQRYNVVAGVASALNYLHHEFEQTVIHRDIKPSNIMLDSSFHARLGDFGLARALESDKTSYTDK
LGVPGTLGYIAPECFHTGRATRESDVFGFGAVVLETVCGRRVSCDNPAGCSQLLELVWRLHGAGRLLEAVDPRLAAGGYG
YDGEEAERLLLLGLACSHPNPRQRPKAQAILQNLQTRSVPPLPVPMSKPVFMWPAPLADGEEDATQASSMSRSGVTSSGV
TSSSNYPYTWSSSGYNTQTFQVSREVHDAAERDVATV

AGPv4:

Zm00001d051615

Chr4: 164593515..164603165

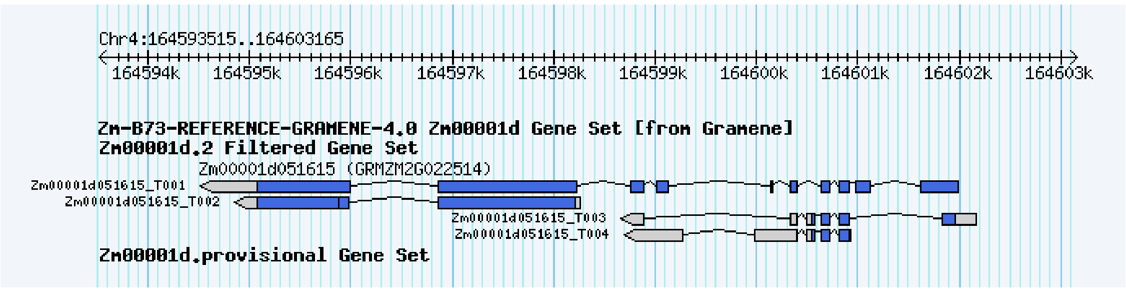

>Zm00001d051615 Genomic DNA

AGCCAGAGAGCGTCACCTTGGACCCTCAGGGTCGCGAGCCATACACGGGCGTCGCTGACGACACCACGCTGCCAACCCTA
GCCGCGCCTGCCTTCCTCCCCCGCTTGCCGTCGAGTATCGATGTTTGGAGGTGGGGGCGCCTTCGCCGGTGAGGTACCTG
GTGGGGGCAGCGATCATGATGAGCGGCGTGGTGCCTTCCTCCCTCCATGTCCTCCTCCGCGCCGAGCGCGCCCTCCGCGA

ACGCGACGTCGGCGAGGTCCACATCCCGCTGTCCGAGCTCCTCTCCGGCGCCCCCGACGGCCCCGTCCCCGCCAAGTTCG
TCGCCTACCAGGTGCGCAAGATCTCCTCCGGCAAGCCCCAGGGAGTCCTCAACCTCTCGTACAAGCTCGGCGAGGTCGCC
AACGGCTACGCCCCCGCCCCGCCGCCCAGTCCGCCTACGCCCAGCCTCCACCGACCGCCGCGTACCCGCCTCCTTCAGGC
AAGGCTGATGCCTACCCGCCTCCCGCCGCGTACCCGCCTCCTTCAGGCAAGGCCGACGCGTACGCCCCCCTGCCACCGCA
TACCCGCCGGCAGCCAAGGCCGACGCGTACCCACCTCCATACGGCGCCTACCCGCCCGCCGGCGGCAAGGCCGACGCTGC
TTACCCGCCCTCATCCGGCTACCCACCTGCGGCAGGCAAGCCAGGCAAGGGGGGCGAACCGGTGACGGCTTACCCTTCGG
CCGGTCCCAGCACGGCGGCGCCCTACGCTAGGTTCTTCAGAAGCATTTATTTGTTTTGCCACGGTAGTGCTAGAACAATA
GTGTTATCGTTCTGCTATTTTAGTTCCATTTGTTTCCTGGTTAGTTGTTATCCTCCAGGCTCTGAACAATAGGGACATCT
GAATTAATCAGATTCCAGGAAAAATCGTTGTGACTAGATCCGATATCTGTATCACTAAGGCCTACAAATGATTTGAACGC
CACTGTCCAGAAAGCCAATCCGTTCACTGGCCAAGTAGACATACCTTTCTATCAGAATTTTACCTCTGTTTTTTAATCAT
TTTAGGAAGTAGGACGATTGCTTTCTACCCCCGTGTCCGCGCACACATTCTATCCTATGCCGGTTTTGAACCCACCACAT
GGCTTTCTGATATGCTTTTATCTGAACCAGTAGTGGGAGTGACATGGATCTGCCTACTTTCCATTCAGAGGTGCTGTTTT
ATCTGCCTTTATGTGTTCTTATTTTTTTGCATTCTTTACTACAAGGTCTGCAATGTTGCCGATGTCGAACCCTTCAGCTA
ATCCCACACAGCTTACAATTTTCTATGGTGGATCAGTATGTGTGTATGACTCGGTGCCACCAGAAAAGGTAAATATTATC
TTCACGCCATTTACAGATATCTTGTCCTTCTGTACCATCTGTTAAAATACACATGCTAATACACTAATTTGGCATTGACA
GGCTCAGGCAATCATGCTTATAGCTGCAGCTGCGGCAGCTGCGGCAGCCACCAAAGGCAGTGCTGCCACTGCTTTTAATC
CTCCAATGGTACATACAGCCACTGTCGCCCCAGCAGCAGTCTTCTCTCCTGTGCTTACACGGTCTCCATCACTGCAGAGC
ACTTCTGTAGCAGCTGGACAAGCTTAGGTTGTTGCTGACCCTAGCTCAATAAGCAAGCTTCAGGCTGGTAAGACCACTGC
ATCTTTGTGTAAAAATCCAGAAATGGAATACCTTTTCCAGTGTTGACATTGTCCAACACGTCTTTAAACTATCTTGATCT
GTTGGCCAGATCTCCCCATTGCCAGGAGGCACTCTCTCCAGCGCTTCCTTGAGAAACGTCGTGACAGGTTAGCATGATAC
ATGAAAGCAACAGGGCTCTGATATTCTGAATCTTATTGTTAGGCCTCTTATTCTACAATCTACATGTTTAAAACTTTTGT
TATAAACTGCTGTGTGTGGTACCACACTAATTTTTTCCCCGACTATTGGCACCACAGTAATCAACAGTATATTTTGTACC
GTATTCTGTTTTTTGTCCTCCAGGCAGGGTAAAAATAAAATAAAATTTGCTGTTATAGCATTTAAATGATTGCACTTTCT
GCTGACAAGTGCTCAATGACATCCCTAAAAAAATCTATAATTTCTTCACAGAAAGATTGGTAGAGAGCCTGCTTCCATAA
TACAACATATAGCTGCAAATAGCCTTGGATCTAGAGATGGCAACGGGTACAAACCCGCTGGGTTTTGCTGTCCCAAACCC
GTACCCGTGAAAAATATCTATGCCCATTAAAAAACCCGTACCCATGACGGGTTTGAGATTTTGCCCAAACCCGTACCCAT
CGGGTTAACGGGTACCCATGGGTTACCCGCGGGTTTCATCTCCAATATACTTATCTTCTCATAATCAATAAGTATCGTAA
TGATTAATGATATCATGATCCAAAATCTATGTAATGAACAACGAGTTCATGATTTGGTATAAAAATTATTAGTAGAGATA
ATGAAATACAAATAATAAGTTGTATAATTAAGTGACCTTACACTAAGTTATTCATCCACCACATATATAACGCTAGTAAA
AACTATAATAGCAAGCAAGCAACACTCTCACTGACTACTGATACATTCACCTATTGGTAAAAATTATGAAGTAAATAAGG
AATAACAAGTTTTTTGTTCGTTTATAAAATAAAATGACAATATACACTAGGTTTGGTCGGGTTTAAAAAACCCACGGGTT
CACGAGTTTGGGTACTATAGGAACAAACCCGTACCCATAAACCCGCTGGGTACAGATTTATGTCCATTAACAAACCCATG
GGTATGAAAATTAGCCCAAACCCGTACCCTAATGGGGTAAAAACCCATCGGGTTTCGGGTTTCGGGTACCCATTGCCATC
TCTACTTGGATCATGTAACTCCCTTGAATTTTTTTTTTAAAAATCTGCAAATCGATATTAACACAAGACAGAGTTCTGGG
ACCAGCAGTAGATACAAAAACCAACATTACCCCCATTTAGCTGCTCCAAATACCATGTTTTCCCCTGTTAGGAAAATAAC
ATATCTGAGAATCGGGATGTTCCTGTGTATTATCCTGTTCTATTTGGAAAAGCTGGCCACTTAAAGATCCAGTCTCAAGG
TTGCAAACTAGCACCATTTTTTTTTCTGATGTAAGCTTTAGATGCCTGTTTTTGCCTTATCTTAGAACTGAGTTTAGAAT
TGTTTCTTAGTATAACTTTTTTTTATCGTGGATCTACTGATTTCAAGTGATTAAATAAATGATGGCACGAAACAGGGTCG
TGAGCAAAGCTCCGTACAGCCCCGCCAAGTCGTTCGATGGCATGGAGTCAGCGGGAATGGAGATGACTGTGGACGGCAAG
CAGGGTGCAAGGCCCAGTATCTTGAAAGGTTGGGGAGCCGCGAAGTCTGAAGTCTGAACGGTGAAAGCGTAGCCCCGCGG
GAGCGTGGCGGTGTCTGGGAAGCGCCAGAAGCCGAGGTAGAGGGTGCAGAGGGAGGCAAGGTGGAGAACGTTGATGAGGC
AATTGACTCCGTAGCAACGCACGGGCATACAACTAGTGTGAATGAAGAGAGAGAGAGCCAATGAGAGTCCCTCCGTCTGC
TGGTGTTCACTTGTTTGCTCAAACAAGCTTCTGATCTGTGGTCCCAAATTCTGGCTCTGGCGTAGTCACCGTCTCATGGG
GTTATGTTGTTAGGCGCGTCACTAGGAAGGCCACAGAGTTTCTGAGAACAGAGAACCTGATGTGGGGAATAGCTGAATAA

GATACAGTTCAAAACGGAGGAGGGAGTGTCAATTTATAACGTGGATTCCAAACCGCCCCTTAACCCCTTTGTCCCCGGCG
CACACCCCGTCTCCGCAGCACCACACCACCTCCTTCCCCTGCGGGCTGCGGCTCTGCTGCCTGCCACTCTTCTCTTCTCT
TCTCCTCCGCTCCTGCAGAGTGCAGGCCAGCACATGACGACGGGGCCTTGGCGCTGGTGCCACGGCGTCCGCGCCACGGC
CAGCCTGGCGCTGCTCCTGCTGCTCGCGTGCGTGTCCGGCCGCTCCGTCACGTGCGCGCGAGCGCAGTCCACGGCGTTCA
CCGGCAGGGAGACCACCAGCTTCTCCTTCCCCAAGTTCGACCAGAACCGGCTCCAGCTCTCCACCAACCTGACGTTCACC
GGAAACTCCACCGTCAGCCAGGGGGCGCTGCAGGTCACCCCGGACAGCGGCAACAACTTCAGCACGTACCTCGCCAACCA
GGCCGGCCGCGTCTTCTACTCCACGCCCTTCGTGCTCTGGGCCTCCAACGCGTCCAGCGCCGCCGCCGCCGGCCGCCGCG
TCGCGTCCTTCTCCACGGTGTTCCAGTTCAACCTGTACCGCACCAACGCGTCCGTCAAGGGCGAGGGGCTGGCGTTCGTC
GTCGCGTCCGCGATAGCCGACCCGCCGCCCGGCAGCCACGGCGGGTTCCTCGGCCTCACCAACGCGTCCACGGACGGGCT
CGGCGCCAACGGGTTCGCCGCCGTGGAGCTGGACACGGTGAAGCAGCCCTACGACCCCGACGGCAACCACATCGGCCTCG
ACGTCAACGGCGTCCGCTCCTCCAGCGCCACCTGCCCGCTCGCGGCCCTGGGCATCGAGCTCGCGCCGGCCGACACCGGC
GCCAGCGACGGCAGCAACTTCGTCTGGGTCGACTACGACGGCGCCGCGCGGCGCCTGCGGGCGTACATCTCGCCCAACGC
CACCAAGCCGTCCGCCGCCGCGCTCGACGCGTCGCTGGACCTCTCCGCGGTCGTCGCCGCCAGGGACGCCTACTTCGGCT
TCTCCGCGTCCACCGGCGCGGACGACTACCAGCTCAACTGCGTCAAGATGTGGAACATGACCGTAGAGGTGCTGCACGAC
GACGACGACAGGCTGCCGAAGAAGCTGTCCGGCTGGAAGCTCGGGCTGGCCGTCGGCGCGCCGTGCGCCGCCGCGCTGGC
GGCGCTCGCGCTGCTCGCGGGCCTGTACCTGATGAAGAAGCGGAGGAAGGTCGGGGACGACCCGAGCTCCGTGTCCCACA
GCGCCATCGACCTGAGGAGCATCCCGGGCGTGCCCAAGGAGTTCGACTACGAGGCGCTCAGGAAAGGCACCAACGGCTTC
GACGACAAGATGAAGCTGGGGCAGGGCGGGTACGGCGTGGTGTACCGCGCCACCGTGCCCGGGGACAACGGCCGCAGCAT
GGAGGTGGCCGTCAAGCAGTTCTCCGGCGCCAACACCAAGGGGCAGGAGGACTTCCTCGCCGAGCTCGGCATCATCAACC
GCCTCCGCCACCGGAATCTCGTCAAGCTCATCGGTAAGGAACTCCTTGTCATGCTCATTACATTACATTACATCTTACGT
ATTATTATTTATTATTGCTCTTATAAACTTTTTTATCGTTATCGTCTGTTATCGTCTTCGTCTTGGCTGCCGTGGCCGTT
CTTATCGACAGTAATGTCAAACTTAGTTGGATTGATTAGATATATAGTAGTAGATCACAGAATTCCAATAAGCACTCCAC
TGCAAACGCCAAACCGAATCAGAAATACCGGCACGGGGCTTTGGTACTGAGACAGCTACGCTTTCATTTCTTGCTGTGGA
CGGTTCAACGAGACGCTTTCCGCGGTCGTACCTGGAATAGAGAAATTAGAGACAACAATTAGCCGTAAATTGCAGCGCTT
TAGAGATGCCGATCCCTGTCGTCGACTCGTCGTTACTTGCTTAACAACCATTTTCTATTTTCATTGGATGATGGATGCTA
GTTGCGTGAATGACAATGAAATGCTTCCAGGCTGCCAAACGCAACCTGTAAAAAAATGTCAGTTTTTTTTTTCTCGTAGG
ACACCAATGTCAATGGGGAGATAACGAGCGACTATTCAACTCTCATTATTCTACAAGATCTCAAGCCAATCGAAACATTA
TTACTACATGCTCTCGTTGAGATTGAGAGTAAACAATGCTTAGAATCGTACGGTACGGACCAGTTGGCGCCTAATCACAA
AGATCAAACTAGGACTACTTTGTGGTGAAGGTTTTGGCTATATATTATTTTAGTTTGGGATCTGCGATCACGGTCGGATC
GTGAGCAATGTACCCGGCCGGACGTTTAAAAAACGAGTAAAACAAGCTTCTGAAACCCAGCAAAGTAATCTGAATGCCAC
GTCTCTGCAGGCTGGTGCCACCAGGACGGCGTGCTGCTGCTGGTGTACGACTACATGCCACACGGCAGCCTGGACAGGCA
CCTGTTCGGCGGCAGGGAGGCGTCGGCGGCGGCGACGACGACGACGACGCTGGACTGGAAGCAGCGCTACAACGTGGTCG
CCGGCGTGGCGTCGGCGCTGAACTACCTGCACCACGAGTTCGAGCAGACGGTGATCCACCGCGACATCAAGCCGTCCAAC
ATCATGCTGGACTCGTCGTTCCACGCGCGGCTGGGCGACTTCGGCCTGGCGCGCGCGCTCGAGTCCGACAAGACCTCGTA
CACGGACAAGCTGGGCGTGCCGGGCACGCTGGGGTACATCGCGCCCGAGTGCTTCCACACGGGGCGGGCCACGCGCGAGT
CGGACGTGTTCGGCTTCGGCGCCGTGGTCCTGGAGACCGTCTGCGGCCGCCGCGTCTCCTGCGACAACCCGGCGGGGTGC
AGCCAGCTGCTGGAGCTGGTGTGGAGGCTCCACGGCGCCGGGCGCCTCCTGGAGGCCGTGGACCCGCGCCTCGCCGCCGG
CGGGTACGGGTACGACGGCGAGGAGGCCGAGCGGCTGCTGCTGCTGGGCCTGGCGTGCAGCCACCCCAACCCGCGGCAGC
GGCCCAAGGCGCAGGCCATCCTGCAGAACCTGCAGACGCGGTCCGTGCCGCCGCTCCCCGTGCCCATGTCCAAGCCGGTG
TTCATGTGGCCCGCGCCGCTCGCCGACGGGGAGGAGGACGCGACGCAGGCGTCGTCCATGTCGCGCAGCGGGGTCACCAG
CTCGGGCGTCACCTCCTCGTCCAACTACCCCTACACGTGGTCGTCGTCGGGGTACAATACCCAGACCTTCCAGGTGAGCA
GGGAGGTGCACGACGCGGCGGAGAGGGACGTGGCGACCGTTTGACTGAGAAGAAGACGGTGTCGAGCTCAGCATGCTGCC
GCCGGCAGGACGGCTCACTGACGGTGGTGCACGTGGAGCCCGGGGGGCGGGCCAGTCGGCAGTCGGGTTGCGCGCTTTCA
TCAGGAGTCAAAAGGGGCTTGTGGCAACGCGGCTCACGATCGAATCGGACAAAATAAAGACGCCAACAAAGCGCACGCGA

CAACGCGGGCAGGGTTTTCGTCGTCGGAGAGCCTTTTCCGTTGCAGCTTTGAGTGGGTAGCCAAGTGGGTCCTTTTTTCT
GCAGCTGTTGCTTGGTTTGCTTCTTGGAAGCCATCACAGTTTGGTGTAGATAGCCGCCACATTGTCAGCATAAAGATGAT
ATGCTGACACACATCTCCGTGTTTGTCACATTGAGCATTCAGCAGAGCCTGCACCAACCCAGCGATTCTCCAAACACAGA
CAGTGCACTGCTCTCGTCTGGACTGGTGCTCTCTCTGGAAAACTCTGCATTCCTCCTTCCACAATTCCCTAACGGTTCAA
GATCGTGTCTCAGTGTATCCACTCAACTCGCAGGATCAAGTTCGCGGTGAT
>Zm00001d051615_T001 cDNA
Chr4: 164594515..164601990
ATGATGAGCGGCGTGGTGCCTTCCTCCCTCCATGTCCTCCTCCGCGCCGAGCGCGCCCTCCGCGAACGCGACGTCGGCGA
GGTCCACATCCCGCTGTCCGAGCTCCTCTCCGGCGCCCCCGACGGCCCCGTCCCCGCCAAGTTCGTCGCCTACCAGGTGC
GCAAGATCTCCTCCGGCAAGCCCCAGGGAGTCCTCAACCTCTCGTACAAGCTCGGCGAGGTCGCCAACGGCTACGCCCCC
GCCCCGCCGCCCAGTCCGCCTACGCCCAGCCTCCACCGACCGCCGCGTACCCGCCTCCTTCAGGCAAGGCTGATGCCTAC
CCGCCTCCCGCCGCGTACCCGCCTCCTTCAGGCAAGGCCGACGCGAAGTAGGACGATTGCTTTCTACCCCCGTGTCCGCG
CACACATTCTATCCTATGCCGGTTTTGAACCCACCACATGGCTTTCTGATATGCTTTTATCTGAACCAGTAGTGGGAGTG
ACATGGATCTGCCTACTTTCCATTCAGAGGTCTGCAATGTTGCCGATGTCGAACCCTTCAGCTAATCCCACACAGCTTAC
AATTTTCTATGGTGGATCAGTATGTGTGTATGACTCGGTGCCACCAGAAAAGGCTCAGGCAATCATGCTTATAGCTGCAG
CTGCGGCAGCTGCGGCAGCCACCAAAGGCAGTGCTGCCACTGCTTTTAATCCTCCAATGATCTCCCCATTGCCAGGAGGC
ACTCTCTCCAGCGCTTCCTTGAGAAACGTCGTGACAGGCAGGGAAAATAACATATCTGAGAATCGGGATGTTCCTGTGTA
TTATCCTGTTCTATTTGGAAAAGCTGGCCACTTAAAGATCCAGTCTCAAGGTTGCAAACTAGCACCATTTTTTTTTCTGA
TGGTCGTGAGCAAAGCTCCGTACAGCCCCGCCAAGTCGTTCGATGGCATGGAGTCAGCGGGAATGGAGATGACTGTGGAC
GGCAAGCAGGGTGCAAGGCCCAGTATCTTGAAAGGTTGGGGAGCCGCGAAGTCTGAAAGTGCAGGCCAGCACATGACGAC
GGGGCCTTGGCGCTGGTGCCACGGCGTCCGCGCCACGGCCAGCCTGGCGCTGCTCCTGCTGCTCGCGTGCGTGTCCGGCC
GCTCCGTCACGTGCGCGCGAGCGCAGTCCACGGCGTTCACCGGCAGGGAGACCACCAGCTTCTCCTTCCCCAAGTTCGAC
CAGAACCGGCTCCAGCTCTCCACCAACCTGACGTTCACCGGAAACTCCACCGTCAGCCAGGGGGCGCTGCAGGTCACCCC
GGACAGCGGCAACAACTTCAGCACGTACCTCGCCAACCAGGCCGGCCGCGTCTTCTACTCCACGCCCTTCGTGCTCTGGG
CCTCCAACGCGTCCAGCGCCGCCGCCGCCGGCCGCCGCGTCGCGTCCTTCTCCACGGTGTTCCAGTTCAACCTGTACCGC
ACCAACGCGTCCGTCAAGGGCGAGGGGCTGGCGTTCGTCGTCGCGTCCGCGATAGCCGACCCGCCGCCCGGCAGCCACGG
CGGGTTCCTCGGCCTCACCAACGCGTCCACGGACGGGCTCGGCGCCAACGGGTTCGCCGCCGTGGAGCTGGACACGGTGA
AGCAGCCCTACGACCCCGACGGCAACCACATCGGCCTCGACGTCAACGGCGTCCGCTCCTCCAGCGCCACCTGCCCGCTC
GCGGCCCTGGGCATCGAGCTCGCGCCGGCCGACACCGGCGCCAGCGACGGCAGCAACTTCGTCTGGGTCGACTACGACGG
CGCCGCGCGGCGCCTGCGGGCGTACATCTCGCCCAACGCCACCAAGCCGTCCGCCGCCGCGCTCGACGCGTCGCTGGACC
TCTCCGCGGTCGTCGCCGCCAGGGACGCCTACTTCGGCTTCTCCGCGTCCACCGGCGCGGACGACTACCAGCTCAACTGC
GTCAAGATGTGGAACATGACCGTAGAGGTGCTGCACGACGACGACGACAGGCTGCCGAAGAAGCTGTCCGGCTGGAAGCT
CGGGCTGGCCGTCGGCGCGCCGTGCGCCGCCGCGCTGGCGGCGCTCGCGCTGCTCGCGGGCCTGTACCTGATGAAGAAGC
GGAGGAAGGTCGGGGACGACCCGAGCTCCGTGTCCCACAGCGCCATCGACCTGAGGAGCATCCCGGGCGTGCCCAAGGAG
TTCGACTACGAGGCGCTCAGGAAAGGCACCAACGGCTTCGACGACAAGATGAAGCTGGGGCAGGGCGGGTACGGCGTGGT
GTACCGCGCCACCGTGCCCGGGGACAACGGCCGCAGCATGGAGGTGGCCGTCAAGCAGTTCTCCGGCGCCAACACCAAGG
GGCAGGAGGACTTCCTCGCCGAGCTCGGCATCATCAACCGCCTCCGCCACCGGAATCTCGTCAAGCTCATCGGCTGGTGC
CACCAGGACGGCGTGCTGCTGCTGGTGTACGACTACATGCCACACGGCAGCCTGGACAGGCACCTGTTCGGCGGCAGGGA
GGCGTCGGCGGCGGCGACGACGACGACGACGCTGGACTGGAAGCAGCGCTACAACGTGGTCGCCGGCGTGGCGTCGGCGC
TGAACTACCTGCACCACGAGTTCGAGCAGACGGTGATCCACCGCGACATCAAGCCGTCCAACATCATGCTGGACTCGTCG
TTCCACGCGCGGCTGGGCGACTTCGGCCTGGCGCGCGCGCTCGAGTCCGACAAGACCTCGTACACGGACAAGCTGGGCGT
GCCGGGCACGCTGGGGTACATCGCGCCCGAGTGCTTCCACACGGGGCGGGCCACGCGCGAGTCGGACGTGTTCGGCTTCG
GCGCCGTGGTCCTGGAGACCGTCTGCGGCCGCCGCGTCTCCTGCGACAACCCGGCGGGGTGCAGCCAGCTGCTGGAGCTG
GTGTGGAGGCTCCACGGCGCCGGGCGCCTCCTGGAGGCCGTGGACCCGCGCCTCGCCGCCGGCGGGTACGGGTACGACGG

CGAGGAGGCCGAGCGGCTGCTGCTGCTGGGCCTGGCGTGCAGCCACCCCAACCCGCGGCAGCGGCCCAAGGCGCAGGCCA
TCCTGCAGAACCTGCAGACGCGGTCCGTGCCGCCGCTCCCCGTGCCCATGTCCAAGCCGGTGTTCATGTGGCCCGCGCCG
CTCGCCGACGGGGAGGAGGACGCGACGCAGGCGTCGTCCATGTCGCGCAGCGGGGTCACCAGCTCGGGCGTCACCTCCTC
GTCCAACTACCCCTACACGTGGTCGTCGTCGGGGTACAATACCCAGACCTTCCAGGTGAGCAGGGAGGTGCACGACGCGG
CGGAGAGGGACGTGGCGACCGTTTGACTGAGAAGAAGACGGTGTCGAGCTCAGCATGCTGCCGCCGGCAGGACGGCTCAC
TGACGGTGGTGCACGTGGAGCCCGGGGGGCGGGCCAGTCGGCAGTCGGGTTGCGCGCTTTCATCAGGAGTCAAAAGGGGC
TTGTGGCAACGCGGCTCACGATCGAATCGGACAAAATAAAGACGCCAACAAAGCGCACGCGACAACGCGGGCAGGGTTTT
CGTCGTCGGAGAGCCTTTTCCGTTGCAGCTTTGAGTGGGTAGCCAAGTGGGTCCTTTTTTCTGCAGCTGTTGCTTGGTTT
GCTTCTTGGAAGCCATCACAGTTTGGTGTAGATAGCCGCCACATTGTCAGCATAAAGATGATATGCTGACACACATCTCC
GTGTTTGTCACATTGAGCATTCAGCAGAGCCTGCACCAACCCAGCGATTCTCCAAACACAGACAGTGCACTGCTCTCGTC
TGGACTGGTGCTCTCTCTGGAAAACTCTGCATTCCTCCTTCCACAATTCCCTAACGGTTCAAGATCGTGTCTCAGTGTAT
CCACTCAACTCGCAGGATCAAGTTCGCGGTGAT
>Zm00001d051615_P001 peptide
MMSGVVPSSLHVLLRAERALRERDVGEVHIPLSELLSGAPDGPVPAKFVAYQVRKISSGKPQGVLNLSYKLGEVANGYAP
APPPSPPTPSLHRPPRTRLLQARLMPTRLPPRTRLLQARPTRSRTIAFYPRVRAHILSYAGFEPTTWLSDMLLSEPVVGV
TWICLLSIQRSAMLPMSNPSANPTQLTIFYGGSVCVYDSVPPEKAQAIMLIAAAAAAAAATKGSAATAFNPPMISPLPGG
TLSSASLRNVVTGRENNISENRDVPVYYPVLFGKAGHLKIQSQGCKLAPFFFLMVVSKAPYSPAKSFDGMESAGMEMTVD
GKQGARPSILKGWGAAKSESAGQHMTTGPWRWCHGVRATASLALLLLLACVSGRSVTCARAQSTAFTGRETTSFSFPKFD
QNRLQLSTNLTFTGNSTVSQGALQVTPDSGNNFSTYLANQAGRVFYSTPFVLWASNASSAAAAGRRVASFSTVFQFNLYR
TNASVKGEGLAFVVASAIADPPPGSHGGFLGLTNASTDGLGANGFAAVELDTVKQPYDPDGNHIGLDVNGVRSSSATCPL
AALGIELAPADTGASDGSNFVWVDYDGAARRLRAYISPNATKPSAAALDASLDLSAVVAARDAYFGFSASTGADDYQLNC
VKMWNMTVEVLHDDDDRLPKKLSGWKLGLAVGAPCAAALAALALLAGLYLMKKRRKVGDDPSSVSHSAIDLRSIPGVPKE
FDYEALRKGTNGFDDKMKLGQGGYGVVYRATVPGDNGRSMEVAVKQFSGANTKGQEDFLAELGIINRLRHRNLVKLIGWC
HQDGVLLLVYDYMPHGSLDRHLFGGREASAAATTTTTLDWKQRYNVVAGVASALNYLHHEFEQTVIHRDIKPSNIMLDSS
FHARLGDFGLARALESDKTSYTDKLGVPGTLGYIAPECFHTGRATRESDVFGFGAVVLETVCGRRVSCDNPAGCSQLLEL
VWRLHGAGRLLEAVDPRLAAGGYGYDGEEAERLLLLGLACSHPNPRQRPKAQAILQNLQTRSVPPLPVPMSKPVFMWPAP
LADGEEDATQASSMSRSGVTSSGVTSSSNYPYTWSSSGYNTQTFQVSREVHDAAERDVATV
>Zm00001d051615_T002 cDNA
Chr4: 164594848..164598254
CTCTTCTCTTCTCCTCCGCTCCTGCAGAGTGCAGGCCAGCACATGACGACGGGGCCTTGGCGCTGGTGCCACGGCGTCCG
CGCCACGGCCAGCCTGGCGCTGCTCCTGCTGCTCGCGTGCGTGTCCGGCCGCTCCGTCACGTGCGCGCGAGCGCAGTCCA
CGGCGTTCACCGGCAGGGAGACCACCAGCTTCTCCTTCCCCAAGTTCGACCAGAACCGGCTCCAGCTCTCCACCAACCTG
ACGTTCACCGGAAACTCCACCGTCAGCCAGGGGGCGCTGCAGGTCACCCCGGACAGCGGCAACAACTTCAGCACGTACCT
CGCCAACCAGGCCGGCCGCGTCTTCTACTCCACGCCCTTCGTGCTCTGGGCCTCCAACGCGTCCAGCGCCGCCGCCGCCG
GCCGCCGCGTCGCGTCCTTCTCCACGGTGTTCCAGTTCAACCTGTACCGCACCAACGCGTCCGTCAAGGGCGAGGGGCTG
GCGTTCGTCGTCGCGTCCGCGATAGCCGACCCGCCGCCCGGCAGCCACGGCGGGTTCCTCGGCCTCACCAACGCGTCCAC
GGACGGGCTCGGCGCCAACGGGTTCGCCGCCGTGGAGCTGGACACGGTGAAGCAGCCCTACGACCCCGACGGCAACCACA
TCGGCCTCGACGTCAACGGCGTCCGCTCCTCCAGCGCCACCTGCCCGCTCGCGGCCCTGGGCATCGAGCTCGCGCCGGCC
GACACCGGCGCCAGCGACGGCAGCAACTTCGTCTGGGTCGACTACGACGGCGCCCGCGCGGCGCCTGCGGGCGTACATCTC
GCCCAACGCCACCAAGCCGTCCGCCGCCGCGCTCGACGCGTCGCTGGACCTCTCCGCGGTCGTCGCCGCCAGGGACGCCT
ACTTCGGCTTCTCCGCGTCCACCGGCGCGGACGACTACCAGCTCAACTGCGTCAAGATGTGGAACATGACCGTAGAGGTG
CTGCACGACGACGACGACAGGCTGCCGAAGAAGCTGTCCGGCTGGAAGCTCGGGCTGGCCGTCGGCGCGCCGTGCGCCGC
CGCGCTGGCGGCGCTCGCGCTGCTCGCGGGCCTGTACCTGATGAAGAAGCGGAGGAAGGTCGGGGACGACCCGAGCTCCG
TGTCCCACAGCGCCATCGACCTGAGGAGCATCCCGGGCGTGCCCAAGGAGTTCGACTACGAGGCGCTCAGGAAAGGCACC

AACGGCTTCGACGACAAGATGAAGCTGGGGCAGGGCGGGTACGGCGTGGTGTACCGCGCCACCGTGCCCGGGGACAACGG
CCGCAGCATGGAGGTGGCCGTCAAGCAGTTCTCCGGCGCCAACACCAAGGGGCAGGAGGACTTCCTCGCCGAGCTCGGCA
TCATCAACCGCCTCCGCCACCGGAATCTCGTCAAGCTCATCGGCTGGTGCCACCAGGACGGCGTGCTGCTGCTGGTGTAC
GACTACATGCCACACGGCAGCCTGGACAGGCACCTGTTCGGCGGCAGGGAGGCGTCGGCGGCGGACTGGAAGCAGCGCTA
CAACGTGGTCGCCGGCGTGGCGTCGGCGCTGAACTACCTGCACCACGAGTTCGAGCAGACGGTGATCCACCGCGACATCA
AGCCGTCCAACATCATGCTGGACTCGTCGTTCCACGCGCGGCTGGGCGACTTCGGCCTGGCGCGCGCGCTCGAGTCCGAC
AAGACCTCGTACACGGACAAGCTGGGCGTGCCGGGCACGCTGGGGTACATCGCGCCCGAGTGCTTCCACACGGGGCGGGC
CACGCGCGAGTCGGACGTGTTCGGCTTCGGCGCCGTGGTCCTGGAGACCGTCTGCGGCCGCCGCGTCTCCTGCGACAACC
CGGCGGGGTGCAGCCAGCTGCTGGAGCTGGTGTGGAGGCTCCACGGCGCCGGGCGCCTCCTGGAGGCCGTGGACCCGCGC
CTCGCCGCCGGCGGGTACGGGTACGACGGCGAGGAGGCCGAGCGGCTGCTGCTGCTGGGCCTGGCGTGCAGCCACCCCAA
CCCGCGGCAGCGGCCCAAGGCGCAGGCCATCCTGCAGAACCTGCAGACGCGGTCCGTGCCGCCGCTCCCCGTGCCCATGT
CCAAGCCGGTGTTCATGTGGCCCGCGCCGCTCGCCGACGGGGAGGAGGACGCGACGCAGGCGTCGTCCATGTCGCGCAGC
GGGGTCACCAGCTCGGGCGTCACCTCCTCGTCCAACTACCCCTACACGTGGTCGTCGTCGGGGTACAATACCCAGACCTT
CCAGGTGAGCAGGGAGGTGCACGACGCGGCGGAGAGGGACGTGGCGACCGTTTGACTGAGAAGAAGACGGTGTCGAGCTC
AGCATGCTGCCGCCGGCAGGACGGCTCACTGACGGTGGTGCACGTGGAGCCCGGGGGGCGGGCCAGTCGGCAGTCGGGTT
GCGCGCTTTCATCAGGAGTCAAAAGGGGCTTGTGGCAACGCGGCTCACGATCGAATCGGACAAAATAAAGACGCCAACAA
AGCGCACGCGACAACGCGGGCAGGGTTTTCGTCGTCGGAGAGCCTTTTC
>Zm00001d051615_P002 peptide
MTTGPWRWCHGVRATASLALLLLLACVSGRSVTCARAQSTAFTGRETTSFSFPKFDQNRLQLSTNLTFTGNSTVSQGALQ
VTPDSGNNFSTYLANQAGRVFYSTPFVLWASNASSAAAAGRRVASFSTVFQFNLYRTNASVKGEGLAFVVASAIADPPPG
SHGGFLGLTNASTDGLGANGFAAVELDTVKQPYDPDGNHIGLDVNGVRSSSATCPLAALGIELAPADTGASDGSNFVWVD
YDGAARRLRAYISPNATKPSAAALDASLDLSAVVAARDAYFGFSASTGADDYQLNCVKMWNMTVEVLHDDDDRLPKKLSG
WKLGLAVGAPCAAALAALALLAGLYLMKKRRKVGDDPSSVSHSAIDLRSIPGVPKEFDYEALRKGTNGFDDKMKLGQGGY
GVVYRATVPGDNGRSMEVAVKQFSGANTKGQEDFLAELGIINRLRHRNLVKLIGWCHQDGVLLLVYDYMPHGSLDRHLFG
GREASAADWKQRYNVVAGVASALNYLHHEFEQTVIHRDIKPSNIMLDSSFHARLGDFGLARALESDKTSYTDKLGVPGTL
GYIAPECFHTGRATRESDVFGFGAVVLETVCGRRVSCDNPAGCSQLLELVWRLHGAGRLLEAVDPRLAAGGYGYDGEEAE
RLLLLGLACSHPNPRQRPKAQAILQNLQTRSVPPLPVPMSKPVFMWPAPLADGEEDATQASSMSRSGVTSSGVTSSSNYP
YTWSSSGYNTQTFQVSREVHDAAERDVATV
>Zm00001d051615_T003 cDNA
Chr4: 164598663..164602165
AGCCAGAGAGCGTCACCTTGGACCCTCAGGGTCGCGAGCCATACACGGGCGTCGCTGACGACACCACGCTGCCAACCCTA
GCCGCGCCTGCCTTCCTCCCCCGCTTGCCGTCGAGTATCGATGTTTGGAGGTGGGGGCGCCTTCGCCGGTGAGGTACCTG
GTGGGGGCAGCGATCATGATGAGCGGCGTGGTGCCTTCCTCCCTCCATGTCCTCCTCCGCGCCGAGCGCGCCCTCCGCGA
ACGCGACGTCGGCGAGGTCCACATCCCGCTGTCCGAGCTCCTCTCCGGCGCCCCCGACGGCCCCGTCCCCGCCAAGTTCG
TCGCCTACCAGGTCTGCAATGTTGCCGATGTCGAACCCTTCAGCTAATCCCACACAGCTTACAATTTTCTATGGTGGATC
AGTATGTGTGTATGACTCGGTGCCACCAGAAAAGGCTCAGGCAATCATGCTTATAGCTGCAGCTGCGGCAGCTGCGGCAG
CCACCAAAGGCAGTGCTGCCACTGCTTTTAATCCTCCAATGAGCACTTCTGTAGCAGCTGGACAAGCTTAGGTTGTTGCT
GACCCTAGCTCAATAAGCAAGCTTCAGGCTGATCTCCCCATTGCCAGGAGGCACTCTCTCCAGCGCTTCCTTGAGAAACG
TCGTGACAGGGTCGTGAGCAAAGCTCCGTACAGCCCCGCCAAGTCGTTCGATGGCATGGAGTCAGCGGGAATGGAGATGA
CTGTGGACGGCAAGCAGGGTGCAAGGCCCAGTATCTTGAAAGGTTGGGGAGCCGCGAAGTCTGAAGTCTGAACGGTGAAA
GCGTAGCCCCGCGGGAGCGTGGCGGTGTCTGGGAAGCGCCAGAAGCCGAGGTAGAGGGTGCAGAGGGAGGCAAGGTG
>Zm00001d051615_P003 peptide
MSSSAPSAPSANATSARSTSRCPSSSPAPPTAPSPPSSSPTRSAMLPMSNPSANPTQLTIFYGGSVCVYDSVPPEKAQAI
MLIAAAAAAAAATKGSAATAFNPPMSTSVAAGQA

>Zm00001d051615_T004 cDNA
Chr4: 164598701..164600922
AGGTCTGCAATGTTGCCGATGTCGAACCCTTCAGCTAATCCCACACAGCTTACAATTTTCTATGGTGGATCAGTATGTGT
GTATGACTCGGTGCCACCAGAAAAGGCTCAGGCAATCATGCTTATAGCTGCAGCTGCGGCAGCTGCGGCAGCCACCAAAG
GCAGTGCTGCCACTGCTTTTAATCCTCCAATGAGCACTTCTGTAGCAGCTGGACAAGCTTAGGTTGTTGCTGACCCTAGC
TCAATAAGCAAGCTTCAGGCTGATCTCCCCATTGCCAGGAGGCACTCTCTCCAGCGCTTCCTTGAGAAACGTCGTGACAG
GTTAGCATGATACATGAAAGCAACAGGGCTCTGATATTCTGAATCTTATTGTTAGGCCTCTTATTCTACAATCTACATGT
TTAAAACTTTTGTTATAAACTGCTGTGTGTGGTACCACACTAATTTTTTCCCCGACTATTGGCACCACAGTAATCAACAG
TATATTTTGTACCGTATTCTGTTTTTTGTCCTCCAGGCAGGGTAAAAATAAAATAAAATTTGCTGTTATAGCATTTAAAT
GATTGCACTTTCTGCTGACAAGTGCTCAATGACATCCCTAAAAAAATCTATAATTTCTTCACAGAAAGATTGGTAGAGAG
CCTGCTTCCATAATACAACATATAGCTGCAAATAGCCTTGGATCATGTAACTCCCTTGAATTTTTTTTTTAAAAATCTGC
AAATCGATATTAACACAAGACAGAGTTCTGGGACCAGCAGTAGATACAAAAACCAACATTACCCCCATTTAGCTGCTCCA
AATACCATGTTTTCCCCTGTTAGGAAAATAACATATCTGAGAATCGGGATGTTCCTGTGTATTATCCTGTTCTATTTGGA
AAAGCTGGCCACTTAAAGATCCAGTCTCAAGGTTGCAAACTAGCACCATTTTTTTTTCTGATGTAAGCTTTAGATGCCTG
TTTTTGCCTTATCTTAGAACTGAGTTTAGAATTGTTTCTTAGTATAACTTTTTTTTATCGTGGATCTACTGATTTCAAGT
GATTAAATAAATGATGGCACGAAACAGGGTCGTGAGCAAAGCTCCGTACAGCCCCGCCAAGTCGTTCGATGGCATGGAGT
CAGCGGGAATGGAGATGACTGTGGACGGCAAGCAGGGTGCAAGGCCCAGTATCTTGAAAGGTTGGGGAGCCGCGAAGTCT
GAAGTCTGAACGGTGAAAGCGTAGCCCCGCGGGAGCGTGGCGGTGTCTGGGAAGCGC
>Zm00001d051615_P004 peptide
MLPMSNPSANPTQLTIFYGGSVCVYDSVPPEKAQAIMLIAAAAAAAAATKGSAATAFNPPMSTSVAAGQA

ZmZML1 (TIFY18)

B73 RefGen_v3:
GRMZM2G065896 (zim2 - ZIM-transcription factor 2)
Chr5: 8462838..8467789

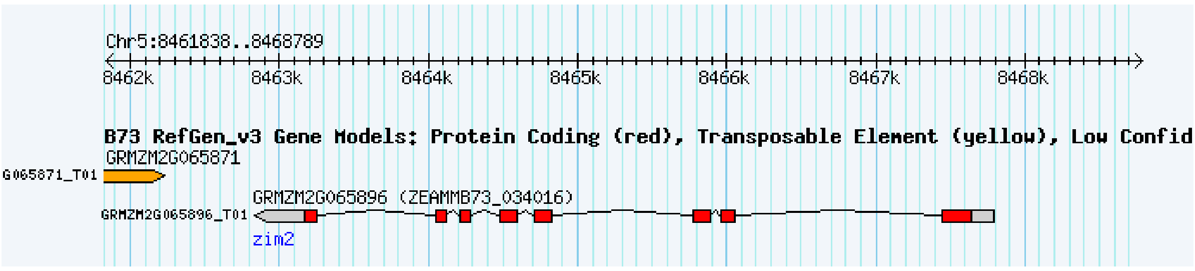

>GRMZM2G065896 Genomic DNA
CAGAGTGGGACCAGCAAGCCGCGCCAGTGTACATGTCTTCACCGTCACACGTGCGCTCCTCTCGTCCATCCTCCACACGT
GCGCTCAAACCGAGCTTGTCCACGGACCGCACCCTGTCTGCACGCATCTCCACCGTCCGATCCCCAGCCGATGGCGGCGG
AGCCCGCGGCGGCCGACCACGATCTCCGGCCACCCCTGGCGGATGGCGCCGCTGCTGCCGGCGTGGGCGCCGCCTCCTTG
GCGGCGGCGGCGGGCGCGGCAGAGGCGCTGATGAGCGCGACGTCGGAGCAGCTCACGCTGGTGTATCAGGGCGACGTCTA
CGTCTTCGACCCCGTCCCGCCCCAAAAGGTCGCGCCTCTTGCACCCCTGATAATTCCATTGGCCCCTCCCAAATTCTCTC
CTTGGTTCCTATATGTTGCGATGGTCGTTAATAGTCTTGGCCGGAGATTAGCGTGCGCGGGGCGCCGGCTATGAATTAGC
AAATATTGTTCTGCTTTTGCGGGGGCGCTCTGCTGTCGAGTTGCACATAATTAAATTCTGTCTTCATCGTTCTTGATTGA
ATTCTAAGGGGACTTGATTGCCAGCTATGGCTAAGTTAGTCGCAGTTTCTGCTTCGCTACTGTAAATTGACCATATTCCT
TTGTTCCCTTGCTGAATTGCACCAAAGAATACGGGTGCTATATGATATGACTGTGCTTTTGGTATCCTGTAAGGGGCTGA
ACTAATGTTATTATGAATTGCTCTGTCCATTTTTCGATCAGTATGGATATACCGTTCTACTGTTCGAGTGGAATCGAGAA
TGCTTGATCGCTAGGTAGACACTTTTTTGTGCGTGTTGAGATGTTTTATAGTTGTGTCGGGGAAAAGTCGCGAGTTTGAT

GAACAGCGGAGTAGATATCTTCGTTCTTCAGCTAGGATTTTAACATATTTGTGTTTTTGTTATAGTTGCCAAATAAAGTT
CCAATAGAAGCATGTATGTTCTTTTTTTCCGATGAGAAGTATAAATTCTTTGTTCCTTAAGTTTTATTAGTGTTTTAAAC
CCAAATATGAAAATTTGGAACTATGTTTCATCTTTGGTCCTGGACTAAGCTCACTCATATGATTTGCTTTTCTCATTGGC
AAGTTGAGGATTATTTTTGTTCTAGCTATTAGGGAAGATTATTTGGAAAGATGGAAGCAACTAGATTAGGGAACTTCTGT
GAGTAAGAAGTTGAGATGACTTGCCTGGATTGGTCAGAGTCATAGTCATAACTTTCAAAATGTTACTAGAGATCTAATCA
TGAAACAACATAAATATTTTGTCTTCACGGTTACAAGGTGATTTTGCCAGTCTTCCATTTGAACGTGTTTTTGAAATCTA
TTTGGTCTATTTTTCTTCAAAAAAAAATCTATTTGGTCTATCTCAATTCCTTATCTGCTTATTAGTGAGAATGCCTATAA
ATGCAATCATGAGCCCTAGTGATGTCGTTTGTAGTGAAAAGCTTCACTGGGATGTATGTCCTAAGATAAACCAAGACCAA
AGTAAGCATGATGCGACCATACTGAAGGTTTTCAGGGTGACCATATGCCTATGTCATTATTGATTTGTTTGAGTGCATAT
GGTGATGAGATTGTTTCACTGACACGTGTATCCCAGGGCCACATGCGAGCCAGCGTGTCAGTGAGATATGGGAGTCACAT
GTGCAGAAACTCAACCTAGGGGAGCTGTATATCCTAAGTACTTTCTCTTGATCAGGTTCAGGCTGTTCTTTTGGTACTTG
GAGGGTACGAGGTTCCGCCTGGTTTGGTAAACATGGCTGTTTCTTCAGCAAATGATGAGAAGGTATCCGCATACTTACTG
CAGTGAGGATAGGTATTTCCAGCTTCTCCTAAAGCATTGTCTCATGTGCAGAATACAACTGTGGCTGCTAGAAGGGTTGC
TTCCTTAATGAGATTCCGTGAGAAGAGAAAGGAAAGATGTTTCGATAAAAGGATTAGATACAGTGTGCGCAAGGAGGTTG
CCCAAAAGTAAGTACAGAATAATGTCACTTCTATGTTTAGCAATGATATAATTATTCTTCGCATGGGCATAATGAATTTT
TAAAGGCAATCTTGATTAACAGATTGTTTGCTGTCATTTGGAGGATTTGAGATATAAATAGTAAGAAGTCTGCATTTGTT
AGTCTCCACATTAGATAAAATTATCAGCTTATTGCAGACTTGATTATGTTTGACTTTGACATTGCTTTACCACATTAATA
GGATGTGCTGTAAGACCAGGATTTACTTAATTCTCTCCTTGTATCACTCTGTTTAAATAGGAATACACATGCCATATTCA
GATTATTATCACTGTTATATCATATCCAATCTTAGGTCACCTTTTTATCTCGAATAGTTTCTCCAAGTACTAATGCCAGA
AAAAAACTAAATAGGATAATAATCTGAATGTGCACCATGGATCTACCTATTTGGAGTACTTGTGCACTAAAGGTGTTATT
CAAGGTTACCATAAGCGAAGTCAATGTTTAAAAGATTTATTTCAGTCAGATATAAAAATGAACGAGGACTTTCTTCTATG
CCTTTTAGTCCACGATTTGTAAGAAGTATCATGTAAAGGCTTAGATGAACTCCGTGGAGAATGGAACAGATGATTTCTAT
TATTTCCATTTGTCAGAACTTTTCCAGAACTTATGTGCTTATTTGCATACCTACCTGGTTCAATCCAGTTACCTTTTTTT
ATATCAATCATGCTAGAAAAGGTGTTTTTTTCTTCTTTTCATCATACTGTTTACATTCTGTTGCAATAAACCCTGGCCAA
ACAATTGCTGCTCACCATCATTCACCACTGGGAATATTTCCCAGGTTTATTTACCGTAACCATTGCATAACCATATTTTG
CATTCAAATGATCGCTACTCAGTGATGTTTTTGTTGTTTTTTTTTTTTTGAATCTTGCATTGCACAATCTCAATAATACTT
TCAGGATGAAACGGCGTAAAGGCCAATTTGCTGGGAGATCAGATTTTGGCGATGGTGCCTGTTCTTCTGCAGCTTGTGGC
TCTCCAGCTAACGGCGAGGATGATCATTTTCGAGAAACCCAGTGAGTTAGTTGCCATGTGCCAACTATCACTTTGTGATT
GTGTGTATGTGTTACATCAGTTTCATATGCAGAAAGTTGTTGCCTTCATGTATGGACTAATGTTGGCCTGTTTTGCTAGT
TGCCAAAATTGTGGTATCAGCTCAAGGCTTACTCCAGCGATGCGTCGGGGCCCAGCTGGTCCGAGGTCCCTCTGCAATGC
TTGTGGCTTAATGTGGGCAAATAAGGTACAACTTTCTCTATGCCAATCATCGCACCTCTTGTCCAGTTGTCCTGTTTGGC
TGTTTGGTACCTTTTTCCTCATGTCAAATAAAAAGCATCATTCTTCTTAATTTGAAAAACTTCCCAATGAGATATTCTAA
TTGGTCAAAAAATGATCTGATTAAAGTGTGTCTATTAGCAACATTCCTACTAGTTAACCTTTGTTTTTTTCAGGGTACTC
TACGAAGTCCTCTAAATGCACCCAAAATGACTCAGCAGCTTCTTGCCAATCCATGTAACATGGTGCGCTACATTTGTAAT
AGAAACTTGTTTATTTGAACGCCTTTTCTTGTAGCGAAGATTATCTGAACAGTACACTTGTGCAGGTAGATACTGATGAC
AAAAACTCAAATGTTCTTCCTGTGGAGCATAATCAAGCCACACCCAAAACTGACTCGATGTAAGCATGCTCTTTACTGTA
CGTGAAATCATTGTCTGGAAGCTGTGTCATGTACATGGTCGGATGTGCAAATCTGAACTTCGTGTTCTCTTGAGTTAAGC
TCAAACTGGGAGGGACAAAGGCGCTGTTTGTTTACCCTCTAGATTATATAATCCAACTTAAATAAGTTGAGAGGCAAACA
TATAACTCAGATTATTAGGTGGATTATATAATCTAGATTCGTACAACAACAACAACAAAGCCTTTTTGTCCCAAGCACGT
TGGGGTAGGCTAGAGATGAAACCCCGTATGAAAACTTCAGAGCTCAACCCCAAAGAAAAGAAAAGGGAAACAATGGCAAA
AGAGAACCGAAAAACGACAAAACGGGGAAACACATAAAAAGAGATAAAACCCACAAGAAACAGCCAGATCTAAATGGGCA
CAAGAAAAGGTCAAACGATTAAGGAGGAAAAGCGAAACTATGAATCAAGGTTCTGGCACGTGAATTGCACACTTCCACCC
CTTCCTATCCACGGCGAGCTCTTTATCAATATTCCACTCTTTATCAATAATCTAGATTCGTAGATTATGATAATTCATAA
GCAGGTTATTAGGTGCTTCTATAATCCATAAGCTAGATTATAATAATCCTAGAAGGAAACAACAGGGCCAAAGTCTCATG

ACACAGTTTAGATGGATTTTTTTCATCTGCTTTATCAGTGCATATAGCTGCCTAGGTAACTTCTATTTTTTACCCAAGA
GGTCTCTCCAAAACTTTCTTTATAATGAAGTACACCAATCACTGAACCTTAATTTATTGGTCAGGATGCCAAAAGAGGAG
CAGAAGCTGGACATACGCTTGCCCACCGAAGAAGATACGAAGGCAGTTTCATGACAAATGCTCAAACATGTATATAGATC
GAGACAGGTTTCTGAGTTTAGGATGACATATAAATCAGTAAAACTAACAAAGGCAGCACAAAAAAAATTACATGCATTGT
GTTGCCACATTTATCTCCGTACCCGATTCATATTGTATTGCTAACTGATGCAGTTCTTACTCCTTACCCTGTGTCAACCT
GACCAGGTGACTTGGCAGGAACTCAAAATGTTTTCAAGAATACGACGCATTCTTGAGAAACGTTTTGTTTATTGTTGTAA
AAACCACTTCAATAGTAATTTGCATCAACTTTGATGCAATTTATTTTATAGAAAAACATGTGATATTTTGGT
>GRMZM2G065896_T01 cDNA
CAGAGTGGGACCAGCAAGCCGCGCCAGTGTACATGTCTTCACCGTCACACGTGCGCTCCTCTCGTCCATCCTCCACACGT
GCGCTCAAACCGAGCTTGTCCACGGACCGCACCCTGTCTGCACGCATCTCCACCGTCCGATCCCCAGCCGATGGCGGCGG
AGCCCGCGGCGGCCGACCACGATCTCCGGCCACCCCTGGCGGATGGCGCCGCTGCTGCCGGCGTGGGCGCCGCCTCCTTG
GCGGCGGCGGCGGGCGCGGCAGAGGCGCTGATGAGCGCGACGTCGGAGCAGCTCACGCTGGTGTATCAGGGCGACGTCTA
CGTCTTCGACCCCGTCCCGCCCCAAAAGGTTCAGGCTGTTCTTTTGGTACTTGGAGGGTACGAGGTTCCGCCTGGTTTGG
TAAACATGGCTGTTTCTTCAGCAAATGATGAGAAGAATACAACTGTGGCTGCTAGAAGGGTTGCTTCCTTAATGAGATTC
CGTGAGAAGAGAAAGGAAAGATGTTTCGATAAAAGGATTAGATACAGTGTGCGCAAGGAGGTTGCCCAAAAGATGAAACG
GCGTAAAGGCCAATTTGCTGGGAGATCAGATTTTGGCGATGGTGCCTGTTCTTCTGCAGCTTGTGGCTCTCCAGCTAACG
GCGAGGATGATCATTTTCGAGAAACCCATTGCCAAAATTGTGGTATCAGCTCAAGGCTTACTCCAGCGATGCGTCGGGGC
CCAGCTGGTCCGAGGTCCCTCTGCAATGCTTGTGGCTTAATGTGGGCAAATAAGGGTACTCTACGAAGTCCTCTAAATGC
ACCCAAAATGACTCAGCAGCTTCTTGCCAATCCATGTAACATGGTAGATACTGATGACAAAAACTCAAATGTTCTTCCTG
TGGAGCATAATCAAGCCACACCCAAAACTGACTCGATGATGCCAAAAGAGGAGCAGAAGCTGGACATACGCTTGCCCACC
GAAGAAGATACGAAGGCAGTTTCATGACAAATGCTCAAACATGTATATAGATCGAGACAGGTTTCTGAGTTTAGGATGAC
ATATAAATCAGTAAAACTAACAAAGGCAGCACAAAAAAAATTACATGCATTGTGTTGCCACATTTATCTCCGTACCCGAT
TCATATTGTATTGCTAACTGATGCAGTTCTTACTCCTTACCCTGTGTCAACCTGACCAGGTGACTTGGCAGGAACTCAAA
ATGTTTTCAAGAATACGACGCATTCTTGAGAAACGTTTTGTTTATTGTTGTAAAAACCACTTCAATAGTAATTTGCATCA
ACTTTGATGCAATTTATTTTATAGAAAAACATGTGATATTTTGGT
>GRMZM2G065896_P01 peptide
MAAEPAAADHDLRPPLADGAAAAGVGAASLAAAAGAAEALMSATSEQLTLVYQGDVYVFDPVPPQKVQAVLLVLGGYEVP
PGLVNMAVSSANDEKNTTVAARRVASLMRFREKRKERCFDKRIRYSVRKEVAQKMKRRKGQFAGRSDFGDGACSSAACGS
PANGEDDHFRETHCQNCGISSRLTPAMRRGPAGPRSLCNACGLMWANKGTLRSPLNAPKMTQQLLANPCNMVDTDDKNSN
VLPVEHNQATPKTDSMMPKEEQKLDIRLPTEEDTKAVS

AGPv4:

Zm00001d013331 (zim2 - ZIM-transcription factor 2)

Chr5: 8802187..8809065

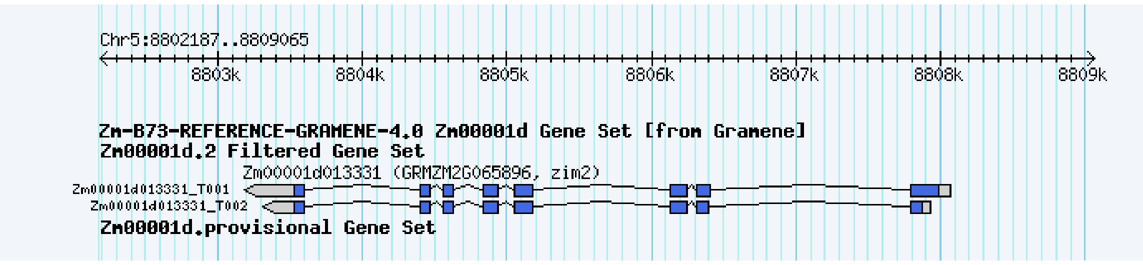

>Zm00001d013331 Genomic DNA
CACACGTGCGCTCAAACCGAGCTTGTCCACGGACCGCACCCTGTCTGCACGCATCTCCACCGTCCGATCCCCAGCCGATG
GCGGCGGAGCCCGCGGCGGCCGACCACGATCTCCGGCCACCCCTGGCGGATGGCGCCGCTGCTGCCGGCGTGGGCGCCGC
CTCCTTGGCGGCGGCGGCGGGCGCGGCAGAGGCGCTGATGAGCGCGACGTCGGAGCAGCTCACGCTGGTGTATCAGGGCG

ACGTCTACGTCTTCGACCCCGTCCCGCCCCAAAAGGTCGCGCCTCTTGCACCCCTGATAATTCCATTGGCCCCTCCCAAA
TTCTCTCCTTGGTTCCTATATGTTGCGATGGTCGTTAATAGTCTTGGCCGGAGATTAGCGTGCGCGGGGCGCCGGCTATG
AATTAGCAAATATTGTTCTGCTTTTGCGGGGGCGCTCTGCTGTCGAGTTGCACATAATTAAATTCTGTCTTCATCGTTCT
TGATTGAATTCTAAGGGGACTTGATTGCCAGCTATGGCTAAGTTAGTCGCAGTTTCTGCTTCGCTACTGTAAATTGACCA
TATTCCTTTGTTCCCTTGCTGAATTGCACCAAAGAATACGGGTGCTATATGATATGACTGTGCTTTTGGTATCCTGTAAG
GGGCTGAACTAATGTTATTATGAATTGCTCTGTCCATTTTTCGATCAGTATGGATATACCGTTCTACTGTTCGAGTGGAA
TCGAGAATGCTTGATCGCTAGGTAGACACTTTTTTGTGCGTGTTGAGATGTTTTATAGTTGTGTCGGGGAAAAGTCGCGA
GTTTGATGAACAGCGGAGTAGATATCTTCGTTCTTCAGCTAGGATTTTAACATATTTGTGTTTTTGTTATAGTTGCCAAA
TAAAGTTCCAATAGAAGCATGTATGTTCTTTTTTTCCGATGAGAAGTATAAATTCTTTGTTCCTTAAGTTTTATTAGTGT
TTTAAACCCAAATATGAAAATTTGGAACTATGTTTCATCTTTGGTCCTGGACTAAGCTCACTCATATGATTTGCTTTTCT
CATTGGCAAGTTGAGGATTATTTTTGTTCTAGCTATTAGGGAAGATTATTTGGAAAGATGGAAGCAACTAGATTAGGGAA
CTTCTGTGAGTAAGAAGTTGAGATGACTTGCCTGGATTGGTCAGAGTCATAGTCATAACTTTCAAAATGTTACTAGAGAT
CTAATCATGAAACAACATAAATATTTTGTCTTCACGGTTACAAGGTGATTTTGCCAGTCTTCCATTTGAACGTGTTTTTG
AAATCTATTTGGTCTATTTTTCTTCAAAAAAAAATCTATTTGGTCTATCTCAATTCCTTATCTGCTTATTAGTGAGAATG
CCTATAAATGCAATCATGAGCCCTAGTGATGTCGTTTGTAGTGAAAAGCTTCACTGGGATGTATGTCCTAAGATAAACCA
AGACCAAAGTAAGCATGATGCGACCATACTGAAGGTTTTCAGGGTGACCATATGCCTATGTCATTATTGATTTGTTTGAG
TGCATATGGTGATGAGATTGTTTCACTGACACGTGTATCCCAGGGCCACATGCGAGCCAGCGTGTCAGTGAGATATGGGA
GTCACATGTGCAGAAACTCAACCTAGGGGAGCTGTATATCCTAAGTACTTTCTCTTGATCAGGTTCAGGCTGTTCTTTTG
GTACTTGGAGGGTACGAGGTTCCGCCTGGTTTGGTAAACATGGCTGTTTCTTCAGCAAATGATGAGAAGGTATCCGCATA
CTTACTGCAGTGAGGATAGGTATTTCCAGCTTCTCCTAAAGCATTGTCTCATGTGCAGAATACAACTGTGGCTGCTAGAA
GGGTTGCTTCCTTAATGAGATTCCGTGAGAAGAGAAAGGAAAGATGTTTCGATAAAAGGATTAGATACAGTGTGCGCAAG
GAGGTTGCCCAAAAGTAAGTACAGAATAATGTCACTTCTATGTTTAGCAATGATATAATTATTCTTCGCATGGGCATAAT
GAATTTTTAAAGGCAATCTTGATTAACAGATTGTTTGCTGTCATTTGGAGGATTTGAGATATAAATAGTAAGAAGTCTGC
ATTTGTTAGTCTCCACATTAGATAAAATTATCAGCTTATTGCAGACTTGATTATGTTTGACTTTGACATTGCTTTACCAC
ATTAATAGGATGTGCTGTAAGACCAGGATTTACTTAATTCTCTCCTTGTATCACTCTGTTTAAATAGGAATACACATGCC
ATATTCAGATTATTATCACTGTTATATCATATCCAATCTTAGGTCACCTTTTTATCTCGAATAGTTTCTCCAAGTACTAA
TGCCAGAAAAAAACTAAATAGGATAATAATCTGAATGTGCACCATGGATCTACCTATTTGGAGTACTTGTGCACTAAAGG
TGTTATTCAAGGTTACCATAAGCGAAGTCAATGTTTAAAAGATTTATTTCAGTCAGATATAAAAATGAACGAGGACTTTC
TTCTATGCCTTTTAGTCCACGATTTGTAAGAAGTATCATGTAAAGGCTTAGATGAACTCCGTGGAGAATGGAACAGATGA
TTTCTATTATTTCCATTTGTCAGAACTTTTCCAGAACTTATGTGCTTATTTGCATACCTACCTGGTTCAATCCAGTTACC
TTTTTTTATATCAATCATGCTAGAAAAGGTGTTTTTTTCTTCTTTTCATCATACTGTTTACATTCTGTTGCAATAAACCC
TGGCCAAACAATTGCTGCTCACCATCATTCACCACTGGGAATATTTCCCAGGTTTATTTACCGTAACCATTGCATAACCA
TATTTTGCATTCAAATGATCGCTACTCAGTGATGTTTTTGTTGTTTTTTTTTTTTGAATCTTGCATTGCACAATCTCAAT
AATACTTTCAGGATGAAACGGCGTAAAGGCCAATTTGCTGGGAGATCAGATTTTGGCGATGGTGCCTGTTCTTCTGCAGC
TTGTGGCTCTCCAGCTAACGGCGAGGATGATCATTTTCGAGAAACCCAGTGAGTTAGTTGCCATGTGCCAACTATCACTT
TGTGATTGTGTGTATGTGTTACATCAGTTTCATATGCAGAAAGTTGTTGCCTTCATGTATGGACTAATGTTGGCCTGTTT
TGCTAGTTGCCAAAATTGTGGTATCAGCTCAAGGCTTACTCCAGCGATGCGTCGGGGCCCAGCTGGTCCGAGGTCCCTCT
GCAATGCTTGTGGCTTAATGTGGGCAAATAAGGTACAACTTTCTCTATGCCAATCATCGCACCTCTTGTCCAGTTGTCCT
GTTTGGCTGTTTGGTACCTTTTTCCTCATGTCAAATAAAAAGCATCATTCTTCTTAATTTGAAAAACTTCCCAATGAGAT
ATTCTAATTGGTCAAAAAATGATCTGATTAAAGTGTGTCTATTAGCAACATTCCTACTAGTTAACCTTTGTTTTTTTCAG
GGTACTCTACGAAGTCCTCTAAATGCACCCAAAATGACTCAGCAGCTTCTTGCCAATCCATGTAACATGGTGCGCTACAT
TTGTAATAGAAACTTGTTTATTTGAACGCCTTTTCTTGTAGCGAAGATTATCTGAACAGTACACTTGTGCAGGTAGATAC
TGATGACAAAAACTCAAATGTTCTTCCTGTGGAGCATAATCAAGCCACACCCAAAACTGACTCGATGTAAGCATGCTCTT
TACTGTACGTGAAATCATTGTCTGGAAGCTGTGTCATGTACATGGTCGGATGTGCAAATCTGAACTTCGTGTTCTCTTGA

GTTAAGCTCAAACTGGGAGGGACAAAGGCGCTGTTTGTTTACCCTCTAGATTATATAATCCAACTTAAATAAGTTGAGAG
GCAAACATATAACTCAGATTATTAGGTGGATTATATAATCTAGATTCGTACAACAACAACAACAAAGCCTTTTTGTCCCA
AGCACGTTGGGGTAGGCTAGAGATGAAACCCCGTATGAAAACTTCAGAGCTCAACCCCAAAGAAAAGAAAAGGGAAACAA
TGGCAAAAGAGAACCGAAAAACGACAAAACGGGGAAACACATAAAAAGAGATAAAACCCACAAGAAACAGCCAGATCTAA
ATGGGCACAAGAAAAGGTCAAACGATTAAGGAGGAAAAGCGAAACTATGAATCAAGGTTCTGGCACGTGAATTGCACACT
TCCACCCCTTCCTATCCACGGCGAGCTCTTTATCAATATTCCACTCTTTATCAATAATCTAGATTCGTAGATTATGATAA
TTCATAAGCAGGTTATTAGGTGCTTCTATAATCCATAAGCTAGATTATAATAATCCTAGAAGGAAACAACAGGGCCAAAG
TCTCATGACACAGTTTAGATGGATTTTTTTTCATCTGCTTTATCAGTGCATATAGCTGCCTAGGTAACTTCTATTTTTTA
CCCAAGAGGTCTCTCCAAAACTTTCTTTATAATGAAGTACACCAATCACTGAACCTTAATTTATTGGTCAGGATGCCAAA
AGAGGAGCAGAAGCTGGACATACGCTTGCCCACCGAAGAAGATACGAAGGCAGTTTCATGACAAATGCTCAAACATGTAT
ATAGATCGAGACAGGTTTCTGAGTTTAGGATGACATATAAATCAGTAAAACTAACAAAGGCAGCACAAAAAAAAATTACAT
GCATTGTGTTGCCACATTTATCTCCGTACCCGATTCATATTGTATTGCTAACTGATGCAGTTCTTACTCCTTACCCTGTG
TCAACCTGACCAGGTGACTTGGCAGGAACTCAAAATGTTTTCAAGAATACGACGCATTCTTGAGAAACGTTTTGTTTATT
GTTGTAAAAACCACTTCAATAGTAATTTGCATCAACTTTGATGCAATTTATTTTATAGAAAAACATGTGATATTTTGGT
>Zm00001d013331_T001 cDNA
Chr5: 8803187..8808065
CACACGTGCGCTCAAACCGAGCTTGTCCACGGACCGCACCCTGTCTGCACGCATCTCCACCGTCCGATCCCCAGCCGATG
GCGGCGGAGCCCGCGGCGGCCGACCACGATCTCCGGCCACCCCTGGCGGATGGCGCCGCTGCTGCCGGCGTGGGCGCCGC
CTCCTTGGCGGCGGCGGCGGGCGCGGCAGAGGCGCTGATGAGCGCGACGTCGGAGCAGCTCACGCTGGTGTATCAGGGCG
ACGTCTACGTCTTCGACCCCGTCCCGCCCCAAAAGGTTCAGGCTGTTCTTTTGGTACTTGGAGGGTACGAGGTTCCGCCT
GGTTTGGTAAACATGGCTGTTTCTTCAGCAAATGATGAGAAGAATACAACTGTGGCTGCTAGAAGGGTTGCTTCCTTAAT
GAGATTCCGTGAGAAGAGAAAGGAAAGATGTTTCGATAAAAGGATTAGATACAGTGTGCGCAAGGAGGTTGCCCAAAAGA
TGAAACGGCGTAAAGGCCAATTTGCTGGGAGATCAGATTTTGGCGATGGTGCCTGTTCTTCTGCAGCTTGTGGCTCTCCA
GCTAACGGCGAGGATGATCATTTTCGAGAAACCCATTGCCAAAATTGTGGTATCAGCTCAAGGCTTACTCCAGCGATGCG
TCGGGGCCCAGCTGGTCCGAGGTCCCTCTGCAATGCTTGTGGCTTAATGTGGGCAAATAAGGGTACTCTACGAAGTCCTC
TAAATGCACCCAAAATGACTCAGCAGCTTCTTGCCAATCCATGTAACATGGTAGATACTGATGACAAAAACTCAAATGTT
CTTCCTGTGGAGCATAATCAAGCCACACCCAAAACTGACTCGATGATGCCAAAAGAGGAGCAGAAGCTGGACATACGCTT
GCCCACCGAAGAAGATACGAAGGCAGTTTCATGACAAATGCTCAAACATGTATATAGATCGAGACAGGTTTCTGAGTTTA
GGATGACATATAAATCAGTAAAACTAACAAAGGCAGCACAAAAAAAAATTACATGCATTGTGTTGCCACATTTATCTCCGT
ACCCGATTCATATTGTATTGCTAACTGATGCAGTTCTTACTCCTTACCCTGTGTCAACCTGACCAGGTGACTTGGCAGGA
ACTCAAAATGTTTTCAAGAATACGACGCATTCTTGAGAAACGTTTTGTTTATTGTTGTAAAAACCACTTCAATAGTAATT
TGCATCAACTTTGATGCAATTTATTTTATAGAAAAACATGTGATATTTTGGT
>Zm00001d013331_P001 peptide
MAAEPAAADHDLRPPLADGAAAAGVGAASLAAAAGAAEALMSATSEQLTLVYQGDVYVFDPVPPQKVQAVLLVLGGYEVP
PGLVNMAVSSANDEKNTTVAARRVASLMRFREKRKERCFDKRIRYSVRKEVAQKMKRRKGQFAGRSDFGDGACSSAACGS
PANGEDDHFRETHCQNCGISSRLTPAMRRGPAGPRSLCNACGLMWANKGTLRSPLNAPKMTQQLLANPCNMVDTDDKNSN
VLPVEHNQATPKTDSMMPKEEQKLDIRLPTEEDTKAVS
>Zm00001d013331_T002 cDNA
Chr5: 8803305..8807925
GCTGCCGGCGTGGGCGCCGCCTCCTTGGCGGCGGCGGCGGGCGCGGCAGAGGCGCTGATGAGCGCGACGTCGGAGCAGCT
CACGCTGGTGTATCAGGGCGACGTCTACGTCTTCGACCCCGTCCCGCCCCAAAAGGCTGTTCTTTTGGTACTTGGAGGGT
ACGAGGTTCCGCCTGGTTTGGTAAACATGGCTGTTTCTTCAGCAAATGATGAGAAGAATACAACTGTGGCTGCTAGAAGG
GTTGCTTCCTTAATGAGATTCCGTGAGAAGAGAAAGGAAAGATGTTTCGATAAAAGGATTAGATACAGTGTGCGCAAGGA
GGTTGCCCAAAAGATGAAACGGCGTAAAGGCCAATTTGCTGGGAGATCAGATTTTGGCGATGGTGCCTGTTCTTCTGCAG

CTTGTGGCTCTCCAGCTAACGGCGAGGATGATCATTTTCGAGAAACCCATTGCCAAAATTGTGGTATCAGCTCAAGGCTT
ACTCCAGCGATGCGTCGGGGCCCAGCTGGTCCGAGGTCCCTCTGCAATGCTTGTGGCTTAATGTGGGCAAATAAGGGTAC
TCTACGAAGTCCTCTAAATGCACCCAAAATGACTCAGCAGCTTCTTGCCAATCCATGTAACATGGTAGATACTGATGACA
AAAACTCAAATGTTCTTCCTGTGGAGCATAATCAAGCCACACCCAAAACTGACTCGATGATGCCAAAAGAGGAGCAGAAG
CTGGACATACGCTTGCCCACCGAAGAAGATACGAAGGCAGTTTCATGACAAATGCTCAAACATGTATATAGATCGAGACA
GGTTTCTGAGTTTAGGATGACATATAAATCAGTAAAACTAACAAAGGCAGCACAAAAAAAATTACATGCATTGTGTTGCC
ACATTTATCTCCGTACCCGATTCATATTGTATTGCTAACTGATGCAGTTCTTACTCCTTACCCTGTGTCAACCTGACCAG
GTGACTTGGCAGGAACTCAAAATGTTTT
>Zm00001d013331_P002 peptide
MSATSEQLTLVYQGDVYVFDPVPPQKAVLLVLGGYEVPPGLVNMAVSSANDEKNTTVAARRVASLMRFREKRKERCFDKR
IRYSVRKEVAQKMKRRKGQFAGRSDFGDGACSSAACGSPANGEDDHFRETHCQNCGISSRLTPAMRRGPAGPRSLCNACG
LMWANKGTLRSPLNAPKMTQQLLANPCNMVDTDDKNSNVLPVEHNQATPKTDSMMPKEEQKLDIRLPTEEDTKAVS

ZmZML2

B73 RefGen_v3:
GRMZM2G058479 (zim36 - ZIM-transcription factor 36)
Chr5: 55956384..55962158

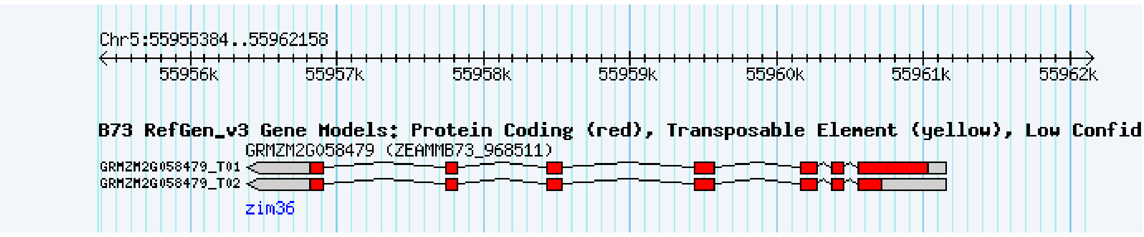

>GRMZM2G058479 Genomic DNA
ACTACGTCGTCTTCGTCTCTCCAACCCGGGGACATTTTCCACCCTCCAAATCGAAACCAGATTGAAATCCTAGGGGCTAG
GGTTACCCTCCATCCCATCGTCCGATCCGATCCGATCCCGCCGCTCATCCATGTCCCACCACGACGGAAGCAAGCCATAC
CAGCCGCGCCGGGGGCCCGAGCGGCACCCGCAGCCAGCGGATGGGATCGCCGCCCCGCCTCCCGCCGCCGTTGCCCCGTC
GGTGGAGCACCTCGTGGCGGCCGCCGCCGAGGCGGAGGCATTGAACCGCTTCGCCGCGGAACAACAGCAGCAGCTGCAGG
GGCACGAGCAGGAGGTTGGGGAGGAAGAGGAGGAGGAGGACGAGCAGGAAGACGAGATGGAGGAGGAGGACGAGGATGAG
CACGAAGGTCAGCACGGCGGCATCGGTGGGGAGCACGTTCCCATGGACGCGGATGCTGCTGCCGCGGCGGCGGCGGCCGC
AGTCTCGCAGATGGACCCGCACTCGGCGTTGGTGGCTGGGACCGTGCCACCCATGGCGACCAACCAGCTCACCCTCTCGT
TCCAGGGCGAGGTCTATGTGTTCGACTCCGTCTCCCCTGATAAGGTCCCCCCTCCTCTTCTGCTCTCTGTAGATGTTAAC
TTGTTTTTCTCTGCGTGTAGTCAATTCAATTCGCTATACCGATGAGAGCTGTTGGTTTTGTCTGCAGGTCCAAGCCGTGC
TTTTGCTGCTTGGAGGGAGGGAGCTGAGCAGCCTGGGCGGAGCGTCGTCCTCTGCACCTTATAGTAAGGTTAGGATAAGT
GATGTTGAATTTATGTATGATGCCTATGTTCATATTAACTGCAAACTGGAATGGATGGTTCACATGCGTGTATTTGCTCT
TCTCCTTTCAGAGGTTGAATTATCCACATCGGGTGGCATCTCTGATGAGATTTAGGGAAAAGCGGAAGGAGCGGAACTTT
GATAAGAAGATCCGATACTCTGTCCGGAAGGAAGTTGCACTTAGGTTGGTGATAGCTGTTTAACTTTGGTTGGATACACT
GTTCGCACTATCTGAAGTGGCATTAAGTTTGTTTTGTCGGAACTCAATAAACTTAATTTTTGTGATTGGAAACCAAGGAT
TTGCCCTATTGTATGGCATCATGTTCCTTGTTCAAATGTAACTAGTTCCAAGTGGGAAGATGCACACCATAACTCCATGG
CATTTTCCAAGTTCTTATGATTAGAATAGATTGATTATTCGTGAGCCCTAATTTTTATGTGTTCGTTTTTAGCAGTGACT
ATTGTTTGGTAGTCATTAGGAATCCTTCGAGTCATGACTCATGAGTGACTGTATGTACTACTTTCACCAGTTGTCATGTA
TGTTGCAAAAAAAAAAGTTGCTCACCTGATTAACTAGTTTCATTATTTCTTACTGTTGCATGGTTTTTGGTTGCGTAGC
AGCTTTGTCCATTTATGCTGACGTGGTGTTTCTTGATTTAAATGTAGGATTATGATGTTATGTTGGATGTTGCTATATAG

TCTGCTTCATCATAGAAGGTTGTGCATATGCATTCTTGGGATTTACACAATTTGCTTGTATCTGTAGGATGCAGCGTAAT
CGAGGTCAATTTACATCTTCAAAACCAAAGCCTGATGAAATAGCAGCATCAGAAATGGCATCCGCAGATGGCTCCCCGAA
TTGGGCATTAGTTGAAGGCCGACCTCCATCTGCTGCCGAGTAAGTCACAGAAGTGTTTTCAGTTTGCTTGTGGTTTACAG
GTGTTTGACTTATGCATGAATGTTTTGAGATGATTAAAAGACACTATTGCATATTTTCTTGTTATTTCATTGTATATGCC
TTGAATTTCAGATCCTTGTTTGTTCTCTGGAACAATTATCAGAAAGAAGTACAGATCTGAGCTATAATACAAAAAAATAT
ATAAACCATGCTGTGGTTACTATACATCTTCCTGGTTGACACCATTGTATGTTAGTACTTCAATACTTCCCACCTAGCCG
CACACAGAATGTTCATTTGTTACTCACATGATATGCCTCTACAGTATTAGCAATCAAATGTTGTGGTGTTGGTAACTGTT
GAAATCTTTTTTATTCTGCTTCCTAGATTACCATGCACATCAGTCATGAACTCAAGATTCTCCAGCTTTGCTGGTTCTGG
TGGTTAGATAGGTCTGTACTGCACGGACTATGTGGTTTAAGATCAGCTTCCTGCTGGTTTAGGCATGATTTGGATAATCA
AACTTCAAACTGTTGTGCCACATTACAAGCAAGTTTGCTAAAAGAAAAAAGAAACACTTGTTGGTGTACCTAGCATGCAT
TGACACATGCTTCTTTCAAAAGGTTCTTGTTTTGTGGATATTATGTTTTCATATTGCATGTGATTGGAGTTCCTTTTAAA
TATTTAAAAGGGGGGCAGACTCAGTGGCGAAGGTTCTTAGATGAGTGGGGTTTGGAGAAGGGCTAAATCGAGGCAAGTCT
TCCCCCGCAAATGCGGAGAGGCTGCTTAGAACCCAGTCACCAGTGAGACAGTTGTCATCACTGCACTAGGCCTGCCCTTC
TTTTAAATATTTAAAAAGTAAAAAAATCATTTCTGAATTGTTCTGATGTCCAATTCTTTCAGATGTCATCACTGTGGTAC
TAATGCAACAGCTACACCAATGATGCGTCGCGGACCTGATGGACCAAGAACATTATGCAATGCATGTGGCCTCATGTGGG
CAAATAAGGTATTTGTGATTGCTTAGCAGTACTTACAATGCAAGATTTTAGCATCATTGAAACTTCAAACATATTTATTT
ATAGCTTAGACTACGTAATGGACCAAGAGGCGAGTGTGAGTAAGAAATCCACGTCCCAGAACAATGACCCAGGCTAATGG
TCCCCCCTTTTATCCTGGCCCAACGTGCTCGGGAAGACAGTTGCTGTTTTTGTAAACTATAAAATGGACCCAACATAACA
TGTAACAACCATCTTAGTATTAAGAATTTTTAAAAATATGTAAAAAAATCTTGCTGGGTACTTTGCCTTTGGATGCTTAC
ATGACAATCACAATGCTACACTGAAGTTTTGCTGACCCTGTAATTTACAAAACATTGTGTCGATCTTTTTTTATATTGCG
CCACTGTAATCTAACCTGTGCTCACGCACTCTTCTGGTGTATTTTGGTTGTTTCTGTTTTAGAGATAAAATGCAGATCTT
TCTCATCTAGTGAATTTGTTCTTTCTTTTCTGGTTTGTATTATGGTTGCATTAGAGAAAATACCTCATAATGTGGAACCT
TATTGGTTTAACCACAAGTAAATCTAGGGTTGATCGTCCATTCACATCTTTATAGGGTCTCCTGAGGGACGTAACAAAAT
CTCCTGTACCTCTCCAAGCTACGCAATCAGCTCCGCATCTAGATGGTGGTGTAAGTTTTTCTTTCTTTGTTCGTTGCGTA
TGTTTTCTACCTAATCTTGGTGGGGATCCTGTTTGGTTGAGCCTTATCCGATAAGTTGTAAGTGAAAATCCTTGATTCAG
TTTTGTTGATTCCTTATCGAATTATTTGCAACGCACCTTTCATGATCTCTGTCCTAGTTATGGTATCTGATTCTAACAAA
TCGATCTGAGAGGTCGTTTTAACATTGTACTCAAGTGCAGTAGGCGCATGCTTTTTCCATCTATCCCTTTTTCCTCTTAG
TGCTTGTTTGGGAGCAAAGGTAATAGAGGGGGTCGAGGGGGCCAAAATCCGTTGCTATTCAGGCTTGTTTTGGGAGCAAA
GGTAATAGTGGTGGTTGAGGGGGCCAAAATCTCTTGCTATTCAATTTTGAATAGCAAGGAATTTTGGCTCCTCAACCCCC
TCCATTACCCTTGGTCCTAAACGAGCCCTTAGGCTATATCCATCAGATCCCTATTCCATCTCCTATCTCATCTCCTATTT
TAAACCTCTCTCTGCAAACAGTGTCACCTAGCGTCCCCTATTTTATGCACTCTACTGGAGATAGTCTTATGCTTCAAACT
AAGAAAAAATGTTTATTGCTCCACTAAACAAGAGTTACTGTTAATCTTCTCTTTACATTACTACTTCATAGTTCATAATGT
TTCAGGGACAGTATAAGATGGATTACCTTATCATCCTTTCCCAATTTCTCTTCCACAGCTCCCATTATTTTCTCCTGTCT
ACCTTGTTTGTTTTGATAAATTGTATGCCTTCGTATTCTGAATAATTAACTGGTTGGAACTTAATTGCGGTCTACTTTTT
TTCCAAGCAGAATGGAAGCGCCATGTCTGCGCCTGGCTCTGAGCTAGAGAATGCTGCAGCAGCCATGACCAATGGCCACG
AGTCATCGAGTAGTGGTGTTTAATCCAGCGATGGGGTAAAAGCCACGAGGAGCATCATCCCGGAGTGATCTCTGCAGTAG
GCGGTCAGCTGCTGTACTATAATTGAGTTGGAGGCTGCTTAGATATGGAATCCTTCCATTTACCTTGGTGCCCTGTAGTT
AAGAGCTATGACAGTGCTTTATCCCGTGTCATGTTTGCAGCCGCTGCGTCTATAACGAAGCTATTTCTTGTCTTGAGGCG
TGACTGTTAAGAACCGTTGAAGTCCACTCAGTTTTAACCGTGCAGCAACGTCTTCGATCAATATCTCAGAGTACTTAGGG
AGCGCTTGCCACACGGAGCAGCTAAACCACCGAAACGTTTCCATTTTCAACTAGAATCGGACTGTGCCATTTTGAAATGC
GTCACCGAAATGTAATCGATGTATATCGAATTGGCAGAAGGTTGGTTCCGTATAT

>GRMZM2G058479_T01 cDNA

Chr5: 55956384..55961158

ACTACGTCGTCTTCGTCTCTCCAACCCGGGGACATTTTCCACCCTCCAAATCGAAACCAGATTGAAATCCTAGGGGCTAG

GGTTACCCTCCATCCCATCGTCCGATCCGATCCGATCCCGCCGCTCATCCATGTCCCACCACGACGGAAGCAAGCCATAC
CAGCCGCGCCGGGGGCCCGAGCGGCACCCGCAGCCAGCGGATGGGATCGCCGCCCCGCCTCCCGCCGCCGTTGCCCCGTC
GGTGGAGCACCTCGTGGCGGCCGCCGCCGAGGCGGAGGCATTGAACCGCTTCGCCGCGGAACAACAGCAGCAGCTGCAGG
GGCACGAGCAGGAGGTTGGGGAGGAAGAGGAGGAGGAGGACGAGCAGGAAGACGAGATGGAGGAGGAGGACGAGGATGAG
CACGAAGGTCAGCACGGCGGCATCGGTGGGGAGCACGTTCCCATGGACGCGGATGCTGCTGCCGCGGCGGCGGCGGCCGC
AGTCTCGCAGATGGACCCGCACTCGGCGTTGGTGGCTGGGACCGTGCCACCCATGGCGACCAACCAGCTCACCCTCTCGT
TCCAGGGCGAGGTCTATGTGTTCGACTCCGTCTCCCCTGATAAGGTCCAAGCCGTGCTTTTGCTGCTTGGAGGGAGGGAG
CTGAGCAGCCTGGGCGGAGCGTCGTCCTCTGCACCTTATAGTAAGAGGTTGAATTATCCACATCGGGTGGCATCTCTGAT
GAGATTTAGGGAAAAGCGGAAGGAGCGGAACTTTGATAAGAAGATCCGATACTCTGTCCGGAAGGAAGTTGCACTTAGGA
TGCAGCGTAATCGAGGTCAATTTACATCTTCAAAACCAAAGCCTGATGAAATAGCAGCATCAGAAATGGCATCCGCAGAT
GGCTCCCCGAATTGGGCATTAGTTGAAGGCCGACCTCCATCTGCTGCCGAATGTCATCACTGTGGTACTAATGCAACAGC
TACACCAATGATGCGTCGCGGACCTGATGGACCAAGAACATTATGCAATGCATGTGGCCTCATGTGGGCAAATAAGGGTC
TCCTGAGGGACGTAACAAAATCTCCTGTACCTCTCCAAGCTACGCAATCAGCTCCGCATCTAGATGGTGGTAATGGAAGC
GCCATGTCTGCGCCTGGCTCTGAGCTAGAGAATGCTGCAGCAGCCATGACCAATGGCCACGAGTCATCGAGTAGTGGTGT
TTAATCCAGCGATGGGGTAAAAGCCACGAGGAGCATCATCCCGGAGTGATCTCTGCAGTAGGCGGTCAGCTGCTGTACTA
TAATTGAGTTGGAGGCTGCTTAGATATGGAATCCTTCCATTTACCTTGGTGCCCTGTAGTTAAGAGCTATGACAGTGCTT
TATCCCGTGTCATGTTTGCAGCCGCTGCGTCTATAACGAAGCTATTTCTTGTCTTGAGGCGTGACTGTTAAGAACCGTTG
AAGTCCACTCAGTTTTAACCGTGCAGCAACGTCTTCGATCAATATCTCAGAGTACTTAGGGAGCGCTTGCCACACGGAGC
AGCTAAACCACCGAAACGTTTCCATTTTCAACTAGAATCGGACTGTGCCATTTTGAAATGCGTCACCGAAATGTAATCGA
TGTATATCGAATTGGCAGAAGGTTGGTTCCGTATAT
>GRMZM2G058479_P01 peptide
MSHHDGSKPYQPRRGPERHPQPADGIAAPPPAAVAPSVEHLVAAAAEAEALNRFAAEQQQQLQGHEQEVGEEEEEEDEQE
DEMEEEDEDEHEGQHGGIGGEHVPMDADAAAAAAAAAVSQMDPHSALVAGTVPPMATNQLTLSFQGEVYVFDSVSPDKVQ
AVLLLLGGRELSSLGGASSSAPYSKRLNYPHRVASLMRFREKRKERNFDKKIRYSVRKEVALRMQRNRGQFTSSKPKPDE
IAASEMASADGSPNWALVEGRPPSAAECHHCGTNATATPMMRRGPDGPRTLCNACGLMWANKGLLRDVTKSPVPLQATQS
APHLDGGNGSAMSAPGSELENAAAAMTNGHESSSSGV
>GRMZM2G058479_T02 cDNA
Chr5: 55956384..55961158
ACTACGTCGTCTTCGTCTCTCCAACCCGGGGACATTTTCCACCCTCCAAATCGAAACCAGATTGAAATCCTAGGGGCTAG
GGTTACCCTCCATCCCATCGTCCGATCCGATCCGATCCCGCCGCTCATCCATGTCCCACCACGACGGAAGCAAGCCATAC
CAGCCGCGCCGGGGGCCCGAGCGGCACCCGCAGCCAGCGGATGGGATCGCCGCCCCGCCTCCCGCCGCCGTTGCCCCGTC
GGTGGAGAGCACGTTCCCATGGACGCGGATGCTGCTGCCGCGGCGGCGGCGGCCGCAGTCTCGCAGATGGACCCGCACTC
GGCGTTGGTGGCTGGGACCGTGCCACCCATGGCGACCAACCAGCTCACCCTCTCGTTCCAGGGCGAGGTCTATGTGTTCG
ACTCCGTCTCCCCTGATAAGGTCCAAGCCGTGCTTTTGCTGCTTGGAGGGAGGGAGCTGAGCAGCCTGGGCGGAGCGTCG
TCCTCTGCACCTTATAGTAAGAGGTTGAATTATCCACATCGGGTGGCATCTCTGATGAGATTTAGGGAAAAGCGGAAGGA
GCGGAACTTTGATAAGAAGATCCGATACTCTGTCCGGAAGGAAGTTGCACTTAGGATGCAGCGTAATCGAGGTCAATTTA
CATCTTCAAAACCAAAGCCTGATGAAATAGCAGCATCAGAAATGGCATCCGCAGATGGCTCCCCGAATTGGGCATTAGTT
GAAGGCCGACCTCCATCTGCTGCCGAATGTCATCACTGTGGTACTAATGCAACAGCTACACCAATGATGCGTCGCGGACC
TGATGGACCAAGAACATTATGCAATGCATGTGGCCTCATGTGGGCAAATAAGGGTCTCCTGAGGGACGTAACAAAATCTC
CTGTACCTCTCCAAGCTACGCAATCAGCTCCGCATCTAGATGGTGGTAATGGAAGCGCCATGTCTGCGCCTGGCTCTGAG
CTAGAGAATGCTGCAGCAGCCATGACCAATGGCCACGAGTCATCGAGTAGTGGTGTTTAATCCAGCGATGGGGTAAAAGC
CACGAGGAGCATCATCCCGGAGTGATCTCTGCAGTAGGCGGTCAGCTGCTGTACTATAATTGAGTTGGAGGCTGCTTAGA
TATGGAATCCTTCCATTTACCTTGGTGCCCTGTAGTTAAGAGCTATGACAGTGCTTTATCCCGTGTCATGTTTGCAGCCG
CTGCGTCTATAACGAAGCTATTTCTTGTCTTGAGGCGTGACTGTTAAGAACCGTTGAAGTCCACTCAGTTTTAACCGTGC

AGCAACGTCTTCGATCAATATCTCAGAGTACTTAGGGAGCGCTTGCCACACGGAGCAGCTAAACCACCGAAACGTTTCCA
TTTTCAACTAGAATCGGACTGTGCCATTTTGAAATGCGTCACCGAAATGTAATCGATGTATATCGAATTGGCAGAAGGTT
GGTTCCGTATAT
>GRMZM2G058479_P02 peptide
MDADAAAAAAAAAVSQMDPHSALVAGTVPPMATNQLTLSFQGEVYVFDSVSPDKVQAVLLLLGGRELSSLGGASSSAPYS
KRLNYPHRVASLMRFREKRKERNFDKKIRYSVRKEVALRMQRNRGQFTSSKPKPDEIAASEMASADGSPNWALVEGRPPS
AAECHHCGTNATATPMMRRGPDGPRTLCNACGLMWANKGLLRDVTKSPVPLQATQSAPHLDGGNGSAMSAPGSELENAAA
AMTNGHESSSSGV
AGPv4:
Zm00001d014656 (zim36 - ZIM-transcription factor 36)
Chr5: 57722133..57729010

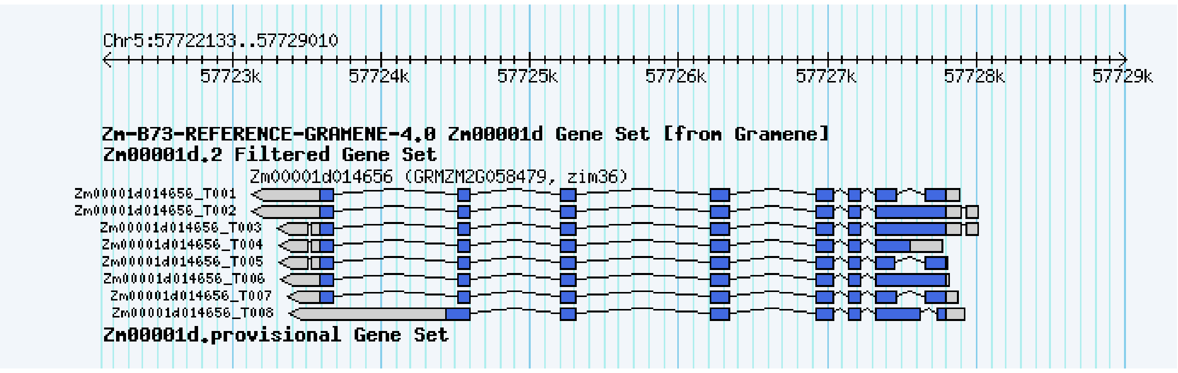

>Zm00001d014656 Genomic DNA
CTAGGAACACCGTACAAAAAAAAACAATGCATCCGACGGCTGTGGTGTGTCCACAAAACCTACCCGAACCCATGGACGCC
TACTACGTCGTCTTCGTCTCTCCAACCCGGGGACATTTTCCACCCTCCAAATCGAAACCAGATTGAAATCCTAGGGGCTA
GGGTTACCCTCCATCCCATCGTCCGATCCGATCCGATCCCGCCGCTCATCCATGTCCCACCACGACGGAAGCAAGCCATA
CCAGCCGCGCCGGGGGCCCGAGCGGCACCCGCAGCCAGCGGATGGGATCGCCGCCCCGCCTCCCGCCGCCGTTGCCCCGT
CGGTGGAGCACCTCGTGGCGGCCGCCGCCGAGGCGGAGGCATTGAACCGCTTCGCCGCGGAACAACAGCAGCAGCTGCAG
GGGCACGAGCAGGAGGTTGGGGAGGAAGAGGAGGAGGAGGACGAGCAGGAAGACGAGATGGAGGAGGAGGACGAGGATGA
GCACGAAGGTCAGCACGGCGGCATCGGTGGGGAGCACGTTCCCATGGACGCGGATGCTGCTGCCGCGGCGGCGGCGGCCG
CAGTCTCGCAGATGGACCCGCACTCGGCGTTGGTGGCTGGGACCGTGCCACCCATGGCGACCAACCAGCTCACCCTCTCG
TTCCAGGGCGAGGTCTATGTGTTCGACTCCGTCTCCCCTGATAAGGTCCCCCCTCCTCTTCTGCTCTCTGTAGATGTTAA
CTTGTTTTTCTCTGCGTGTAGTCAATTCAATTCGCTATACCGATGAGAGCTGTTGGTTTTGTCTGCAGGTCCAAGCCGTG
CTTTTGCTGCTTGGAGGGAGGGAGCTGAGCAGCCTGGGCGGAGCGTCGTCCTCTGCACCTTATAGTAAGGTTAGGATAAG
TGATGTTGAATTTATGTATGATGCCTATGTTCATATTAACTGCAAACTGGAATGGATGGTTCACATGCGTGTATTTGCTC
TTCTCCTTTCAGAGGTTGAATTATCCACATCGGGTGGCATCTCTGATGAGATTTAGGGAAAAGCGGAAGGAGCGGAACTT
TGATAAGAAGATCCGATACTCTGTCCGGAAGGAAGTTGCACTTAGGTTGGTGATAGCTGTTTAACTTTGGTTGGATACAC
TGTTCGCACTATCTGAAGTGGCATTAAGTTTGTTTTGTCGGAACTCAATAAACTTAATTTTTGTGATTGGAAACCAAGGA
TTTGCCCTATTGTATGGCATCATGTTCCTTGTTCAAATGTAACTAGTTCCAAGTGGGAAGATGCACACCATAACTCCATG
GCATTTTCCAAGTTCTTATGATTAGAATAGATTGATTATTCGTGAGCCCTAATTTTTATGTGTTCGTTTTTAGCAGTGAC
TATTGTTTGGTAGTCATTAGGAATCCTTCGAGTCATGACTCATGAGTGACTGTATGTACTACTTTCACCAGTTGTCATGT
ATGTTGCAAAAAAAAAAGTTGCTCACCTGATTAACTAGTTTTCATTATTTCTTACTGTTGCATGGTTTTTGGTTGCGTAG
CAGCTTTGTCCATTTATGCTGACGTGGTGTTTCTTGATTTAAATGTAGGATTATGATGTTATGTTGGATGTTGCTATATA
GTCTGCTTCATCATAGAAGGTTGTGCATATGCATTCTTGGGATTTACACAATTTGCTTGTATCTGTAGGATGCAGCGTAA
TCGAGGTCAATTTACATCTTCAAAACCAAAGCCTGATGAAATAGCAGCATCAGAAATGGCATCCGCAGATGGCTCCCCGA
ATTGGGCATTAGTTGAAGGCCGACCTCCATCTGCTGCCGAGTAAGTCACAGAAGTGTTTTCAGTTTGCTTGTGGTTTACA

GGTGTTTGACTTATGCATGAATGTTTTGAGATGATTAAAAGACACTATTGCATATTTTCTTGTTATTTCATTGTATATGC
CTTGAATTTCAGATCCTTGTTTGTTCTCTGGAACAATTATCAGAAAGAAGTACAGATCTGAGCTATAATACAAAAAAATA
TATAAACCATGCTGTGGTTACTATACATCTTCCTGGTTGACACCATTGTATGTTAGTACTTCAATACTTCCCACCTAGCC
GCACACAGAATGTTCATTTGTTACTCACATGATATGCCTCTACAGTATTAGCAATCAAATGTTGTGGTGTTGGTAACTGT
TGAAATCTTTTTTATTCTGCTTCCTAGATTACCATGCACATCAGTCATGAACTCAAGATTCTCCAGCTTTGCTGGTTCTG
GTGGTTAGATAGGTCTGTACTGCACGGACTATGTGGTTTAAGATCAGCTTCCTGCTGGTTTAGGCATGATTTGGATAATC
AAACTTCAAACTGTTGTGCCACATTACAAGCAAGTTTGCTAAAAGAAAAAAGAAACACTTGTTGGTGTACCTAGCATGCA
TTGACACATGCTTCTTTCAAAAGGTTCTTGTTTTGTGGATATTATGTTTTCATATTGCATGTGATTGGAGTTCCTTTTAA
ATATTTAAAAGGGGGGCAGACTCAGTGGCGAAGGTTCTTAGATGAGTGGGGTTTGGAGAAGGGCTAAATCGAGGCAAGTC
TTCCCCCGCAAATGCGGAGAGGCTGCTTAGAACCCAGTCACCAGTGAGACAGTTGTCATCACTGCACTAGGCCTGCCCTT
CTTTTAAATATTTAAAAAGTAAAAAAATCATTTCTGAATTGTTCTGATGTCCAATTCTTTCAGATGTCATCACTGTGGTA
CTAATGCAACAGCTACACCAATGATGCGTCGCGGACCTGATGGACCAAGAACATTATGCAATGCATGTGGCCTCATGTGG
GCAAATAAGGTATTTGTGATTGCTTAGCAGTACTTACAATGCAAGATTTTAGCATCATTGAAACTTCAAACATATTTATT
TATAGCTTAGACTACGTAATGGACCAAGAGGCGAGTGTGAGTAAGAAATCCACGTCCCAGAACAATGACCCAGGCTAATG
GTCCCCCCTTTTATCCTGGCCCAACGTGCTCGGGAAGACAGTTGCTGTTTTTGTAAACTATAAAATGGACCCAACATAAC
ATGTAACAACCATCTTAGTATTAAGAATTTTTAAAAATATGTAAAAAAATCTTGCTGGGTACTTTGCCTTTGGATGCTTA
CATGACAATCACAATGCTACACTGAAGTTTTGCTGACCCTGTAATTTACAAAACATTGTGTCGATCTTTTTTTATATTGC
GCCACTGTAATCTAACCTGTGCTCACGCACTCTTCTGGTGTATTTTGGTTGTTTCTGTTTTAGAGATAAAATGCAGATCT
TTCTCATCTAGTGAATTTGTTCTTTCTTTTCTGGTTTGTATTATGGTTGCATTAGAGAAAATACCTCATAATGTGGAACC
TTATTGGTTTAACCACAAGTAAATCTAGGGTTGATCGTCCATTCACATCTTTATAGGGTCTCCTGAGGGACGTAACAAAA
TCTCCTGTACCTCTCCAAGCTACGCAATCAGCTCCGCATCTAGATGGTGGTGTAAGTTTTTCTTTCTTTGTTCGTTGCGT
ATGTTTTCTACCTAATCTTGGTGGGGATCCTGTTTGGTTGAGCCTTATCCGATAAGTTGTAAGTGAAAATCCTTGATTCA
GTTTTGTTGATTCCTTATCGAATTATTTGCAACGCACCTTTCATGATCTCTGTCCTAGTTATGGTATCTGATTCTAACAA
ATCGATCTGAGAGGTCGTTTTAACATTGTACTCAAGTGCAGTAGGCGCATGCTTTTTCCATCTATCCCTTTTTCCTCTTA
GTGCTTGTTTGGGAGCAAAGGTAATAGAGGGGGTCGAGGGGGCCAAAATCCGTTGCTATTCAGGCTTGTTTTGGGAGCAA
AGGTAATAGTGGTGGTTGAGGGGGCCAAAATCTCTTGCTATTCAATTTTGAATAGCAAGGAATTTTGGCTCCTCAACCCC
CTCCATTACCCTTGGTCCTAAACGAGCCCTTAGGCTATATCCATCAGATCCCTATTCCATCTCCTATCTCATCTCCTATT
TTAAACCTCTCTCTGCAAACAGTGTCACCTAGCGTCCCCTATTTTATGCACTCTACTGGAGATAGTCTTATGCTTCAAAC
TAAGAAAAATGTTTATTGCTCCACTAAACAAGAGTTACTGTTAATCTTCTCTTTACATTACTACTTCATAGTTCATAATG
TTTCAGGGACAGTATAAGATGGATTACCTTATCATCCTTTCCCAATTTCTCTTCCACAGCTCCCATTATTTTCTCCTGTC
TACCTTGTTTGTTTTGATAAATTGTATGCCTTCGTATTCTGAATAATTAACTGGTTGGAACTTAATTGCGGTCTACTTTT
TTTCCAAGCAGAATGGAAGCGCCATGTCTGCGCCTGGCTCTGAGCTAGAGAATGCTGCAGCAGCCATGACCAATGGCCAC
GAGTCATCGAGTAGTGGTGTTTAATCCAGCGATGGGGTAAAAGCCACGAGGAGCATCATCCCGGAGTGATCTCTGCAGTA
GGCGGTCAGCTGCTGTACTATAATTGAGTTGGAGGCTGCTTAGATATGGAATCCTTCCATTTACCTTGGTGCCCTGTAGT
TAAGAGCTATGACAGTGCTTTATCCCGTGTCATGTTTGCAGCCCGCTGCGTCTATAACGAAGCTATTTCTTGTCTTGAGGC
GTGACTGTTAAGAACCGTTGAAGTCCACTCAGTTTTAACCGTGCAGCAACGTCTTCGATCAATATCTCAGAGTACTTAGG
GAGCGCTTGCCACACGGAGCAGCTAAACCACCGAAACGTTTCCATTTTCAACTAGAATCGGACTGTGCCATTTTGAAATG
CGTCACCGAAATGTAATCGATGTATATCGAATTGGCAGAAGGTTGGTTCCGTATATGAACCATCTCAGTTTCTTTGCC
>Zm00001d014656_T001 cDNA
Chr5: 57723133..57727886
CTCCAAATCGAAACCAGATTGAAATCCTAGGGGCTAGGGTTACCCTCCATCCCATCGTCCGATCCGATCCGATCCCGCCG
CTCATCCATGTCCCACCACGACGGAAGCAAGCCATACCAGCCGCGCCGGGGGCCCGAGCGGCACCCGCAGCCAGCGGATG
GGATCGCCGCCCCGCCTCCCGCCGCCGTTGCCCCGTCGGTGGAGCACCTCGTGGCGGCCGCCGCCGAGGCGGCGGCGGCG
GCCGCAGTCTCGCAGATGGACCCGCACTCGGCGTTGGTGGCTGGGACCGTGCCACCCATGGCGACCAACCAGCTCACCCT

CTCGTTCCAGGGCGAGGTCTATGTGTTCGACTCCGTCTCCCCTGATAAGGTCCAAGCCGTGCTTTTGCTGCTTGGAGGGA
GGGAGCTGAGCAGCCTGGGCGGAGCGTCGTCCTCTGCACCTTATAGTAAGAGGTTGAATTATCCACATCGGGTGGCATCT
CTGATGAGATTTAGGGAAAAGCGGAAGGAGCGGAACTTTGATAAGAAGATCCGATACTCTGTCCGGAAGGAAGTTGCACT
TAGGATGCAGCGTAATCGAGGTCAATTTACATCTTCAAAACCAAAGCCTGATGAAATAGCAGCATCAGAAATGGCATCCG
CAGATGGCTCCCCGAATTGGGCATTAGTTGAAGGCCGACCTCCATCTGCTGCCGAATGTCATCACTGTGGTACTAATGCA
ACAGCTACACCAATGATGCGTCGCGGACCTGATGGACCAAGAACATTATGCAATGCATGTGGCCTCATGTGGGCAAATAA
GGGTCTCCTGAGGGACGTAACAAAATCTCCTGTACCTCTCCAAGCTACGCAATCAGCTCCGCATCTAGATGGTGGTAATG
GAAGCGCCATGTCTGCGCCTGGCTCTGAGCTAGAGAATGCTGCAGCAGCCATGACCAATGGCCACGAGTCATCGAGTAGT
GGTGTTTAATCCAGCGATGGGGTAAAAGCCACGAGGAGCATCATCCCGGAGTGATCTCTGCAGTAGGCGGTCAGCTGCTG
TACTATAATTGAGTTGGAGGCTGCTTAGATATGGAATCCTTCCATTTACCTTGGTGCCCTGTAGTTAAGAGCTATGACAG
TGCTTTATCCCGTGTCATGTTTGCAGCCGCTGCGTCTATAACGAAGCTATTTCTTGTCTTGAGGCGTGACTGTTAAGAAC
CGTTGAAGTCCACTCAGTTTTAACCGTGCAGCAACGTCTTCGATCAATATCTCAGAGTACTTAGGGAGCGCTTGCCACAC
GGAGCAGCTAAACCACCGAAACGTTTCCATTTTCAACTAGAATCGGACTGTGCCATTTTGAAATGCGTCACCGAAATGTA
ATCGATGTATATCGAATTGGCAGAAGGTTGGTTCCGTATATGAACCATCTCAGTTTCTTTGCC

>Zm00001d014656_P001 peptide

MSHHDGSKPYQPRRGPERHPQPADGIAAPPPAAVAPSVEHLVAAAAEAAAAAAVSQMDPHSALVAGTVPPMATNQLTLSF
QGEVYVFDSVSPDKVQAVLLLLGGRELSSLGGASSSAPYSKRLNYPHRVASLMRFREKRKERNFDKKIRYSVRKEVALRM
QRNRGQFTSSKPKPDEIAASEMASADGSPNWALVEGRPPSAAECHHCGTNATATPMMRRGPDGPRTLCNACGLMWANKGL
LRDVTKSPVPLQATQSAPHLDGGNGSAMSAPGSELENAAAAMTNGHESSSSGV

>Zm00001d014656_T002 cDNA

Chr5: 57723133..57728010

CTAGGAACACCGTACAAAAAAAAACAATGCATCCGACGGCTGTGGTGTGTCCACAAAACCTACCCGAACCCATGGACGCC
TAGGACATTTTCCACCCTCCAAATCGAAACCAGATTGAAATCCTAGGGGCTAGGGTTACCCTCCATCCCATCGTCCGATC
CGATCCGATCCCGCCGCTCATCCATGTCCCACCACGACGGAAGCAAGCCATACCAGCCGCGCCGGGGGCCCGAGCGGCAC
CCGCAGCCAGCGGATGGGATCGCCGCCCCGCCTCCCGCCGCCGTTGCCCCGTCGGTGGAGCACCTCGTGGCGGCCGCCGC
CGAGGCGGAGGCATTGAACCGCTTCGCCGCGGAACAACAGCAGCAGCTGCAGGGGCACGAGCAGGAGGTTGGGGAGGAAG
AGGAGGAGGAGGACGAGCAGGAAGACGAGATGGAGGAGGAGGACGAGGATGAGCACGAAGGTCAGCACGGCGGCATCGGT
GGGGAGCACGTTCCCATGGACGCGGATGCTGCTGCCGCGGCGGCGGCGGCCGCAGTCTCGCAGATGGACCCGCACTCGGC
GTTGGTGGCTGGGACCGTGCCACCCATGGCGACCAACCAGCTCACCCTCTCGTTCCAGGGCGAGGTCTATGTGTTCGACT
CCGTCTCCCCTGATAAGGTCCAAGCCGTGCTTTTGCTGCTTGGAGGGAGGGAGCTGAGCAGCCTGGGCGGAGCGTCGTCC
TCTGCACCTTATAGTAAGAGGTTGAATTATCCACATCGGGTGGCATCTCTGATGAGATTTAGGGAAAAGCGGAAGGAGCG
GAACTTTGATAAGAAGATCCGATACTCTGTCCGGAAGGAAGTTGCACTTAGGATGCAGCGTAATCGAGGTCAATTTACAT
CTTCAAAACCAAAGCCTGATGAAATAGCAGCATCAGAAATGGCATCCGCAGATGGCTCCCCGAATTGGGCATTAGTTGAA
GGCCGACCTCCATCTGCTGCCGAATGTCATCACTGTGGTACTAATGCAACAGCTACACCAATGATGCGTCGCGGACCTGA
TGGACCAAGAACATTATGCAATGCATGTGGCCTCATGTGGGCAAATAAGGGTCTCCTGAGGGACGTAACAAAATCTCCTG
TACCTCTCCAAGCTACGCAATCAGCTCCGCATCTAGATGGTGGTAATGGAAGCGCCATGTCTGCGCCTGGCTCTGAGCTA
GAGAATGCTGCAGCAGCCATGACCAATGGCCACGAGTCATCGAGTAGTGGTGTTTAATCCAGCGATGGGGTAAAAGCCAC
GAGGAGCATCATCCCGGAGTGATCTCTGCAGTAGGCGGTCAGCTGCTGTACTATAATTGAGTTGGAGGCTGCTTAGATAT
GGAATCCTTCCATTTACCTTGGTGCCCTGTAGTTAAGAGCTATGACAGTGCTTTATCCCGTGTCATGTTTGCAGCCGCTG
CGTCTATAACGAAGCTATTTCTTGTCTTGAGGCGTGACTGTTAAGAACCGTTGAAGTCCACTCAGTTTTAACCGTGCAGC
AACGTCTTCGATCAATATCTCAGAGTACTTAGGGAGCGCTTGCCACACGGAGCAGCTAAACCACCGAAACGTTTCCATTT
TCAACTAGAATCGGACTGTGCCATTTTGAAATGCGTCACCGAAATGTAATCGATGTATATCGAATTGGCAGAAGGTTGGT
TCCGTATATGAACCATCTCAGTTTCTTTGCC

>Zm00001d014656_P002 peptide

MSHHDGSKPYQPRRGPERHPQPADGIAAPPPAAVAPSVEHLVAAAAEAEALNRFAAEQQQQLQGHEQEVGEEEEEEDEQE
DEMEEEDEDEHEGQHGGIGGEHVPMDADAAAAAAAAAVSQMDPHSALVAGTVPPMATNQLTLSFQGEVYVFDSVSPDKVQ
AVLLLLGGRELSSLGGASSSAPYSKRLNYPHRVASLMRFREKRKERNFDKKIRYSVRKEVALRMQRNRGQFTSSKPKPDE
IAASEMASADGSPNWALVEGRPPSAAECHHCGTNATATPMMRRGPDGPRTLCNACGLMWANKGLLRDVTKSPVPLQATQS
APHLDGGNGSAMSAPGSELENAAAAMTNGHESSSSGV
>Zm00001d014656_T003 cDNA
Chr5: 57723307..57728010
CTAGGAACACCGTACAAAAAAAAACAATGCATCCGACGGCTGTGGTGTGTCCACAAAACCTACCCGAACCCATGGACGCC
TAGGACATTTTCCACCCTCCAAATCGAAACCAGATTGAAATCCTAGGGGCTAGGGTTACCCTCCATCCCATCGTCCGATC
CGATCCGATCCCGCCGCTCATCCATGTCCCACCACGACGGAAGCAAGCCATACCAGCCGCGCCGGGGGCCCGAGCGGCAC
CCGCAGCCAGCGGATGGGATCGCCGCCCCGCCTCCCGCCGCCGTTGCCCCGTCGGTGGAGCACCTCGTGGCGGCCGCCGC
CGAGGCGGAGGCATTGAACCGCTTCGCCGCGGAACAACAGCAGCAGCTGCAGGGGCACGAGCAGGAGGTTGGGGAGGAAG
AGGAGGAGGAGGACGAGCAGGAAGACGAGATGGAGGAGGAGGACGAGGATGAGCACGAAGGTCAGCACGGCGGCATCGGT
GGGGAGCACGTTCCCATGGACGCGGATGCTGCTGCCGCGGCGGCGGCGGCCGCAGTCTCGCAGATGGACCCGCACTCGGC
GTTGGTGGCTGGGACCGTGCCACCCATGGCGACCAACCAGCTCACCCTCTCGTTCCAGGGCGAGGTCTATGTGTTCGACT
CCGTCTCCCCTGATAAGGTCCAAGCCGTGCTTTTGCTGCTTGGAGGGAGGGAGCTGAGCAGCCTGGGCGGAGCGTCGTCC
TCTGCACCTTATAGTAAGAGGTTGAATTATCCACATCGGGTGGCATCTCTGATGAGATTTAGGGAAAAGCGGAAGGAGCG
GAACTTTGATAAGAAGATCCGATACTCTGTCCGGAAGGAAGTTGCACTTAGGATGCAGCGTAATCGAGGTCAATTTACAT
CTTCAAAACCAAAGCCTGATGAAATAGCAGCATCAGAAATGGCATCCGCAGATGGCTCCCCGAATTGGGCATTAGTTGAA
GGCCGACCTCCATCTGCTGCCGAATGTCATCACTGTGGTACTAATGCAACAGCTACACCAATGATGCGTCGCGGACCTGA
TGGACCAAGAACATTATGCAATGCATGTGGCCTCATGTGGGCAAATAAGGGTCTCCTGAGGGACGTAACAAAATCTCCTG
TACCTCTCCAAGCTACGCAATCAGCTCCGCATCTAGATGGTGGTCAGAATGGAAGCGCCATGTCTGCGCCTGGCTCTGAG
CTAGAGAATGCTGCAGCAGCCATGACCAATGGCCACGAGTCATCGAGTAGTGGTGTTTAATCCAGCGATGGGGTAAAAGC
CACGAGGAGCATCATCCCGGAGTGATCTCTGCAGTAGGCGTGAGTTGGAGGCTGCTTAGATATGGAATCCTTCCATTTAC
CTTGGTGCCCTGTAGTTAAGAGCTATGACAGTGCTTTATCCCGTGTCATGTTTGCAGCCGCTGCGTCTATAACGAAGCTA
TTTCTTGTCTTGAGGCGTGACTGTTAAGAACCGTTGAAGTCCACTCAGTTTTAACCGTGCAGCAACGTCTTCGATCAATA
>Zm00001d014656_P003 peptide
MSHHDGSKPYQPRRGPERHPQPADGIAAPPPAAVAPSVEHLVAAAAEAEALNRFAAEQQQQLQGHEQEVGEEEEEEDEQE
DEMEEEDEDEHEGQHGGIGGEHVPMDADAAAAAAAAAVSQMDPHSALVAGTVPPMATNQLTLSFQGEVYVFDSVSPDKVQ
AVLLLLGGRELSSLGGASSSAPYSKRLNYPHRVASLMRFREKRKERNFDKKIRYSVRKEVALRMQRNRGQFTSSKPKPDE
IAASEMASADGSPNWALVEGRPPSAAECHHCGTNATATPMMRRGPDGPRTLCNACGLMWANKGLLRDVTKSPVPLQATQS
APHLDGGQNGSAMSAPGSELENAAAAMTNGHESSSSGV
>Zm00001d014656_T004 cDNA
Chr5: 57723312..57727772
TACCAGCCGCGCCGGGGGCCCGAGCGGCACCCGCAGCCAGCGGATGGGATCGCCGCCCCGCCTCCCGCCGCCGTTGCCCC
GTCGGTGGAGCACCTCGTGGCGGCCGCCGCCGAGGCGGAGGCATTGAACCGCTTCGCCGCGGAACAACAGCAGCAGCTGC
AGGGGCACGAGCAGGAGGTTGGGGAGGAAGAGGAGGAGGAGGACGAGCAGGAAGACGAGATGGAGGAGGAGGACGAGGAT
GAGCACGAAGGTCAGCACGGCGGCATCGGTGGGGAGCACGTTCCCATGGACGCGGATGCTGCTGCCGCGGCGGCGGCGGC
CGCAGTCTCGCAGATGGACCCGCACTCGGCGTTGGTGGCTGGGACCGTGCCACCCATGGCGACCAACCAGCTCACCCTCT
CGTTCCAGGGCGAGGTCTATGTGTTCGACTCCGTCTCCCCTGATAAGGTCCAAGCCGTGCTTTTGCTGCTTGGAGGGAGG
GAGCTGAGCAGCCTGGGCGGAGCGTCGTCCTCTGCACCTTATAGTAAGAGGTTGAATTATCCACATCGGGTGGCATCTCT
GATGAGATTTAGGGAAAAGCGGAAGGAGCGGAACTTTGATAAGAAGATCCGATACTCTGTCCGGAAGGAAGTTGCACTTA
GGATGCAGCGTAATCGAGGTCAATTTACATCTTCAAAACCAAAGCCTGATGAAATAGCAGCATCAGAAATGGCATCCGCA
GATGGCTCCCCGAATTGGGCATTAGTTGAAGGCCGACCTCCATCTGCTGCCGAATGTCATCACTGTGGTACTAATGCAAC

AGCTACACCAATGATGCGTCGCGGACCTGATGGACCAAGAACATTATGCAATGCATGTGGCCTCATGTGGGCAAATAAGG
GTCTCCTGAGGGACGTAACAAAATCTCCTGTACCTCTCCAAGCTACGCAATCAGCTCCGCATCTAGATGGTGGTAATGGA
AGCGCCATGTCTGCGCCTGGCTCTGAGCTAGAGAATGCTGCAGCAGCCATGACCAATGGCCACGAGTCATCGAGTAGTGG
TGTTTAATCCAGCGATGGGGTAAAAGCCACGAGGAGCATCATCCCGGAGTGATCTCTGCAGTAGGCGTGAGTTGGAGGCT
GCTTAGATATGGAATCCTTCCATTTACCTTGGTGCCCTGTAGTTAAGAGCTATGACAGTGCTTTATCCCGTGTCATGTTT
GCAGCCGCTGCGTCTATAACGAAGCTATTTCTTGTCTTGAGGCGTGACTGTTAAGAACCGTTGAAGTCCACTCAGTTTTA
ACCGTGCAGCAACGTCTTCGAT
>Zm00001d014656_P004 peptide
MEEEDEDEHEGQHGGIGGEHVPMDADAAAAAAAAAVSQMDPHSALVAGTVPPMATNQLTLSFQGEVYVFDSVSPDKVQAV
LLLLGGRELSSLGGASSSAPYSKRLNYPHRVASLMRFREKRKERNFDKKIRYSVRKEVALRMQRNRGQFTSSKPKPDEIA
ASEMASADGSPNWALVEGRPPSAAECHHCGTNATATPMMRRGPDGPRTLCNACGLMWANKGLLRDVTKSPVPLQATQSAP
HLDGGNGSAMSAPGSELENAAAAMTNGHESSSSGV
>Zm00001d014656_T005 cDNA
Chr5: 57723312..57727802
TCCATGTCCCACCACGACGGAAGCAAGCCATACCAGCCGCGCCGGGGGCCCGAGCGGCACCCGCAGCCAGCGGATGGGAT
CGCCGCCCCGCCTCCCGCCGCCGTTGCCCCGTCGGTGGAGCACCTCGTGGCGGCCGCCGTCTCGCAGATGGACCCGCACT
CGGCGTTGGTGGCTGGGACCGTGCCACCCATGGCGACCAACCAGCTCACCCTCTCGTTCCAGGGCGAGGTCTATGTGTTC
GACTCCGTCTCCCCTGATAAGGTCCAAGCCGTGCTTTTGCTGCTTGGAGGGAGGGAGCTGAGCAGCCTGGGCGGAGCGTC
GTCCTCTGCACCTTATAGTAAGAGGTTGAATTATCCACATCGGGTGGCATCTCTGATGAGATTTAGGGAAAAGCGGAAGG
AGCGGAACTTTGATAAGAAGATCCGATACTCTGTCCGGAAGGAAGTTGCACTTAGGATGCAGCGTAATCGAGGTCAATTT
ACATCTTCAAAACCAAAGCCTGATGAAATAGCAGCATCAGAAATGGCATCCGCAGATGGCTCCCCGAATTGGGCATTAGT
TGAAGGCCGACCTCCATCTGCTGCCGAATGTCATCACTGTGGTACTAATGCAACAGCTACACCAATGATGCGTCGCGGAC
CTGATGGACCAAGAACATTATGCAATGCATGTGGCCTCATGTGGGCAAATAAGGGTCTCCTGAGGGACGTAACAAAATCT
CCTGTACCTCTCCAAGCTACGCAATCAGCTCCGCATCTAGATGGTGGTCAGAATGGAAGCGCCATGTCTGCGCCTGGCTC
TGAGCTAGAGAATGCTGCAGCAGCCATGACCAATGGCCACGAGTCATCGAGTAGTGGTGTTTAATCCAGCGATGGGGTAA
AAGCCACGAGGAGCATCATCCCGGAGTGATCTCTGCAGTAGGCGTGAGTTGGAGGCTGCTTAGATATGGAATCCTTCCAT
TTACCTTGGTGCCCTGTAGTTAAGAGCTATGACAGTGCTTTATCCCGTGTCATGTTTGCAGCCGCTGCGTCTATAACGAA
GCTATTTCTTGTCTTGAGGCGTGACTGTTAAGAACCGTTGAAGTCCACTCAGTTTTAACCGTGCAGCAACGTCTTCGAT
>Zm00001d014656_P005 peptide
MSHHDGSKPYQPRRGPERHPQPADGIAAPPPAAVAPSVEHLVAAAVSQMDPHSALVAGTVPPMATNQLTLSFQGEVYVFD
SVSPDKVQAVLLLLGGRELSSLGGASSSAPYSKRLNYPHRVASLMRFREKRKERNFDKKIRYSVRKEVALRMQRNRGQFT
SSKPKPDEIAASEMASADGSPNWALVEGRPPSAAECHHCGTNATATPMMRRGPDGPRTLCNACGLMWANKGLLRDVTKSP
VPLQATQSAPHLDGGQNGSAMSAPGSELENAAAAMTNGHESSSSGV
>Zm00001d014656_T006 cDNA
Chr5: 57723330..57727813
CCCGCCGCTCATCCATGTCCCACCACGACGGAAGCAAGCCATACCAGCCGCGCCGGGGGCCCGAGCGGCACCCGCAGCCA
GCGGATGGGATCGCCGCCCCGCCTCCCGCCGCCGTTGCCCCGTCGGTGGAGCACCTCGTGGCGGCCGCCGCCGAGGCGGA
GGCATTGAACCGCTTCGCCGCGGAACAACAGCAGCAGCTGCAGGGGCACGAGCAGGAGGTTGGGGAGGAAGAGGAGGAGG
AGGACGAGCAGGAAGACGAGATGGAGGAGGAGGACGAGGATGAGCACGAAGGTCAGCACGGCGGCATCGGTGGGGAGCAC
GTTCCCATGGACGCGGATGCTGCTGCCGCGGCGGCGGCGGCCGCAGTCTCGCAGATGGACCCGCACTCGGCGTTGGTGGC
TGGGACCGTGCCACCCATGGCGACCAACCAGCTCACCCTCTCGTTCCAGGGCGAGGTCTATGTGTTCGACTCCGTCTCCC
CTGATAAGGTCCAAGCCGTGCTTTTGCTGCTTGGAGGGAGGGAGCTGAGCAGCCTGGGCGGAGCGTCGTCCTCTGCACCT
TATAGTAAGAGGTTGAATTATCCACATCGGGTGGCATCTCTGATGAGATTTAGGGAAAAGCGGAAGGAGCGGAACTTTGA
TAAGAAGATCCGATACTCTGTCCGGAAGGAAGTTGCACTTAGGATGCAGCGTAATCGAGGTCAATTTACATCTTCAAAAC

CAAAGCCTGATGAAATAGCAGCATCAGAAATGGCATCCGCAGATGGCTCCCCGAATTGGGCATTAGTTGAAGGCCGACCT
CCATCTGCTGCCGAATGTCATCACTGTGGTACTAATGCAACAGCTACACCAATGATGCGTCGCGGACCTGATGGACCAAG
AACATTATGCAATGCATGTGGCCTCATGTGGGCAAATAAGGGTCTCCTGAGGGACGTAACAAAATCTCCTGTACCTCTCC
AAGCTACGCAATCAGCTCCGCATCTAGATGGTGGTAATGGAAGCGCCATGTCTGCGCCTGGCTCTGAGCTAGAGAATGCT
GCAGCAGCCATGACCAATGGCCACGAGTCATCGAGTAGTGGTGTTTAATCCAGCGATGGGGTAAAAGCCACGAGGAGCAT
CATCCCGGAGTGATCTCTGCAGTAGGCGGTCAGCTGCTGTACTATAATTGAGTTGGAGGCTGCTTAGATATGGAATCCTT
CCATTTACCTTGGTGCCCTGTAGTTAAGAGCTATGACAGTGCTTTATCCCGTGTCATGTTTGCAGCCGCTGCGTCTATAA
CGAAGCTATTTCTTGTCTTGAGGCGTGACTGTTAAGAACCGTTGAAGTCCACTCAGTTTTAACCG
>Zm00001d014656_P006 peptide
MSHHDGSKPYQPRRGPERHPQPADGIAAPPPAAVAPSVEHLVAAAAEAEALNRFAAEQQQQLQGHEQEVGEEEEEEDEQE
DEMEEEDEDEHEGQHGGIGGEHVPMDADAAAAAAAAAVSQMDPHSALVAGTVPPMATNQLTLSFQGEVYVFDSVSPDKVQ
AVLLLLGGRELSSLGGASSSAPYSKRLNYPHRVASLMRFREKRKERNFDKKIRYSVRKEVALRMQRNRGQFTSSKPKPDE
IAASEMASADGSPNWALVEGRPPSAAECHHCGTNATATPMMRRGPDGPRTLCNACGLMWANKGLLRDVTKSPVPLQATQS
APHLDGGNGSAMSAPGSELENAAAAMTNGHESSSSGV
>Zm00001d014656_T007 cDNA
Chr5: 57723376..57727876
AAACCAGATTGAAATCCTAGGGGCTAGGGTTACCCTCCATCCCATCGTCCGATCCGATCCGATCCCGCCGCTCATCCATG
TCCCACCACGACGGAAGCAAGCCATACCAGCCGCGCCGGGGGCCCGAGCGGCACCCGCAGCCAGCGGATGGGATCGCCGC
CCCGCCTCCCGCCGCCGTTGCCCCGTCGGTGGAGCACCTCGTGGCGGCCGCCGCCGAGGCGGCGGCCGCAGTCTCGCAGA
TGGACCCGCACTCGGCGTTGGTGGCTGGGACCGTGCCACCCATGGCGACCAACCAGCTCACCCTCTCGTTCCAGGGCGAG
GTCTATGTGTTCGACTCCGTCTCCCCTGATAAGGTCCAAGCCGTGCTTTTGCTGCTTGGAGGGAGGGAGCTGAGCAGCCT
GGGCGGAGCGTCGTCCTCTGCACCTTATAGTAAGAGGTTGAATTATCCACATCGGGTGGCATCTCTGATGAGATTTAGGG
AAAAGCGGAAGGAGCGGAACTTTGATAAGAAGATCCGATACTCTGTCCGGAAGGAAGTTGCACTTAGGATGCAGCGTAAT
CGAGGTCAATTTACATCTTCAAAACCAAAGCCTGATGAAATAGCAGCATCAGAAATGGCATCCGCAGATGGCTCCCCGAA
TTGGGCATTAGTTGAAGGCCGACCTCCATCTGCTGCCGAATGTCATCACTGTGGTACTAATGCAACAGCTACACCAATGA
TGCGTCGCGGACCTGATGGACCAAGAACATTATGCAATGCATGTGGCCTCATGTGGGCAAATAAGGGTCTCCTGAGGGAC
GTAACAAAATCTCCTGTACCTCTCCAAGCTACGCAATCAGCTCCGCATCTAGATGGTGGTAATGGAAGCGCCATGTCTGC
GCCTGGCTCTGAGCTAGAGAATGCTGCAGCAGCCATGACCAATGGCCACGAGTCATCGAGTAGTGGTGTTTAATCCAGCG
ATGGGGTAAAAGCCACGAGGAGCATCATCCCGGAGTGATCTCTGCAGTAGGCGGTCAGCTGCTGTACTATAATTGAGTTG
GAGGCTGCTTAGATATGGAATCCTTCCATTTACCTTGGTGCCCTGTAGTTAAGAGCTATGACAGTGCTTTATCCCGTGTC
ATGTTTGCAGCCGCTGCGTCTATAACGAAGCTATTTCTTGTCTT
>Zm00001d014656_P007 peptide
MSHHDGSKPYQPRRGPERHPQPADGIAAPPPAAVAPSVEHLVAAAAEAAAAVSQMDPHSALVAGTVPPMATNQLTLSFQG
EVYVFDSVSPDKVQAVLLLLGGRELSSLGGASSSAPYSKRLNYPHRVASLMRFREKRKERNFDKKIRYSVRKEVALRMQR
NRGQFTSSKPKPDEIAASEMASADGSPNWALVEGRPPSAAECHHCGTNATATPMMRRGPDGPRTLCNACGLMWANKGLLR
DVTKSPVPLQATQSAPHLDGGNGSAMSAPGSELENAAAAMTNGHESSSSGV
>Zm00001d014656_T008 cDNA
Chr5: 57723379..57727926
ACGTCGTCTTCGTCTCTCCAACCCGGGGACATTTTCCACCCTCCAAATCGAAACCAGATTGAAATCCTAGGGGCTAGGGT
TACCCTCCATCCCATCGTCCGATCCGATCCGATCCCGCCGCTCATCCATGTCCCACCACGACGGAAGCAAGCCATACCAG
CCGCGCCGGGGGCCCGAGCGGCACCCGCAGCAGCTGCAGGGGCACGAGCAGGAGGTTGGGGAGGAAGAGGAGGAGGAGGA
CGAGCAGGAAGACGAGATGGAGGAGGAGGACGAGGATGAGCACGAAGGTCAGCACGGCGGCATCGGTGGGGAGCACGTTC
CCATGGACGCGGATGCTGCTGCCGCGGCGGCGGCGGCCGCAGTCTCGCAGATGGACCCGCACTCGGCGTTGGTGGCTGGG
ACCGTGCCACCCATGGCGACCAACCAGCTCACCCTCTCGTTCCAGGGCGAGGTCTATGTGTTCGACTCCGTCTCCCCTGA

TAAGGTCCAAGCCGTGCTTTTGCTGCTTGGAGGGAGGGAGCTGAGCAGCCTGGGCGGAGCGTCGTCCTCTGCACCTTATA
GTAAGAGGTTGAATTATCCACATCGGGTGGCATCTCTGATGAGATTTAGGGAAAAGCGGAAGGAGCGGAACTTTGATAAG
AAGATCCGATACTCTGTCCGGAAGGAAGTTGCACTTAGGATGCAGCGTAATCGAGGTCAATTTACATCTTCAAAACCAAA
GCCTGATGAAATAGCAGCATCAGAAATGGCATCCGCAGATGGCTCCCCGAATTGGGCATTAGTTGAAGGCCGACCTCCAT
CTGCTGCCGAATGTCATCACTGTGGTACTAATGCAACAGCTACACCAATGATGCGTCGCGGACCTGATGGACCAAGAACA
TTATGCAATGCATGTGGCCTCATGTGGGCAAATAAGGGTCTCCTGAGGGACGTAACAAAATCTCCTGTACCTCTCCAAGC
TACGCAATCAGCTCCGCATCTAGATGGTGGTGTAAGTTTTTCTTTCTTTGTTCGTTGCGTATGTTTTCTACCTAATCTTG
GTGGGGATCCTGTTTGGTTGAGCCTTATCCGATAAGTTGTAAGTGAAAATCCTTGATTCAGTTTTGTTGATTCCTTATCG
AATTATTTGCAACGCACCTTTCATGATCTCTGTCCTAGTTATGGTATCTGATTCTAACAAATCGATCTGAGAGGTCGTTT
TAACATTGTACTCAAGTGCAGTAGGCGCATGCTTTTTCCATCTATCCCTTTTTCCTCTTAGTGCTTGTTTGGGAGCAAAG
GTAATAGAGGGGGTCGAGGGGGCCAAAATCCGTTGCTATTCAGGCTTGTTTTGGGAGCAAAGGTAATAGTGGTGGTTGAG
GGGGCCAAAATCTCTTGCTATTCAATTTTGAATAGCAAGGAATTTTGGCTCCTCAACCCCCTCCATTACCCTTGGTCCTA
AACGAGCCCTTAGGCTATATCCATCAGATCCCTATTCCATCTCCTATCTCATCTCCTATTTTAAACCTCTCTCTGCAAAC
AGTGTCACCTAGCGTCCCCTATTTTATGCACTCTACTGGAGATAGTCTTATGCTTCAAACTAAGAAAAATGTTTATTGCT
CCACTAAACAAGAGTTACTGTTAATCTTCTCTTTACATTACTACTTCATAGTTCATAATGTTTCAGGGACAGTATAAGAT
GGATTACCTTATCATCCTTTCCCAATTTCTCTTCCACAGCTCCCATTATTTTCTCCTGTCTACCTTGTTTGTTTTGATAA
ATTGTATGCCTTCGTATTCTGAATAATTAACTGGTTGGAACTTAATTGCGGTCTACTTTTTTTCCAAGCAGAATGGAAGC
GCCATGTCTGCGCCTGGCTCTGAGCTAGAGAATGCTGCAGCAGCCATGACCAATGGCCACGAGTCATCGAGTAGTGGTGT
TTAATCCAGCGATGGGGTAAAAGCCACGAGGAGCATCATCCCGGAGTGATCTCTGCAGTAGGCGGTCAGCTGCTGTACTA
TAATTGAGTTGGAGGCTGCTTAGATATGGAATCCTTCCATTTACCTTGGTGCCCTGTAGTTAAGAGCTATGACAGTGCTT
TATCCCGTGTCATGTTTGCAGCCGCTGCGTCTATAACGAAGCTATTTCTTGT
>Zm00001d014656_P008 peptide
MSHHDGSKPYQPRRGPERHPQQLQGHEQEVGEEEEEEDEQEDEMEEEDEDEHEGQHGGIGGEHVPMDADAAAAAAAAAVS
QMDPHSALVAGTVPPMATNQLTLSFQGEVYVFDSVSPDKVQAVLLLLGGRELSSLGGASSSAPYSKRLNYPHRVASLMRF
REKRKERNFDKKIRYSVRKEVALRMQRNRGQFTSSKPKPDEIAASEMASADGSPNWALVEGRPPSAAECHHCGTNATATP
MMRRGPDGPRTLCNACGLMWANKGLLRDVTKSPVPLQATQSAPHLDGGVSFSFFVRCVCFLPNLGGDPVWLSLIR

ZmZML3 (TIFY23)

B73 RefGen_v3:
GRMZM2G080509 (zim20 - ZIM-transcription factor 20)
Chr6: 87857244..87864266

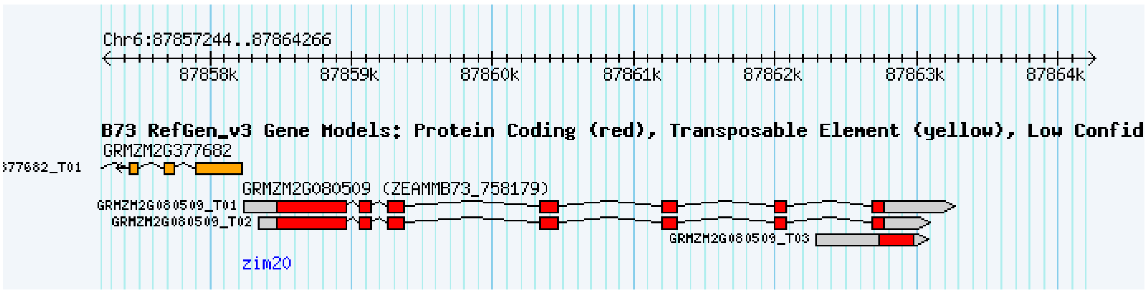

>GRMZM2G080509 Genomic DNA
GAGTCTACACTATTGACTTTAATAGATAGTAGAGAAGTAGCGACTAGGGACACTGTACTGCAAAAACTAGTGGATCCGAC
GTCTGTGATCTGTCAACAAAAACCCGGACCCACACGGCCGCCTCCTGCATGTCCACCCTCCAAATCGAAAACAGATCGAA
ATCCTAGGGTAGGGTTACCCTCCACCCCAATCCCCATCGTCCGTTCCGATCCGATCCCTCACCGTCCATCAATGTCCCAC

TCCCACCATGACGGAAGCAAGCCCTACCAGCCGCGCCGGGGGCCCGAGCGCCCCCGCAGCCGGCGGACGGGATAGCCGT
CCCGCCTCCTGCCGCCGTTGCCCCGTCGGTGGAGCACCTCGTGGCGGCCGCCGCCGAGGCGGAGGCGTTGAGTCGCTTGG
GCGCGGAACAACAGCAGCTGCTGCAGGGGCACGAGCAGGAGGTTGGGGAGGAAGAAGGGGAGGACGAGGAGGAAGACGAG
ATGGAGGACGACGACGACGACGACGATGAGCAGGAAGGGCAGCACGGCGGCATCGGTGTGGAGCACGTTCCCATGGACGC
AGATGCTGCTGCTGCCGCCGCCGTGGCGGCCGCTGGCGCGCAGATGGACCCGCACTCGGTGTTGGTGCCTGGGACCGTGC
CACCCATGGCGACCAACCAGCTCACCCTCTCGTTCCAGGGCGAGGTCTATGTATTCGACTCTGTCTCCCCTGATAAGGTG
CCTCTCCTCTTCTGCCCTCTGTAGACGTGAACTTGTTTTCTCTGCGTGTAGCCAATTCAATTCGCTAGACCGATGAGAGT
TTTTGGTTCTGTCTGCAGGTCCAAGCCGTGCTTCTGCTGCTTGGAGGCAGGGAGCTGAGTAGCCTGAGCGGAGCGTCGTC
CTCTGCACCTTATAGTAAGGTTGGGATATGTGATGTTGAATCTTATGTATAATAATGCCCGTGACTATGTCCATAGTAAC
TGCAAACTGGAATGGATAGTTCACATACCTGTGTTTGCTCGTCTCCTCCTTTCAGAGGTTGAACTTTCCACATCGGGTGG
CATCACTGATGAGATTTAGGGAAAAGCGGAAGGAGCGGAACTTTGATAAGAAGATCCGGTACAATGTCCGGAAGGAAGTT
GCACTTAGGTTGGTGATAGCTGTTTAACTTTGGCTGGTTACACTGTTCGTACTATCTAAAGTGGCATTAAGTTTGTTCTG
TCGGAACTTAATAAACTTAAATTGTGATTGGAAACCAAGGATTTGTTCTATTGTATGGTATCCTATTGCCCTTGAAACTC
AATACATCTCTTGTGTACATTTGTTCAAATGTAACTAGTTCCAAGTGGGAAGATGCATGTCCTAACTCCATGGCATTTTC
CAAGTTATTATGATTAGAATAAATCAATTATTCGTGAACCCTGTTTTTATGTGGTATTTTTAGTAGTGCCTAGAAGGGCA
GGATTGGTGTAGTGGTGAGAGTTGTGTCACCGAGTCACTGTTTGTGTGTTCGAAGCAGCCTCTCTGCATTTGCGTGAGAA
GGCTTGTCTCGGTTTATCCCTTCCCAAGTCCCAATCATGTGGGAGCCTCGAGCACTGGGTCTGTCATTTTCCTTAAGTAG
TGCCTATGTTTGGTAGTCCTTAGGAATGCTTTGAATCATGAGTGACTTTATGTAGTACTTCACAAGTTGACATGTATGTT
GCGCAAACAGTTGCTCACTGCTCAGCTTATAGCTATTTCTCATTGTTTCTTAATCAGTCCATTGAGTAGTTTTTGGTTAC
GTAGCAGCTTTGTCCATTCATGTTGTTTTAATGGTTGTGGAGCTATCTCATTGAGTCACCAGGTTTTTCGTTTGAAGCAG
TCTCTCCGCATTTGCGGGGGAAAGTTTGCCTCGGTTTATCCCTTCCCTAGACCCCCCACTCATGTGGGAGCCTCCGGCAC
TGGGTCTGCCCTAATGGTTGTGGATACTGATGTGGTGTTTCTTGATTTAAACATATGATTATGATGGTATGTTCGATGTT
GCAATTGGAGCCTGCTATATAGTCCTCTTCATCATAGATGATCATGCATATGCACTATTGGGATTTACACAGTTTATTTG
TATTTGTAGGATGCAGCGTAATAGAGGTCAGTTTACATCTTCAAAACCAAAGCCTGATGAAATAGCAGCATCAGAAATGG
CAGCTGCAGATGGCTCTCTGAATTGGGCACTAGTTGAAGGCCGACCTCCATCTGCTGCTGAGTGAGTTACAAAAGTGTTT
CCAGTTCGCTTGTGGTTTACATGTGTTTGACTTACGCAGCAATGTTTTTAGATGGTTAAAAGATGCTATTGCAGTTTTTT
CTTATTATTTCATTGTTGATGCCATGGACTTCAGATCCTTGTTTGTTCTCTGGAACAATTACCAGAAAGGAGTACAGATC
TGAGATATAATACAAAAAATCAATTAACCATGCTGTGGTTACTACACGTCTTGCTGGTTGACACCATAGTATGATAGTAC
CTCAATACTTCTCATCTAGCAGCACATAGAATGTTCATTTGTTACTCACATGATATGCCCCTACAATTTTAGCAATCAAA
TGTCGTGGTCTCGGTAGTTGTCGAAATCTTCGTTATTCTGCTTCCGAGATTACCATGCACATCAGTCATGAACTCGAGAT
TCTCCAAATTTGCTGGTTCTGGTGGTTACATAGGTCTGTACTTCACAGACTATTTTGTTTAAGATCAGCTTCCTGCTGGT
TTAGGCATGGTTTGGTTAATCAAACTTCAAACTGTTGTGCAACATTACAAGCAAGTTTGCTAATAGAAAAAGAACTGTTC
GTTGGTATCTAGCATGCATTGACACATGCTTCTTTCAAAAGGTTCTTGTTTTGTGGGTATTATGTTTTCATGTTGCATGT
GATTGGATTTCCTTTTAATCATTAAAAACTAAAAACGAACCATCATTTCTGAATTGTTCTGATGTCCAATTATTTCAGAT
GTCATCACTGTGGTATTAATGCAACAGCTACACCAATGATGCGTCGGGGACCTGATGGACCAAGAACACTATGCAACGCT
TGTGGCCTCATGTGGGCAAATAAGGTATTTGCGATTGCTTAGCAGTACAATTAGGATCCAAGATTTTATTGATCATTGAA
ACTTCGAAACTTATTTTTTTATAGCTTAGACTATGTAATGGAGTAATGGATCAAGAGGCGTGTGTGAATAAGAAAACCGC
GTGCCAGAACCATGACCCAGGCTAGTGGTCCCCTTTTAGCCTGCCCTTACATGCTTGGGAAGGCACTTGCTGTTTTTATA
AACTATAAAATGCACCCAACATAACATGTAACAACTGAGTATTAAAATTTCTTTAAAACAAAATCTTGATAGGTTCTTTG
CCTTGGGTGCTTAGTTGACAATCATGATGGTACACTGAAGTTTTGCTGACCCTGTAATTATTTACAAAATACTACATATC
AATCTATATTTATATTGTGCCACTGCAGTCTAATCTGTACTGAAGCACTCTTCTGGTGGTGCTGCGATTTTTTTGTCTGC
TTCTGTTGTAGTGATAAAATCCACTTTCTATTGAAGTTGATCTTTCGTTCTTTTTTGTTATGACTGCATTAGAGAAAATA
CCTCTTAATATACCTTATTGTTTCTGATCCCATCTACCCACTTTAAAAAATAAGAAACTTATGTTTGTTTTATTAGCTTG
ATCATGTAAATTTAGACGAAGTATTATGTAACTGCAAGTAAATCTAGGGTTGATCGTCCATTCACTCTTTATAGGGTCTC

CTGAGGGACCTATCCAAATCTCCTGTACCTCTTCACTCTATACAACAATCAGCTCCAATTCTAAATGGTGGTGTAAGTTT
TTCTTTTCTCTGCTCTTTGCATATGTTTTCTACCTAAGCTTGGTGGGGATCTTGTTTGGTTGAGATGTGTCATAAATTGT
AAGTGAAAATCCTTTGCTCAGAATAGTCGATTTCTTATCTAATTATTTGACTAGTAATCCACCTTGCATGATCTCTGTCC
TAGCGATGGTACCCAATTCTAACAGATGGATCTTATCTCTTTAACGTTGTCAAGTACAGTAGGTGCTTGCCATTTACATC
TATCATTTTCTCTCTTAAGACTTGAAACTGAAAAAGATATTTATTGCTCCACTAAACAATAGTTATTGCTAATCTTCTCT
TTCCATTACTACTGGTGAATTGTTATCTCTTTGCTTGGACTTGAGACTAGCTCAAAGTGATAGGGTTCTTCATAGTTCAT
AGCATTTTAGGGAATAGGGAATGTATAAAATGGATTATCTTATCACCCTCTTACAATGCCTCTTACACACCTCTTCCATT
GCCTCTTACACAGCTTCCATTACTTTCTCCTGCCTTGTTTACTTTGATAAACTTGTATGCCTTCAGTTTCTGAAATAATC
AACTGGTTGACACTTAAGTTGCTGTCTTTTTTTTTCTTCCAGCAGAACGGAAGTGCCATGTCTGCGCTTGGCTCTGAGCT
AGAGAATGCTGCAGCAGCCATGGGCAATGGCCACGAGCCATAAGGTAGTGGTGTTTAGCCATGTGCTAAAGCCACGAGCA
TCATCGCGCAATGATCTCTGCAGTAGGCGGTCAGTTGGAGCCTGCTTAGATATATGGAATCCTGCGCCTGTAGGTTTTAA
GAGCTGCGACAGTGTTTTGTCCCGTGTAGTCATGTTTGCAGCCGTCGCAGCTATAGCAAGTTTGTTTCTTGTCTTGAGTC
GTGACTGTTAAGAACTGATGAAGTTCATTACTTACGTGCGTGTAGTTATACTTAGATATGAGATGTTCTAGCCATGCAGC
AACGTTTTCTATCAATGTCAGTAATTAGGGAGCGAGCGTTTGTCTCATGGATTGGGCCTCGAAACAAAGAATCGACGCCC
AGGAGCTAAACTACAGAAACATTTCAGCTAGAGTAGGTCTGGAATGATGGAAGAGAAGACAACAATGCAAATGAAATTGA
TAAAATAACTTAATATAATATGGGACAACTATGGTTCAAATGAAAAAGTATGGTTGACTTAGA
>GRMZM2G080509_T01 cDNA
Chr6: 87858244..87863266
GAGTCTACACTATTGACTTTAATAGATAGTAGAGAAGTAGCGACTAGGGACACTGTACTGCAAAAACTAGTGGATCCGAC
GTCTGTGATCTGTCAACAAAAACCCGGACCCACACGGCCGCCTCCTGCATGTCCACCCTCCAAATCGAAAACAGATCGAA
ATCCTAGGGTAGGGTTACCCTCCACCCCAATCCCCATCGTCCGTTCCGATCCGATCCCTCACCGTCCATCAATGTCCCAC
TCCCACCATGACGGAAGCAAGCCCTACCAGCCGCGCCGGGGGCCCGAGCGCCCCCCGCAGCCGGCGGACGGGATAGCCGT
CCCGCCTCCTGCCGCCGTTGCCCCGTCGGTGGAGCACCTCGTGGCGGCCGCCGCCGAGGCGGAGGCGTTGAGTCGCTTGG
GCGCGGAACAACAGCAGCTGCTGCAGGGGCACGAGCAGGAGGTTGGGGAGGAAGAAGGGGAGGACGAGGAGGAAGACGAG
ATGGAGGACGACGACGACGACGACGATGAGCAGGAAGGGCAGCACGGCGGCATCGGTGTGGAGCACGTTCCCATGGACGC
AGATGCTGCTGCTGCCGCCGCCGTGGCGGCCGCTGGCGCGCAGATGGACCCGCACTCGGTGTTGGTGCCTGGGACCGTGC
CACCCATGGCGACCAACCAGCTCACCCTCTCGTTCCAGGGCGAGGTCTATGTATTCGACTCTGTCTCCCCTGATAAGGTC
CAAGCCGTGCTTCTGCTGCTTGGAGGCAGGGAGCTGAGTAGCCTGAGCGGAGCGTCGTCCTCTGCACCTTATAGTAAGAG
GTTGAACTTTCCACATCGGGTGGCATCACTGATGAGATTTAGGGAAAAGCGGAAGGAGCGGAACTTTGATAAGAAGATCC
GGTACAATGTCCGGAAGGAAGTTGCACTTAGGATGCAGCGTAATAGAGGTCAGTTTACATCTTCAAAACCAAAGCCTGAT
GAAATAGCAGCATCAGAAATGGCAGCTGCAGATGGCTCTCTGAATTGGGCACTAGTTGAAGGCCGACCTCCATCTGCTGC
TGAATGTCATCACTGTGGTATTAATGCAACAGCTACACCAATGATGCGTCGGGGACCTGATGGACCAAGAACACTATGCA
ACGCTTGTGGCCTCATGTGGGCAAATAAGGGTCTCCTGAGGGACCTATCCAAATCTCCTGTACCTCTTCACTCTATACAA
CAATCAGCTCCAATTCTAAATGGTGGTAACGGAAGTGCCATGTCTGCGCTTGGCTCTGAGCTAGAGAATGCTGCAGCAGC
CATGGGCAATGGCCACGAGCCATAAGGTAGTGGTGTTTAGCCATGTGCTAAAGCCACGAGCATCATCGCGCAATGATCTC
TGCAGTAGGCGGTCAGTTGGAGCCTGCTTAGATATATGGAATCCTGCGCCTGTAGGTTTTAAGAGCTGCGACAGTGTTTT
GTCCCGTGTAGTCATGTTTGCAGCCGTCGCAGCTATAGCAAGTTTGTTTCTTGTCTTGAGTCGTGACTGTTAAGAACTGA
TGAAGTTCATTACTTACGTGCGTGTAGTTATACTTAGATATGAGATGTTCTAGCCATGCAGCAACGTTTTCTATCAATGT
CAGTAATTAGGGAGCGAGCGTTTGTCTCATGGATTGGGCCTCGAAACAAAGAATCGACGCCCAGGAGCTAAACTACAGAA
ACATTTCAGCTAGAGTAGGTCTGGAATGATGGAAGAGAAGACAACAATGCAAATGAAATTGATAAAATAACTTAATATAA
TATGGGACAACTATGGTTCAAATGAAAAAGTATGGTTGACTTAGA
>GRMZM2G080509_P01 peptide
MSHSHHDGSKPYQPRRGPERPPQPADGIAVPPPAAVAPSVEHLVAAAAEAEALSRLGAEQQQLLQGHEQEVGEEEGEDEE
EDEMEDDDDDDDEQEGQHGGIGVEHVPMDADAAAAAAVAAAGAQMDPHSVLVPGTVPPMATNQLTLSFQGEVYVFDSVSP

DKVQAVLLLLGGRELSSLSGASSSAPYSKRLNFPHRVASLMRFREKRKERNFDKKIRYNVRKEVALRMQRNRGQFTSSKP
KPDEIAASEMAAADGSLNWALVEGRPPSAAECHHCGINATATPMMRRGPDGPRTLCNACGLMWANKGLLRDLSKSPVPLH
SIQQSAPILNGGNGSAMSALGSELENAAAAMGNGHEP
>GRMZM2G080509_T02 cDNA
Chr6: 87858349..87863093
GGACCCACACGGCCGCCTCCTGCATGTCCACCCTCCAAATCGAAAACAGATCGAAATCCTAGGGTAGGGTTACCCTCCAC
CCCAATCCCCATCGTCCGTTCCGATCCGATCCCTCACCGTCCATCAATGTCCCACTCCCACCATGACGGAAGCAAGCCCT
ACCAGCCGCGCCGGGGGCCCGAGCGCCCCCCGCAGCCGGCGGACGGGATAGCCGTCCCGCCTCCTGCCGCCGTTGCCCCG
TCGGTGGAGCACCTCGTGGCGGCCGCCGCCGAGGCGGAGGCGTTGAGTCGCTTGGGCGCGGAACAACAGCAGCTGCTGCA
GGGGCACGAGCAGGAGGTTGGGGAGGAAGAAGGGGAGGACGAGGAGGAAGACGAGATGGAGGACGACGACGACGACGACG
ATGAGCAGGAAGGGCAGCACGGCGGCATCGGTGTGGAGCACGTTCCCATGGACGCAGATGCTGCTGCTGCCGCCGCCGTG
GCGGCCGCTGGCGCGCAGATGGACCCGCACTCGGTGTTGGTGCCTGGGACCGTGCCACCCATGGCGACCAACCAGCTCAC
CCTCTCGTTCCAGGGCGAGGTCTATGTATTCGACTCTGTCTCCCCTGATAAGGTCCAAGCCGTGCTTCTGCTGCTTGGAG
GCAGGGAGCTGAGTAGCCTGAGCGGAGCGTCGTCCTCTGCACCTTATAGTAAGAGGTTGAACTTTCCACATCGGGTGGCA
TCACTGATGAGATTTAGGGAAAAGCGGAAGGAGCGGAACTTTGATAAGAAGATCCGGTACAATGTCCGGAAGGAAGTTGC
ACTTAGGATGCAGCGTAATAGAGGTCAGTTTACATCTTCAAAACCAAAGCCTGATGAAATAGCAGCATCAGAAATGGCAG
CTGCAGATGGCTCTCTGAATTGGGCACTAGTTGAAGGCCGACCTCCATCTGCTGCTGAATGTCATCACTGTGGTATTAAT
GCAACAGCTACACCAATGATGCGTCGGGGACCTGATGGACCAAGAACACTATGCAACGCTTGTGGCCTCATGTGGGCAAA
TAAGGGTCTCCTGAGGGACCTATCCAAATCTCCTGTACCTCTTCACTCTATACAACAATCAGCTCCAATTCTAAATGGTG
GTCAGAACGGAAGTGCCATGTCTGCGCTTGGCTCTGAGCTAGAGAATGCTGCAGCAGCCATGGGCAATGGCCACGAGCCA
TAAGGTAGTGGTGTTTAGCCATGTGCTAAAGCCACGAGCATCATCGCGCAATGATCTCTGCAGTAGGCGGTCAGTTGGAG
CCTGCTTAGATATATGGAATCCTGCGCCTGTAGGTTTTAAGAGCTGCGACAGTGTTTTGTCCCGTGTAGTCATGTTTGCA
GCCGTCGCAGCTATAGCAAGTTTGTTTCTTGTCTTGAGTCGTGACTGTTAAGAACTGATGAAGTTCATTACTTACGTGCG
TGTAGTTATACTTAGATATGAGATGTTCTAGCCATGCAGCAACGTTTTCTATCAATGTCAGTAATTAGGGAGCGAGCGTT
TGTCTCATGG
>GRMZM2G080509_P02 peptide
MSHSHHDGSKPYQPRRGPERPPQPADGIAVPPPAAVAPSVEHLVAAAAEAEALSRLGAEQQQLLQGHEQEVGEEEGEDEE
EDEMEDDDDDDDEQEGQHGGIGVEHVPMDADAAAAAAVAAAGAQMDPHSVLVPGTVPPMATNQLTLSFQGEVYVFDSVSP
DKVQAVLLLLGGRELSSLSGASSSAPYSKRLNFPHRVASLMRFREKRKERNFDKKIRYNVRKEVALRMQRNRGQFTSSKP
KPDEIAASEMAAADGSLNWALVEGRPPSAAECHHCGINATATPMMRRGPDGPRTLCNACGLMWANKGLLRDLSKSPVPLH
SIQQSAPILNGGQNGSAMSALGSELENAAAAMGNGHEP
>GRMZM2G080509_T03
Chr6: 87862289..87863078
GTTGTCAAGTACAGTAGGTGCTTGCCATTTACATCTATCATTTTCTCTCTTAAGACTTGAAACTGAAAAAGATATTTATT
GCTCCACTAAACAATAGTTATTGCTAATCTTCTCTTTCCATTACTACTGGTGAATTGTTATCTCTTTGCTTGGACTTGAG
ACTAGCTCAAAGTGATAGGGTTCTTCATAGTTCATAGCATTTTAGGGAATAGGGAATGTATAAAATGGATTATCTTATCA
CCCTCTTACAATGCCTCTTACACACCTCTTCCATTGCCTCTTACACAGCTTCCATTACTTTCTCCTGCCTTGTTTACTTT
GATAAACTTGTATGCCTTCAGTTTCTGAAATAATCAACTGGTTGACACTTAAGTTGCTGTCTTTTTTTTTCTTCCAGCAG
AACGGAAGTGCCATGTCTGCGCTTGGCTCTGAGCTAGAGAATGCTGCAGCAGCCATGGGCAATGGCCACGAGCCATAAGG
TAGTGGTGTTTAGCCATGTGCTAAAGCCACGAGCATCATCGCGCAATGATCTCTGCAGTAGGCGGTCAGTTGGAGCCTGC
TTAGATATATGGAATCCTGCGCCTGTAGGTTTTAAGAGCTGCGACAGTGTTTTGTCCCGTGTAGTCATGTTTGCAGCCGT
CGCAGCTATAGCAAGTTTGTTTCTTGTCTTGAGTCGTGACTGTTAAGAACTGATGAAGTTCATTACTTACGTGCGTGTAG
TTATACTTAGATATGAGATGTTCTAGCCATGCAGCAACGTTTTCTATCAATGTCAGTAATTAGGGAGCGA
>GRMZM2G080509_P03 peptide

MLQQPWAMATSHKVVVFSHVLKPRASSRNDLCSRRSVGACLDIWNPAPVGFKSCDSVLSRVVMFAAVAAIASLFLVLSRD
C

AGPv4:

Zm00001d036494 (zim20 - ZIM-transcription factor 20)

Chr6: 90505221..90512243

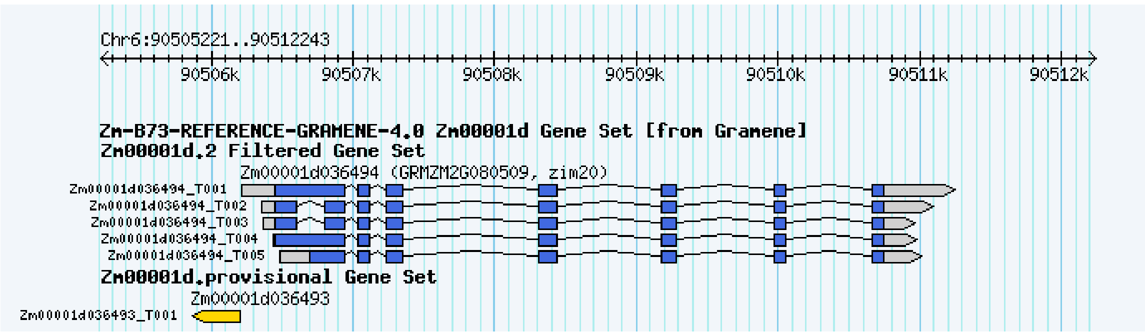

>Zm00001d036494 Genomic DNA

GAGTCTACACTATTGACTTTAATAGATAGTAGAGAAGTAGCGACTAGGGACACTGTACTGCAAAAACTAGTGGATCCGAC
GTCTGTGATCTGTCAACAAAAACCCGGACCCACACGGCCGCCTCCTGCATGTCCACCCTCCAAATCGAAAACAGATCGAA
ATCCTAGGGTAGGGTTACCCTCCACCCCAATCCCCATCGTCCGTTCCGATCCGATCCCTCACCGTCCATCAATGTCCCAC
TCCCACCATGACGGAAGCAAGCCCTACCAGCCGCGCCGGGGGCCCGAGCGCCCCCCGCAGCCGGCGGACGGGATAGCCGT
CCCGCCTCCTGCCGCCGTTGCCCCGTCGGTGGAGCACCTCGTGGCGGCCGCCGCCGAGGCGGAGGCGTTGAGTCGCTTGG
GCGCGGAACAACAGCAGCTGCTGCAGGGGCACGAGCAGGAGGTTGGGGAGGAAGAAGGGGAGGACGAGGAGGAAGACGAG
ATGGAGGACGACGACGACGACGACGATGAGCAGGAAGGGCAGCACGGCGGCATCGGTGTGGAGCACGTTCCCATGGACGC
AGATGCTGCTGCTGCCGCCGCCGTGGCGGCCGCTGGCGCGCAGATGGACCCGCACTCGGTGTTGGTGCCTGGGACCGTGC
CACCCATGGCGACCAACCAGCTCACCCTCTCGTTCCAGGGCGAGGTCTATGTATTCGACTCTGTCTCCCCTGATAAGGTG
CCTCTCCTCTTCTGCCCTCTGTAGACGTGAACTTGTTTTCTCTGCGTGTAGCCAATTCAATTCGCTAGACCGATGAGAGT
TTTTGGTTCTGTCTGCAGGTCCAAGCCGTGCTTCTGCTGCTTGGAGGCAGGGAGCTGAGTAGCCTGAGCGGAGCGTCGTC
CTCTGCACCTTATAGTAAGGTTGGGATATGTGATGTTGAATCTTATGTATAATAATGCCCGTGACTATGTCCATAGTAAC
TGCAAACTGGAATGGATAGTTCACATACCTGTGTTTGCTCGTCTCCTCCTTTCAGAGGTTGAACTTTCCACATCGGGTGG
CATCACTGATGAGATTTAGGGAAAAGCGGAAGGAGCGGAACTTTGATAAGAAGATCCGGTACAATGTCCGGAAGGAAGTT
GCACTTAGGTTGGTGATAGCTGTTTAACTTTGGCTGGTTACACTGTTCGTACTATCTAAAGTGGCATTAAGTTTGTTCTG
TCGGAACTTAATAAACTTAAATTGTGATTGGAAACCAAGGATTTGTTCTATTGTATGGTATCCTATTGCCCTTGAAACTC
AATACATCTCTTGTGTACATTTGTTCAAATGTAACTAGTTCCAAGTGGGAAGATGCATGTCCTAACTCCATGGCATTTTC
CAAGTTATTATGATTAGAATAAATCAATTATTCGTGAACCCTGTTTTTATGTGGTATTTTTAGTAGTGCCTAGAAGGGCA
GGATTGGTGTAGTGGTGAGAGTTGTGTCACCGAGTCACTGTTTGTGTGTTCGAAGCAGCCTCTCTGCATTTGCGTGAGAA
GGCTTGTCTCGGTTTATCCCTTCCCAAGTCCCAATCATGTGGGAGCCTCGAGCACTGGGTCTGTCATTTTCCTTAAGTAG
TGCCTATGTTTGGTAGTCCTTAGGAATGCTTTGAATCATGAGTGACTTTATGTAGTACTTCACAAGTTGACATGTATGTT
GCGCAAACAGTTGCTCACTGCTCAGCTTATAGCTATTTCTCATTGTTTCTTAATCAGTCCATTGAGTAGTTTTTGGTTAC
GTAGCAGCTTTGTCCATTCATGTTGTTTTAATGGTTGTGGAGCTATCTCATTGAGTCACCAGGTTTTTCGTTTGAAGCAG
TCTCTCCGCATTTGCGGGGGAAAGTTTGCCTCGGTTTATCCCTTCCCTAGACCCCCCACTCATGTGGGAGCCTCCGGCAC
TGGGTCTGCCCTAATGGTTGTGGATACTGATGTGGTGTTTCTTGATTTAAACATATGATTATGATGGTATGTTCGATGTT
GCAATTGGAGCCTGCTATATAGTCCTCTTCATCATAGATGATCATGCATATGCACTATTGGGATTTACACAGTTTATTTG
TATTTGTAGGATGCAGCGTAATAGAGGTCAGTTTACATCTTCAAAACCAAAGCCTGATGAAATAGCAGCATCAGAAATGG
CAGCTGCAGATGGCTCTCTGAATTGGGCACTAGTTGAAGGCCGACCTCCATCTGCTGCTGAGTGAGTTACAAAAGTGTTT
CCAGTTCGCTTGTGGTTTACATGTGTTTGACTTACGCAGCAATGTTTTTAGATGGTTAAAAGATGCTATTGCAGTTTTTT

CTTATTATTTCATTGTTGATGCCATGGACTTCAGATCCTTGTTTGTTCTCTGGAACAATTACCAGAAAGGAGTACAGATC
TGAGATATAATACAAAAAATCAATTAACCATGCTGTGGTTACTACACGTCTTGCTGGTTGACACCATAGTATGATAGTAC
CTCAATACTTCTCATCTAGCAGCACATAGAATGTTCATTTGTTACTCACATGATATGCCCCTACAATTTTAGCAATCAAA
TGTCGTGGTCTCGGTAGTTGTCGAAATCTTCGTTATTCTGCTTCCGAGATTACCATGCACATCAGTCATGAACTCGAGAT
TCTCCAAATTTGCTGGTTCTGGTGGTTACATAGGTCTGTACTTCACAGACTATTTTGTTTAAGATCAGCTTCCTGCTGGT
TTAGGCATGGTTTGGTTAATCAAACTTCAAACTGTTGTGCAACATTACAAGCAAGTTTGCTAATAGAAAAAGAACTGTTC
GTTGGTATCTAGCATGCATTGACACATGCTTCTTTCAAAAGGTTCTTGTTTTGTGGGTATTATGTTTTCATGTTGCATGT
GATTGGATTTCCTTTTAATCATTAAAAACTAAAAACGAACCATCATTTCTGAATTGTTCTGATGTCCAATTATTTCAGAT
GTCATCACTGTGGTATTAATGCAACAGCTACACCAATGATGCGTCGGGGACCTGATGGACCAAGAACACTATGCAACGCT
TGTGGCCTCATGTGGGCAAATAAGGTATTTGCGATTGCTTAGCAGTACAATTAGGATCCAAGATTTTATTGATCATTGAA
ACTTCGAAACTTATTTTTTTATAGCTTAGACTATGTAATGGAGTAATGGATCAAGAGGCGTGTGTGAATAAGAAAACCGC
GTGCCAGAACCATGACCCAGGCTAGTGGTCCCCTTTTAGCCTGCCCTTACATGCTTGGGAAGGCACTTGCTGTTTTTATA
AACTATAAAATGCACCCAACATAACATGTAACAACTGAGTATTAAAATTTCTTTAAAACAAAATCTTGATAGGTTCTTTG
CCTTGGGTGCTTAGTTGACAATCATGATGGTACACTGAAGTTTTGCTGACCCTGTAATTATTTACAAAATACTACATATC
AATCTATATTTATATTGTGCCACTGCAGTCTAATCTGTACTGAAGCACTCTTCTGGTGGTGCTGCGATTTTTTTGTCTGC
TTCTGTTGTAGTGATAAAATCCACTTTCTATTGAAGTTGATCTTTCGTTCTTTTTTGTTATGACTGCATTAGAGAAAATA
CCTCTTAATATACCTTATTGTTTCTGATCCCATCTACCCACTTTAAAAAATAAGAAACTTATGTTTGTTTTATTAGCTTG
ATCATGTAAATTTAGACGAAGTATTATGTAACTGCAAGTAAATCTAGGGTTGATCGTCCATTCACTCTTTATAGGGTCTC
CTGAGGGACCTATCCAAATCTCCTGTACCTCTTCACTCTATACAACAATCAGCTCCAATTCTAAATGGTGGTGTAAGTTT
TTCTTTTCTCTGCTCTTTGCATATGTTTTCTACCTAAGCTTGGTGGGGATCTTGTTTGGTTGAGATGTGTCATAAATTGT
AAGTGAAAATCCTTTGCTCAGAATAGTCGATTTCTTATCTAATTATTTGACTAGTAATCCACCTTGCATGATCTCTGTCC
TAGCGATGGTACCCAATTCTAACAGATGGATCTTATCTCTTTAACGTTGTCAAGTACAGTAGGTGCTTGCCATTTACATC
TATCATTTTCTCTCTTAAGACTTGAAACTGAAAAAGATATTTATTGCTCCACTAAACAATAGTTATTGCTAATCTTCTCT
TTCCATTACTACTGGTGAATTGTTATCTCTTTGCTTGGACTTGAGACTAGCTCAAAGTGATAGGGTTCTTCATAGTTCAT
AGCATTTTAGGGAATAGGGAATGTATAAAATGGATTATCTTATCACCCTCTTACAATGCCTCTTACACACCTCTTCCATT
GCCTCTTACACAGCTTCCATTACTTTCTCCTGCCTTGTTTACTTTGATAAACTTGTATGCCTTCAGTTTCTGAAATAATC
AACTGGTTGACACTTAAGTTGCTGTCTTTTTTTTTCTTCCAGCAGAACGGAAGTGCCATGTCTGCGCTTGGCTCTGAGCT
AGAGAATGCTGCAGCAGCCATGGGCAATGGCCACGAGCCATAAGGTAGTGGTGTTTAGCCATGTGCTAAAGCCACGAGCA
TCATCGCGCAATGATCTCTGCAGTAGGCGGTCAGTTGGAGCCTGCTTAGATATATGGAATCCTGCGCCTGTAGGTTTTAA
GAGCTGCGACAGTGTTTTGTCCCGTGTAGTCATGTTTGCAGCCGTCGCAGCTATAGCAAGTTTGTTTCTTGTCTTGAGTC
GTGACTGTTAAGAACTGATGAAGTTCATTACTTACGTGCGTGTAGTTATACTTAGATATGAGATGTTCTAGCCATGCAGC
AACGTTTTCTATCAATGTCAGTAATTAGGGAGCGAGCGTTTGTCTCATGGATTGGGCCTCGAAACAAAGAATCGACGCCC
AGGAGCTAAACTACAGAAACATTTCAGCTAGAGTAGGTCTGGAATGATGGAAGAGAAGACAACAATGCAAATGAAATTGA
TAAAATAACTTAATATAATATGGGACAACTATGGTTCAAATGAAAAAGTATGGTTGACTTAGA
>Zm00001d036494_T001 cDNA
Chr6: 90506221..90511243
GAGTCTACACTATTGACTTTAATAGATAGTAGAGAAGTAGCGACTAGGGACACTGTACTGCAAAAACTAGTGGATCCGAC
GTCTGTGATCTGTCAACAAAAACCCGGACCCACACGGCCGCCTCCTGCATGTCCACCCTCCAAATCGAAAACAGATCGAA
ATCCTAGGGTAGGGTTACCCTCCACCCCAATCCCCATCGTCCGTTCCGATCCGATCCCTCACCGTCCATCAATGTCCCAC
TCCCACCATGACGGAAGCAAGCCCTACCAGCCGCGCCGGGGGCCCGAGCGCCCCCCGCAGCCGGCGGACGGGATAGCCGT
CCCGCCTCCTGCCGCCGTTGCCCCGTCGGTGGAGCACCTCGTGGCGGCCGCCGCCGAGGCGGAGGCGTTGAGTCGCTTGG
GCGCGGAACAACAGCAGCTGCTGCAGGGGCACGAGCAGGAGGTTGGGGAGGAAGAAGGGGAGGACGAGGAGGAAGACGAG
ATGGAGGACGACGACGACGACGACGATGAGCAGGAAGGGCAGCACGGCGGCATCGGTGTGGAGCACGTTCCCATGGACGC
AGATGCTGCTGCTGCCGCCGCCGTGGCGGCCGCTGGCGCGCAGATGGACCCGCACTCGGTGTTGGTGCCTGGGACCGTGC

CACCCATGGCGACCAACCAGCTCACCCTCTCGTTCCAGGGCGAGGTCTATGTATTCGACTCTGTCTCCCCTGATAAGGTC
CAAGCCGTGCTTCTGCTGCTTGGAGGCAGGGAGCTGAGTAGCCTGAGCGGAGCGTCGTCCTCTGCACCTTATAGTAAGAG
GTTGAACTTTCCACATCGGGTGGCATCACTGATGAGATTTAGGGAAAAGCGGAAGGAGCGGAACTTTGATAAGAAGATCC
GGTACAATGTCCGGAAGGAAGTTGCACTTAGGATGCAGCGTAATAGAGGTCAGTTTACATCTTCAAAACCAAAGCCTGAT
GAAATAGCAGCATCAGAAATGGCAGCTGCAGATGGCTCTCTGAATTGGGCACTAGTTGAAGGCCGACCTCCATCTGCTGC
TGAATGTCATCACTGTGGTATTAATGCAACAGCTACACCAATGATGCGTCGGGGACCTGATGGACCAAGAACACTATGCA
ACGCTTGTGGCCTCATGTGGGCAAATAAGGGTCTCCTGAGGGACCTATCCAAATCTCCTGTACCTCTTCACTCTATACAA
CAATCAGCTCCAATTCTAAATGGTGGTCAGAACGGAAGTGCCATGTCTGCGCTTGGCTCTGAGCTAGAGAATGCTGCAGC
AGCCATGGGCAATGGCCACGAGCCATAAGGTAGTGGTGTTTAGCCATGTGCTAAAGCCACGAGCATCATCGCGCAATGAT
CTCTGCAGTAGGCGGTCAGTTGGAGCCTGCTTAGATATATGGAATCCTGCGCCTGTAGGTTTTAAGAGCTGCGACAGTGT
TTTGTCCCGTGTAGTCATGTTTGCAGCCGTCGCAGCTATAGCAAGTTTGTTTCTTGTCTTGAGTCGTGACTGTTAAGAAC
TGATGAAGTTCATTACTTACGTGCGTGTAGTTATACTTAGATATGAGATGTTCTAGCCATGCAGCAACGTTTTCTATCAA
TGTCAGTAATTAGGGAGCGAGCGTTTGTCTCATGGATTGGGCCTCGAAACAAAGAATCGACGCCCAGGAGCTAAACTACA
GAAACATTTCAGCTAGAGTAGGTCTGGAATGATGGAAGAGAAGACAACAATGCAAATGAAATTGATAAAATAACTTAATA
TAATATGGGACAACTATGGTTCAAATGAAAAAGTATGGTTGACTTAGA
>Zm00001d036494_P001 peptide
MSHSHHDGSKPYQPRRGPERPPQPADGIAVPPPAAVAPSVEHLVAAAAEAEALSRLGAEQQQLLQGHEQEVGEEEGEDEE
EDEMEDDDDDDDEQEGQHGGIGVEHVPMDADAAAAAAVAAAGAQMDPHSVLVPGTVPPMATNQLTLSFQGEVYVFDSVSP
DKVQAVLLLLGGRELSSLSGASSSAPYSKRLNFPHRVASLMRFREKRKERNFDKKIRYNVRKEVALRMQRNRGQFTSSKP
KPDEIAASEMAAADGSLNWALVEGRPPSAAECHHCGINATATPMMRRGPDGPRTLCNACGLMWANKGLLRDLSKSPVPLH
SIQQSAPILNGGQNGSAMSALGSELENAAAAMGNGHEP
>Zm00001d036494_T002 cDNA
Chr6: 90506358..90511094
CTCCAAATCGAAAACAGATCGAAATCCTAGGGTAGGGTTACCCTCCACCCCAATCCCCATCGTCCGTTCCGATCCGATCC
CTCACCGTCCATCAATGTCCCACTCCCACCATGACGGAAGCAAGCCCTACCAGCCGCGCCGGGGGCCCGAGCGCCCCCCG
CAGCCGGCGGACGGGATAGCCGTCCCGCCTCCTGCCGCCGTTGCCCCGTCGGTGGAGCACCTCGTGGCGGCCGCCGCCGA
GGCGGAGGCGGCGGCCGCTGGCGCGCAGATGGACCCGCACTCGGTGTTGGTGCCTGGGACCGTGCCACCCATGGCGACCA
ACCAGCTCACCCTCTCGTTCCAGGGCGAGGTCTATGTATTCGACTCTGTCTCCCCTGATAAGGTCCAAGCCGTGCTTCTG
CTGCTTGGAGGCAGGGAGCTGAGTAGCCTGAGCGGAGCGTCGTCCTCTGCACCTTATAGTAAGAGGTTGAACTTTCCACA
TCGGGTGGCATCACTGATGAGATTTAGGGAAAAGCGGAAGGAGCGGAACTTTGATAAGAAGATCCGGTACAATGTCCGGA
AGGAAGTTGCACTTAGGATGCAGCGTAATAGAGGTCAGTTTACATCTTCAAAACCAAAGCCTGATGAAATAGCAGCATCA
GAAATGGCAGCTGCAGATGGCTCTCTGAATTGGGCACTAGTTGAAGGCCGACCTCCATCTGCTGCTGAATGTCATCACTG
TGGTATTAATGCAACAGCTACACCAATGATGCGTCGGGGACCTGATGGACCAAGAACACTATGCAACGCTTGTGGCCTCA
TGTGGGCAAATAAGGGTCTCCTGAGGGACCTATCCAAATCTCCTGTACCTCTTCACTCTATACAACAATCAGCTCCAATT
CTAAATGGTGGTAACGGAAGTGCCATGTCTGCGCTTGGCTCTGAGCTAGAGAATGCTGCAGCAGCCATGGGCAATGGCCA
CGAGCCATAAGGTAGTGGTGTTTAGCCATGTGCTAAAGCCACGAGCATCATCGCGCAATGATCTCTGCAGTAGGCGGTCA
GTTGGAGCCTGCTTAGATATATGGAATCCTGCGCCTGTAGGTTTTAAGAGCTGCGACAGTGTTTTGTCCCGTGTAGTCAT
GTTTGCAGCCGTCGCAGCTATAGCAAGTTTGTTTCTTGTCTTGAGTCGTGACTGTTAAGAACTGATGAAGTTCATTACTT
ACGTGCGTGTAGTTATACTTAGATATGAGATGTTCTAGCCATGCAGCAACGTTTTCTATCAATGTCAGTAATTAGGGAGC
GAGCGTTTGTCTCATGGATTGGGCCTCGAAACAAAGAATCG
>Zm00001d036494_P002 peptide
MSHSHHDGSKPYQPRRGPERPPQPADGIAVPPPAAVAPSVEHLVAAAAEAEAAAAGAQMDPHSVLVPGTVPPMATNQLTL
SFQGEVYVFDSVSPDKVQAVLLLLGGRELSSLSGASSSAPYSKRLNFPHRVASLMRFREKRKERNFDKKIRYNVRKEVAL
RMQRNRGQFTSSKPKPDEIAASEMAAADGSLNWALVEGRPPSAAECHHCGINATATPMMRRGPDGPRTLCNACGLMWANK

GLLRDLSKSPVPLHSIQQSAPILNGGNGSAMSALGSELENAAAAMGNGHEP
>Zm00001d036494_T003 cDNA
Chr6: 90506368..90510973
AAAACAGATCGAAATCCTAGGGTAGGGTTACCCTCCACCCCAATCCCCATCGTCCGTTCCGATCCGATCCCTCACCGTCC
ATCAATGTCCCACTCCCACCATGACGGAAGCAAGCCCTACCAGCCGCGCCGGGGGCCCGAGCGCCCCCCGCAGCCGGCGG
ACGGGATAGCCGTCCCGCCTCCTGCCGCCGTTGCCCCGTCGGTGGAGCACCTCGTGGCGGCCGCCGCCGAGGCGGCGGCC
GCTGGCGCGCAGATGGACCCGCACTCGGTGTTGGTGCCTGGGACCGTGCCACCCATGGCGACCAACCAGCTCACCCTCTC
GTTCCAGGGCGAGGTCTATGTATTCGACTCTGTCTCCCCTGATAAGGTCCAAGCCGTGCTTCTGCTGCTTGGAGGCAGGG
AGCTGAGTAGCCTGAGCGGAGCGTCGTCCTCTGCACCTTATAGTAAGAGGTTGAACTTTCCACATCGGGTGGCATCACTG
ATGAGATTTAGGGAAAAGCGGAAGGAGCGGAACTTTGATAAGAAGATCCGGTACAATGTCCGGAAGGAAGTTGCACTTAG
GATGCAGCGTAATAGAGGTCAGTTTACATCTTCAAAACCAAAGCCTGATGAAATAGCAGCATCAGAAATGGCAGCTGCAG
ATGGCTCTCTGAATTGGGCACTAGTTGAAGGCCGACCTCCATCTGCTGCTGAATGTCATCACTGTGGTATTAATGCAACA
GCTACACCAATGATGCGTCGGGGACCTGATGGACCAAGAACACTATGCAACGCTTGTGGCCTCATGTGGGCAAATAAGGG
TCTCCTGAGGGACCTATCCAAATCTCCTGTACCTCTTCACTCTATACAACAATCAGCTCCAATTCTAAATGGTGGTAACG
GAAGTGCCATGTCTGCGCTTGGCTCTGAGCTAGAGAATGCTGCAGCAGCCATGGGCAATGGCCACGAGCCATAAGGTAGT
GGTGTTTAGCCATGTGCTAAAGCCACGAGCATCATCGCGCAATGATCTCTGCAGTAGGCGGTCAGTTGGAGCCTGCTTAG
ATATATGGAATCCTGCGCCTGTAGGTTTTAAGAGCTGCGACAGTGTTTTGTCCCGTGTAGTCATGTTTGCAGCCGTCGCA
GCTATAGCAAGTTTGTTTCTTGTCTTGAGTCGTGACTGTTAAGAACTGATGAAGTTCATTACTT
>Zm00001d036494_P003 peptide
MSHSHHDGSKPYQPRRGPERPPQPADGIAVPPPAAVAPSVEHLVAAAAEAAAAGAQMDPHSVLVPGTVPPMATNQLTLSF
QGEVYVFDSVSPDKVQAVLLLLGGRELSSLSGASSSAPYSKRLNFPHRVASLMRFREKRKERNFDKKIRYNVRKEVALRM
QRNRGQFTSSKPKPDEIAASEMAAADGSLNWALVEGRPPSAAECHHCGINATATPMMRRGPDGPRTLCNACGLMWANKGL
LRDLSKSPVPLHSIQQSAPILNGGNGSAMSALGSELENAAAAMGNGHEP
>Zm00001d036494_T004 cDNA
Chr6: 90506438..90510982
CTCACCGTCCATCAATGTCCCACTCCCACCATGACGGAAGCAAGCCCTACCAGCCGCGCCGGGGGCCCGAGCGCCCCCCG
CAGCCGGCGGACGGGATAGCCGTCCCGCCTCCTGCCGCCGTTGCCCCGTCGGTGGAGCACCTCGTGGCGGCCGCCGCCGA
GGCGGAGGCGTTGAGTCGCTTGGGCGCGGAACAACAGCAGCTGCTGCAGGGGCACGAGCAGGAGGTTGGGGAGGAAGAAG
GGGAGGACGAGGAGGAAGACGAGATGGAGGACGACGACGACGACGACGATGAGCAGGAAGGGCAGCACGGCGGCATCGGT
GTGGAGCACGTTCCCATGGACGCAGATGCTGCTGCTGCCGCCGCCGTGGCGGCCGCTGGCGCGCAGATGGACCCGCACTC
GGTGTTGGTGCCTGGGACCGTGCCACCCATGGCGACCAACCAGCTCACCCTCTCGTTCCAGGGCGAGGTCTATGTATTCG
ACTCTGTCTCCCCTGATAAGGTCCAAGCCGTGCTTCTGCTGCTTGGAGGCAGGGAGCTGAGTAGCCTGAGCGGAGCGTCG
TCCTCTGCACCTTATAGTAAGAGGTTGAACTTTCCACATCGGGTGGCATCACTGATGAGATTTAGGGAAAAGCGGAAGGA
GCGGAACTTTGATAAGAAGATCCGGTACAATGTCCGGAAGGAAGTTGCACTTAGGATGCAGCGTAATAGAGGTCAGTTTA
CATCTTCAAAACCAAAGCCTGATGAAATAGCAGCATCAGAAATGGCAGCTGCAGATGGCTCTCTGAATTGGGCACTAGTT
GAAGGCCGACCTCCATCTGCTGCTGAATGTCATCACTGTGGTATTAATGCAACAGCTACACCAATGATGCGTCGGGGACC
TGATGGACCAAGAACACTATGCAACGCTTGTGGCCTCATGTGGGCAAATAAGGGTCTCCTGAGGGACCTATCCAAATCTC
CTGTACCTCTTCACTCTATACAACAATCAGCTCCAATTCTAAATGGTGGTAACGGAAGTGCCATGTCTGCGCTTGGCTCT
GAGCTAGAGAATGCTGCAGCAGCCATGGGCAATGGCCACGAGCCATAAGGTAGTGGTGTTTAGCCATGTGCTAAAGCCAC
GAGCATCATCGCGCAATGATCTCTGCAGTAGGCGGTCAGTTGGAGCCTGCTTAGATATATGGAATCCTGCGCCTGTAGGT
TTTAAGAGCTGCGACAGTGTTTTGTCCCGTGTAGTCATGTTTGCAGCCGTCGCAGCTATAGCAAGTTTGTTTCTTGTCTT
GAGTCGTGACTGTTAAGAACTGATGAAGTTCATTACTTACGTGCGTG
>Zm00001d036494_P004 peptide
MSHSHHDGSKPYQPRRGPERPPQPADGIAVPPPAAVAPSVEHLVAAAAEAEALSRLGAEQQQLLQGHEQEVGEEEGEDEE

EDEMEDDDDDDDEQEGQHGGIGVEHVPMDADAAAAAAVAAAGAQMDPHSVLVPGTVPPMATNQLTLSFQGEVYVFDSVSP
DKVQAVLLLLGGRELSSLSGASSSAPYSKRLNFPHRVASLMRFREKRKERNFDKKIRYNVRKEVALRMQRNRGQFTSSKP
KPDEIAASEMAAADGSLNWALVEGRPPSAAECHHCGINATATPMMRRGPDGPRTLCNACGLMWANKGLLRDLSKSPVPLH
SIQQSAPILNGGNGSAMSALGSELENAAAAMGNGHEP
>Zm00001d036494_T005 cDNA
Chr6: 90506484..90511012
CTACCAGCCGCGCCGGGGGCCCGAGCGCCCCCCGCAGCCGGCGGACGGGATAGCCGTCCCGCCTCCTGCCGCCGTTGCCC
CGTCGGTGGAGCACCTCGTGGCGGCCGCCGCCGAGGCGGAGGCGTTGAGTCGCTTGGGCGCGGAACAACAGCAGCTGCTG
CAGGGGCACGAGCAGGAGGTTGGGGAGGAAGAAGGGGAGGACGAGGAGGAAGACGAGATGGAGGACGACGACGACGACGA
CGATGAGCAGGAAGGGCAGCACGGCGGCATCGGTGTGGAGCACGTTCCCATGGACGCAGATGCTGCTGCTGCCGCCGCCG
TGGCGGCCGCTGGCGCGCAGATGGACCCGCACTCGGTGTTGGTGCCTGGGACCGTGCCACCCATGGCGACCAACCAGCTC
ACCCTCTCGTTCCAGGGCGAGGTCTATGTATTCGACTCTGTCTCCCCTGATAAGGTCCAAGCCGTGCTTCTGCTGCTTGG
AGGCAGGGAGCTGAGTAGCCTGAGCGGAGCGTCGTCCTCTGCACCTTATAGTAAGAGGTTGAACTTTCCACATCGGGTGG
CATCACTGATGAGATTTAGGGAAAAGCGGAAGGAGCGGAACTTTGATAAGAAGATCCGGTACAATGTCCGGAAGGAAGTT
GCACTTAGGATGCAGCGTAATAGAGGTCAGTTTACATCTTCAAAACCAAAGCCTGATGAAATAGCAGCATCAGAAATGGC
AGCTGCAGATGGCTCTCTGAATTGGGCACTAGTTGAAGGCCGACCTCCATCTGCTGCTGAATGTCATCACTGTGGTATTA
ATGCAACAGCTACACCAATGATGCGTCGGGGACCTGATGGACCAAGAACACTATGCAACGCTTGTGGCCTCATGTGGGCA
AATAAGGGTCTCCTGAGGGACCTATCCAAATCTCCTGTACCTCTTCACTCTATACAACAATCAGCTCCAATTCTAAATGG
TGGTAACGGAAGTGCCATGTCTGCGCTTGGCTCTGAGCTAGAGAATGCTGCAGCAGCCATGGGCAATGGCCACGAGCCAT
AAGGTAGTGGTGTTTAGCCATGTGCTAAAGCCACGAGCATCATCGCGCAATGATCTCTGCAGTAGGCGGTCAGTTGGAGC
CTGCTTAGATATATGGAATCCTGCGCCTGTAGGTTTTAAGAGCTGCGACAGTGTTTTGTCCCGTGTAGTCATGTTTGCAG
CCGTCGCAGCTATAGCAAGTTTGTTTCTTGTCTTGAGTCGTGACTGTTAAGAACTGATGAAGTTCATTACTTACGTGCGT
GTAGTTATACTTAGATATGAGATGTTCTAGC
>Zm00001d036494_P005 peptide
MEDDDDDDDEQEGQHGGIGVEHVPMDADAAAAAAVAAAGAQMDPHSVLVPGTVPPMATNQLTLSFQGEVYVFDSVSPDKV
QAVLLLLGGRELSSLSGASSSAPYSKRLNFPHRVASLMRFREKRKERNFDKKIRYNVRKEVALRMQRNRGQFTSSKPKPD
EIAASEMAAADGSLNWALVEGRPPSAAECHHCGINATATPMMRRGPDGPRTLCNACGLMWANKGLLRDLSKSPVPLHSIQ
QSAPILNGGNGSAMSALGSELENAAAAMGNGHEP

ZmZML4

AGPv4:
Zm00001d033523
Chr1: 265546924..265551800

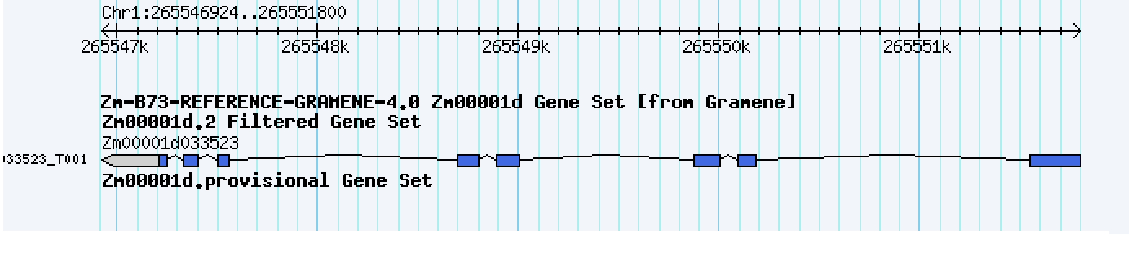

>Zm00001d033523 Genomic DNA
ATGCCCGACGCGGATGTTGACACCGTGATTCGCGAAGCCGCCGCGGCCGCGGCCCCAGCCGGCGACGACGACAGCGAGGG

TGACACCGAGGAGGAGGAGGAGGAGGGGGACGAGGTCGACGAGGACGAGGACGACGACGAGGAGTTACCCGCCGAGGCGC
CGGCGCCGGACGAGGAGCAACCTGCTCCAGCGCCCATCTCGACCTTGCCGGGGAATCCAAACCAGCTGACGCTGGTCTTC
CAGGGCGAGGTTTACGTTTTCGAATCCGTCACCCCCGAGAAGGTCCCTCTCCACGATTCATTTCGTTCTGTTTTGCAGTT
CACGTGGAAATGTTACTCCAAAATTATTGCGATCGAATATGAAATTTGACGTGTTTAGTGGTACATACTTTACTAGTCAG
AGATAAATGGCAAAATAGCAATTTCTTCGGAGAGATGCTTGATTTCCCTGTTTCTGTTTTTTTCGGCGGGGGGGTTGGGG
GGTGCAAATTTGATTTTCAAGGTTTCTCAACTGTTCCCACAGTTCGTGCTGGTCGTAATCTTTTCCCACTTGACTGATTT
TGTGTTGGGGTTGGGTTGGTTAATCAATTTTAGTTCGCCAAAACATAGTCATTATCAAGTACATAAAACTCTGCCGGTAG
GGGAAGACCACCCCACCGTATTATATTAAGAAGAAGTTCAATTGGAGCCTCGACCGAGAAAGGTCACGAAACCTGGTCCC
CGTGCAACATGAGGGTCCGTCACCTATAGGCCAGATCTTTGCTCGTGCTTTGAGCCCTCTCAACCCCTACACGAGGATCC
GCCCTCCATAGGTCAGTCAATCTCGTGTGCACACGACCAGGGAACCTGTGAGTGACCTTTTTAAATCTCAGCTTGAAATT
CGCTCTCACTGAGATTCAAACTCAGGACCTAAGGAGTGCTACTCATACCATCTAACCAACTCAGCTAGAGGCCCTTTCGC
CATTATCAAGTACATAAATGAGTTATGTCGTGGCAATGCCGCAATGGTTTAGGCTTAATGGCTTTTATGAATTTTTTTTC
TTTGTGTTAACTTGAATAGCAATAATGTTATGCATACTAGGCTGGTAGGTTATACCGCTTACCACTGTACTGTGCAGCTG
TGCTAATCTGTGTACTGTGTACAAGCCCTATGTTTCTGAATCAAGGTAACTTCCAACAAACTAGTAGCCACCACTGAGAT
TATGTGATTCAGTAATTGGTTGCTAGTCTGTTATGCAGCCTCACTGTATAATAGTTTTGGTTTACTACAATAAAAAGGAT
ACAGTCCTTAGCCACTATGTCTTAAGGCAGTTTTCAACCCTGTCATACCATGCTTTTTTCCTGTAAGAAGCATATTAACC
AAACATCATCTTTGTATGTACTATGTACTACTTACTGCCATCATTCATGTTTTACCATAGTACTCGAGCATTCATCTGGA
CATCTGGTTATACATCTAGGTATAGGTAATTCTGGATCTATTCAGGTGGTGTTTTGCCAGCGCTGCTGCTGCATATTTCA
CTGTTTAAACAGTAGCTTATGTGCGCCTGCCAATAATGGCTTCTGAACGGACCCCTTGGTCCACGGCTTTTTTTTTCTGA
ACTGAAGTTTATTTTCCAGGTTCAAGCTGTGCTGCTACTTTTAGGACGTGGTGAACTACCACCTGGTTTAGCTGGCATGG
TTTTACCCAATCAAAATGAAAACAAGGTATGCACAAATTGATCTGCAGCTTATATGACAATAATTCTATCACTGAGTTAT
GTGTAATAGTACCCTATTACCATTCTTCTGTAGGGCTATGACGATATACTTCAGAGGACAGACATTCCAGCAAAAAGGGT
TGCTTCACTAATTAGGTTCCGTGAGAAAAGGAAGGAAAGAAATTTTGATAAGAAAATACGTTATGCTGTTCGCAAAGAAG
TGGCACTCAGGTTTGTAGCTCGTCTTGATTCTTGAGCAATCATCAACTTTATTTCTATTTCTATTGAAAGTGGTGTGGT
GCACTAGTATATATCTACAATCATCAGTGGACCTTGATTGTATCTAATGGTGAAATTACTTTTGTGCCTATTGGAGCATA
ATGTATTTTTGATGAGTAGTTTCAGCTATCAGAACTCAGAAGGGCAGGTTTGGTGCATTGGTTAGAGTTGTTTCACTAAG
TCACCAGGTCGCGAGTTCGAAGCAGCCTCTCTGCGTTTGCAGGGGAAGGCTTGCCTCGGTTTATGCCTTCTCTAGACCCC
ACTCATGTGGGAGCCTCAACCACTGGGTCTGCGCTGCCCTAGTATAGCTAGCAGCATGTCATGCTTCTGCTGCTTAATTG
AATAAATGGTCATTATTGGAGCATAGCAATTGGTTTCATCAGATATGGAACAAAATTCTGGTCATCCTTTTTTAAAAATA
ACTGTCATAACCAAGAAATGTGCCATTATTTTTTTTTCAGTCACTGAAAGTTCAGCTTGACCAGCTTATATCTGAGCCAA
GGAACCTGAATTTGTCCCAGCTACTGACTTTACTTGCTTGGTCAATGTCTTTGTTTCGTACATATAGTCAGTTAAGGAAA
CCTGAAATGGTTTTTTCATGACGATAGCAATAATGTGATTGTATAGGATCATAGGATAGCCTGGGGCCTAGGATGCAGAG
GGAGACTGCCTCTGAACTGCTTGAAACATAGGAACATAACATTGTTCTACGTCAAAACATGAAACCAACTAGCAACTGCA
GCACACAGCTAGCAGGTATAGTGCATGTCTTAATAACTCATGGTCCTTTTTTGTCCTTTTTTTGTTACTCTCAGGATGCA
GCGTCGGAAGGGGCAATTTGCTGGAAGAGCCAGTCTGGAGGGAGAATCTCCAGCTCCTGGATGTGATCCTGGTTCTCAGG
GCTCAGGCCTAGATTTTGCATCTCGAGAATCAATGTATGTCATGTGATATGTTTTTTTCTTCTGTTCATTACTTAATGGA
AAACTGACCCCTTTCCCTCCATGTCATACTCTGCAGGTGCCAGAACTGTGGTACTAGTGAAAAAATGACCCCAGCAATGC
GTCGTGGTCCAGCGGGTCCAAGGACTTTGTGCAATGCTTGTGGATTGATGTGGGCAAACAAGGTACAATTACATGCTTAA
ACATGCTGGATTCTTTCTTTTTGGTGGTTGACATGATTATAGGTTCATAATTTCATGTCATATTCGTTTCGACTTATTAG
GCGATACTTTGTATGTCTTCTCTTGTACTTCTTCAGGTGTCGTGAATATGAAAAGAGCCTCTAGACCACATCTTTGACCA
ATAATTTTATTTTAAAACTATGTATGAAACGAAATAAATTATGGCATCACCAAAGCATCGCCCAAGATGAATTGGTTGAT
TTTGCTCCAAGGTGTCACTCATTAGATGAGTCTATTCTAGGTCAAACTTAAAATTGGTTTATTGTGCTCCAAGGTGTCAC
TCTTGTGAGTAGAGGGAGAACACGGTAATATTGCACCATGTTGGAGTTATTTTGTGTGGTTGAACTTGAAAAACAATTCT
TCAAAACATGGATAACCCATACTTTATTGCAATGGTGTGACATGAGTACATGACATGACATGACATGCTAAGGCAGAAAA

TTATGTTGCAGCAGTGGTATATGTGTGGTTGCAGCTTGGTAGGTAAAATTTTATGTCATGTGTCAGCCGAATCATGAAGA
TATGGCTATGTTTAGTCATGTACTCCTATTGCTCTTTTAATTTGTTATAAAAATATGAATTTTCAGTTGCAGCACCTTGT
CCTTTTCTTTTAAGAAAAAAAAAAATGTCGCCCCATGGAGTTCTGCTAAATGTTCTTCACAGGCGAGATGTCAGAATATTA
AGTATGTTGTCCTCTGGAGATGAACAGAAGGAATGGTCTTATATAAAACTTGAAAGGTGAAGGGAGCCAATTGTTTCTGT
TGTGCTGTTAGTAAACTGCTCCTTTGGCAATTATTTCTGTCGTACAAAGACTGGAAGAAAGCAACTTGTTATCTGTGCTA
TGAACATACGCCCACCGTCCCAAATTAGTATTCTTTTTAACTCTTGATTTTTATATCTATATTCAAATGGATGATGATGA
ATCTAGACACATATATGAAACACATACATCAAGTATTGTATGAATCCATTAAAATTCTAAAACGAATTTTAATTTGGGAC
AGAGGTAATATCTCGCATATCCAGACTTGACCCCCATCCCTATTAGACTTCTATAGTTACTACTTCATGTCTGGTGGCTG
CAGGGTACCCTGAGAAGTTGCCCCAGGGCAAAAGTTGAATCTCCTGCGGTTGCAACTGAGCAGGTTCACTTTTTTCTTAA
TTACTGTATCAGCATGACTTTCTGCAATTTCTGACCTTTGAATATTTTTAAAACCCCAAAGCAATGCTGTCTTTTCTCAG
GGAACAAGTGACAACAAAGCCCTGGCAACACTGAATAATAACAATGTTGCTGCAAGCAATGGCGAAGCGTAAGTTTTGAA
ATCGTTTGCCCTTTTCCAGTGGTAGCTATTCAGGCAGGGAGCTCATGAACTGCAACTGTGTGTTATCTTATCCAGCACAA
GTGCAGCAGAGAAAGGAGTGCCAAAGGCGCCGTGACAGTATATAGACATTATTGTCAGAGACAAAGATTTCGGATTCTTT
GCCTATAGCTAGACAAGACACCCCGTTAGAAATATTTGGTAATGATAATGATTGTCATCAAGTGTACATTCATTCTTGCT
TGACAGTGTTAGACGTGTAATTAATATAAGCTGTCCGATTGTGTAAACAGTAAACCCATATGGCATCTGTTGTGTTACAA
CTTACACTTTCTAACTTTCTTCATTCTCATAACATCATGATCAAAGATTCGATGCTACCCATTGATTGTCACATGGC

>Zm00001d033523_T001 cDNA

ATGCCCGACGCGGATGTTGACACCGTGATTCGCGAAGCCGCCGCGGCCGCGGCCCCAGCCGGCGACGACGACAGCGAGGG
TGACACCGAGGAGGAGGAGGAGGAGGGGGACGAGGTCGACGAGGACGAGGACGACGACGAGGAGTTACCCGCCGAGGCGC
CGGCGCCGGACGAGGAGCAACCTGCTCCAGCGCCCATCTCGACCTTGCCGGGGAATCCAAACCAGCTGACGCTGGTCTTC
CAGGGCGAGGTTCAAGCTGTGCTGCTACTTTTAGGACGTGGTGAACTACCACCTGGTTTAGCTGGCATGGTTTTACCCAA
TCAAAATGAAAACAAGGGCTATGACGATATACTTCAGAGGACAGACATTCCAGCAAAAAGGGTTGCTTCACTAATTAGGT
TCCGTGAGAAAAGGAAGGAAAGAAATTTTGATAAGAAAATACGTTATGCTGTTCGCAAAGAAGTGGCACTCAGGATGCAG
CGTCGGAAGGGGCAATTTGCTGGAAGAGCCAGTCTGGAGGGAGAATCTCCAGCTCCTGGATGTGATCCTGGTTCTCAGGG
CTCAGGCCTAGATTTTGCATCTCGAGAATCAATGTGCCAGAACTGTGGTACTAGTGAAAAAATGACCCCAGCAATGCGTC
GTGGTCCAGCGGGTCCAAGGACTTTGTGCAATGCTTGTGGATTGATGTGGGCAAACAAGGGTACCCTGAGAAGTTGCCCC
AGGGCAAAAGTTGAATCTCCTGCGGTTGCAACTGAGCAGGGAACAAGTGACAACAAAGCCCTGGCAACACTGAATAATAA
CAATGTTGCTGCAAGCAATGGCGAAGCCACAAGTGCAGCAGAGAAAGGAGTGCCAAAGGCGCCGTGACAGTATATAGACA
TTATTGTCAGAGACAAAGATTTCGGATTCTTTGCCTATAGCTAGACAAGACACCCCGTTAGAAATATTTGGTAATGATAA
TGATTGTCATCAAGTGTACATTCATTCTTGCTTGACAGTGTTAGACGTGTAATTAATATAAGCTGTCCGATTGTGTAAAC
AGTAAACCCATATGGCATCTGTTGTGTTACAACTTACACTTTCTAACTTTCTTCATTCTCATAACATCATGATCAAAGAT
TCGATGCTACCCATTGATTGTCACATGGC

>Zm00001d033523_P001 peptide

MPDADVDTVIREAAAAAAPAGDDDSEGDTEEEEEEGDEVDEDEDDDEELPAEAPAPDEEQPAPAPISTLPGNPNQLTLVF
QGEVQAVLLLLGRGELPPGLAGMVLPNQNENKGYDDILQRTDIPAKRVASLIRFREKRKERNFDKKIRYAVRKEVALRMQ
RRKGQFAGRASLEGESPAPGCDPGSQGSGLDFASRESMCQNCGTSEKMTPAMRRGPAGPRTLCNACGLMWANKGTLRSCP
RAKVESPAVATEQGTSDNKALATLNNNNVAASNGEATSAAEKGVPKAP

ZmTIFY4（*TIFY44*）

B73 RefGen_v3:

GRMZM2G036349 (zim6 - ZIM-transcription factor 6)

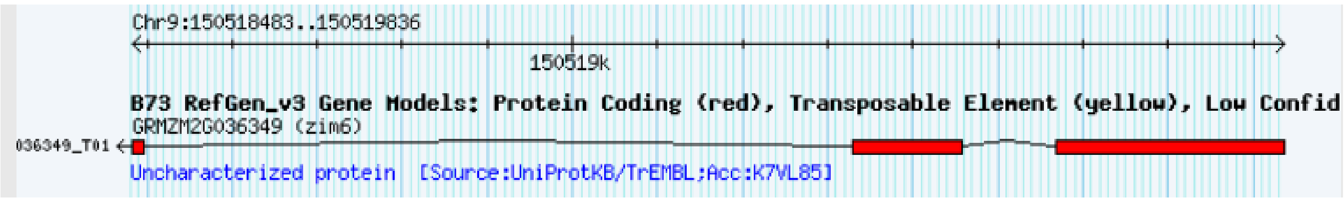

>GRMZM2G036349 Genomic DNA
ATGTGGTTCTTAATCGGGCCGTGTCTTAATAGGCTCAGGCCCGCTTCAACATCTGTACAAGCTAGCCACCCTGGCCCGTC
ATGTCACCCGCTCGCCTTTCACGTGTTGCAATGCTTTCCCCTCACCACCGGGTGTCTCGCGCCGGCCGTAGCTATGGGCC
TCATGCCGGCTGCTGCTGCGGCGGCGGAGGCGACCGAGGAGAGGAGCACAGTGCTGGAGTTCTTCCCGCAGCAGGCTGGC
ACGCTGAAAGACGAGCAGCAAAATAAAGGTGTGTTCGTGCCTCGCCGCCTTCAGTGTGACCTTTACTCAGTCCTCATCCG
TGCGCACATATATATAGGAAGAGGAAGGACGGGAGAGCGGCGCTGACCATCTTCTACAGCCGGCGGCAAGGTGGTGGTCA
TGGACAACTTCCCCGCCGACAAGGCCGATGAGCTCATGCAGCTCACCGGCTCCGGCAACGCCGCGCTCGCCGCCGCCCAG
AGCGCGCTGGGACAGCCCAGCCTCAAAGGTAGGAACGAACTGGACGCGCGCGTATGCCTACGCTGTCCTCCATCAGAGTT
CACAGGGACGATAGATATATAAGATTATAAGGTGTTCATACGTACGGCGAGCTGAGAGTGACAGCATAGATGTGCATTAC
CGTTTCAAAGCTCGCTCCGACAGACCTTGGGGACACGGCGCGCGCGTTAGGTGGCGTCGTGGGCTCGTGCCACGTTTCGC
GTAAAAGTGAACTCAGCGCAGCGGTGGAACTAGAAGGCAGTAGAAGGAGAGAGAGAGAGAGAGAGAGAGGACGGGGAGCA
AGTTCTGCTGGCCTTCTCGCTCTTCTGCTTGTTCCTCGTCGTCTCCACTCCACGTCTGTCTAGCTGGCTGGCTGAATTTT
CATTCCACTTGTAGAGAATATCTTGAGACGATGTAGTAATAGAGAGATGTAGGTAGGAAATGGTCGGAGGAGTCTTAACT
CAGTAAGCTTTGTTCCTTTATAATGGATTGGTAGGTCGGAACGATTTTTAACCAAATTGCTTCTTTAATTTATATAAATT
TTAATTAGCTGAAACGATTTCGGGTGTAATCTGACACAAATGAACAAGGCCTAAGCAAAATAGAACCATTCAGATTACCC
CTCGATCTAGTATGTACTATTAGTTATAGTTTTGAAAAAGATTTAAGTTAAATTTATAAAATTTTGACTACAAATAACTG
TTTTATTATTTAATATTAAAACCTAATGTTTATATATATATATAGTGATTTGTCTTAAAAAGTGTTTTTTATAAAAGAAT
ATAAATGTATTAAAAAAATATTGATCAAACACGCTAGTTCACACCCATGTGTCCGTGTAGGTCTCATTGGTTGA
>GRMZM2G036349_T01(cDNA)
ATGTGGTTCTTAATCGGGCCGTGTCTTAATAGGCTCAGGCCCGCTTCAACATCTGTACAAGCTAGCCACCCTGGCCCGTC
ATGTCACCCGCTCGCCTTTCACGTGTTGCAATGCTTTCCCCTCACCACCGGGTGTCTCGCGCCGGCCGTAGCTATGGGCC
TCATGCCGGCTGCTGCTGCGGCGGCGGAGGCGACCGAGGAGAGGAGCACAGTGCTGGAGTTCTTCCCGCAGCAGGCTGGC
ACGCTGAAAGACGAGCAGCAAAATAAAGCCGGCGGCAAGGTGGTGGTCATGGACAACTTCCCCGCCGACAAGGCCGATGA
GCTCATGCAGCTCACCGGCTCCGGCAACGCCGCGCTCGCCGCCGCCCAGAGCGCGCTGGGACAGCCCAGCCTCAAAGGTC
TCATTGGTTGA
>GRMZM2G036349_P01
MWFLIGPCLNRLRPASTSVQASHPGPSCHPLAFHVLQCFPLTTGCLAPAVAM**GLMPAAAAAAE**ATEERSTVLEFFPQQAG
TLKDEQQNKAGGKVVVMDNFPADKADELMQLTGSGNAALAAAQSALGQPSLKGLIG

AGPv4:NO
Zm00001d048264, this gene model does not exist in MaizeGDB and GRMZM2G0363 has not contained the TIFY and Jas motifs.

ZmTIFY5（*TIFY45*）

Zm00001d049005, this gene model does not exist anymore in MaizeGDB. GRMZM2G122160 has TIFY motif but no Jas.
B73 RefGen_v3:
GRMZM2G122160 (zim11 - ZIM-transcription factor 11)

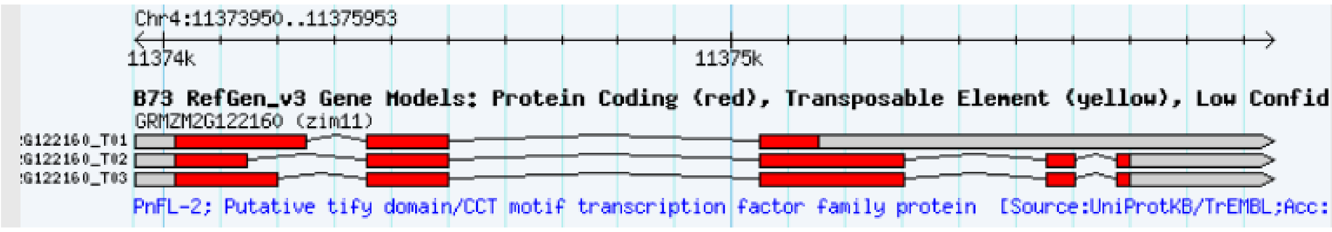

>GRMZM2G122160 genomic DNA
ACTACCCTGCTCCCTCTCCCAAGCCACCGCCACTCCTTCTCCCTCTTGCGCAACCAGAGCGCACAAACCCAATGCGGCGC
CGGCCCCTCCTCCCTCTTGCGCAATTTGGCTGTGTGGCCACGGACTATCAAGAACAGGCAGTGGATCCTGACCCCTTTTT
CTCTCATTCCAGCAGCAGCGGTGGAGAAGATCCTGCAGGTAGTGCAGCAGTGAGGCGGTGGATCTCCAGACAGTGCAGCA
GTTGGGTGGCAGGTGGAGCAGCCCCAGCCAGTTTCGTTTCTAGTGGGGAGCGTGTTGTGCTGGTGTGAGATCTGGGCGGG
GCCTTTGTTATCCGGTTTGAAACCACGTTTTCGGGCTTCGAATTTTGCGGTCCCTTATCCGGATGTTTTTTTGGGTTCC
CGTTCTCAGGCGAGGTCGAGCAGCAGAGGACGCCGGCGACAACTAACTTGCTCTCCGGAGCGGAGGGCGAGGAGGTCGAA
AGGAGGAAGGAGACCATGGAGCTGTTCCCGCAGAGCGTCGGGTTCAGCATCAAGGATGCTGCTGCCCCTAGGTGCGTACC
ACTGCTGCTTCACATCATCTAAATCCATTTCGATTTTGGTTTGCGCAACAGTTGTTACGTAGTAGCACAGCAGAACTCAA
GCCTAGCTAGCTGTCTTATGTCTCCTGGACAAAAGGCCAAGCAATTTGACTTTCTAACATAGAGATCTGAGTTTAATGAG
TGTTTATAGGTGGAAGTGGAACATGTGGTATAAAATCTAGTTGAGCCGGATGGCAAATGACAGAGCATGTTATTACGTAT
GTGTAAAGCTAAAGCATCCCTTGCTGTTCAGTCTGTGTTGGTATTCCTTGGTCAAAGCTGTATCTTTGTAATCTTAAGAA
GTTACCGCCTTTGCTAAAACACCGTGTTCTGCAAGGTAACACTGTTTCCTTTTATCAGGCAAAGCAACCAACATGCTCAG
AGATTGTACCTAATGTTTATCATTAACTGCCAAGGATTATTAACAGGAAAAAAAAACAGAGTGGATAATTGATTGGCTAA
GCTGAAGTAGCCTACTACATGCACTGTTCCTTAGTGTTCTTCTCTGTAATATTTGTCAGGGAGGAGCAAGGAGATAAAGA
GAAGCCTAAGCAGCTCACAATCTTCTATGGCGGGAAGGTGCTGGTATTTGACGATTTCCCCGCCGACAAGGCAAAGGATC
TGATGCAGCTGGCCAGCAAGGGCAGCCCAGTGGTACAGAACGTTGTTTTGCCTCAACCCTCTGCAGCTGCTGCTGTCAGT
ACTGACAAGGCCGTGCTGGACCCGGTCATCAGCTTGGCCGCTGCTAAGAAGCCTGCTCGCACAAATGCTTCTGGTATAAA
CACTCTCCCTCTGGCCAGCTGGGGTATTTATTGAGTTAGCATCTCTAAAATATTACATTTAAGTTAGCATCTCTAAAATA
TTACATGATATTTATTGAGTTTTTTATATGTCTTATAAGTTTTCCCAATTATTTCTAAACCAACCTATAGCTTATGTTTT
GATCTACATGCTCACTAAATATATTTGATATCTTTTCAGCGACAAGAAAATTATAACCAAGTGCAATTGTTTTTTTGAAG
CAGATGCATTACATCAAGAAAAAGATTTAGTGAAACTGTTTAGAAAGCTATTGTAAGTACATATTTACCTTTTTCCCTTC
ATTTTCGACTTATGTTACGCTAGACTAAGTTTTAATCTTGATGCTAGGATGATATTGATGCTCGGATGATATGGAAGCAA
CGATAGCAGTACCAGTTGAAGATGGACAGTTTGCGAAGTCTTCTACATAAATTTCACATGTCTTGCTTTTGTGACTGATC
AGCTCTGGAGTAGCTAGAGTGATGTAGTTGTGGGCTTTTGAACAATAGCTGGAGTGATATGAAACATTAGTGATGTAGTT
GTGGGCTTTATAATTGTGAATGATATTAGTAAATGATCTAGTTGTGAATGATATTATAATTGTGATGCTTTTGTGAATAT
ATAA
>GRMZM2G122160_T01 (cDNA)
ACTACCCTGCTCCCTCTCCCAAGCCACCGCCACTCCTTCTCCCTCTTGCGCAACCAGAGCGCACAAACCCAATGCGGCGC
CGGCCCCTCCTCCCTCTTGCGCAATTTGGCTGTGTGGCCACGGACTATCAAGAACAGGCAGTGGATCCTGACCCCTTTTT
CTCTCATTCCAGCAGCAGCGGTGGAGAAGATCCTGCAGGTAGTGCAGCAGTGAGGCGGTGGATCTCCAGACAGTGCAGCA
GTTGGGTGGCAGGTGGAGCAGCCCCAGCCAGTTTCGTTTCTAGTGGGGAGCGTGTTGTGCTGGCGAGGTCGAGCAGCAGA
GGACGCCGGCGACAACTAACTTGCTCTCCGGAGCGGAGGGCGAGGAGGTCGAAAGGAGGAAGGAGACCATGGAGCTGTTC
CCGCAGAGCGTCGGGTTCAGCATCAAGGATGCTGCTGCCCCTAGGGAGGAGCAAGGAGATAAAGAGAAGCCTAAGCAGCT
CACAATCTTCTATGGCGGGAAGGTGCTGGTATTTGACGATTTCCCCGCCGACAAGGCAAAGGATCTGATGCAGCTGGCCA
GCAAGGGCAGCCCAGTGGTACAGAACGTTGTTTTGCCTCAACCCTCTGCAGCTGCTGCTGTCAGTACTGACAAGGCCGTG
CTGGACCCGGTCATCAGCTTGGCCGCTGCTAAGAAGCCTGCTCGCACAAATGCTTCTGATGCATTACATCAAGAAAAAGA
TTTAGTGAAACTGTTTAGAAAGCTATTGATGATATTGATGCTCGGATGATATGGAAGCAACGATAGCAGTACCAGTTGAA

GATGGACAGTTTGCGAAGTCTTCTACATAAATTTCACATGTCTTGCTTTTGTGACTGATCAGCTCTGGAGTAGCTAGAGT
GATGTAGTTGTGGGCTTTTGAACAATAGCTGGAGTGATATGAAACATTAGTGATGTAGTTGTGGGCTTTATAATTGTGAA
TGATATTAGTAAATGATCTAGTTGTGAATGATATTATAATTGTGATGCTTTTGTGAATATATAA

>GRMZM2G122160_P01

MRRRPLLPLAQFGCVATDYQEQAVDPDPFFSHSSSSGGEDPAGSAAVRRWISRQCSSWVAGGAAPASFVSSGERVVLARS
SSRGRRRQLTCSPERRARRSKGGRRPWSCSRRASGSASRMLLPLGRSKEIKRSLSSSQSSMAGRCWYLTISPPTRQRI

>GRMZM2G122160_T02 (cDNA)

ACTACCCTGCTCCCTCTCCCAAGCCACCGCCACTCCTTCTCCCTCTTGCGCAACCAGAGCGCACAAACCCAATGCGGCGC
CGGCCCCTCCTCCCTCTTGCGCAATTTGGCTGTGTGGCCACGGACTATCAAGAACAGGCAGTGGATCCTGACCCCTTTTT
CTCTCATTCCAGCAGCAGCGGTGGAGAAGATCCTGCAGGCGAGGTCGAGCAGCAGAGGACGCCGGCGACAACTAACTTGC
TCTCCGGAGCGGAGGGCGAGGAGGTCGAAAGGAGGAAGGAGACCATGGAGCTGTTCCCGCAGAGCGTCGGGTTCAGCATC
AAGGATGCTGCTGCCCCTAGGGAGGAGCAAGGAGATAAAGAGAAGCCTAAGCAGCTCACAATCTTCTATGGCGGGAAGGT
GCTGGTATTTGACGATTTCCCCGCCGACAAGGCAAAGGATCTGATGCAGCTGGCCAGCAAGGGCAGCCCAGTGGTACAGA
ACGTTGTTTTGCCTCAACCCTCTGCAGCTGCTGCTGTCAGTACTGACAAGGCCGTGCTGGACCCGGTCATCAGCTTGGCC
GCTGCTAAGAAGCCTGCTCGCACAAATGCTTCTGATGCATTACATCAAGAAAAAGATTTAGTGAAACTGTTTAGAAAGCT
ATTGATGATATTGATGCTCGGATGATATGGAAGCAACGATAGCAGTACCAGTTGAAGATGGACAGTTTGCGAAGTCTTCT
ACATAAATTTCACATGTCTTGCTTTTGTGACTGATCAGCTCTGGAGTAGCTAGAGTGATGTAGTTGTGGGCTTTTGAACA
ATAGCTGGAGTGATATGAAACATTAGTGATGTAGTTGTGGGCTTTATAATTGTGAATGATATTAGTAAATGATCTAGTTG
TGAATGATATTATAATTGTGATGCTTTTGTGAATATATAA

>GRMZM2G122160_P02

MRRRPLLPLAQFGCVATDYQEQAVDPDPFFSHSSSSGGEDPAGEVEQQRTPATTNLLSGAEGEEVERRKETMELFPQSVG
FSIKDAAAPREEQGDKEKPKQLTIFYGGKVLVFDDFPADKAKDLMQLASKGSPVVQNVVLPQPSAAAAVSTDKAVLDPVI
SLAAAKKPARTNASDALHQEKDLVKLFRKLLMILMLG

>GRMZM2G122160_T03 (cDNA)

ACTACCCTGCTCCCTCTCCCAAGCCACCGCCACTCCTTCTCCCTCTTGCGCAACCAGAGCGCACAAACCCAATGCGGCGC
CGGCCCCTCCTCCCTCTTGCGCAATTTGGCTGTGTGGCCACGGACTATCAAGAACAGGCAGTGGATCCTGACCCCTTTTT
CTCTCATTCCAGCAGCAGCGGTGGAGAAGATCCTGCAGGTAGTGCAGCAGTGAGGCGGTGGATCTCCAGACAGTGCAGCA
GTTGGGTGGCAGGCGAGGTCGAGCAGCAGAGGACGCCGGCGACAACTAACTTGCTCTCCGGAGCGGAGGGCGAGGAGGTC
GAAAGGAGGAAGGAGACCATGGAGCTGTTCCCGCAGAGCGTCGGGTTCAGCATCAAGGATGCTGCTGCCCCTAGGGAGGA
GCAAGGAGATAAAGAGAAGCCTAAGCAGCTCACAATCTTCTATGGCGGGAAGGTGCTGGTATTTGACGATTTCCCCGCCG
ACAAGGCAAAGGATCTGATGCAGCTGGCCAGCAAGGGCAGCCCAGTGGTACAGAACGTTGTTTTGCCTCAACCCTCTGCA
GCTGCTGCTGTCAGTACTGACAAGGCCGTGCTGGACCCGGTCATCAGCTTGGCCGCTGCTAAGAAGCCTGCTCGCACAAA
TGCTTCTGATGCATTACATCAAGAAAAAGATTTAGTGAAACTGTTTAGAAAGCTATTGATGATATTGATGCTCGGATGAT
ATGGAAGCAACGATAGCAGTACCAGTTGAAGATGGACAGTTTGCGAAGTCTTCTACATAAATTTCACATGTCTTGCTTTT
GTGACTGATCAGCTCTGGAGTAGCTAGAGTGATGTAGTTGTGGGCTTTTGAACAATAGCTGGAGTGATATGAAACATTAG
TGATGTAGTTGTGGGCTTTATAATTGTGAATGATATTAGTAAATGATCTAGTTGTGAATGATATTATAATTGTGATGCTT
TTGTGAATATATAA

>GRMZM2G122160_P03

MRRRPLLPLAQFGCVATDYQEQAVDPDPFFSHSSSSGGEDPAGSAAVRRWISRQCSSWVAGEVEQQRTPATTNLLSGAEG
EEVERRKETMELFPQSVGFSIKDAAAPREEQGDKEKPKQLTIFYGGKVLVFDDFPADKAKDLMQLASKGSPVVQNVVLPQ
PSAAAAVSTDKAVLDPVISLAAAKKPARTNASDALHQEKDLVKLFRKLLMILMLG

AGPv4:NO

ZmJAZ37（TIFY46）

This region of Chr3 is too complicated to make gene models. The models in v3, v4 and v5 are different. The gene model of **GRMZM2G327263** is a very big gene model. The part of this model (showing in red box) is containing TIFY and Jas domain, suggesting it is a zim gene. The gene model **Zm00001d044707 (v4) located outside "TIFY plus Jas region"** of **GRMZM2G327263 (**the red box) has no TIFY and Jas motifs. Other gene models (in red box) for this region are already included in Table 1 and Table S1. In conclusion, this region in red box has no gene model v4 and must be represented by **GRMZM2G327263** (v3) or **Zm00001e020904** (v5).

B73 RefGen_v3:

GRMZM2G327263 (zim17 - ZIM-transcription factor 17)

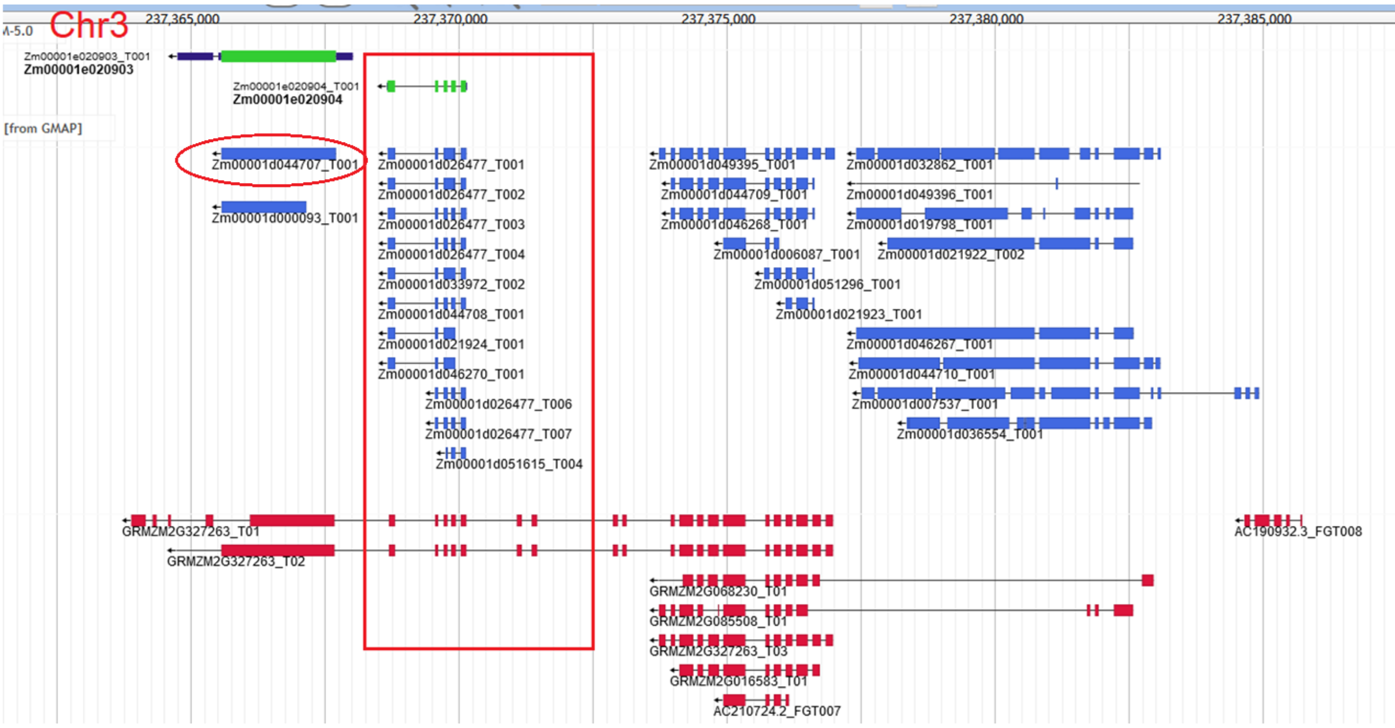

>GRMZM2G327263 Genomic DNA

ATGGAGTCTAAAGAGGAAAATGTTGCACTTACGGTCATCCAAAGAGATAATACATTATCTATAGATGAATCTCTACTAAT
GTCCTTGTCTCATAACAAGCCAAAAACATCGGTGACTACTACAATAGTTCCAAAAGGTATGATAACTTCCAGACTACCGT
ATGGATCAATTTGTATTGTTTTTATTGCAAGTGATTTATGTTTGATCTCATGACTGTTTTGTTCGTAGATTATATTTGCA
CGCAGGGTGATCTTGCACTCATCGATTGGATTAAAGAAATCCCTTGTGAACCAAGGGTAGAAGTCGTGCTTATTGATGAT
GCCTTTGTGGAAAGGAAGTGGATGGAATGCCTATTTGAACCGGACGCATATCTAGGTGATGAGGTAATTCATGATAACTC
AACTAGCTAGGAGCACCACACTTCCAAGATACTCAACCTGGTATTTTAAACCATAACTTACATACTTGCAGGTGATAGAC
TGTTACATAAATTTAATAAAAGCTCAAGAGCATCTAAAATGCCGATCTGGAGGTCGTGTACACATAGAAAATGCTTTTCA
GTTCAACTTTCTAAAGCGAGACGGTGATGTTGACACTAAAACAGATGAGTTATATCCAAGTAAAGACATGGCACAAATAT
CTAGCGCCGAAAGAAGGGTTTACTTTATCTAGACCATGACATGGTAAATACGAGATTGCAATCTACATGATTAGTGTTT
ACATGTATTATTATGCAATATTTATAATTGACTTGTAGGTGTTTATTCCAATAAATATCCGAGAAATGCACTGGTATCTT
GCCGTGATCAATGCACGAAATATGGAGATACAAGTGCTCGACTCACTTGGTACATCATCCGGTCGCAATGACCTCATTGA
CACTGTGAGTTTATACAAACTCAACAATTACTAATCCCTTATTAACTTAAAATCTCAACTTCTAAATCTATGTTTTTATA
ATAATATAAAGATAAAAGGACTCCAAAGACAAATTGATATGGTATCTCAGCGTAAGGAGTTAAAAGATCACAGGTGGCCA
GACCTCCGAGTTGCTTCTTGGCCACTTAGAGAAATAGAAATGGAATATGCAAAGCAAACAGATAGGTACACCATACATTC
ATATGTGTTGCTATTAATATTCATTGTTGTTAATCTTGACATTTTATCATCTGGTTGATGTAGCTCCTCGTGTGGCCTCT

TTCTACTAAACTATATCGAATACTGGACAGGGGATGAACTATCTGATAATTTTACCCAGGTATATTATTACTACTATGAC
ATTATGCGTGCATCATGGACGTCGTGAATTGTGGTAGAGCTTTCTTTCAGGCTGCAGACTTGTTTGCATCCCTACTGTTC
CAGGTCGTGCTCGCCTTTGTTGTTTGTCGTACGACCGACGATTTTTTATGATTGGTTCAGTCGTTTTGCTTTGCATACAC
TGTGAACATAACTGCAACCTGCATATTTATCACATGCACAGATTTTCTTGTTGGCAGTTTGCACTTTCTGATGGGTCTCT
TTTTTAACAATAAATTACATGTGGCTATAACATGAATATCTGTTTGCTGTCTTCTGTTATTATGCCAAACCAATTGATAC
ATATAATTGCTAAATAAATTGTTTCTTATTTGCAGGATGACATGTCACACTTTAGGAAAAAATTGGCTGCTATATTACTA
TCTTCAGATATAAACAAGAGAAAGGGGTGTCCGTTATACAAGTATGACAAAGAAGTCGATGCTGGATGCTCCTCTGATGT
TCAGATATTAGATAGTCCCACAAATCCTAAGAAGAGGAAACTACTTTGTGTATCTGAAAAGAGCGAAGTATTGATGGAAG
ATGATGATGGCCCCATCACTCAAGCAGACTTAGAAAAGTGGTTTGTTCATGATTGGGATAAAAGAACTCCTATAAAAATC
TCGACCGATGAGTGCACTAATGAATTTTTACTGAGTGGCCTTTCCACAAAGGACATGCCAGTGACCAAAGCCGATTTAAT
AGATGTTCTATGTGATTACATCATGACAATCCAAGATGATACGGCATTAGAGTAAGTGCTCATTGTTTTACCTATTTTAT
ATGTACCAACTACATAGAGTTTTGATATATACTTTTGTCATGTTCCATTACAGGATGACATGGGTGCGAAGTTTCAACCC
TTTTAAGATAGAAATATCTGTCAAAGACCTGCAAAACGTATTAAGGGTAAATCTAGATATGACTCTAAAATGTTTTGACA
TGGCTGTTCGATTGTTGGCCATTAAAGAGTCACATATGTCGAAAGATGAAATGATAAAGGATAAAAAACATTATATGGAC
ACGCGCTTCTGGGTAAGTTTGTTTCCAATCAATAATGTAGTAATATATTTTATCTATACATACAATCCTCATGTGGACAT
GTATATATATTTATCTAGAGAATGGTTGGTTTTGGTAAACTTCCAAAGTACCATCAGGATCCTACAGCAGAAGAGTTAGC
TAACACACTCGACTGTTGGCCTTCATTGAACTATTATATCACTGGTTGTAAATATGTAAGTATTTGTTCATTTCTAATGT
ATTTATAAAAGACACTTCGTTGCAATCCTAATGACTGATATTTTGTAGGTTCTTATGCCATGGAAATTCAACGGATGCTA
TGCACTTTTCGTAATCGATCATGGTAAAAAGCACGTAACTTTTATAGACTTTACACCCACTCAAGATTGGTGTAAACACA
TGCCATACAAGAGGTTTGCGGAAGCAATTATTATGGCCTCCAAGAAATATAAGATTGCCTACAATAAGAAACGTTCTGGA
TGGGCAGATGATATTTTTAAGTGGGAACATACAATTCGGTCTGGTCTTCCGATGGACTTAAAAGGGTATTTTCTCAATAC
CATGTTCTCGACAGTTTAAATTATGTCTATGATAACCATTGTATATAATATAGTTGGCCAAACATTATTTTGTAGGGTTA
ATACAAGCTACTTCGTTCTACAAGCTATGGTTATGTGGGGGAGTGGTAGACGAATGGAATTTAATAGGGTGAGTGGAGTA
CGTTTGGTTCATGTTCAAACATACATTTCAGTTACATAATATAAATTTGACGTTTTGTTCTCTTATGTCTTTGTGTGTAG
GATGCAAAAATTATTCGAAGGAACTTTGTAATTGATCTATTAAGTTATGAGGAGAATTCATGCCACTATGCTATCCCTCC
AAATATACAACAACGACTTATCAGTATCGCTAAAAAAGATTAGATTAATGTACGGTTGTCGACATATTTGACTATTATTA
AATATTGTAAGACATCATAATTTTAATTGAGGTTGCATCAATAAACAGGTTGATGTAGACCAATATGGTTTGTGTGTGGT
TTACATGTTTTAAATCTCTACTAACTATTAAGGGTGCAGTGTACTAAATCCATTTTACATCTTGTCATCTACACGCGAAA
TCCATTTTTTCTATTGATGCACCTAGATACCAAGCTCACCTTTTTTGGTTGGAGCTGGGAGTTTTAGATAGGAGGAGCAG
AGGGTGATCGAGGTGCAGATTATGATGTGGCAGCAGGGGGTATAGGACAATGTGGAGCAGATGAAGGACGATCAGACTGC
AACTGGCTCCATGCTGGCCTAATCCATTTGGTTGTGAACATGCTAAGTTTGCTGTTTATTGGAATCCGCCTTGAGCAGCA
ATTTGGATTTGGTAAGCTTTTAAAATCATTTTCATGGTACACCAATATCGATCCAACCATTCATTTCAGTTAAGTGTTGC
AACCATTCGTCCTTCATGGTCATTTCACTGACACTGTTTTTCCTGCTTGCAAGTTCGCATTGGTGCAATATACCTACTGT
CTGGTTTCAGTGGACTTCTCTGGATCTTGCTAAGATGTCTCGTGAAATTAGAGGCAGTCCAGAGATCTTGTTTGCAAGAG
AAGTTGCAAGGTAGTCATACCATCAAGCATTATTTTGTGCTTACCATATTTGTAATAGCTTTCATCTAATGCCTACATTT
CCTATACATTCAGAGCATGCAGAATGGGGAACTTTATTTCCTTCTTCCGTCTTGCAAGGAAAGCTACATATTTGGAGGCA
TGTTTGATGCATGCTCATTTTGCAAAGGTAGAATTATTGTCTCTAAAAGCTGTTCATCAACATACCCTTTCAGTGGAATT
TATTCTTCCTAAGCTCTTATATTTCCTAGGCCAACTGTGTTTGAAATACTGTAATGGCAACAACAAAATTAGAGCAGAAA
AATGTGAGGTAATTGATTTCCTTCGACACACACATAGTTCTCATTACACAAGTATCAGTTGTCTAACTTGGTTCGTTGGT
GTACCCTTTACCTAAAACATTGCTTGTTGATATTATCGGGATGGTCTCAATTTTACAAGAACTAAGCTGATTGTAGATGA
TAGTGCTAAATTTCTAGATGCAGTTTAACTCATTGATGGTAGTCCTGTTGAAAGTTTCCTTTCTTCTGTACATAGAGCCA
ATCTGGTTTGTGATTGAACTGGTGCACATGTTTACTTGCAACCTTGTTGGATTCTATATGACATTTCTTTTTTTACTTTG
CATTAGATAGCTTGAGATGGACAATCAATATCCTTGGTTCTCAGTTAAACTGCATCATGATAGTTTAATACATGAAAAAC
ATAATGTTTTCGTGAAGATATACTTGAAATCTTATAGTGCATTTGCTGTCAGTTAAGGAAGAATAATTTGGACTGCATAT

ATTTCACAAACATACTGAAGAAAACATTAGGAATCTCCTGTCATCATGTTTGTTTGTCATCATGTTTGTTTGTCTAGACT
TGGACTGGTGTTGAATATATAGATTATAGAGTGGAAATGATTCGTCGCCCCTGTATCTCAGTGACTTAGTGTCCCTTGTG
GCTGTAGATTATGAAAGAAATATGCTATGATCAATTTAAAGATAACGCTGTGGGATGATGTGACCTGTGATTACTTCCCT
CTCCCATTGTTGTGACCTGTGATTACCTACCTCTCAACTTTCAATGGCTCGCACGTGTTGATGTTGGCATTCTGGTTATC
TCACTGCCAACTGAAGTTGATTATTCATTTGCTAAATCACGGAGAAACAATATTATGGGGGGAAATAGACGGGAAGATGG
TGCATTCTGCCATATTTTACACTAACTACTAATGGTGACCTGATGTGTGCTTTAAGTATGTAAACATGATTGGTGCTCGA
TGAGCCTATGTACATCAGAGGAATTAAGCCGAGGAATTGAGCCACATATTTGAGGCATCCTTGTGCACCTAGAAATCGCT
TCCTGTTTTGCCCATCCTTCAGATAAAACGGGGTTCTAACTGCTTGTTCATGTAGCTGATCCTATATCCTAATAACTAGA
CTAAGCTGTCACTTATAACTTGTGTTATAGCTAATGCATTGTAGTTGTATGCCAATAGATCTTGCTTATTGATGTAACTT
TGTTTCTTTGGTTCTTCCTGTATTAGAAACAATACCCATTTGTGAACATAATCTCACATGCTGGTTATGACTGTTATAGG
AGTGGTTGGAAGCAAATAAAGACTACCGAGCTATAGTCGATGGTGCGAATATTGCACTATATCAACCAAATTTTGCGGAG
GGCGGTTTCAGTTTGACCCAGGTCTGTGCACCACTTTGCCCTGAATTTCAACATGTTGACATCGAGAATTAAGCAGTGCA
TGCTAATTATTGTTCAACTTTGCATGCTTAATATAGAGCAGCTGGATGGCGTTATAACAGAGCTTCGAGATAGATATCAT
GGAAAATGGCCACTTGTCATACTACACAATAAATGAATTTCCAAGCTTATGGAAAATCCATCTAATAGGCATTTGATTGA
GACCTGGAGAACAAATGGGGCTTTGTATACTTCACCAAGTGGGTCAAATGATGACTGGTAATGCTAAGTTTCTAGCCTTC
AGTATCTCTGAACTTGCTGTTTATGCTCAATTCTCAAAGACAACGGAAAGACCTTGTGGTTGCGGCCACCTTTTCTTGTA
AACCAGGAGGTGTTTCTTGTTTATGTTGTAATTCTGTAGAGATCACAGACATCTATTATCACATAGTTTGTTGATTATTC
AGTTTTCTTCATTGTTATGTTTCCTCCTGGATGTTTCTAATTATATGAGATGCAAACATCCAGGTATTGGCTATATGCAG
CGATCAAACTAAATTGTTTGCTTGTGACTAATGATGAAATGAGAGATCACATATTCGAGCTTCTAGGGCTGTCTTTTTTT
CAAAAGTGGAAGCAACGCCATCGGGTGAGCTCTACAAAACCGATCATATAATGCTATTTCTCTATGATTTTTTTATGAAT
TGGTTGAAATTCTGAATTCTTGCTGTTTCATCTTGCTGTGCATCGACGTGCTCATTCAGAATTGTGTTTAACTTGCTGTT
CCATCTTGCTGTTCCATTTCATCTTGCTGTTCCATTTCATCTTGTTGTTCCAGGCTCTGAACAATAGGGACATCTGAATT
AATCAGATTCCAGGAAAAATCGTTGTGACTAGATCCGATATCTGTATCACTAAGGCCTACAAATGATTTGAACGCCACTG
TCCAGAAAGCCAATCCGTTCACTGGCCAAGTAGACATACCTTTCTATCAGAATTTTACCTCCGTTTTTTAATCATTTTAG
GAAGTAGGACGATTGCTTTCTACCCCCGTGTCCGCGCACACATTCTATCCTATGCCGGTTTTGAACCCACCACATGGCTT
TCTGATATGCTTTTATCTGAACCAGTAGTGGGAGTGACATGGATCTGCCTACTTTCAATTCAGAGGTGCTGTTTTATCTG
CCTTTGTGTGTTCTTATTTTTTTGCATTCTTTACTACAAGGTCTGCAATGTTGCCGATGTCGAACCCTTCAGCTAATCCC
ACACAGCTTACAATTTTCTATGGTGGATCAGTATGTGTGTATGACTCGGTGCCACCAGAAAAGGTAAATATTATCTTCAC
GCCATTTACAGATATCTTGTCCTTCTGTACCATCTGTTAAAATACACATGCTAATACACTAATTTGGCATTGACAGGCTC
AGGCAATCATGCTTATAGCTGCAGCTGCGGCAGCTGCGGCAGCCACCAAAGGCAGTGCTGCCACTGCTTTTAATCCTCCA
ATGGTACATACAGCCACTGTCTCCCCAGCAGCAGTCTTCTCTCCTGTGCTTACACGGTCTCCATCACTGCAGAGCACTTC
TGTAGCAGCTGGACAAGCTCAGGTTGTTGCTGACCCTAGCTCAATAAGCAAGCTTCAGGCTGGTAAGACCACTGCATCTT
TGTGTAAAAATCCATAAATGGAATACCTTTTCCAGTGTTGACATTGTCCAACACGTCTTTAAACTATCTTGATCTGTTGG
CCAGATCTCCCCATTGCCAGGAGGCACTCTCTCCATCGCTTCCTTGAGAAACGTCGTGACAGGTTAGCATGATACATGAG
AGCAACAGGGCTCTGATATTCTGAATCTTATTGTTAGGCCTCTTATTCTACAATCTACATGTTTAAAACTTTTGTTATAA
ACTGCTGTATGTGGTACCACACTAATTTTTTCCCCGACTATTGGCACCACAGTAATCAACAGTATATTTTGTACCGTATT
CTGTTTTTTGTCCTCCAGGCAGGGTAAAAATAAAATAAAATTTGCTGTTATAGCATTTAAATGATTGCACTTTCTGCTGA
CAAGTGCTCAATGACATCCCTAAAAAAATCTATAATTTCTTCACAGAAAGATTGGTAGAGAGCCTGCTTCCATAATACAA
CATATAGCTGCAAATAGCCTTGGATCATGTAACTCCCTTGAGATTTTTTTTTAAAAAATCTGCAAATCGATATTAACACA
AGACAGAGTTCTGGGACCAGCAGTAGATACAAAAACCAACATTACCCCCATTTAGCTGCTCCAAATACCATGTTTTTCCC
CTGTTAGGAAAATAACATATTTGAGAATCGGGATGTTCCTGTGTATTATCCTGTTCTATTTGGAAAAGCTGGCCACTTAA
AGATCCAGTCTCAAGGTTGCAAACTAGCACCATTTTTTTTCTGATGTAAGCTTTAGATGCCTGTTTTTGCCTTATCTTAG
AACTGAGTTTAGAATTGTTTCTTAGTATAACTTTTTTTTATCGTGGATCTACTGATTTCAAGTGATTAAATAAATGATGG
CACGAAACAGGGTCGTGAGCAAAGCTCCGTACAGCCCCGCCAAGTCGTTCGATGGCATGGAGTCAGCGGGAATGGAGATG

ACTGTGGACGGCAAGCAGGGCGCAAGGCCCAGTATCTTGAAAGGTTGGGGAGCCGCGAAGTCTGAAGTCTGAACGGTGAA
AGCGTAGCCCTGCGGGAGCGTGGCGTTGTCTGGGAAGCGCCAGAAGCCGAGGTAGAGGGTGCAGAGGGAGGCAAGGTGGA
GGAATTGACCCGAACCTGGAATACCCGAACCCGAACCCGAAATACCCGAAACCCGAATTTTGTTCGGGAATTTCGGATAG
CAACTTGCAAAACCCGAAATTATTTCGGGTAATTCGGGTATCACAATCGGGAACCCGAATTACCCGAACTTTCATGTGTC
AAGTACTCATATGTCATGCTTAATTATTAATTTGTACATCTATAATTATGTGTAAGTGTGCAAAACTTATGATTGTAGAT
TGCTTGTGTTTTATATGTCCATGTATATCATTATTCAAACTATATTTTTATACTAATTTTATAGGGTGTATGTGTTTTTA
TGAAGCCAACACAATAGTTCGGGTAGTTCGGGAATACCCGAACCCGAACCCGAAATTCCGGGTACCCGAATTTTCGGGTA
TTGAAAAACCCGTTGTAATTTCGGATATCGATTCTCAAAACCCAAAATTTTAAAAACTCGAATTACCCGACTCGAAAATT
TCGGGTAACCCGAACGCCCACCCCTAGCACGGGCATACACCTAGTCAAGTATGATGTATGATATGATATGATTTGGATAG
CGAGGTAAACGTGGACGTGGCCTTGGCTTCCTGCCTTCCTCCGATCCCTCTCCCCGAGGCGCCTTCCTGCCTTTCTGTCT
CACCAGCCGCTGCCGCTTCCTGGGCATCTCAGTCAAGCACCCCCCCCCCCCCCCCCCCCCCCCCCAAAGGTCGGGGGCCG
CAGCCTGGGGTGCGCGCCCTCGACCCCAATCCGCATACGTGCGCCGGAATCTAGTGGATGCGCGGCAGCAAACTGCCACA
GATTCCTTCTCGCCGCCGCCACGCCATCATTCATGGCGAGGCGTCGCCTGGCGGTACAAGAACCATAATCCATGCTATTC
AACATCCCACACTGCAAAGCACAAGCTCCTGTAGCCGCCGTCGTAGCAGCCATTTCCAGCATCCGCTTCTCCTCCTCCCT
GCCGGCACTGGTTCCGCCGCCTCCGCCCCTGCACGATGAGAACCCGTTCGCTGCGCTCCTCGCCTCTGAGCCCCCACCGC
CGGAGCCTCTCCGCCAGGTGCTCGCCACGGGCGACGTCCACTCCGCGCTCCGCGGCCTCCCGGGCCTCGCGCGCCAGCTG
TTCCGGTGGGCGGAGACTACTCCGTGTGGCTTCCCCCGCTCCGCCTCCGCGTTCGCCGCCGTCCTCATCCCGCTCGCCCG
AGCCAACCACATCCGGGCCGCCTACCCTGTCTCCCTCCGCGCCCTGCACCTCGACCTCCTCCTCCCCCTGGTGTCCCTCC
TGTCCGCTCCCCTCTCTACCGCTCCACGGTCACTATTGAGCCTCCTCTTACGCTTGTCCACCAAGTACTCGAAGGAATGC
AAAGCCCGCGACGCCACGCTCGACACCTGTTCGACGCTGTGCTTGTCCGCCTTCCGCGAGATGGCAAGCCACGGCGTGGC
CCCTGACGTTAAAGACTGCAACCGTGTGCTCCGTGTATTACGTGATGCGGCCAGGTGGGATGACATTTGTGCTGTGCATG
AGGAGATGCTCGAGCTTGGGATTGAGCCCAGTATTGTCACGTACAATACTTTGTTGGATTCTTTCTTGAAGGAGGGAAGG
AAGGACAAGGTTGCCATGCTGCTGAAGGAGATGGAGACCCGGGGGAGTGGTTGCTTGCCAAATGATGTCACGTACAATGT
GGTGATTACTGGGTTGACCAGGAAAGGTGACCTTGAGGAGGCAGCAGAGCTGGTCGAGGGAATGCGGCTGTCCAAGAAGG
CTTCATCCTTCACTTATAACCCACTTATCACTGGGTTGCTTGCAAGGGGCTGTGTCAAAAAGGTATACGATTTGCAGTTG
GAGATGGAGAATGAGGGCATTATGCCTACAGTGGTGACGTACAATGCGATGATTCATGGGCTGCTTCAAAGTGGGCTGGT
AGAGGCTGCACAGGTGAAGTTTGCGGAAATGAGGGCGATGGGCTTGCTACCGGACGTGATCACCTACAATTCGTTGCTAA
ATGGGTATTGTAAGGCAGGTAACTTGAAAGAGGCTCTTTTGTTGTTTGGCGATTTGAGGCGTGCAGGGTTAGCGCCGACA
GTTTTGACCTATAACATTCTTATAGATGGTTATTGTAGATTAGGTGATTTAGAGGAAGCCAGGATATTGAAAGAGGAAAT
GGGAGAGCAAGGTTGTTTGCCCAATGTTTGTACATATACAATTCTCATGAAGGGTTCGCTTAATGTGCGTAGCCTTGCTA
TGGCAAGAGAATTCTTTGATGAGATGCTGAGCAAAGGTTTGCAGCCAGATTGCTTTGCTTATAATACAAGGATTTGTGCG
GAGCTAATCCTAGGGGATATTGCCAGAGCTTTCGAGTTGAGAGAGGTGTTGATGTTGGAAGGCATATCTTCTGATACAGT
GACATACAATATTCTTATTCATGGACTGTGCAAGACTGGTAACCTGAAAGATGCCAAAGAGCTGCAGATGAAGATGGTCA
GTAATGGTTTACAGCCTGACTGTATCACATACACTTGCTTGATTCATGCACATTGTGAAAGGGGACTTCTAAGAGAAGCA
AGAAAGATCTTTAATAACATGATCTCAGATGGTTTGCTACCCTCAGCTGTGACCTTCACTGTTATTATTCATGCATATTG
TAGAAGAGGAAACCTTTATTCAGCATATGGTTGGTTTCGAAAGATGCTGGAGGAAGGAGTTGAACCTAACGAAATAACAT
ATAATGTCCTCATACATGCATTATGCAGGATGGGCAGAACTCAACTGGCTTCTCATCATTTTCATGAGATGCTAGAAAGG
GGATTGGTGGCAAACAAATACACATATACTTTGTTGATAGATGGGAACTGTAAAGTGGGCAACTGGGAAGATGCAATGAG
ATTCTATTTTGAAATGCATCAGAATGGTATTCATCCAGATTATTTAACACATAAAGCCTTGCTCAAGGGATTTGATGGTC
ACGTGCACCATACAATTGAGTACTTGGATAACGTTATTTTGGGTGAATAGATTGATTGACCTCTGAAAGCTTGTCCTTTT
GTCCTCGCTTCGCTGATATCTCCCTGTAAGATATTTCTCGTATATGCATTTTTCTCTTCATCAGTGTCTTGTTACCTAGC
TTTGCTCATGTATCCAACAATATTTCCGTTTTTGTTTTCAGGTCAGAGGTGATAGATGGAGTTCCTGGGCCCTTCATTTC
AAACTGGAAACAAAAAGAGAGTTTGAAATAACATTCTCTGAGTTAAGCTGCTGGGTGCTTTCTAAACGAATAGTTAAGCA
CAAACATGCCTTCCTCTTGCTGGTCAGCGATGGTAGATGGAGCTTGGGGGCAATTCCATCCAAAAGAAGAAAAGCAAGAC

TTTGGGACCATGTTCTTTTAGTCATTCAGCTGGGTGCTTCCTAAATCAGTAGCTGATCACAAACAGGTCTTCCCCTTGCT
GCTTAAACCTTGTCAGAATTCCTTTCTTGGTATCATAGCCATGGAAATATGTTTTCTAGTGAAAACTTGGTGGTAGGCAT
CTGCTCCATTTCTAAAAACCTCTCTTGATACGTCCCCTTTGTTATTTCCTTTTGATAAGGTAACCTGGAAACCTGTTGGC
CTTCAATTCCTCATCCAACTAGCTGAGACAGTAAATGGACATGGTCCTTTGCGGCTGACTTTCTTCAATTATATATAGGA
TTTCTCTTGGTAAGAACAAACCAAGATTTTTTTGTAACGAATAATTCTTGTATCACCATAAATTTTGCCATAGGCATAAG
TCATAGTATATTGACCTGTCAGATATGTAACTGTATTCTCATTCATTGAATCATTTAATGAGAAGATTATTCATTTAAAA
AATTCAGCTCTGTGCCAGCTGTTATTGACATGAACATTTTTTTTGCCAAAAAGGTTCTTTAAATTTTCAGCTCATATCAG
CATTTGTTGTTCACTTGCGTTAAAACTTCAGAGCTCCAGAGTTCGCAAGATGTTGGATCTGAGGTGCAAACCTGTAGCTG
AATGTATGTACTTCATGGTACTTGGTCTTGGTTAGTGATTTTTACAAACTAAGTAGAAGAAAGTGTATGGCCTGCATGCA
AGTGAGTTTTGACTACATGCATAATATCATAATCATGGACAAGCTAATACATCACATGATATGCTTGTACTCGTACGAAA
GATTCAATGGTACATTGGTGCTAACGCCTAACATAGTTTGGTATGCTATTGTCCATAGTTGTTAAGGCATCCGTTAAGCG
TCGCTTAAGCGGTAAGGCGCAACAAGGCGTCCTTGTCGCCTTAGGATGGAGAGTAAGGCGTCCGCCTTGGCCAATATGGC
GTCGCTCTGGCGCCTTGAATCTTGCTAAGGCGTCGCATTGAGGACTCCTTGAAGACCTGTGGCGGCGACGGTGGGGCACA
GGTGGCTGTGGTGGTGCACAGGGCGCAAGCGGCCGCAGCGCGCAGAGCTCTGGAAGGGTGCGACAACAGCATAGGGACAT
GAAGAGAGAGAACCACAGGAGAGGGAGGGAGAAGAACGACAACACCGGGGGAGAGGGAGAAAGAGGGGAGCGGCGGCGTG
GGGGACAAAGGATAGAGGAGGGTGGCGGTGCTAGGGGAGTGGGAGAAAGAGAGCGGCAGCGCGGGGGAGAGGCTTGCTGT
GCGGACAGGGTCAGGGTGGTGGCTGAGAGGGATATGAGAAGTGTAGGTTAG
>GRMZM2G327263_T01 (cDNA)
ATGGAGTCTAAAGAGGAAAATGTTGCACTTACGGTCATCCAAAGAGATAATACATTATCTATAGATGAATCTCTACTAAT
GTCCTTGTCTCATAACAAGCCAAAAACATCGGTGACTACTACAATAGTTCCAAAAGATTATATTTGCACGCAGGGTGATC
TTGCACTCATCGATTGGATTAAAGAAATCCCTTGTGAACCAAGGGTAGAAGTCGTGCTTATTGATGATGCCTTTGTGGAA
AGGAAGTGGATGGAATGCCTATTTGAACCGGACGCATATCTAGGTGATGAGGTGATAGACTGTTACATAAATTTAATAAA
AGCTCAAGAGCATCTAAAATGCCGATCTGGAGGTCGTGTACACATAGAAAATGCTTTTCAGTTCAACTTTCTAAAGCGAG
ACGGTGATGTTGACACTAAAACAGATGAGTTATATCCAAGTAAAGACATGGCACAAATATCTAGCGCCGAAAGAAGGGTT
TTACTTTATCTAGACCATGACATGGTGTTTATTCCAATAAATATCCGAGAAATGCACTGGTATCTTGCCGTGATCAATGC
ACGAAATATGGAGATACAAGTGCTCGACTCACTTGGTACATCATCCGGTCGCAATGACCTCATTGACACTATAAAAGGAC
TCCAAAGACAAATTGATATGGTATCTCAGCGTAAGGAGTTAAAAGATCACAGGTGGCCAGACCTCCGAGTTGCTTCTTGG
CCACTTAGAGAAATAGAAATGGAATATGCAAAGCAAACAGATAGCTCCTCGTGTGGCCTCTTTCTACTAAACTATATCGA
ATACTGGACAGGGGATGAACTATCTGATAATTTTACCCAGGATGACATGTCACACTTTAGGAAAAAATTGGCTGCTATAT
TACTATCTTCAGATATAAACAAGAGAAAGGGGTGTCCGTTATACAAGTATGACAAAGAAGTCGATGCTGGATGCTCCTCT
GATGTTCAGATATTAGATAGTCCCACAAATCCTAAGAAGAGGAAACTACTTTGTGTATCTGAAAAGAGCGAAGTATTGAT
GGAAGATGATGATGGCCCCATCACTCAAGCAGACTTAGAAAAGTGGTTTGTTCATGATTGGGATAAAAGAACTCCTATAA
AAATCTCGACCGATGAGTGCACTAATGAATTTTTACTGAGTGGCCTTTCCACAAAGGACATGCCAGTGACCAAAGCCGAT
TTAATAGATGTTCTATGTGATTACATCATGACAATCCAAGATGATACGGCATTAGAGATGACATGGGTGCGAAGTTTCAA
CCCTTTTAAGATAGAAATATCTGTCAAAGACCTGCAAAACGTATTAAGGGTAAATCTAGATATGACTCTAAAATGTTTTG
ACATGGCTGTTCGATTGTTGGCCATTAAAGAGTCACATATGTCGAAAGATGAAATGATAAAGGATAAAAAACATTATATG
GACACGCGCTTCTGGAGAATGGTTGGTTTTGGTAAACTTCCAAAGTACCATCAGGATCCTACAGCAGAAGAGTTAGCTAA
CACACTCGACTGTTGGCCTTCATTGAACTATTATATCACTGGTTGTAAATATGTTCTTATGCCATGGAAATTCAACGGAT
GCTATGCACTTTTCGTAATCGATCATGGTAAAAAGCACGTAACTTTTATAGACTTTACACCCACTCAAGATTGGTGTAAA
CACATGCCATACAAGAGGTTTGCGGAAGCAATTATTATGGCCTCCAAGAAATATAAGATTGCCTACAATAAGAAACGTTC
TGGATGGGCAGATGATATTTTTAAGTGGGAACATACAATTCGGTCTGGTCTTCCGATGGACTTAAAAGGGGTTAATACAA
GCTACTTCGTTCTACAAGCTATGGTTATGTGGGGGAGTGGTAGACGAATGGAATTTAATAGGTGGACTTCTCTGGATCTT
GCTAAGATGTCTCGTGAAATTAGAGGCAGTCCAGAGATCTTGTTTGCAAGAGAAGTTGCAAGAGCATGCAGAATGGGGAA
CTTTATTTCCTTCTTCCGTCTTGCAAGGAAAGCTACATATTTGGAGGCATGTTTGATGCATGCTCATTTTGCAAAGGAGT

GGTTGGAAGCAAATAAAGACTACCGAGCTATAGTCGATGGTGCGAATATTGCACTATATCAACCAAATTTTGCGGAGGGC
GGTTTCAGTTTGACCCAGCTTATGGAAAATCCATCTAATAGGCATTTGATTGAGACCTGGAGAACAAATGGGGCTTTGTA
TACTTCACCAAGTGGGTCAAATGATGACTGGTCTGCAATGTTGCCGATGTCGAACCCTTCAGCTAATCCCACACAGCTTA
CAATTTTCTATGGTGGATCAGTATGTGTGTATGACTCGGTGCCACCAGAAAAGGCTCAGGCAATCATGCTTATAGCTGCA
GCTGCGGCAGCTGCGGCAGCCACCAAAGGCAGTGCTGCCACTGCTTTTAATCCTCCAATGAGCACTTCTGTAGCAGCTGG
ACAAGCTCAGGTTGTTGCTGACCCTAGCTCAATAAGCAAGCTTCAGGCTGATCTCCCCATTGCCAGGAGGCACTCTCTCC
ATCGCTTCCTTGAGAAACGTCGTGACAGGGTCGTGAGCAAAGCTCCGTACAGCCCCGCCAAGTCGTTCGATGGCATGGAG
TCAGCGGGAATGGAGATGACTGTGGACGGCAAGCAGGGCGCAAGGCCCAGTATCTTGAAAGCACAAGCTCCTGTAGCCGC
CGTCGTAGCAGCCATTTCCAGCATCCGCTTCTCCTCCTCCCTGCCGGCACTGGTTCCGCCGCCTCCGCCCCTGCACGATG
AGAACCCGTTCGCTGCGCTCCTCGCCTCTGAGCCCCCACCGCCGGAGCCTCTCCGCCAGGTGCTCGCCACGGGCGACGTC
CACTCCGCGCTCCGCGGCCTCCCGGGCCTCGCGCGCCAGCTGTTCCGGTGGGCGGAGACTACTCCGTGTGGCTTCCCCCG
CTCCGCCTCCGCGTTCGCCGCCGTCCTCATCCCGCTCGCCCGAGCCAACCACATCCGGGCCGCCTACCCTGTCTCCCTCC
GCGCCCTGCACCTCGACCTCCTCCTCCCCCTGGTGTCCCTCCTGTCCGCTCCCCTCTCTACCGCTCCACGGTCACTATTG
AGCCTCCTCTTACGCTTGTCCACCAAGTACTCGAAGGAATGCAAAGCCCGCGACGCCACGCTCGACACCTGTTCGACGCT
GTGCTTGTCCGCCTTCCGCGAGATGGCAAGCCACGGCGTGGCCCCTGACGTTAAAGACTGCAACCGTGTGCTCCGTGTAT
TACGTGATGCGGCCAGGTGGGATGACATTTGTGCTGTGCATGAGGAGATGCTCGAGCTTGGGATTGAGCCCAGTATTGTC
ACGTACAATACTTTGTTGGATTCTTTCTTGAAGGAGGGAAGGAAGGACAAGGTTGCCATGCTGCTGAAGGAGATGGAGAC
CCGGGGGAGTGGTTGCTTGCCAAATGATGTCACGTACAATGTGGTGATTACTGGGTTGACCAGGAAAGGTGACCTTGAGG
AGGCAGCAGAGCTGGTCGAGGGAATGCGGCTGTCCAAGAAGGCTTCATCCTTCACTTATAACCCACTTATCACTGGGTTG
CTTGCAAGGGGCTGTGTCAAAAAGGTATACGATTTGCAGTTGGAGATGGAGAATGAGGGCATTATGCCTACAGTGGTGAC
GTACAATGCGATGATTCATGGGCTGCTTCAAAGTGGGCTGGTAGAGGCTGCACAGGTGAAGTTTGCGGAAATGAGGGCGA
TGGGCTTGCTACCGGACGTGATCACCTACAATTCGTTGCTAAATGGGTATTGTAAGGCAGGTAACTTGAAAGAGGCTCTT
TTGTTGTTTGGCGATTTGAGGCGTGCAGGGTTAGCGCCGACAGTTTTGACCTATAACATTCTTATAGATGGTTATTGTAG
ATTAGGTGATTTAGAGGAAGCCAGGATATTGAAAGAGGAAATGGGAGAGCAAGGTTGTTTGCCCAATGTTTGTACATATA
CAATTCTCATGAAGGGTTCGCTTAATGTGCGTAGCCTTGCTATGGCAAGAGAATTCTTTGATGAGATGCTGAGCAAAGGT
TTGCAGCCAGATTGCTTTGCTTATAATACAAGGATTTGTGCGGAGCTAATCCTAGGGGATATTGCCAGAGCTTTCGAGTT
GAGAGAGGTGTTGATGTTGGAAGGCATATCTTCTGATACAGTGACATACAATATTCTTATTCATGGACTGTGCAAGACTG
GTAACCTGAAAGATGCCAAAGAGCTGCAGATGAAGATGGTCAGAGGTGATAGATGGAGTTCCTGGGCCCTTCATTTCAAA
CTGGAAACAAAAAGAGAGTTTGAAATAACATTCTCTGAGTTAAGCTGCTGGGTGCTTTCTAAACGAATAGTTAAGCACAA
ACATGCCTTCCTCTTGCTGAGCTCCAGAGTTCGCAAGATGTTGGATCTGAGGTGCAAACCTGTAGCTGAATTTGTTAAGG
CATCCGTTAAGCGTCGCTTAAGCGGTAAGGCGCAACAAGGCGTCCTTGTCGCCTTAGGATGGAGAGGCGCAAGCGGCCGC
AGCGCGCAGAGCTCTGGAAGGGTGCGACAACAGCATAGGGACATGAAGAGAGAGAACCACAGGAGAGGGAGGGAGAAGAA
CGACAACACCGGGGGAGAGGGAGAAAGAGGGGAGCGGCGGCGTGGGGGACAAAGGATAGAGGAGGGTGGCGGTGCTAGGG
GAGTGGGAGAAAGAGAGCGGCAGCGCGGGGGAGAGGCTTGCTGTGCGGACAGGGTCAGGGTGGTGGCTGAGAGGGATATG
AGAAGTGTAGGTTAG
>GRMZM2G327263_P01
MESKEENVALTVIQRDNTLSIDESLLMSLSHNKPKTSVTTTIVPKDYICTQGDLALIDWIKEIPCEPRVEVVLIDDAFVE
RKWMECLFEPDAYLGDEVIDCYINLIKAQEHLKCRSGGRVHIENAFQFNFLKRDGDVDTKTDELYPSKDMAQISSAERRV
LLYLDHDMVFIPINIREMHWYLAVINARNMEIQVLDSLGTSSGRNDLIDTIKGLQRQIDMVSQRKELKDHRWPDLRVASW
PLREIEMEYAKQTDSSSCGLFLLNYIEYWTGDELSDNFTQDDMSHFRKKLAAILLSSDINKRKGCPLYKYDKEVDAGCSS
DVQILDSPTNPKKRKLLCVSEKSEVLMEDDDGPITQADLEKWFVHDWDKRTPIKISTDECTNEFLLSGLSTKDMPVTKAD
LIDVLCDYIMTIQDDTALEMTWVRSFNPFKIEISVKDLQNVLRVNLDMTLKCFDMAVRLLAIKESHMSKDEMIKDKKHYM
DTRFWRMVGFGKLPKYHQDPTAEELANTLDCWPSLNYYITGCKYVLMPWKFNGCYALFVIDHGKKHVTFIDFTPTQDWCK
HMPYKRFAEAIIMASKKYKIAYNKKRSGWADDIFKWEHTIRSGLPMDLKGVNTSYFVLQAMVMWGSGRRMEFNRWTSLDL

AKMSREIRGSPEILFAREVARACRMGNFISFFRLARKATYLEACLMHAHFAKEWLEANKDYRAIVDGANIALYQPNFAEG
GFSLTQLMENPSNRHLIETWRTNGALYTSPSGSNDDWSAMLPMSNPSANPTQLTIFYGGSVCVYDSVPPEKAQAIMLIAA
AAAAAAATKGSAATAFNPPMSTSVAAGQAQVVADPSSISKLQADLPIARRHSLHRFLEKRRDRVVSKAPYSPAKSFDGME
SAGMEMTVDGKQGARPSILKAQAPVAAVVAAISSIRFSSSLPALVPPPPPLHDENPFAALLASEPPPPEPLRQVLATGDV
HSALRGLPGLARQLFRWAETTPCGFPRSASAFAAVLIPLARANHIRAAYPVSLRALHLDLLLPLVSLLSAPLSTAPRSLL
SLLLRLSTKYSKECKARDATLDTCSTLCLSAFREMASHGVAPDVKDCNRVLRVLRDAARWDDICAVHEEMLELGIEPSIV
TYNTLLDSFLKEGRKDKVAMLLKEMETRGSGCLPNDVTYNVVITGLTRKGDLEEAAELVEGMRLSKKASSFTYNPLITGL
LARGCVKKVYDLQLEMENEGIMPTVVTYNAMIHGLLQSGLVEAAQVKFAEMRAMGLLPDVITYNSLLNGYCKAGNLKEAL
LLFGDLRRAGLAPTVLTYNILIDGYCRLGDLEEARILKEEMGEQGCLPNVCTYTILMKGSLNVRSLAMAREFFDEMLSKG
LQPDCFAYNTRICAELILGDIARAFELREVLMLEGISSDTVTYNILIHGLCKTGNLKDAKELQMKMVRGDRWSSWALHFK
LETKREFEITFSELSCWVLSKRIVKHKHAFLLLSSRVRKMLDLRCKPVAEFVKASVKRRLSGKAQQGVLVALGWRGASGR
SAQSSGRVRQQHRDMKRENHRRGREKNDNTGGEGERGERRRGGQRIEEGGGARGVGERERQRGGEACCADRVRVVAERDM
RSVG

>GRMZM2G327263_T02(cDNA)

ATGGAGTCTAAAGAGGAAAATGTTGCACTTACGGTCATCCAAAGAGATAATACATTATCTATAGATGAATCTCTACTAAT
GTCCTTGTCTCATAACAAGCCAAAAACATCGGTGACTACTACAATAGTTCCAAAAGATTATATTTGCACGCAGGGTGATC
TTGCACTCATCGATTGGATTAAAGAAATCCCTTGTGAACCAAGGGTAGAAGTCGTGCTTATTGATGATGCCTTTGTGGAA
AGGAAGTGGATGGAATGCCTATTTGAACCGGACGCATATCTAGGTGATGAGGTGATAGACTGTTACATAAATTTAATAAA
AGCTCAAGAGCATCTAAAATGCCGATCTGGAGGTCGTGTACACATAGAAAATGCTTTTCAGTTCAACTTTCTAAAGCGAG
ACGGTGATGTTGACACTAAAACAGATGAGTTATATCCAAGTAAAGACATGGCACAAATATCTAGCGCCGAAAGAAGGGTT
TTACTTTATCTAGACCATGACATGGTGTTTATTCCAATAAATATCCGAGAAATGCACTGGTATCTTGCCGTGATCAATGC
ACGAAATATGGAGATACAAGTGCTCGACTCACTTGGTACATCATCCGGTCGCAATGACCTCATTGACACTATAAAAGGAC
TCCAAAGACAAATTGATATGGTATCTCAGCGTAAGGAGTTAAAAGATCACAGGTGGCCAGACCTCCGAGTTGCTTCTTGG
CCACTTAGAGAAATAGAAATGGAATATGCAAAGCAAACAGATAGCTCCTCGTGTGGCCTCTTTCTACTAAACTATATCGA
ATACTGGACAGGGGATGAACTATCTGATAATTTTACCCAGGATGACATGTCACACTTTAGGAAAAAATTGGCTGCTATAT
TACTATCTTCAGATATAAACAAGAGAAAGGGGTGTCCGTTATACAAGTATGACAAAGAAGTCGATGCTGGATGCTCCTCT
GATGTTCAGATATTAGATAGTCCCACAAATCCTAAGAAGAGGAAACTACTTTGTGTATCTGAAAAGAGCGAAGTATTGAT
GGAAGATGATGATGGCCCCATCACTCAAGCAGACTTAGAAAAGTGGTTTGTTCATGATTGGGATAAAAGAACTCCTATAA
AAATCTCGACCGATGAGTGCACTAATGAATTTTTACTGAGTGGCCTTTCCACAAAGGACATGCCAGTGACCAAAGCCGAT
TTAATAGATGTTCTATGTGATTACATCATGACAATCCAAGATGATACGGCATTAGAGATGACATGGGTGCGAAGTTTCAA
CCCTTTTAAGATAGAAATATCTGTCAAAGACCTGCAAAACGTATTAAGGGTAAATCTAGATATGACTCTAAAATGTTTTG
ACATGGCTGTTCGATTGTTGGCCATTAAAGAGTCACATATGTCGAAAGATGAAATGATAAAGGATAAAAAACATTATATG
GACACGCGCTTCTGGAGAATGGTTGGTTTTGGTAAACTTCCAAAGTACCATCAGGATCCTACAGCAGAAGAGTTAGCTAA
CACACTCGACTGTTGGCCTTCATTGAACTATTATATCACTGGTTGTAAATATGTTCTTATGCCATGGAAATTCAACGGAT
GCTATGCACTTTTCGTAATCGATCATGGTAAAAAGCACGTAACTTTTATAGACTTTACACCCACTCAAGATTGGTGTAAA
CACATGCCATACAAGAGGTTTGCGGAAGCAATTATTATGGCCTCCAAGAAATATAAGATTGCCTACAATAAGAAACGTTC
TGGATGGGCAGATGATATTTTTAAGTGGGAACATACAATTCGGTCTGGTCTTCCGATGGACTTAAAAGGGGTTAATACAA
GCTACTTCGTTCTACAAGCTATGGTTATGTGGGGGAGTGGTAGACGAATGGAATTTAATAGGTGGACTTCTCTGGATCTT
GCTAAGATGTCTCGTGAAATTAGAGGCAGTCCAGAGATCTTGTTTGCAAGAGAAGTTGCAAGAGCATGCAGAATGGGGAA
CTTTATTTCCTTCTTCCGTCTTGCAAGGAAAGCTACATATTTGGAGGCATGTTTGATGCATGCTCATTTTGCAAAGGAGT
GGTTGGAAGCAAATAAAGACTACCGAGCTATAGTCGATGGTGCGAATATTGCACTATATCAACCAAATTTTGCGGAGGGC
GGTTTCAGTTTGACCCAGCTTATGGAAAATCCATCTAATAGGCATTTGATTGAGACCTGGAGAACAAATGGGGCTTTGTA
TACTTCACCAAGTGGGTCAAATGATGACTGGTCTGCAATGTTGCCGATGTCGAACCCTTCAGCTAATCCCACACAGCTTA
CAATTTTCTATGGTGGATCAGTATGTGTGTATGACTCGGTGCCACCAGAAAAGGCTCAGGCAATCATGCTTATAGCTGCA

GCTGCGGCAGCTGCGGCAGCCACCAAAGGCAGTGCTGCCACTGCTTTTAATCCTCCAATGAGCACTTCTGTAGCAGCTGG
ACAAGCTCAGGTTGTTGCTGACCCTAGCTCAATAAGCAAGCTTCAGGCTGATCTCCCCATTGCCAGGAGGCACTCTCTCC
ATCGCTTCCTTGAGAAACGTCGTGACAGGGTCGTGAGCAAAGCTCCGTACAGCCCCGCCAAGTCGTTCGATGGCATGGAG
TCAGCGGGAATGGAGATGACTGTGGACGGCAAGCAGGGCGCAAGGCCCAGTATCTTGAAAGCACAAGCTCCTGTAGCCGC
CGTCGTAGCAGCCATTTCCAGCATCCGCTTCTCCTCCTCCCTGCCGGCACTGGTTCCGCCGCCTCCGCCCCTGCACGATG
AGAACCCGTTCGCTGCGCTCCTCGCCTCTGAGCCCCCACCGCCGGAGCCTCTCCGCCAGGTGCTCGCCACGGGCGACGTC
CACTCCGCGCTCCGCGGCCTCCCGGGCCTCGCGCGCCAGCTGTTCCGGTGGGCGGAGACTACTCCGTGTGGCTTCCCCCG
CTCCGCCTCCGCGTTCGCCGCCGTCCTCATCCCGCTCGCCCGAGCCAACCACATCCGGGCCGCCTACCCTGTCTCCCTCC
GCGCCCTGCACCTCGACCTCCTCCTCCCCCTGGTGTCCCTCCTGTCCGCTCCCCTCTCTACCGCTCCACGGTCACTATTG
AGCCTCCTCTTACGCTTGTCCACCAAGTACTCGAAGGAATGCAAAGCCCGCGACGCCACGCTCGACACCTGTTCGACGCT
GTGCTTGTCCGCCTTCCGCGAGATGGCAAGCCACGGCGTGGCCCCTGACGTTAAAGACTGCAACCGTGTGCTCCGTGTAT
TACGTGATGCGGCCAGGTGGGATGACATTTGTGCTGTGCATGAGGAGATGCTCGAGCTTGGGATTGAGCCCAGTATTGTC
ACGTACAATACTTTGTTGGATTCTTTCTTGAAGGAGGGAAGGAAGGACAAGGTTGCCATGCTGCTGAAGGAGATGGAGAC
CCGGGGGAGTGGTTGCTTGCCAAATGATGTCACGTACAATGTGGTGATTACTGGGTTGACCAGGAAAGGTGACCTTGAGG
AGGCAGCAGAGCTGGTCGAGGGAATGCGGCTGTCCAAGAAGGCTTCATCCTTCACTTATAACCCACTTATCACTGGGTTG
CTTGCAAGGGGCTGTGTCAAAAAGGTATACGATTTGCAGTTGGAGATGGAGAATGAGGGCATTATGCCTACAGTGGTGAC
GTACAATGCGATGATTCATGGGCTGCTTCAAAGTGGGCTGGTAGAGGCTGCACAGGTGAAGTTTGCGGAAATGAGGGCGA
TGGGCTTGCTACCGGACGTGATCACCTACAATTCGTTGCTAAATGGGTATTGTAAGGCAGGTAACTTGAAAGAGGCTCTT
TTGTTGTTTGGCGATTTGAGGCGTGCAGGGTTAGCGCCGACAGTTTTGACCTATAACATTCTTATAGATGGTTATTGTAG
ATTAGGTGATTTAGAGGAAGCCAGGATATTGAAAGAGGAAATGGGAGAGCAAGGTTGTTTGCCCAATGTTTGTACATATA
CAATTCTCATGAAGGGTTCGCTTAATGTGCGTAGCCTTGCTATGGCAAGAGAATTCTTTGATGAGATGCTGAGCAAAGGT
TTGCAGCCAGATTGCTTTGCTTATAATACAAGGATTTGTGCGGAGCTAATCCTAGGGGATATTGCCAGAGCTTTCGAGTT
GAGAGAGGTGTTGATGTTGGAAGGCATATCTTCTGATACAGTGACATACAATATTCTTATTCATGGACTGTGCAAGACTG
GTAACCTGAAAGATGCCAAAGAGCTGCAGATGAAGATGGTCAGTAATGGTTTACAGCCTGACTGTATCACATACACTTGC
TTGATTCATGCACATTGTGAAAGGGGACTTCTAAGAGAAGCAAGAAAGATCTTTAATAACATGATCTCAGATGGTTTGCT
ACCCTCAGCTGTGACCTTCACTGTTATTATTCATGCATATTGTAGAAGAGGAAACCTTTATTCAGCATATGGTTGGTTTC
GAAAGATGCTGGAGGAAGGAGTTGAACCTAACGAAATAACATATAATGTCCTCATACATGCATTATGCAGGATGGGCAGA
ACTCAACTGGCTTCTCATCATTTTCATGAGATGCTAGAAAGGGGATTGGTGGCAAACAAATACACATATACTTTGTTGAT
AGATGGGAACTGTAAAGTGGGCAACTGGGAAGATGCAATGAGATTCTATTTTGAAATGCATCAGAATGGTATTCATCCAG
ATTATTTAACACATAAAGCCTTGCTCAAGGGATTTGATGGTCACGTGCACCATACAATTGAGTACTTGGATAACGTTATT
TTGGGTGAATAGATTGATTGACCTCTGAAAGCTTGTCCTTTTGTCCTCGCTTCGCTGATATCTCCCTGTCAGAGGTGATA
GATGGAGTTCCTGGGCCCTTCATTTCAAACTGGAAACAAAAAGAGAGTTTGAAATAACATTCTCTGAGTTAAGCTGCTGG
GTGCTTTCTAAACGAATAGTTAAGCACAAACATGCCTTCCTCTTGCTGGTCAGCGATGGTAGATGGAGCTTGGGGGCAAT
TCCATCCAAAAGAAGAAAAGCAAGACTTTGGGACCATGTTCTTTTAGTCATTCAGCTGGGTGCTTCCTAAATCAGTAGCT
GATCACAAACAGGTCTTCCCCTTGCTGCTTAAACCTTGTCAGAATTCCTTTCTTGGTATCATAGCCATGGAAATATGTTT
TCTAGTGAAAACTTGGTGGTAGGCATCTGCTCCATTTCTAAAAACCTCTCTTGATACGTCCCCTTTGTTATTTCCTTTTG
ATAAGGTAACCTGGAAACCTGTTGGCCTTCAATTCCTCATCCAACTAGCTGAGACAGTAAATGGACATGGTCCTTTGCGG
CTGACTTTCTTCAATTATATATAGGATTTCTCTTGGTAAGAACAAACCAAGATTTTTTTGTAACGAATAATTCTTGTATC
ACCATAAATTTTGCCATAGGCATAAGTCATAGTATATTGACCTGTCAGATATGTAACTGTATTCTCATTCATTGAATCAT
TTAATGAGAAGATTATTCATTTAAAAAA

>GRMZM2G327263_P02

MESKEENVALTVIQRDNTLSIDESLLMSLSHNKPKTSVTTTIVPKDYICTQGDLALIDWIKEIPCEPRVEVVLIDDAFVE
RKWMECLFEPDAYLGDEVIDCYINLIKAQEHLKCRSGGRVHIENAFQFNFLKRDGDVDTKTDELYPSKDMAQISSAERRV
LLYLDHDMVFIPINIREMHWYLAVINARNMEIQVLDSLGTSSGRNDLIDTIKGLQRQIDMVSQRKELKDHRWPDLRVASW

PLREIEMEYAKQTDSSSCGLFLLNYIEYWTGDELSDNFTQDDMSHFRKKLAAILLSSDINKRKGCPLYKYDKEVDAGCSS
DVQILDSPTNPKKRKLLCVSEKSEVLMEDDDGPITQADLEKWFVHDWDKRTPIKISTDECTNEFLLSGLSTKDMPVTKAD
LIDVLCDYIMTIQDDTALEMTWVRSFNPFKIEISVKDLQNVLRVNLDMTLKCFDMAVRLLAIKESHMSKDEMIKDKKHYM
DTRFWRMVGFGKLPKYHQDPTAEELANTLDCWPSLNYYITGCKYVLMPWKFNGCYALFVIDHGKKHVTFIDFTPTQDWCK
HMPYKRFAEAIIMASKKYKIAYNKKRSGWADDIFKWEHTIRSGLPMDLKGVNTSYFVLQAMVMWGSGRRMEFNRWTSLDL
AKMSREIRGSPEILFAREVARACRMGNFISFFRLARKATYLEACLMHAHFAKEWLEANKDYRAIVDGANIALYQPNFAEG
GFSLTQLMENPSNRHLIETWRTNGALYTSPSGSNDDWSAMLPMSNPSANPTQLTIFYGGSVCVYDSVPPEKAQAIMLIAA
AAAAAAATKGSAATAFNPPMSTSVAAGQAQVVADPSSISKLQADLPIARRHSLHRFLEKRRDRVVSKAPYSPAKSFDGME
SAGMEMTVDGKQGARPSILKAQAPVAAVVAAISSIRFSSSLPALVPPPPPLHDENPFAALLASEPPPPEPLRQVLATGDV
HSALRGLPGLARQLFRWAETTPCGFPRSASAFAAVLIPLARANHIRAAYPVSLRALHLDLLLPLVSLLSAPLSTAPRSLL
SLLLRLSTKYSKECKARDATLDTCSTLCLSAFREMASHGVAPDVKDCNRVLRVLRDAARWDDICAVHEEMLELGIEPSIV
TYNTLLDSFLKEGRKDKVAMLLKEMETRGSGCLPNDVTYNVVITGLTRKGDLEEAAELVEGMRLSKKASSFTYNPLITGL
LARGCVKKVYDLQLEMENEGIMPTVVTYNAMIHGLLQSGLVEAAQVKFAEMRAMGLLPDVITYNSLLNGYCKAGNLKEAL
LLFGDLRRAGLAPTVLTYNILIDGYCRLGDLEEARILKEEMGEQGCLPNVCTYTILMKGSLNVRSLAMAREFFDEMLSKG
LQPDCFAYNTRICAELILGDIARAFELREVLMLEGISSDTVTYNILIHGLCKTGNLKDAKELQMKMVSNGLQPDCITYTC
LIHAHCERGLLREARKIFNNMISDGLLPSAVTFTVIIHAYCRRGNLYSAYGWFRKMLEEGVEPNEITYNVLIHALCRMGR
TQLASHHFHEMLERGLVANKYTYTLLIDGNCKVGNWEDAMRFYFEMHQNGIHPDYLTHKALLKGFDGHVHHTIEYLDNVI
LGE
>GRMZM2G327263_T03(cDNA)
ATGGAGTCTAAAGAGGAAAATGTTGCACTTACGGTCATCCAAAGAGATAATACATTATCTATAGATGAATCTCTACTAAT
GTCCTTGTCTCATAACAAGCCAAAAACATCGGTGACTACTACAATAGTTCCAAAAGATTATATTTGCACGCAGGGTGATC
TTGCACTCATCGATTGGATTAAAGAAATCCCTTGTGAACCAAGGGTAGAAGTCGTGCTTATTGATGATGCCTTTGTGGAA
AGGAAGTGGATGGAATGCCTATTTGAACCGGACGCATATCTAGGTGATGAGGTGATAGACTGTTACATAAATTTAATAAA
AGCTCAAGAGCATCTAAAATGCCGATCTGGAGGTCGTGTACACATAGAAAATGCTTTTCAGTTCAACTTTCTAAAGCGAG
ACGGTGATGTTGACACTAAAACAGATGAGTTATATCCAAGTAAAGACATGGCACAAATATCTAGCGCCGAAAGAAGGGTT
TTACTTTATCTAGACCATGACATGGTGTTTATTCCAATAAATATCCGAGAAATGCACTGGTATCTTGCCGTGATCAATGC
ACGAAATATGGAGATACAAGTGCTCGACTCACTTGGTACATCATCCGGTCGCAATGACCTCATTGACACTATAAAAGGAC
TCCAAAGACAAATTGATATGGTATCTCAGCGTAAGGAGTTAAAAGATCACAGGTGGCCAGACCTCCGAGTTGCTTCTTGG
CCACTTAGAGAAATAGAAATGGAATATGCAAAGCAAACAGATAGCTCCTCGTGTGGCCTCTTTCTACTAAACTATATCGA
ATACTGGACAGGGGATGAACTATCTGATAATTTTACCCAGGATGACATGTCACACTTTAGGAAAAAATTGGCTGCTATAT
TACTATCTTCAGATATAAACAAGAGAAAGGGGTGTCCGTTATACAAGTATGACAAAGAAGTCGATGCTGGATGCTCCTCT
GATGTTCAGATATTAGATAGTCCCACAAATCCTAAGAAGAGGAAACTACTTTGTGTATCTGAAAAGAGCGAAGTATTGAT
GGAAGATGATGATGGCCCCATCACTCAAGCAGACTTAGAAAAGTGGTTTGTTCATGATTGGGATAAAAGAACTCCTATAA
AAATCTCGACCGATGAGTGCACTAATGAATTTTTACTGAGTGGCCTTTCCACAAAGGACATGCCAGTGACCAAAGCCGAT
TTAATAGATGTTCTATGTGATTACATCATGACAATCCAAGATGATACGGCATTAGAGATGACATGGGTGCGAAGTTTCAA
CCCTTTTAAGATAGAAATATCTGTCAAAGACCTGCAAAACGTATTAAGGGTAAATCTAGATATGACTCTAAAATGTTTTG
ACATGGCTGTTCGATTGTTGGCCATTAAAGAGTCACATATGTCGAAAGATGAAATGATAAAGGATAAAAAACATTATATG
GACACGCGCTTCTGGAGAATGGTTGGTTTTGGTAAACTTCCAAAGTACCATCAGGATCCTACAGCAGAAGAGTTAGCTAA
CACACTCGACTGTTGGCCTTCATTGAACTATTATATCACTGGTTGTAAATATGTTCTTATGCCATGGAAATTCAACGGAT
GCTATGCACTTTTCGTAATCGATCATGGTAAAAAGCACGTAACTTTTATAGACTTTACACCCACTCAAGATTGGTGTAAA
CACATGCCATACAAGAGGTTTGCGGAAGCAATTATTATGGCCTCCAAGAAATATAAGATTGCCTACAATAAGAAACGTTC
TGGATGGGCAGATGATATTTTTAAGTGGGAACATACAATTCGGTCTGGTCTTCCGATGGACTTAAAAGGGGTTAATACAA
GCTACTTCGTTCTACAAGCTATGGTTATGTGGGGGAGTGGTAGACGAATGGAATTTAATAGGGATGCAAAAATTATTCGA
AGGAACTTTGTAATTGATCTATTAAGTTATGAGGAGAATTCATGCCACTATGCTATCCCTCCAAATATACAACAACGACT

TATCAGTATCGCTAAAAAAGATTAGATTAATGT
>GRMZM2G327263_P03
MESKEENVALTVIQRDNTLSIDESLLMSLSHNKPKTSVTTTIVPKDYICTQGDLALIDWIKEIPCEPRVEVVLIDDAFVE
RKWMECLFEPDAYLGDEVIDCYINLIKAQEHLKCRSGGRVHIENAFQFNFLKRDGDVDTKTDELYPSKDMAQISSAERRV
LLYLDHDMVFIPINIREMHWYLAVINARNMEIQVLDSLGTSSGRNDLIDTIKGLQRQIDMVSQRKELKDHRWPDLRVASW
PLREIEMEYAKQTDSSSCGLFLLNYIEYWTGDELSDNFTQDDMSHFRKKLAAILLSSDINKRKGCPLYKYDKEVDAGCSS
DVQILDSPTNPKKRKLLCVSEKSEVLMEDDDGPITQADLEKWFVHDWDKRTPIKISTDECTNEFLLSGLSTKDMPVTKAD
LIDVLCDYIMTIQDDTALEMTWVRSFNPFKIEISVKDLQNVLRVNLDMTLKCFDMAVRLLAIKESHMSKDEMIKDKKHYM
DTRFWRMVGFGKLPKYHQDPTAEELANTLDCWPSLNYYITGCKYVLMPWKFNGCYALFVIDHGKKHVTFIDFTPTQDWCK
HMPYKRFAEAIIMASKKYKIAYNKKRSGWADDIFKWEHTIRSGLPMDLKGVNTSYFVLQAMVMWGSGRRMEFNRDAKIIR
RNFVIDLLSYEENSCHYAIPPNIQQRLISIAKKD
AGPv4: NO
AGPv5:
>Zm00001e020904
A**ATG**TTGCCGATGTCGAACCCTTCAGCTAATCCCACACAGCTTACAATTTTCTATGGTGGATCAGTATGTG
TGTATGACTCGGTGCCACCAGAAAAGGTAAATATTATCTTCACGCCATTTACAGATATCTTGTCCTTCTGTA
CCATCTGTTAAAATACACATGCTAATACACTAATTTGGCATTGACAGGCTCAGGCAATCATGCTTATAGCT
GCAGCTGCGGCAGCTGCGGCAGCCACCAAAGGCAGTGCTGCCACTGCTTTTAATCCTCCAATGGTACAT
ACAGCCACTGTCTCCCCAGCAGCAGTCTTCTCTCCTGTGCTTACACGGTCTCCATCACTGCAGAGCACTT
CTGTAGCAGCTGGACAAGCTCAGGTTGTTGCTGACCCTAGCTCAATAAGCAAGCTTCAGGCTGGTAAGA
CCACTGCATCTTTGTGTAAAAATCCATAAATGGAATACCTTTTCCAGTGTTGACATTGTCCAACACGTCTT
TAAACTATCTTGATCTGTTGGCCAGATCTCCCCATTGCCAGGAGGCACTCTCTCCATCGCTTCCTTGAGAA
ACGTCGTGACAGGTTAGCATGATACATGAGAGCAACAGGGCTCTGATATTCTGAATCTTATTGTTAGGCCT
CTTATTCTACAATCTACATGTTTAAAACTTTTGTTATAAACTGCTGTATGTGGTACCACACTAATTTTTTCCC
CGACTATTGGCACCACAGTAATCAACAGTATATTTTGTACCGTATTCTGTTTTTTGTCCTCCAGGCAGGGT
AAAAATAAAATAAAATTTGCTGTTATAGCATTTAAATGATTGCACTTTCTGCTGACAAGTGCTCAATGACA
TCCCTAAAAAAATCTATAATTTCTTCACAGAAAGATTGGTAGAGAGCCTGCTTCCATAATACAACATATAG
CTGCAAATAGCCTTGGATCATGTAACTCCCTTGAGATTTTTTTTTAAAAAATCTGCAAATCGATATTAACA
CAAGACAGAGTTCTGGGACCAGCAGTAGATACAAAAACCAACATTACCCCCATTTAGCTGCTCCAAATA
CCATGTTTTTCCCCTGTTAGGAAAATAACATATTTGAGAATCGGGATGTTCCTGTGTATTATCCTGTTCTAT
TTGGAAAAGCTGGCCACTTAAAGATCCAGTCTCAAGGTTGCAAACTAGCACCATTTTTTTTCTGATGTAA
GCTTTAGATGCCTGTTTTTGCCTTATCTTAGAACTGAGTTTAGAATTGTTTCTTAGTATAACTTTTTTTTATC
GTGGATCTACTGATTTCAAGTGATTAAATAAATGATGGCACGAAACAGGGTCGTGAGCAAAGCTCCGTAC
AGCCCCGCCAAGTCGTTCGATGGCATGGAGTCAGCGGAATGGAGATGACTGTGGACGGCAAGCAGGG
CGCAAGGCCCAGTATCTTGAAAGGTTGGGGAGCCGCGAAGTCTGAAGTC**TGA**ACGGTGAAAG (the shallow area indicated the introns)
>Zm00001e020904
MLPMSNPSANPT**QLTIFYGGSVCVYDSVPPEKAQAIMLIAA**AAAAAAATKGSAATAFNPPMSTSVAAGQAQVVADPSSIS
KLQA**DLPIARRHSLHRFLEKRRDRVVSKAPY**SPAKSFDGMESAGMEMTVDGKQGARPSILKGWGAAKSEV

ZmJAZ38（*TIFY47*）

B73 RefGen_v3:
GRMZM2G314145 (zim25 - ZIM-transcription factor 25)

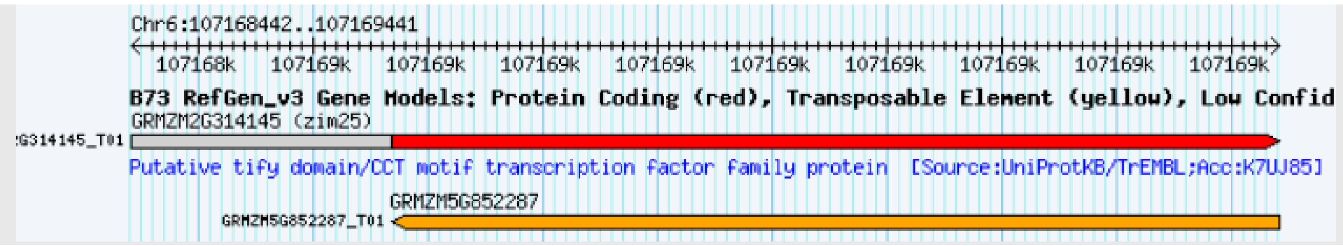

>Zm00001d037082_P001

>GRMZM2G314145 Genomic DNA

TGGCTCCCTCTGCCCCCTCCCGTTCACAGTTCTCCTCCCAACACGCACGCTAGCAACCGCTCCCTCCCTCCACCTCCGGG
CGCCCCGACCACCACGACCCTGAGCCTGATGCCCGGCGCGGACGTCGGCGCCGACGAGCAGCAGGGGCCGGTCGCCGCGA
GGGCCGAGGAGATGCGCGGGCCCGCCCCCATCGCGGCGCCGCCGCTCACCATCTTCTACGCGCAGCATGGTGGTGGTGTT
CGAGGACTTCCCGGCGGAGAAGGCGGCCGAGGTCATGCGCCTCGCGACCGGCGATGACCTGTCCATCGCCCGGAAGGCGT
CGTTGCAGCGGTTCCTGGCCAAGCGCAAGGCCTGTCTCGTCGAGCACGCGCCCTACGCCCGCCCGTCGTCCCCCGCGGAG
GAGCCGGAGAAGAAGACGGTCAAGCCGGCCTCGACCTTGGCCTCCTGGCTCGGGCTTGGCAGCACGGAAGCCGACCGCCT
CACCATCGCGCTGTTCTGCGGTTCTGCCCACACTCGACAGGTGGGCCCATCTTTGAGTGTCTGTTTCCTGCTGCAGCTCG
CCCGCAGAACCGCGCCCGCCGCCCCACTCCCAAGTTTTTGTCGTGCTCGCTACCTGGTCTGCCCCACCGTCTTCCCCAG
ATCGTGCGAGACCTGGGTCCACCCAGTCCCCACTCCCTCCCCCCCCCTCAACCTCCAAGCCTAGGTAACCTCCGCCTCAC
GCTCTCTAGCTCCGCCCCATCCACTGCCCCGTCATTGCCGATGGTGCCGCCAGATGCCACAAGGAAGGGGGAGGGATGCG
GATGGGGACGATCTACGTCGTCGCGGCACTGGCGCTCGACGTGGCGGTGGACTGCGAGGACAACCTCATCCCCGCAAAAG
GGTCAGCTCTGGCACTTCGGCATCAACGGTGACTATTGTTGGAACTTCTTGGTCAGCGAAACGATCTTGTTCAGCCTGAC
GCAGCAGAGGACCTGCTCAGGGAGCTCCGACTGCTTCTAG

>GRMZM2G314145_T01(cDNA)

TGGCTCCCTCTGCCCCCTCCCGTTCACAGTTCTCCTCCCAACACGCACGCTAGCAACCGCTCCCTCCCTCCACCTCCGGG
CGCCCCGACCACCACGACCCTGAGCCTGATGCCCGGCGCGGACGTCGGCGCCGACGAGCAGCAGGGGCCGGTCGCCGCGA
GGGCCGAGGAGATGCGCGGGCCCGCCCCCATCGCGGCGCCGCCGCTCACCATCTTCTACGCGCAGCATGGTGGTGGTGTT
CGAGGACTTCCCGGCGGAGAAGGCGGCCGAGGTCATGCGCCTCGCGACCGGCGATGACCTGTCCATCGCCCGGAAGGCGT
CGTTGCAGCGGTTCCTGGCCAAGCGCAAGGCCTGTCTCGTCGAGCACGCGCCCTACGCCCGCCCGTCGTCCCCCGCGGAG
GAGCCGGAGAAGAAGACGGTCAAGCCGGCCTCGACCTTGGCCTCCTGGCTCGGGCTTGGCAGCACGGAAGCCGACCGCCT
CACCATCGCGCTGTTCTGCGGTTCTGCCCACACTCGACAGGTGGGCCCATCTTTGAGTGTCTGTTTCCTGCTGCAGCTCG
CCCGCAGAACCGCGCCCGCCGCCCCACTCCCAAGTTTTTGTCGTGCTCGCTACCTGGTCTGCCCCACCGTCTTCCCCAG
ATCGTGCGAGACCTGGGTCCACCCAGTCCCCACTCCCTCCCCCCCCCTCAACCTCCAAGCCTAGGTAACCTCCGCCTCAC
GCTCTCTAGCTCCGCCCCATCCACTGCCCCGTCATTGCCGATGGTGCCGCCAGATGCCACAAGGAAGGGGGAGGGATGCG
GATGGGGACGATCTACGTCGTCGCGGCACTGGCGCTCGACGTGGCGGTGGACTGCGAGGACAACCTCATCCCCGCAAAAG
GGTCAGCTCTGGCACTTCGGCATCAACGGTGACTATTGTTGGAACTTCTTGGTCAGCGAAACGATCTTGTTCAGCCTGAC
GCAGCAGAGGACCTGCTCAGGGAGCTCCGACTGCTTCTAG

>GRMZM2G314145_P01

MVVVFEDFPAEKAAEVMRLATGDDLSIARKASLQRFLAKRKACLVEHAPYARPSSPAEEPEKKTVKPASTLASWLGLGST
EADRLTIALFCGSAHTRQVGPSLSVCFLLQLARRTAPAAPLPSFLSCSLPGLPHRLPQIVRDLGPPSPHSLPPPQPPSLG
NLRLTLSSSAPSTAPSLPMVPPDATRKGEGCGWGRSTSSRHWRSTWRWTARTTSSPQKGQLWHFGINGDYCWNFLVSETI
LFSLTQQRTCSGSSDCF

AGPv4:

Zm00001d037082 (zim25 - ZIM-transcription factor 25)

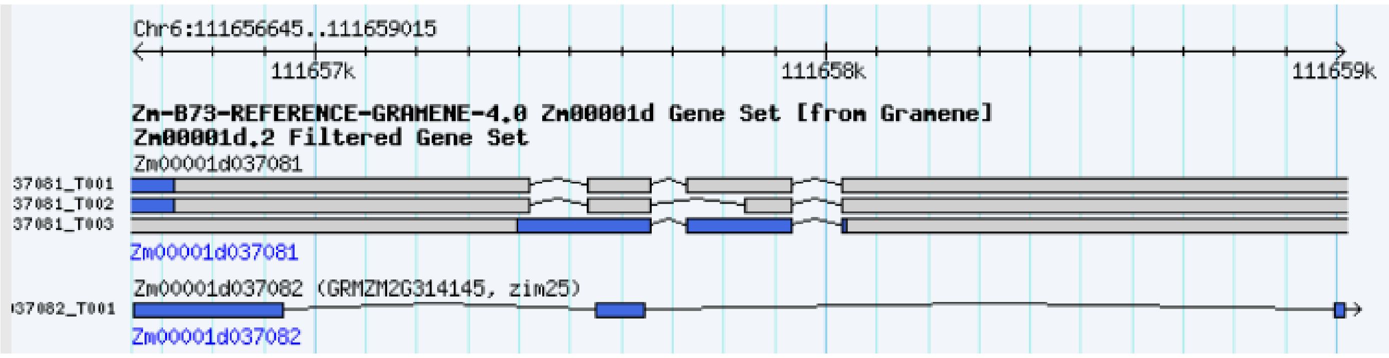

>Zm00001d037082 Genomic DNA

ATGGTGGTGGTGTTCGAGGACTTCCCGGCGGAGAAGGCGGCCGAGGTCATGCGCCTCGCGACCGGCGATGACCTGTCCAT
CGCCCGGAAGGCGTCGTTGCAGCGGTTCCTGGCCAAGCGCAAGGCCTGTCTCGTCGAGCACGCGCCCTACGCCCGCCCGT
CGTCCCCCGCGGAGGAGCCGGAGAAGAAGACGGTCAAGCCGGCCTCGACCTTGGCCTCCTGGCTCGGGCTTGGCAGCACG
GAAGCCGACCGCCTCACCATCGCGCTGTTCTGCGGTTCTGCCCACACTCGACAGGTGGGCCCATCTTTGAGTGTCTGTTT
CCTGCTGCAGCTCGCCCGCAGAACCGCGCCCGCCGCCCCACTCCCAAGTTTTTGTCGTGCTCGCTACCTGGTCTGCCCC
ACCGTCTTCCCCAGATCGTGCGAGACCTGGGTCCACCCAGTCCCCACTCCCTCCCCCCCCCTCAACCTCCAAGCCTAGGT
AACCTCCGCCTCACGCTCTCTAGCTCCGCCCCATCCACTGCCCCGTCATTGCCGATGGTGCCGCCAGATGCCACAAGGAA
GGGGGAGGGATGCGGATGGGGACGATCTACGTCGTCGCGGCACTGGCGCTCGACGTGGCGGTGGACTGCGAGGACAACCT
CATCCCCGCAAAAGGGTCAGCTCTGGCACTTCGGCATCAACGGTGACTATTGTTGGAACTTCTTGGTCAGCGAAACGATC
TTGTTCAGCCTGACGCAGCAGAGGACCTGCTCAGGGAGCTCCGACTGCTTCTAGGCCTGCAAGATCACAGCGGTCAACTG
GAATATAGACAAACAAGGAGAAGCTAAAGAAGACTGTGATAGATAGCTTCTGCTTCTTCTCTGTTGGAGGATCTAAATGG
AATCTGACCTGGATCGTTAACCCCAGATCGAGGATGCCGTTCTTGAGGCGTGCCCTGAACGAATCCAGCCCCTTCCTAGC
AATGTAGCTGAACCGGTGGATGTCAAGGTCCACCTCAAGGTAGTCCTCTCCCTGGCGATGCAAAGATGGTAGATTCGAAG
GTAGGTGGTTTATCTGAAGCATCGATACGAACAAGGAACGACGTGGTTTGGGTACCTCGTAGAAGCTGTGCTGGGGCCGC
GAGAGGACTGGCTTCTCGTTGTACGCCTGGACGAGCTTCCTCTCCGTAGAGCCCAGCTGGAGGTCGTCCGGTTGACCAGC
CCGGCCATGATCTTGAGCCGGTCTCTGTAGCTGACGGTGGATTCTGACGTGAACCCTTTCACCTTCTCAGTTTCGTCCTC
AAAAAACCTCTGTTGTCGTCAGGAAAGAGTCATTTATTAGACTTCGACTTAAACCACAGGAAGTACAAGTATTAAAGATG
GCGGATTGACTGAAAAAAACGGCTAACCATCATAGACTCCTTGAAATGTTCGGAAACCTCCTTGTCGTAGCATTCGGACA
CCCTGAAGTACAGGACGAGGCTCAAGACTGTGTTTGTTCATGTAAGATTGTAAGAACTTTGTGATTAGTGTATCCATTGC
ATTAATAAGGGTCAAGTTAAGCTGATCCATCTACTGCCTTTAGATATATGTATTGAGATGCCACCGTTATGCTTGTGTGT
TCGTTTTTCTAAGTTGCAGCATCTTTAATTTGTTATTACTCCTTTATAGCATACTATTTAGGTAGGCTTCTGGATGTTGG
GAAGAAATGACCAATTCAGTCAATGATTAGTATAGTATCACTATGGGCTATGTGTTCTCCCTTAAAATCAGAGAGCAGTA
CAATTCAAATATGAAGGGCTTGGCTTGATAGTTGGTACTTAATATCATGACAAAAGAGAAGTCACGTTGCTATATTCTGT
TGCATTTCTTCTGAATATTGAAGTACAATTAGTACAATTTCACCATGGGCTATATGTTCTTCCACAAAATTGGGTATATG
CATGGTTCGAATTTTAAGAACATGGTTATTCTAGAGATGAGAATCTCTTGATACTTTATACTTGATATCATGAGAAGGGA
GACATCATGTTGTAATAACTAACACAATTCTTCTGAATATTAGAAAAAAATGACTAGCACACATTGCTGGTCATCTTGTT
TCTTCTTCATGCTTCATACTCTGTGCTGAGATATGTAGAAAATACTGAGTGATCAATACAATATTAGAAAGATTTTGCGC
GTGTAGGTATCAGTTGATACTTCTCACTTTCCTAAATATAAGTGGAAAATTTTTACACGTGTAGCTGCCAGTTGATACTC
GTCTACCTTGCATAAGTTGGGAGCAGTTCATCATCCAATTAAACCAGGTCAACTCATGCTCTGATATGTAATGTTTTTTA
CTAATCACTTCATCTTTGACTTCTTTTCAGGGTTTTTTCATGGATCTTTAA

>Zm00001d037082_T001(cDNA)

ATGGTGGTGGTGTTCGAGGACTTCCCGGCGGAGAAGGCGGCCGAGGTCATGCGCCTCGCGACCGGCGATGACCTGTCCAT
CGCCCGGAAGGCGTCGTTGCAGCGGTTCCTGGCCAAGCGCAAGGCCTGTCTCGTCGAGCACGCGCCCTACGCCCGCCCGT
CGTCCCCCGCGGAGGAGCCGGAGAAGAAGACGGTCAAGCCGGCCTCGACCTTGGCCTCCTGGCTCGGGCTTGGCAGCACG

GAAGCCGACCGCCTCACCATCGCGCTGTTCTGCGGTTCTGCCCACACTCGACAGATCGAGGATGCCGTTCTTGAGGCGTG
CCCTGAACGAATCCAGCCCCTTCCTAGCAATGTAGCTGAACCGGTGGATGTCAAGGTCCACCTCAAGGGTTTTTTCATGG
ATCTTTAA
>Zm00001d037082_P001
MVVVFEDFPAEKAAEVMRLATGDDLSIARKASLQRFLAKRKACLVEHAPYARPSSPAEEPEKKTVKPASTLASWLGLGST
EADRLTIALFCGSAHTRQIEDAVLEACPERIQPLPSNVAEPVDVKVHLKGFFMDL
