# Supplementary material for: An updated census of the maize TIFY family
Source: PLoS One. 2021 Feb 23;16(2):e0247271. doi: 10.1371/journal.pone.0247271 (PMC7901733; doi:10.1371/journal.pone.0247271)
Supplement: Attachment — Submitted filename: Review of Manuscript.docx [file pone.0247271.s008.docx]

Review of Manuscript Number: PONE-D-20-28741

An updated census of the maize TIFY family

The authors characterize a family of transcription factor genes and build upon previous studies by Bai et al. 2011, Zhang et al. 2015, and Zhou et al. 2015. This study includes the identification of new genes in this family, includes mapping analyses, analyzes splicing variants, examines conserved domains, examines gene expression, and enhances phylogenic analyses.

This work is well documented and presents important novel information that would be of interest to the readers of PLOS One.

There is one major issue with the classification of this gene family in that is does not build upon the earlier work of the Grassius project (Yilmaz et al. 2009. Plant Physiol 149:171-180; <https://doi.org/10.1104/pp.108.128579>). This project had identified 36 genes in this “super” family (see: <https://grassius.org/family.php?family=ZIM&species=Maize>) and used the symbols *zim1* - *zim36* (ZIM = zinc-finger inflorescence meristem) to describe them. While the authors state that the symbol “ZIM” had more recently been replaced by the symbol “TIFY”, this nomenclature developed in 2009 has precedence, represents the official symbols for these 36 genes and is how they are listed at MaizeGDB.org.

Of these 36 genes, all but 4 overlap with the 43 genes predicted in this manuscript.

The overlapping genes are as follows:

MaizeGDB symbol	v4 model	NCBI LOC	v3 model	Table 1 symbol

[zim1](https://www.maizegdb.org/gene_center/gene/2776302)	Zm00001d020409 100273108 GRMZM2G126507	ZmJAZ17

[zim2](https://www.maizegdb.org/gene_center/gene/2776313)	Zm00001d013331 100281895 GRMZM2G065896	ZmZML1

[zim3](https://www.maizegdb.org/gene_center/gene/2776324)	Zm00001d033049 100284979 GRMZM2G145458	ZmJAZ6

[zim4](https://www.maizegdb.org/gene_center/gene/2776332)	Zm00001d048263 100284433 GRMZM2G036351	ZmJAZ21

[zim5](https://www.maizegdb.org/gene_center/gene/2776333)	Zm00001d009438 103636978 GRMZM2G054689	ZmJAZ24

[zim7](https://www.maizegdb.org/gene_center/gene/2776335)	Zm00001d013855 103626102 GRMZM2G063632	ZmJAZ25

[zim8](https://www.maizegdb.org/gene_center/gene/2776336)	Zm00001d004277 103648809 GRMZM2G171830	ZmJAZ10

[zim9](https://www.maizegdb.org/gene_center/gene/2776337)	Zm00001d035382 103629067 GRMZM2G338829	ZmJAZ16

[zim10](https://www.maizegdb.org/gene_center/gene/2776303)	Zm00001d014253 100286212 GRMZM2G173596	ZmJAZ15

[zim12](https://www.maizegdb.org/gene_center/gene/2776305)	Zm00001d006860 100282471 GRMZM2G101769	ZmJAZ12

[zim13](https://www.maizegdb.org/gene_center/gene/2776306)	Zm00001d005813 100191257 GRMZM2G005954	ZmJAZ11

[zim14](https://www.maizegdb.org/gene_center/gene/484866)	Zm00001d048268 100284541 GRMZM2G036288	ZmJAZ22

[zim15](https://www.maizegdb.org/gene_center/gene/2776308)	Zm00001d005726 103647411 GRMZM2G114681	ZmJAZ26

[zim16](https://www.maizegdb.org/gene_center/gene/2776309)	Zm00001d027901 100283151 GRMZM2G445634	ZmJAZ2

[zim18](https://www.maizegdb.org/gene_center/gene/955780)	Zm00001d033050 100284894 GRMZM2G145412	ZmJAZ5

[zim19](https://www.maizegdb.org/gene_center/gene/2776312)	Zm00001d034536 103644213 GRMZM2G382794	ZmJAZ7

[zim20](https://www.maizegdb.org/gene_center/gene/2776314)	Zm00001d036494 100282175 GRMZM2G080509	ZmZML3

[zim21](https://www.maizegdb.org/gene_center/gene/2776315)	Zm00001d033048 103643602 GRMZM2G024680	ZmJAZ4

[zim22](https://www.maizegdb.org/gene_center/gene/2776316)	Zm00001d028313 100284291 GRMZM2G110131	ZmTIFY1

[zim23](https://www.maizegdb.org/gene_center/gene/2776317)	Zm00001d022139 100276383 GRMZM2G089736	ZmJAZ20

[zim24](https://www.maizegdb.org/gene_center/gene/2776318)	Zm00001d029448 100276585 GRMZM2G117513	ZmJAZ3

[zim26](https://www.maizegdb.org/gene_center/gene/2776320)	Zm00001d027899 100284778 GRMZM2G343157	ZmJAZ1

[zim27](https://www.maizegdb.org/gene_center/gene/2776321)	Zm00001d027900 100281912 GRMZM5G838098	ZmJAZ27

[zim28](https://www.maizegdb.org/gene_center/gene/273175)	Zm00001d020614 100384222 GRMZM2G116614	ZmJAZ18

[zim29](https://www.maizegdb.org/gene_center/gene/2776323)	Zm00001d014249 103626248 GRMZM2G064775	ZmJAZ14

[zim30](https://www.maizegdb.org/gene_center/gene/2776325)	Zm00001d014250 103626249, AC197764.4_FG003	ZmJAZ28

[zim31](https://www.maizegdb.org/gene_center/gene/2776326)	Zm00001d021274 103633081 GRMZM2G066020	ZmJAZ19

zim32	Zm00001d002029 100384736 GRMZM2G086920	ZmJAZ8

[zim33](https://www.maizegdb.org/gene_center/gene/2776328)	Zm00001d003903 100274370 GRMZM2G145407	ZmJAZ9

[zim34](https://www.maizegdb.org/gene_center/gene/2776329)	Zm00001d026477 100383759 GRMZM2G143402	ZmJAZ23

[zim35](https://www.maizegdb.org/gene_center/gene/2776330)	Zm00001d050365 100285509 GRMZM2G151519	ZmJAZ13

[zim36](https://www.maizegdb.org/gene_center/gene/2776331)	Zm00001d014656 100277669 GRMZM2G058479	ZmZML2

The 11 genes described in this manuscript that have not as yet been assigned gene symbols in MaizeGDB:

Zm00001d016316 (not in NCBI, but overlaps with 103628582)	ZmJAZ29

Zm00001d019692 (NCBI 103633876)	ZmJAZ30

Zm00001d021924 (NCBI 103633348)	ZmJAZ31

Zm00001d024455 (not in NCBI, but overlaps with 103641214; v3 GRMZM2G442458)	ZmJAZ32

Zm00001d033972 (NCBI 103643992)	ZmJAZ33

Zm00001d041045 (NCBI 103652103)	ZmJAZ34

Zm00001d044708 (NCBI 103651689)	ZmJAZ35

Zm00001d046270 (NCBI 103638584)	ZmJAZ36

Zm00001d033523 (NCBI 100283192)	ZmZML4

Zm00001d004173 (not in NCBI, but overlaps with 103646667; v3 GRMZM2G028313)	ZmTIFY2

Zm00001d051615 (not in NCBI, but overlaps with 103653981; v3 GRMZM2G022514)	ZmTIFY3

There are also four zim genes that were described by the Grassius project that are not described in this manuscript:

In MaizeGDB and Grassius, but not listed in Table 1:

[zim6](https://www.maizegdb.org/gene_center/gene/2776334)	Zm00001d048264 109942667 GRMZM2G036349

[zim11](https://www.maizegdb.org/gene_center/gene/2776304)	Zm00001d049005 100275133 GRMZM2G122160

[zim17](https://www.maizegdb.org/gene_center/gene/2776310)	Zm00001d044707 103651688 GRMZM2G327263

[zim25](https://www.maizegdb.org/gene_center/gene/2776319)	Zm00001d037082 GRMZM2G314145 (overlaps with NCBI 103631324 and 103631325)

In order for this manuscript to be fully acceptable, at the very least, the authors need to modify Table 1 with an extra column to include the official ‘zim’ gene symbols. The text should also be modified appropriately and the Grassius project and/or the Yilmaz et al. 2009 article cited.

While it may be preferable to change the symbols used in this manuscript to conform with the official symbols (and add zim37-zim47 to accommodate the additional genes described), since the authors are consistent with those used previously by Bai et al. 2011, and Zhou et al. 2015 (but differs from the nomenclature used by Zhang et al. 2015), it would be acceptable to do the modification of Table 1 as suggested. This would have the added benefit of showing how members of the “super-family” are divided into the JAZ, TIFY and ZML subfamilies.

The authors should also examine zim6, zim11, zim17, and zim25 to determine if it would be appropriate to include these in their analyses; or explain in the text of the manuscript why these four do not fit into the ZIM/TIFY super-family.

Additional minor edits that the authors should make:

Page 3, Line 10: “In the recently years” should be “In recent years”

Page 4, Line 14: “Recently, the maize reference genome”. **Note:** this is not so recent. In fact, B73 v5 has been released (<https://www.ncbi.nlm.nih.gov/bioproject/PRJEB32225>) and is now beginning to be used. I suggest that this be changed from “Recently” to “In 2016”

Page 4, Line 16: “In this study, we utilized this latest version of” should be “In this study, we utilized version 4 of”
