# Supplementary material for: An updated census of the maize TIFY family
Source: PLoS One. 2021 Feb 23;16(2):e0247271. doi: 10.1371/journal.pone.0247271 (PMC7901733; doi:10.1371/journal.pone.0247271)
Supplement: Attachment — Submitted filename: rebutal letter.2021.1.24.docx [file pone.0247271.s010.docx]

Dear reviewers and editors,

# We are extremely grateful to the comments of the reviewers who paid a lot of time to review again the revised version of “An updated census of the maize TIFY family” (PONE-D-20-28741R1). Based on the information the reviewer provided this time, the revised manuscript contained one error in the caption of Table 1. This is an inadvertent mistake of our revision process. Now, it is corrected.

# We thank a lot for the comments from reviewers and editor.

# With the best

Yuanxin Yan Prof.

State Key Laboratory of Crop Genetics and Germplasm Enhancement

College of Agriculture

Nanjing Agricultural University

Weigang #1, Nanjing,Jiangsu Province, China.

Post code 210095

e-mail: [yuanxin.yan@njau.edu.cn](mailto:yuanxin.yan@njau.edu.cn)

tel: 0086 25 84399517

**Responses to the reviewer’s comments**

**Comments to the Author**

1. If the authors have adequately addressed your comments raised in a previous round of review and you feel that this manuscript is now acceptable for publication, you may indicate that here to bypass the “Comments to the Author” section, enter your conflict of interest statement in the “Confidential to Editor” section, and submit your "Accept" recommendation.

Reviewer #1: All comments have been addressed

2. Is the manuscript technically sound, and do the data support the conclusions?

The manuscript must describe a technically sound piece of scientific research with data that supports the conclusions. Experiments must have been conducted rigorously, with appropriate controls, replication, and sample sizes. The conclusions must be drawn appropriately based on the data presented.

Reviewer #1: Yes

3. Has the statistical analysis been performed appropriately and rigorously?

Reviewer #1: N/A

4. Have the authors made all data underlying the findings in their manuscript fully available?

The [PLOS Data policy](http://www.plosone.org/static/policies.action#sharing) requires authors to make all data underlying the findings described in their manuscript fully available without restriction, with rare exception (please refer to the Data Availability Statement in the manuscript PDF file). The data should be provided as part of the manuscript or its supporting information, or deposited to a public repository. For example, in addition to summary statistics, the data points behind means, medians and variance measures should be available. If there are restrictions on publicly sharing data—e.g. participant privacy or use of data from a third party—those must be specified.

Reviewer #1: Yes

5. Is the manuscript presented in an intelligible fashion and written in standard English?

PLOS ONE does not copyedit accepted manuscripts, so the language in submitted articles must be clear, correct, and unambiguous. Any typographical or grammatical errors should be corrected at revision, so please note any specific errors here.

Reviewer #1: Yes

6. Review Comments to the Author

Please use the space provided to explain your answers to the questions above. You may also include additional comments for the author, including concerns about dual publication, research ethics, or publication ethics. (Please upload your review as an attachment if it exceeds 20,000 characters)

Reviewer #1: All of the comments I made for the previous version have been addressed. However, in the caption of Table 1: "Five TIFY genes have no typical TIFY domain but include Jas motif." This was not modified. Should this now be modified in light of the four additional genes being presented?
Other than this, I do not see any additional issues. This work is well documented and presents important novel information that would be of interest to the readers of PLOS One. The manuscript should be accepted.

Re: This is a careless mistake of the last revision. It is corrected now. Thank a lot.

7. PLOS authors have the option to publish the peer review history of their article ([what does this mean?](https://journals.plos.org/plosone/s/editorial-and-peer-review-process#loc-peer-review-history)). If published, this will include your full peer review and any attached files.


If you choose “no”, your identity will remain anonymous but your review may still be made public.


**Do you want your identity to be public for this peer review?** For information about this choice, including consent withdrawal, please see our [Privacy Policy](https://www.plos.org/privacy-policy).

Reviewer #1: No

[NOTE: If reviewer comments were submitted as an attachment file, they will be attached to this email and accessible via the submission site. Please log into your account, locate the manuscript record, and check for the action link "View Attachments". If this link does not appear, there are no attachment files.]

While revising your submission, please upload your figure files to the Preflight Analysis and Conversion Engine (PACE) digital diagnostic tool, <https://pacev2.apexcovantage.com/>. PACE helps ensure that figures meet PLOS requirements. To use PACE, you must first register as a user. Registration is free. Then, login and navigate to the UPLOAD tab, where you will find detailed instructions on how to use the tool. If you encounter any issues or have any questions when using PACE, please email PLOS at [figures@plos.org](mailto:figures@plos.org). Please note that Supporting Information files do not need this step.
